# Supplementary material for: Nickel-catalyzed electrophiles-controlled enantioselective reductive arylative cyclization and enantiospecific reductive alkylative cyclization of 1,6-enynes
Source: Nat Commun. 2024 Feb 27;15:1787. doi: 10.1038/s41467-024-45617-0 (PMC10899222; doi:10.1038/s41467-024-45617-0)
Supplement: Supplementary file 1 — Supplementary Information [file 41467_2024_45617_MOESM1_ESM.pdf]

Supplementary Information for

Nickel-Catalyzed Electrophiles-Controlled Enantioselective Reductive Arylative Cyclization and Enantiospecific Reductive Alkylative Cyclization of 1,6-Enynes

Wenfeng Liu,[1,3] Yunxin Xing,[2,3] Denghong Yan,[2,3] Wangqing Kong,[1,*] and Kun Shen[2,*]

[1]The Institute for Advanced Studies, Wuhan University, Wuhan 430072, China

[2]Department of Radiology, Zhongnan Hospital of Wuhan University, School of Pharmaceutical Sciences, Wuhan University, Wuhan 430071, China

[3]These authors contributed equally: Wenfeng Liu, Yunxin Xing, Denghong Yan

E-mail: wqkong@whu.edu.cn; kun.shen@whu.edu.cn

Table of Contents

1. General Information S1

2. General Procedures S2

3. Synthesis of Starting Materials S4

4. Optimization Details S43

5. Exploring the Effect of Leaving Groups S46

6. Exploring the Effect of NaI S47

7. Exploring the Effect of Alkene Geometry S48

8. Characterization Data of Products S51

9. Synthetic Transformations and Applications S158

10. Mechanistic Studies S168

11. X-Ray Crystallographic Data S177

12. NMR Spectra S201

13. Supplementary References S303

1. General Information

Nuclear magnetic resonance (NMR) spectroscopy measurements were carried out at room temperature. ^{1}H NMR, ^{13}C NMR, and ^{19}F NMR experiments were carried out using Bruker ADVANCE III (600 MHz) or JNM-ECZ400S/L1 (400 MHz) spectrometers. Chemical shifts (δ) are reported in ppm relative to the residual solvent peak with corresponding coupling constants (J) in Hertz (Hz) and multiplicities (s: singlet, d: doublet, t: triplet, q: quartet, m: multiplet and combinations of these and app.: apparent multiplicities). High-resolution mass spectra (HRMS) were recorded on Thermo Fisher Orbitrap Elite mass spectrometer. Enantiomeric excesses were determined with a SHIMADZU LC-20ADXR system using chiral stationary phase columns (DAICEL) by comparing the samples with the corresponding racemic samples. Column and elution details were specified in each entry.

Materials and Methods: Commercially available reagents and ligands were purchased from Sigma Aldrich, Alfa Aesar, and Strem Chemicals and unless otherwise stated were used without further purification. $NiBr_2{\cdot}DME$, and NiI_2 were bought from Strem Chemicals. All reactions dealing with air- or moisture-sensitive compounds were performed in the argon-filled glove box or by standard Schlenk techniques in oven-dried reaction vessels under argon atmosphere. Solvents were purchased in HPLC quality, degassed by purging thoroughly with argon and dried over 4 Å activated molecular sieves. More sensitive compounds were stored in a desiccator or in a glove-box if required. Reactions were monitored by thin layer chromatography (TLC) using glass 0.25 mm silica gel plates. Compounds were visualized by UV-light at 254 nm and by dipping the plates in an aqueous potassium permanganate solution followed by heating. Flash column chromatography was performed over silica gel (200-400 mesh).

2. General Procedures

2.1 General procedure for the enantioselective reductive arylative cyclization

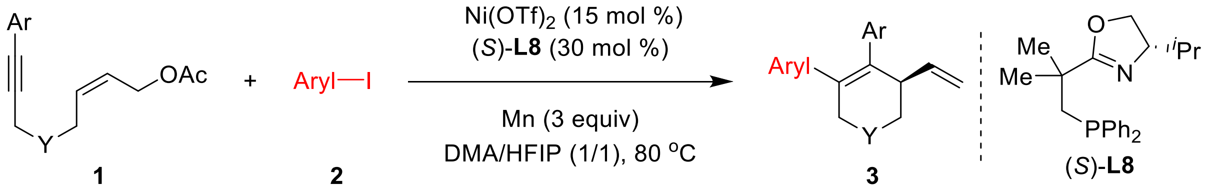

An oven-dried sealed tube equipped with a PTFE-coated stir bar was charged with $Ni(OTf)_2$ (0.03 mmol, 10.6 mg), (*S*)-**L8** (0.06 mmol, 21.2 mg) and anhydrous DMA/HFIP (1:1, 2 mL). This reaction mixture was stirred at room temperature for 1 hour in an argon-filled glovebox. Mn (0.6 mmol, 33.0 mg), 1,6-enyne **1** (0.2 mmol), aryl iodide **2** (0.4 mmol) and DMA/HFIP (1:1, 2 mL) were then added. The sealed tube was sealed and removed from the glovebox. The reaction mixture was allowed to stir at 80 °C for 12 h. The reaction was quenched by the addition of H_2O (10 mL) and EtOAc (20 mL). The organic layer was separated and the aqueous layer was extracted with EtOAc (20 mL × 3). The combined organic layers were washed with brine, dried over Na_2SO_4, filtered and concentrated. Purification by chromatography on silica gel, eluting with PE/EtOAc (50/1~5/1) to give the desired products **3**.

2.2 General procedure for the enantiospecific reductive alkylative cyclization

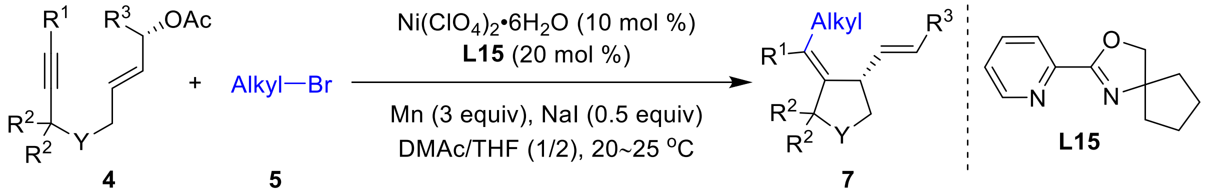

An oven-dried sealed tube equipped with a PTFE-coated stir bar was charged with $Ni(ClO)_2•6H_2O$ (0.01 mmol, 3.7 mg), **L15** (0.02 mmol, 4.0 mg), NaI (0.05mmol, 7.5 mg) and anhydrous DMA (0.67 mL). This reaction mixture was stirred at room temperature for 1 hour in an argon-filled glovebox. 1,6-Enyne **4** (0.1 mmol), alkyl bromide **5** (0.2 mmol), Mn powder (0.3 mmol, 16.5 mg), and anhydrous THF (1.33 mL) were then added. The sealed tube was sealed and removed from the glovebox. The reaction mixture was allowed to stir at 20 °C until the reaction was completed (monitored by TLC). The reaction was quenched by the addition of saturated aqueous solution of NH_4Cl (10 mL) and EtOAc (20 mL). The organic layer was

separated and the aqueous layer was extracted with EtOAc (20 mL × 3). The combined organic layers were washed with brine, dried over Na_2SO_4, filtered and concentrated. Purification by chromatography on silica gel, eluting with PE/EtOAc (20/1~5/1) to give the desired products **7**.

3. Synthesis of Starting Materials

3.1 Procedures for the synthesis of 1,6-enynes (*Z*)-1a and (*E*)-1a

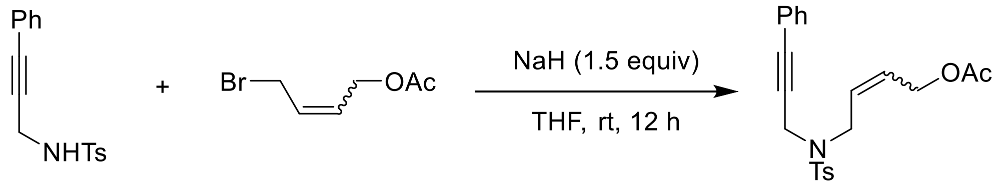

S1 **(*Z*)-S2** or **(*E*)-S2** **(*Z*)-1a** or **(*E*)-1a**

To a suspension of sodium hydride (60% dispersion in mineral oil, 3.01 g, 75 mmol) in THF (130 mL) was added sulfonamide **S1** (15.03 g, 50 mmol) at 0 °C. The resulting mixture was stirred at 0 °C for 30 min before a solution of (*Z*)-4-bromobut-2-en-1-yl acetate ((*Z*)-**S2**) (10.62 g, 55 mmol)[1] in THF (20 mL) was added. The reaction mixture was stirred at room temperature for 12 h, and then was quenched with a saturated aqueous solution of NH_4Cl. The organic layer was separated and the aqueous layer was extracted with EtOAc. The combined organic layers were dried over Na_2SO_4, filtered and concentrated. Purification by column chromatography (25% EtOAc in petroleum ether) gave (*Z*)-*N*-(3-phenylprop-2-ynyl)-*N*-(4-acetoxy-2-butenyl)-*p*-methylphenylsulfonamide ((*Z*)-**1a)** as a yellow solid (16.87 g, 85% yield).

^{1}H NMR (400 MHz, $CDCl_3$): δ 7.78 (d, *J* = 8.4 Hz, 2H), 7.29–7.23 (m, 5H), 7.09–7.07 (m, 2H), 5.86–5.80 (m, 1H), 5.71–5.64 (m, 1H), 4.67 (d, *J* = 6.8 Hz, 2H), 4.31 (s, 2H), 3.99 (d, *J* = 7.2 Hz, 2H), 2.33 (s, 3H), 2.00 (s, 3H);

^{13}C NMR (100 MHz, $CDCl_3$): δ 170.6, 143.6, 135.4, 131.4, 129.5, 129.2, 128.4, 128.0, 127.9, 127.7, 121.8, 85.7, 81.3, 59.6, 43.2, 36.8, 21.3, 20.7; IR (KBr): 3442, 1732, 1640, 1348, 1250, 1163, 764, 659, 582, 542 cm^{-1};

HRMS (m/z) Calcd for ($C_{22}H_{23}NO_4SNa$) ($[M+Na]^+$): 420.1240; found: 420.1240.

To a suspension of sodium hydride (60% dispersion in mineral oil, 0.24 g, 6 mmol) in THF (20 mL) was added sulfonamide **S1** (1.42 g, 5 mmol) at 0 °C. The resulting mixture was stirred at room temperature for 30 min before a solution of (*E*)-4-bromobut-2-en-1-yl acetate ((*E*)-**S2**) (1.45 g, 7.5 mmol)[2] in THF (10 mL) was added. The reaction mixture was allowed to stir at 60 °C overnight, and then was quenched with a saturated aqueous solution of NH_4Cl (20 mL). The organic layer was separated and the aqueous layer was extracted with EtOAc (30 mL × 3).

The combined organic layers were dried over Na_2SO_4, filtered and concentrated. Purification by column chromatography gave (*E*)-4-((4-methyl-N-(3-phenylprop-2-yn-1-yl)phenyl)sulfonamido)but-2-en-1-yl acetate (**(*E*)-1a**) as a yellow solid (1.59 g, 80% yield).

^{1}H NMR (400 MHz, $CDCl_3$): δ 7.82–7.75 (m, 2H), 7.32–7.23 (m, 5H), 7.10–7.04 (m, 2H), 5.87 (dt, *J* = 15.2 5.6 Hz, 1H), 5.75 (dt, *J* = 15.6, 6.4 Hz, 1H), 4.59 (dd, *J* = 5.6, 0.8 Hz, 2H), 4.32 (s, 2H), 3.92 (d, *J* = 6.0 Hz, 2H), 2.35 (s, 3H), 2.08 (s, 3H);

^{13}C NMR (100 MHz, $CDCl_3$): δ 170.4, 143.4, 135.5, 131.2, 129.4, 129.1, 128.2, 127.9, 127.6, 121.8, 85.6, 81.3, 63.6, 47.8, 36.7, 21.2, 20.6; IR (KBr): 3457, 3063, 2924, 1736, 1597, 1491, 1443, 1347, 1228, 1163, 1092, 738 cm^{-1};

HRMS (m/z) Calcd for ($C_{22}H_{23}NO_4SNa$) ($[M+Na]^+$): 420.1240; found: 420.1248.

3.2 Procedures for the synthesis of 1,6-enynes 1b-1k

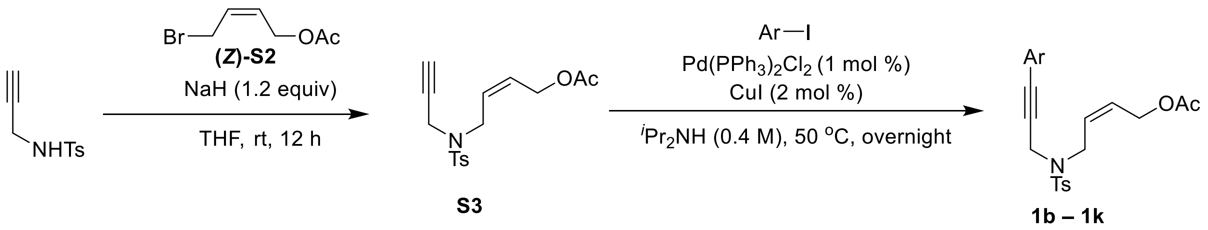

Step I: To a suspension of sodium hydride (60% dispersion in mineral oil, 7.20 g, 180 mmol) in THF (200 mL) was added a solution of 4-methyl-*N*-(prop-2-yn-1-yl)benzenesulfonamide (31.39 g, 150 mmol) in THF (150 mL) at 0 °C. The resulting mixture was stirred at 0 °C for 30 min before (*Z*)-4-bromobut-2-en-1-yl acetate ((*Z*)-**S2**) (34.75 g, 180 mmol) in THF (50 mL) was added. The reaction mixture was allowed to stir at room temperature for 12 h, and then was quenched with a saturated aqueous solution of NH_4Cl. The organic layer was separated and the aqueous layer was extracted with EtOAc. The combined organic layers were dried over Na_2SO_4, filtered and concentrated. Purification by column chromatography gave (*Z*)-*N*-(prop-2-ynyl)-N-(4-acetoxy-2-butenyl)-*p*-methylphenylsulfonamide (**S3**) as a yellow solid (47.60 g, 98% yield).

Step II: To a 100 mL round-bottom flask was added **S3** (20 mmol), $Pd(PPh_3)_2Cl_2$ (0.2 mmol, 1 mol %) and CuI (0.4 mmol, 2 mol %). The flask was degassed and refilled with N_2 for three times. Then diisopropylamine (50 mL) and aryl iodide (24 mmol, 1.2 equiv) were added via

syringe under N_2. The resulting mixture was stirred at 50 °C under N_2 overnight. The reaction was quenched with a saturated aqueous solution of NH_4Cl (30 mL). The organic layer was separated and the aqueous layer was extracted with EtOAc (20 mL × 3). The combined organic layers were washed with brine, dried over Na_2SO_4, filtered and concentrated. Purification by column chromatography gave the corresponding 1,6-enynes **1b**-**1k**.

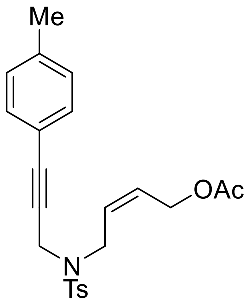

Chemical Formula: $C_{23}H_{25}NO_4S$
Exact Mass: 411.1504

Purification by column chromatography (25% EtOAc in petroleum ether) gave (*Z*)-*N*-(3-(4-methylphenyl)prop-2-ynyl)-*N*-(4-acetoxy-2-butenyl)-*p*-methylphenylsulfonamide (**1b**) as an off-white solid (2.83 g, 69% yield, 10 mmol scale).

^{1}H NMR (400 MHz, $CDCl_3$): δ 7.78 (d, *J* = 8.2 Hz, 2H), 7.27 (d, *J* = 8.1 Hz, 2H), 7.05 (d, *J* = 8.0 Hz, 2H), 6.97 (d, *J* = 8.0 Hz, 2H), 5.82 (dt, *J* = 10.8, 6.8 Hz, 1H), 5.66 (dt, *J* = 10.8, 7.6 Hz, 1H), 4.67 (d, *J* = 6.7 Hz, 2H), 4.30 (s, 2H), 3.98 (d, *J* = 7.2 Hz, 2H), 2.34 (s, 3H), 2.32 (s, 3H), 2.00 (s, 3H);

^{13}C NMR (100 MHz, $CDCl_3$): δ 170.5, 143.5, 138.5, 135.4, 131.3, 129.5, 129.1, 128.7, 127.9, 127.7, 118.7, 85.8, 80.5, 59.6, 43.1, 36.8, 21.3 (2C), 20.7;

IR (KBr): 3446, 1748, 1342, 1234, 1160, 900, 817, 764, 673, 575 cm^{-1};

HRMS-ESI (m/z) Calcd for ($C_{23}H_{25}NO_4SNa$) ([M+Na]$^+$): 434.1397; found: 434.1402.

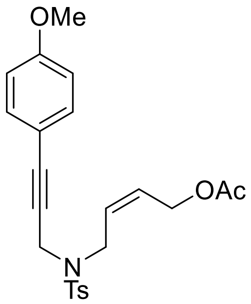

Chemical Formula: $C_{23}H_{25}NO_5S$
Exact Mass: 427.1453

Purification by column chromatography (25% EtOAc in petroleum ether) gave (*Z*)-*N*-(3-(4-methoxylphenyl)prop-2-ynyl)-*N*-(4-acetoxy-2-butenyl)-*p*-methylphenylsulfonamide (**1c**) as a yellow oil (2.51 g, 59% yield, 10 mmol scale).

^{1}H NMR (400 MHz, $CDCl_3$): δ 7.74 (d, *J* = 8.2 Hz, 2H), 7.24 (d, *J* = 8.1 Hz, 2H), 6.99 (d, *J* = 8.8 Hz, 2H), 6.74 (d, *J* = 8.8 Hz, 2H), 5.83 (dt, *J* = 11.2, 6.8 Hz, 1H), 5.65 (dt, *J* = 10.8, 7.2 Hz, 1H), 4.64 (d, *J* = 6.8 Hz, 2H), 4.26 (s, 2H), 3.95 (d, *J* = 7.3 Hz, 2H), 3.76 (s, 3H), 2.32 (s, 3H), 1.98 (s, 3H);

^{13}C NMR (100 MHz, $CDCl_3$): δ 170.6, 159.6, 143.5, 135.5, 132.7, 129.5, 129.1, 128.0, 127.7,

113.9, 113.6, 85.6, 79.8, 59.6, 55.1, 43.1, 36.9, 21.3, 20.7;

IR (neat): 2930, 1738, 1605, 1510, 1347, 1161, 1030, 900, 835, 671, 575 cm^{-1};

HRMS-ESI (m/z) Calcd for ($C_{23}H_{25}NO_5SNa$) ($[M+Na]^+$): 450.1346; found: 450.1351.

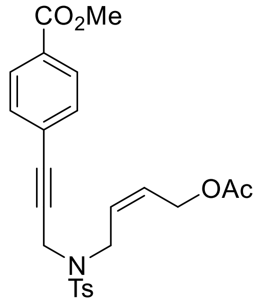

Chemical Formula: $C_{24}H_{25}NO_6S$
Exact Mass: 455.1403

Purification by column chromatography (25% EtOAc in petroleum ether) gave (*Z*)-*N*-(3-(4-methoxycarbonylphenyl)prop-2-ynyl)-*N*-(4-acetoxy-2-butenyl)-*p*-methylphenylsulfonamide (**1d**) as a yellow solid (8.33 g, 91% yield).

^{1}H NMR (400 MHz, $CDCl_3$): δ 7.91 (d, *J* = 8.3 Hz, 2H), 7.76 (d, *J* = 8.2 Hz, 2H), 7.27 (s, 2H), 7.12 (d, *J* = 8.3 Hz, 2H), 5.82 (dt, *J* = 11.2, 6.8 Hz, 1H), 5.66 (dt, *J* = 11.2, 7.2 Hz, 1H), 4.64 (d, *J* = 6.8 Hz, 2H), 4.31 (s, 2H), 3.97 (d, *J* = 7.2 Hz, 2H), 3.91 (s, 3H), 2.33 (s, 3H), 2.00 (s, 3H);

^{13}C NMR (100 MHz, $CDCl_3$): δ 170.5, 166.1, 143.6, 135.3, 131.3, 129.6 ,129.5, 129.2, 129.1, 127.7, 127.6, 126.4, 84.8, 84.4, 59.5, 52.1, 43.2, 36.7, 21.3, 20.6;

IR (KBr): 3463, 2956, 1741, 1601, 1425, 1275, 959, 770, 692, 586 cm^{-1};

HRMS-ESI (m/z) Calcd for ($C_{24}H_{25}NO_6SNa$) ($[M+Na]^+$): 478.1295; found: 478.1303.

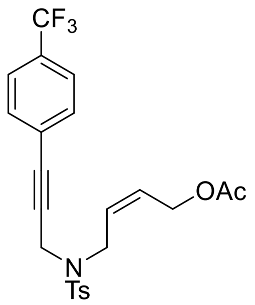

Chemical Formula: $C_{23}H_{22}F_3NO_4S$
Exact Mass: 465.1222

Purification by column chromatography (25% EtOAc in petroleum ether) gave (*Z*)-*N*-(3-(4-trifluoromethylphenyl)prop-2-ynyl)-*N*-(4-acetoxy-2-butenyl)-*p*-methylphenylsulfonamide (**1e**) as a colorless solid (8.61 g, 92% yield).

^{1}H NMR (400 MHz, $CDCl_3$): δ 7.77 (d, *J* = 8.0 Hz, 2H), 7.51 (d, *J* = 8.0 Hz, 2H), 7.27 (d, *J* = 8.4 Hz, 2H), 7.20 (d, *J* = 8.0 Hz, 2H), 5.85 (dt, *J* = 11.2, 6.8 Hz, 1H), 5.67 (dt, *J* = 10.8, 7.2 Hz, 1H), 4.66 (d, *J* = 7.2 Hz, 2H), 4.32 (s, 2H), 3.99 (d, *J* = 7.2 Hz, 2H), 2.33 (s, 3H), 2.01 (s, 3H);

^{13}C NMR (100 MHz, $CDCl_3$): δ 170.6, 143.7, 135.4, 131.7, 130.1 (q, *J* = 32.4 Hz, 1C), 129.5, 129.3, 127.8, 127.7, 125.6, 125.0 (q, *J* = 3.7Hz, 2C), 123.7 (q, *J* = 271Hz, 1C), 84.3, 84.0, 59.6, 43.3, 36.7, 21.3, 20.7;

^{19}F NMR (376 MHz, $CDCl_3$): δ -62.9 (s, 3F);

IR (KBr): 3436, 1727, 1323, 1243, 1161, 1066, 903, 850, 664, 581 cm^{-1};

HRMS-ESI (m/z) Calcd for ($C_{23}H_{22}F_3NO_4SNa$) ($[M+Na]^+$): 488.1114; found: 488.1117.

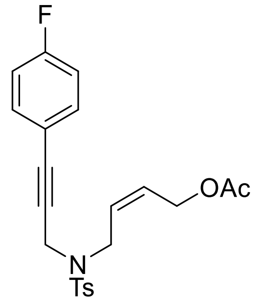

Chemical Formula: $C_{22}H_{22}FNO_4S$
Exact Mass: 415.1254

Purification by column chromatography (25% EtOAc in petroleum ether) gave (*Z*)-*N*-(3-(4-fluorophenyl)prop-2-ynyl)-*N*-(4-acetoxy-2-butenyl)-*p*-methylphenylsulfonamide (**1f**) as a yellow solid (7.54 g, 91% yield).

^{1}H NMR (400 MHz, $CDCl_3$): δ 7.75 (d, *J* = 8.4 Hz, 2H), 7.25 (d, *J* = 8.0 Hz, 2H), 7.10–7.00 (m, 2H), 6.93 (t, *J* = 8.4 Hz, 2H), 5.81 (dt, *J* = 10.8, 6.8 Hz, 1H), 5.64 (dt, *J* = 10.8, 7.6 Hz, 1H), 4.64 (d, *J* = 6.8 Hz, 2H), 4.27 (s, 2H), 3.95 (d, *J* = 7.6 Hz, 2H), 2.33 (s, 3H), 2.00 (s, 3H);

^{13}C NMR (100 MHz, $CDCl_3$): δ 170.6, 162.4 (d, *J* = 248.6 Hz), 143.6, 135.4, 133.3 (d, *J* = 8.3 Hz), 129.5, 129.2, 127.9, 127.7, 117.9 (d, *J* = 3.5 Hz), 115.4 (d, *J* = 22.0 Hz), 84.6, 81.0, 59.6, 43.2, 36.7, 21.3, 20.7; ^{19}F NMR (376 MHz, $CDCl_3$): δ -110.2 (s, 1F);

IR (KBr): 3447, 1735, 1506, 1345, 1248, 1163, 899, 841, 766, 675, 575 cm^{-1};

HRMS-ESI (m/z) Calcd for ($C_{22}H_{22}FNO_4SNa$) ($[M+Na]^+$): 438.1146; found: 438.1145.

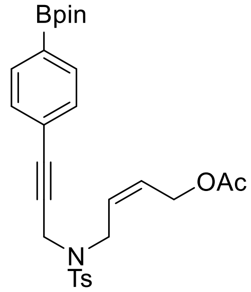

Chemical Formula: $C_{22}H_{22}NO_4S$
Exact Mass: 396.1270

Purification by column chromatography (25% EtOAc in petroleum ether) gave (*Z*)-*N*-(3-(4-pinacolborylphenyl) prop-2-ynyl)-*N*-(4-acetoxy-2-butenyl)-*p*-methylphenyl sulfonamide (**1g**) as a colorless solid (6.64 g, 85% yield, 15 mmol scale).

^{1}H NMR (400 MHz, $CDCl_3$): δ 7.76 (d, *J* = 8.4 Hz, 2H), 7.66 (d, *J* = 8.0 Hz, 2H), 7.25 (d, *J* = 8.0 Hz, 2H), 7.03 (d, *J* = 8.4 Hz, 2H), 5.80 (dt, *J* = 11.2, 6.8 Hz, 1H), 5.65 (dt, *J* = 11.2, 7.2 Hz, 1H), 4.64 (d, *J* = 6.8 Hz, 2H), 4.30 (s, 2H), 3.96 (d, *J* = 7.2 Hz, 2H), 2.33 (s, 3H), 1.98 (s, 3H), 1.33 (s, 12H);

^{13}C NMR (100 MHz, $CDCl_3$): δ 170.6, 143.7, 135.4, 134.3, 130.5, 129.5, 129.2, 127.9, 127.7, 124.5, 85.8, 83.9, 82.6, 59.6, 43.2, 36.8, 24.8, 21.4, 20.7;

IR (KBr): 3451, 2981, 1742, 1608, 1361, 1230, 1162, 1089, 895, 761, 662, 588 cm^{-1};

HRMS-ESI (m/z) Calcd for ($C_{28}H_{34}BNO_6SNa$) ([M+Na]$^+$): 546.2092; found: 546.2099.

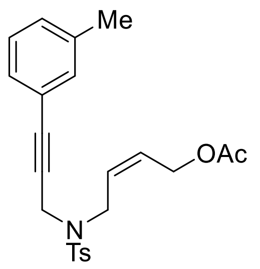

Chemical Formula: $C_{23}H_{25}NO_4S$
Exact Mass: 411.1504

Purification by column chromatography (25% EtOAc in petroleum ether) gave (*Z*)-*N*-(3-(3-methylphenyl)prop-2-ynyl)-*N*-(4-acetoxy-2-butenyl)-*p*-methylphenylsulfonamide (**1h**) as a yellow oil (6.95 g, 84% yield).

^{1}H NMR (400 MHz, $CDCl_3$): δ 7.75 (d, *J* = 8.2 Hz, 2H), 7.25 (d, *J* = 4.7 Hz, 2H), 7.13–7.03 (m, 2H), 6.85 (s, br, 2H), 5.80 (dt, *J* = 10.8, 6.8 Hz 1H), 5.64 (dt, *J* = 11.2, 7.2 Hz 1H), 4.64 (d, *J* = 6.8 Hz, 2H), 4.28 (s, 2H), 3.95 (d, *J* = 7.2 Hz, 2H), 2.32 (s, 3H), 2.27 (s, 3H), 1.97 (s, 3H);

^{13}C NMR (100 MHz, $CDCl_3$): 170.5, 143.5, 137.6, 135.4, 131.9, 129.5 (2C), 129.2, 129.1, 128.4, 127.8 (2C), 127.7 (2C), 121.6, 85.9, 80.8, 59.6, 43.1, 36.8, 21.3, 20.1, 20.6;

IR (neat): 2923, 1739, 1599, 1444, 1348, 1233, 1162, 903, 786, 693, 660, 582 cm^{-1};

HRMS-ESI (m/z) Calcd for ($C_{23}H_{25}NO_4SNa$) ([M+Na]$^+$): 434.1397; found: 434.1406.

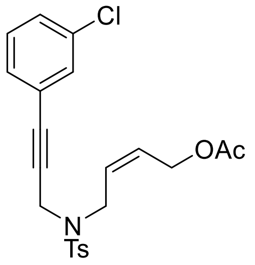

Chemical Formula: $C_{22}H_{22}ClNO_4S$
Exact Mass: 431.0958

Purification by column chromatography (25% EtOAc in petroleum ether) gave (*Z*)-*N*-(3-(3-chlorophenyl)prop-2-ynyl)-*N*-(4-acetoxy-2-butenyl)-*p*-methylphenylsulfonamide (**1i**) as a yellow solid (6.91 g, 80% yield).

^{1}H NMR (400 MHz, $CDCl_3$): δ 7.73 (d, *J* = 8.3 Hz, 2H), 7.27–7.18 (m, 3H), 7.13 (t, *J* = 7.9 Hz, 1H), 6.98–6.87 (m, 2H), 5.80 (dt, *J* = 10.8, 6.8 Hz 1H), 5.64 (dt, *J* = 11.2, 7.2 Hz 1H), 4.64 (d, *J* = 6.8 Hz, 2H), 4.28 (s, 2H), 3.95 (d, *J* = 7.2 Hz, 2H), 2.33 (s, 3H), 1.97 (s, 3H);

^{13}C NMR (100 MHz, $CDCl_3$) δ 170.4, 143.7, 135.3, 133.7, 131.2, 129.5, 129.4, 129.3, 129.2, 128.6, 127.7, 127.6, 123.4, 84.2, 82.5, 59.5, 43.2, 36.6, 21.3, 20.6;

IR (KBr): 3439, 1728, 1342, 1246, 1159, 894, 795, 677, 579 cm^{-1};

HRMS-ESI (m/z) Calcd for ($C_{22}H_{22}ClNO_4SNa$) ([M+Na]$^+$): 454.0850; found: 454.0856.

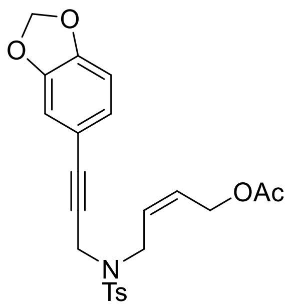

Chemical Formula: $C_{23}H_{23}NO_6S$
Exact Mass: 441.1246

Purification by column chromatography (25% EtOAc in petroleum ether) gave (*Z*)-*N*-(3-(benzo[d][1,3]dioxol-5-yl)prop-2-ynyl)-*N*-(4-acetoxy-2-butenyl)-*p*-methylphenyl sulfonamide (**1j**) as a yellow solid (6.42 g, 73% yield).

^{1}H NMR (400 MHz, $CDCl_3$): δ 7.70 (d, *J* = 8.2 Hz, 2H), 7.23 (d, *J* = 8.0 Hz, 2H), 6.58 (m, 2H), 6.40 (s, 1H), 5.88 (s, 2H), 5.76 (dt, *J* = 11.2, 6.8 Hz, 1H), 5.58 (dt, *J* = 10.8, 7.2 Hz, 1H), 4.60 (d, *J* = 6.8 Hz, 2H), 4.21 (s, 2H), 3.91 (d, *J* = 7.2 Hz, 2H), 2.31 (s, 3H), 1.95 (s, 3H);

^{13}C NMR (100 MHz, $CDCl_3$): δ 170.5, 147.9, 147.1, 143.5, 135.5, 129.5, 129.1, 127.9, 127.6, 126.0, 114.9, 111.3, 108.1, 101.2, 85.5, 79.5, 59.6, 43.1, 36.7, 21.2, 20.7;

IR (KBr): 3435, 1725, 1492, 1340, 1249, 1117, 1037, 905, 808, 722, 662, 572 cm^{-1};

HRMS-ESI (m/z) Calcd for ($C_{23}H_{23}NO_6SNa$) ([M+Na]$^+$): 464.1138; found: 464.1147.

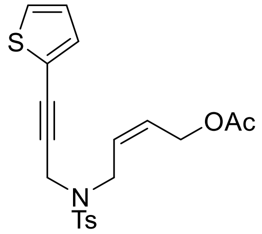

Chemical Formula: $C_{20}H_{21}NO_4S_2$
Exact Mass: 403.0912

Purification by column chromatography (25% EtOAc in petroleum ether) gave (*Z*)-*N*-(3-(thiophen-2-yl)prop-2-ynyl)-N-(4-acetoxy-2-butenyl)-*p*-methylphenylsulfonamide (**1k**) as a yellow oil (6.98 g, 86% yield).

^{1}H NMR (400 MHz, $CDCl_3$): δ 7.71 (d, *J* = 8.2 Hz, 2H), 7.32-7.20 (m, 2H), 7.16 (d, *J* = 4.4 Hz, 1H), 6.91 (d, *J* = 3.4 Hz, 1H), 6.88–6.82 (m, 1H), 5.78 (dt, *J* = 10.8, 6.8 Hz, 1H), 5.64 (dt, *J* = 10.8, 7.2 Hz, 1H), 4.61 (d, *J* = 6.8 Hz, 2H), 4.27 (s, 2H), 3.90 (d, *J* = 7.2 Hz, 2H), 2.32 (s, 3H), 1.98 (s, 3H);

^{13}C NMR (100 MHz, $CDCl_3$): δ 170.6, 143.7, 135.2, 132.3, 129.6, 129.2, 127.8, 127.6, 127.3, 126.7, 121.6, 85.2, 79.0, 59.6, 43.2, 36.9, 21.4, 20.7;

IR (neat): 3443, 1731, 1345, 1252, 1161, 1050, 886, 733, 670, 582 cm^{-1};

HRMS-ESI (m/z) Calcd for ($C_{20}H_{21}NO_4S_2Na$) ([M+Na]$^+$): 426.0804; found: 426.0810.

3.3 Procedures for the synthesis of 1,6-enyne 1l

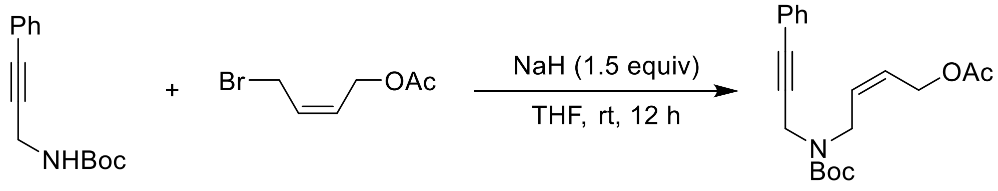

(*Z*)-S2 **1l**

To a suspension of sodium hydride (60% dispersion in mineral oil, 1.8 g, 45 mmol) in THF (100 mL) was added dropwise a solution of *N*-*tert*-butyloxycarbonyl-3-phenylprop-2-yn-1-amine (6.94 g, 30 mmol) in THF (50 mL). The resulting mixture was stirred at room temperature for 30 min before (*Z*)-**S2** (6.95 g, 36 mmol) was added. The reaction mixture was stirred at room temperature for overnight, and then was quenched with a saturated aqueous solution of NH_4Cl (50 mL). The organic layer was separated and the aqueous layer was extracted with EtOAc (100 mL × 3). The combined organic layers were dried over Na_2SO_4, filtered, concentrated and purified by column chromatography to give (*Z*)-4-((*tert*-butoxycarbonyl)(3-phenylprop-2-yn-1-yl)amino)but-2-en-1-yl acetate (**1l**) as a yellow oil (6.66 g, 65% yield).

^{1}H NMR (500 MHz, $CDCl_3$, 50 °C): δ 7.44–7.34 (m, 2H), 7.32–7.22 (m, 2H), 5.75–5.65 (m, 2H), 4.70 (d, J = 5.0 Hz, 2H), 4.24 (s, br, 2H), 4.10 (d, J = 5.0 Hz, 2H), 2.01 (s, 3H), 1.48 (s, 9H);

^{13}C NMR (125 MHz, $CDCl_3$, 50 °C): δ 170.5, 154.8, 131.6, 130.2, 130.0, 128.2, 128.1, 122.8, 84.7, 83.5, 80.4, 59.8, 43.0, 36.5, 28.3, 20.6;

IR (neat): 3371, 2978, 1738, 1692, 1408, 1236, 1165, 1029, 872, 759, 692 cm^{-1};

HRMS-ESI (m/z) Calcd for ($C_{20}H_{25}NO_4Na$) ($[M+Na]^+$): 366.1676, found: 366.1675.

3.4 Procedures for the synthesis of 1,6-enyne 1m

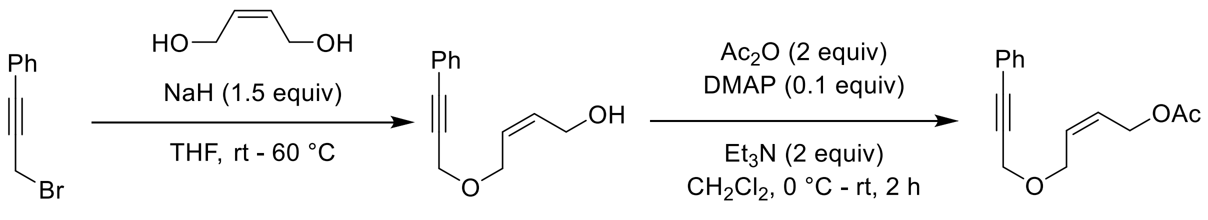

S4 **1m**

Step I: To a suspension of sodium hydride (7.08 g, 60% dispersion in mineral oil, 177 mmol) in THF (150 mL) was added dropwise a solution of (*Z*)-but-2-ene-1,4-diol (20.8 g, 236 mmol) in THF (30 mL). The resulting mixture was stirred at room temperature for 30 min before 3-phenylpropargyl bromide (23.05 g, 118 mmol) was added. The resulting reaction mixture was stirred at 60 °C for 6 hours and quenched with a saturated aqueous solution of Na_2CO_3. The organic layer was separated, and the aqueous layer was extracted with EtOAc. The combined extracts were dried over Na_2SO_4, filtered and concentrated. Purification by column chromatography (25% EtOAc in petroleum ether) gave (*Z*)-4-((3-phenylprop-2-yn-1-yl)oxy)but-2-en-1-ol (**S4**) as a yellow oil (18.50 g, 77% yield).

Step II: To a solution of **S4** (4.02 g, 20 mmol) in CH_2Cl_2 (40 mL) was added Et_3N (8.4 mL, 60 mmol) and DMAP (243.9 mg, 2 mmol) at 0 °C. Then acetic anhydride (5.6 mL, 60 mmol) was added dropwise at the same temperature. The reaction mixture was stirred at room temperature for addition 2 hours. The solvent was removed under reduced pressure. Purification by column chromatography (10% EtOAc in petroleum ether) gave (*Z*)-4-((3-phenylprop-2-yn-1-yl)oxy)but-2-en-1-yl acetate (**1m**) as a yellow oil (4.64 g, 95% yield).

^{1}H NMR (400 MHz, $CDCl_3$): δ 7.48–7.46 (m, 2H), 7.33–7.31 (m, 3H), 5.86–5.73 (m, 2H), 4.70 (d, J = 6.4 Hz, 2H), 4.39 (s, 2H), 4.26 (d, J = 6.0 Hz, 2H), 2.06 (s, 3H);

^{13}C NMR (100 MHz, $CDCl_3$): δ170.7, 131.6, 130.0, 128.4, 128.2, 127.3, 122.4, 86.4, 84.6, 64.9, 60.1, 58.0, 20.8;

IR (neat): 2938, 1739, 1490, 1442, 1372, 1233, 1081, 1031, 758, 692 cm^{-1};

HRMS (m/z) Calcd for ($C_{15}H_{16}O_3Na$) ($[M+Na]^+$): 267.0992; found: 267.0990.

3.5 Procedure for the synthesis of Substrate 1n

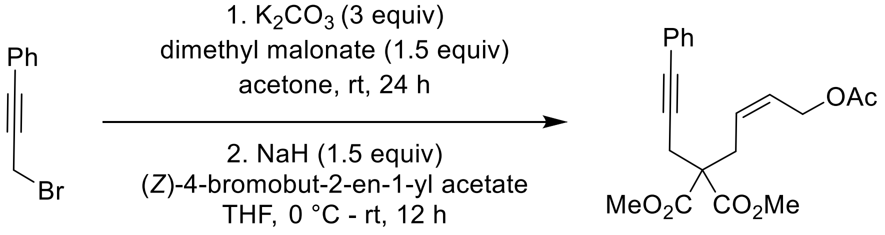

To a 250-mL round-bottomed flask was added K_2CO_3 (12.44 g, 90 mmol), (3-bromoprop-1-yn-1-yl)benzene (5.87 g, 30 mmol), dimethyl malonate (5.97 g, 45 mmol) and acetone (100 mL). The mixture was stirred at room temperature for 24 h. Then the reaction mixture was quenched with a saturated aqueous solution of NH_4Cl and extracted with EtOAc. The combined organic layers were dried over Na_2SO_4, filtered and concentrated. The residue was dissolved in THF (30 mL), and the resulting solution was added to a suspension of sodium hydride (60% dispersion in mineral oil, 1.81 g, 45 mmol) in THF (60 mL) dropwise. The mixture was stirred at 0 °C for 30 min before (*Z*)-4-bromobut-2-en-1-yl acetate (5.79 g, 30 mmol) was added. The reaction mixture was allowed to stir at room temperature for 12 h, and then was quenched with a saturated aqueous solution of NH_4Cl. The organic layer was separated and the aqueous layer was extracted with EtOAc. The combined organic layers were dried over Na_2SO_4, filtered and concentrated. Purification by column chromatography (10% EtOAc in petroleum ether) gave dimethyl (*Z*)-2-(4-acetoxybut-2-en-1-yl)-2-(3-phenylprop-2-yn-1-yl)malonate (**1n**) as a colorless oil (2.73 g, 25% yield).

^{1}H NMR (400 MHz, $CDCl_3$): δ 7.40–7.37 (m, 2H), 7.30–7.28 (m, 3H), 5.78–5.72 (m, 1H), 5.56–5.49 (m, 1H), 4.70 (d, J = 7.2 Hz, 2H), 3.78 (s, 6H), 3.03 (s, 2H), 2.96 (d, J = 8.0 Hz, 2H), 2.04 (s, 3H);

^{13}C NMR (100 MHz, $CDCl_3$): δ 170.6, 170.0, 131.5, 128.3, 128.1, 128.0, 127.3, 122.8, 83.7, 83.6, 60.0, 56.9, 52.8, 30.3, 29.6, 23.6, 20.7;

IR (neat): 2955, 1738, 1491, 1438, 1373, 1231, 1028, 759, 693 cm^{-1};

HRMS (m/z) Calcd for ($C_{20}H_{22}O_6Na$) ($[M+Na]^+$):381.1309; found: 381.1306.

3.6 Procedures for the synthesis of 1,6-enynes (*R,E*)-4a–4i

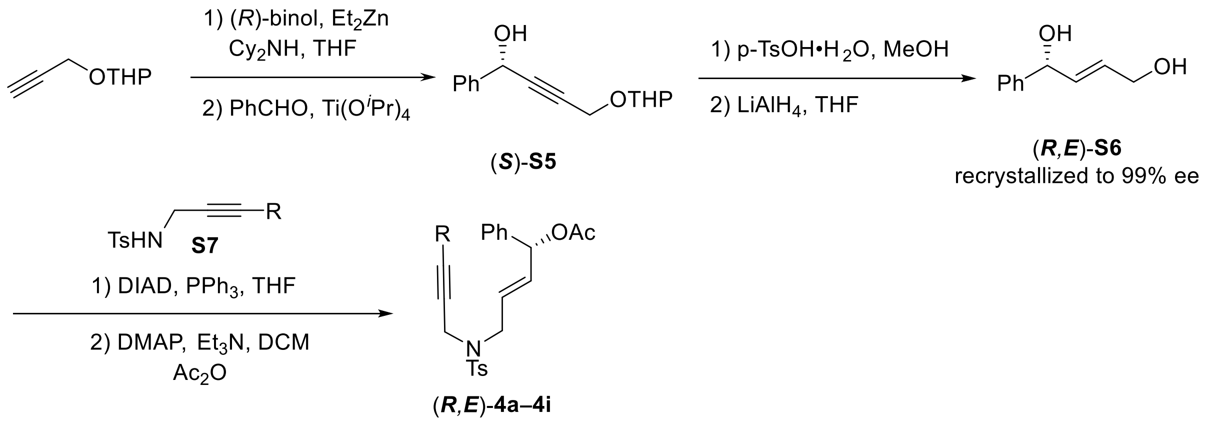

1,6-Enynes **(*R,E*)-4a–4i** were synthesized according to the literature procedure.[3]

Step I: To an oven-dried round-bottomed flask was added (*R*)-binol (2.3 g, 8 mmol), Cy_2NH (200 μl, 1 mmol), and THF (40 mL). The mixture was cooled to 0 °C before $ZnEt_2$ (60 mL, 180 mmol) was added. The resulting mixture was then stirred at room temperature for 16 hours. Then 2-(prop-2-yn-1-yloxy)tetrahydro-2*H*-pyran (8.5 mL, 60 mmol) was added, and the mixture was stirred at room temperature for additional 8 hours. The mixture was cooled to 0 °C before $Ti(O^{i}Pr)_4$ (6 ml, 20 mmol) and benzaldehyde (2 mL, 20 mmol) were added. The mixture was stirred at room temperature for another 16 hours. The reaction was quenched with a saturated aqueous solution of NH_4Cl and extracted with EtOAc. The combined organic layers were dried over Na_2SO_4, filtered and concentrated. Purification by column chromatography (PE/EA = 5/1) gave (1*S*)-1-phenyl-4-((tetrahydro-2H-pyran-2-yl)oxy)but-2-yn-1-ol (*S*)-**S5** as a colorless oil (4.3 g, 88% yield).

Step II: To a solution of (*S*)-**S5** (4.3 g, 17.5mmol) in MeOH (30 mL) was added PTSA•H_2O (166.4 mg,0.9 mmol). After stirring at room temperature for 1 hour, the mixture was quenched with a saturated aqueous solution of $NaHCO_3$ and extracted with EtOAc. The combined organic layers were dried over Na_2SO_4, filtered and concentrated. The residue was dissolved in anhydrous THF (40 mL). Then $LiAlH_4$ (0.88g, 23.1 mmol) was added in portions at 0 °C under N_2. After stirring at 0 °C for 3 hours, the reaction mixture was quenched by the addition of Na_2SO_4•$10H_2O$ and stirred for another 1 hour. The reaction mixture was filtered through a celite pad, and the filtrate was concentrated and purified by chromatography on silica gel, eluting with PE/EA = 2/1 to afford the (*R*,*E*)-**S6** as a white solid (594.9 mg, 70% yield), which

was recrystallized in THF/PE and the final ee value was determined to be 99%.

Step III: To a solution of (*R*,*E*)-**S6** (2.6 mmol, 426.9 mg), propargyl amines **S7** (2.0 mmol) and PPh_3 (2.6 mmol, 681.2 mg) in THF (30 mL) was added DIAD (2.6 mmol, 525.2 mg) dropwise at 0 °C under N_2. The reaction mixture was stirred at room temperature overnight. The mixture was concentrated under vacuum and purified by flash column chromatography (PE/EA = 10/1) to give the alkyne-tethered allylic alcohol. To a solution of the allylic alcohol in CH_2Cl_2 (5 mL) was added Et_3N (0.7 mL, 6 mmol) and DMAP (25.0 mg, 0.2 mmol) at 0 °C. Then acetic anhydride (0.5 mL, 6 mmol) was added dropwise. The reaction mixture was stirred at room temperature for 2 hours. The solvent was removed under vacuum. Purification by column chromatography gave the corresponding 1,6-enynes (*R,E*)-**4a**–**4i**.

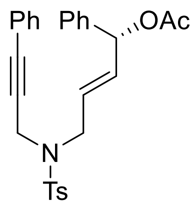

Chemical Formula: $C_{28}H_{27}NO_4S$
Exact Mass: 473.1661

Purification by column chromatography (PE/EA = 10/1) gave (*R*,*E*)-4-((4-methyl-*N*-(3-phenylprop-2-yn-1-yl)phenyl)sulfonamido)-1-phenylbut-2-en-1-yl acetate (*R*,*E*)-**4a** as a colorless oil (563.0 mg, 60% yield, 99% ee).

^{1}H NMR (600 MHz, $CDCl_3$): δ 7.77–7.72 (m, 2H), 7.37–7.29 (m, 5H), 7.28–7.26 (m, 1H), 7.25–7.20 (m, 4H), 7.05–7.00 (m, 2H), 6.26 (dd, *J* = 5.8, 1.3 Hz, 1H), 5.97–5.90 (m, 1H), 5.75–5.67 (m, 1H), 4.27 (s, 2H), 3.91 (d, *J* = 6.5 Hz, 2H), 2.33 (s, 3H), 2.08 (s, 3H);

^{13}C NMR (151 MHz, $CDCl_3$): δ 169.9, 143.5, 138.6, 135.8, 133.7, 131.4, 129.5, 128.6, 128.4, 128.3, 128.1, 127.8, 127.1, 126.3, 122.1, 85.7, 81.5, 75.0, 48.0, 36.9, 21.4, 21.2;

HRMS: (ESI) calcd for $C_{28}H_{28}NO_4S^+$ ($[M+H]^+$): 474.1734; found: 474.1735;

HPLC conditions: AD-H column (15% ^{i}PrOH in hexane, 1.0 mL/min, λ = 254 nm, 30 °C), t_R (major) = 11.6 min, t_R (minor) = 14.3 min;

Optical Rotation: $[\alpha]^{25}_D$ = -20.3 (*c* = 0.05, $CHCl_3$) for 99% ee.

With the use of (*S*)-binol, (*S*,*E*)-**4a** was also prepared in 99% ee.

HPLC conditions: AD-H column (15% ^{i}PrOH in hexane, 1.0 mL/min, λ = 254 nm, 30 °C), t_R (minor) = 11.0 min, t_R (major) = 14.5 min;

Optical Rotation: $[\alpha]^{25}_D$ = 28.9 (*c* = 0.07, $CHCl_3$) for 99% ee.

<Chromatogram>

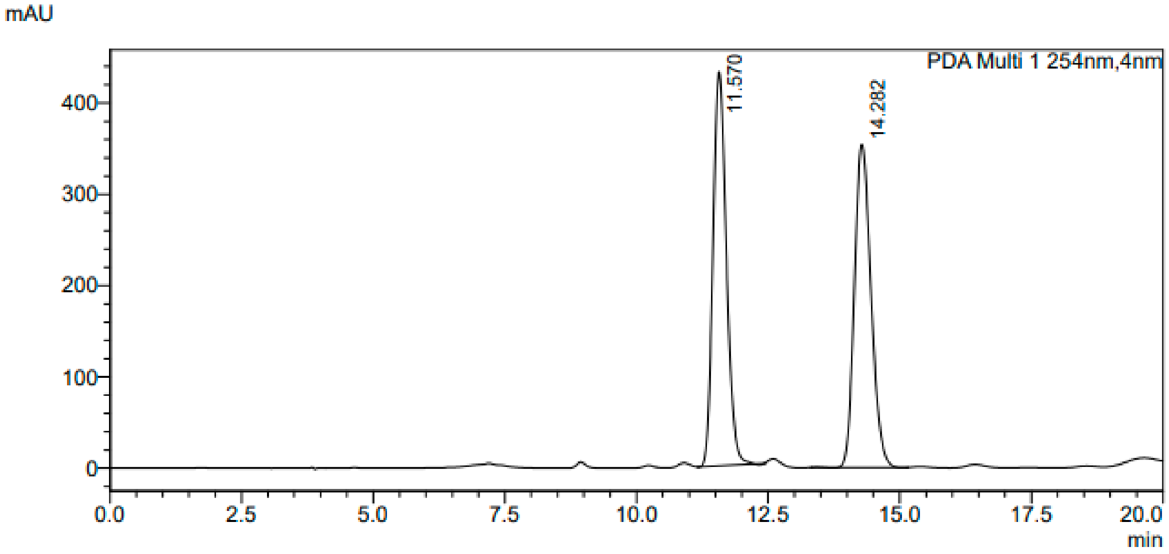

<Peak Table>

PDA Ch1 254nm

Peak#	Ret. Time	Area	Height	Conc.	Unit	Mark	Name
1	11.570	7630879	431371	0.000		M	
2	14.282	7752261	354013	0.000		M	
Total		15383140	785384				

Supplementary Figure 1. HPLC spectrum of racemic-(*E*)-**4a**

<Chromatogram>

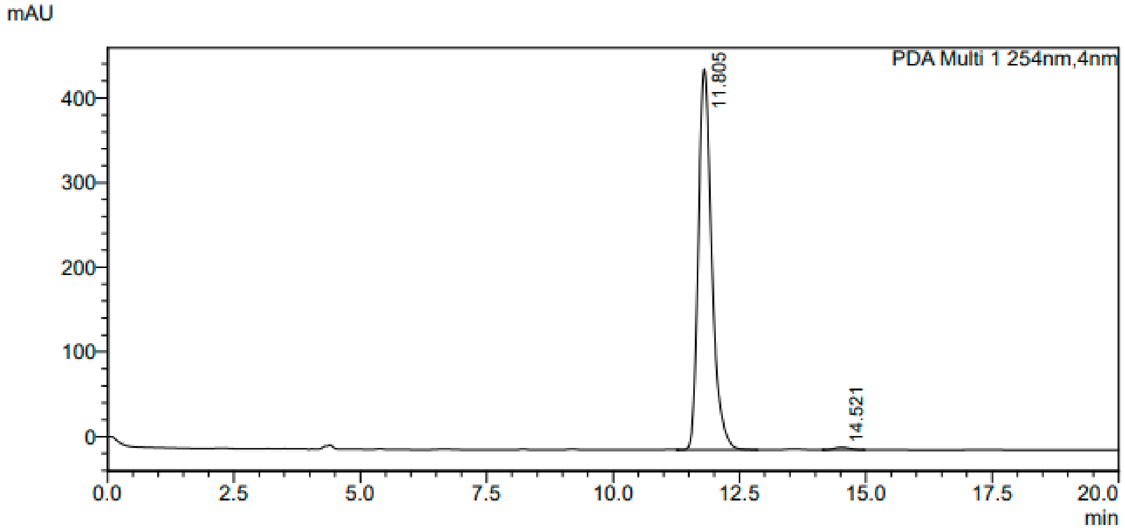

<Peak Table>

PDA Ch1 254nm

Peak#	Ret. Time	Area	Height	Conc.	Unit	Mark	Name
1	11.805	8292311	449584	0.000		M	
2	14.521	57186	2806	0.000		M	
Total		8349497	452390				

Supplementary Figure 2. HPLC spectrum of (*R*,*E*)-**4a**

<Chromatogram>

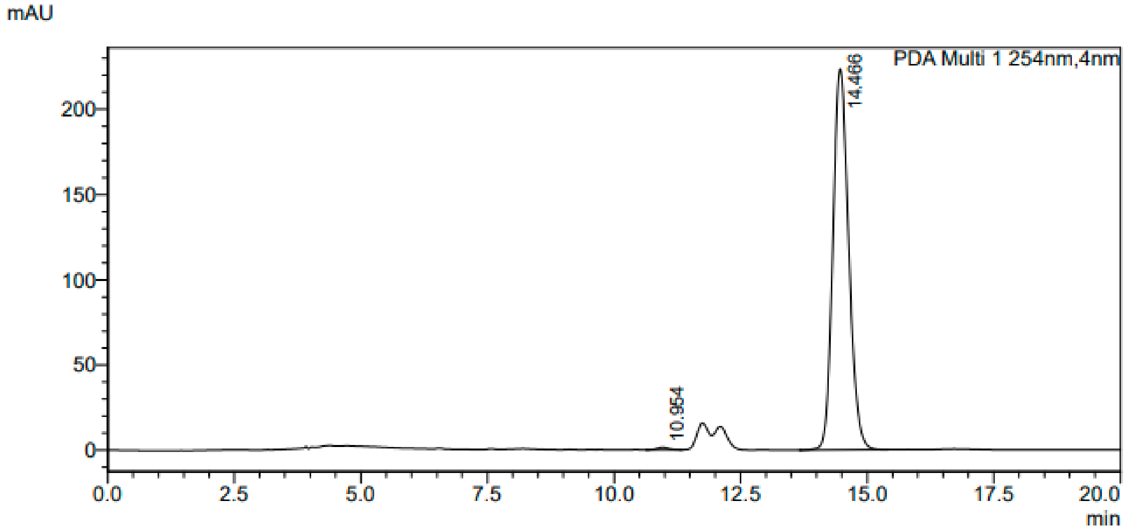

<Peak Table>

PDA Ch1 254nm

Peak#	Ret. Time	Area	Height	Conc.	Unit	Mark	Name
1	10.954	23053	1456	0.000		M	
2	14.466	4855014	223428	0.000		M	
Total		4878067	224884				

Supplementary Figure 3. HPLC spectrum of (*S*,*E*)-**4a**

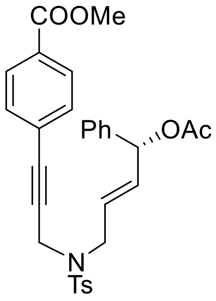

Chemical Formula: $C_{30}H_{29}NO_6S$
Exact Mass: 531.1716

Purification by column chromatography (PE/EA = 5/1) gave methyl (*R*,*E*)-4-(3-((*N*-(4-acetoxy-4-phenylbut-2-en-1-yl)-4-methylphenyl)sulfonamido)prop-1-yn-1-yl)benzoate (*R*,*E*)-**4b** as a colorless oil (425.0 mg, 40% yield, 99% ee).

^{1}H NMR (600 MHz, $CDCl_3$): δ 7.91–7.88 (m, 2H), 7.76–7.72 (m, 2H), 7.36–7.32 (m, 2H), 7.32–7.28 (m, 3H), 7.24 (d, J = 8.1 Hz, 2H), 7.06 (d, J = 8.2 Hz, 2H), 6.25 (d, J = 5.8 Hz, 1H), 5.95–5.90 (m, 1H), 5.73–5.67 (m, 1H), 4.28 (s, 2H), 3.92 (d, J = 0.7 Hz, 3H), 3.90 (d, J = 6.7 Hz, 2H), 2.33 (s, 3H), 2.08 (d, J = 0.6 Hz, 3H);

^{13}C NMR (151 MHz, $CDCl_3$): δ 169.9, 166.3, 143.7, 138.5, 135.8, 133.9, 131.4, 129.7, 129.6, 129.3, 128.7, 128.4, 127.8, 127.1, 126.7, 126.2, 85.0, 84.7, 75.0, 52.3, 48.2, 36.9, 21.5, 21.2;

HRMS: (ESI) calcd for $C_{30}H_{30}NO_6S^+$ ($[M+H]^+$): 532.1788; found: 532.1784;

HPLC conditions: AD-H column (15% ^{i}PrOH in hexane, 1.0 mL/min, λ = 254 nm, 30 °C), t_R (major) = 18.0 min, t_R (minor) = 21.5 min;

Optical Rotation: $[\alpha]^{25}_D$ = -17.2 (c = 0.17, $CHCl_3$) for 99% ee.

<Chromatogram>

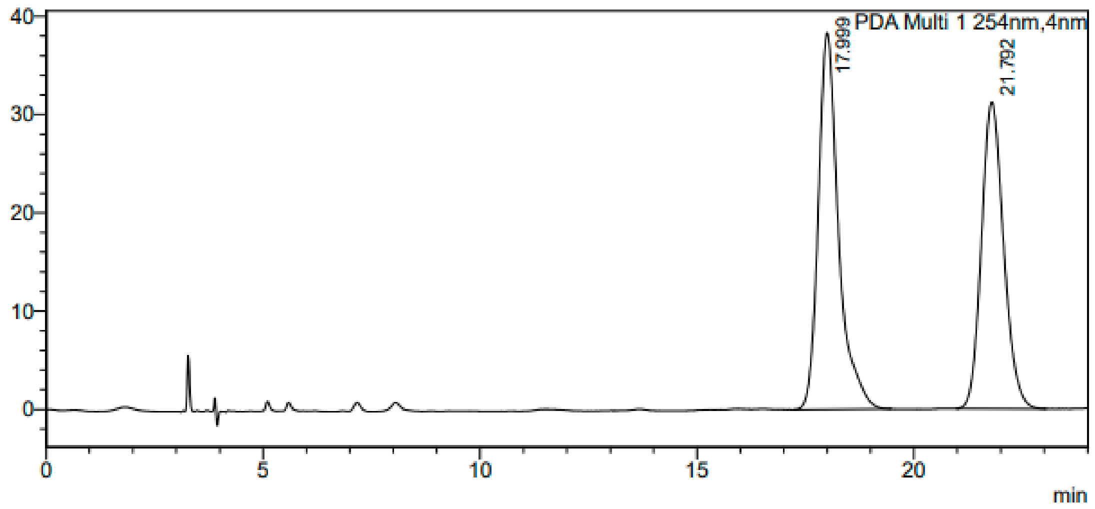

<Peak Table>

PDA Ch1 254nm

Peak#	Ret. Time	Area	Height	Conc.	Unit	Mark	Name
1	17.999	1249026	38324	0.000			
2	21.792	1111758	31217	0.000			
Total		2360784	69541				

Supplementary Figure 4. HPLC spectrum of racemic-(*E*)-**4b**

<Chromatogram>

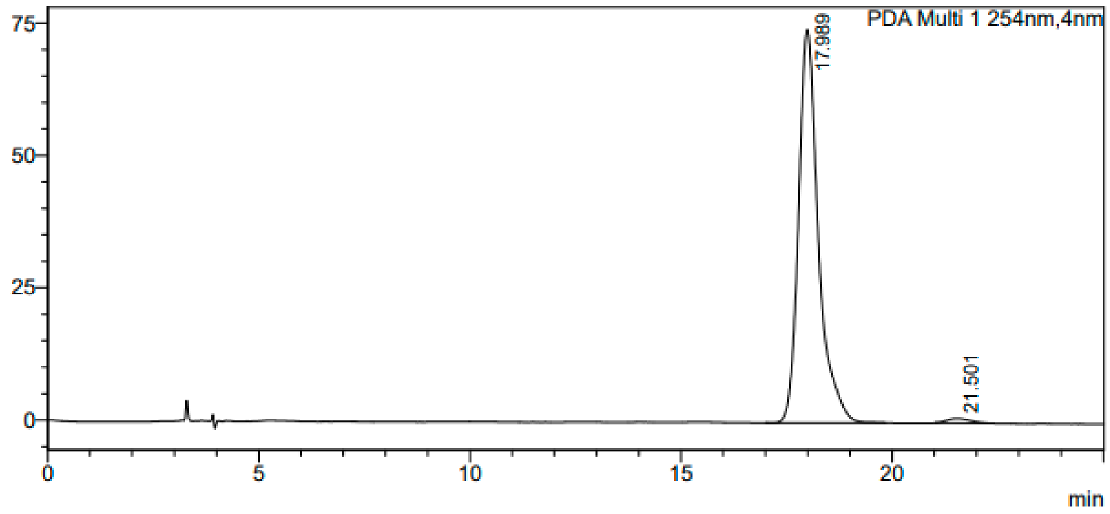

<Peak Table>

PDA Ch1 254nm

Peak#	Ret. Time	Area	Height	Conc.	Unit	Mark	Name
1	17.989	2429439	74310	0.000		M	
2	21.501	33025	873	0.000		M	
Total		2462464	75183				

Supplementary Figure 5. HPLC spectrum of (*R*,*E*)-**4b**

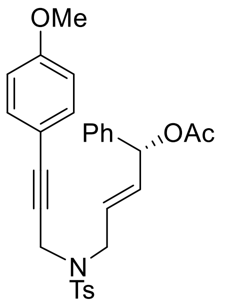

Purification by column chromatography (PE/EA = 5/1) gave (*R*,*E*)-4-((*N*-(3-(4-methoxyphenyl)prop-2-yn-1-yl)-4-methylphenyl)sulfonamido)-1-phenylbut-2-en-1-yl acetate (*R*,*E*)-**4c** as a colorless oil (392.5 mg, 39% yield, 99% ee).

^{1}H NMR (600 MHz, $CDCl_3$): δ 7.77–7.73 (m, 2H), 7.36–7.33 (m, 2H), 7.32–7.29 (m, 3H), 7.25–7.22 (m, 2H), 6.99–6.95 (m, 2H), 6.77–6.73 (m, 2H), 6.26 (dd, *J* = 5.8, 1.3 Hz, 1H), 5.97–5.91 (m, 1H), 5.73–5.66 (m, 1H), 4.25 (s, 2H), 3.92–3.88 (m, 2H), 3.78 (s, 3H), 2.35 (s, 3H), 2.08 (s, 3H);

^{13}C NMR (151 MHz, $CDCl_3$): δ 169.8, 159.6, 143.4, 138.6, 135.8, 133.5, 132.9, 129.4, 128.6, 128.2, 127.7, 127.0, 126.4, 114.1, 113.7, 85.6, 80.0, 75.0, 55.2, 47.9, 37.0, 21.4, 21.1;

HRMS: (ESI) calcd for $C_{29}H_{30}NO_4S^+$ ([M+H]$^+$): 504.1839; found: 504.1833;

HPLC conditions: AD-H column (20% ^{i}PrOH in hexane, 1.0 mL/min, λ = 214 nm, 30 °C), t_R (major) = 11.9 min, t_R (minor) = 14.5 min;

Optical Rotation: $[\alpha]^{25}_D$ = -58.7 (*c* = 0.17, $CHCl_3$) for 99% ee.

<Chromatogram>

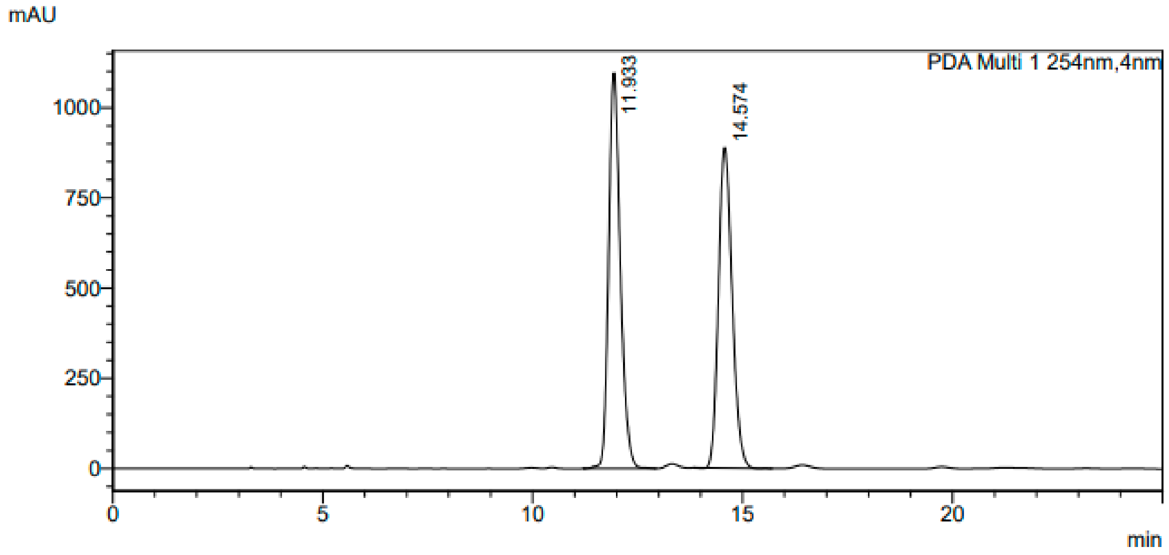

<Peak Table>

PDA Ch1 254nm

Peak#	Ret. Time	Area	Height	Conc.	Unit	Mark	Name
1	11.933	21205307	1096274	0.000		M	
2	14.574	20141791	888790	0.000		M	
Total		41347098	1985064				

Supplementary Figure 6. HPLC spectrum of racemic-(*E*)-**4c**

<Chromatogram>

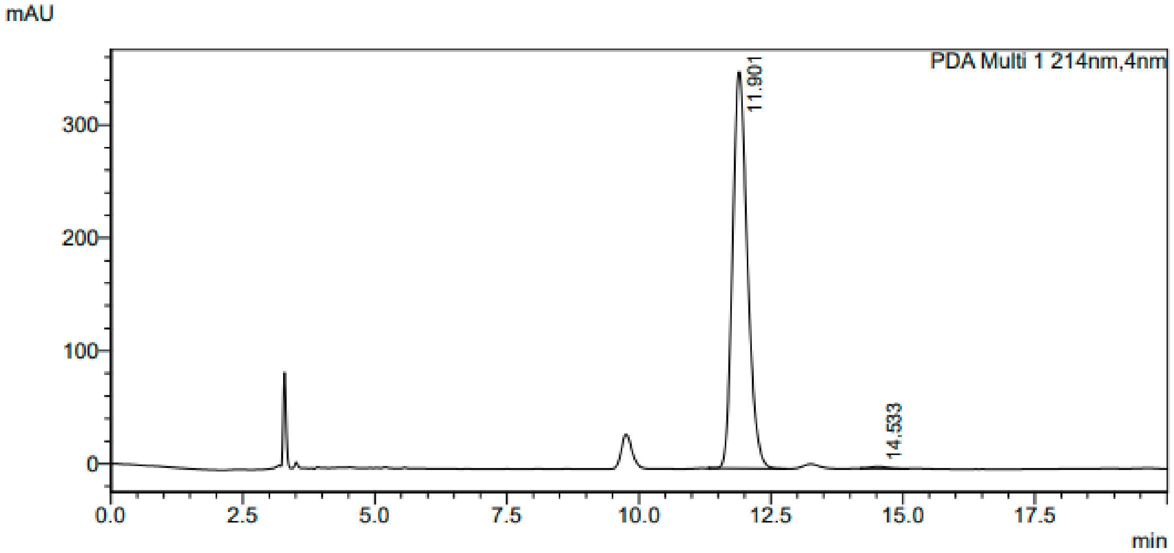

<Peak Table>

PDA Ch1 214nm

Peak#	Ret. Time	Area	Height	Conc.	Unit	Mark	Name
1	11.901	6851275	351274	99.507		M	
2	14.533	33922	1649	0.493		M	
Total		6885198	352923				

Supplementary Figure 7. HPLC spectrum of (*R*,*E*)-**4c**

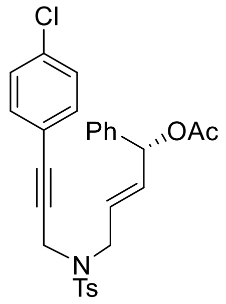

Chemical Formula: $C_{28}H_{26}ClNO_4S$
Exact Mass: 507.1271

Purification by column chromatography (PE/EA = 5/1) gave (*R*,*E*)-4-((*N*-(3-(4-chlorophenyl)prop-2-yn-1-yl)-4-methylphenyl)sulfonamido)-1-phenylbut-2-en-1-yl acetate (*R*,*E*)-**4d** as a colorless oil (486.8 mg, 48% yield, 99% ee).
^{1}H NMR (600 MHz, $CDCl_3$): δ 7.76–7.72 (m, 2H), 7.36–7.28 (m, 5H), 7.25–7.18 (m, 4H), 6.96–6.92 (m, 2H), 6.26 (dd, *J* = 5.8, 1.3 Hz, 1H), 5.96–5.90 (m, 1H), 5.73–5.67 (m, 1H), 4.25 (s, 2H), 3.92–3.88 (m, 2H), 2.34 (s, 3H), 2.08 (s, 3H);

^{13}C NMR (151 MHz, $CDCl_3$): δ 169.8, 143.5, 138.5, 135.7, 134.4, 133.7, 132.6, 129.4, 128.6, 128.4, 128.2, 127.7, 127.0, 126.2, 120.5, 84.5, 82.6, 74.9, 48.0, 36.8, 21.3, 21.1;

HRMS: (ESI) calcd for $C_{28}H_{27}ClNO_4S^+$ ($[M+H]^+$): 508.1344; found: 508.1343;

HPLC conditions: AD-H column (15% ^{i}PrOH in hexane, 1.0 mL/min, λ = 254 nm, 30 °C), t_R (major) = 13.8 min, t_R (minor) = 16.7 min;

Optical Rotation: $[\alpha]^{25}_D$ = -3.4 (*c* = 0.07, $CHCl_3$) for 99% ee.

<Chromatogram>

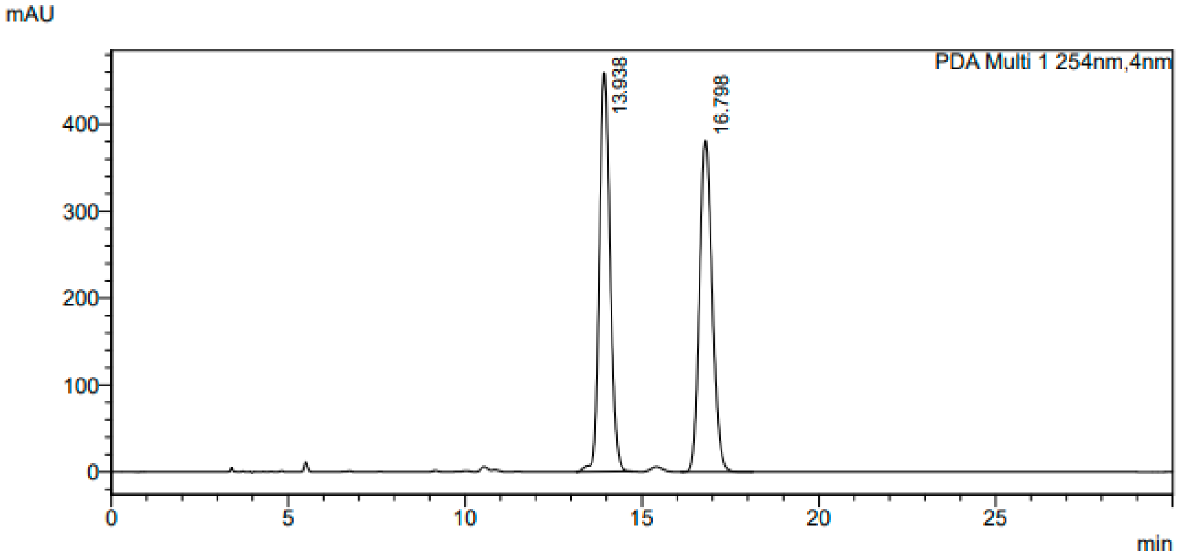

<Peak Table>

PDA Ch1 254nm

Peak#	Ret. Time	Area	Height	Conc.	Unit	Mark	Name
1	13.938	9686044	458830	0.000		M	
2	16.798	9599979	381178	0.000		M	
Total		19286022	840008				

Supplementary Figure 8. HPLC spectrum of racemic-(*E*)-**4d**

<Chromatogram>

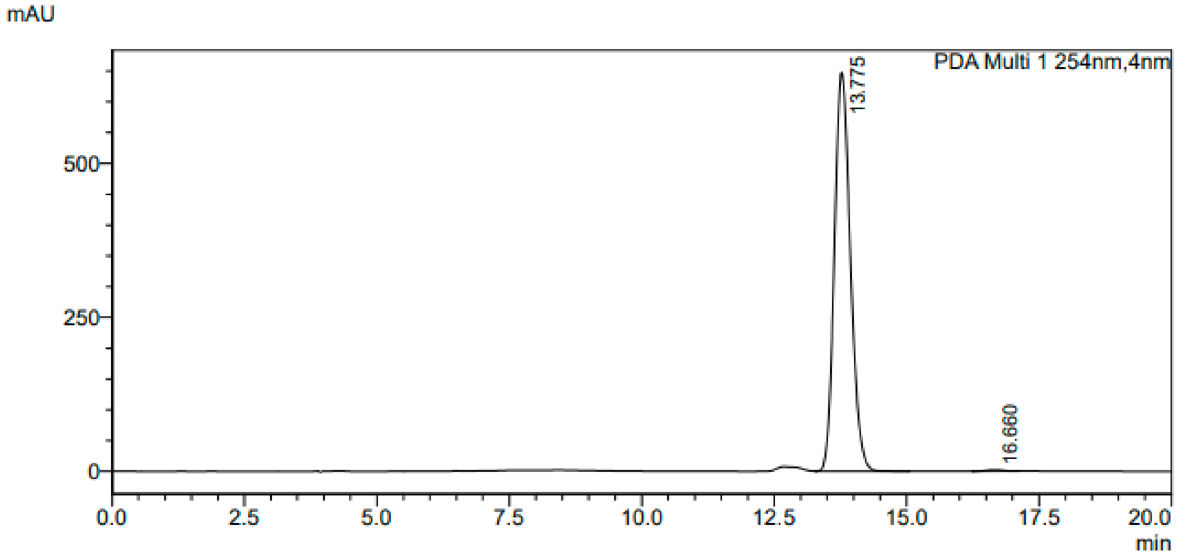

<Peak Table>

PDA Ch1 254nm

Peak#	Ret. Time	Area	Height	Conc.	Unit	Mark	Name
1	13.775	13586998	647236	0.000		M	
2	16.660	51710	2396	0.000		M	
Total		13638708	649632				

Supplementary Figure 9. HPLC spectrum of (*R*,*E*)-**4d**

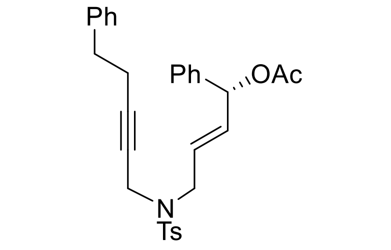

Purification by column chromatography (PE/EA = 5/1) gave (*R*,*E*)-4-((4-methyl-*N*-(5-phenylpent-2-yn-1-yl)phenyl)sulfonamido)-1-phenylbut-2-en-1-yl acetate (*R*,*E*)-**4e** as a colorless oil (551.3 mg, 55% yield, 99% ee).

^{1}H NMR (600 MHz, $CDCl_3$): δ 7.70–7.68 (m, 2H), 7.37–7.33 (m, 2H), 7.32–7.28 (m, 3H), 7.26 (s, 1H), 7.25–7.21 (m, 3H), 7.18–7.14 (m, 1H), 7.05–7.01 (m, 2H), 6.21 (d, *J* = 5.7 Hz, 1H), 5.74–5.69 (m, 1H), 5.62–5.56 (m, 1H), 3.99 (t, *J* = 2.2 Hz, 2H), 3.71 (d, *J* = 6.5 Hz, 2H), 2.52 (t, *J* = 7.5 Hz, 2H), 2.40 (s, 3H), 2.21–2.16 (m, 2H), 2.09 (s, 3H);

^{13}C NMR (151 MHz, $CDCl_3$): δ 169.8, 143.3, 140.2, 138.6, 136.1, 133.4, 129.3, 128.6, 128.4, 128.3, 128.2, 127.8, 127.1, 126.3, 85.4, 75.0, 73.0, 47.5, 36.4, 34.5, 21.5, 21.2, 20.3;

HRMS: (ESI) calcd for $C_{30}H_{32}NO_4S^+$ ([M+H]$^+$): 502.2047; found: 502.2050;

HPLC conditions: IA-H column (15% ^{i}PrOH in hexane, 1.0 mL/min, λ = 190 nm, 30 °C), t_R (major) = 10.1 min, t_R (minor) = 10.9 min;

Optical Rotation: $[\alpha]^{25}_{D}$ = -57.0 (*c* = 0.17, $CHCl_3$) for 99% ee.

<Chromatogram>

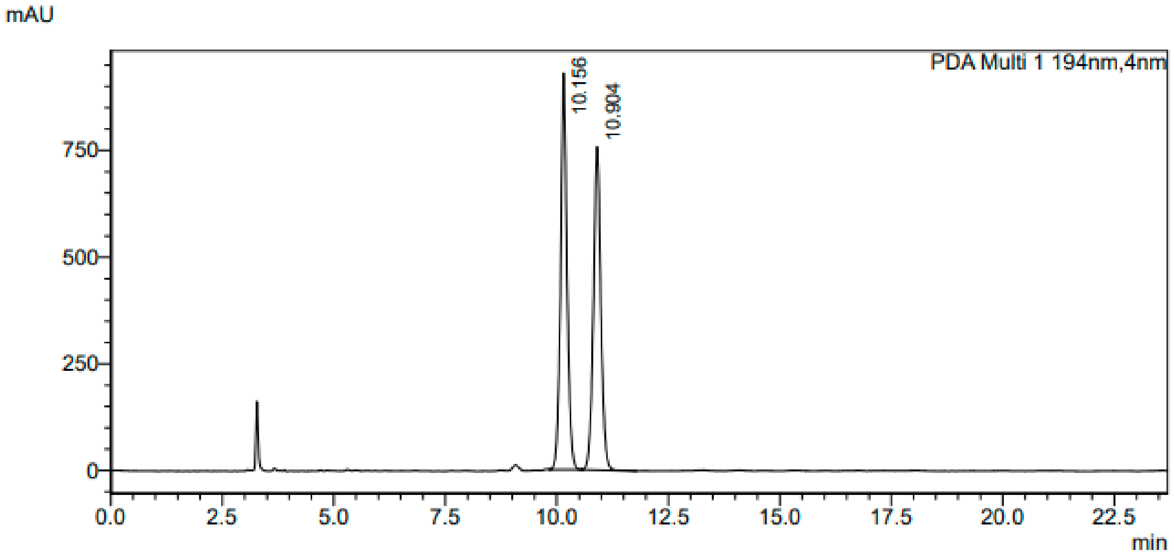

<Peak Table>

PDA Ch1 194nm

Peak#	Ret. Time	Area	Height	Conc.	Unit	Mark	Name
1	10.156	9142172	929075	0.000		M	
2	10.904	8725404	757548	0.000		M	
Total		17867576	1686622				

Supplementary Figure 10. HPLC spectrum of racemic-(*E*)-**4e**

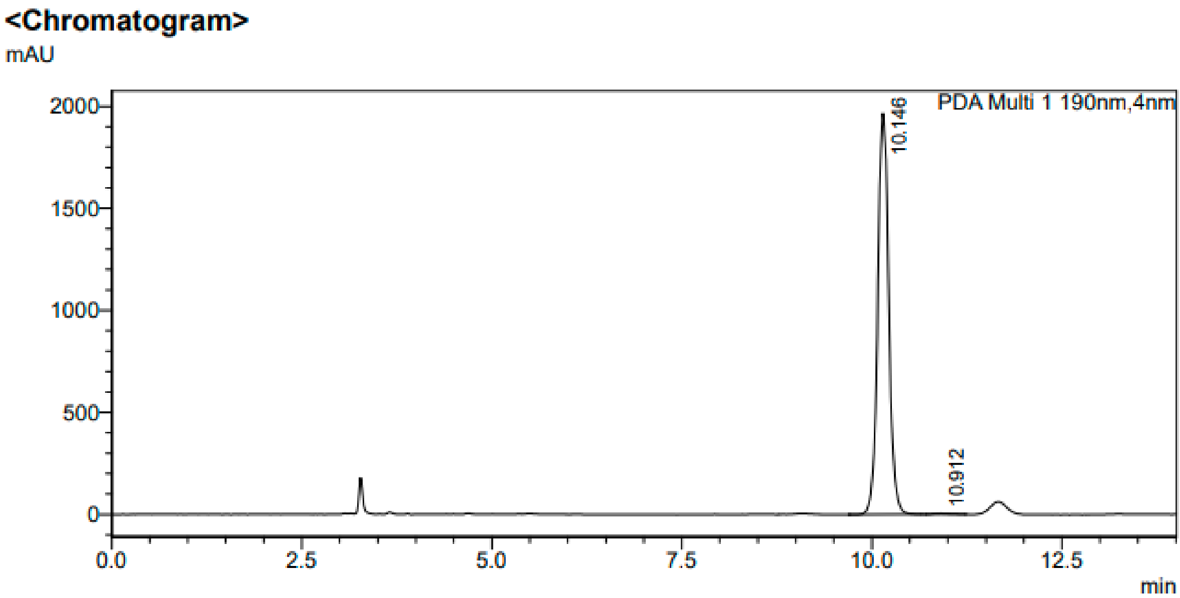

<Peak Table>

PDA Ch1 190nm

Peak#	Ret. Time	Area	Height	Conc.	Unit	Mark	Name
1	10.146	19910145	1965837	0.000		M	
2	10.912	62354	4140	0.000		M	
Total		19972499	1969976				

Supplementary Figure 11. HPLC spectrum of (*R,E*)-**4e**

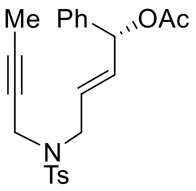

Chemical Formula: $C_{23}H_{25}NO_4S$
Exact Mass: 411.1504

Purification by column chromatography (PE/EA = 5/1) gave (*R*,*E*)-4-((*N*-(but-2-yn-1-yl)-4-methylphenyl)sulfonamido)-1-phenylbut-2-en-1-yl acetate (*R*,*E*)-**4f** as a colorless oil (509.9 mg, 62% yield, 99% ee).

^{1}H NMR (600 MHz, $CDCl_3$): δ 7.72–7.68 (m, 2H), 7.36–7.33 (m, 2H), 7.32–7.28 (m, 3H), 7.27 (s, 1H), 7.26 (s, 1H), 6.23 (d, *J* = 5.8 Hz, 1H), 5.91–5.84 (m, 1H), 5.66–5.59 (m, 1H), 4.00–3.94 (m, 2H), 3.81 (d, *J* = 6.5 Hz, 2H), 2.41 (s, 3H), 2.09 (s, 3H), 1.51 (t, *J* = 2.4 Hz, 3H);

^{13}C NMR (151 MHz, $CDCl_3$): δ 169.8, 143.3, 138.6, 136.0, 133.2, 129.2, 128.6, 128.3, 127.8, 127.1, 126.6, 81.6, 75.0, 71.5, 47.7, 36.5, 21.5, 21.2, 3.2;

HRMS: (ESI) calcd for $C_{23}H_{26}NO_4S^+$ ($[M+H]^+$): 412.1577; found: 412.1574;

HPLC conditions: AD-H column (15% ^{i}PrOH in hexane, 1.0 mL/min, λ = 204 nm, 30 °C), t_R (major) = 12.0 min, t_R (minor) = 15.0 min;

Optical Rotation: $[\alpha]^{25}_D$ = 1.2 (*c* = 0.33, $CHCl_3$) for 99% ee.

<Chromatogram>

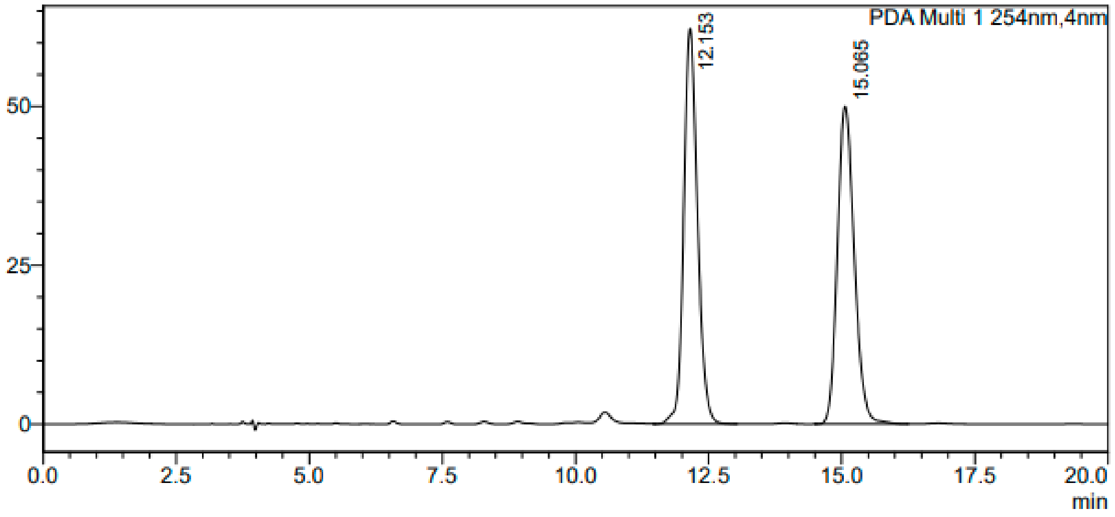

<Peak Table>

PDA Ch1 254nm

Peak#	Ret. Time	Area	Height	Conc.	Unit	Mark	Name
1	12.153	1116928	62187	0.000		M	
2	15.065	1098832	49994	0.000		M	
Total		2215760	112181				

Supplementary Figure 12. HPLC spectrum of racemic-(*E*)-**4f**

<Chromatogram>

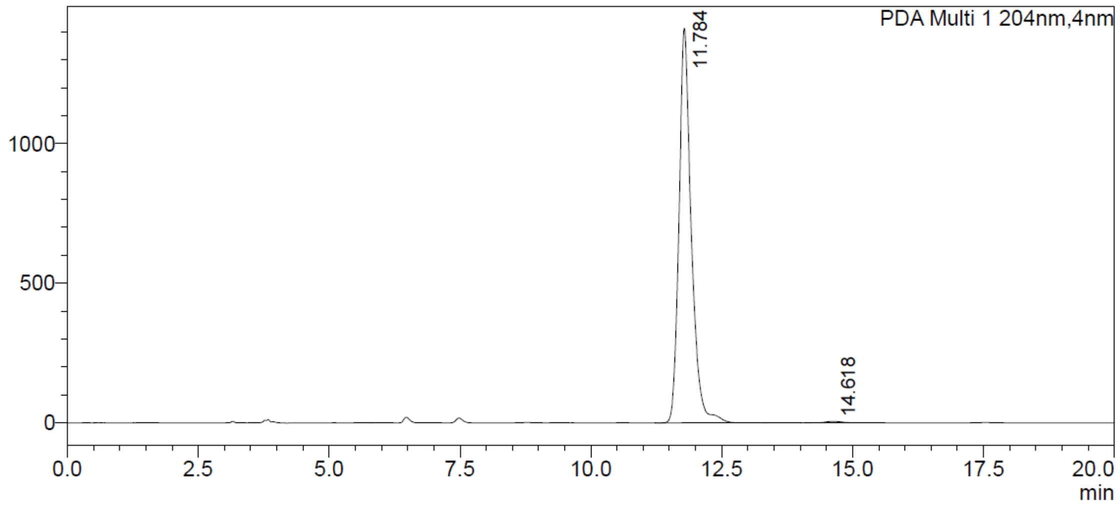

<Peak Table>

PDA Ch1 204nm

Peak#	Ret. Time	Area	Height	Conc.	Unit	Mark	Name
1	11.784	23016462	1412453	0.000		M	
2	14.618	89238	4513	0.000		M	
Total		23105701	1416965				

Supplementary Figure 13. HPLC spectrum of (*R*,*E*)-**4f**

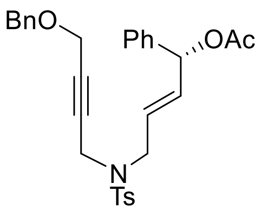

Chemical Formula: $C_{30}H_{31}NO_5S$
Exact Mass: 517.1923

Purification by column chromatography (PE/EA = 5/1) gave (*R*,*E*)-4-((N-(4-(benzyloxy)but-2-yn-1-yl)-4-methylphenyl) sulfonamido)-1-phenylbut-2-en-1-yl acetate (*R*,*E*)-**4g** as a colorless oil (517.2 mg, 50% yield, 99% ee).

^{1}H NMR (600 MHz, $CDCl_3$): δ 7.73–7.70 (m, 2H), 7.36–7.32 (m, 4H), 7.32–7.28 (m, 4H), 7.26–7.22 (m, 4H), 6.24 (dd, *J* = 5.7, 1.3 Hz, 1H), 5.93–5.87 (m, 1H), 5.69–5.61 (m, 1H), 4.36 (s, 2H), 4.12 (t, *J* = 1.9 Hz, 2H), 3.89 (t, *J* = 1.9 Hz, 2H), 3.85 (dd, *J* = 6.6, 1.2 Hz, 2H), 2.33 (s, 3H), 2.08 (s, 3H);

^{13}C NMR (151 MHz, $CDCl_3$): δ 169.8, 143.5, 138.5, 137.1, 135.8, 133.6, 129.4, 128.6, 128.4, 128.2, 127.9, 127.8, 127.7, 127.0, 126.2, 79.0, 74.9, 71.3, 56.9, 47.8, 36.3, 21.4, 21.1;

HRMS: (ESI) calcd for $C_{30}H_{35}N_2O_5S^+$ ($[M+NH_4]^+$): 535.2261; found: 535.2263;

HPLC conditions: OD-H column (20% ^{i}PrOH in hexane, 1.0 mL/min, λ = 195 nm, 30 °C), t_R (major) = 16.3 min, t_R (minor) = 19.0 min;

Optical Rotation: $[\alpha]^{25}_D = 3.5$ (*c* = 0.17, $CHCl_3$) for 99% ee.

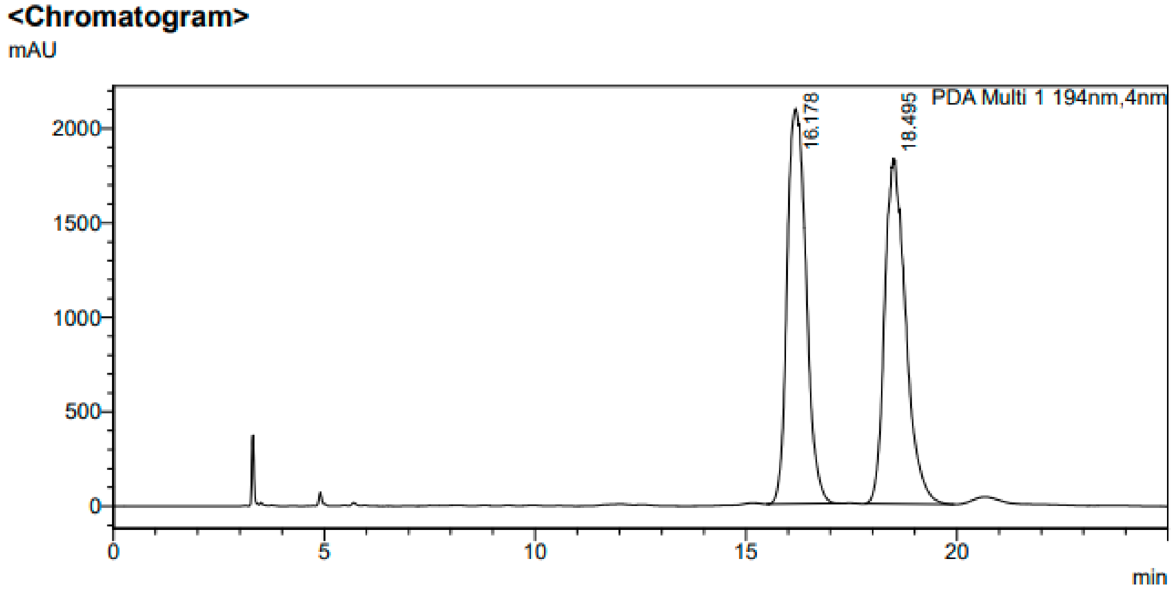

<Peak Table>

PDA Ch1 194nm

Peak#	Ret. Time	Area	Height	Conc.	Unit	Mark	Name
1	16.178	65760839	2093896	0.000		M	
2	18.495	63705189	1830298	0.000		M	
Total		129466028	3924193				

Supplementary Figure 14. HPLC spectrum of racemic-(*E*)-**4g**

<Chromatogram>

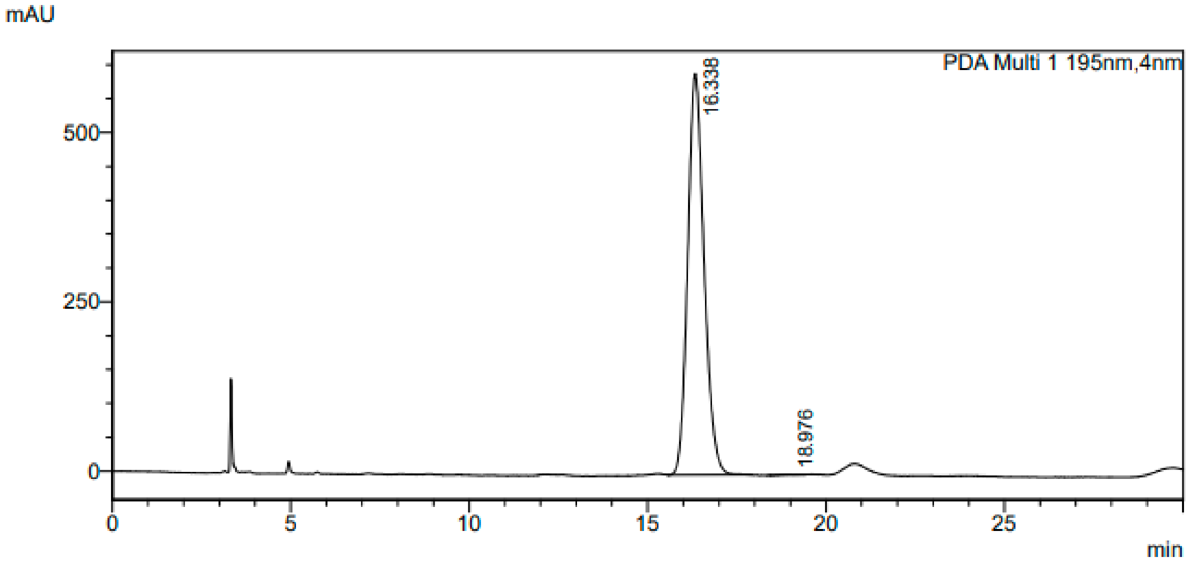

<Peak Table>

PDA Ch1 195nm

Peak#	Ret. Time	Area	Height	Conc.	Unit	Mark	Name
1	16.338	18834851	592315	0.000		M	
2	18.976	17372	758	0.000		M	
Total		18852223	593072				

Supplementary Figure 15. HPLC spectrum of (*R,E*)-**4g**

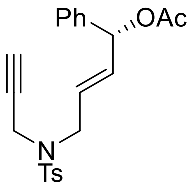

Chemical Formula: $C_{22}H_{23}NO_4S$
Exact Mass: 397.1348

Purification by column chromatography (PE/EA = 5/1) gave (*R,E*)-4-((4-methyl-*N*-(prop-2-yn-1-yl)phenyl)sulfonamido)-1-phenylbut-2-en-1-yl acetate (*R*,*E*)-**4h** as a colorless oil (46% yield, 99% ee).

^{1}H NMR (600 MHz, $CDCl_3$) δ 7.73-7.67 (m, 2H), 7.36-7.32 (m, 2H), 7.32-7.27 (m, 4H), 7.26 (s, 1H), 6.23 (dd, *J* = 5.8, 1.3 Hz, 1H), 5.93-5.86 (m, 1H), 5.67-5.59 (m, 1H), 4.04 (d, *J* = 2.5 Hz, 2H), 3.84 (d, *J* = 6.6 Hz, 2H), 2.41 (s, 3H), 2.08 (s, 3H), 1.98 (t, *J* = 2.5 Hz, 1H); ^{13}C NMR (151 MHz, $CDCl_3$) δ 169.9, 143.6, 138.5, 135.8, 133.7, 129.5, 128.6, 128.3, 127.7, 127.0, 126.2, 76.4, 75.0, 73.9, 47.7, 36.0, 21.5, 21.2.

HRMS: (ESI) calcd for $C_{22}H_{24}NO_4S^+$ ([M+H]$^+$): 398.1421; found: 398.1420;

HPLC conditions: OD-H column (20% ^{i}PrOH in hexane, 1.0 mL/min, λ = 196 nm, 30 °C), t_R (minor) = 8.6 min, t_R (major) = 9.1 min;

Optical Rotation: $[\alpha]^{25}_D$ = 4.9 (*c* = 0.87, $CHCl_3$) for 99% ee;

<Chromatogram>

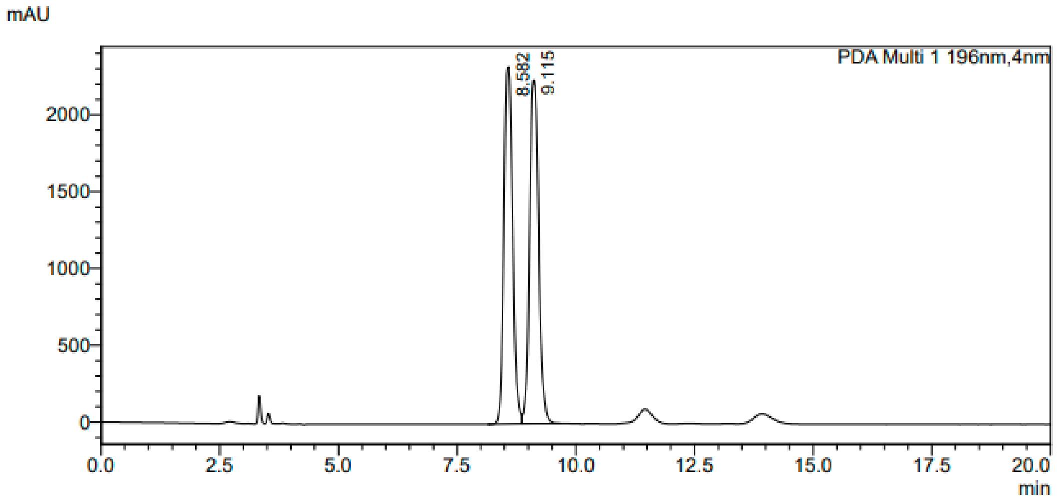

<Peak Table>

PDA Ch1 196nm

Peak#	Ret. Time	Area	Height	Conc.	Unit	Mark	Name
1	8.582	29508240	2324080	0.000		M	
2	9.115	29571322	2235431	0.000		V M	
Total		59079562	4559511				

Supplementary Figure 16. HPLC spectrum of racemic-(*E*)-**4h**

<Chromatogram>

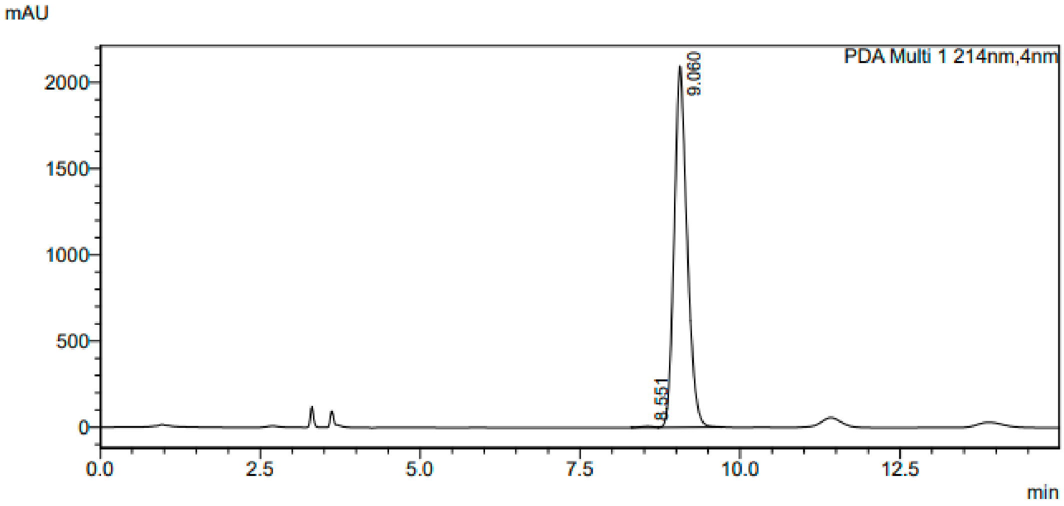

<Peak Table>

PDA Ch1 214nm

Peak#	Ret. Time	Area	Height	Conc.	Unit	Mark	Name
1	8.551	92847	6831	0.000		M	
2	9.060	29356402	2094652	0.000		V M	
Total		29449249	2101483				

Supplementary Figure 17. HPLC spectrum of (*R*,*E*)-**4h**

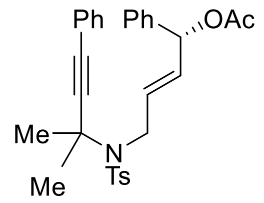

Chemical Formula: $C_{30}H_{31}NO_4S$
Exact Mass: 501.1974

Purification by column chromatography (PE/EA = 5/1) gave (*R,E*)-4-((4-methyl-*N*-(2-methyl-4-phenylbut-3-yn-2-yl)phenyl)sulfonamido)-1-phenylbut-2-en-1-yl acetate (*R,E*)-**4i** as a colorless oil (461.1 mg, 46% yield, 99% ee).

^{1}H NMR (600 MHz, $CDCl_3$): δ 7.66–7.63 (m, 2H), 7.29–7.26 (m, 2H), 7.26–7.21 (m, 3H), 7.21–7.16 (m, 3H), 7.14–7.09 (m, 4H), 6.21 (dd, *J* = 5.9, 1.1 Hz, 1H), 5.94–5.87 (m, 1H), 5.87–5.82 (m, 1H), 4.20–4.15 (m, 2H), 2.29 (s, 3H), 2.00 (s, 3H), 1.66 (d, *J* = 1.8 Hz, 6H);

^{13}C NMR (151 MHz, $CDCl_3$): δ 169.9, 142.9, 139.5, 139.0, 131.5, 131.2, 131.1, 129.4, 128.5, 128.3, 128.2, 128.1, 127.3, 127.1, 91.4, 83.8, 75.4, 57.2, 49.4, 30.7, 30.6, 21.4, 21.2;

HRMS: (ESI) calcd for $C_{30}H_{32}NO_4S^+$ ($[M+H]^+$): 502.2047; found: 502.2044;

HPLC conditions: AD-H column (15% ^{i}PrOH in hexane, 1.0 mL/min, λ = 254 nm, 30 °C), t_R (major) = 8.9 min, t_R (minor) = 10.2 min;

Optical Rotation: $[\alpha]^{25}_D$ = 11.5 (*c* = 0.17, $CHCl_3$) for 99% ee.

<Chromatogram>

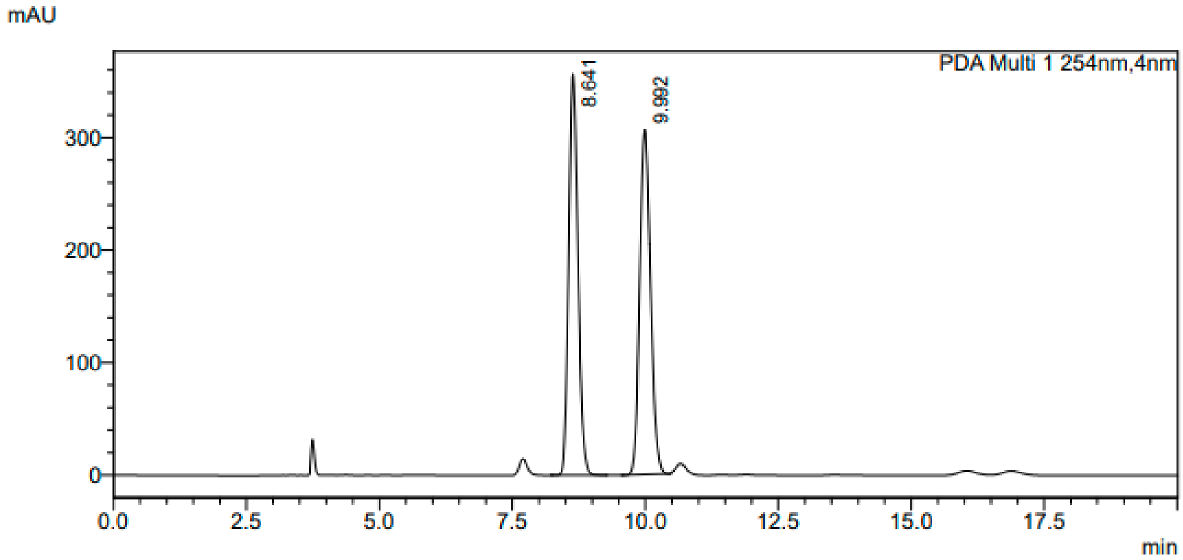

<Peak Table>

PDA Ch1 254nm

Peak#	Ret. Time	Area	Height	Conc.	Unit	Mark	Name
1	8.641	4366609	356251	0.000		M	
2	9.992	4369362	305835	0.000		M	
Total		8735971	662086				

Supplementary Figure 18. HPLC spectrum of racemic-(*E*)-**4i**

<Chromatogram>

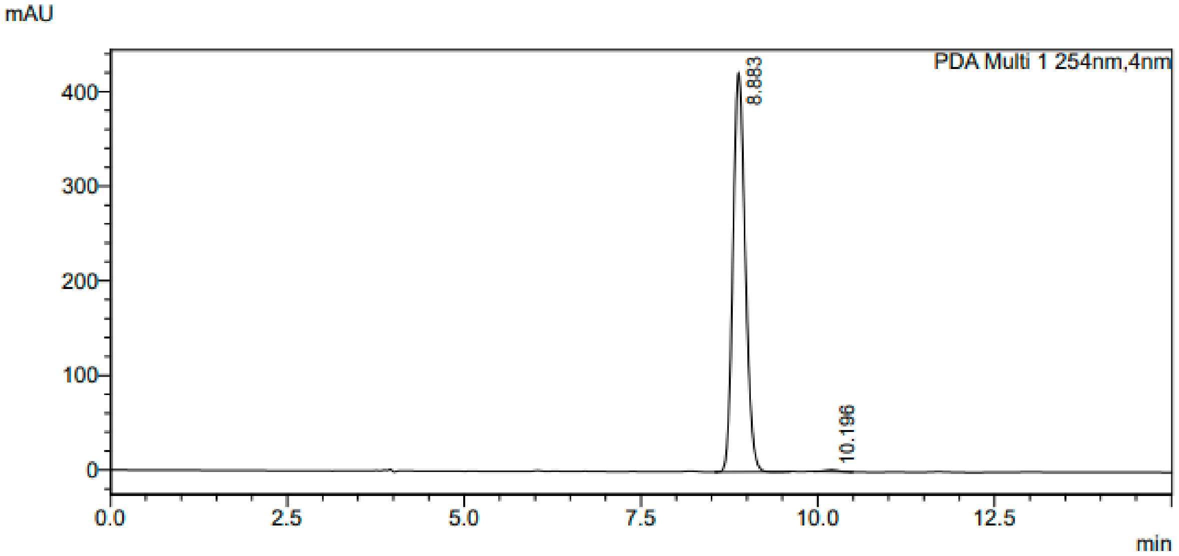

<Peak Table>

PDA Ch1 254nm

Peak#	Ret. Time	Area	Height	Conc.	Unit	Mark	Name
1	8.883	5093297	422116	0.000		M	
2	10.196	24700	1792	0.000		M	
Total		5117998	423908				

Supplementary Figure 19. HPLC spectrum of (*R*,*E*)-**4i**

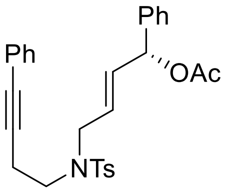

Chemical Formula: $C_{29}H_{29}NO_4S$
Exact Mass: 487.1817

Purification by column chromatography (PE/EA = 10/1) gave (*R*,*E*)-4-((4-methyl-*N*-(4-phenylbut-3-yn-1-yl)phenyl) sulfonamido)-1-phenylbut-2-en-1-yl acetate (*R*,*E*)-**4j** as a colorless oil (643.1 mg, 66% yield, 99% ee).

^{1}H NMR (600 MHz, $CDCl_3$): δ 7.72–7.67 (m, 2H), 7.36–7.31 (m, 4H), 7.31–7.27 (m, 5H), 7.26 (s, 1H), 7.25 (d, *J* = 8.0 Hz, 2H), 6.20 (d, *J* = 5.9 Hz, 1H), 5.86–5.77 (m, 1H), 5.64–5.56 (m, 1H), 3.96–3.87 (m, 2H), 3.35 (t, *J* = 7.5 Hz, 2H), 2.64 (t, *J* = 7.4 Hz, 2H), 2.40 (s, 3H), 2.06 (s, 3H);

^{13}C NMR (151 MHz, $CDCl_3$): δ 169.8, 143.4, 138.5, 136.8, 133.0, 131.5, 129.7, 128.6, 128.3, 128.2, 127.9, 127.4, 127.1, 127.0, 123.3, 86.4, 82.3, 75.0, 50.0, 46.3, 21.5, 21.2, 20.3;

HRMS: (ESI) calcd for $C_{29}H_{30}NO_4S^+$ ($[M+H]^+$): 488.1890; found: 488.1898;

HPLC conditions: AD-H column (15% ^{i}PrOH in hexane, 1.0 mL/min, λ = 204 nm, 30 °C), t_R (major) = 15.8 min, t_R (minor) = 16.6 min;

Optical Rotation: $[\alpha]^{25}_D$ = -3.2 (*c* = 0.17, $CHCl_3$) for 99% ee.

<Chromatogram>

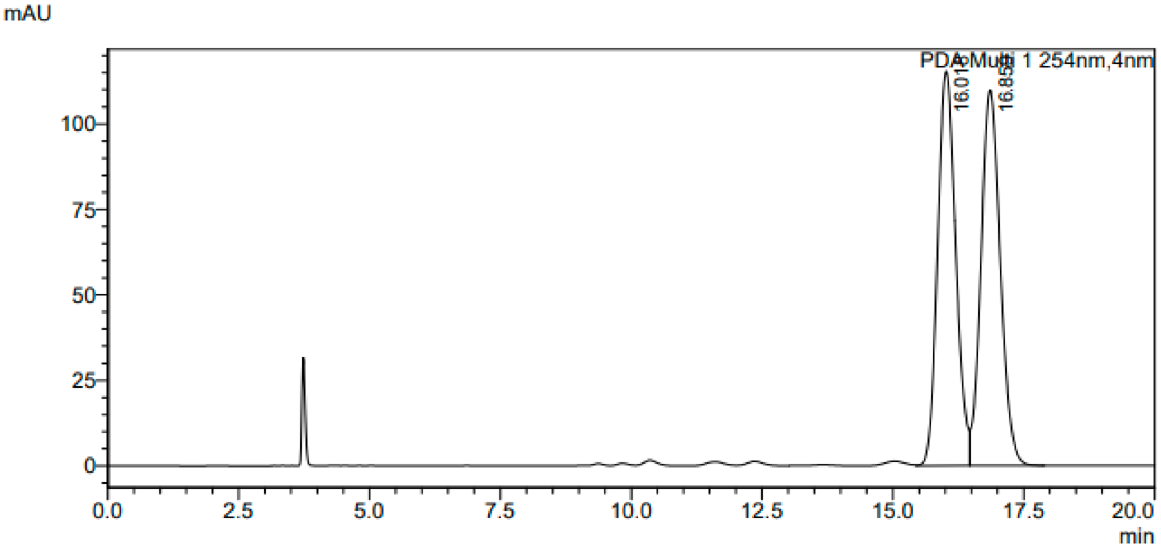

<Peak Table>

PDA Ch1 254nm

Peak#	Ret. Time	Area	Height	Conc.	Unit	Mark	Name
1	16.018	2781701	115381	0.000			
2	16.859	2816597	109785	0.000		V	
Total		5598298	225166				

Supplementary Figure 20. HPLC spectrum of racemic-(*E*)-**4j**

<Chromatogram>

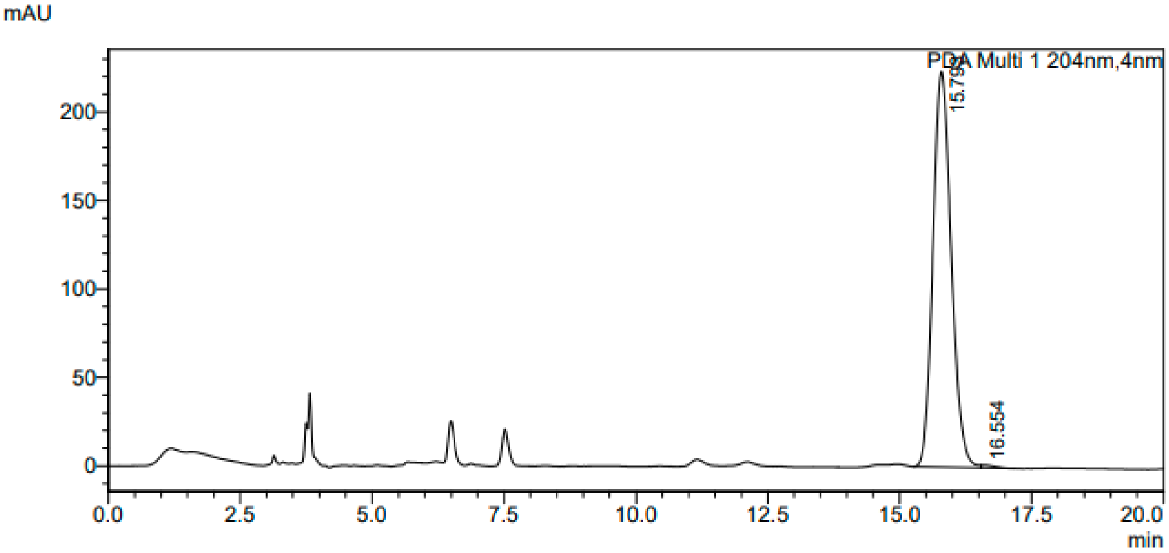

<Peak Table>

PDA Ch1 204nm

Peak#	Ret. Time	Area	Height	Conc.	Unit	Mark	Name
1	15.793	5393868	223494	0.000		M	
2	16.554	27524	1591	0.000		V M	
Total		5421393	225086				

Supplementary Figure 21. HPLC spectrum of (*R*,*E*)-**4j**

3.7 Procedures for the synthesis of 1,6-enyne (*Z*)-4a

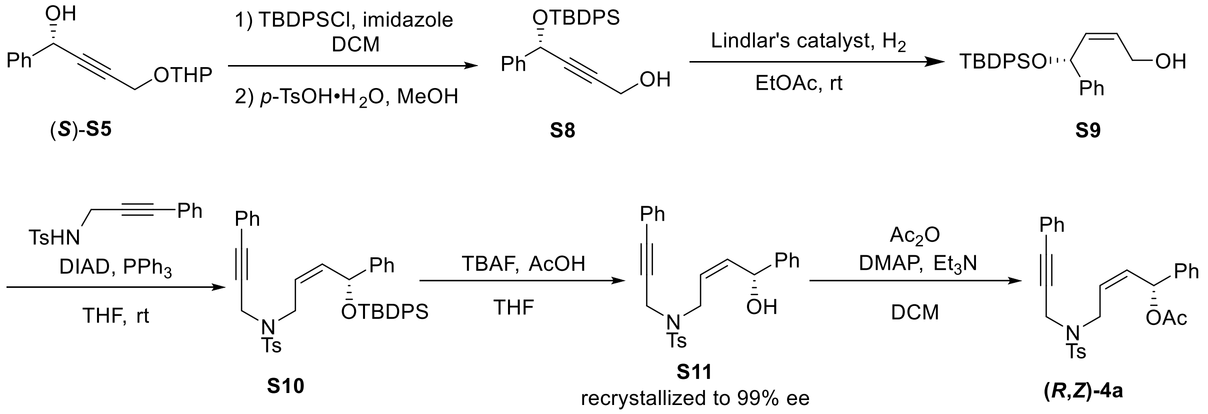

Step I: (*S*)-**S8** was prepared according to the literature procedure.[4] To a solution of (*S*)-**S5** (4.9 g, 20.0 mmol) and 1*H*-imidazole (2.0 g, 30 mmol) in dry DCM (30 mL) was added TBDPSCl (6.6 g, 24.0 mmol) at 0 °C, then the mixture was stirred at room temperature for 5 h. Then H_2O (40 mL) was added. The organic layer was separated and the aqueous layer extracted with DCM (2 ×20 mL). The combined organic layer was dried over Na_2SO_4 and concentrated to afford the crude product. To a solution of above crude product in MeOH (20 mL) was added PTSA•H_2O (184.8 mg, 1.0 mmol). After stirring at room temperature for 1 hour, the mixture was quenched with a saturated aqueous solution of $NaHCO_3$ and extracted with EtOAc. The combined organic layers were dried over Na_2SO_4, filtered, and concentrated. The residue was purified by chromatography on silica gel, eluting with PE/EA = 2/1 to afford the **S8** as a colorless oil (6.2 g, 78% yield).

Step II: To a 100 mL rounded-bottomed flask was added Lindlar's catalyst (750 mg). The flask was evacuated and refilled with H_2 three times. Under a H_2 airbag, a solution of **S8** (2.43 g, 15 mmol) in EtOAc (20 mL) was added, and the reaction mixture was stirred at room temperature for 3 hours. The reaction mixture was filtered through a celite pad, and the filtrate was concentrated and purified by chromatography on silica gel, eluting with PE/EA = 2/1 to afford the (*R*,*Z*)-1-phenylbut-2-ene-1,4-diol (**S9**) as a colorless oil (5.6 g, 93% yield).

Step III: To the solution of **S9** (4.0 g, 10 mmol), 4-methyl-*N*-(3-phenylprop-2-yn-1-yl)benzenesulfonamide (2.9 g, 10 mmol) and PPh_3 (2.9 g, 11 mmol) in THF (100 mL) under N_2 was added DIAD (2.2 g, 11mmol) dropwise at 0 °C. The reaction mixture was stirred at room temperature overnight. Then the mixture was concentrated under vacuum and purified

by flash column chromatography (PE/EA = 30/1) to give the product **S10** as a colorless oil (5.4 g, 80% yield).

Step IV: To a solution of **S10** (3.35 g, 5 mmol) in THF (15 mL) was added AcOH (36 mg, 6 mmol) at 0 °C. Then TBAF (10 mL, 1.0 M in THF) was added dropwise at the same temperature. The reaction mixture was stirred at room temperature for addition 12 hours. The solvent was diluted with water. The organic layer was separated and the aqueous layer was extracted with EtOAc. The combined organic layers were dried over Na_2SO_4, filtered and concentrated. Purification by column chromatography (PE/EA = 4/1) afforded **S11** as a white solid (1.94 g, 90% yield), which was recrystallization in DCM/^{i}PrOH and the final ee value was determined to be 99%.

Step V: To a solution of **S11** (1.72 g, 4.0 mmol, 99% ee) in CH_2Cl_2 (15 mL) was added Et_3N (0.94 mL, 6.8 mmol) and DMAP (17.0 mg, 0.14 mmol) at 0 °C. Then acetic anhydride (1.27 mL, 13.5 mmol) was added dropwise at the same temperature. The reaction mixture was stirred at room temperature for addition 2 hours. The solvent was removed under reduced pressure. Purification by column chromatography (25% EtOAc in petroleum ether) gave (*R*,*Z*)-**4a** as a white solid (1.6 g, 84% yield, 99% ee).

^{1}H NMR (600 MHz, $CDCl_3$): δ 7.78 (d, *J* = 7.9 Hz, 2H), 7.46–7.15 (m, 10H), 7.07 (d, *J* = 7.5 Hz, 2H), 6.53 (d, *J* = 9.2 Hz, 1H), 5.85 (t, *J* = 10.1 Hz, 1H), 5.67 (dt, *J* = 10.9, 7.0 Hz, 1H), 4.35 (d, *J* = 18.4 Hz, 1H), 4.20 (dd, *J* = 16.4, 8.5 Hz, 2H), 4.08 (dd, *J* = 14.9, 6.5 Hz, 1H), 2.32 (s, 3H), 2.05 (s, 3H);

^{13}C NMR (151 MHz, $CDCl_3$): δ 169.9, 143.6, 139.1, 135.8, 132.6, 131.5, 129.6, 128.7, 128.4, 128.2, 128.1, 127.8, 127.6, 126.7, 122.1, 85.9, 81.7, 71.2, 43.9, 37.2, 21.4, 21.2;

HRMS: (ESI) calcd for $C_{28}H_{28}NO_4S^+$ ($[M+H]^+$): 474.1734; found: 474.1729;

HPLC conditions: AD-H column (15% ^{i}PrOH in hexane, 1.0 mL/min, λ = 254 nm, 30 °C), t_R (minor) = 10.5 min, t_R (major) = 11.1 min;

Optical Rotation: $[\alpha]^{25}_D$ = 61.0 (*c* = 0.13, $CHCl_3$) for 99% ee.

With the use of (*R*)-**S8**, (*S,Z*)-**4a** product was also prepared in 99% ee.

HPLC conditions: AD-H column (15% ^{i}PrOH in hexane, 1.0 mL/min, λ = 254 nm, 30 °C), t_R (major) = 10.5 min, t_R (minor) = 11.1 min;

Optical Rotation: $[\alpha]^{25}_D$ = -106.7 (*c* = 0.07, $CHCl_3$) for 99% ee.

<Chromatogram>

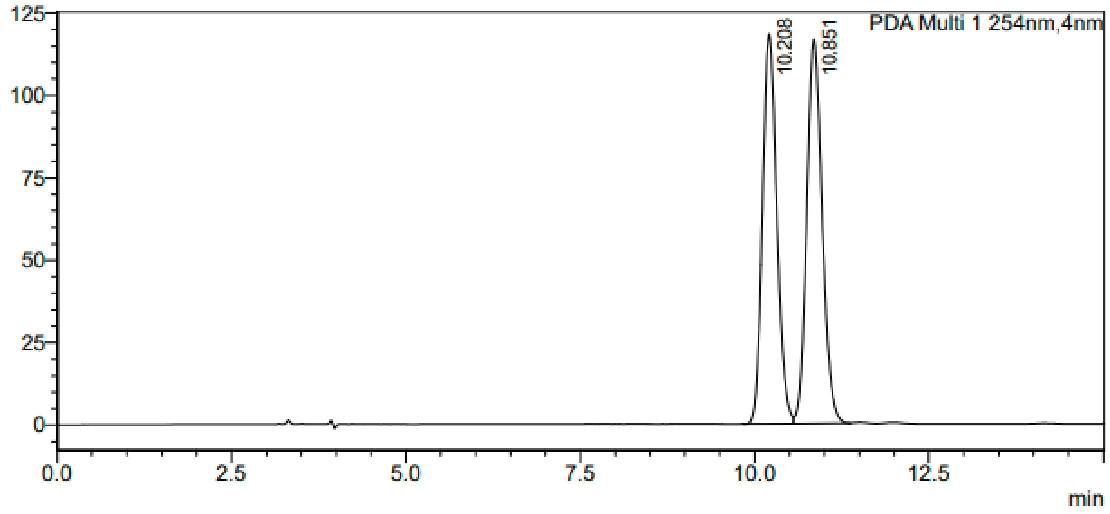

<Peak Table>

PDA Ch1 254nm

Peak#	Ret. Time	Area	Height	Conc.	Unit	Mark	Name
1	10.208	1702679	118274	0.000		M	
2	10.851	1798122	116491	0.000		V M	
Total		3500800	234764				

Supplementary Figure 22. HPLC spectrum of racemic-(*Z*)-**4a**

<Chromatogram>

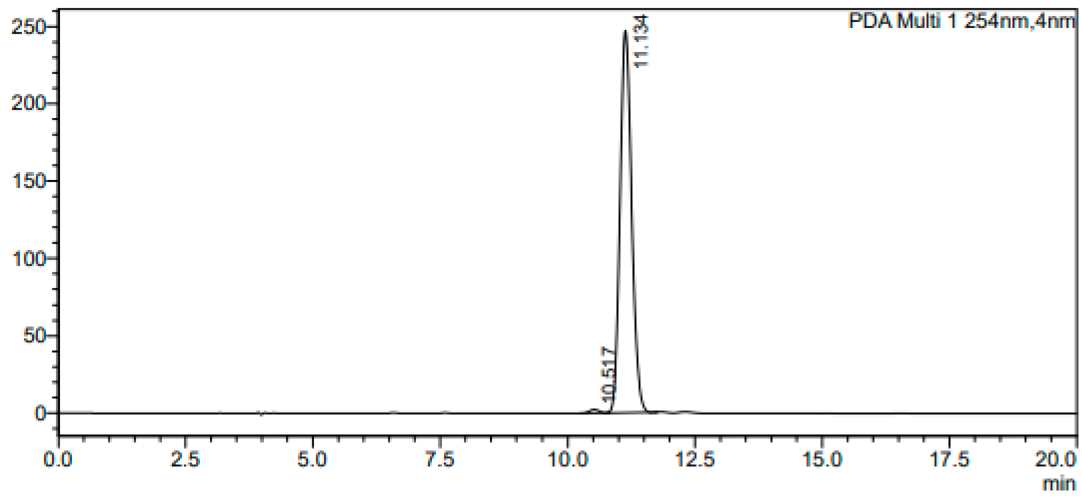

<Peak Table>

PDA Ch1 254nm

Peak#	Ret. Time	Area	Height	Conc.	Unit	Mark	Name
1	10.517	30663	2282	0.000		M	
2	11.134	3917379	247043	0.000		V M	
Total		3948042	249325				

Supplementary Figure 23. HPLC spectrum of (*R*,*Z*)-**4a**

<Chromatogram>

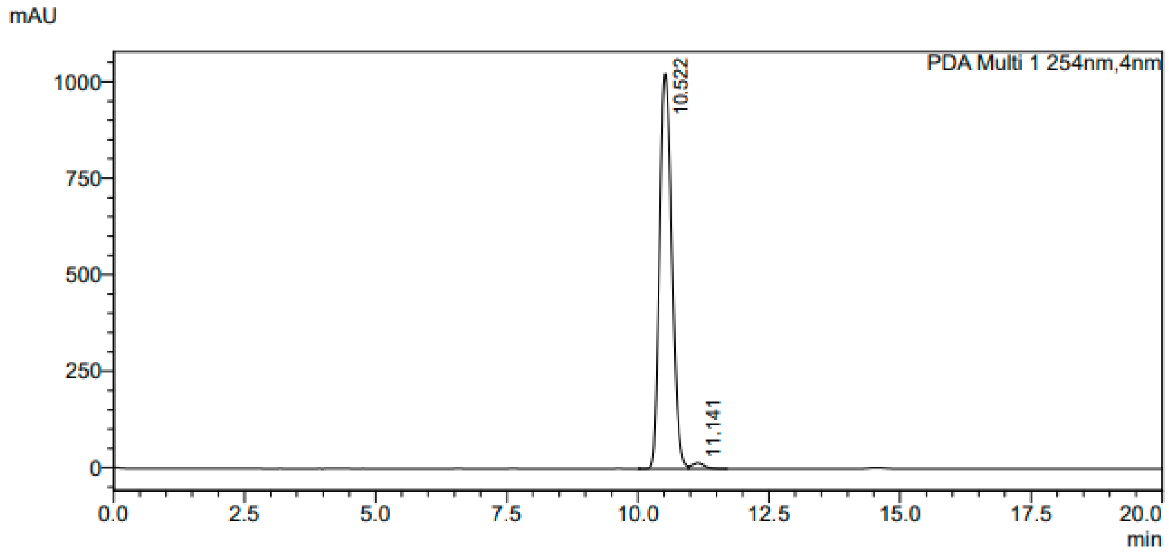

<Peak Table>

PDA Ch1 254nm

Peak#	Ret. Time	Area	Height	Conc.	Unit	Mark	Name
1	10.522	16726925	1023357	0.000		M	
2	11.141	234307	14810	0.000		V M	
Total		16961233	1038167				

Supplementary Figure 24. HPLC spectrum of (*S,Z*)-**4a**

3.8 Procedure for the synthesis of substrate 4k

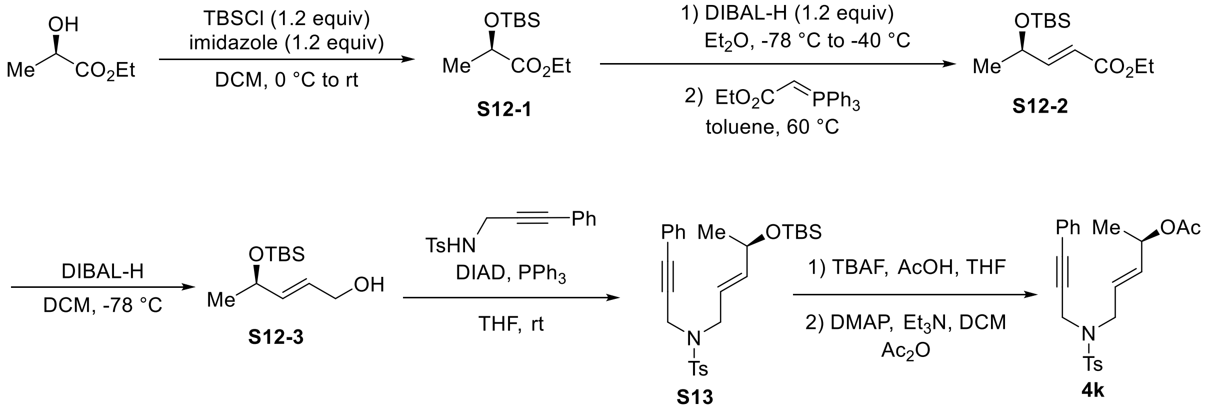

Step I: According to the literature procedure.[5] To a solution of (*R*)-ethyl lactate (6.0 g, 50.8 mmol) and 1*H*-imidazole (4.2 g, 61 mmol) in dry DCM (60 mL) was added TBSCl (9.2 g, 61 mmol) at 0 °C, then the mixture was stirred at room temperature for 5 h. Then H_2O (40 mL) was added. The organic layer was separated and the aqueous layer was extracted with DCM (2 ×20 mL). The combined organic layer was dried over Na_2SO_4. The residue obtained after evaporation of solvent was purified by silica gel column chromatography (EA/PE = 1:20) to afford **S12-1** (11.0 g, 93% yield) as a colorless liquid.

Step II: To a stirred solution of **S12-1** (11.0 g, 47.3 mmol) in Et_2O (200 mL), a solution of diisobutyl aluminium hydride (60 mL, 1.0 M in THF) was added dropwise at –78 °C and the resulting mixture was stirred at –40 °C for 1 h. After completion of the reaction (monitored by TLC), the reaction mixture was diluted with sodium sulfate decahydrate and stirred for further 4 h. The organic phase was filtrated with kieselguhr and washed with EtOAc. The combined organic layer was concentrated under reduced pressure to afford crude aldehyde. To a solution of above crude aldehyde in toluene (100 mL) at 60 °C was added $Ph_3P{=}CHCO_2Et$ (24 g, 69 mmol) and the reaction mixture was stirred for 3 h. After completion of reaction (monitored by TLC), the solvent was distilled off under reduced pressure and the residue was purified by silica gel column chromatography (PE/EA = 10/1) to afford **S12-2** (4.9 g, 40%) as a slightly yellow liquid.

Step III: To a solution of **S12-2** (4.9 g, 19 mmol) in dry DCM (50 mL), a solution of diisobutyl aluminium hydride (42 mL, 1.0 M in THF) was added dropwise at –78 °C and then stirred at this temperature for 1 h. After completion of the reaction (monitored by TLC), it was diluted with sodium sulfate decahydrate and stirred for further 4 h. The organic phase was filtrated with kieselguhr and washed with EtOAc multiple times. The combined organic layer was concentrated under reduced pressure and purified by silica gel column chromatography (EA/PE = 1:10) to afford **S12-3** (2.4 g, 59%) as a colorless liquid.

Step IV: To the solution of **S12-3** (476 mg, 2.2 mmol), 4-methyl-*N*-(3-phenylprop-2-yn-1-yl)benzenesulfonamide (570 mg, 2.0 mmol) and PPh_3 (786 mg, 4.8 mmol) in THF (30 mL) was added DIAD (2.6 mmol, 525.2 mg) dropwise at 0 °C under N_2. Then the reaction mixture was stirred at room temperature overnight. The mixture was concentrated under vacuum and purified by flash column chromatography (PE/EA = 10/1) to give the product **S13** as a colorless liquid (870 mg, 90% yield).

Step V: To a solution of **S13** (869 mg, 1.8 mmol) in THF (10 mL) was added AcOH (130 mg, 2.2 mmol) at 0 °C. Then TBAF (3.6 mL, 1.0 M in THF) was added dropwise at the same temperature. The reaction mixture was stirred at room temperature for 12 hours. The solvent was diluted with water. The organic layer was separated and the aqueous layer was extracted with EtOAc. The combined organic layers were dried over Na_2SO_4, filtered and concentrated. The residue was direct used without purified for the next step. To a solution of the residue in

CH_2Cl_2 (8 mL) was added Et_3N (0.72 mL, 5.1 mmol) and DMAP (64.0 mg, 0.051 mmol) at 0 °C. Then acetic anhydride (0.52 mL, 5.1 mmol) was added dropwise at the same temperature. The reaction mixture was stirred at room temperature for addition 2 hours. The solvent was removed under reduced pressure. Purification by column chromatography (PE/EA = 20/1) gave **4k** as a yellow solid (632 mg, 90% yield, 98% ee).

^{1}H NMR (600 MHz, $CDCl_3$): δ 7.80–7.74 (m, 2H), 7.30–7.27 (m, 1H), 7.26–7.22 (m, 4H), 7.08–7.02 (m, 2H), 5.79–5.73 (m, 1H), 5.69–5.62 (m, 1H), 5.37–5.31 (m, 1H), 4.28 (s, 2H), 3.91–3.85 (m, 2H), 2.33 (s, 3H), 2.02 (d, J = 1.0 Hz, 3H), 1.29 (d, J = 6.5 Hz, 3H);

^{13}C NMR (151 MHz, $CDCl_3$): δ 170.1, 143.5, 135.8, 135.0, 131.4, 129.5, 128.3, 128.1, 127.7, 125.2, 122.1, 85.7, 81.5, 69.8, 47.9, 36.7, 21.3, 21.2, 20.0;

HRMS: (ESI) calcd for $C_{23}H_{26}NO_4S^+$ ($[M+H]^+$): 412.1577; found: 412.1567;

HPLC conditions: OD-H column (15% ^{i}PrOH in hexane, 1.0 mL/min, λ = 254 nm, 30 °C), t_R (major) = 9.0 min, t_R (minor) = 9.6 min;

Optical Rotation: $[\alpha]^{25}_D$ = 75.1 (c = 0.17, $CHCl_3$) for 98% ee.

<Chromatogram>

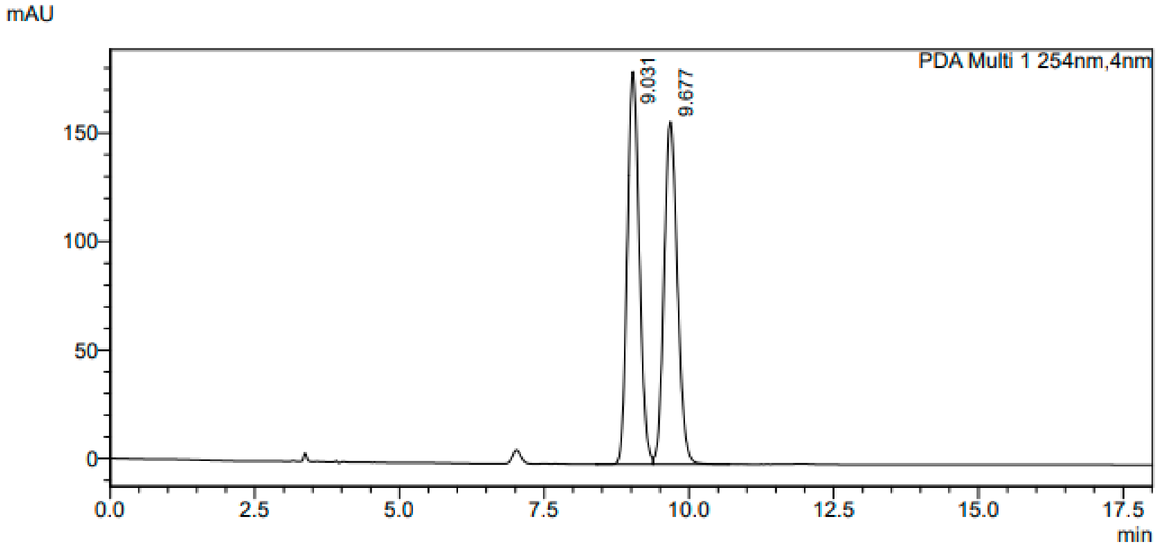

<Peak Table>

PDA Ch1 254nm

Peak#	Ret. Time	Area	Height	Conc.	Unit	Mark	Name
1	9.031	2600813	180991	0.000		M	
2	9.677	2533740	157925	0.000		V M	
Total		5134553	338916				

Supplementary Figure 25. HPLC spectrum of racemic-(E)-**4k**

<Chromatogram>

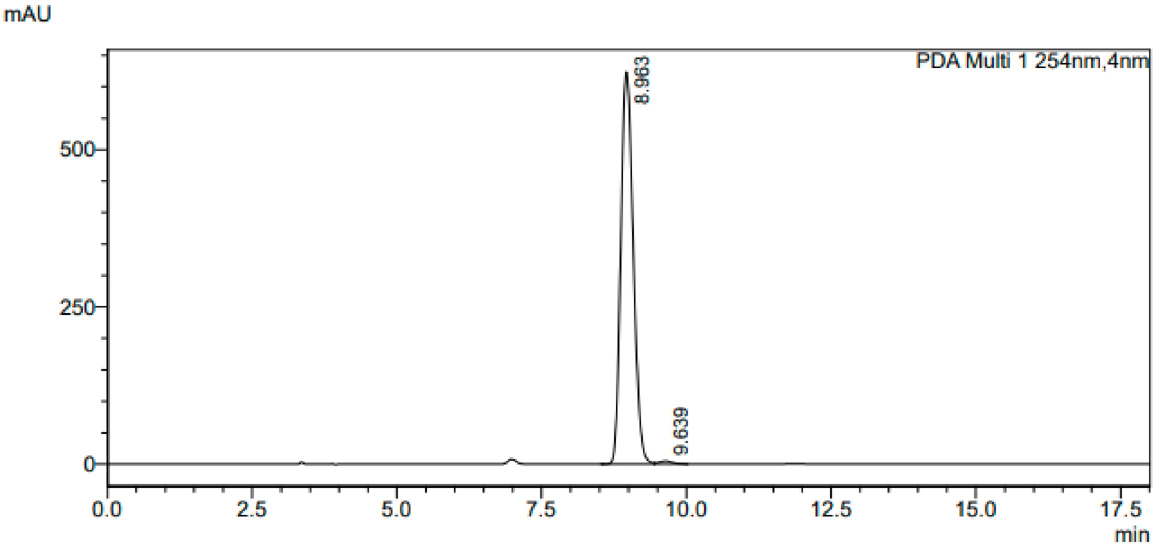

<Peak Table>

PDA Ch1 254nm

Peak#	Ret. Time	Area	Height	Conc.	Unit	Mark	Name
1	8.963	9336086	623685	0.000		M	
2	9.639	78274	4829	0.000		V M	
Total		9414360	628515				

Supplementary Figure 26. HPLC spectrum of (*R*,*E*)-**4k**

3.9 Procedure for the synthesis of Substrate 4l

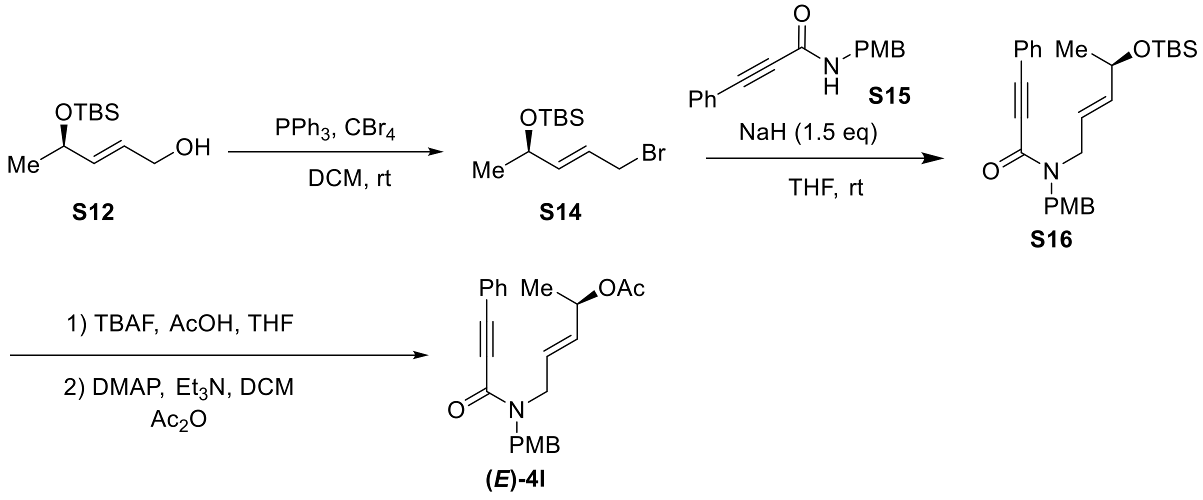

Step I: To a 100 mL rounded-bottomed flask was added **S12** (3.28 g, 15.0 mmol), CBr_4 (9.9 g, 30 mmol), PPh_3(7.9 g, 30 mmol), and anhydrous DCM (60 mL) at 0 °C. The mixture was stirred at room temperature for 3 hours. The solvent was removed under reduced pressure and the residue was purified by column chromatography (PE/EA = 100/1) to give **S14** as a faint yellow oil (3.46 g, 82% yield).

Step II: To a solution of **S14** (2.64 g, 10 mmol) in anhydrous THF (20 mL) was added NaH (360 mg, 15 mmol) at 0 °C. The mixture was stirred at 0 °C for 30 min, and **S15** (3.34 g, 12 mmol) was added to the reaction mixture. The mixture was stirred at room temperature for additional 12 hours and quenched with a saturated aqueous solution of NH_4Cl. The organic layer was separated and the aqueous layer was extracted with EtOAc. Purification by column chromatography (PE/EA = 10/1) gave S**16** as a colorless oil (4.16 g, 90% yield).

Step III: The synthetic procedure to access compound **4l** is referred to the compound **4j**. Purification by column chromatography (PE/EA = 5/1) gave (*R*,*E*)-5-(*N*-(4-methoxybenzyl)-3-phenylpropiolamido)pent-3-en-2-yl acetate (**4l**) as a colorless oil (626 mg, 80% yield, 98% ee).

^{1}H NMR (600 MHz, $CDCl_3$): δ 7.54–7.50 (m, 2H), 7.43–7.38 (m, 1H), 7.38–7.32 (m, 2H), 7.24–7.18 (m, 2H), 6.90–6.84 (m, 2H), 5.68–5.60 (m, 1H), 5.60–5.53 (m, 1H), 5.37–5.29 (m, 1H), 4.81–4.71 (m, 1H), 4.61–4.53 (m, 1H), 4.16–4.10 (m, 1H), 4.01–3.89 (m, 1H), 3.80 (d, J = 5.3 Hz, 3H), 2.03 (d, J = 14.9 Hz, 3H), 1.28 (dd, J = 6.5, 3.7 Hz, 3H);

^{13}C NMR (151 MHz, $CDCl_3$): δ 170.2, 170.2, 159.3, 159.1, 154.7, 154.4, 133.6, 133.5, 132.4, 132.3, 130.1, 130.1, 129.8, 129.0, 128.5, 128.4, 128.0, 126.1, 125.4, 120.4, 120.3, 114.2, 114.0,

90.4, 90.4, 81.7, 81.5, 70.0, 69.8, 55.3, 55.2, 51.3, 49.2, 46.4, 44.6, 21.3, 21.2, 20.1, 20.1;

HRMS: (ESI) calcd for $C_{24}H_{26}NO_4^+$ ($[M+H]^+$): 392.1856; found: 392.1855;

HPLC conditions: OD-H column (15% ^{i}PrOH in hexane, 1.0 mL/min, λ = 254 nm, 30 °C), t_R (major) = 10.9 min, t_R (minor) = 12.0 min;

Optical Rotation: $[\alpha]^{25}_D$ = 7.7 (*c* = 0.33, $CHCl_3$) for 98% ee.

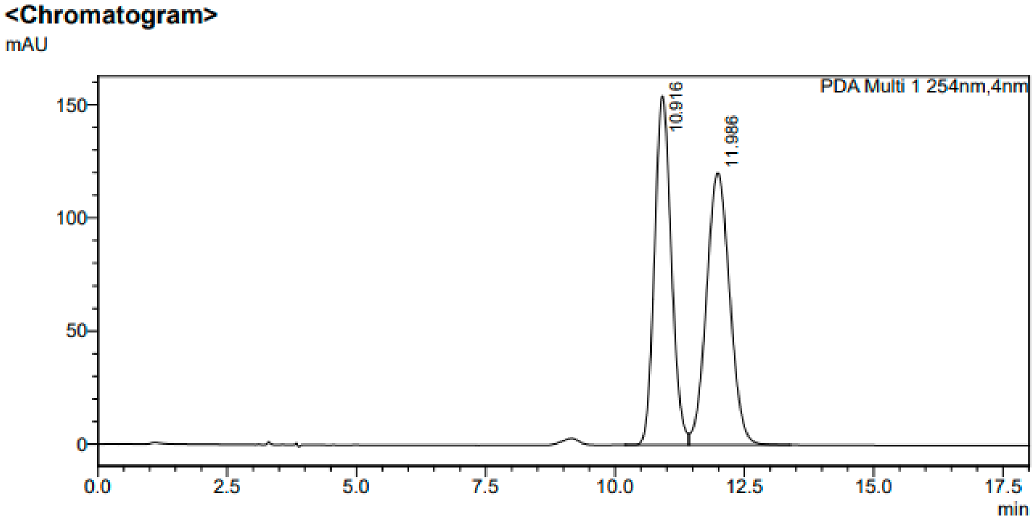

<Peak Table>

PDA Ch1 254nm

Peak#	Ret. Time	Area	Height	Conc.	Unit	Mark	Name
1	10.916	3496935	154374	0.000		M	
2	11.986	3766074	120274	0.000		V M	
Total		7263009	274648				

Supplementary Figure 27. HPLC spectrum of racemic-(*E*)-**4l**

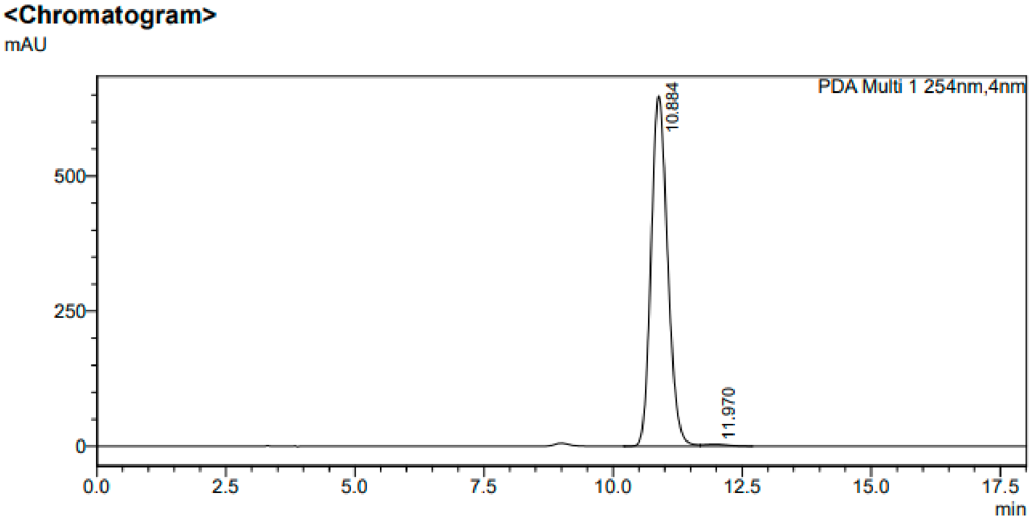

<Peak Table>

PDA Ch1 254nm

Peak#	Ret. Time	Area	Height	Conc.	Unit	Mark	Name
1	10.884	14478665	647931	0.000		M	
2	11.970	118496	3797	0.000		V M	
Total		14597161	651728				

Supplementary Figure 28. HPLC spectrum of (*R*,*E*)-**4l**

3.10 Synthesis of 1,6-enyne 1o

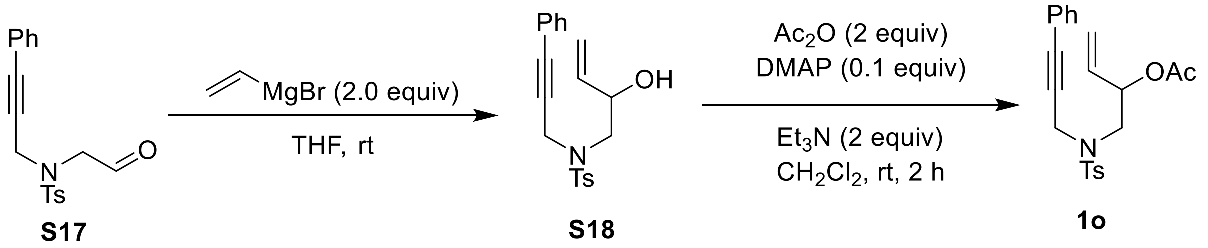

Step I: To a 250 mL round-bottom flask was added 4-methyl-*N*-(2-oxoethyl)-*N*-(3-phenylprop-2-yn-1-yl)benzenesulfonamide **S17** (2.616 g, 8 mmol).[6] The flask was degassed and refilled with N_2 for three times. Then vinyl magnesium bromide (1.0 M solution in THF, 16 mL) was added. The reaction mixture was stirred at room temperature for 12 hours and quenched with a saturated aqueous solution of NH_4Cl. The organic layer was separated and the aqueous layer was extracted with EtOAc. The combined organic layers were dried over Na_2SO_4, filtered and concentrated. Purification by flash column chromatography (PE/EA = 10/1) gave **S18** as a brown oil (0.76 g, 26% yield).

Step II: To a solution of the above allylic alcohol **S18** (676.7 mg, 2 mmol) in CH_2Cl_2 (10 mL) was added Et_3N (0.55 mL, 4 mmol) and DMAP (25.0 mg, 0.2 mmol) at 0 °C. Then acetic anhydride (0.38 mL, 4 mmol) was added dropwise at the same temperature. The reaction mixture was stirred at room temperature for 2 hours. The solvent was removed under reduced pressure. Purification by column chromatography (10% EtOAc in petroleum ether) gave 1-((4-methyl-*N*-(3-phenylprop-2-yn-1-yl)phenyl)sulfonamido)but-3-en-2-yl acetate (**1o**) as a yellow solid (644.7 mg, 81% yield).

^{1}H NMR (400 MHz, $CDCl_3$): δ7.75 (d, *J* = 8.4 Hz, 2H), 7.28–7.20 (m, 5H), 7.10–7.00 (m, 2H), 5.92–5.80 (m, 1 H), 5.58–5.50 (m, 1 H), 5.38 (d, *J* = 17.6 Hz, 1 H), 5.29 (d, *J* = 10.4 Hz, 1 H), 4.48 (d, *J* = 18.8 Hz, 1 H), 4.35 (d, *J* = 18.8 Hz, 1 H), 3.56 (dd, *J* = 14.4, 8 Hz, 1 H), 3.36 (dd, *J* = 14.4, 4 Hz, 1 H), 2.32 (s, 3H), 2.10 (s, 3H);

^{13}C NMR (100 MHz, $CDCl_3$): δ 169.8, 143.5, 135.5, 133.0, 131.2, 129.4, 128.3, 127.9, 127.5, 121.7, 118.4, 85.7, 81.3, 71.6, 49.0, 38.2, 21.2, 20.9;

IR (KBr): 3467, 2926, 1744, 1646, 1597, 1491, 1442, 1352, 1227, 1161, 1006, 915, 818, 758 cm^{-1};

HRMS (m/z) Calcd for ($C_{22}H_{23}NO_4SNa$) ($[M+Na]^+$):420.1240; found: 420.1247.

3.11 Synthesis of 1,6-enyne 13

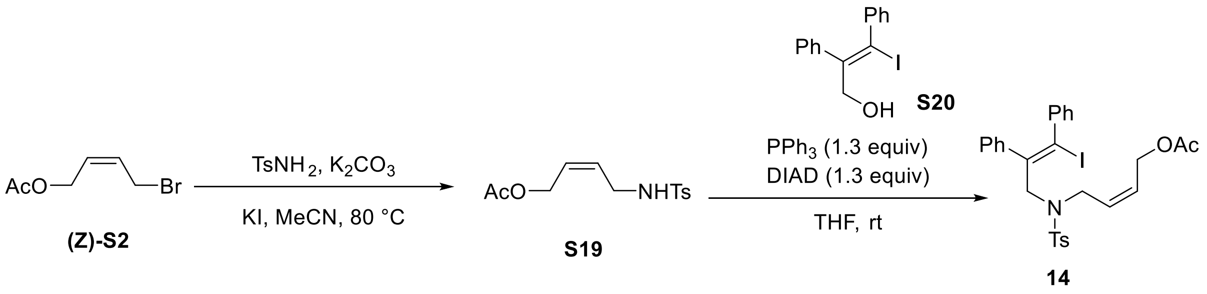

Step I: To a solution of $TsNH_2$ (6.0 mmol, 1.0 g), K_2CO_3 (6.0 mmol, 829.2 mg) and KI (0.3 mmol, 49.8 mg) in MeCN (8 mL) was added (*Z*)-**S2** (3.0 mmol, 579.0 mg). The reaction mixture was stirred under N_2 at 80 °C for 12 hours. The resulting mixture was filtered through a celite pad, and the filtrate was concentrated and purified by chromatography on silica gel, eluting with PE/EA = 5/1 to afford **S19** (594.9 mg, 70% yield).

Step II: To a solution of **S19** (2.0 mmol, 560.0 mg), **S20** (2.2 mmol, 739.6 mg)[7] and PPh_3 (2.6 mmol, 681.2 mg) in THF (5 mL) was added a solution of DIAD (2.6 mmol, 525.2 mg) in THF (2 mL) dropwise under N_2 at 0 °C. The reaction mixture was stirred at room temperature overnight. Then the resulting mixture was concentrated under vacuum and purified by flash column chromatography (PE/EA = 10/1) to give **14** (793.9 mg, 66% yield).

^{1}H NMR (600 MHz, $CDCl_3$): δ 7.49 (d, *J* = 8.3 Hz, 2H), 7.21 (d, *J* = 8.1 Hz, 2H), 7.13–7.05 (m, 5H), 7.05–7.00 (m, 3H), 6.98–6.93 (m, 2H), 5.68–5.61 (m, 1H), 5.49–5.42 (m, 1H), 4.66–4.58 (m, 4H), 3.72 (d, *J* = 6.5 Hz, 2H), 2.41 (s, 3H), 2.08 (s, 3H);

^{13}C NMR (151 MHz, $CDCl_3$): δ 170.8, 144.2, 143.4, 143.3, 136.7, 136.4, 129.6, 129.6, 129.3, 127.9, 127.7, 127.5, 127.5, 127.2, 126.9, 102.9, 60.0, 59.4, 44.6, 21.5, 21.0;

HRMS: (ESI) calcd for $C_{28}H_{29}INO_4S^+$ ($[M+H]^+$): 602.0857; found: 602.0853.

3.11 Synthesis of 1,6-enyne 14

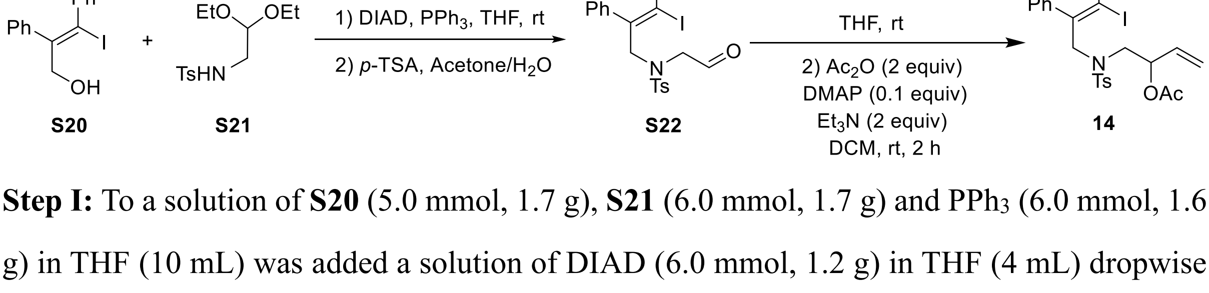

Step I: To a solution of **S20** (5.0 mmol, 1.7 g), **S21** (6.0 mmol, 1.7 g) and PPh_3 (6.0 mmol, 1.6 g) in THF (10 mL) was added a solution of DIAD (6.0 mmol, 1.2 g) in THF (4 mL) dropwise

under N_2 at 0 °C. The reaction mixture was stirred at room temperature overnight. Then the resulting mixture was concentrated under vacuum and purified by flash column chromatography (PE/EA = 10/1) to give crude product (2.4 g, 80% yield). Dissolved above crude product in Acetone (5 mL), *p*-TSA (68.9 mg, 0.4 mmol) and H_2O (14.4 mg, 0.8 mmol) was added. The mixture was stirred at room temperature for 12 hours and concentrated under vacuum to give **S22**, and without purification for next step.

Step II: The synthetic procedure to access compound **14** is referred to the compound **1o**. Purification by column chromatography (PE/EA = 5/1) gave **14** as a colorless oil (962 mg, 40% yield).

^{1}H NMR (600 MHz, $CDCl_3$) δ 7.54-7.47 (m, 2H), 7.22-7.16 (m, 2H), 7.10-7.03 (m, 3H), 7.03 -6.95 (m, 5H), 6.87-6.82 (m, 2H), 5.72-5.63 (m, 1H), 5.64-5.56 (m, 1H), 5.31-5.24 (m, 1H), 5.23-5.15 (m, 1H), 4.78-4.67 (m, 2H), 3.34 (dd, *J* = 15.0, 8.5 Hz, 1H), 3.11 (dd, *J* = 14.9, 4.9 Hz, 1H), 2.40 (s, 3H), 1.99 (s, 3H).

4. Optimization Details

4.1 Supplementary Table 1. Optimization of the enantioselective reductive alkylative cyclization

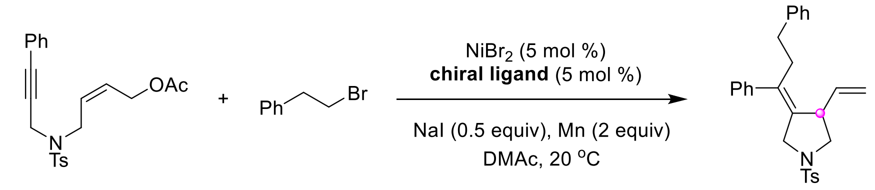

1a **5a** **6aa**

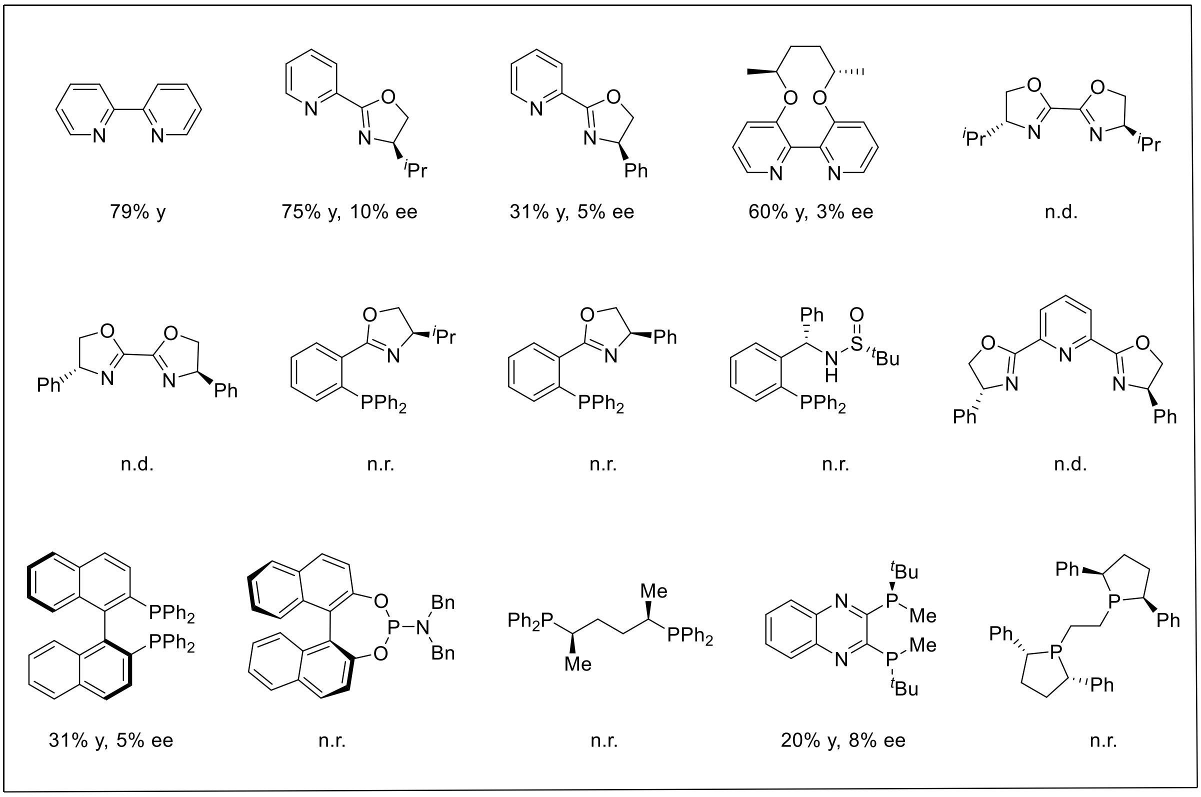

4.2 Supplementary Table 2. Screening of catalyst in the enantiospecific reductive alkylative cyclization

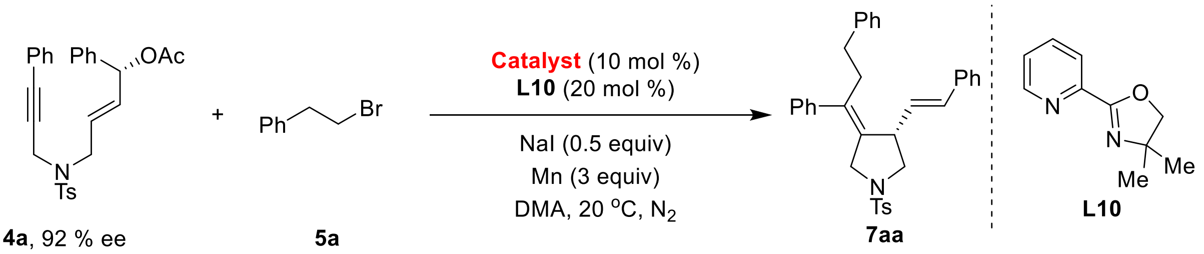

4a, 92 % ee　**5a**　**7aa**　**L10**

Entry	[Ni]	Yield of **7aa** (%)[a]	ee of **7aa** (%)[b]
1	$NiBr_2$	60	74
2	NiI_2	68	79
3	$NiCl_2$	58	74
4	$NiBr_2$·dme	64	77
5	$NiCl_2$·dme	58	70
6	$Ni(ClO_4)_2·6H_2O$	69	80
7	$Ni(cod)_2$	62	64
8	$Ni(acac)_2$	N.R	-
9	$Ni(dppe)Cl_2$	66	74
10	$Ni(PCy_3)_2Cl_2$	52	53
11	$Ni(OAc)_2·4H_2O$	60	79
12	$Ni(OTf)_2$	N.R	-
13	$CoBr_2$	31	68

Reaction conditions: **4a** (0.1 mmol, 92% ee), **5a** (0.2 mmol), catalyst (10 mol %), **L10** (20 mol %), Mn dust (0.3 mmol), NaI (0.05 mmol) in sealed tube in 2 mL DMA at 20 °C, unless noted otherwise. [a]Isolated yields. [b]The ee values were determined by HPLC analysis with a chiral column.

4.3 Supplementary Table 3. Screening of additive and reductant in the enantiospecific reductive alkylative cyclization

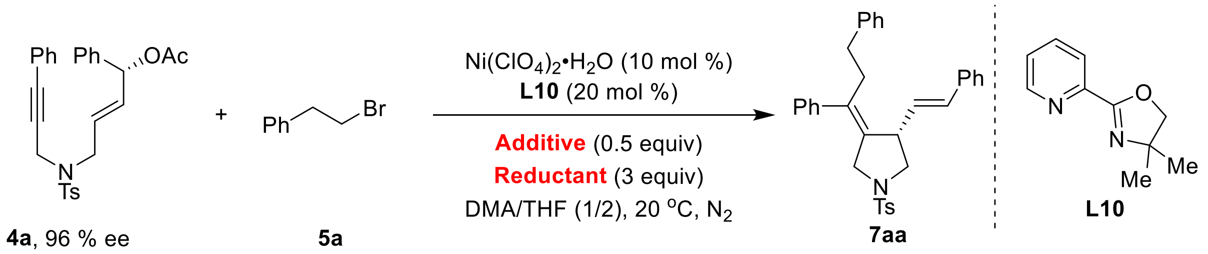

Entry	Additive	Reductant	Yield of **7aa** (%)[a]	ee of **7aa** (%)
1	NaI	Mn	73	87
2[c]	NaI	Mn	72	86
3	NaI	Zn	36	84
4	TBAI	Mn	61	86
5	TBAB	Mn	23	88
6	$MgCl_2$	Mn	trace	-
7	$MnBr_2$	Mn	44	83

Reaction conditions: **4a** (0.1 mmol, 96% ee), **5a** (0.2 mmol), $Ni(ClO_4)_2$•H_2O (10 mol %), **L10** (20 mol %), Mn dust (0.3 mmol), Additive (0.05 mmol) in sealed tube in 2 mL DMA/THF (1/2) at 20 °C, unless noted otherwise. [a]Isolated yields; [b]The ee values were determined by HPLC analysis with a chiral column. [c]1.0 equiv of NaI was used.

5. Exploring the Effect of Leaving Groups

5.1 Effect of other leaving group in enantioselective arylative cyclization.

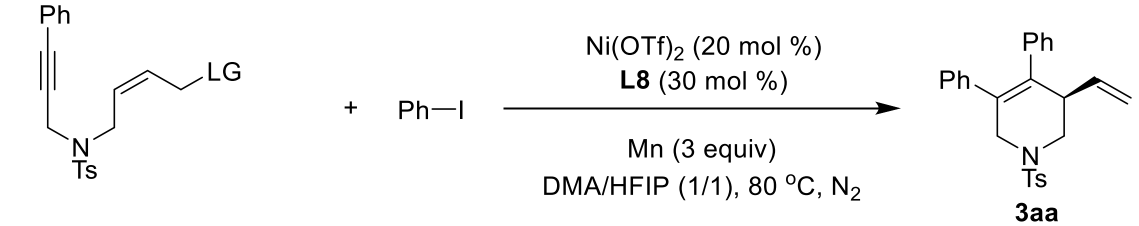

3aa

1a', LG = $OPO(OEt)_2$
1a", LG = OCO_2Me

2a

from **1a'**, 38%, 92% ee
from **1a"**, 42%, 95% ee

5.2 Effect of other leaving group in enantiospecific alkylative cyclization.

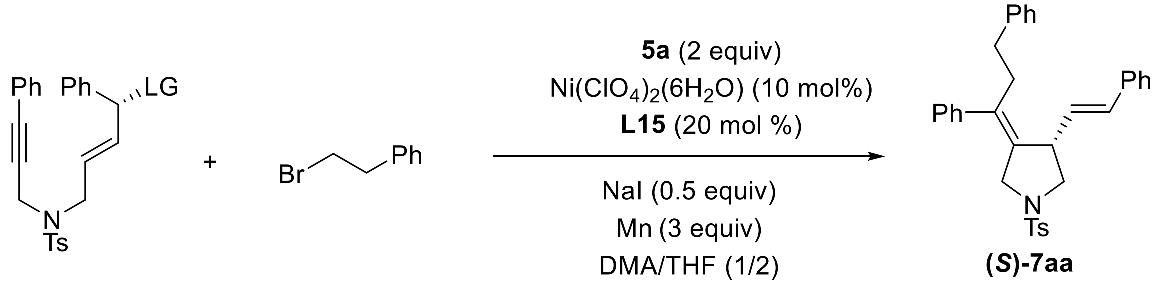

(*S*)-7aa

4a', LG = OPiv, 99% ee
4a", LG = OBoc, 99% ee

from **4a'**, 52%, 89% ee
from **4a"**, 60%, 86% ee

6. Exploring the Effect of NaI

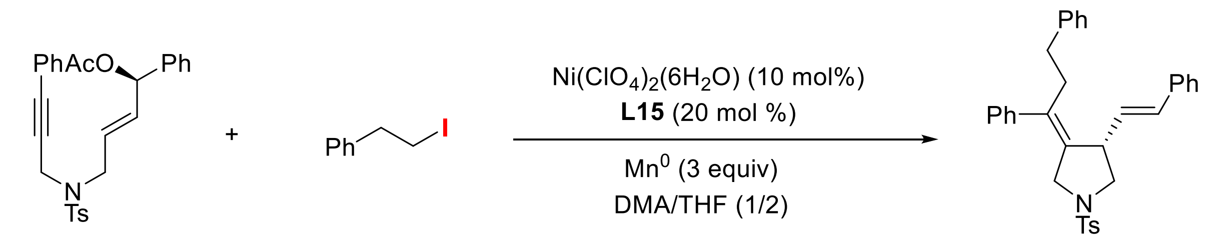

(*R*)-4a (99% ee)

additive	**(*S*)-7aa**
NaI (0.5 equiv)	75%, 90% ee
---	55%, 93% ee

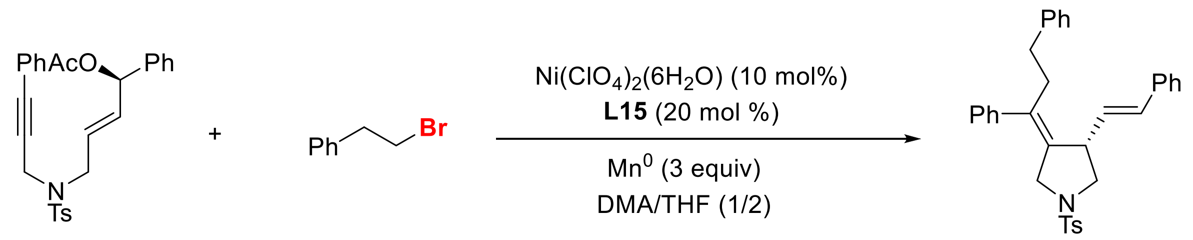

(*R*)-4a (99% ee)

additive	**(*S*)-7aa**
NaI (0.5 equiv)	78%, 91% ee
---	21%, 91% ee

To test the possibility of halide exchange between alkyl bromides and NaI, we used alkyl iodide as an alkylation source in the presence or absence of NaI. The product **7aa** could be obtained in 72% and 55% yield, respectively. By comparing these results of alkyl bromide with alkyl iodide, we cannot rule out the possibility of halide exchange between alkyl bromides with NaI.

7. Exploring the Effect of Alkene Geometry

(*R*,*Z*)-3-(1,3-diphenylpropylidene)-4-((*E*)-styryl)-1-tosylpyrrolidine ((*R*)-7aa)

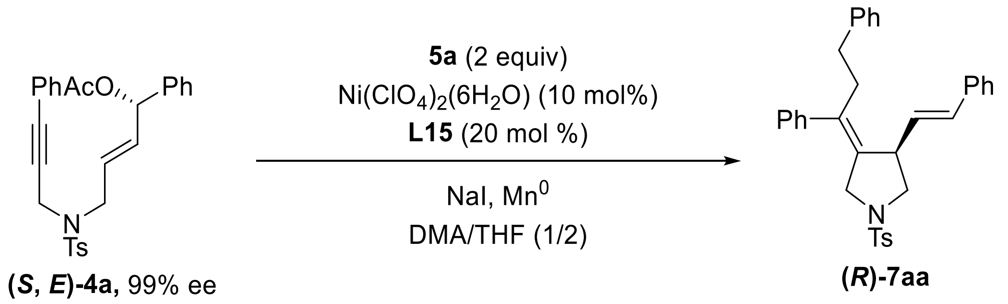

(*R*)-**7aa** was prepared according to general procedure **2.2** from (*S*,*E*)-**4a** (0.1 mmol, 47.4 mg) and (2-bromoethyl)benzene **5a** (0.2 mmol, 37.0 mg). Purification by silica gel column chromatography (PE/EA = 10/1) gave (*R*)-**7aa** as a white solid (41.1 mg, 79% yield, 95% ee). HPLC conditions: AD-H column (15% ^{i}PrOH in hexane, 1.0 mL/min, λ = 254 nm, 30 °C), t_R (major) = 10.2 min, t_R (minor) = 11.0 min;

Optical Rotation: $[\alpha]^{25}_D$ = 3.3 (*c* = 0.23, $CHCl_3$) for 95% ee.

<Chromatogram>

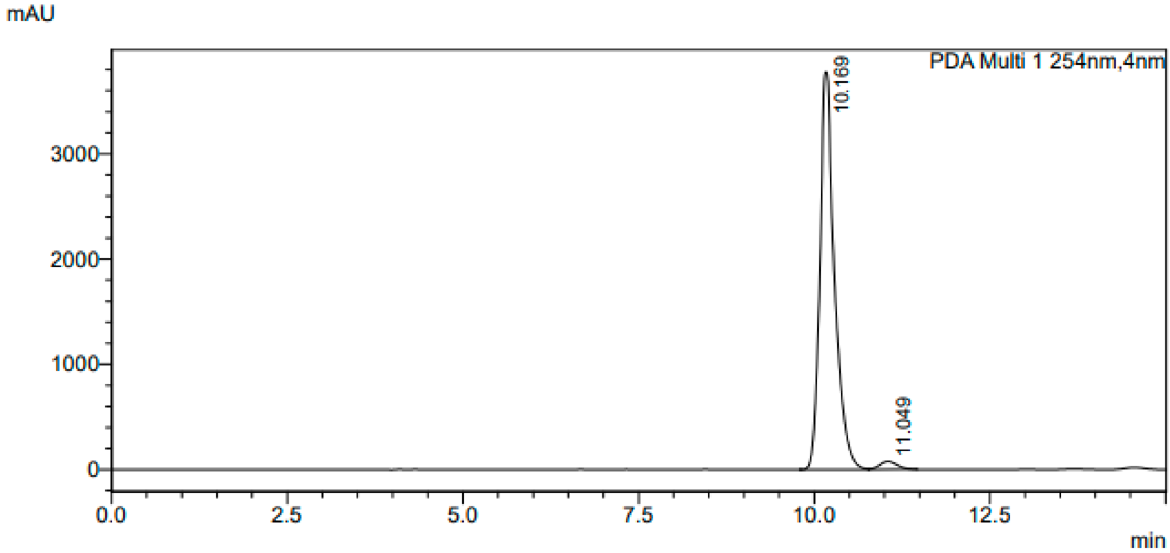

<Peak Table>

PDA Ch1 254nm

Peak#	Ret. Time	Area	Height	Conc.	Unit	Mark	Name
1	10.169	54617708	3776908	0.000		M	
2	11.049	1328039	77558	0.000		V M	
Total		55945746	3854465				

Supplementary Figure 29. HPLC spectrum of (*R*)-**7aa** prepared from (*S*,*E*)-**4a**

(*S*,*Z*)-3-(1,3-diphenylpropylidene)-4-((*E*)-styryl)-1-tosylpyrrolidine ((*S*)-7aa)

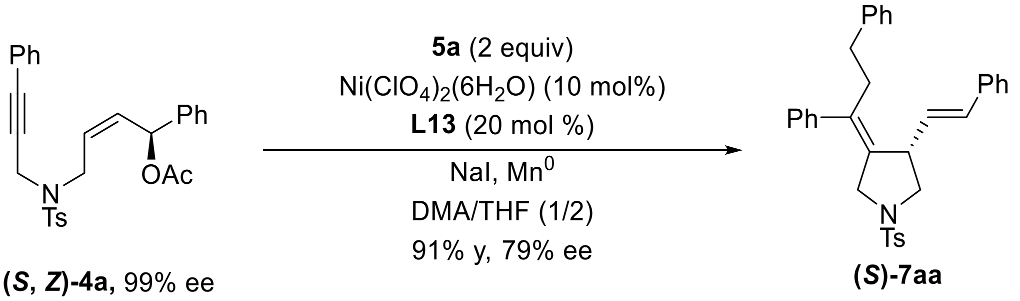

(*S*, *Z*)-4a, 99% ee **(*S*)-7aa**

(*S*)-**7aa** was prepared according to general procedure **2.2** using **L13** (0.02 mmol, 3.0 mg) from (*S*,*Z*)-**4a** (0.1 mmol, 47.4 mg) and (2-bromoethyl)benzene **5a** (0.2 mmol, 37.0 mg). Purification by silica gel column chromatography (PE/EA = 10/1)gave (*S*)-**7aa** as a white solid (47.2 mg, 91% yield, 79% ee).

HPLC conditions: AD-H column (15% ^{i}PrOH in hexane, 1.0 mL/min, λ = 254 nm, 30 °C), t_R (minor) = 10.3 min, t_R (major) = 11.4 min.

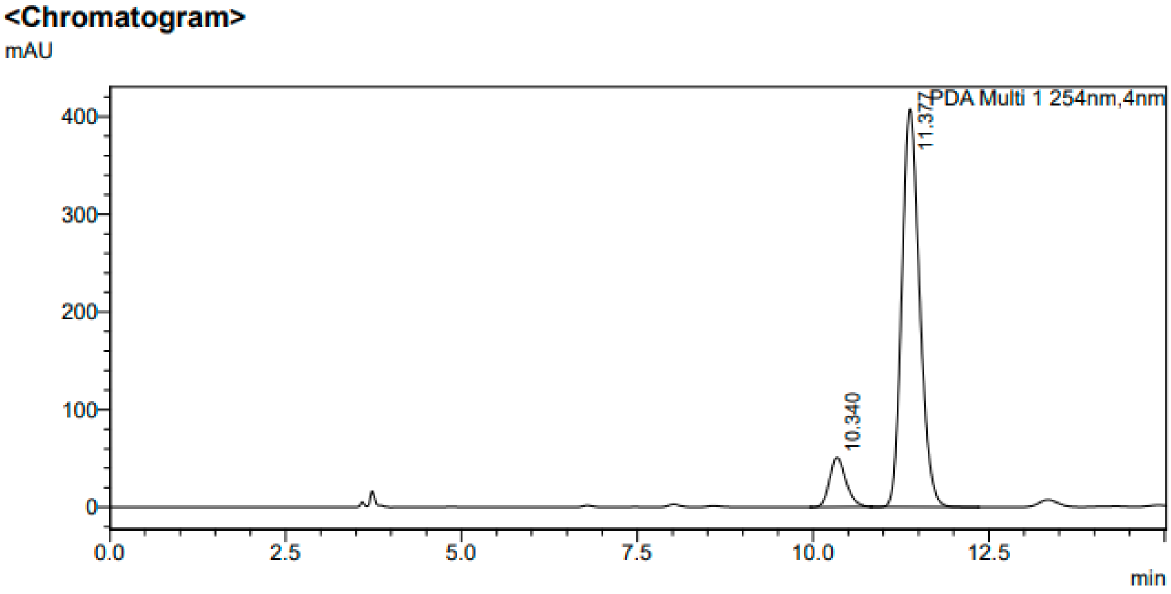

<Peak Table>

PDA Ch1 254nm

Peak#	Ret. Time	Area	Height	Conc.	Unit	Mark	Name
1	10.340	831660	50752	0.000		M	
2	11.377	7183261	407473	0.000		M	
Total		8014921	458225				

Supplementary Figure 30. HPLC spectrum of (*S*)-**7aa** prepared from (*S*,*Z*)-**4a**

(*R*,*Z*)-3-(1,3-diphenylpropylidene)-4-((*E*)-styryl)-1-tosylpyrrolidine ((*R*)-7aa)

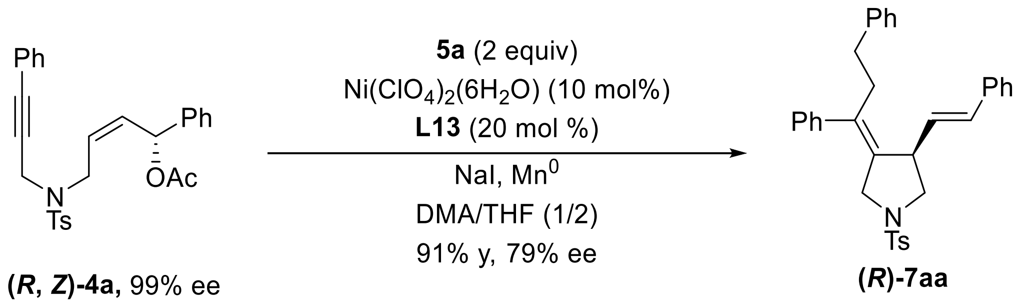

(*R*)-**7aa** was prepared according to general procedure **2.2** using **L13** (0.02 mmol, 3.0 mg) from (*R*,*Z*)-**4a** (0.1 mmol, 47.4 mg) and (2-bromoethyl)benzene **5a** (0.2 mmol, 37.0 mg). Purification by silica gel column chromatography (PE/EA = 10/1) to give (*S*)-**7aa** as a white solid (48.9 mg, 94% yield, 83% ee).

HPLC conditions: AD-H column (15% ^{i}PrOH in hexane, 1.0 mL/min, λ = 254 nm, 30 °C), t_R (major) = 10.4 min, t_R (minor) = 11.3 min.

<Chromatogram>

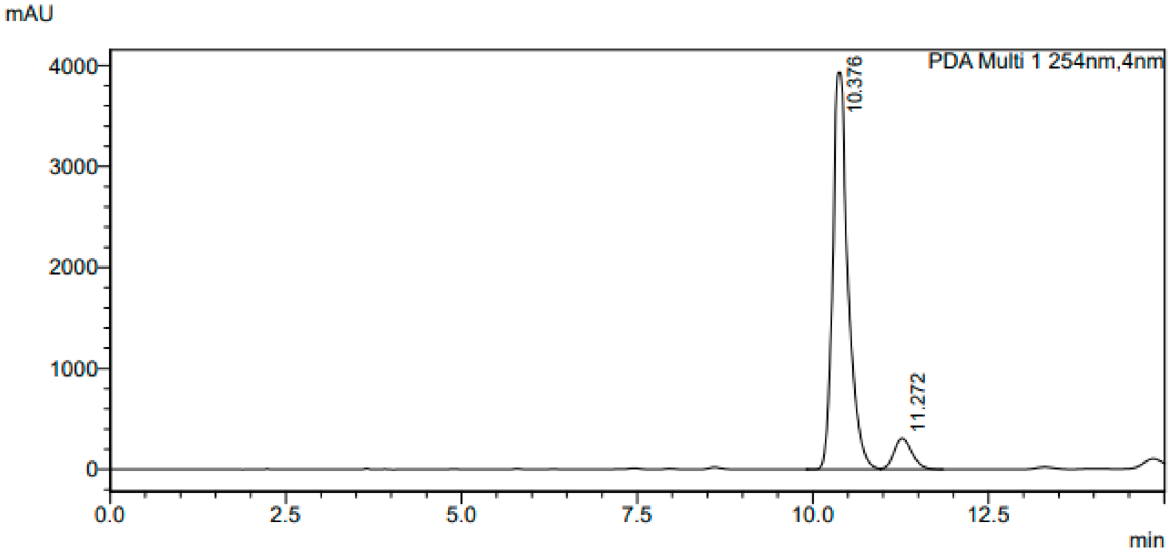

<Peak Table>

PDA Ch1 254nm

Peak#	Ret. Time	Area	Height	Conc.	Unit	Mark	Name
1	10.376	59644521	3934356	0.000		M	
2	11.272	5505610	308232	0.000		V M	
Total		65150130	4242588				

Supplementary Figure 31. HPLC spectrum of (*R*)-**7aa** prepared from (*R*,*Z*)-**4a**

8. Characterization Data of Products

4,5-diphenyl-1-tosyl-3-vinyl-1,2,3,6-tetrahydropyridine (3aa)

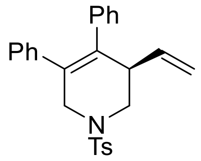

Chemical Formula: $C_{26}H_{25}NO_2S$
Exact Mass: 415.1606

3aa was prepared according to general procedure **2.1** using (*Z*)-4-((4-methyl-*N*-(3-phenylprop-2-yn-1-yl)phenyl)sulfonamido)but-2-en-1-yl acetate **1a** (0.4 mmol, 79.5 mg) and iodobenzene **2a** (0.4 mmol, 81.6 mg). Purification by silica gel column chromatography (PE/EA = 20/1) gave **3aa** as a white solid (61.7 mg, 74% yield).

^{1}H NMR (400 MHz, $CDCl_3$): δ 7.71 (d, *J* = 8.0 Hz, 2H), 7.33 (d, *J* = 8.0 Hz, 2H), 7.16–7.02 (m, 6H), 7.01–6.93 (m, 2H), 6.92–6.82 (m, 2H), 5.85 (ddd, *J* = 17.6, 10.0, 7.6 Hz, 1H), 4.99 (d, *J* = 10.0 Hz, 1H), 4.96 (d, *J* = 17.6 Hz, 1H), 4.35 (d, *J* = 16.0 Hz, 1H), 3.74 (dd, *J* = 11.2, 3.2 Hz, 1H), 3.47–3.33 (m, 2H), 3.02 (dd, *J* = 11.2, 4.0 Hz, 1H), 2.43 (s, 3H);

^{13}C NMR (100 MHz, $CDCl_3$): δ 143.6, 139.8, 138.9, 136.8, 135.6, 132.7, 131.7, 129.7, 129.4, 129.3, 127.9, 127.7, 127.6, 127.0, 126.4, 116.9, 49.3, 47.9, 44.7, 21.5;

IR (KBr): 3439, 1597, 1492, 1342, 1163, 1092, 995, 815, 772, 699, 549 cm^{-1};

HRMS-ESI (m/z) Calcd for ($C_{26}H_{25}NO_2SNa$) ([M+Na]$^+$): 438.1498; found: 438.1504;

HPLC conditions: OD-H column (5% ^{i}PrOH in hexane, 1.0 mL/min, λ = 254 nm, 35 °C), t_R (major) = 8.6 min, t_R (minor) = 9.3 min;

Optical Rotation: $[\alpha]^{25}_D$ = -267.6 (*c* = 0.85, $CHCl_3$) for 95% ee.

Absolute stereochemistry was determined through analogy with **3aj**.

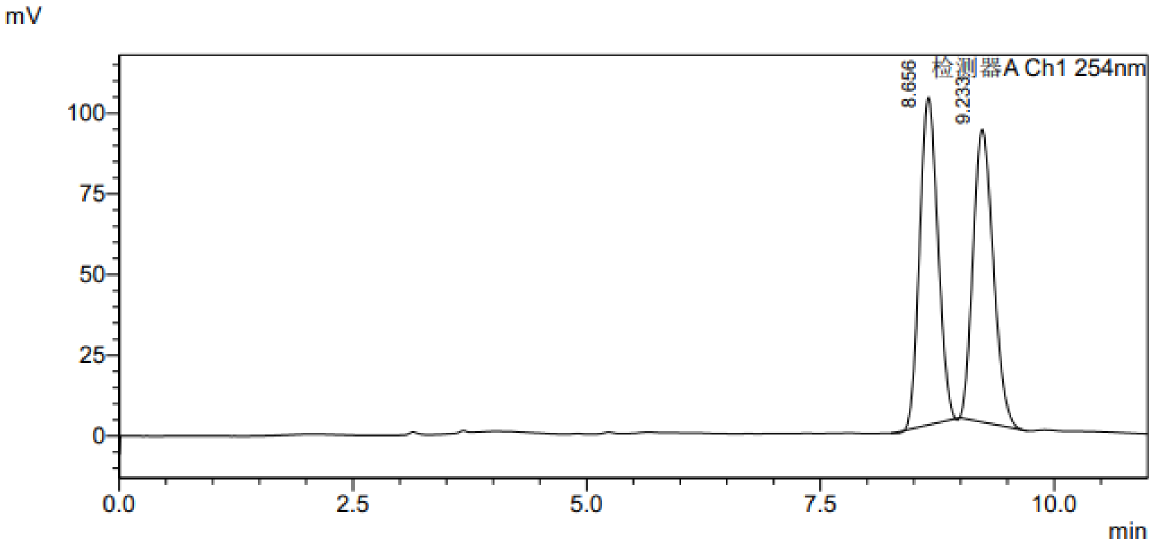

Peak#	Ret. Time	Area	Height	Conc.	Unit	Mark	Name
1	8.656	1376970	101445	50.129		M	
2	9.233	1369898	90625	49.871		M	
总计		2746867	192070	100.000			

Supplementary Figure 32. HPLC spectrum of racemic-**3aa**

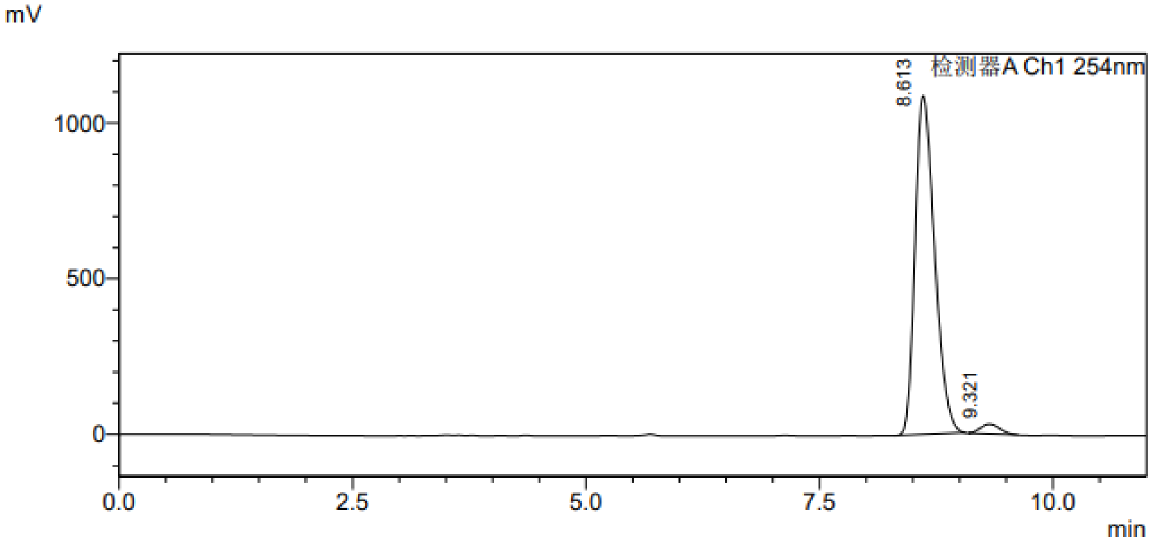

Peak#	Ret. Time	Area	Height	Conc.	Unit	Mark	Name
1	8.613	15880841	1087285	97.268		M	
2	9.321	446079	30760	2.732		M	
总计		16326920	1118045	100.000			

Supplementary Figure 33. HPLC spectrum of (*R*)-**3aa**

4-phenyl-5-(*p*-tolyl)-1-tosyl-3-vinyl-1,2,3,6-tetrahydropyridine (3ab)

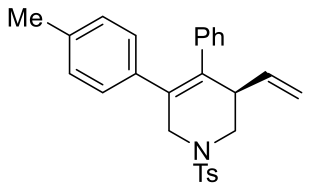

Chemical Formula: $C_{27}H_{27}NO_2S$
Exact Mass: 429.1762

3ab was prepared according to general procedure **2.1** using (*Z*)-4-((4-methyl-*N*-(3-phenylprop-2-yn-1-yl)phenyl)sulfonamido)but-2-en-1-yl acetate **1a** (0.2 mmol, 79.5 mg) and 1-iodo-4-methylbenzene **2b** (0.4 mmol, 87.2 mg). Purification by silica gel column chromatography (PE/EA = 20/1) gave **3ab** as a white solid (68.4 mg, 80% yield).

^{1}H NMR (400 MHz, $CDCl_3$): δ 7.70 (d, *J* = 8.4 Hz, 2H), 7.33 (d, *J* = 8.0 Hz, 2H), 7.13–7.02 (m, 3H), 6.97–6.82 (m, 6H), 5.85 (ddd, *J* = 17.6, 10.0, 8.0 Hz, 1H), 4.99 (d, *J* = 10.0 Hz, 1H), 4.95 (d, *J* = 17.6 Hz, 1H), 4.35 (d, *J* = 16.0 Hz, 1H), 3.75 (dd, *J* = 11.2, 2.8 Hz, 1H), 3.47–3.30 (m, 2H), 3.00 (dd, *J* = 11.2, 3.6 Hz, 1H), 2.43 (s, 3H), 2.23 (s, 3H);

^{13}C NMR (100 MHz, $CDCl_3$): δ 143.6, 140.0, 136.8, 136.7, 135.8, 135.2, 132.6, 131.5, 129.6, 129.4, 129.1, 128.6, 127.7, 127.6, 126.3, 116.8, 49.3, 47.9, 44.7, 21.5, 21.0;

IR (KBr): 3473, 2920, 1597, 1461, 1343, 1165, 998, 812, 700, 673, 548 cm^{-1};

HRMS-ESI (m/z) Calcd for ($C_{27}H_{27}NO_2SNa$) ($[M+Na]^+$): 452.1655; found: 452.1660;

HPLC conditions: AD-H column (2% ^{i}PrOH in hexane, 1.0 mL/min, λ = 254 nm, 35 °C), t_R (major) = 23.1 min, t_R (minor) = 26.5 min;

Optical Rotation: $[\alpha]^{25}_D$ = -308.3 (*c* = 2.00, $CHCl_3$) for 93% ee.

Absolute stereochemistry was determined through analogy with **3aj**.

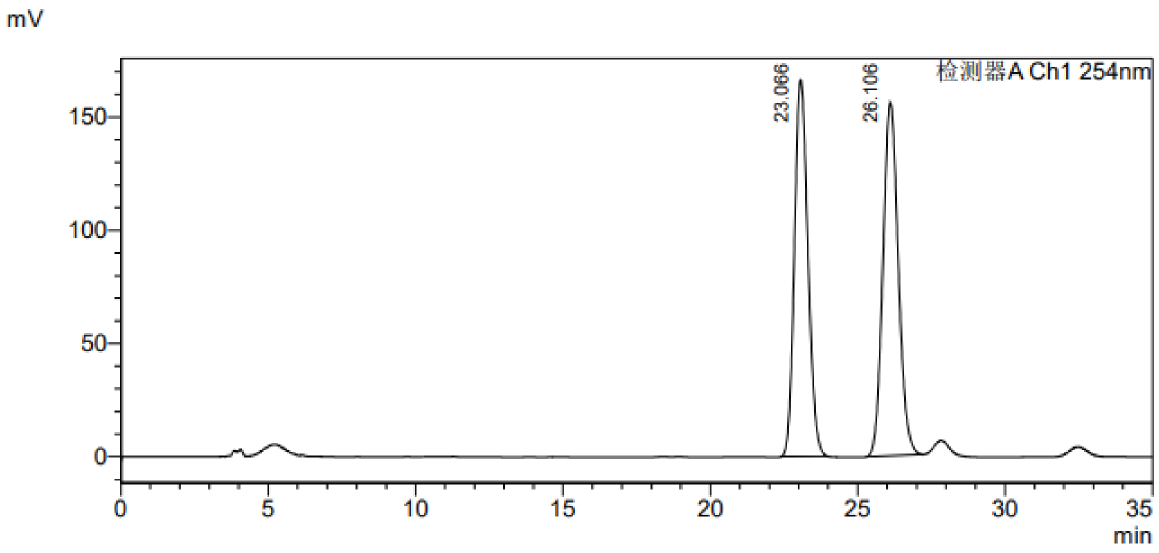

Peak#	Ret. Time	Area	Height	Conc.	Unit	Mark	Name
1	23.066	5292295	166189	48.360		M	
2	26.106	5651317	155797	51.640		M	
总计		10943612	321986	100.000			

Supplementary Figure 34. HPLC spectrum of racemic-**3ab**

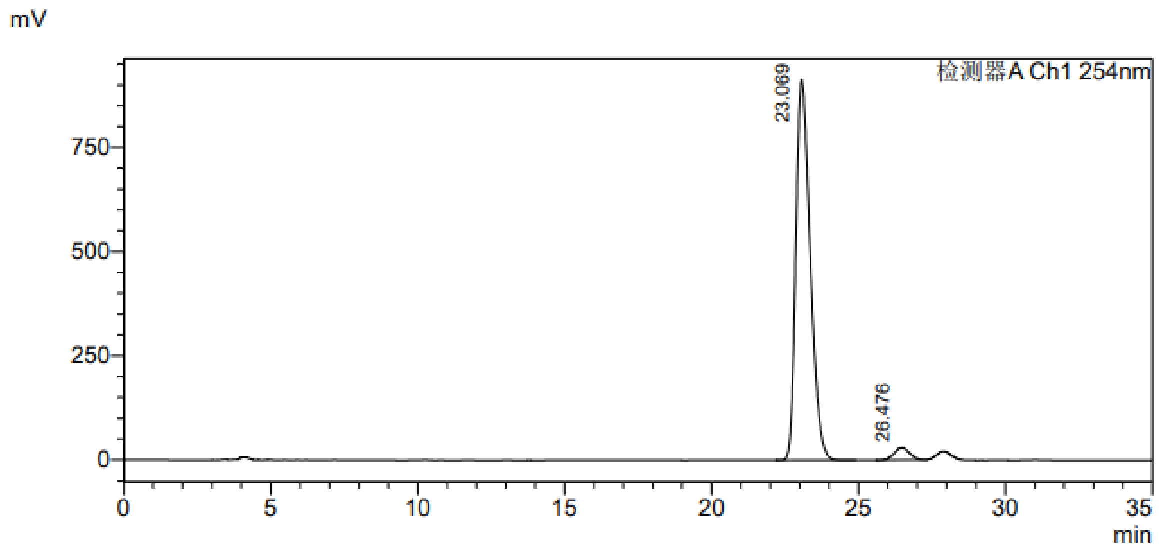

Peak#	Ret. Time	Area	Height	Conc.	Unit	Mark	Name
1	23.069	30975113	913108	96.706		M	
2	26.476	1054997	29089	3.294		M	
总计		32030110	942198	100.000			

Supplementary Figure 35. HPLC spectrum of (*R*)-**3ab**

5-(4-methoxyphenyl)-4-phenyl-1-tosyl-3-vinyl-1,2,3,6-tetrahydropyridine (3ac)

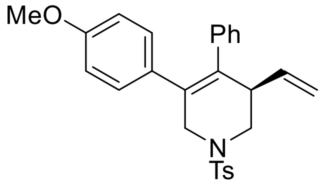

Chemical Formula: $C_{27}H_{27}NO_3S$
Exact Mass: 445.1712

3ac was prepared according to general procedure **2.1** using (*Z*)-4-((4-methyl-*N*-(3-phenylprop-2-yn-1-yl)phenyl)sulfonamido)but-2-en-1-yl acetate **1a** (0.2 mmol, 79.5 mg) and 1-iodo-4-methoxybenzene **2c** (0.4 mmol, 93.6 mg). Purification by silica gel column chromatography (PE/EA = 20/1) gave **3ac** as a white solid (48.8 mg, 55% yield).

^{1}H NMR (400 MHz, $CDCl_3$): δ 7.70 (d, *J* = 8.4 Hz, 2H), 7.33 (d, *J* = 8.0 Hz, 2H), 7.14–7.01 (m, 3H), 6.95–6.85 (m, 4H), 6.65 (d, *J* = 8.8 Hz, 2H), 5.84 (ddd, *J* = 17.6, 10.0, 8.0 Hz, 1H), 4.98 (d, *J* = 10.0 Hz, 1H), 4.94 (d, *J* = 17.6 Hz, 1H), 4.34 (d, *J* = 16.0 Hz, 1H), 3.75 (dd, *J* = 11.6, 2.4 Hz, 1H), 3.71 (s, 3H), 3.45–3.30 (m, 2H), 2.98 (dd, *J* = 11.6, 4.0 Hz, 1H), 2.42 (s, 3H);

^{13}C NMR (100 MHz, $CDCl_3$): δ 158.3, 143.6, 140.1, 136.9, 135.0, 132.7, 131.0, 130.4, 129.6, 129.4, 127.8, 127.7, 126.3, 116.7, 113.3, 55.0, 49.3, 47.9, 44.7, 21.5;

IR (KBr): 3418, 2967, 1606, 1513, 1341, 1163, 1032, 838, 705, 548 cm^{-1};

HRMS-ESI (m/z) Calcd for ($C_{27}H_{27}NO_3SNa$) ($[M+Na]^+$): 468.1604; found: 468.1610;

HPLC conditions: OD-H column (5% ^{i}PrOH in hexane, 1.0 mL/min, λ = 254 nm, 35 °C), t_R (major) = 11.2 min, t_R (minor) = 13.2 min;

Optical Rotation: $[\alpha]^{25}_D$ = -290.3 (*c* = 2.00, $CHCl_3$) for 92% ee.

Absolute stereochemistry was determined through analogy with **3aj**.

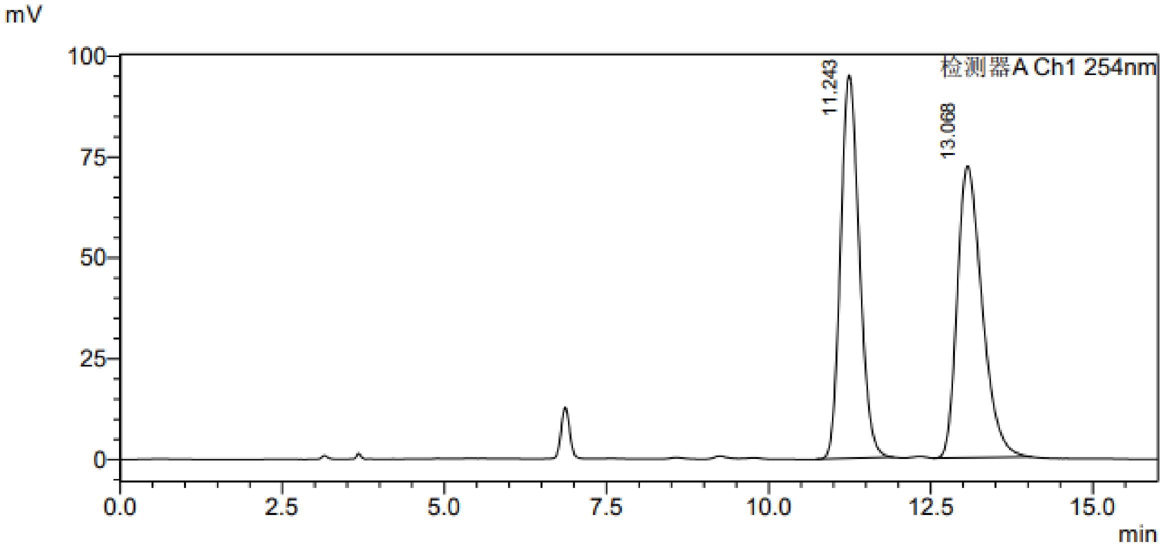

Peak#	Ret. Time	Area	Height	Conc.	Unit	Mark	Name
1	11.243	1916863	94873	49.731		M	
2	13.068	1937584	72275	50.269		M	
总计		3854446	167148	100.000			

Supplementary Figure 36. HPLC spectrum of racemic-**3ac**

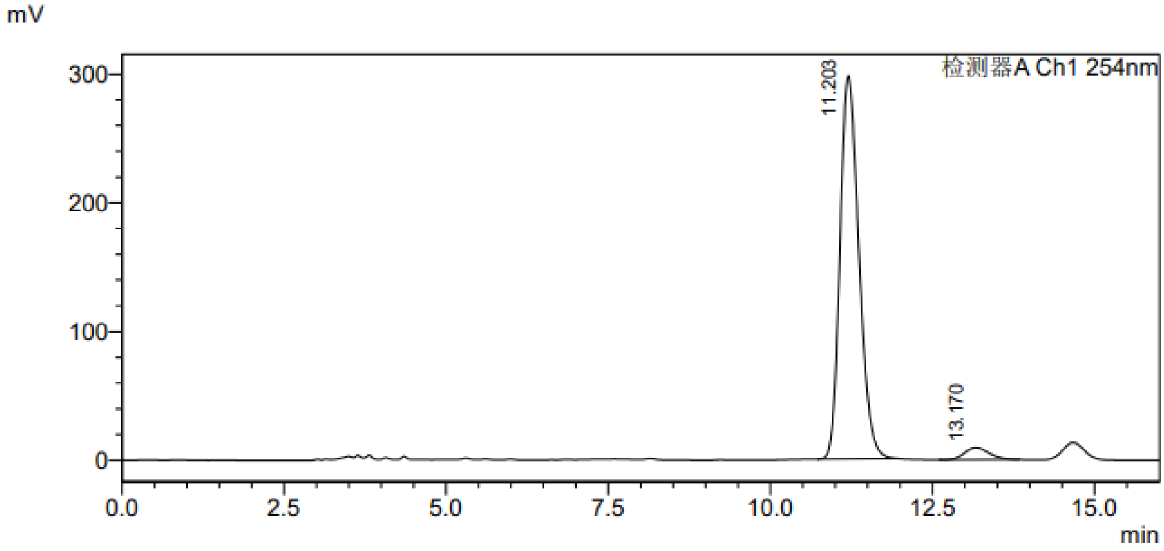

Peak#	Ret. Time	Area	Height	Conc.	Unit	Mark	Name
1	11.203	6046268	297645	96.073		M	
2	13.170	247130	9358	3.927		M	
总计		6293399	307003	100.000			

Supplementary Figure 37. HPLC spectrum of (*R*)-**3ac**

5-(4-cyanophenyl)-4-phenyl-1-tosyl-3-vinyl-1,2,3,6-tetrahydropyridine (3ad)

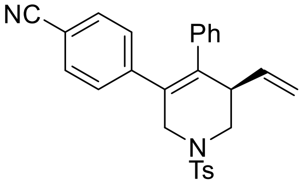

Chemical Formula: $C_{27}H_{24}N_2O_2S$
Exact Mass: 440.1558

3ad was prepared according to general procedure **2.1** using (*Z*)-4-((4-methyl-*N*-(3-phenylprop-2-yn-1-yl)phenyl)sulfonamido)but-2-en-1-yl acetate **1a** (0.2 mmol, 79.5 mg) and 4-iodobenzonitrile **2d** (0.4 mmol, 91.6 mg). Purification by silica gel column chromatography (PE/EA = 10/1) gave **3ad** as a white solid (64.4 mg, 73% yield).

^{1}H NMR (400 MHz, $CDCl_3$): δ 7.70 (d, *J* = 8.0 Hz, 2H), 7.45–7.30 (m, 4H), 7.16–7.01 (m, 5H), 6.90–6.76 (m, 2H), 5.80 (ddd, *J* = 17.2, 10.4, 7.7 Hz, 1H), 5.00 (d, *J* = 10.4 Hz, 1H), 4.95 (d, *J* = 17.2 Hz, 1H), 4.30 (d, *J* = 15.6 Hz, 1H), 3.71 (dd, *J* = 11.4, 3.1 Hz, 1H), 3.53–3.28 (m, 2H), 3.06 (dd, *J* = 11.4, 4.0 Hz, 1H), 2.43 (s, 3H);

^{13}C NMR (100 MHz, $CDCl_3$): δ 144.0, 143.9, 138.9, 138.1, 136.2, 132.6, 131.7, 130.2, 130.0, 129.8, 129.1, 127.9, 127.7, 127.1, 118.5, 117.4, 110.7, 48.6, 47.8, 44.8, 21.5;

IR (KBr): 3429, 2924, 2227, 1736, 1604, 1343, 1167, 932, 840, 706, 655, 550 cm^{-1};

HRMS-ESI (m/z) Calcd for ($C_{27}H_{24}N_2O_2SNa$) ($[M+Na]^+$): 463.1451; found: 463.1458;

HPLC conditions: AD-H column (10% ^{i}PrOH in hexane, 1.0 mL/min, λ = 254 nm, 35 °C), t_R (major) = 25.0 min, t_R (minor) = 28.1 min;

Optical Rotation: $[\alpha]^{25}_D$ = -138.4 (*c* = 2.80, $CHCl_3$) for 93% ee.

Absolute stereochemistry was determined through analogy with **3aj**.

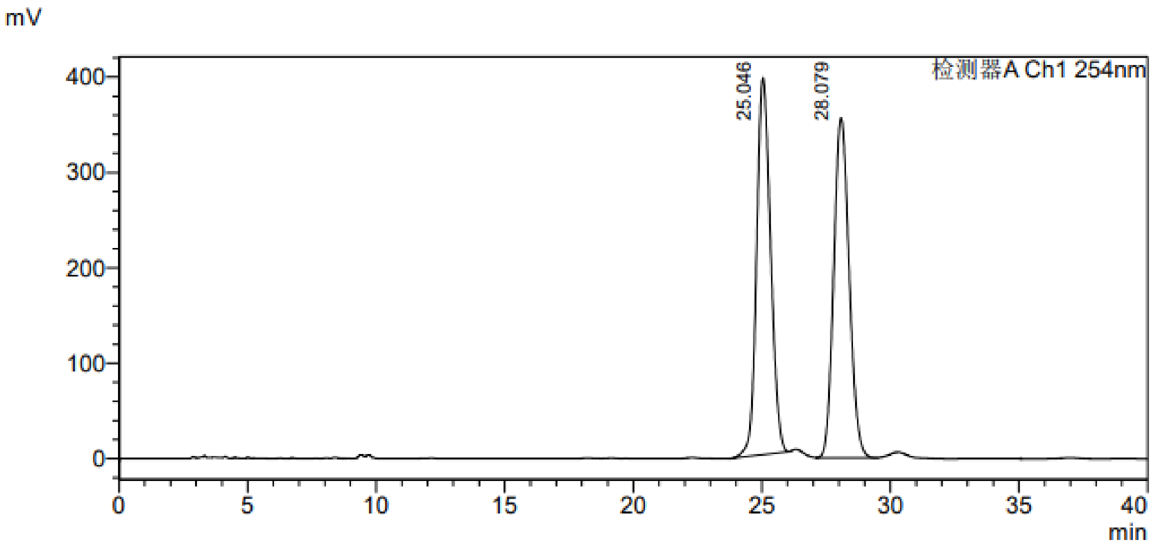

Peak#	Ret. Time	Area	Height	Conc.	Unit	Mark	Name
1	25.046	14881876	394804	50.045		M	
2	28.079	14855197	356047	49.955		M	
总计		29737073	750851	100.000			

Supplementary Figure 38. HPLC spectrum of racemic-**3ad**

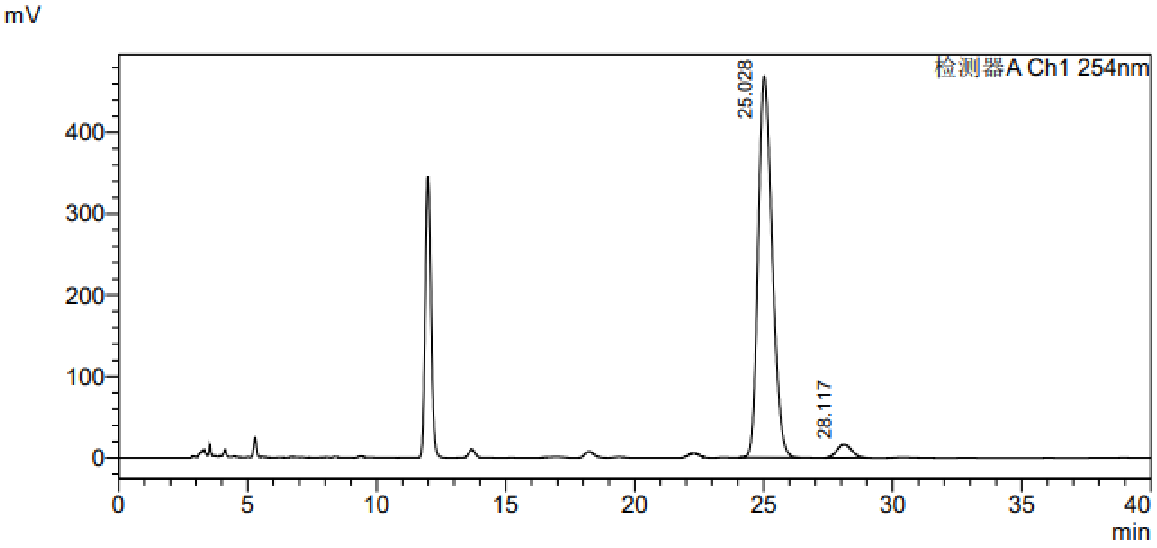

Peak#	Ret. Time	Area	Height	Conc.	Unit	Mark	Name
1	25.028	17713204	469186	96.414		M	
2	28.117	658911	16574	3.586		M	
总计		18372115	485759	100.000			

Supplementary Figure 39. HPLC spectrum of (*R*)-**3ad**

(*R*)-4-phenyl-5-(p-tolyl)-1-tosyl-3-vinyl-1,2,3,6-tetrahydropyridine--carbon dioxide (1/1) (3ae)

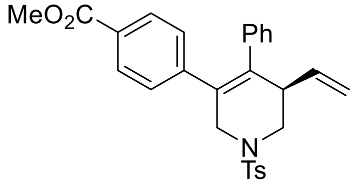

Chemical Formula: $C_{28}H_{27}NO_4S$
Exact Mass: 473.1661

3ae was prepared according to general procedure **2.1** using (*Z*)-4-((4-methyl-*N*-(3-phenylprop-2-yn-1-yl)phenyl)sulfonamido)but-2-en-1-yl acetate **1a** (0.2 mmol, 79.5 mg) and methyl 4-iodobenzoate **2e** (0.4 mmol, 104.8 mg). Purification by silica gel column chromatography (PE/EA = 10/1) gave **3ae** as a white solid (58.8 mg, 62% yield).

^{1}H NMR (400 MHz, $CDCl_3$): δ 7.78 (d, *J* = 8.2 Hz, 2H), 7.71 (d, *J* = 8.2 Hz, 2H), 7.34 (d, *J* = 8.1 Hz, 2H), 7.14–6.98 (m, 5H), 6.85 (dd, *J* = 6.5, 2.9 Hz, 2H), 5.82 (ddd, *J* = 17.6, 10.0, 7.7 Hz, 1H), 5.00 (d, *J* = 10.0 Hz, 1H), 4.95 (d, *J* = 17.6 Hz, 1H), 4.34 (d, *J* = 16.0 Hz, 1H), 3.85 (s, 3H), 3.72 (dd, *J* = 11.3, 2.9 Hz, 1H), 3.49–3.28 (m, 2H), 3.05 (dd, *J* = 11.3, 4.0 Hz, 1H), 2.43 (s, 3H);

^{13}C NMR (100 MHz, $CDCl_3$): δ 166.6, 143.8, 143.7, 139.3, 137.1, 136.5, 132.7, 130.8, 129.7, 129.3, 129.28, 129.22, 128.5, 127.8, 127.7, 126.8, 117.2, 52.0, 48.9, 47.9, 44.8, 21.5;

IR (KBr): 3416, 2953, 1717, 1607, 1437, 1279, 1115, 928, 859, 777, 676, 549 cm^{-1};

HRMS-ESI (m/z) Calcd for ($C_{28}H_{27}NO_4SNa$) ($[M+Na]^+$): 496.1553; found: 496.1556;

HPLC conditions: AD-H column (10% ^{i}PrOH in hexane, 1.0 mL/min, λ = 254 nm, 35 °C), t_R (major) = 23.7 min, t_R (minor) = 31.0 min;

Optical Rotation: $[\alpha]^{25}_D$ = -226.0 (*c* = 2.15, $CHCl_3$) for 94% ee.

Absolute stereochemistry was determined through analogy with **3aj**.

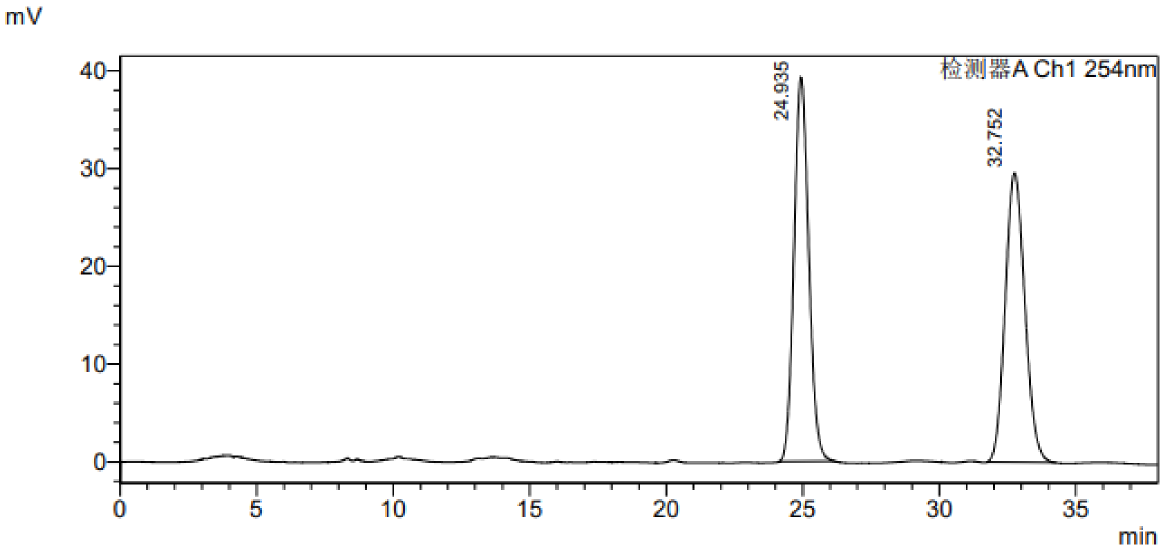

Peak#	Ret. Time	Area	Height	Conc.	Unit	Mark	Name
1	24.935	1472885	39256	49.889		M	
2	32.752	1479465	29587	50.111		M	
总计		2952350	68843	100.000			

Supplementary Figure 40. HPLC spectrum of racemic-**3ae**

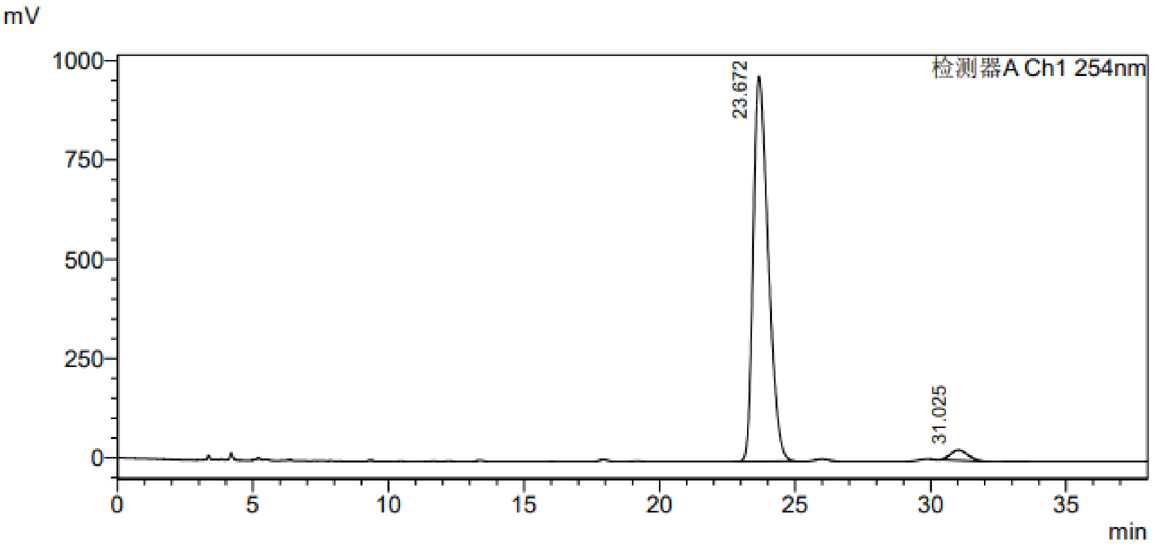

Peak#	Ret. Time	Area	Height	Conc.	Unit	Mark	Name
1	23.672	37164721	969469	96.961		M	
2	31.025	1165027	26273	3.039		M	
总计		38329748	995741	100.000			

Supplementary Figure 41. HPLC spectrum of (*R*)-**3ae**

(*R*)-4-phenyl-1-tosyl-5-(4-(trifluoromethyl)phenyl)-3-vinyl-1,2,3,6-tetrahydropyridine (3af)

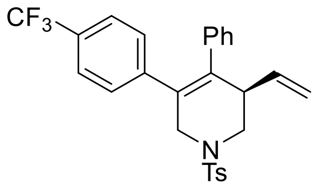

Chemical Formula: $C_{27}H_{24}F_3NO_2S$
Exact Mass: 483.1480

3af was prepared according to general procedure **2.1** using (*Z*)-4-((4-methyl-*N*-(3-phenylprop-2-yn-1-yl)phenyl)sulfonamido)but-2-en-1-yl acetate **1a** (0.2 mmol, 79.5 mg), 1-iodo-4-(trifluoromethyl)benzene **2f** (0.4 mmol, 108.8 mg), and was purified by silica gel column chromatography (PE/EA = 10/1) to obtain **3af** as a white solid (69.4 mg, 72% yield, 93% ee);

^{1}H NMR (400 MHz, $CDCl_3$): δ 7.72 (d, *J* = 8.0 Hz, 2H), 7.36 (t, *J* = 8.8 Hz, 4H), 7.15–7.05 (m, 5H), 6.95–6.76 (m, 2H), 5.84 (ddd, *J* = 17.7, 10.2, 7.8 Hz, 1H), 4.99 (d, *J* = 10.2 Hz, 1H), 4.96 (d, *J* = 17.7 Hz, 1H), 4.34 (d, *J* = 16.0 Hz, 1H), 3.75 (dd, *J* = 11.4, 3.0 Hz, 1H), 3.45–3.35 (m, 2H), 3.07 (dd, *J* = 11.4, 4.0 Hz, 1H), 2.43 (s, 3H);

^{13}C NMR (100 MHz, $CDCl_3$): δ 143.8, 142.7, 139.1, 137.3, 136.4, 132.6, 130.4, 129.7, 129.6, 129.3, 128.9 (q, *J* = 32.3 Hz), 127.8, 127.7, 126.8, 124.9 (q, *J* = 3.6 Hz), 123.9 (q, *J* = 270.5 Hz), 117.2, 48.9, 47.8, 44.8, 21.4;

^{19}F NMR (565 MHz, $CDCl_3$): δ -62.61; IR (KBr): 3416, 2928, 1925, 1615, 1494, 1328, 1125, 928, 842, 737, 669, 552 cm^{-1};

HRMS-ESI (m/z) Calcd for ($C_{27}H_{24}F_3NO_2SNa$) ($[M+Na]^+$): 506.1372; found: 506.1380;

HPLC conditions: AD-H column (2% ^{i}PrOH in hexane, 1.0 mL/min, λ = 254 nm, 35 °C), t_R (major) = 23.2 min, t_R (minor) = 28.7 min;

Optical Rotation: $[\alpha]^{25}_D$ = -227.1 (*c* = 3.10, $CHCl_3$) for 93% ee;

Absolute stereochemistry was determined through analogy with **3aj**.

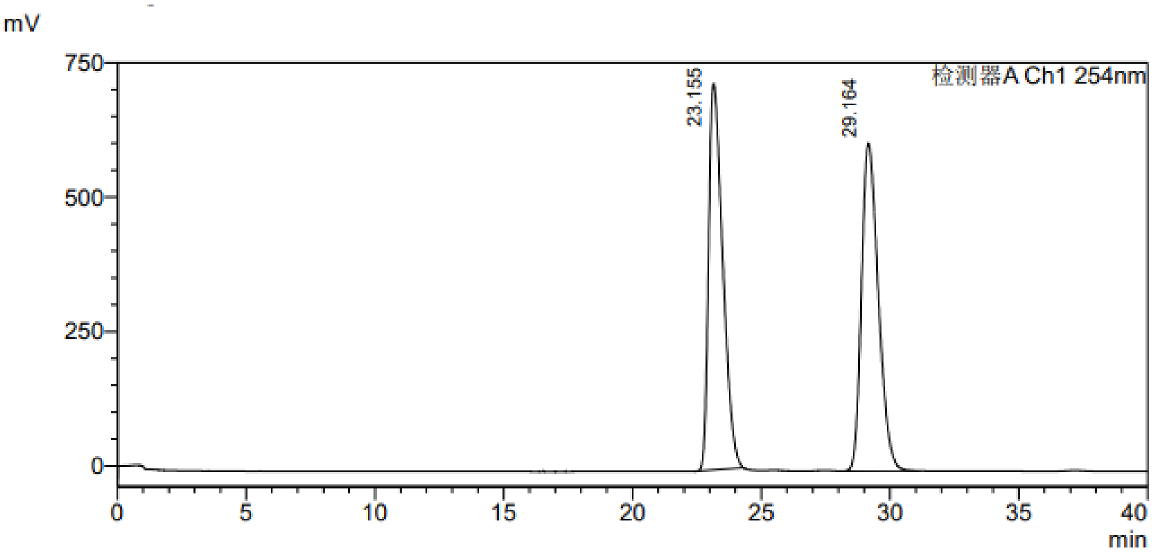

Peak#	Ret. Time	Area	Height	Conc.	Unit	Mark	Name
1	23.155	27348400	717731	49.615		M	
2	29.164	27772567	609924	50.385		M	
总计		55120967	1327655	100.000			

Supplementary Figure 42. HPLC spectrum of racemic-**3af**

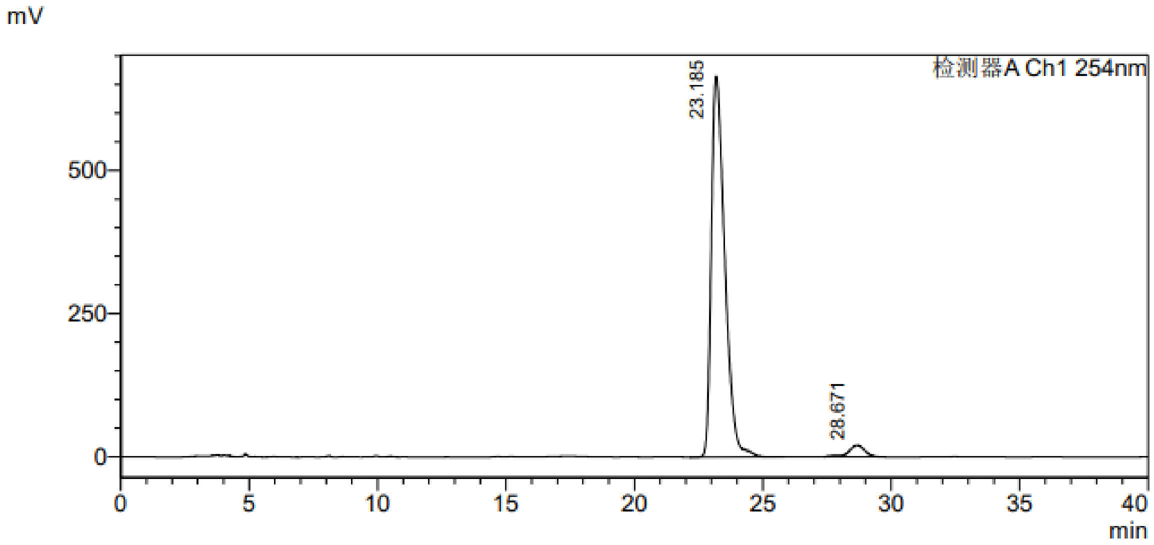

Peak#	Ret. Time	Area	Height	Conc.	Unit	Mark	Name
1	23.185	24227497	664814	96.454		M	
2	28.671	890761	20226	3.546		M	
总计		25118258	685039	100.000			

Supplementary Figure 43. HPLC spectrum of (*R*)-**3af**

(*R*)-5-(4-fluorophenyl)-4-phenyl-1-tosyl-3-vinyl-1,2,3,6-tetrahydropyridine (3ag)

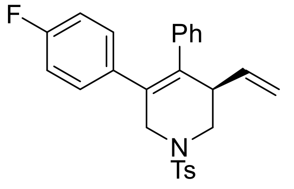

Chemical Formula: $C_{26}H_{24}FNO_2S$
Exact Mass: 433.1512

3ag was prepared according to general procedure **2.1** using (*Z*)-4-((4-methyl-*N*-(3-phenylprop-2-yn-1-yl)phenyl)sulfonamido)but-2-en-1-yl acetate **1a** (0.2 mmol, 79.5 mg) and 1-fluoro-4-iodobenzene **2g** (0.4 mmol, 88.8 mg). Purification by silica gel column chromatography (PE/EA = 20/1) gave **3ag** as a white solid (43.5 mg, 50% yield).

^{1}H NMR (400 MHz, $CDCl_3$): δ 7.71 (d, *J* = 8.3 Hz, 2H), 7.34 (d, *J* = 8.0 Hz, 2H), 7.15–7.02 (m, 3H), 6.94 (dd, *J* = 8.7, 5.4 Hz, 2H), 6.86 (dd, *J* = 7.6, 1.9 Hz, 2H), 6.80 (t, *J* = 8.7 Hz, 2H), 5.83 (ddd, *J* = 17.7, 10.2, 7.7 Hz, 1H), 5.00 (d, *J* = 10.2 Hz, 1H), 4.97 (d, *J* = 17.7 Hz, 1H), 4.30 (d, *J* = 16.0 Hz, 1H), 3.73 (dd, *J* = 11.4, 3.0 Hz, 1H), 3.53–3.22 (m, 2H), 3.02 (dd, *J* = 11.4, 4.0 Hz, 1H), 2.44 (s, 3H);

^{13}C NMR (100 MHz, $CDCl_3$): δ 161.6 (d, *J* = 245.3 Hz), 143.7, 139.6, 136.6, 136.1, 134.8 (d, *J* = 3.5 Hz), 132.7, 130.9 (d, *J* = 8.0 Hz), 130.6, 129.7, 129.3, 127.8, 126.6, 117.0, 115.0 (d, *J* = 21.2 Hz), 49.2, 47.9, 44.7, 21.5;

^{19}F NMR (565 MHz, $CDCl_3$): δ -114.57 – -114.74 (m);

IR (KBr): 3436, 2921, 1600, 1509, 1343, 1166, 1093, 926, 836, 762, 654, 547 cm^{-1};

HRMS-ESI (m/z) Calcd for ($C_{26}H_{24}FNO_2SNa$) ($[M+Na]^+$): 456.1404; found: 456.1411;

HPLC conditions: OD-H column (2% ^{i}PrOH in hexane, 0.8 mL/min, λ = 254 nm, 35 °C), t_R (major) = 15.8 min, t_R (minor) = 17.5 min;

Optical Rotation: $[\alpha]^{25}_D$ = -255.5 (*c* = 1.60, $CHCl_3$) for 95% ee;

Absolute stereochemistry was determined through analogy with **3aj**.

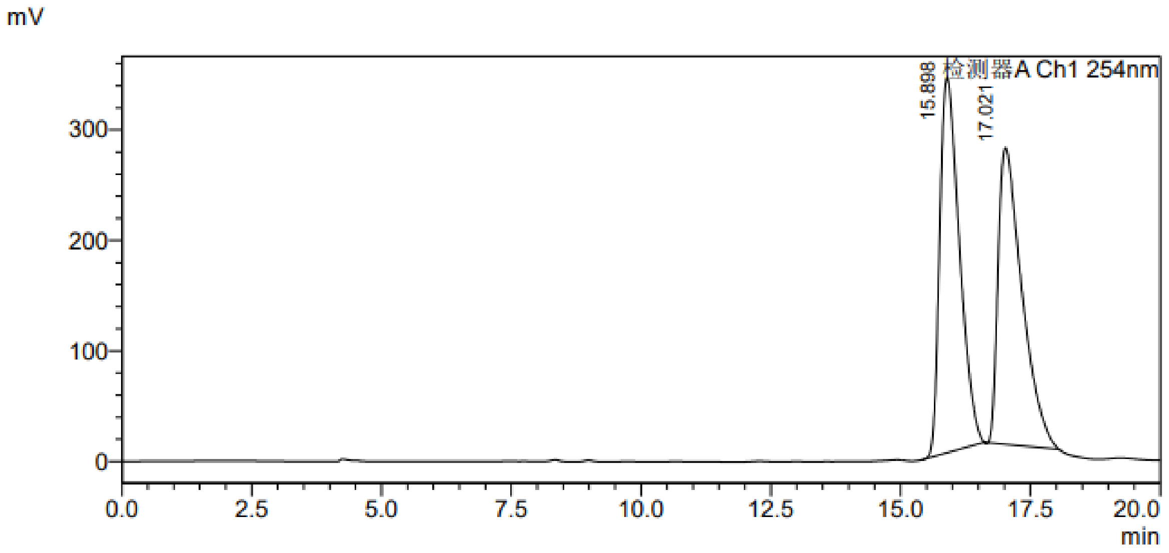

Peak#	Ret. Time	Area	Height	Conc.	Unit	Mark	Name
1	15.898	8971315	339278	51.082		M	
2	17.021	8591379	268336	48.918		M	
总计		17562694	607614	100.000			

Supplementary Figure 44. HPLC spectrum of racemic-**3ag**

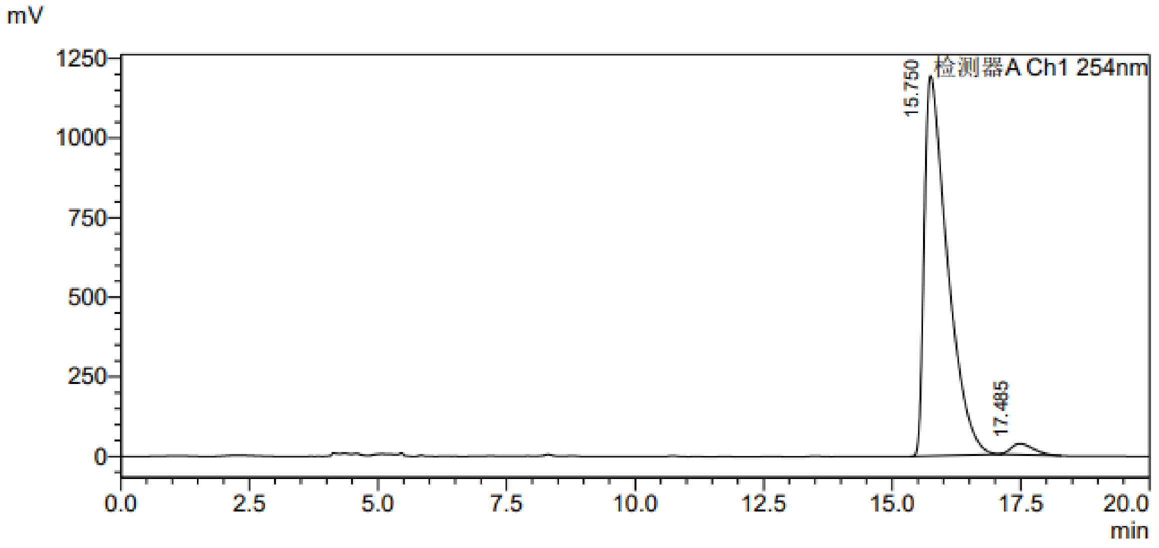

Peak#	Ret. Time	Area	Height	Conc.	Unit	Mark	Name
1	15.750	37538831	1194038	97.312		M	
2	17.485	1036804	34295	2.688		M	
总计		38575634	1228333	100.000			

Supplementary Figure 45. HPLC spectrum of (*R*)-**3ag**

(*R*)-4-phenyl-5-(4-(4,4,5,5-tetramethyl-1,3,2-dioxaborolan-2-yl)phenyl)-1-tosyl-3-vinyl-1,2,3,6-tetrahydropyridine (3ah)

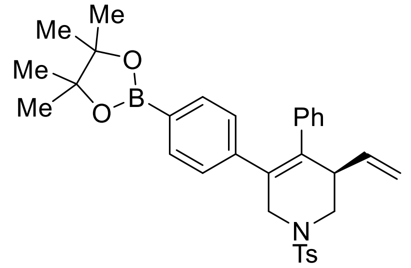

Chemical Formula: $C_{32}H_{36}BNO_4S$
Exact Mass: 541.2458

3ah was prepared according to general procedure **2.1** using (*Z*)-4-((4-methyl-*N*-(3-phenylprop-2-yn-1-yl)phenyl)sulfonamido)but-2-en-1-yl acetate **1a** (0.2 mmol, 79.5 mg) and 2-(4-iodophenyl)-4,4,5,5-tetramethyl-1,3,2-dioxaborolane **2h** (0.4 mmol, 132.0 mg). Purification by silica gel column chromatography (PE/EA = 20/1) gave **3ah** as a white solid (52.3 mg, 48% yield).

^{1}H NMR (400 MHz, $CDCl_3$): δ 7.70 (d, *J* = 8.4 Hz, 2H), 7.57 (d, *J* = 8.0 Hz, 2H), 7.33 (d, *J* = 8.4 Hz, 2H), 7.13–7.03 (m, 3H), 7.00 (d, *J* = 8.0 Hz, 2H), 6.92–6.85 (m, 2H), 5.85 (ddd, *J* = 17.6, 10.0, 8.0 Hz, 1H), 5.00 (d, *J* = 10.0 Hz, 1H), 4.96 (d, *J* = 17.6 Hz, 1H), 4.36 (d, *J* = 16.0 Hz, 1H), 3.75 (dd, *J* = 11.2, 2.2 Hz, 1H), 3.45–3.30 (m, 2H), 3.01 (dd, *J* = 11.2, 3.8 Hz, 1H), 2.43 (s, 3H), 1.30 (s, 12H);

^{13}C NMR (100 MHz, $CDCl_3$): δ 143.6, 141.9, 139.6, 136.7, 135.9, 134.3, 132.6, 131.5, 129.7, 129.3, 128.6, 127.8, 127.7, 126.5, 116.9, 83.7, 49.1, 47.9, 44.7, 24.8, 24.7, 21.4;

IR (KBr): 3456, 2978, 1609, 1359, 1167, 1092, 917, 770, 660, 549 cm^{-1};

HRMS-ESI (m/z) Calcd for ($C_{32}H_{36}BNO_4SNa$) ($[M+Na]^+$): 564.2350; found: 564.2363;

HPLC conditions: OD-H column (2% ^{i}PrOH in hexane, 0.5 mL/min, λ = 254 nm, 35 °C), t_R (minor) = 23.9 min, t_R (major) = 26.3 min;

Optical Rotation: $[\alpha]^{25}_D$ = -185.4 (*c* = 2.13, $CHCl_3$) for 94% ee;

Absolute stereochemistry was determined through analogy with **3aj**.

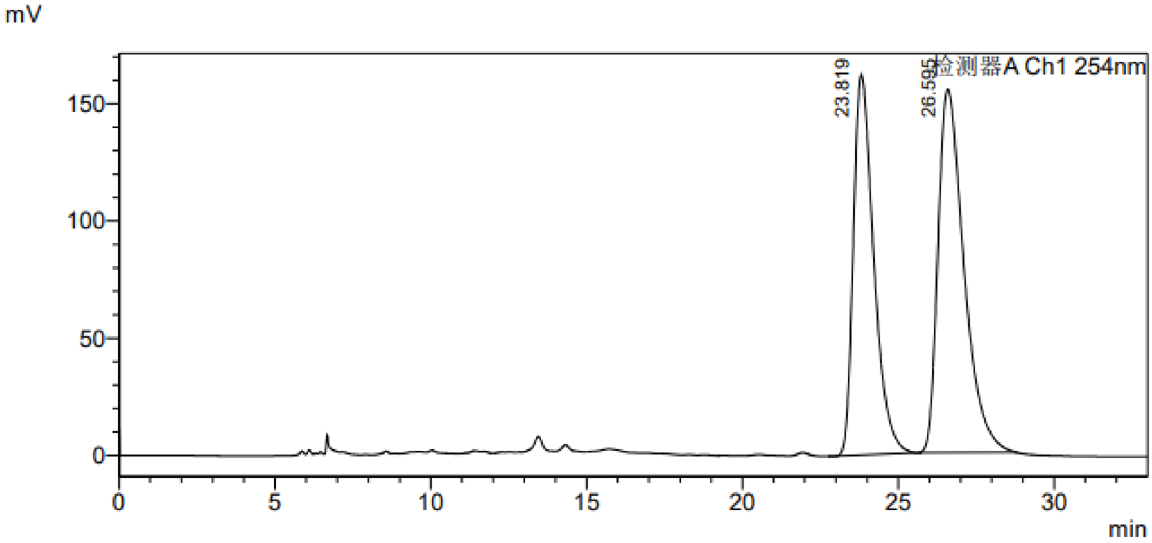

Peak#	Ret. Time	Area	Height	Conc.	Unit	Mark	Name
1	23.819	7371437	162216	45.097		M	
2	26.595	8974245	154967	54.903		M	
总计		16345683	317183	100.000			

Supplementary Figure 46. HPLC spectrum of racemic-**3ah**

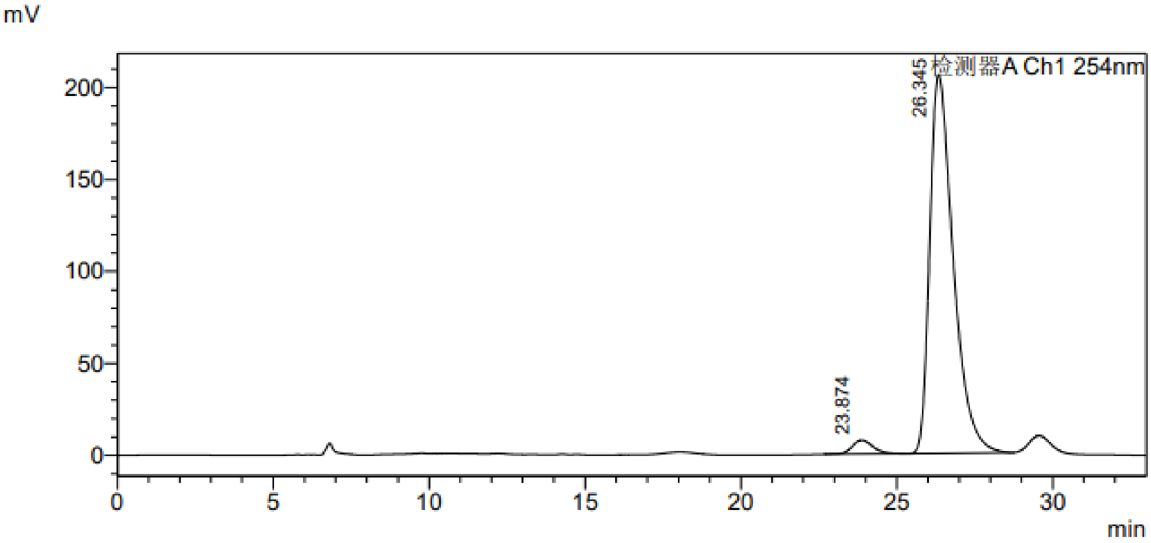

Peak#	Ret. Time	Area	Height	Conc.	Unit	Mark	Name
1	23.874	355517	7578	3.185		M	
2	26.345	10807754	205877	96.815		M	
总计		11163272	213455	100.000			

Supplementary Figure 47. HPLC spectrum of (*R*)-**3ah**

(*R*)-4-phenyl-5-(m-tolyl)-1-tosyl-3-vinyl-1,2,3,6-tetrahydropyridine (3ai)

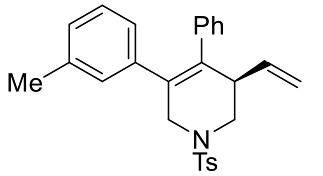

3ai was prepared according to general procedure **2.1** using (*Z*)-4-((4-methyl-*N*-(3-phenylprop-2-yn-1-yl)phenyl)sulfonamido)but-2-en-1-yl acetate **1a** (0.2 mmol, 79.5 mg) and 1-iodo-3-methylbenzene **2i** (0.4 mmol, 87.2 mg). Purification by silica gel column chromatography (PE/EA = 20/1) gave **3ai** as a white solid (53.0 mg, 62% yield).

^{1}H NMR (400 MHz, $CDCl_3$): δ 7.72 (d, *J* = 8.1 Hz, 2H), 7.34 (d, *J* = 8.1 Hz, 2H), 7.14–7.02 (m, 3H), 6.98 (t, *J* = 7.5 Hz, 1H), 6.95–6.83 (m, 4H), 6.72 (d, *J* = 7.5 Hz, 1H), 5.85 (ddd, *J* = 17.6, 10.0, 8.0 Hz, 1H), 5.00 (d, *J* = 10.0 Hz, 1H), 4.96 (d, *J* = 17.6 Hz, 1H), 4.37 (d, *J* = 15.9 Hz, 1H), 3.76 (dd, *J* = 11.3, 2.5 Hz, 1H), 3.54–3.26 (m, 2H), 3.01 (dd, *J* = 11.3, 3.9 Hz, 1H), 2.44 (s, 3H), 2.20 (s, 3H);

^{13}C NMR (100 MHz, $CDCl_3$): δ 143.6, 139.9, 138.8, 137.5, 136.8, 135.3, 132.7, 131.7, 129.8, 129.7, 129.3, 127.8, 127.7, 127.6, 126.5, 126.4, 116.8, 49.3, 47.9, 44.7, 21.5, 21.2;

IR (KBr): 3415, 2919, 1599, 1343, 1165, 1092, 939, 788, 701, 549 cm^{-1};

HRMS-ESI (m/z) Calcd for ($C_{27}H_{27}NO_2SNa$) ($[M+Na]^+$): 452.1655; found: 452.1660;

HPLC conditions: AD-H column (2% ^{i}PrOH in hexane, 1.0 mL/min, λ = 254 nm, 35 °C), t_R (minor) = 15.1 min, t_R (major) = 16.0 min;

Optical Rotation: $[\alpha]^{25}_{D}$ = -220.1 (*c* = 2.50, $CHCl_3$) for 95% ee;

Absolute stereochemistry was determined through analogy with **3aj**.

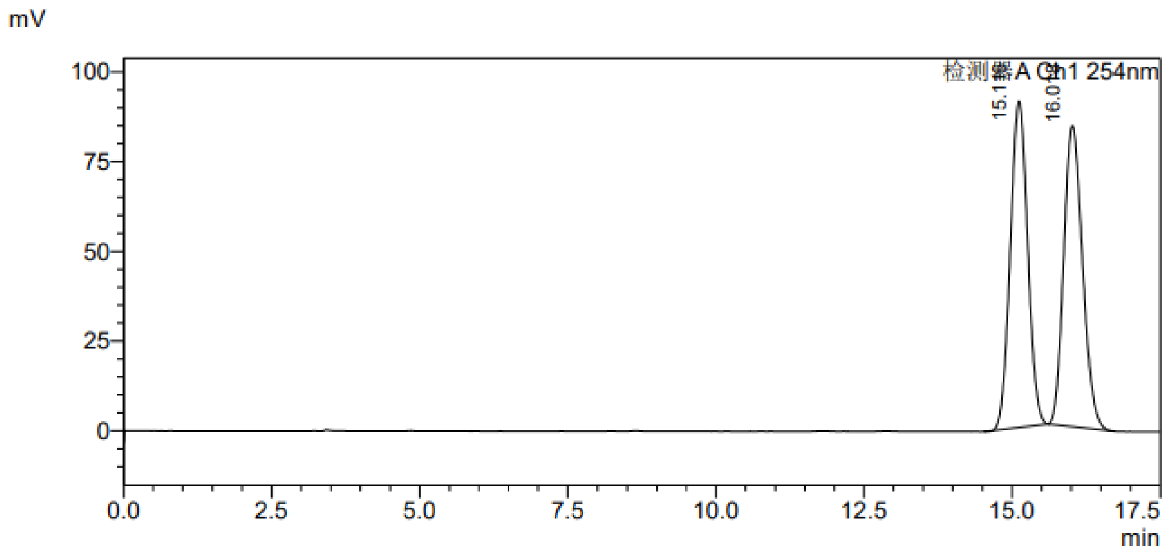

Peak#	Ret. Time	Area	Height	Conc.	Unit	Mark	Name
1	15.116	1855662	90912	50.072		M	
2	16.018	1850333	83774	49.928		M	
总计		3705996	174687	100.000			

Supplementary Figure 48. HPLC spectrum of racemic-**3ai**

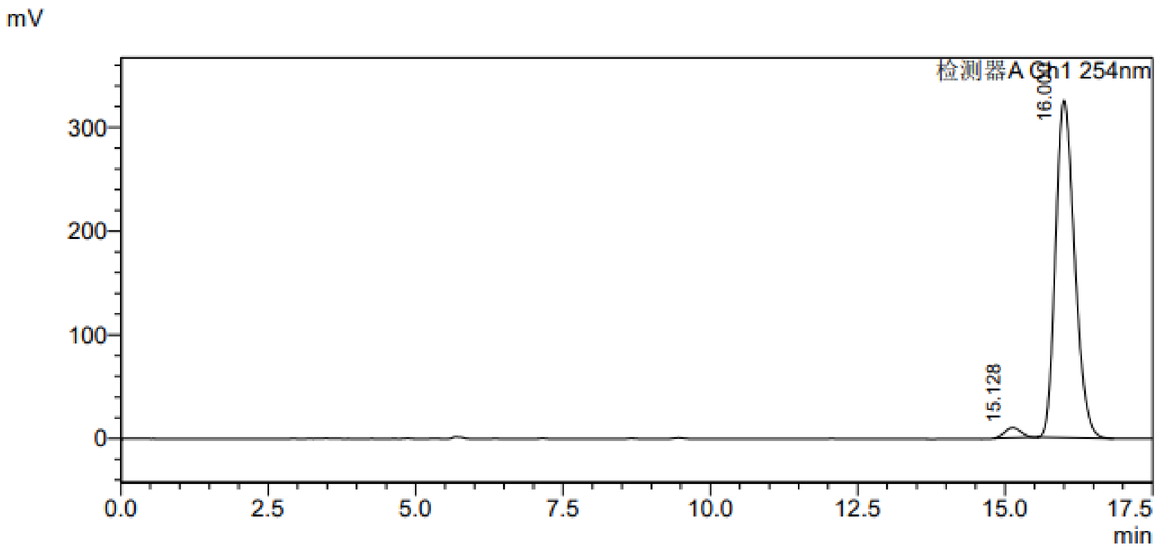

Peak#	Ret. Time	Area	Height	Conc.	Unit	Mark	Name
1	15.128	187612	9868	2.491		M	
2	16.000	7344096	325203	97.509		M	
总计		7531708	335071	100.000			

Supplementary Figure 49. HPLC spectrum of (*R*)-**3ai**

(*R*)-5-(3-chlorophenyl)-4-phenyl-1-tosyl-3-vinyl-1,2,3,6-tetrahydropyridine (3aj)

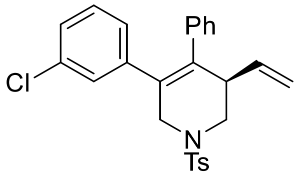

Chemical Formula: $C_{26}H_{24}ClNO_2S$
Exact Mass: 449.1216

3aj was prepared according to general procedure **2.1** using (*Z*)-4-((4-methyl-*N*-(3-phenylprop-2-yn-1-yl)phenyl)sulfonamido)but-2-en-1-yl acetate **1a** (0.2 mmol, 79.5 mg) and 1-chloro-3-iodobenzene **2j** (0.4 mmol, 95.4 mg). Purification by silica gel column chromatography (PE/EA = 20/1) gave **3aj** as a white solid (70.1 mg, 78% yield).

^{1}H NMR (400 MHz, $CDCl_3$): δ 7.71 (d, *J* = 8.0 Hz, 2H), 7.35 (d, *J* = 8.0 Hz, 2H), 7.16–6.97 (m, 6H), 6.93–6.77 (m, 3H), 5.83 (ddd, *J* = 17.6, 10.0, 7.8 Hz, 1H), 4.99 (d, *J* = 10.0 Hz, 1H), 4.96 (d, *J* = 17.6 Hz, 1H), 4.30 (d, *J* = 16.2 Hz, 1H), 3.73 (dd, *J* = 11.3, 2.6 Hz, 1H), 3.50–3.30 (m, 2H), 3.03 (dd, *J* = 11.3, 3.8 Hz, 1H), 2.44 (s, 3H);

^{13}C NMR (100 MHz, $CDCl_3$): δ 143.8, 140.8, 139.3, 136.8, 136.4, 133.7, 132.6, 130.4, 129.7, 129.3, 129.2, 129.1, 127.8, 127.7, 127.6, 127.1, 126.8, 117.1, 49.0, 47.9, 44.7, 21.5;

IR (KBr): 3473, 2918, 1593, 1337, 1163, 1092, 939, 791, 705, 574 cm^{-1};

HRMS-ESI (m/z) Calcd for ($C_{26}H_{24}ClNO_2SNa$) ($[M+Na]^+$): 472.1108; found: 472.1113;

HPLC conditions: AS-H column (2% ^{i}PrOH in hexane, 1.0 mL/min, λ = 254 nm, 35 °C), t_R (minor) = 19.4 min, t_R (major) = 28.3 min;

Optical Rotation: $[\alpha]^{25}_D$ = -220.0 (*c* = 2.75, $CHCl_3$) for 98% ee;

Absolute stereochemistry was determined by X-ray diffraction crystallography.

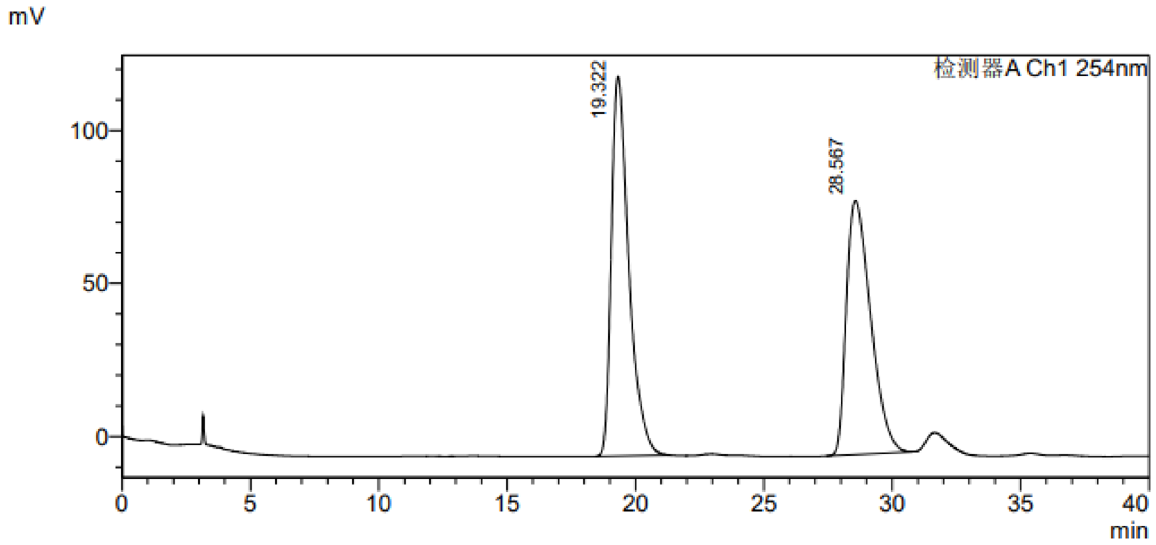

Peak#	Ret. Time	Area	Height	Conc.	Unit	Mark	Name
1	19.322	5889858	123979	51.790		M	
2	28.567	5482635	83061	48.210		M	
总计		11372493	207040	100.000			

Supplementary Figure 50. HPLC spectrum of racemic-**3aj**

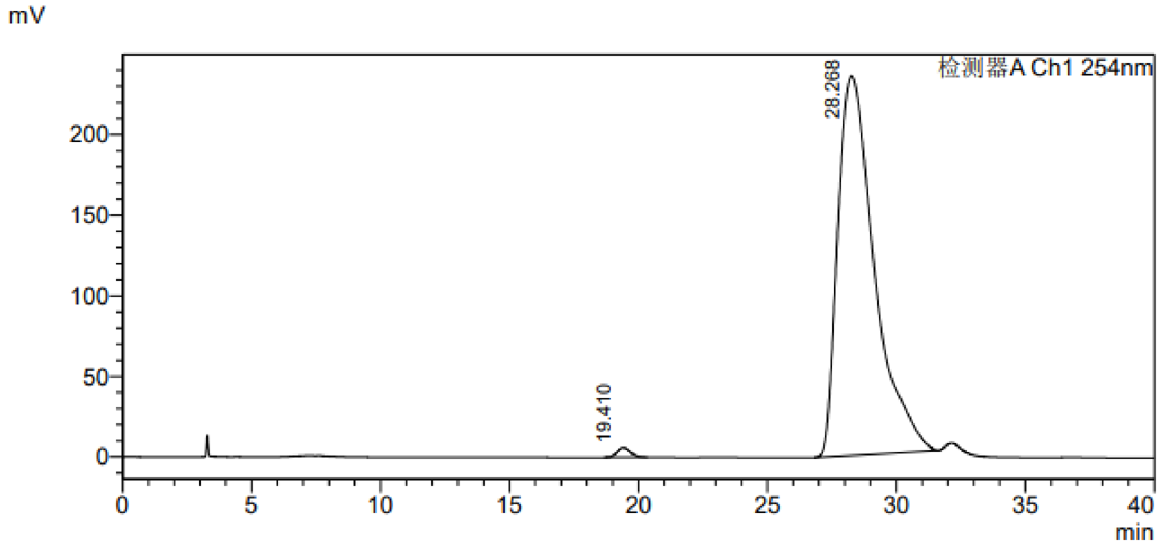

Peak#	Ret. Time	Area	Height	Conc.	Unit	Mark	Name
1	19.410	214618	6020	0.913		M	
2	28.268	23283223	235528	99.087		M	
总计		23497840	241547	100.000			

Supplementary Figure 51. HPLC spectrum of (*R*)-**3aj**

(*R*)-5-(3,5-dimethylphenyl)-4-phenyl-1-tosyl-3-vinyl-1,2,3,6-tetrahydropyridine (3ak)

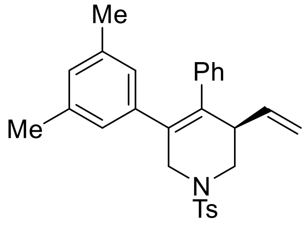

Chemical Formula: $C_{28}H_{29}NO_2S$
Exact Mass: 443.1919

3ak was prepared according to general procedure **2.1** using (*Z*)-4-((4-methyl-*N*-(3-phenylprop-2-yn-1-yl)phenyl)sulfonamido)but-2-en-1-yl acetate **1a** (0.2 mmol, 79.5 mg) and 1-iodo-3,5-dimethylbenzene **2k** (0.4 mmol, 92.8 mg). Purification by silica gel column chromatography (PE/EA = 20/1) gave **3ak** as a white solid (51.9 mg, 59% yield).

^{1}H NMR (400 MHz, $CDCl_3$): δ 7.70 (d, *J* = 8.0 Hz, 2H), 7.32 (d, *J* = 8.0 Hz, 2H), 7.14–7.00 (m, 3H), 6.96–6.82 (m, 2H), 6.74 (s, br, 1H), 6.59 (s, br, 2H), 5.85 (ddd, *J* = 17.6, 10.0, 7.8 Hz, 1H), 5.02–4.90 (m, 2H), 4.34 (d, *J* = 16.0 Hz, 1H), 3.75 (dd, *J* = 11.2, 2.4 Hz, 1H), 3.46–3.26 (m, 2H), 2.97 (dd, *J* = 11.2, 4.0 Hz, 1H), 2.43 (s, 3H), 2.12 (s, 6H);

^{13}C NMR (100 MHz, $CDCl_3$): δ 143.6, 140.0, 138.8, 137.3, 136.9, 135.1, 132.9, 131.8, 129.6, 129.4, 128.6, 127.8, 127.5, 127.1, 126.4, 116.8, 49.4, 48.0, 44.7, 21.5, 21.1;

IR(KBr): 3433, 2917, 1598, 1446, 1346, 1165, 1017, 936, 816, 702, 680, 548 cm^{-1};

HRMS-ESI (m/z) Calcd for ($C_{28}H_{29}NO_2SNa$) ($[M+Na]^+$): 466.1811; found: 466.1808;

HPLC conditions: AS-H column (10% ^{i}PrOH in hexane, 1.0 mL/min, λ = 254 nm, 35 °C), t_R (minor) = 10.4 min, t_R (major) = 11.4 min;

Optical Rotation: $[\alpha]^{25}_{D}$ = -257.2 (*c* = 0.83, $CHCl_3$) for 95% ee;

Absolute stereochemistry was determined through analogy with **3aj**.

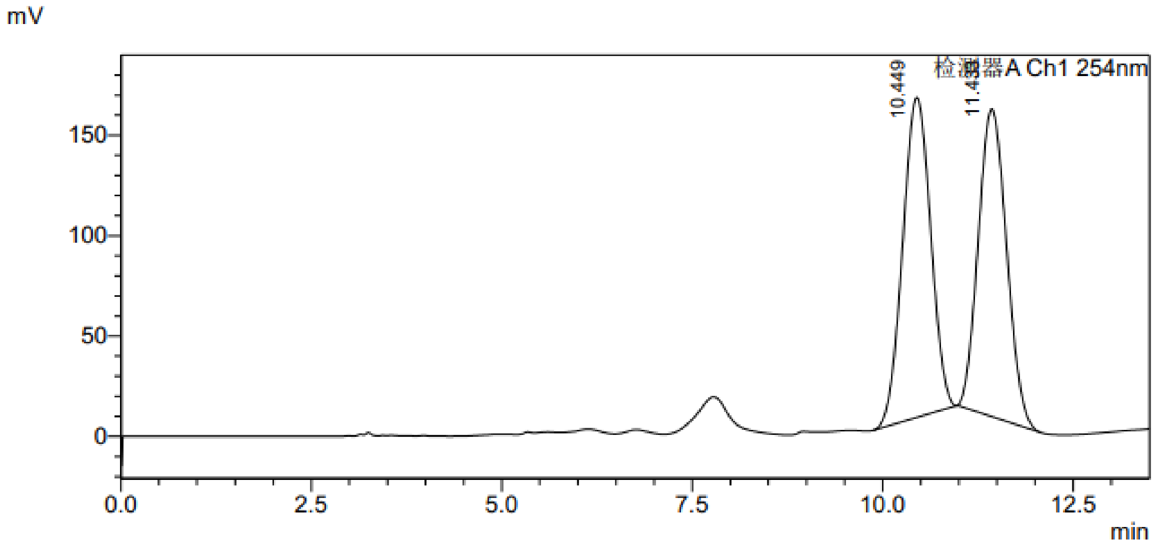

Peak#	Ret. Time	Area	Height	Conc.	Unit	Mark	Name
1	10.449	4069111	159313	50.770		M	
2	11.433	3945665	153086	49.230		M	
总计		8014776	312399	100.000			

Supplementary Figure 52. HPLC spectrum of racemic-**3ak**

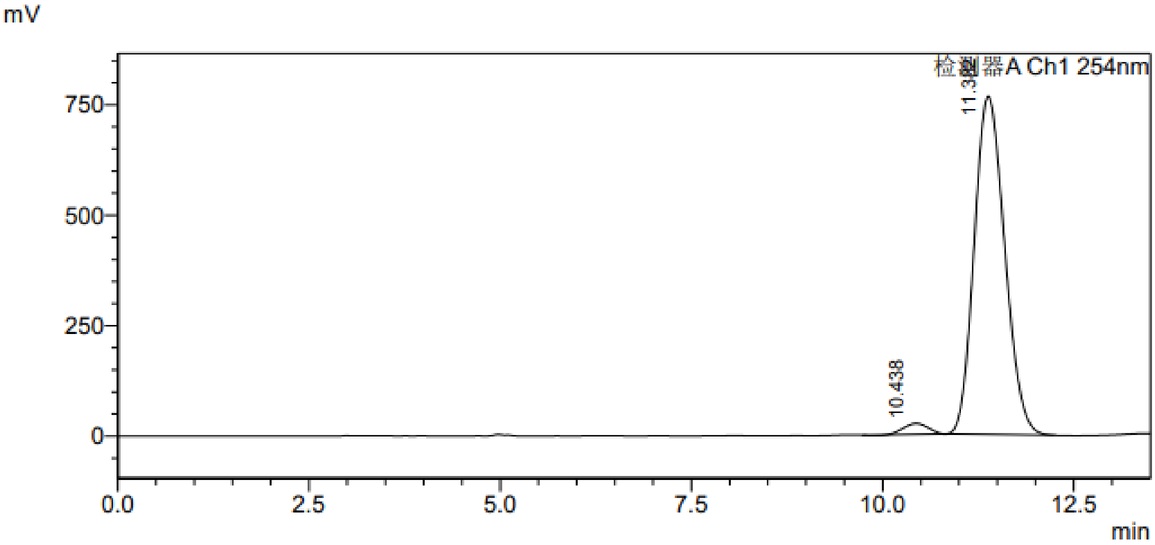

Peak#	Ret. Time	Area	Height	Conc.	Unit	Mark	Name
1	10.438	536585	24485	2.466		M	
2	11.382	21224508	765576	97.534		M	
总计		21761093	790061	100.000			

Supplementary Figure 53. HPLC spectrum of (*R*)-**3ak**

(*R*)-5-(benzo[d][1,3]dioxol-5-yl)-4-phenyl-1-tosyl-3-vinyl-1,2,3,6-tetrahydropyridine (3al)

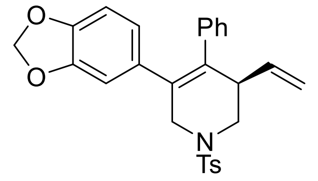

Chemical Formula: $C_{27}H_{25}NO_4S$
Exact Mass: 459.1504

3al was prepared according to general procedure **2.1** using (*Z*)-4-((4-methyl-*N*-(3-phenylprop-2-yn-1-yl)phenyl)sulfonamido)but-2-en-1-yl acetate **1a** (0.2 mmol, 79.5 mg) and 5-iodobenzo[*d*][1,3]dioxole **2l** (0.4 mmol, 99.2 mg). Purification by silica gel column chromatography (PE/EA = 10/1) gave **3ak** as a white solid (48.2 mg, 52% yield).

^{1}H NMR (400 MHz, $CDCl_3$): δ 7.71 (d, *J* = 8.0 Hz, 2H), 7.34 (d, *J* = 8.0 Hz, 2H), 7.18–7.01 (m, 3H), 6.90 (d, *J* = 6.7 Hz, 2H), 6.56 (d, *J* = 8.5 Hz, 1H), 6.45 (d, *J* = 6.9 Hz, 2H), 5.98–5.67 (m, 3H), 4.98 (d, *J* = 10.0 Hz, 1H), 4.95 (d, *J* = 17.6 Hz, 1H), 4.27 (d, *J* = 16.0 Hz, 1H), 3.72 (dd, *J* = 11.4, 2.3 Hz, 1H), 3.43–3.30 (m, 2H), 3.00 (dd, *J* = 11.4, 3.8 Hz, 1H), 2.43 (s, 3H);

^{13}C NMR (100 MHz, $CDCl_3$): δ 147.1, 146.4, 143.7, 139.9, 136.8, 135.5, 132.7, 131.2, 129.7, 129.3, 127.7, 126.5, 122.9, 116.9, 109.7, 107.9, 100.8, 49.4, 47.9, 44.7, 21.5;

IR (KBr): 3429, 2923, 1598, 1486, 1338, 1238, 1162, 1042, 940, 817, 657, 547 cm^{-1};

HRMS-ESI (m/z) Calcd for ($C_{27}H_{25}NO_4SNa$) ($[M+Na]^+$): 482.1397; found: 482.1402;

HPLC conditions: OD-H column (10% ^{i}PrOH in hexane, 1.0 mL/min, λ = 254 nm, 35 °C), t_R (major) = 9.6 min, t_R (minor) = 10.7 min;

Optical Rotation: $[\alpha]^{25}_D$ = -252.8 (*c* = 2.00, $CHCl_3$) for 92% ee;

Absolute stereochemistry was determined through analogy with **3aj**.

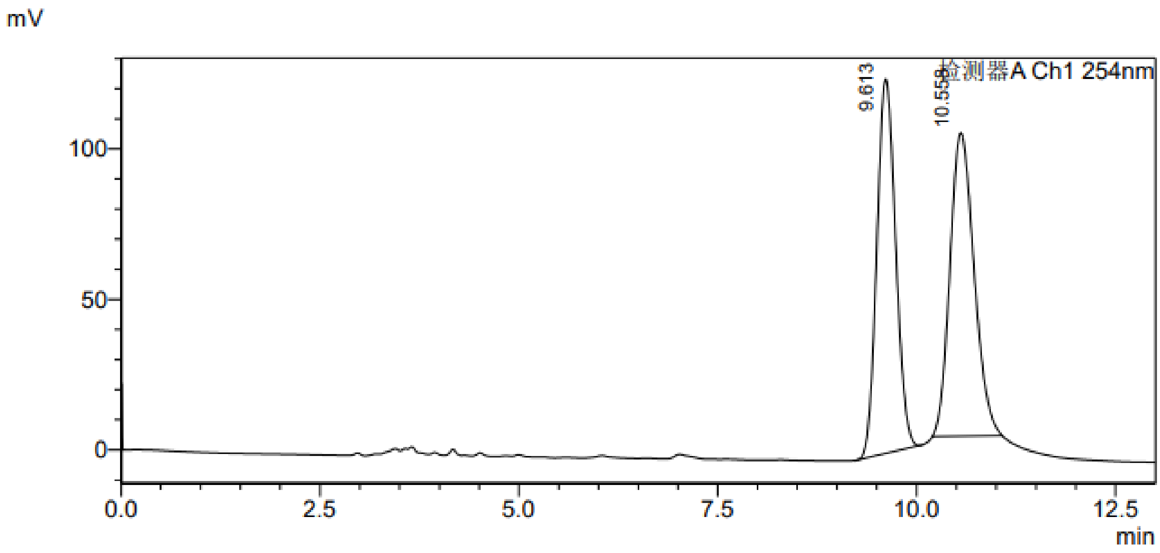

Peak#	Ret. Time	Area	Height	Conc.	Unit	Mark	Name
1	9.613	2037262	124254	49.012		M	
2	10.558	2119416	100761	50.988		M	
总计		4156678	225015	100.000			

Supplementary Figure 54. HPLC spectrum of racemic-**3al**

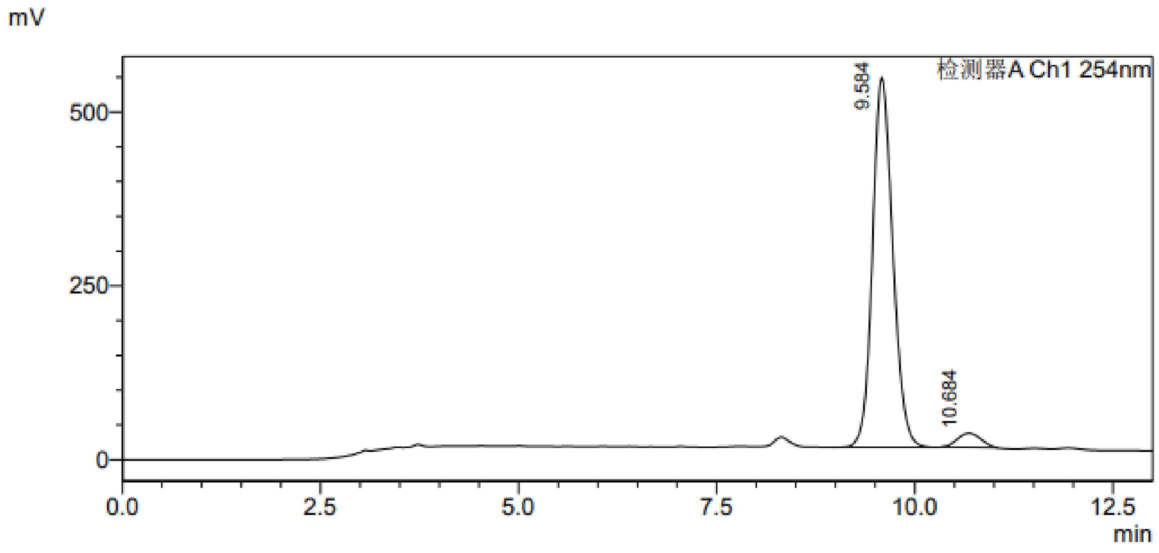

Peak#	Ret. Time	Area	Height	Conc.	Unit	Mark	Name
1	9.584	9470988	531195	95.805		M	
2	10.684	414719	20255	4.195		M	
总计		9885707	551450	100.000			

Supplementary Figure 55. HPLC spectrum of (*R*)-**3al**

(*R*)-5-(naphthalen-2-yl)-4-phenyl-1-tosyl-3-vinyl-1,2,3,6-tetrahydropyridine (3am)

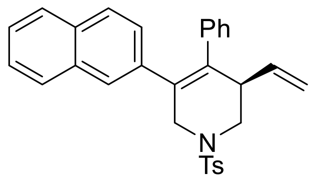

Chemical Formula: $C_{30}H_{27}NO_2S$
Exact Mass: 465.1762

3am was prepared according to general procedure **2.1** using (*Z*)-4-((4-methyl-*N*-(3-phenylprop-2-yn-1-yl)phenyl)sulfonamido)but-2-en-1-yl acetate **1a** (0.2 mmol, 79.5 mg) and 2-iodonaphthalene **2m** (0.4 mmol, 101.6 mg). Purification by silica gel column chromatography (PE/EA = 20/1) gave **3am** as a white solid (50.4 mg, 54% yield, 94% ee).

^{1}H NMR (400 MHz, $CDCl_3$): δ 7.76–7.66 (m, 4H), 7.60 (s, br, 1H), 7.51 (d, *J* = 8.4 Hz, 1H), 7.46–7.38 (m, 2H), 7.32 (d, *J* = 8.4 Hz, 2H), 7.10–7.00 (m, 3H), 6.98–6.89 (m, 3H), 5.89 (ddd, *J* = 17.6, 10.0, 7.8 Hz, 1H), 5.10–4.90 (m, 2H), 4.50 (d, *J* = 16.0 Hz, 1H), 3.80 (dd, *J* = 11.4, 2.6 Hz, 1H), 3.52–3.32 (m, 2H), 3.04 (dd, *J* = 11.2, 4.0 Hz, 1H), 2.42 (s, 3H);

^{13}C NMR (100 MHz, $CDCl_3$): δ 143.6, 139.7, 136.7, 136.4, 136.0, 132.8, 132.7, 132.0, 131.4, 129.6 (2C), 129.3 (2C), 127.8, 127.7 (4C), 127.4, 127.3, 126.5, 126.0, 125.9, 116.9, 49.3, 47.9, 44.7, 21.4;

IR(KBr): 3436, 2804, 1597, 1339, 1164, 1100, 998, 819, 705, 659, 545 cm^{-1};

HRMS-ESI (m/z) Calcd for ($C_{30}H_{27}NO_2SNa$) ([M+Na]$^+$): 488.1655; found: 488.1651;

HPLC conditions: AS-H column (10% ^{i}PrOH in hexane, 1.0 mL/min, λ = 254 nm, 35 °C), t_R (minor) = 12.0 min, t_R (major) = 17.2 min;

Optical Rotation: $[\alpha]^{25}_D$ = -245.8 (*c* = 1.90, $CHCl_3$) for 94% ee;

Absolute stereochemistry was determined through analogy with **3aj**.

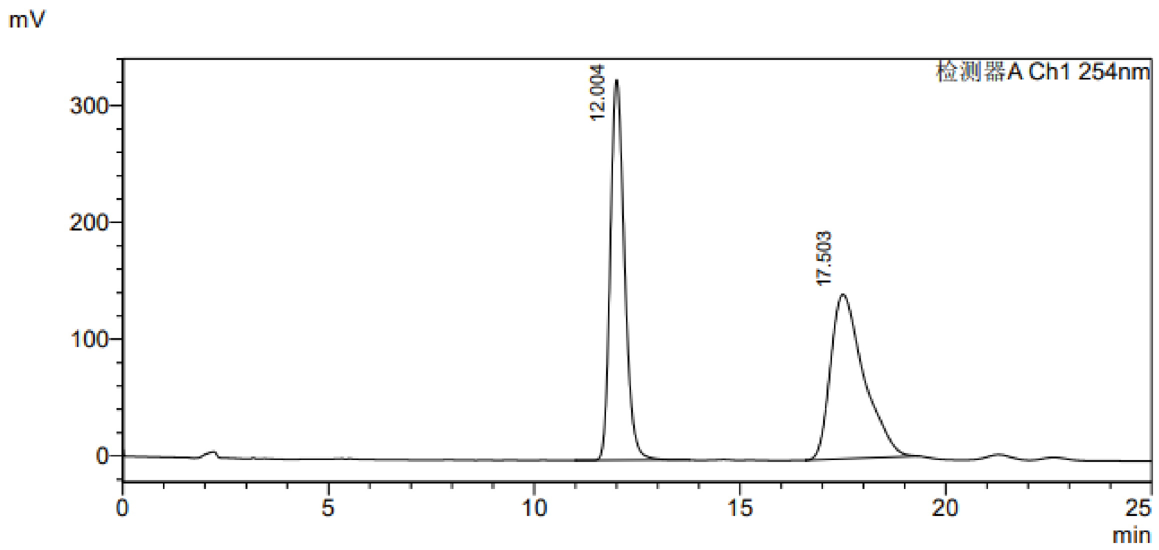

Peak#	Ret. Time	Area	Height	Conc.	Unit	Mark	Name
1	12.004	7813117	325463	49.623		M	
2	17.503	7931935	140921	50.377		M	
总计		15745052	466383	100.000			

Supplementary Figure 56. HPLC spectrum of racemic-**3am**

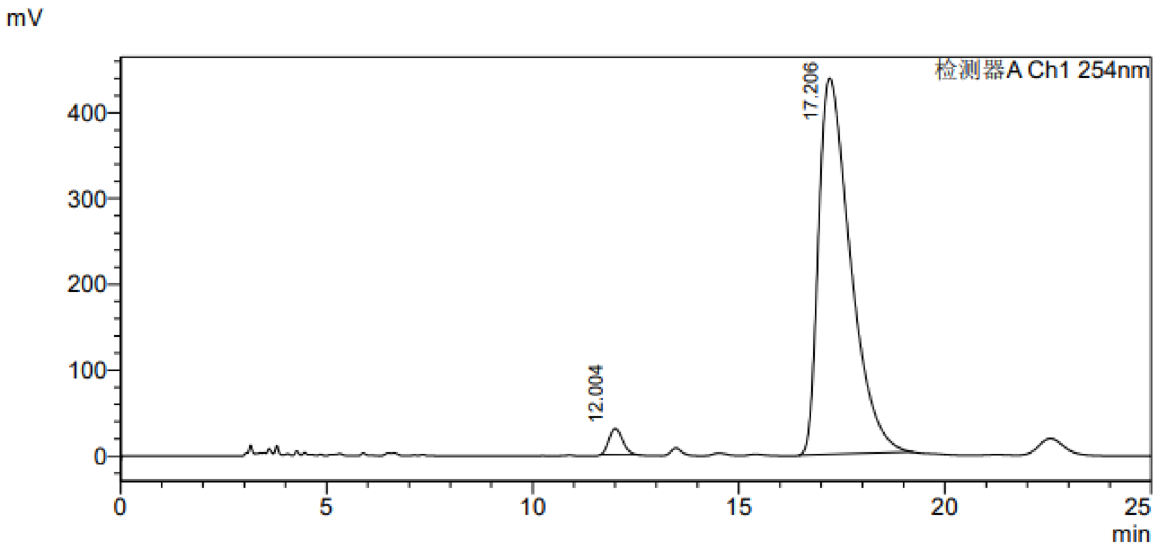

Peak#	Ret. Time	Area	Height	Conc.	Unit	Mark	Name
1	12.004	715596	30729	2.948		M	
2	17.206	23560954	438331	97.052		M	
总计		24276550	469060	100.000			

Supplementary Figure 57. HPLC spectrum of (*R*)-**3am**

(*R*)-5-phenyl-4-(p-tolyl)-1-tosyl-3-vinyl-1,2,3,6-tetrahydropyridine (3ba)

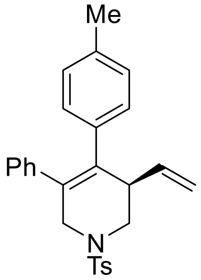

Chemical Formula: $C_{27}H_{27}NO_2S$
Exact Mass: 429.1762

3ba was prepared according to general procedure **2.1** using (*Z*)-4-((4-methyl-*N*-(3-(p-tolyl)prop-2-yn-1-yl)phenyl)sulfonamido)but-2-en-1-yl acetate **1b** (0.4 mmol, 82.3 mg) and iodobenzene **2a** (0.4 mmol, 81.6 mg). Purification by silica gel column chromatography (PE/EA = 20/1) gaqve **3ba** as a light yellow solid (56.7 mg, 66% yield).

^{1}H NMR (400 MHz, $CDCl_3$): δ 7.71 (d, *J* = 8.4 Hz, 2H), 7.33 (d, *J* = 8.0 Hz, 2H), 7.18–7.08 (m, 3H), 7.05–6.94 (m, 2H), 6.89 (d, *J* = 8.0 Hz, 2H), 6.78 (d, *J* = 8.0 Hz, 2H), 5.86 (ddd, *J* = 17.6, 10.0, 7.6 Hz, 1H), 5.01 (d, *J* = 10.0 Hz, 1H), 4.98 (d, *J* = 17.6 Hz, 1H), 4.36 (d, *J* = 16.0 Hz, 1H), 3.76 (dd, *J* = 11.2, 2.8 Hz, 1H), 3.45–3.35 (m, 2H), 3.01 (dd, *J* = 11.2, 4.0 Hz, 1H), 2.43 (s, 3H), 2.21 (s, 3H);

^{13}C NMR (100 MHz, $CDCl_3$): δ 143.6, 139.1, 136.9, 136.7, 136.0, 135.4, 132.7, 131.3, 129.6, 129.3, 129.2, 128.4, 127.9, 127.7, 126.9, 116.8, 49.3, 48.0, 44.6, 21.5, 21.0;

IR (KBr): 3471, 2920, 1597, 1460, 1340, 1164, 1091, 928, 803, 773, 665, 549 cm^{-1};

HRMS-ESI (m/z) Calcd for ($C_{27}H_{27}NO_2SNa$) ($[M+Na]^+$): 452.1655; found: 452.1661;

HPLC conditions: AD-H column (2% ^{i}PrOH in hexane, 1.0 mL/min, λ = 254 nm, 35 °C), t_R (major) = 19.9 min, t_R (minor) = 21.9 min;

Optical Rotation: $[\alpha]^{25}_D$ = -247.4 (*c* = 2.60, $CHCl_3$) for 93% ee;

Absolute stereochemistry was determined through analogy with **3aj**.

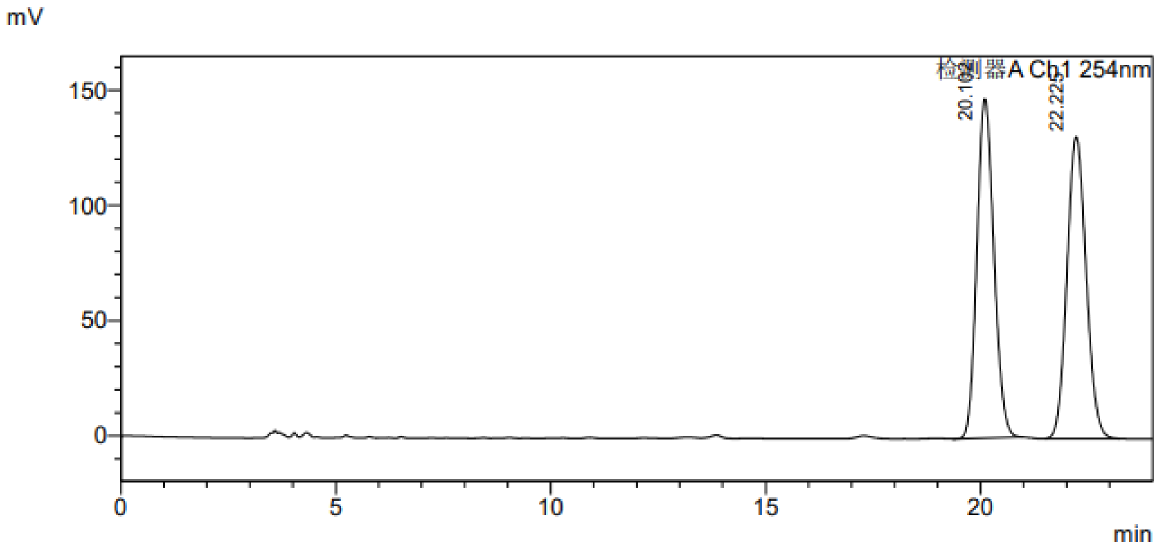

Peak#	Ret. Time	Area	Height	Conc.	Unit	Mark	Name
1	20.102	4053720	147157	49.876		M	
2	22.225	4073801	131088	50.124		M	
总计		8127521	278245	100.000			

Supplementary Figure 58. HPLC spectrum of racemic-**3ba**

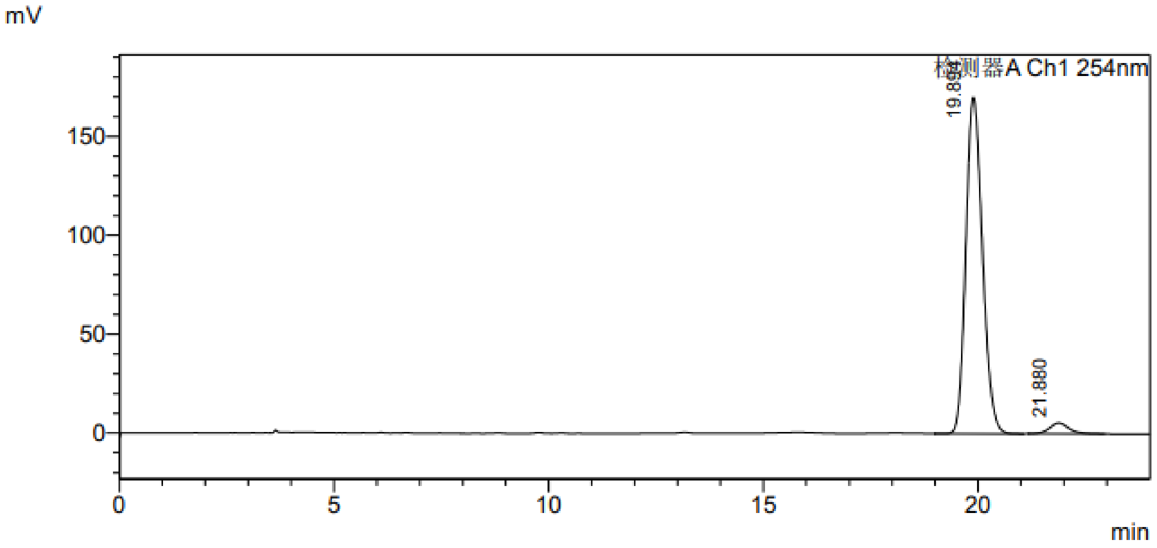

Peak#	Ret. Time	Area	Height	Conc.	Unit	Mark	Name
1	19.894	4575006	169972	96.387		M	
2	21.880	171508	5412	3.613		M	
总计		4746513	175384	100.000			

Supplementary Figure 59. HPLC spectrum of (*R*)-**3ba**

(*R*)-4-(4-methoxyphenyl)-5-phenyl-1-tosyl-3-vinyl-1,2,3,6-tetrahydropyridine (3ca)

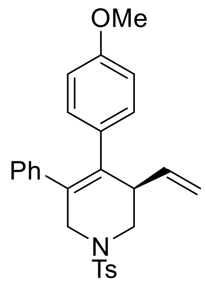

Chemical Formula: $C_{27}H_{27}NO_3S$
Exact Mass: 445.1712

3ca was prepared according to general procedure **2.1** using (*Z*)-4-((*N*-(3-(4-methoxyphenyl)prop-2-yn-1-yl)-4-methylphenyl)sulfonamido)but-2-en-1-yl acetate **1c** (0.1 mmol, 42.8 mg) and iodobenzene **2a** (0.2 mmol, 40.8 mg). Purification by silica gel column chromatography (PE/EA = 10/1) gave **3ca** as a light yellow solid (29.4 mg, 66% yield).

^{1}H NMR (400 MHz, $CDCl_3$): δ 7.70 (d, *J* = 8.0 Hz, 2H), 7.33 (d, *J* = 8.0 Hz, 2H), 7.21–7.06 (m, 3H), 7.02–6.92 (m, 2H), 6.80 (d, *J* = 8.8 Hz, 2H), 6.61 (d, *J* = 8.8 Hz, 2H), 5.85 (ddd, *J* = 17.6, 10.0, 8.0 Hz, 1H), 5.00 (d, *J* = 10.0 Hz, 1H), 4.97 (d, *J* = 17.6 Hz, 1H), 4.34 (d, *J* = 16.0 Hz, 1H), 3.74 (dd, *J* = 11.2, 2.8 Hz, 1H), 3.69 (s, 3H), 3.45–3.30 (m, 2H), 3.00 (dd, *J* = 11.2, 4.0 Hz, 1H), 2.43 (s, 3H);

^{13}C NMR (100 MHz, $CDCl_3$): δ 157.9, 143.6, 139.2, 137.0, 135.1, 132.7, 132.0, 131.1, 130.5, 129.7, 129.3, 128.0, 127.8, 126.9, 116.8, 113.0, 54.9, 49.3, 48.0, 44.7, 21.5;

IR (KBr): 3418, 2931, 1608, 1511, 1341, 1165, 995, 834, 702, 658, 548 cm^{-1};

HRMS-ESI (m/z) Calcd for ($C_{27}H_{27}NO_3SNa$) ([M+Na]$^+$): 468.1604; found: 468.1611;

HPLC conditions: OD-H column (10% ^{i}PrOH in hexane, 1.0 mL/min, λ = 254 nm, 35 °C), t_R (major) = 7.9 min, t_R (minor) = 8.4 min;

Optical Rotation: $[\alpha]^{25}_D$ = -223.4 (*c* = 0.64, $CHCl_3$) for 95% ee;

Absolute stereochemistry was determined through analogy with **3aj**.

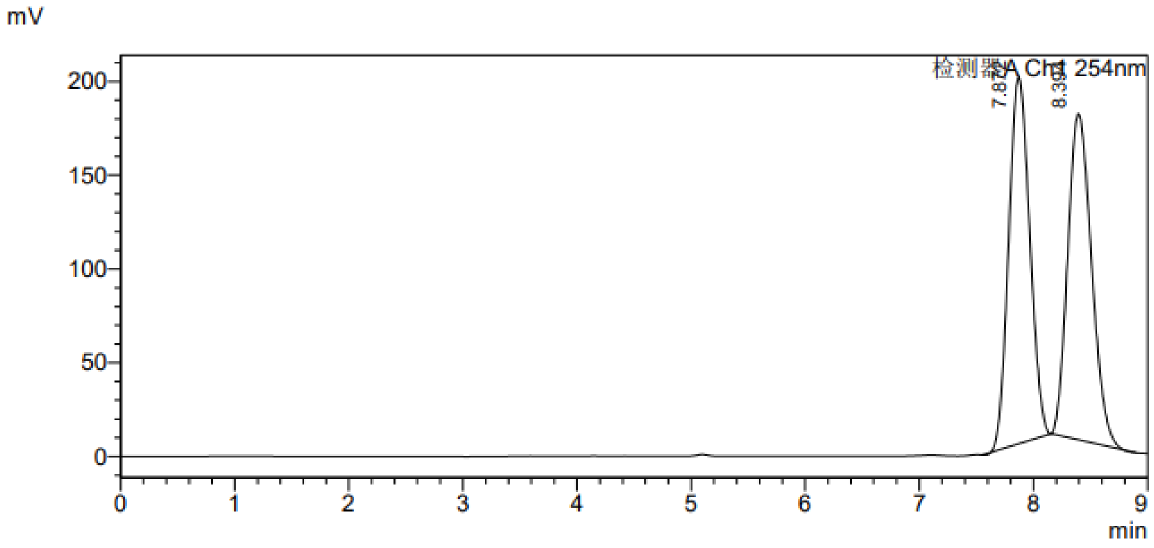

Peak#	Ret. Time	Area	Height	Conc.	Unit	Mark	Name
1	7.872	2504622	195680	49.870		M	
2	8.394	2517718	173815	50.130		M	
总计		5022340	369495	100.000			

Supplementary Figure 60. HPLC spectrum of racemic-**3ca**

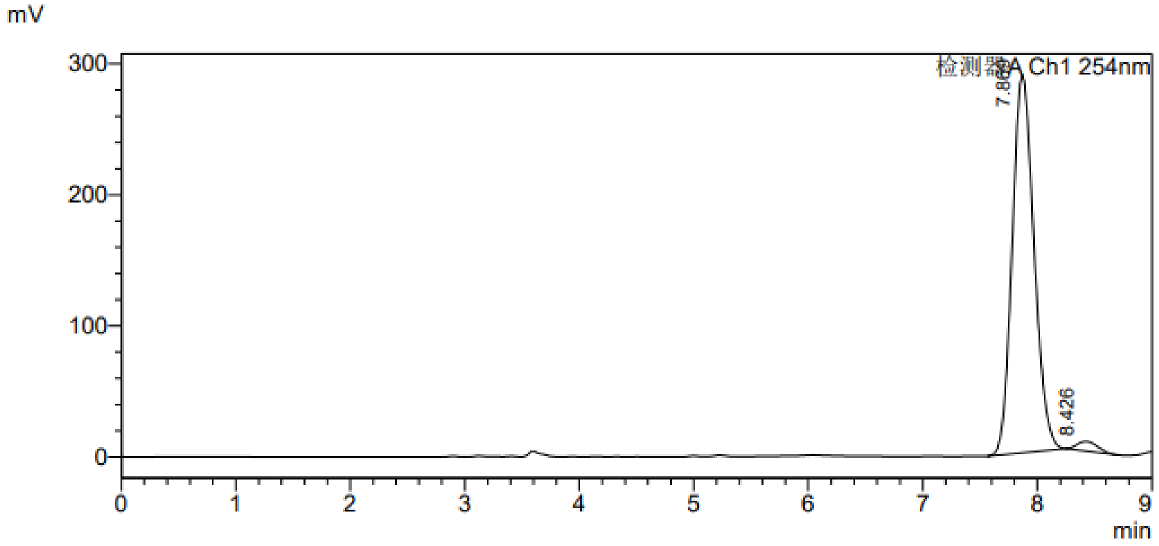

Peak#	Ret. Time	Area	Height	Conc.	Unit	Mark	Name
1	7.869	3823017	288285	97.609		M	
2	8.426	93646	7541	2.391		M	
总计		3916663	295826	100.000			

Supplementary Figure 61. HPLC spectrum of (*R*)-**3ca**

methyl (*R*)-4-(5-phenyl-1-tosyl-3-vinyl-1,2,3,6-tetrahydropyridin-4-yl)benzoate (3da)

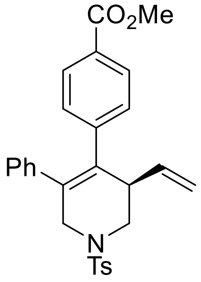

Chemical Formula: $C_{28}H_{27}NO_4S$
Exact Mass: 473.1661

3da was prepared according to general procedure **2.1** using methyl (*Z*)-4-(3-((*N*-(4-acetoxybut-2-en-1-yl)-4-methylphenyl)sulfonamido)prop-1-yn-1-yl)benzoate **1d** (0.1 mmol, 45.6 mg) and iodobenzene **2a** (0.2 mmol, 40.8 mg). Purification by silica gel column chromatography (PE/EA = 10/1) gave **3da** as a white solid (19.9 mg, 42% yield 94% ee).

^{1}H NMR (400 MHz, $CDCl_3$): δ 7.78 (d, *J* = 8.4 Hz, 2H), 7.71 (d, *J* = 8.4 Hz, 2H), 7.33 (d, *J* = 8.0 Hz, 2H), 7.19–7.05 (m, 3H), 6.96 (d, *J* = 8.4 Hz, 4H), 5.82 (ddd, *J* = 17.6, 10.0, 7.6 Hz, 1H), 4.97 (d, *J* = 10.0 Hz, 1H), 4.92 (d, *J* = 17.6 Hz, 1H), 4.35 (d, *J* = 16.4 Hz, 1H), 3.82 (s, 3H), 3.73 (dd, *J* = 11.2, 2.8 Hz, 1H), 3.50–3.35 (m, 2H), 3.04 (dd, *J* = 11.2, 4.0 Hz, 1H), 2.42 (s, 3H);

^{13}C NMR (100 MHz, $CDCl_3$): δ 166.7, 144.9, 143.7, 138.3, 136.4, 134.8, 133.0, 132.6, 129.7, 129.4, 129.1, 128.9, 128.1, 128.0, 127.7, 127.3, 117.2, 51.9, 49.2, 47.9, 44.5, 21.5;

IR (KBr): 3415, 2954, 1719, 1608, 1440, 1281, 1164, 1106, 942, 768, 664, 548 cm^{-1};

HRMS-ESI (m/z) Calcd for ($C_{28}H_{27}NO_4SNa$) ($[M+Na]^+$): 496.1553; found: 496.1557;

HPLC conditions: AS-H column (20% ^{i}PrOH in hexane, 1.0 mL/min, λ = 254 nm, 35 °C), t_R (minor) = 17.3 min, t_R (major) = 25.0 min;

Optical Rotation: $[\alpha]^{25}_D$ = -95.0 (*c* = 0.55, $CHCl_3$) for 94% ee;

Absolute stereochemistry was determined through analogy with **3aj**.

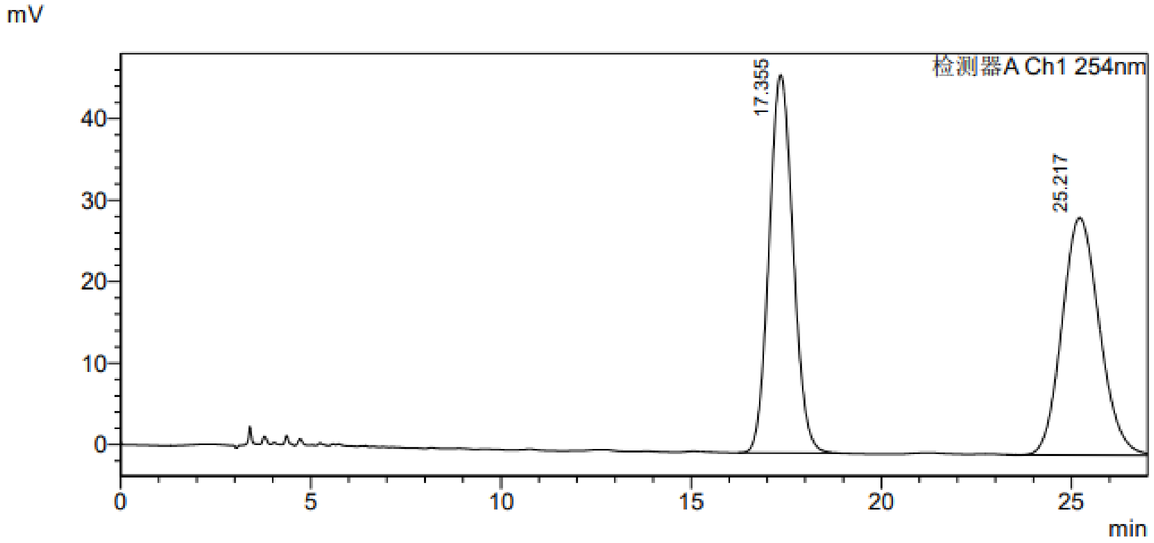

Peak#	Ret. Time	Area	Height	Conc.	Unit	Mark	Name
1	17.355	2041021	46415	50.566		M	
2	25.217	1995342	29118	49.434		M	
总计		4036363	75533	100.000			

Supplementary Figure 62. HPLC spectrum of racemic-**3da**

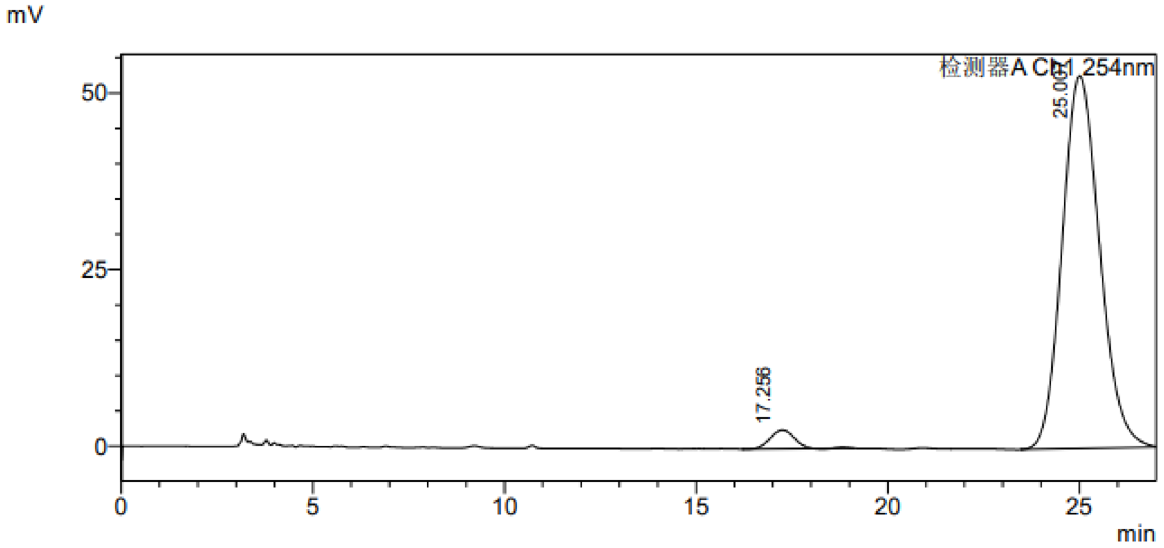

Peak#	Ret. Time	Area	Height	Conc.	Unit	Mark	Name
1	17.256	115150	2635	3.110		M	
2	25.007	3587855	52768	96.890		M	
总计		3703005	55403	100.000			

Supplementary Figure 63. HPLC spectrum of (*R*)-**3da**

(*R*)-5-phenyl-1-tosyl-4-(4-(trifluoromethyl)phenyl)-3-vinyl-1,2,3,6-tetrahydropyridine (3ea)

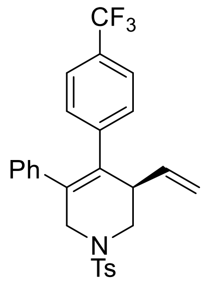

Chemical Formula: $C_{27}H_{24}F_3NO_2S$
Exact Mass: 483.1480

3ea was prepared according to general procedure **2.1** using (*Z*)-4-((4-methyl-*N*-(3-(4-(trifluoromethyl)phenyl)prop-2-yn-1-yl)phenyl)sulfonamido)but-2-en-1-yl acetate **1e** (0.2 mmol, 93.1 mg) and iodobenzene **2a** (0.4 mmol, 81.6 mg). Purification by silica gel column chromatography (PE/EA = 20/1) gave **3ea** as a white solid (30.6 mg, 32% yield).

^{1}H NMR (400 MHz, $CDCl_3$): δ 7.71 (d, *J* = 8.4 Hz, 2H), 7.38–7.30 (m, 4H), 7.22–7.06 (m, 3H), 7.08–6.83 (m, 4H), 5.83 (ddd, *J* = 17.6, 10.2, 7.8 Hz, 1H), 5.00 (d, *J* = 10.2 Hz, 1H), 4.95 (d, *J* = 17.6 Hz, 1H), 4.35 (d, *J* = 16.4 Hz, 1H), 3.74 (dd, *J* = 11.2, 2.8 Hz, 1H), 3.50–3.30 (m, 2H), 3.06 (dd, *J* = 11.2, 4.0 Hz, 1H), 2.43 (s, 3H);

^{13}C NMR (100 MHz, $CDCl_3$): δ 143.8, 143.6 (q, *J* = 1.2 Hz), 138.2, 136.3, 134.4, 133.3, 132.6, 129.73, 129.71, 129.1, 128.4 (q, *J* = 32.2 Hz), 128.1, 127.7, 127.4, 124.6 (q, *J* = 3.6 Hz), 124.0 (q, *J* = 270.1 Hz), 117.4, 49.3, 47.9, 44.6, 21.5;

^{19}F NMR (565 MHz, $CDCl_3$): δ -62.51;

IR (KBr): 3416, 2848, 1615, 1402, 1326, 1165, 993, 848, 770, 696, 546 cm^{-1};

HRMS-ESI (m/z) Calcd for ($C_{27}H_{24}F_3NO_2SNa$) ($[M+Na]^+$): 506.1372; found: 506.1379;

HPLC conditions: D-H column (2% ^{i}PrOH in hexane, 1.0 mL/min, λ = 254 nm, 35 °C), t_R (minor) =18.9 min, t_R (major) = 19.9 min;

Optical Rotation: $[\alpha]_D^{25}$ = –183.6 (*c* = 1.10, $CHCl_3$) for 96% ee;

Absolute stereochemistry was determined through analogy with **3aj**.

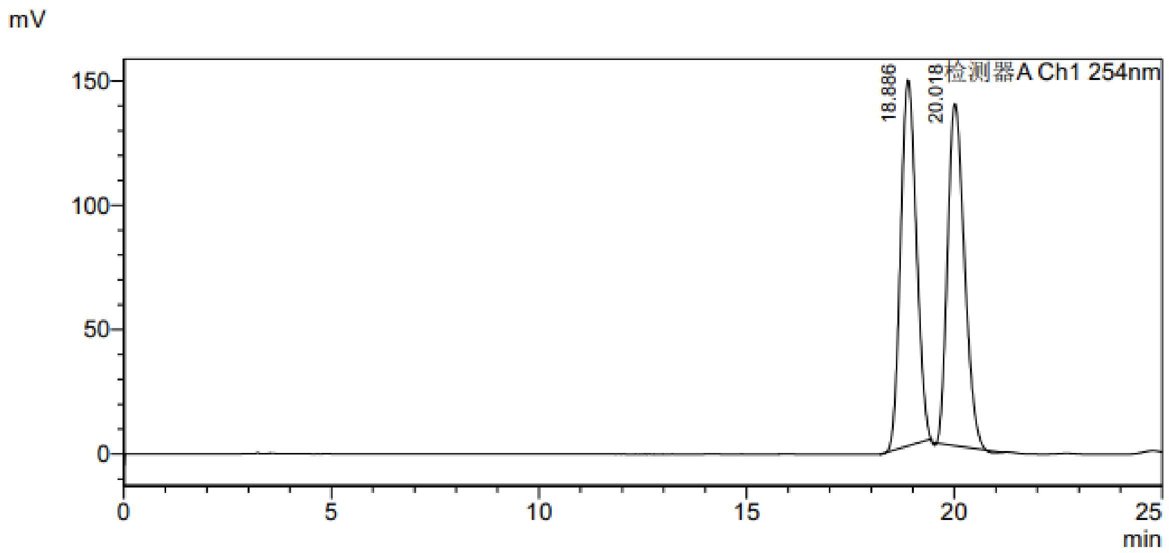

Peak#	Ret. Time	Area	Height	Conc.	Unit	Mark	Name
1	18.886	3869097	146912	49.837		M	
2	20.018	3894468	137540	50.163		M	
总计		7763565	284452	100.000			

Supplementary Figure 64. HPLC spectrum of racemic-**3ea**

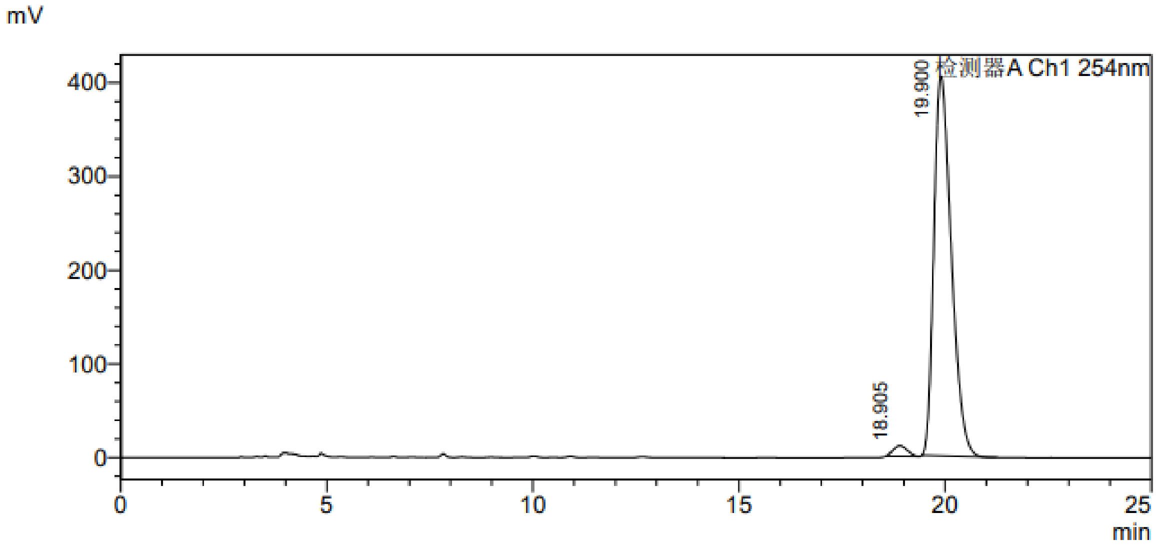

Peak#	Ret. Time	Area	Height	Conc.	Unit	Mark	Name
1	18.905	266656	11283	2.185		M	
2	19.900	11938649	405037	97.815		M	
总计		12205305	416319	100.000			

Supplementary Figure 65. HPLC spectrum of (*R*)-**3ea**

(*R*)-4-(4-fluorophenyl)-5-phenyl-1-tosyl-3-vinyl-1,2,3,6-tetrahydropyridine (3fa)

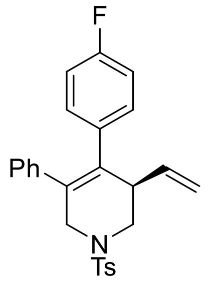

Chemical Formula: $C_{26}H_{24}FNO_2S$
Exact Mass: 433.1512

3fa was prepared according to general procedure **2.1** using (*Z*)-4-((*N*-(3-(4-fluorophenyl)prop-2-yn-1-yl)-4-methylphenyl)sulfonamido)but-2-en-1-yl acetate **1f** (0.1 mmol, 41.5 mg) and iodobenzene **2a** (0.2 mmol, 40.8 mg). Purification by silica gel column chromatography (PE/EA = 20/1) gave **3fa** as a light yellow solid (26.0 mg, 60% yield).

^{1}H NMR (400 MHz, $CDCl_3$): δ 7.70 (d, *J* = 8.4 Hz, 2H), 7.33 (d, *J* = 8.0 Hz, 2H), 7.16–7.02 (m, 3H), 6.99–6.90 (m, 2H), 6.88–6.80 (m, 2H), 6.80–6.71 (m, 2H), 5.83 (ddd, *J* = 17.2, 10.2, 8.0 Hz, 1H), 5.00 (d, *J* = 10.2 Hz, 1H), 4.94 (d, *J* = 17.2 Hz, 1H), 4.33 (d, *J* = 16.4 Hz, 1H), 3.72 (dd, *J* = 11.2, 2.8 Hz, 1H), 3.49–3.26 (m, 2H), 3.02 (dd, *J* = 11.2, 4.0 Hz, 1H), 2.43 (s, 3H);

^{13}C NMR (100 MHz, $CDCl_3$): δ 161.3 (d, *J* = 244.4 Hz), 143.7, 138.7, 136.7, 135.7 (d, *J* = 3.4 Hz), 134.6, 132.8, 132.2, 131.0 (d, *J* = 7.8 Hz), 129.7, 129.2, 128.1, 127.8, 127.1, 117.1, 114.6 (d, *J* = 21.2 Hz), 49.3, 47.9, 44.9, 21.5;

^{19}F NMR (565 MHz, $CDCl_3$): δ -115.48 – -115.55 (m);

IR (KBr): 3459, 1509, 1342, 1227, 1161, 773, 703, 666, 549 cm^{-1};

HRMS-ESI (m/z) Calcd for ($C_{26}H_{24}FNO_2SNa$) ($[M+Na]^+$): 456.1404; found: 456.1406;

HPLC conditions: AD-H column (2% ^{i}PrOH in hexane, 1.0 mL/min, λ = 254 nm, 35 °C), t_R (minor) = 22.2 min, t_R (major) = 23.4 min;

Optical Rotation: $[\alpha]^{25}_D$ = -214.4 (*c* = 2.15, $CHCl_3$) for 90% ee;

Absolute stereochemistry was determined through analogy with **3aj**.

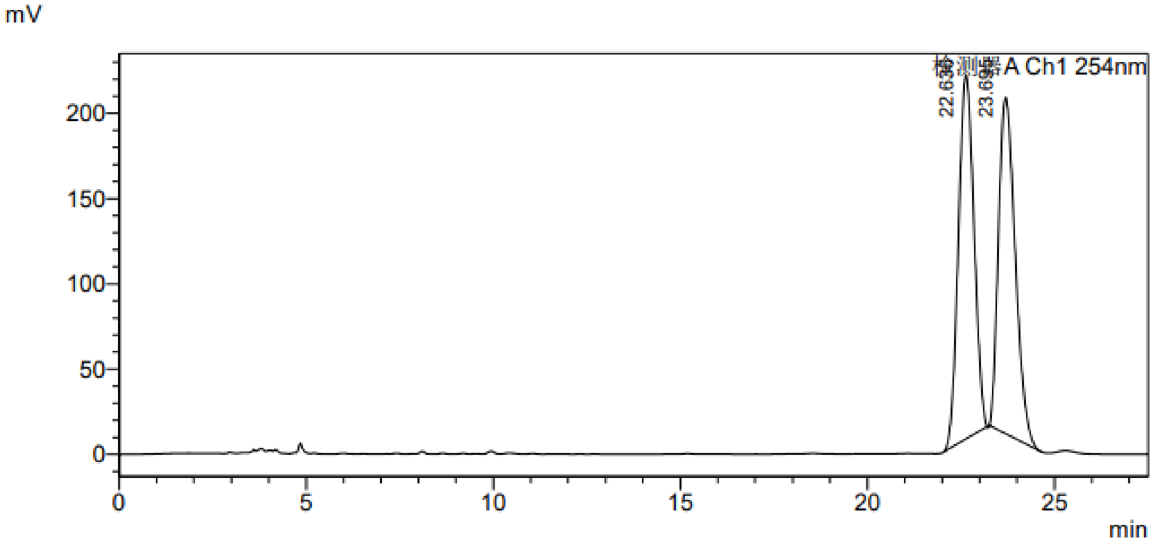

Peak#	Ret. Time	Area	Height	Conc.	Unit	Mark	Name
1	22.636	6179456	213445	50.198		M	
2	23.695	6130728	197207	49.802		M	
总计		12310184	410651	100.000			

Supplementary Figure 66. HPLC spectrum of racemic-**3fa**

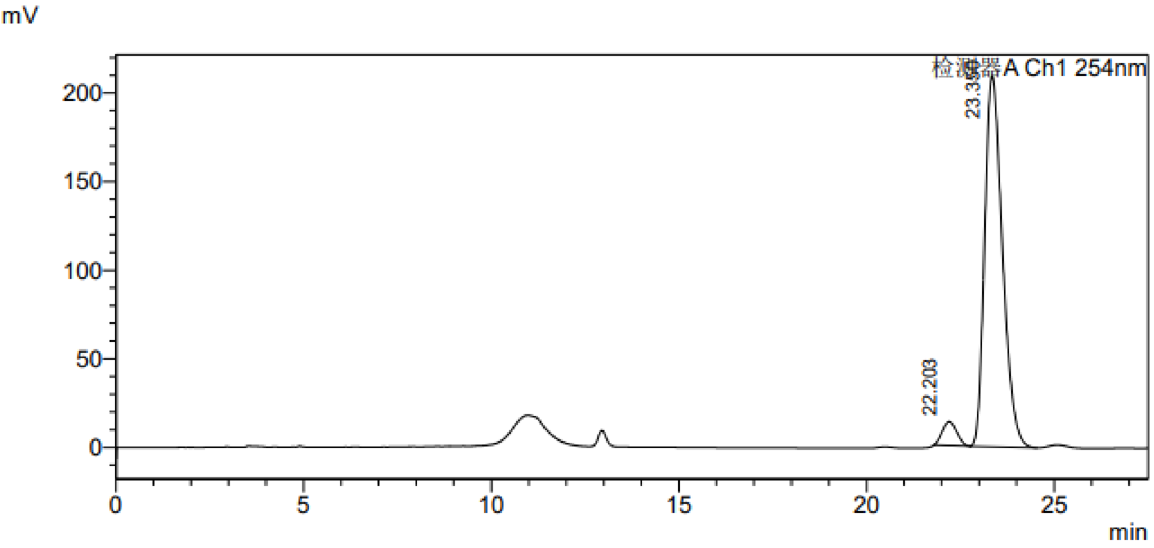

Peak#	Ret. Time	Area	Height	Conc.	Unit	Mark	Name
1	22.203	359495	13386	4.957		M	
2	23.350	6893211	209122	95.043		M	
总计		7252706	222508	100.000			

Supplementary Figure 67. HPLC spectrum of (*R*)-**3fa**

(*R*)-5-phenyl-4-(4-(3,3,4,4-tetramethylborolan-1-yl)phenyl)-1-tosyl-3-vinyl-1,2,3,6-tetrahydropyridine (3ga)

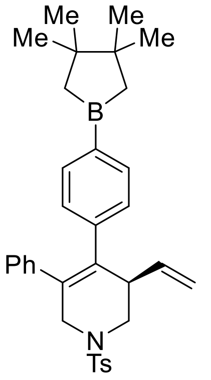

Chemical Formula: $C_{34}H_{40}BNO_2S$
Exact Mass: 537.2873

3ga was prepared according to general procedure **2.1** using (*Z*)-4-((4-methyl-*N*-(3-(4-(3,3,4,4-tetramethylborolan-1-yl)phenyl)prop-2-yn-1-yl)phenyl)sulfonamido)but-2-en-1-yl acetate **1g** (0.2 mmol, 103.9 mg) and iodobenzene **2a** (0.4 mmol, 81.6 mg). Purification by silica gel column chromatography (PE/EA = 20/1) gave **3ga** as a white solid (50.3 mg, 46% yield).

^{1}H NMR (400 MHz, $CDCl_3$): δ 7.70 (d, *J* = 8.4 Hz, 2H), 7.52 (d, *J* = 8.0 Hz, 2H), 7.33 (d, *J* = 8.0 Hz, 2H), 7.16–7.06 (m, 3H), 7.02–6.94 (m, 2H), 6.89 (d, *J* = 8.0 Hz, 2H), 5.82 (ddd, *J* = 17.6, 10.0, 8.0 Hz, 1H), 4.97 (d, *J* = 10.0 Hz, 1H), 4.95 (d, *J* = 17.6 Hz, 1H), 4.35 (d, *J* = 16.0 Hz, 1H), 3.74 (dd, J = 11.2, 2.8 Hz, 1H), 3.52–3.27 (m, 2H), 3.03 (dd, *J* = 11.2, 4.0 Hz, 1H), 2.43 (s, 3H), 1.29 (s, 12H);

^{13}C NMR (100 MHz, $CDCl_3$): δ 143.6, 142.8, 138.7, 136.6, 135.5, 134.0, 132.7, 131.9, 129.6, 129.2, 128.7, 127.9, 127.7, 127.0, 116.9, 83.6, 49.3, 47.9, 44.5, 24.8, 24.7, 21.4;

IR (KBr): 3461, 2978, 1635, 1609, 1399, 1360, 1165, 770, 546 cm^{-1};

HRMS-ESI (m/z) Calcd for ($C_{32}H_{36}BNO_4SNa$) ($[M+Na]^+$): 564.2350; found: 564.2359;

HPLC conditions: OD-H column (2% ^{i}PrOH in hexane, 0.5 mL/min, λ = 254 nm, 35 °C), t_R (minor) = 22.3 min, t_R (major) = 23.4 min;

Optical Rotation: $[\alpha]^{25}_D$ = -210.6 (*c* = 2.14, $CHCl_3$) for 93% ee;

Absolute stereochemistry was determined through analogy with **3aj**.

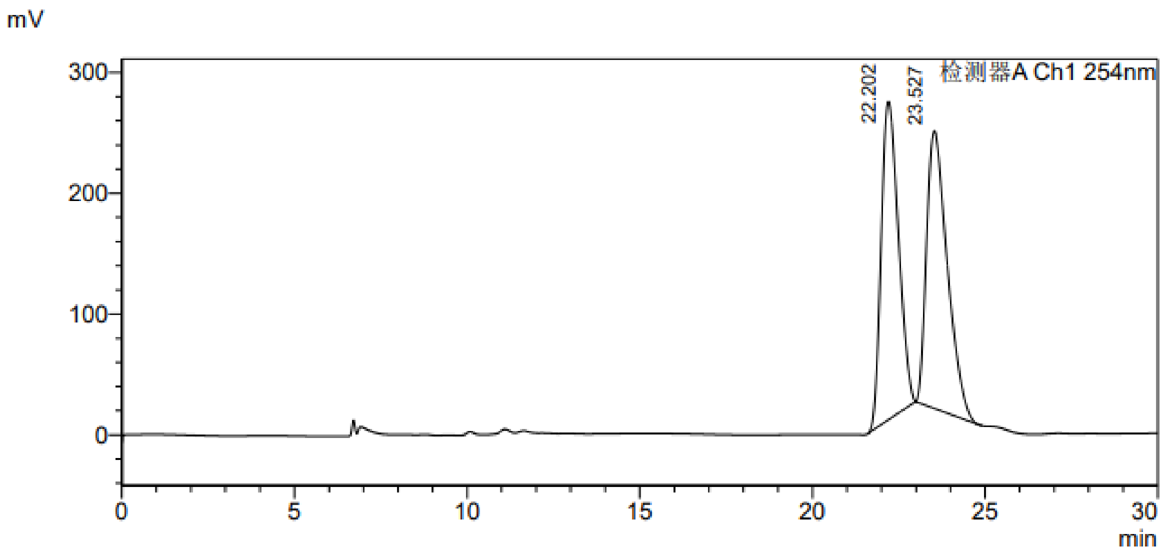

Peak#	Ret. Time	Area	Height	Conc.	Unit	Mark	Name
1	22.202	9102195	263313	48.479		M	
2	23.527	9673326	229743	51.521		M	
总计		18775521	493056	100.000			

Supplementary Figure 68. HPLC spectrum of racemic-**3ga**

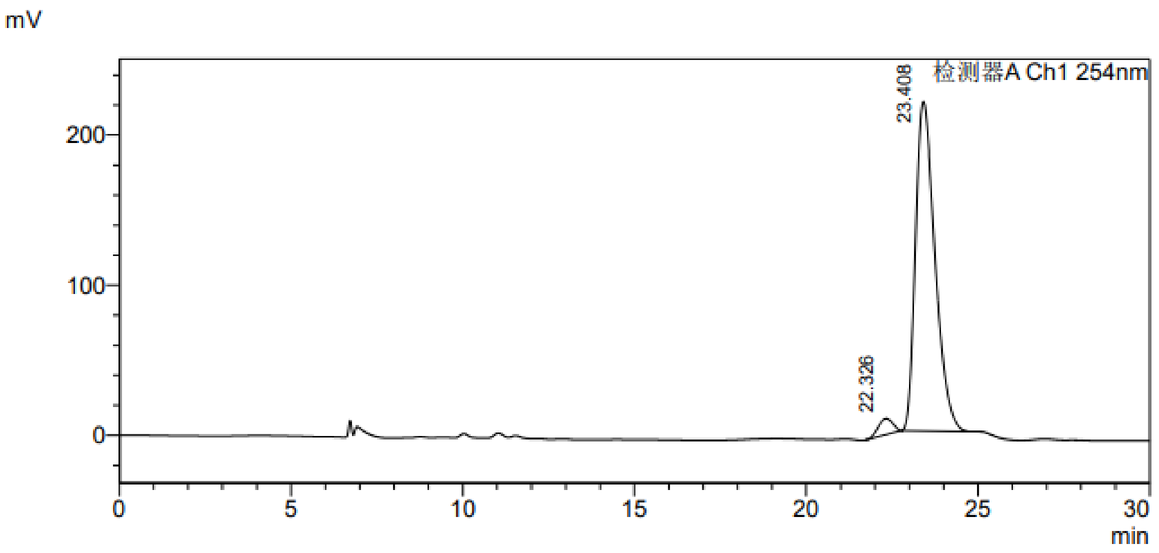

Peak#	Ret. Time	Area	Height	Conc.	Unit	Mark	Name
1	22.326	315930	10676	3.510		M	
2	23.408	8685702	219264	96.490		M	
总计		9001631	229940	100.000			

Supplementary Figure 69. HPLC spectrum of (*R*)-**3ga**

(*R*)-5-phenyl-4-(m-tolyl)-1-tosyl-3-vinyl-1,2,3,6-tetrahydropyridine (3ha)

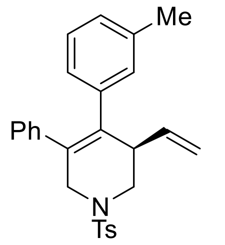

Chemical Formula: $C_{27}H_{27}NO_2S$
Exact Mass: 429.1762

3ha was prepared according to general procedure **2.1** using (*Z*)-4-((4-methyl-*N*-(3-(*m*-tolyl)prop-2-yn-1-yl)phenyl)sulfonamido)but-2-en-1-yl acetate **1h** (0.2 mmol, 82.3 mg) and iodobenzene **2a** (0.4 mmol, 81.6 mg). Purification by silica gel column chromatography (PE/EA = 20/1) gave **3ha** as a light yellow solid (56.6 mg, 66% yield).

^{1}H NMR (400 MHz, $CDCl_3$): δ 7.71 (d, *J* = 8.0 Hz, 2H), 7.34 (d, *J* = 8.0 Hz, 2H), 7.19–7.07 (m, 3H), 7.04–6.91 (m, 3H), 6.86 (d, *J* = 7.6 Hz, 1H), 6.71 (s, 1H), 6.67 (d, *J* = 7.6 Hz, 1H), 5.85 (ddd, *J* = 17.6, 10.0, 7.6 Hz, 1H), 5.01 (d, *J* = 10.0 Hz, 1H), 4.98 (d, *J* = 17.6 Hz, 1H), 4.36 (d, *J* = 16.0 Hz, 1H), 3.76 (dd, *J* = 11.2, 2.8 Hz, 1H), 3.54–3.27 (m, 2H), 3.02 (dd, *J* = 11.2, 4.0 Hz, 1H), 2.44 (s, 3H), 2.15 (s, 3H);

^{13}C NMR (100 MHz, $CDCl_3$): δ 143.6, 139.6, 139.0, 137.0, 136.8, 135.6, 132.7, 131.4, 129.9, 129.7, 129.2, 127.8, 127.7, 127.4, 127.2, 126.9, 126.5, 116.8, 49.3, 47.9, 44.5, 21.5, 21.3;

IR (KBr): 3451, 1636, 1341, 1166, 776, 704, 551 cm^{-1};

HRMS-ESI (m/z) Calcd for ($C_{27}H_{27}NO_2SNa$) ($[M+Na]^+$): 452.1655; found: 452.1655;

HPLC conditions: AD-H column (10% ^{i}PrOH in hexane, 0.5 mL/min, λ = 254 nm, 35 °C), t_R (major) = 13.4 min, t_R (minor) = 14.3 min;

Optical Rotation: $[\alpha]^{25}_D$ = -262.2 (*c* = 0.93, $CHCl_3$) for 92% ee;

Absolute stereochemistry was determined through analogy with **3aj**.

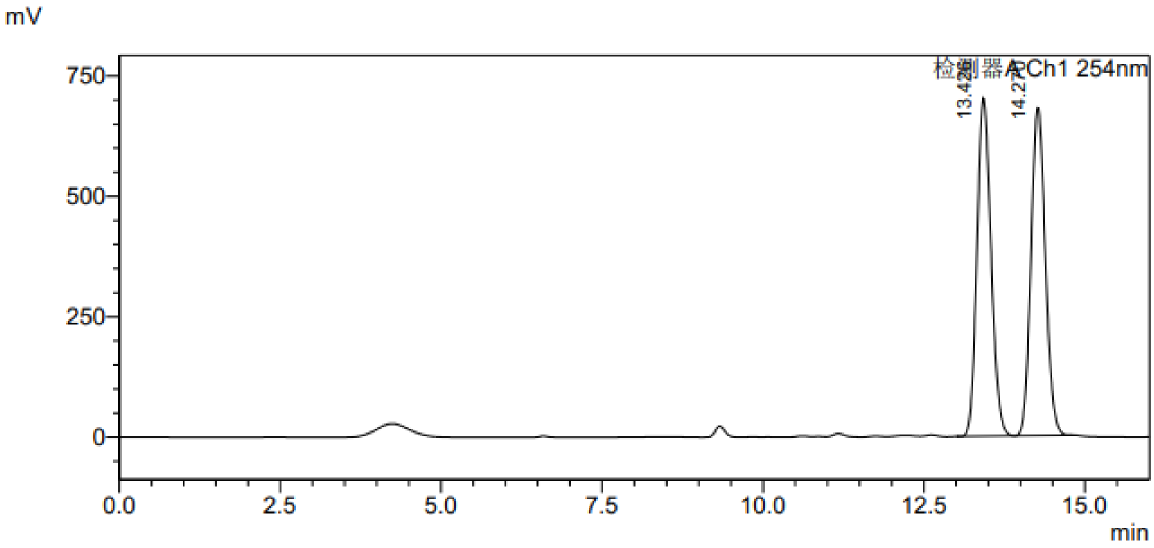

Peak#	Ret. Time	Area	Height	Conc.	Unit	Mark	Name
1	13.426	10626065	701690	50.037		M	
2	14.270	10610264	680874	49.963		M	
总计		21236329	1382564	100.000			

Supplementary Figure 70. HPLC spectrum of racemic-**3ha**

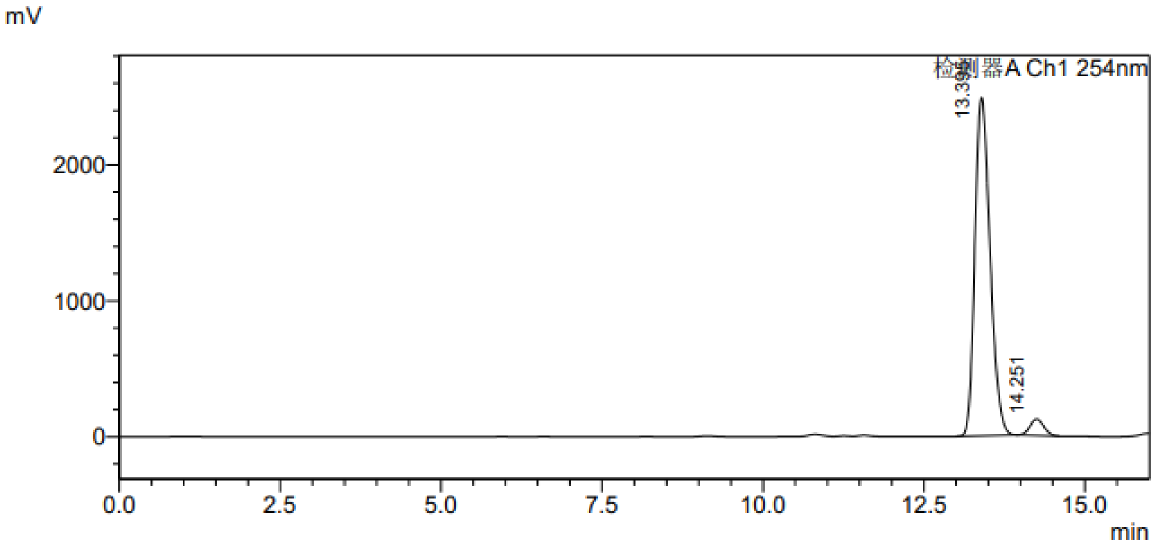

Peak#	Ret. Time	Area	Height	Conc.	Unit	Mark	Name
1	13.395	40734901	2486695	95.791		M	
2	14.251	1789929	119103	4.209		M	
总计		42524829	2605798	100.000			

Supplementary Figure 71. HPLC spectrum of (*R*)-**3ha**

(*R*)-4-(3-chlorophenyl)-5-phenyl-1-tosyl-3-vinyl-1,2,3,6-tetrahydropyridine (3ia)

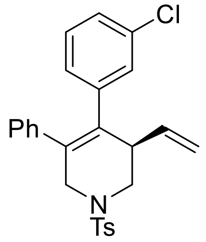

Chemical Formula: $C_{26}H_{24}ClNO_2S$
Exact Mass: 449.1216

3ia was prepared according to general procedure **2.1** using (*Z*)-4-((*N*-(3-(3-chlorophenyl)prop-2-yn-1-yl)-4-methylphenyl)sulfonamido)but-2-en-1-yl acetate **1i** (0.2 mmol, 86.4 mg) and iodobenzene **2a** (0.4 mmol, 81.6 mg). Purification by silica gel column chromatography (PE/EA = 20/1) gave **3ia** as a white solid (46.0 mg, 51% yield).

^{1}H NMR (400 MHz, $CDCl_3$): δ 7.70 (d, *J* = 8.0 Hz, 2H), 7.34 (d, *J* = 8.0 Hz, 2H), 7.20–7.09 (m, 3H), 7.07–6.93 (m, 4H), 6.90 (s, 1H), 6.72 (d, *J* = 7.6 Hz, 1H), 5.82 (ddd, *J* = 17.6, 10.0, 8.0 Hz, 1H), 5.00 (d, *J* = 10.0 Hz, 1H), 4.96 (d, *J* = 17.6 Hz, 1H), 4.33 (d, *J* = 16.4 Hz, 1H), 3.72 (dd, *J* = 11.2, 2.8 Hz, 1H), 3.42 (d, *J* = 16.4 Hz, 1H), 3.37–3.27 (m, 1H), 3.03 (dd, *J* = 11.2, 4.0 Hz, 1H), 2.44 (s, 3H);

^{13}C NMR (100 MHz, $CDCl_3$): δ 143.7, 141.7, 138.4, 136.5, 134.4, 133.5, 132.9, 132.7, 129.7, 129.1, 128.9, 128.1, 127.9, 127.7, 127.3, 126.6, 117.3, 49.3, 47.9, 44.6, 21.5;

IR (KBr): 3418, 1596, 1445, 1346, 1163, 1097, 922, 771, 704, 666, 550 cm^{-1};

HRMS-ESI (m/z) Calcd for ($C_{26}H_{24}ClNO_2SNa$) ($[M+Na]^+$): 472.1108; found: 472.1114;

HPLC conditions: AS-H column (10% ^{i}PrOH in hexane, 1.0 mL/min, λ = 254 nm, 35 °C), t_R (minor) = 16.8 min, t_R (major) = 21.4 min;

Optical Rotation: $[\alpha]^{25}_D$ = -239.8 (*c* =1.83, $CHCl_3$) for 94% ee;

Absolute stereochemistry was determined through analogy with **3aj**.

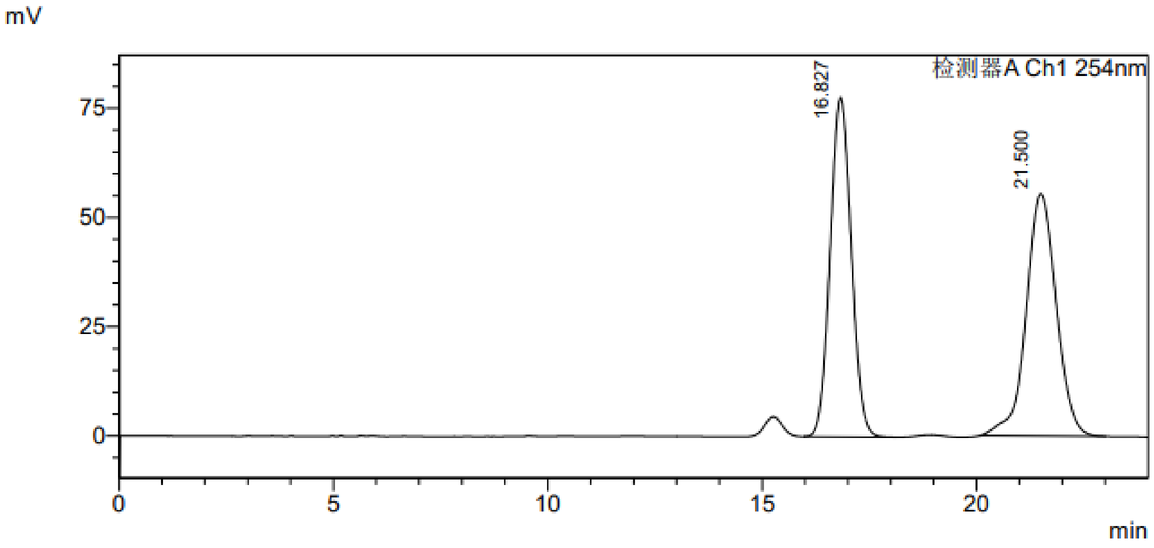

Peak#	Ret. Time	Area	Height	Conc.	Unit	Mark	Name
1	16.827	2669343	77681	49.527		M	
2	21.500	2720283	55426	50.473		M	
总计		5389626	133108	100.000			

Supplementary Figure 72. HPLC spectrum of racemic-**3ia**

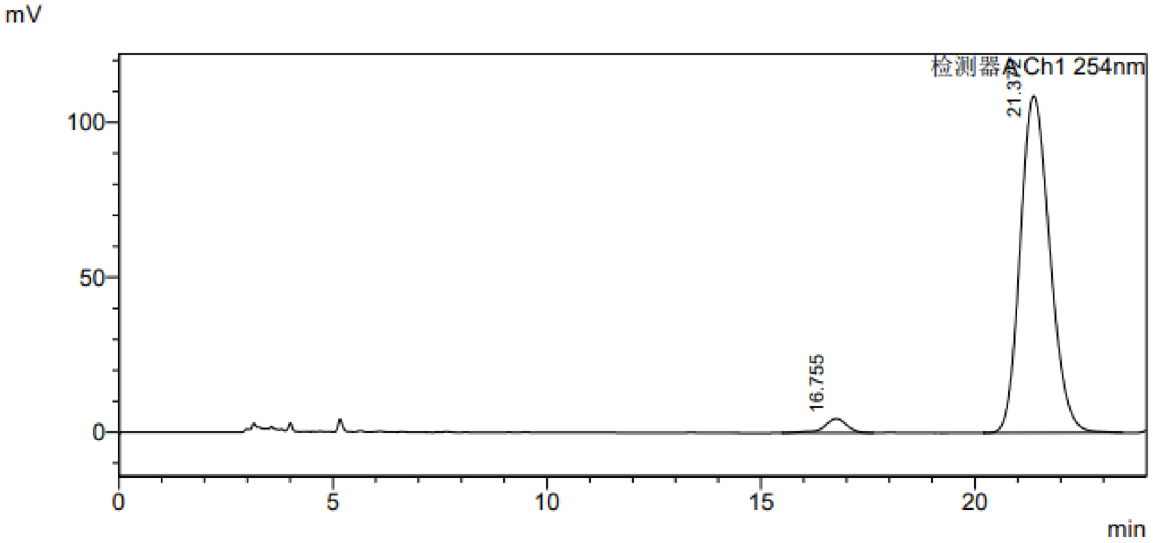

Peak#	Ret. Time	Area	Height	Conc.	Unit	Mark	Name
1	16.755	166375	4551	3.084		M	
2	21.372	5228575	108683	96.916		M	
总计		5394950	113233	100.000			

Supplementary Figure 73. HPLC spectrum of (*R*)-**3ia**

(*R*)-4-(benzo[d][1,3]dioxol-5-yl)-5-phenyl-1-tosyl-3-vinyl-1,2,3,6-tetrahydropyridine (3ja)

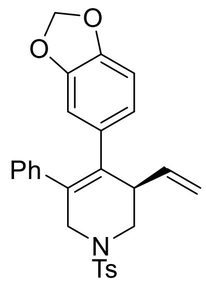

Chemical Formula: $C_{27}H_{25}NO_4S$
Exact Mass: 459.1504

3ja was prepared according to general procedure **2.1** using (*Z*)-4-((*N*-(3-(benzo[*d*][1,3]dioxol-5-yl)prop-2-yn-1-yl)-4-methylphenyl)sulfonamido)but-2-en-1-yl acetate **1j** (0.2 mmol, 88.3 mg) and iodobenzene **2a** (0.4 mmol, 81.6 mg). Purification by silica gel column chromatography (PE/EA = 10/1) gave **3ja** as a white solid (42.1 mg, 46% yield).

^{1}H NMR (400 MHz, $CDCl_3$): δ 7.69 (d, *J* = 8.4 Hz, 2H), 7.32 (d, *J* = 8.0 Hz, 2H), 7.20–7.08 (m, 3H), 7.04–6.93 (m, 2H), 6.52 (d, *J* = 8.0 Hz, 1H), 6.43–6.26 (m, 2H), 5.93–5.73 (m, 3H), 5.11–4.87 (m, 2H), 4.32 (d, *J* = 16.4 Hz, 1H), 3.72 (dd, *J* = 11.2, 2.8 Hz, 1H), 3.38 (d, *J* = 16.4, 2.0 Hz, 1H), 3.34–3.25 (m, 1H), 2.99 (dd, *J* = 11.2, 4.0 Hz, 1H), 2.43 (s, 3H);

^{13}C NMR (100 MHz, $CDCl_3$): δ 146.9, 145.9, 143.6, 139.0, 136.9, 135.2, 133.7, 132.8, 131.6, 129.7, 129.2, 128.1, 127.8, 127.0, 123.0, 116.9, 109.7, 107.7, 100.7, 49.3, 48.0, 44.9, 21.5;

IR (KBr): 3421, 2888, 1598, 1489, 1343, 1341, 1166, 1039, 934, 814, 701, 550 cm^{-1};

HRMS-ESI (m/z) Calcd for ($C_{27}H_{25}NO_4SNa$) ($[M+Na]^+$): 482.1397; found: 482.1403;

HPLC conditions: AD-H column (10% ^{i}PrOH in hexane, 0.5 mL/min, λ = 254 nm, 35 °C), t_R (major) = 26.4 min, t_R (minor) = 27.6 min;

Optical Rotation: $[\alpha]^{25}_D$ = -208.8 (*c* = 1.90, $CHCl_3$) for 93% ee;

Absolute stereochemistry was determined through analogy with **3aj**.

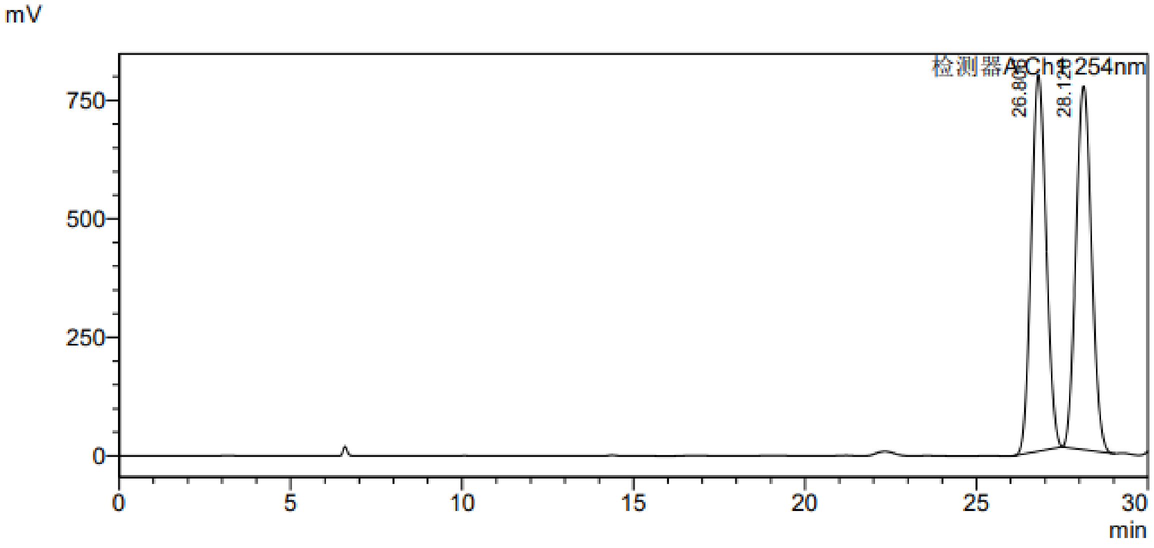

Peak#	Ret. Time	Area	Height	Conc.	Unit	Mark	Name
1	26.806	23957052	793818	49.508		M	
2	28.126	24433105	767412	50.492		M	
总计		48390157	1561230	100.000			

Supplementary Figure 74. HPLC spectrum of racemic-**3ja**

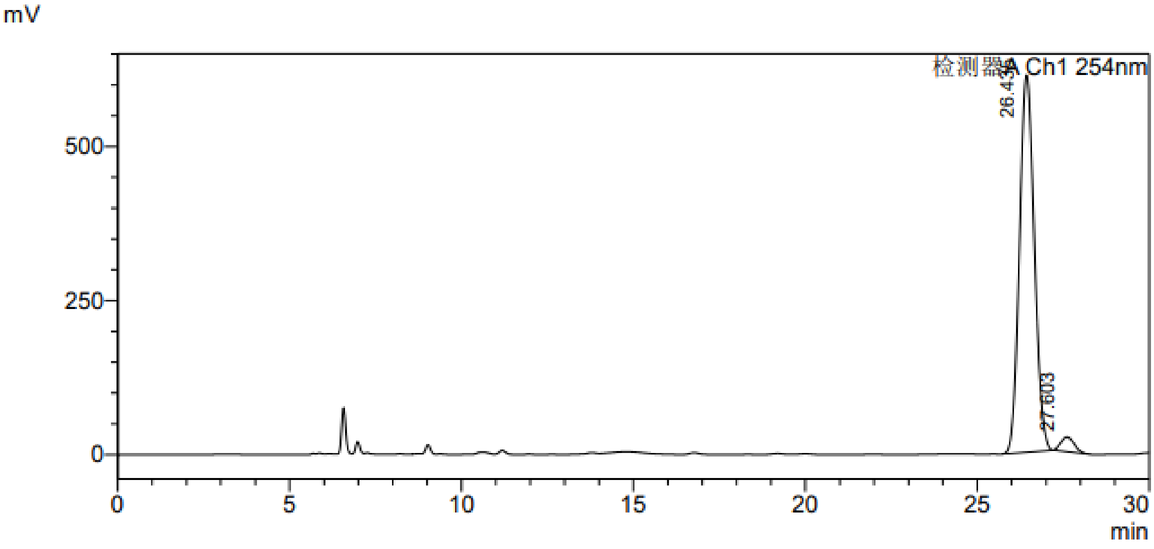

Peak#	Ret. Time	Area	Height	Conc.	Unit	Mark	Name
1	26.435	18278352	611821	96.650		M	
2	27.603	633616	23343	3.350		M	
总计		18911967	635164	100.000			

Supplementary Figure 75. HPLC spectrum of (*R*)-**3ja**

(*R*)-5-phenyl-4-(thiophen-2-yl)-1-tosyl-3-vinyl-1,2,3,6-tetrahydropyridine (3ka)

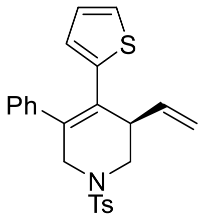

Chemical Formula: $C_{24}H_{23}NO_2S_2$
Exact Mass: 421.1170

3ka was prepared according to general procedure **2.1** using (*Z*)-4-((4-methyl-*N*-(3-(thiophen-2-yl)prop-2-yn-1-yl)phenyl)sulfonamido)but-2-en-1-yl acetate **1k** (0.2 mmol, 80.7 mg) and iodobenzene **2a** (0.4 mmol, 81.6 mg). Purification by silica gel column chromatography (PE/EA = 20/1) gave **3ka** as a white solid (64.7 mg, 77% yield).

^{1}H NMR (400 MHz, $CDCl_3$): δ 7.70 (d, *J* = 8.4 Hz, 2H), 7.34 (d, *J* = 8.0 Hz, 2H), 7.32–7.23 (m, 3H), 7.20–7.10 (m, 2H), 7.03 (dd, *J* = 5.2, 1.2 Hz, 1H), 6.76 (dd, *J* = 5.2, 3.6 Hz, 1H), 6.65 (dd, J = 3.6, 0.8 Hz, 1H), 6.02 (ddd, *J* = 17.2, 10.0, 7.6 Hz, 1H), 5.21 (d, *J* = 10.0 Hz, 1H), 5.16 (d, *J* = 17.2 Hz, 1H), 4.34 (d, *J* = 16.8 Hz, 1H), 3.86 (dd, *J* = 11.2, 2.0 Hz, 1H), 3.53–3.43 (m, 1H), 3.38 (dd, *J* = 16.8, 2.0 Hz, 1H), 2.95 (dd, *J* = 11.2, 4.0 Hz, 1H), 2.44 (s, 3H);

^{13}C NMR (100 MHz, $CDCl_3$): δ 143.6, 141.6, 139.0, 137.2, 132.6, 132.5, 129.6, 129.1, 128.5, 127.9, 127.7, 127.6, 126.7, 126.1, 125.1, 117.3, 49.9, 47.9, 44.5, 21.5;

IR (KBr): 3459, 2850, 2360, 1637, 1342, 1170, 771, 551 cm^{-1};

HRMS-ESI (m/z) Calcd for ($C_{24}H_{23}NO_2S_2Na$) ($[M+Na]^+$): 444.1062; found: 444.1065;

HPLC conditions: AD-H column (10% ^{i}PrOH in hexane, 0.5 mL/min, λ = 254 nm, 35 °C), t_R (major) = 19.4 min, t_R (minor) = 20.5 min;

Optical Rotation: $[\alpha]^{25}_D$ = -142.4 (*c* = 0.63, $CHCl_3$) for 96% ee;

Absolute stereochemistry was determined through analogy with **3aj**.

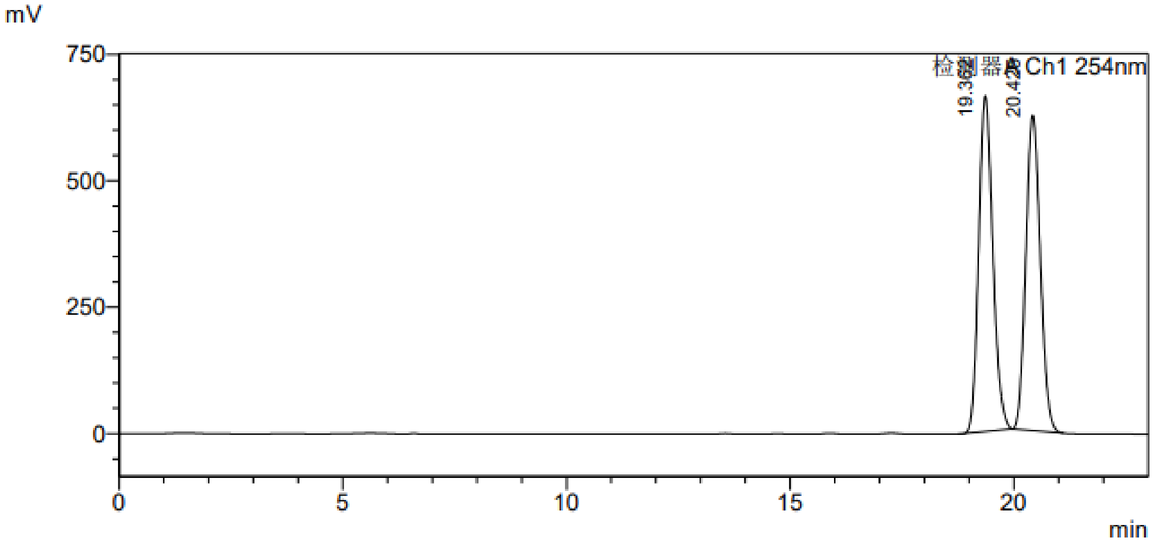

Peak#	Ret. Time	Area	Height	Conc.	Unit	Mark	Name
1	19.362	14512128	663355	50.866		M	
2	20.425	14018157	622807	49.134		M	
总计		28530285	1286162	100.000			

Supplementary Figure 76. HPLC spectrum of racemic-**3ka**

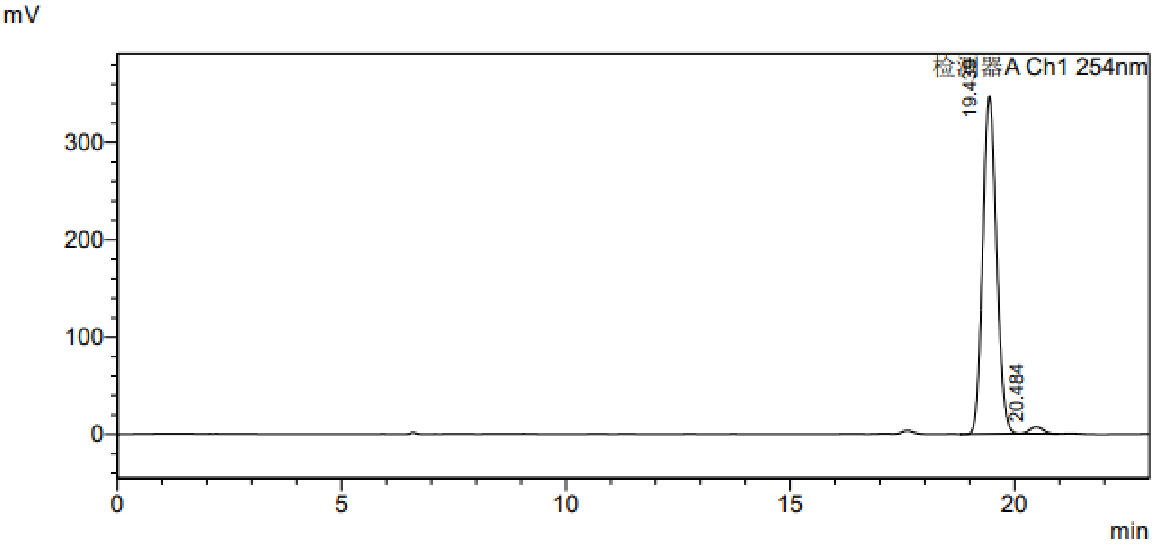

Peak#	Ret. Time	Area	Height	Conc.	Unit	Mark	Name
1	19.439	7444803	347039	98.102		M	
2	20.484	144042	7100	1.898		M	
总计		7588845	354140	100.000			

Supplementary Figure 77. HPLC spectrum of (*R*)-**3ka**

tert-butyl (_R_)-4,5-diphenyl-3-vinyl-3,6-dihydropyridine-1(2_H_)-carboxylate (3la)

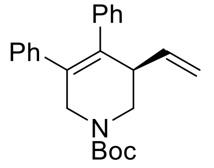

Chemical Formula: $C_{24}H_{27}NO_2$
Exact Mass: 361.2042

3la was prepared according to general procedure **2.1** using (*Z*)-4-((*tert*-butoxycarbonyl)(3-phenylprop-2-yn-1-yl)amino)but-2-en-1-yl acetate **1l** (0.1 mmol, 34.3 mg) and iodobenzene **2a** (0.2 mmol, 40.8 mg). Purification by silica gel column chromatography (PE/EA = 20/1) gave **3la** as a yellow oil (15.9 mg, 41% yield).

^{1}H NMR (400 MHz, $CDCl_3$): δ 7.18–7.02 (m, 8H), 7.01–6.94 (m, 2H), 5.90–5.70 (m, 1H), 5.10–4.90 (m, 2H), 4.90–4.40 (m, 1H), 4.20–4.10 (m, 1H), 4.00–3.70 (m, 1H), 3.45–3.25 (m, 2H), 1.50 (s, 9H);

^{13}C NMR (125 MHz, $CDCl_3$, 50 °C): δ 155.0, 140.9, 137.3, 129.4, 129.3, 127.9, 127.6, 126.7, 126.2, 116.1, 79.7, 44.9, 28.5;

IR (neat): 3459, 3060, 2976, 2930, 2252, 1692, 1605, 1365, 1250, 1164, 913, 699 cm^{-1};

HRMS-ESI (m/z) Calcd for ($C_{24}H_{27}NO_2Na$) ($[M+Na]^+$): 384.1934; found: 384.1933;

HPLC conditions: AD-H column (5% ^{i}PrOH in hexane, 1.0 mL/min, λ = 254 nm, 35 °C), t_R (minor) = 4.9 min, t_R (major) = 5.6 min;

Optical Rotation: $[\alpha]^{25}_D$ = -50.6 (*c* = 0.64, $CHCl_3$) for 95% ee;

Absolute stereochemistry was determined through analogy with **3aj**.

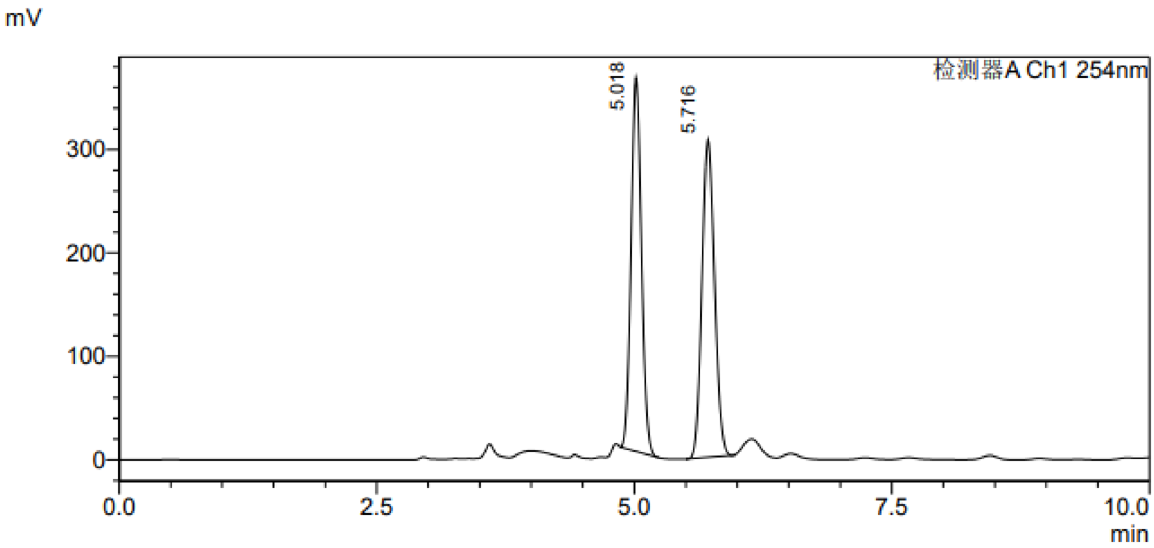

Peak#	Ret. Time	Area	Height	Conc.	Unit	Mark	Name
1	5.018	2568492	360821	49.303		M	
2	5.716	2641143	307566	50.697		M	
总计		5209635	668388	100.000			

Supplementary Figure 78. HPLC spectrum of racemic-**3la**

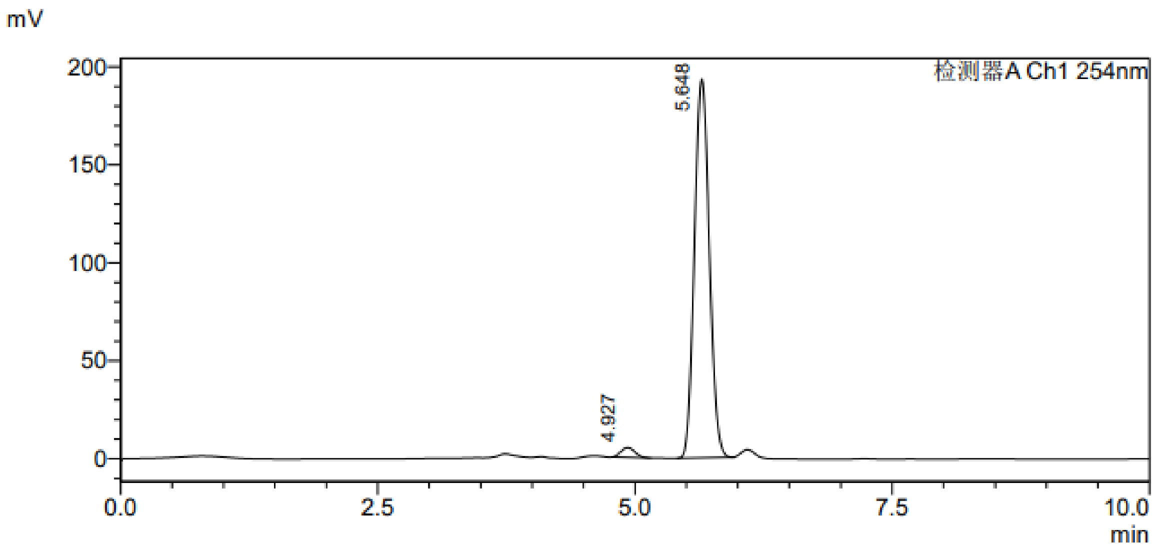

Peak#	Ret. Time	Area	Height	Conc.	Unit	Mark	Name
1	4.927	48453	5014	2.405		M	
2	5.648	1966540	193151	97.595		M	
总计		2014993	198164	100.000			

Supplementary Figure 79. HPLC spectrum of (*R*)-**3la**

(*R*)-4,5-diphenyl-3-vinyl-3,6-dihydro-2*H*-pyran (3ma)

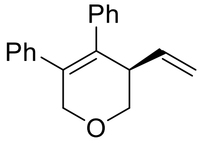

Chemical Formula: $C_{19}H_{18}O$
Exact Mass: 262.1358

3ma was prepared according to general procedure **2.1** using (*Z*)-4-((3-phenylprop-2-yn-1-yl)oxy)but-2-en-1-yl acetate **1m** (0.2 mmol, 48.9 mg) and iodobenzene **2a** (0.4 mmol, 81.6 mg). Purification by silica gel column chromatography (PE/EA = 20/1) gave **3ma** as a colorless oil (35.9 mg, 51% yield).

^{1}H NMR (400 MHz, $CDCl_3$): δ 7.20–7.06 (m, 6H), 7.05–6.95 (m, 4H), 5.95–5.75 (m, 1H), 5.10–4.90 (m, 2H), 4.63 (dd, *J* = 16.4, 1.2 Hz, 1H), 4.28 (dd, *J* = 16.4, 2.4 Hz, 1H), 4.08–3.91 (m, 2H), 3.35–3.15 (m, 1H);

^{13}C NMR (100 MHz, $CDCl_3$): δ 140.0, 138.4, 137.8, 134.9, 134.2, 129.4, 129.2, 127.9, 127.6, 126.8, 126.3, 116.5, 69.4, 69.2, 43.6;

IR (neat): 2961, 1599, 1493, 1443, 1262, 1110, 993, 916, 762, 698, 547 cm^{-1};

HRMS-ESI (m/z) Calcd for ($C_{19}H_{19}O$) ($[M+H]^+$): 263.1430; found: 263.1426;

HPLC conditions: OJ-H column (0.5% ^{i}PrOH in hexane, 0.5 mL/min, λ = 254 nm, 35 °C), t_R (major) = 11.0 min, t_R (minor) =14.6 min;

Optical Rotation: $[\alpha]^{25}_D$ = -127.7 (*c* = 1.50, $CHCl_3$) for 94% ee;

Absolute stereochemistry was determined through analogy with **3aj**.

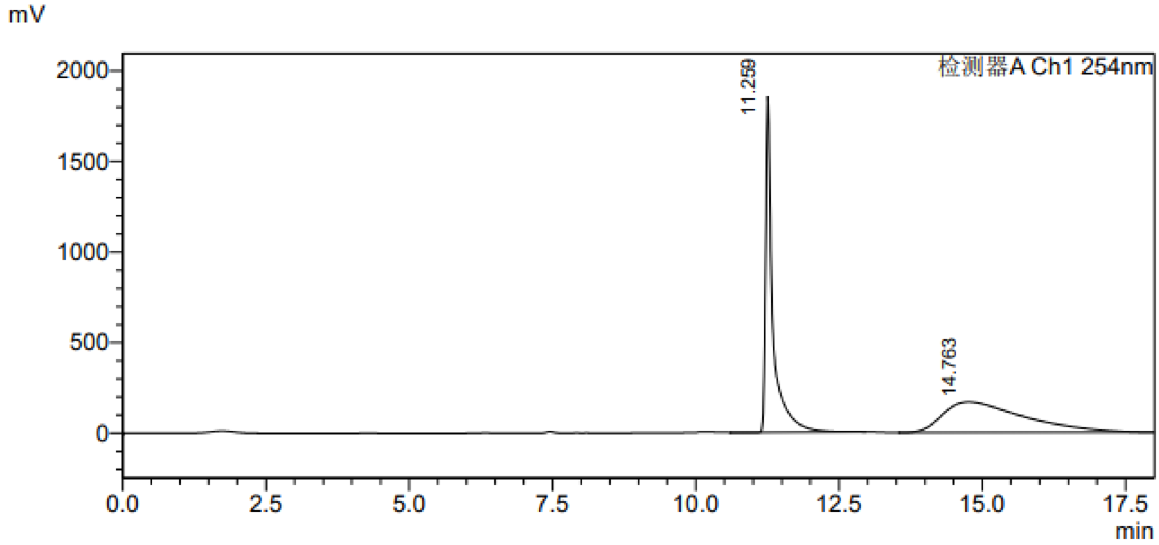

Peak#	Ret. Time	Area	Height	Conc.	Unit	Mark	Name
1	11.259	16405796	1855884	49.699		M	
2	14.763	16604521	169660	50.301		M	
总计		33010317	2025544	100.000			

Supplementary Figure 80. HPLC spectrum of racemic-**3ma**

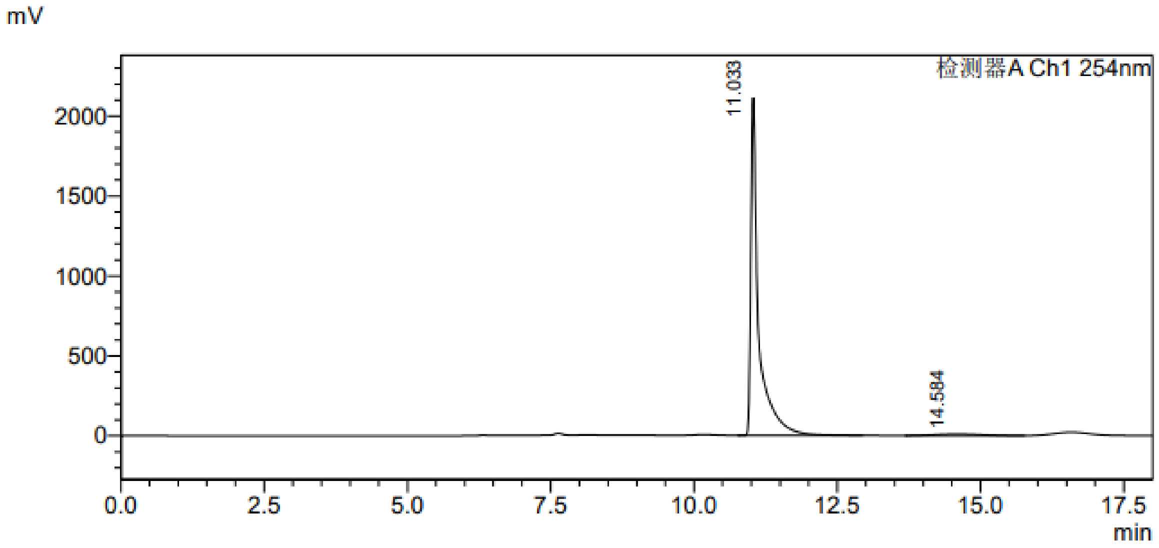

Peak#	Ret. Time	Area	Height	Conc.	Unit	Mark	Name
1	11.033	20072777	2113001	97.142		M	
2	14.584	590637	10433	2.858		M	
总计		20663415	2123433	100.000			

Supplementary Figure 81. HPLC spectrum of (*R*)-**3ma**

dimethyl (*R*)-6'-vinyl-5',6'-dihydro-[1,1':2',1''-terphenyl]-4',4'(3'*H*)-dicarboxylate (3na)

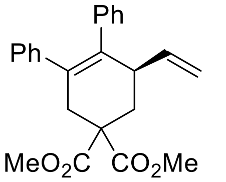

Chemical Formula: $C_{24}H_{24}O_4$
Exact Mass: 376.1675

3na was prepared according to general procedure **2.1** using dimethyl (*Z*)-2-(4-acetoxybut-2-en-1-yl)-2-(3-phenylprop-2-yn-1-yl)malonate **1n** (0.1 mmol, 35.8 mg) and iodobenzene **2a** (0.2 mmol, 40.8 mg). Purification by silica gel column chromatography (PE/EA = 20/1) gave **3na** as a yellow oil (24.5 mg, 65% yield).

^{1}H NMR (400 MHz, $CDCl_3$): δ 7.14–6.94 (m, 8H), 6.90–6.80 (m, 2H), 5.53 (ddd, *J* = 17.2, 10.0, 8.0 Hz, 1H), 4.89 (d, *J* = 17.2 Hz, 1H), 4.86 (d, *J* = 10.0 Hz, 1H), 3.79 (s, 3H), 3.73 (m, 3H), 3.50–3.40 (m, 1H), 3.09 (dd, *J* = 17.2, 2.8 Hz, 1H), 2.87 (dt, *J* = 17.2, 2.0 Hz, 1H), 2.62 (ddd, *J* = 13.6, 6.4, 2.0 Hz, 1H), 2.20 (dd, *J* = 13.6, 8.4 Hz, 1H);

^{13}C NMR (100 MHz, $CDCl_3$): δ 172.0, 171.4, 142.6, 140.7, 139.7, 135.9, 133.5, 129.6, 128.7, 127.6, 127.3, 126.0, 125.8, 116.1, 53.2, 52.6, 43.1, 37.1, 34.6;

IR (neat): 2951, 1738, 1442, 1201, 1057, 916, 760, 700 cm^{-1};

HRMS-ESI (m/z) Calcd for ($C_{24}H_{24}O_4Na$) ($[M+Na]^+$): 399.1567; found: 399.1568;

HPLC conditions: AD-H column (2% ^{i}PrOH in hexane, 1.0 mL/min, λ = 254 nm, 35 °C), t_R (minor) = 7.2 min, t_R (major) = 7.7 min;

Optical Rotation: $[\alpha]^{25}_D$ = -75.0 (*c* = 0.20, $CHCl_3$) for 92% ee;

Absolute stereochemistry was determined through analogy with **3aj**.

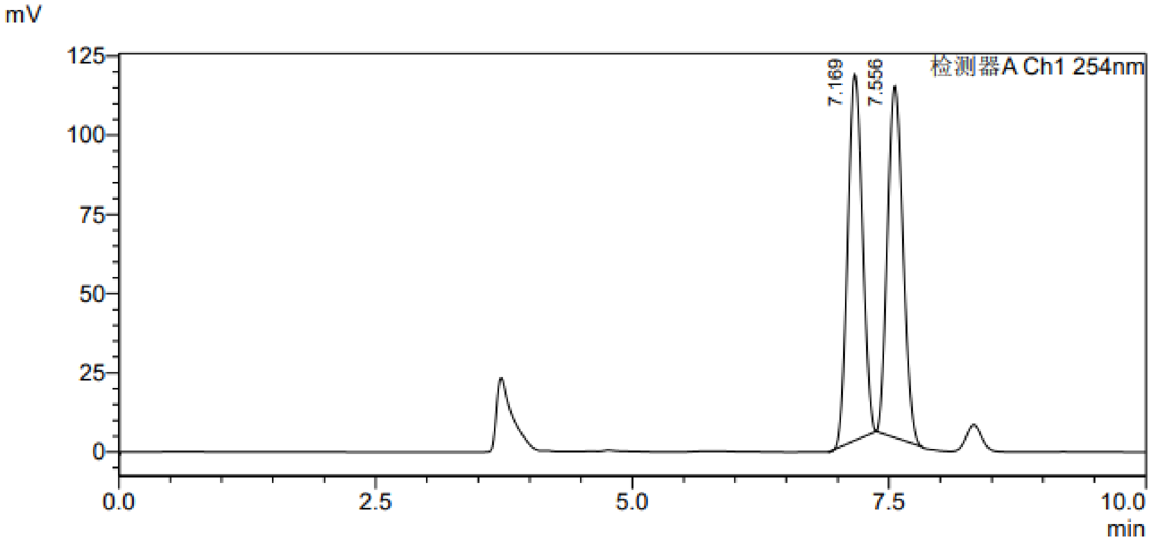

Peak#	Ret. Time	Area	Height	Conc.	Unit	Mark	Name
1	7.169	1170621	115452	50.206		M	
2	7.556	1161006	110927	49.794		M	
总计		2331627	226379	100.000			

Supplementary Figure 82. HPLC spectrum of racemic-**3na**

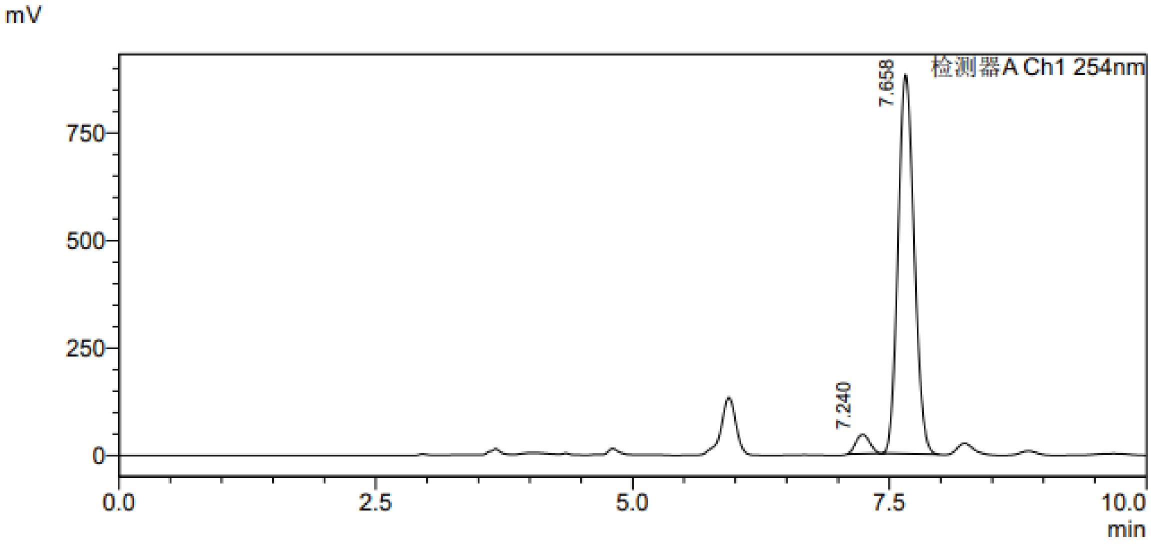

Peak#	Ret. Time	Area	Height	Conc.	Unit	Mark	Name
1	7.240	422441	44969	4.223		M	
2	7.658	9581423	879351	95.777		M	
总计		10003865	924320	100.000			

Supplementary Figure 83. HPLC spectrum of (*R*)-**3na**

(*Z*)-3-(1,3-diphenylpropylidene)-1-tosyl-4-vinylpyrrolidine (6aa)

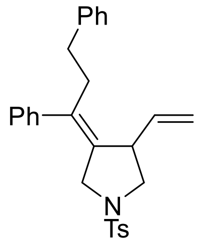

Chemical Formula: $C_{28}H_{29}NO_2S$
Exact Mass: 443.1919

6aa was prepared according to general procedure **2.2** using **1a** (0.2 mmol, 79.4 mg) and (2-bromoethyl)benzene **5a** (0.24 mmol, 44.4 mg). Purification by silica gel column chromatography (PE/EA = 10/1) gave **6aa** as a yellow solid (70.0 mg, 79% yield).

^{1}H NMR (400 MHz, $CDCl_3$): δ 7.61 (d, *J* = 8.4 Hz, 2H), 7.40–7.30 (m, 5H), 7.22 (t, *J* = 7.2 Hz, 2H), 7.16 (t, *J* = 7.2 Hz, 1H), 7.09 (d, *J* = 6.8 Hz, 2H), 7.03 (d, *J* = 6.8 Hz, 2H), 5.72 (ddd, *J* = 17.6, 10.0, 7.6 Hz, 1H), 5.11 (d, *J* = 16.8 Hz, 1H), 5.06 (d, *J* = 10 Hz, 1H), 3.80 (d, *J* = 14.4 Hz, 1H), 3.50 (d, *J* = 14.0 Hz, 1H), 3.42–3.32 (m, 1H), 3.29–3.17 (m, 2H), 2.66–2.54 (m, 2H), 2.50–2.35 (m, 2H), 2.44 (s, 3H);

^{13}C NMR (100 MHz, $CDCl_3$): δ 143.5, 141.7, 141.0, 137.7, 136.6, 133.4, 132.6, 129.5, 128.5, 128.24, 128.22, 127.8, 127.2,127.1, 125.8, 115.4, 53.6, 50.9, 45.1, 36.6, 34.0, 21.5;

IR (KBr): 3450, 2923, 1598, 1453, 1336, 1153, 912, 815, 699, 546 cm^{-1};

HRMS-ESI (m/z) Calcd for ($C_{28}H_{29}NO_2SNa$) ($[M+Na]^+$): 466.1811; found: 466.1821.

(*S*,*Z*)-3-(1,3-diphenylpropylidene)-4-((*E*)-styryl)-1-tosylpyrrolidine (7aa)

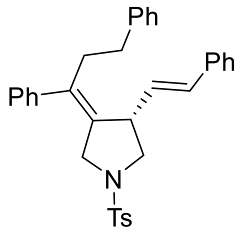

Chemical Formula: $C_{34}H_{33}NO_2S$
Exact Mass: 519.2232

7aa was prepared according to general procedure **2.2** using (*R*,*E*)-**4a** (0.1 mmol, 47.4 mg) and (2-bromoethyl)benzene **5a** (0.2 mmol, 37.0 mg). Purification by silica gel column chromatography (PE/EA = 10/1) gave **7aa** as a white solid (40.5 mg, 78% yield).

^{1}H NMR (600 MHz, $CDCl_3$): δ 7.66–7.58 (m, 2H), 7.40–7.35 (m, 2H), 7.34–7.26 (m, 7H), 7.25–7.22 (m, 2H), 7.16–7.06 (m, 5H), 6.95–6.86 (m, 2H), 6.39 (d, *J* = 15.8 Hz, 1H), 6.01 (dd, *J* = 15.8, 8.2 Hz, 1H), 3.84 (d, *J* = 14.3 Hz, 1H), 3.59 (d, *J* = 14.3 Hz, 1H), 3.56–3.49 (m, 1H), 3.35 (dd, *J* = 10.0, 7.0 Hz, 1H), 3.29 (dd, *J* = 10.0, 3.3 Hz, 1H), 2.71–2.63 (m, 1H), 2.61–2.52 (m, 1H), 2.47–2.36 (m, 5H);

^{13}C NMR (151 MHz, $CDCl_3$): δ 143.7, 141.6, 141.1, 137.0, 136.7, 133.7, 132.7, 130.7, 129.7, 129.3, 128.6, 128.6, 128.3, 128.3, 127.9, 127.7, 127.6, 127.3, 126.3, 125.9, 54.0, 51.0, 44.7, 36.7, 34.2, 21.6;

HRMS: (ESI) calcd for $C_{34}H_{34}NO_2S^+$ ($[M+H]^+$): 520.2305; found: 520.2307;

HPLC conditions: AD-H column (2% ^{i}PrOH in hexane, 1.0 mL/min, λ = 254 nm, 30 °C), t_R (minor) = 7.2 min, t_R (major) = 7.7 min;

Optical Rotation: $[\alpha]^{25}_D$ = -14.5 (*c* = 0.67, $CHCl_3$) for 91% ee;

Absolute stereochemistry was determined by X-ray diffraction crystallography.

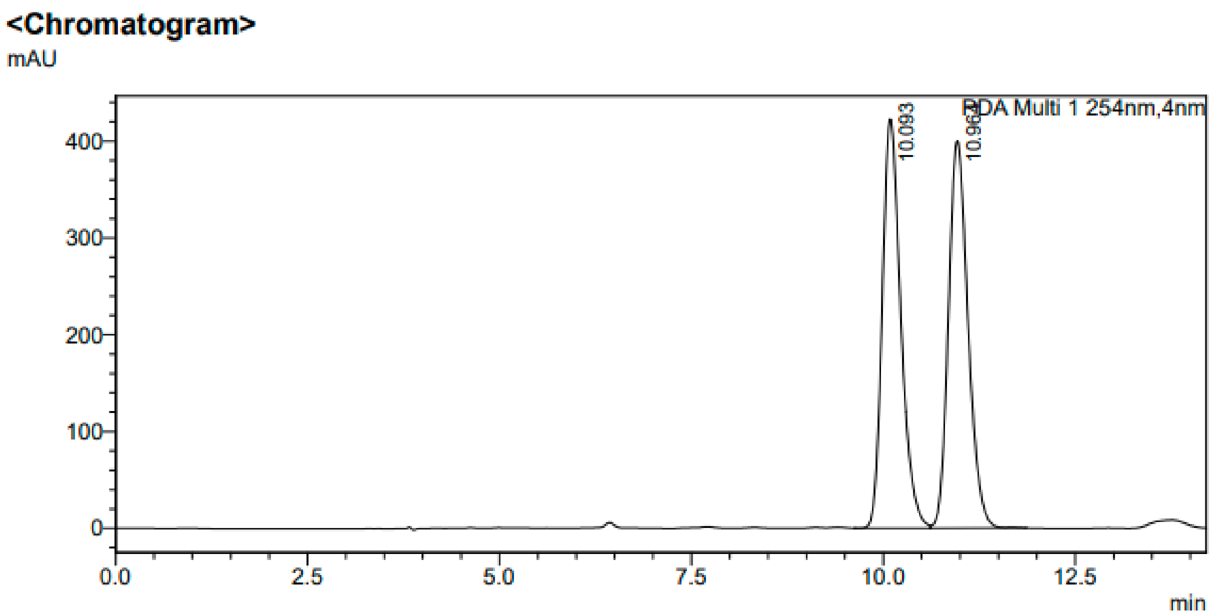

<Peak Table>

PDA Ch1 254nm

Peak#	Ret. Time	Area	Height	Conc.	Unit	Mark	Name
1	10.093	7103110	422761	0.000		M	
2	10.964	7092720	399874	0.000		V M	
Total		14195830	822636				

Supplementary Figure 82. HPLC spectrum of racemic-**7aa**

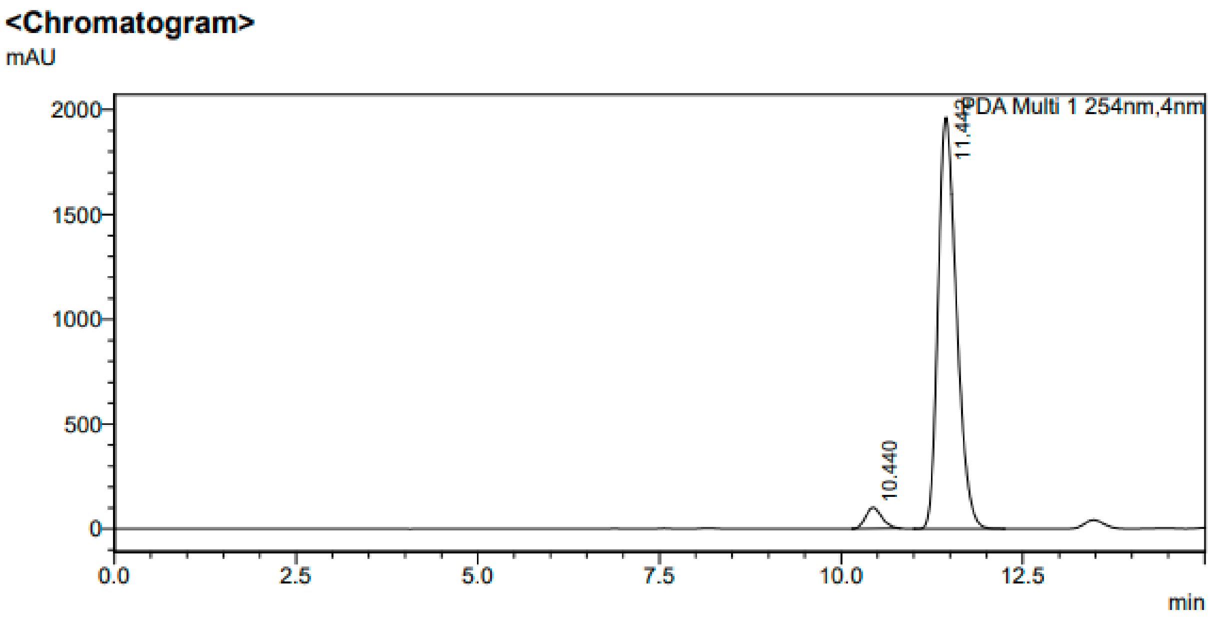

<Peak Table>

PDA Ch1 254nm

Peak#	Ret. Time	Area	Height	Conc.	Unit	Mark	Name
1	10.440	1554141	99539	0.000		M	
2	11.442	34741482	1962086	0.000		M	
Total		36295623	2061625				

Supplementary Figure 83. HPLC spectrum of (*S*)-**7aa** prepared from (*R*,*E*)-**4a**

(*S*,*Z*)-3-(1,4-diphenylbutylidene)-4-((*E*)-styryl)-1-tosylpyrrolidine (7ab)

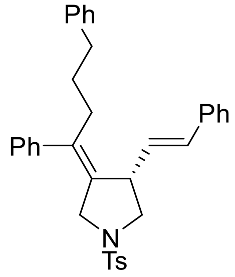

Chemical Formula: $C_{35}H_{35}NO_2S$
Exact Mass: 533.2389

7ab was prepared according to general procedure **2.2** using (*R*,*E*)-**4a** (0.1 mmol, 47.4 mg) and (3-bromopropyl)benzene **5b** (0.2 mmol, 39.8 mg). Purification by silica gel column chromatography (PE/EA = 10/1) gave **7ab** as a colorless oil (40.6 mg, 76% yield).

^{1}H NMR (600 MHz, $CDCl_3$): δ 7.63 (d, *J* = 8.0 Hz, 2H), 7.35–7.26 (m, 9H), 7.24 (d, *J* = 7.4 Hz, 1H), 7.15–7.09 (m, 3H), 7.05–7.00 (m, 2H), 6.87–6.83 (m, 2H), 6.31 (d, *J* = 15.8 Hz, 1H), 5.98 (dd, *J* = 15.8, 8.4 Hz, 1H), 3.80 (d, *J* = 14.3 Hz, 1H), 3.55 (d, *J* = 14.4 Hz, 2H), 3.42 (dd, *J* = 10.0, 7.1 Hz, 1H), 3.29 (dd, *J* = 10.1, 3.2 Hz, 1H), 2.40 (s, 3H), 2.35–2.28 (m, 1H), 1.50-1.41 (m, 2H);

^{13}C NMR (151 MHz, $CDCl_3$): δ 143.6, 141.9, 141.3, 137.7, 136.7, 133.1, 132.6, 130.4, 129.6, 129.5, 128.6, 128.5, 128.2, 128.2, 127.9, 127.5, 127.1, 126.3, 125.6, 54.0, 51.0, 44.7, 35.8, 34.0, 29.8, 21.5;

HRMS: (ESI) calcd for $C_{35}H_{36}NO_2S^+$ ($[M+H]^+$): 534.2461; found: 534.2468;

HPLC conditions: AD-H column (15% ^{i}PrOH in hexane, 1.0 mL/min, λ = 254 nm, 30 °C), t_R (minor) = 7.8 min, t_R (major) = 9.9 min;

Optical Rotation: $[\alpha]^{25}_D$ = 12.9 (*c* = 0.50, $CHCl_3$) for 92% ee;

Absolute stereochemistry was determined through analogy with **7aa**.

<Chromatogram>

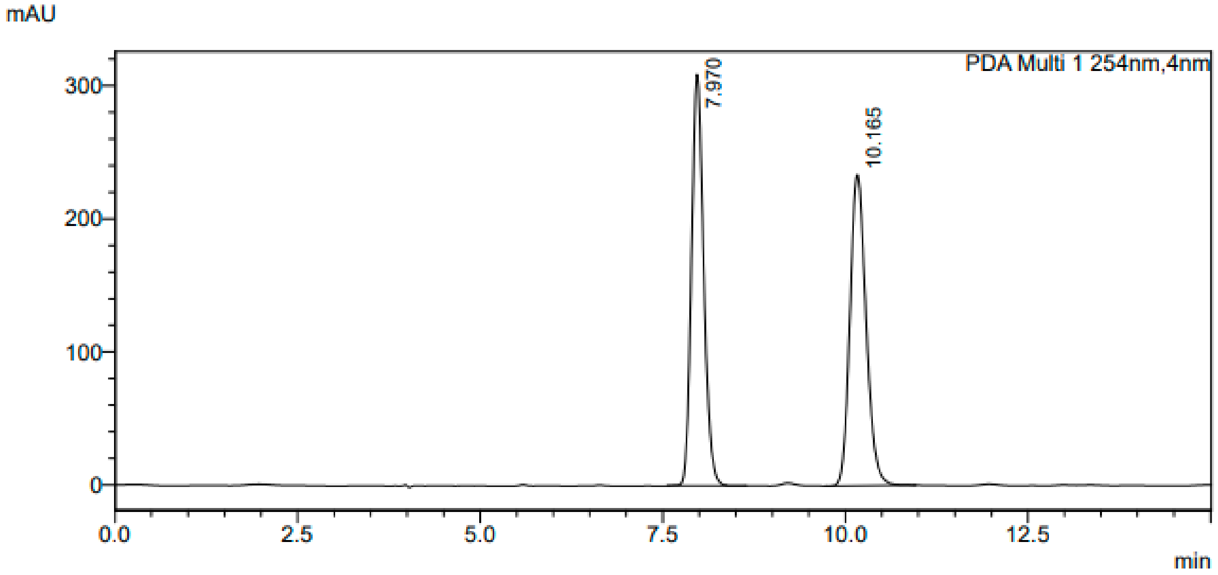

<Peak Table>

PDA Ch1 254nm

Peak#	Ret. Time	Area	Height	Conc.	Unit	Mark	Name
1	7.970	3601330	309177	0.000		M	
2	10.165	3589551	233693	0.000		M	
Total		7190882	542869				

Supplementary Figure 84. HPLC spectrum of racemic-**7ab**

<Chromatogram>

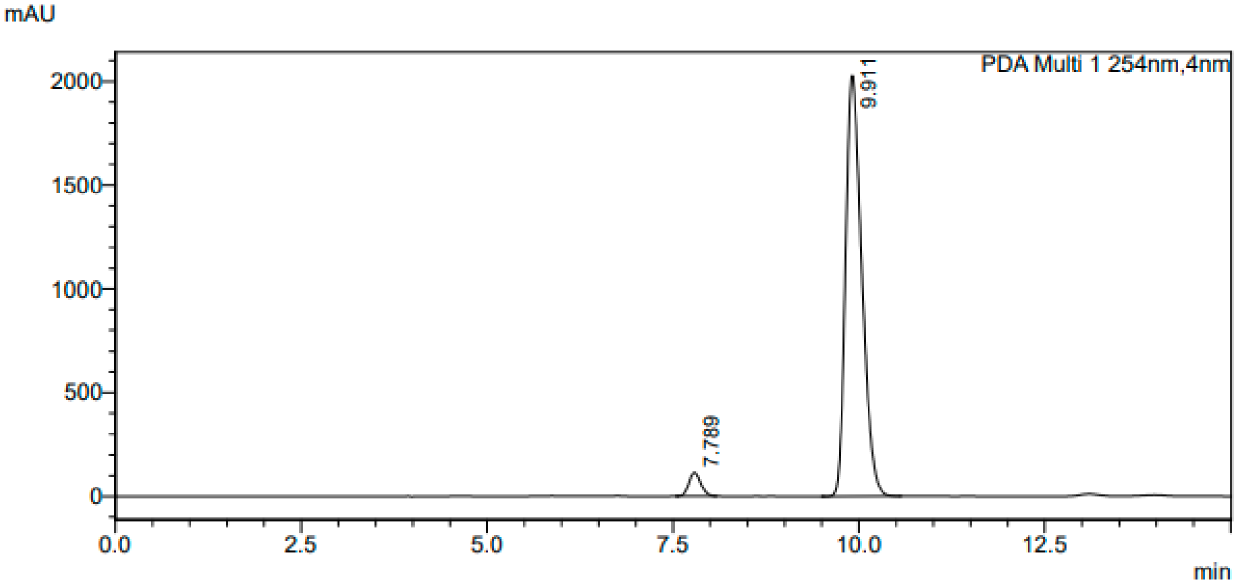

<Peak Table>

PDA Ch1 254nm

Peak#	Ret. Time	Area	Height	Conc.	Unit	Mark	Name
1	7.789	1260729	113706	0.000		M	
2	9.911	30771569	2027236	0.000		M	
Total		32032298	2140942				

Supplementary Figure 85. HPLC spectrum of (*S*)-**7ab**

(*S*,*Z*)-3-(4-chloro-1-phenylbutylidene)-4-((*E*)-styryl)-1-tosylpyrrolidine (7ac)

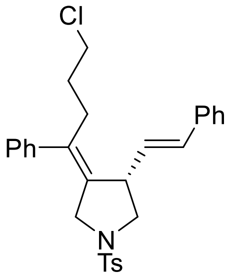

7ac was prepared according to general procedure **2.2** using (*R*,*E*)-**4a** (0.1 mmol, 47.4 mg) and 1-bromo-3-chloropropane **5c** (0.2 mmol, 31.5 mg). Purification by silica gel column chromatography (PE/EA = 10/1) gave **7ac** as a colorless oil (39.9 mg, 81% yield).

^{1}H NMR (600 MHz, $CDCl_3$): δ 7.68–7.60 (m, 2H), 7.37–7.27 (m, 9H), 7.26–7.22 (m, 1H), 7.07–7.00 (m, 2H), 6.46 (d, *J* = 15.8 Hz, 1H), 6.03 (dd, *J* = 15.8, 8.4 Hz, 1H), 3.84 (dd, *J* = 14.4, 1.7 Hz, 1H), 3.75–3.68 (m, 1H), 3.57 (d, *J* = 14.4 Hz, 1H), 3.42 (dd, *J* = 10.0, 6.9 Hz, 1H), 3.39–3.32 (m, 3H), 2.55–2.48 (m, 2H), 2.42 (s, 3H), 1.68–1.59 (m, 2H);

^{13}C NMR (151 MHz, $CDCl_3$): δ 143.7, 140.7, 136.6, 136.1, 134.4, 132.6, 130.7, 129.7, 129.2, 128.7, 128.6, 127.8, 127.6, 127.5, 127.4, 126.2, 53.9, 51.0, 44.7, 44.7, 31.5, 30.5, 21.5;

HRMS: (ESI) calcd for $C_{29}H_{31}ClNO_2S^+$ ($[M+H]^+$): 492.1759; found: 492.17587;

HPLC conditions: AD-H column (20% ^{i}PrOH in hexane, 1.0 mL/min, λ = 254 nm, 30 °C), t_R (minor) = 8.2 min, t_R (major) = 10.6 min;

Optical Rotation: $[\alpha]^{25}_D$ = 8.5 (*c* = 1.25, $CHCl_3$) for 92% ee;

Absolute stereochemistry was determined through analogy with **7aa**

<Chromatogram>

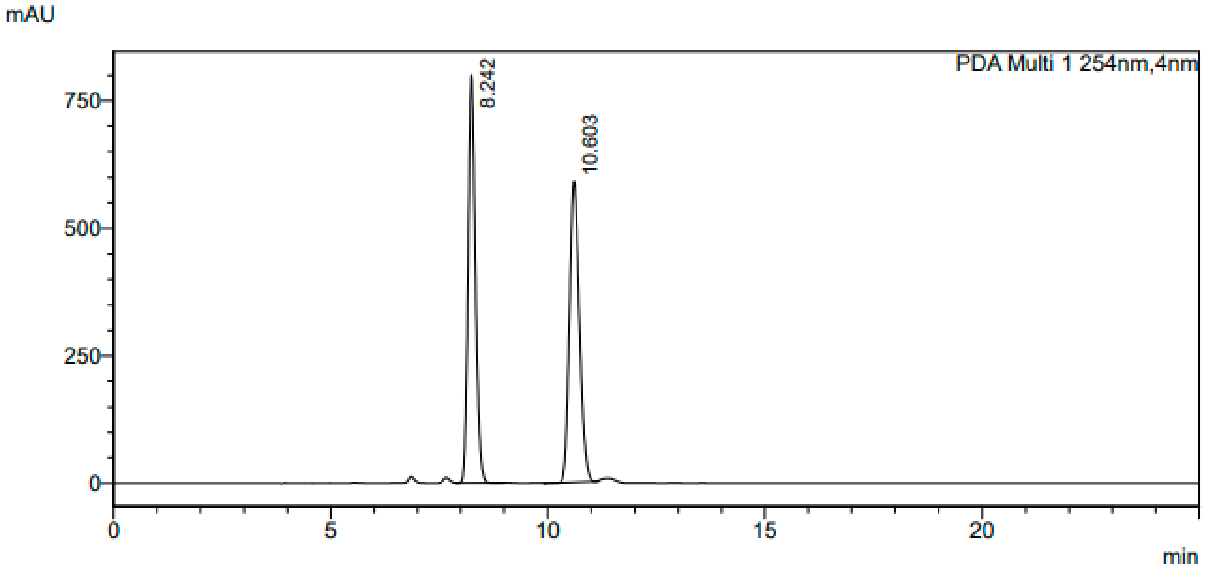

<Peak Table>

PDA Ch1 254nm

Peak#	Ret. Time	Area	Height	Conc.	Unit	Mark	Name
1	8.242	9604796	800460	0.000		M	
2	10.603	9486492	590457	0.000		M	
Total		19091287	1390917				

Supplementary Figure 86. HPLC spectrum of racemic-**7ac**

<Chromatogram>

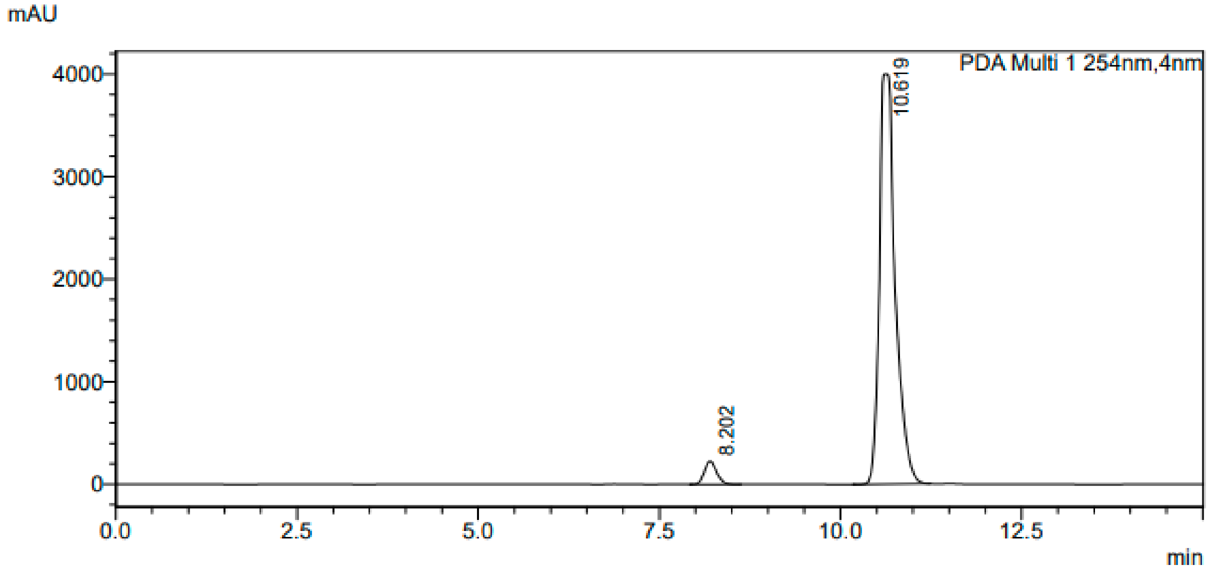

<Peak Table>

PDA Ch1 254nm

Peak#	Ret. Time	Area	Height	Conc.	Unit	Mark	Name
1	8.202	2681794	226292	0.000			
2	10.619	61136268	3998328	0.000		M	
Total		63818062	4224620				

Supplementary Figure 87. HPLC spectrum of (*S*)-**7ac**

(*S*,*Z*)-3-(4-fluoro-1-phenylbutylidene)-4-((*E*)-styryl)-1-tosylpyrrolidine (7ad)

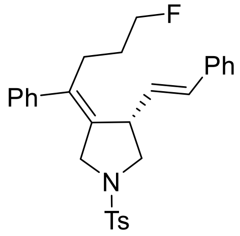

Chemical Formula: $C_{29}H_{30}FNO_2S$
Exact Mass: 475.1981

7ad was prepared according to general procedure **2.2** using (*R*,*E*)-**4a** (0.1 mmol, 47.4 mg) and 1-bromo-3-fluoropropane **5d** (0.2 mmol, 28.2 mg). Purification by silica gel column chromatography (PE/EA = 15/1) gave **7ad** as a colorless oil (39.5 mg, 83% yield).

^{1}H NMR (600 MHz, $CDCl_3$): δ 7.64 (d, *J* = 8.2 Hz, 2H), 7.37–7.31 (m, 2H), 7.33–7.25 (m, 7H), 7.27–7.20 (m, 1H), 7.08–7.01 (m, 2H), 6.43 (d, *J* = 15.8 Hz, 1H), 6.03 (dd, *J* = 15.8, 8.3 Hz, 1H), 4.32–4.25 (m, 1H), 4.24–4.18 (m, 1H), 3.88–3.83 (m, 1H), 3.72–3.65 (m, 1H), 3.57 (d, *J* = 14.3 Hz, 1H), 3.42 (dd, *J* = 10.0, 6.9 Hz, 1H), 3.37 (dd, *J* = 10.1, 3.0 Hz, 1H), 2.56–2.41 (m, 2H), 2.41 (s, 3H), 1.59–1.44 (m, 2H);

^{19}F NMR (565 MHz, $CDCl_3$): δ -219.01–-219.41 (m);

^{13}C NMR (151 MHz, $CDCl_3$): δ 143.6, 140.7, 136.7, 136.3, 134.0, 132.6, 130.6, 129.6, 129.2, 128.6, 128.5, 127.8, 127.5, 127.5, 127.3, 126.2, 83.3 (d, *J* = 165.2 Hz), 53.9, 50.9, 44.6, 30.0 (d, *J* = 4.9 Hz), 28.5 (d, *J* = 19.9 Hz), 21.5;

HRMS: (ESI) calcd for $C_{29}H_{31}F_1N_1O_2S^+$ ($[M+H]^+$): 476.2054; found: 476.2052;

HPLC conditions: AD-H column (15% ^{i}PrOH in hexane, 1.0 mL/min, λ = 254 nm, 30 °C), t_R (minor) = 10.1 min, t_R (major) = 11.8 min;

Optical Rotation: $[\alpha]^{25}_D$ = 9.9 (*c* = 1, $CHCl_3$) for 90% ee;

Absolute stereochemistry was determined through analogy with **7aa**.

<Chromatogram>

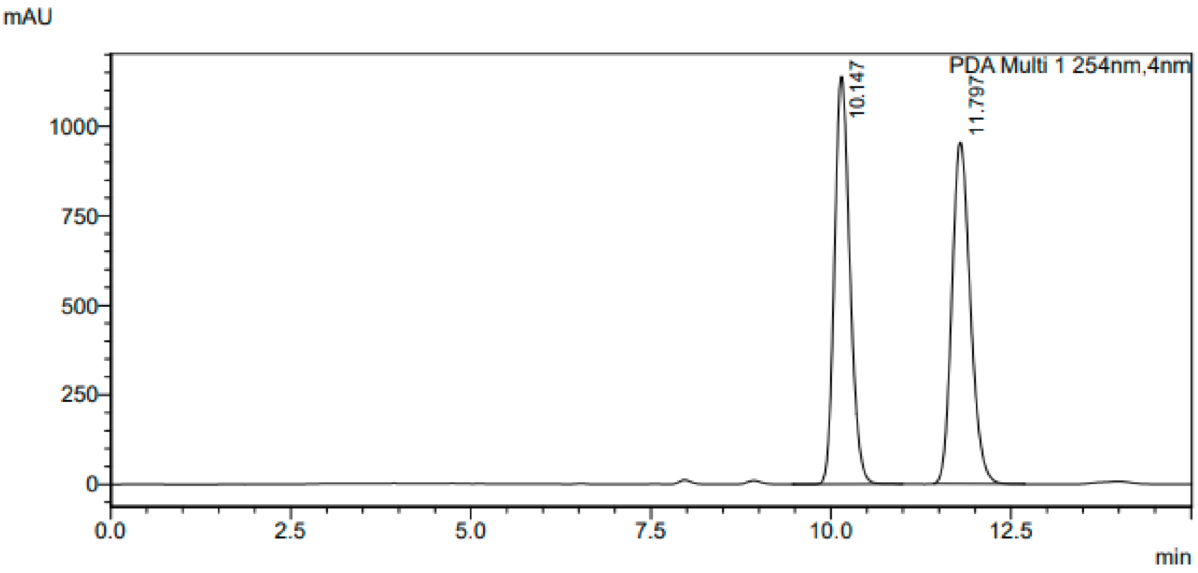

<Peak Table>

PDA Ch1 254nm

Peak#	Ret. Time	Area	Height	Conc.	Unit	Mark	Name
1	10.147	16954478	1138286	0.000		M	
2	11.797	16999305	953898	0.000		M	
Total		33953783	2092185				

Supplementary Figure 88. HPLC spectrum of racemic-**7ad**

<Chromatogram>

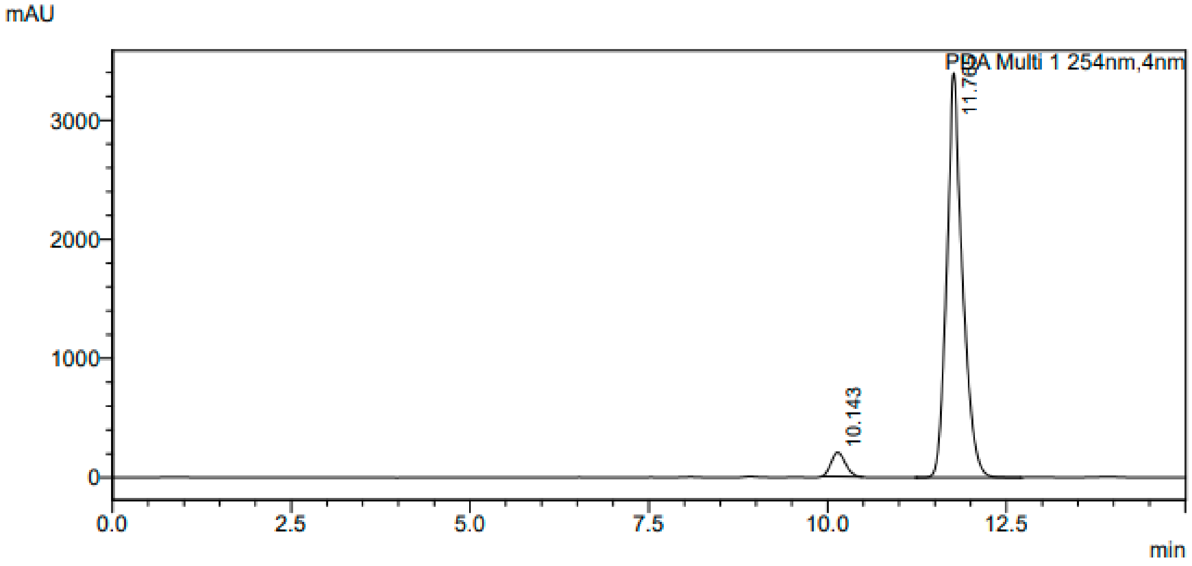

<Peak Table>

PDA Ch1 254nm

Peak#	Ret. Time	Area	Height	Conc.	Unit	Mark	Name
1	10.143	2851471	202870	0.000		M	
2	11.765	51868230	3393562	0.000		M	
Total		54719701	3596432				

Supplementary Figure 89. HPLC spectrum of (*S*)-**7ad**

(*S*,*Z*)-3-(4-((tert-butyldimethylsilyl)oxy)-1-phenylbutylidene)-4-((*E*)-styryl)-1-tosylpyrrolidine (7ae)

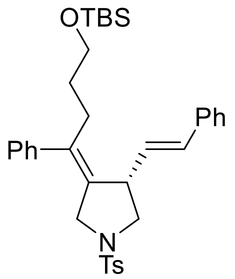

Chemical Formula: $C_{35}H_{45}NO_3SSi$
Exact Mass: 587.2889

7ae was prepared according to general procedure **2.2** using (*R*,*E*)-**4a** (0.1 mmol, 47.4 mg) and (3-bromopropoxy)(*tert*-butyl)dimethylsilane **5e** (0.2 mmol, 50.5 mg). Purification by silica gel column chromatography (PE/EA = 20/1) gave **7ae** as a colorless oil (31.2 mg, 59% yield).

^{1}H NMR (600 MHz, $CDCl_3$): δ 7.75–7.68 (m, 2H), 7.42–7.39 (m, 2H), 7.39–7.34 (m, 7H), 7.31 (s, 1H), 7.15–7.10 (m, 2H), 6.50 (d, *J* = 15.8 Hz, 1H), 6.11 (dd, *J* = 15.8, 8.0 Hz, 1H), 3.92 (dd, *J* = 14.3, 1.6 Hz, 1H), 3.81–3.76 (m, 1H), 3.67 (s, 1H), 3.53–3.45 (m, 4H), 2.49 (s, 4H), 2.47–2.41 (m, 1H), 1.51–1.41 (m, 2H), 0.89 (s, 9H), -0.00 (d, *J* = 3.9 Hz, 6H);

^{13}C NMR (151 MHz, $CDCl_3$): δ 137.3, 136.8, 133.1, 132.8, 130.3, 129.6, 129.4, 128.5, 128.5, 127.8, 127.6, 127.4, 127.1, 126.2, 62.6, 53.9, 50.8, 44.4, 30.9, 30.8, 25.9, 21.5, 18.2, -5.4;

HRMS: (ESI) calcd for $C_{35}H_{46}NO_3SSi^+$ ($[M+H]^+$): 588.2962; found: 588.2957;

HPLC conditions: OD-H column (20% ^{i}PrOH in hexane, 1.0 mL/min, λ = 254 nm, 30 °C), t_R (major) = 5.3 min, t_R (minor) = 6.8 min;

Optical Rotation: $[\alpha]^{25}_D$ = 3.8 (*c* = 0.67, $CHCl_3$) for 92% ee;

Absolute stereochemistry was determined through analogy with **7aa**.

<Chromatogram>

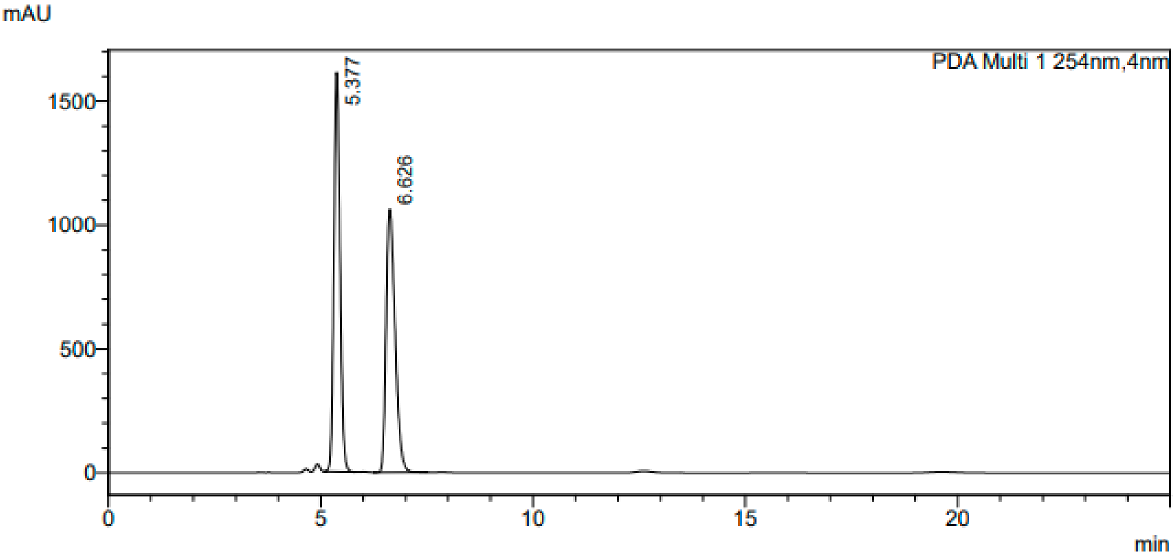

<Peak Table>

PDA Ch1 254nm

Peak#	Ret. Time	Area	Height	Conc.	Unit	Mark	Name
1	5.377	16113895	1611105	0.000		M	
2	6.626	15671128	1061578	0.000		M	
Total		31785023	2672683				

Supplementary Figure 90. HPLC spectrum of racemic-**7ae**

<Chromatogram>

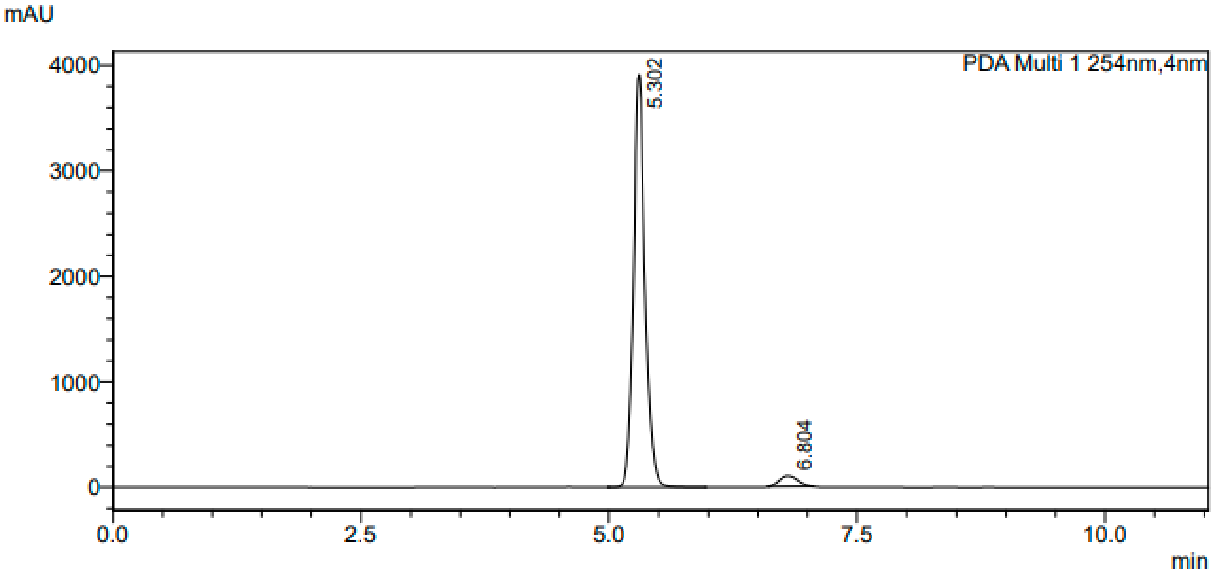

<Peak Table>

PDA Ch1 254nm

Peak#	Ret. Time	Area	Height	Conc.	Unit	Mark	Name
1	5.302	31500707	3911356	0.000		M	
2	6.804	1340455	103234	0.000		M	
Total		32841163	4014589				

Supplementary Figure 91. HPLC spectrum of (*S*)-**7ae**

(*Z*)-4-phenyl-4-((*S*)-4-((*E*)-styryl)-1-tosylpyrrolidin-3-ylidene)butyl acetate (7af)

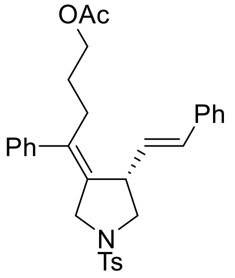

Chemical Formula: $C_{31}H_{33}NO_4S$
Exact Mass: 515.2130

7af was prepared according to general procedure **2.2** using (*R*,*E*)-**4a** (0.1 mmol, 47.4 mg) and 3-bromopropyl acetate **5f** (0.2 mmol, 50.5 mg). Purification by silica gel column chromatography (PE/EA = 5/1) gave **7af** as a colorless oil (39.2 mg, 76% yield).

^{1}H NMR (600 MHz, $CDCl_3$): δ 7.66–7.61 (m, 2H), 7.36–7.33 (m, 2H), 7.31 (d, *J* = 1.7 Hz, 1H), 7.31–7.26 (m, 6H), 7.25–7.22 (m, 1H), 7.06–7.02 (m, 2H), 6.42 (d, *J* = 15.8 Hz, 1H), 6.03 (dd, *J* = 15.8, 8.2 Hz, 1H), 3.91–3.84 (m, 2H), 3.82 (dd, *J* = 14.3, 1.4 Hz, 1H), 3.68–3.63 (m, 1H), 3.56 (d, *J* = 14.3 Hz, 1H), 3.42 (dd, *J* = 10.0, 7.0 Hz, 1H), 3.34 (dd, *J* = 10.0, 3.1 Hz, 1H), 2.45–2.35 (m, 5H), 1.88 (s, 3H), 1.52–1.45 (m, 2H);

^{13}C NMR (151 MHz, $CDCl_3$) δ 170.9, 143.7, 140.8, 136.6, 136.6, 133.8, 132.5, 130.6, 129.6, 129.3, 128.6, 128.6, 127.8, 127.6, 127.5, 127.3, 126.2, 63.9, 54.0, 51.0, 44.6, 30.6, 26.8, 21.5, 20.8;

HRMS: (ESI) calcd for $C_{31}H_{34}NO_4S^+$ ($[M+H]^+$): 516.2203; found: 516.2200;

HPLC conditions: OD-H column (20% ^{i}PrOH in hexane, 1.0 mL/min, λ = 254 nm, 30 °C), t_R (major) = 14.3 min, t_R (minor) = 17.0 min;

Optical Rotation: $[\alpha]^{25}_D$ = -3.7 (*c* = 1.27, $CHCl_3$) for 92% ee;

Absolute stereochemistry was determined through analogy with **7aa**.

<Chromatogram>

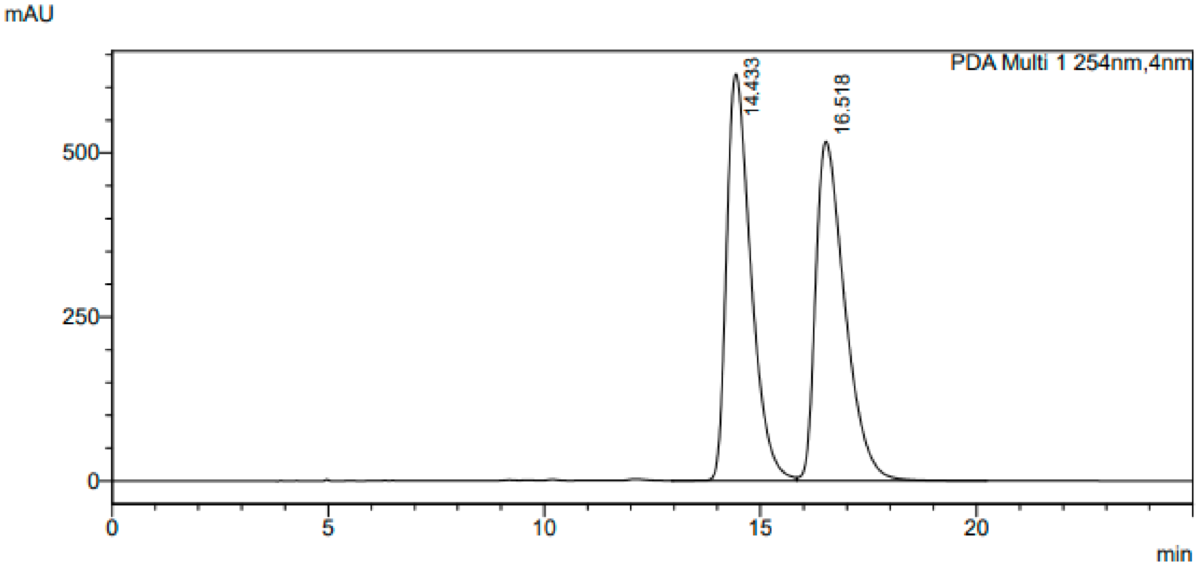

<Peak Table>

PDA Ch1 254nm

Peak#	Ret. Time	Area	Height	Conc.	Unit	Mark	Name
1	14.433	24306808	620233	0.000		M	
2	16.518	24408278	517028	0.000		V M	
Total		48715086	1137260				

Supplementary Figure 92. HPLC spectrum of racemic-**7af**

<Chromatogram>

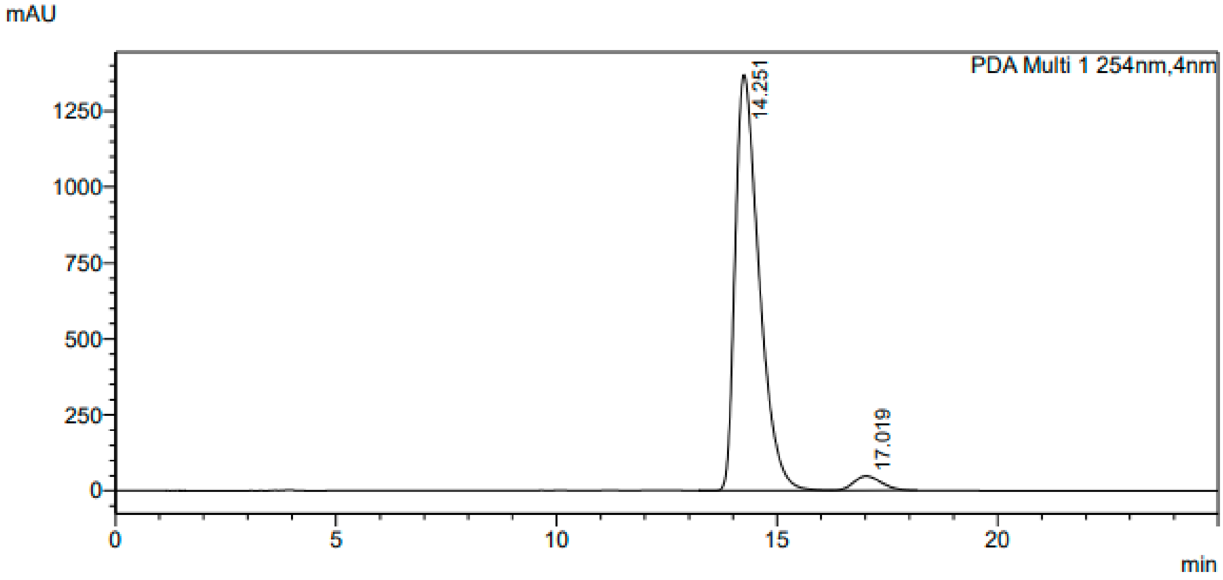

<Peak Table>

PDA Ch1 254nm

Peak#	Ret. Time	Area	Height	Conc.	Unit	Mark	Name
1	14.251	51707430	1367824	0.000		M	
2	17.019	2108894	47443	0.000		V M	
Total		53816324	1415267				

Supplementary Figure 93. HPLC spectrum of (*S*)-**7af**

methyl (*Z*)-5-phenyl-5-((*S*)-4-((*E*)-styryl)-1-tosylpyrrolidin-3-ylidene)pentanoate (7ag)

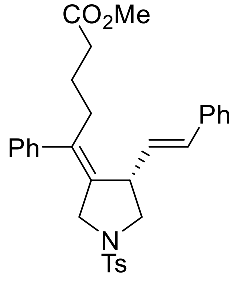

7ag was prepared according to general procedure **2.2** using (*R*,*E*)-**4a** (0.1 mmol, 47.4 mg) and methyl 4-bromobutanoate **5g** (0.2 mmol, 36.2 mg). Purification by silica gel column chromatography (PE/EA = 5/1) gave **7ag** as a colorless oil (35.6 mg, 69% yield).

^{1}H NMR (600 MHz, $CDCl_3$): δ 7.66–7.61 (m, 2H), 7.36–7.27 (m, 9H), 7.25–7.21 (m, 1H), 7.06–7.02 (m, 2H), 6.43 (d, *J* = 15.8 Hz, 1H), 6.03 (dd, *J* = 15.8, 8.2 Hz, 1H), 3.83 (dd, *J* = 14.3, 1.6 Hz, 1H), 3.69–3.64 (m, 1H), 3.57 (d, *J* = 14.4 Hz, 1H), 3.51 (s, 3H), 3.42 (dd, *J* = 10.0, 6.9 Hz, 1H), 3.36 (dd, *J* = 10.0, 3.0 Hz, 1H), 2.42–2.34 (m, 5H), 2.13 (t, *J* = 7.4 Hz, 2H), 1.53–1.45 (m, 2H);

^{13}C NMR (151 MHz, $CDCl_3$): δ 173.5, 140.9, 136.8, 136.6, 133.8, 132.6, 130.5, 129.6, 129.3, 128.5, 128.5, 127.8, 127.5, 127.2, 126.2, 53.9, 51.3, 50.9, 44.5, 33.5, 33.4, 23.0, 21.5;

HRMS: (ESI) calcd for $C_{31}H_{34}NO_4S^+$ ($[M+H]^+$): 516.2203; found: 516.2202;

HPLC conditions: AD-H column (20% ^{i}PrOH in hexane, 1.0 mL/min, λ = 254 nm, 30 °C), t_R (minor) = 10.2 min, t_R (major) = 12.1 min;

Optical Rotation: $[\alpha]^{25}_D$ = -4.2 (*c* = 1.21, $CHCl_3$) for 92% ee;

Absolute stereochemistry was determined through analogy with **7aa**.

<Chromatogram>

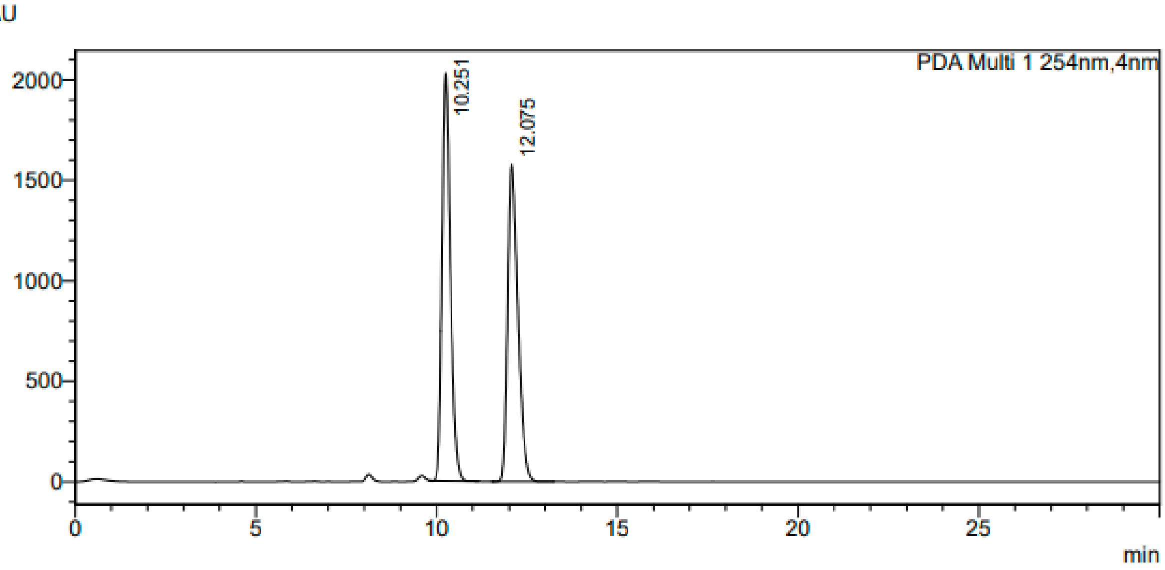

<Peak Table>

PDA Ch1 254nm

Peak#	Ret. Time	Area	Height	Conc.	Unit	Mark	Name
1	10.251	32390876	2027037	0.000		M	
2	12.075	31811664	1579007	0.000		M	
Total		64202540	3606044				

Supplementary Figure 94. HPLC spectrum of racemic-**7ag**

<Chromatogram>

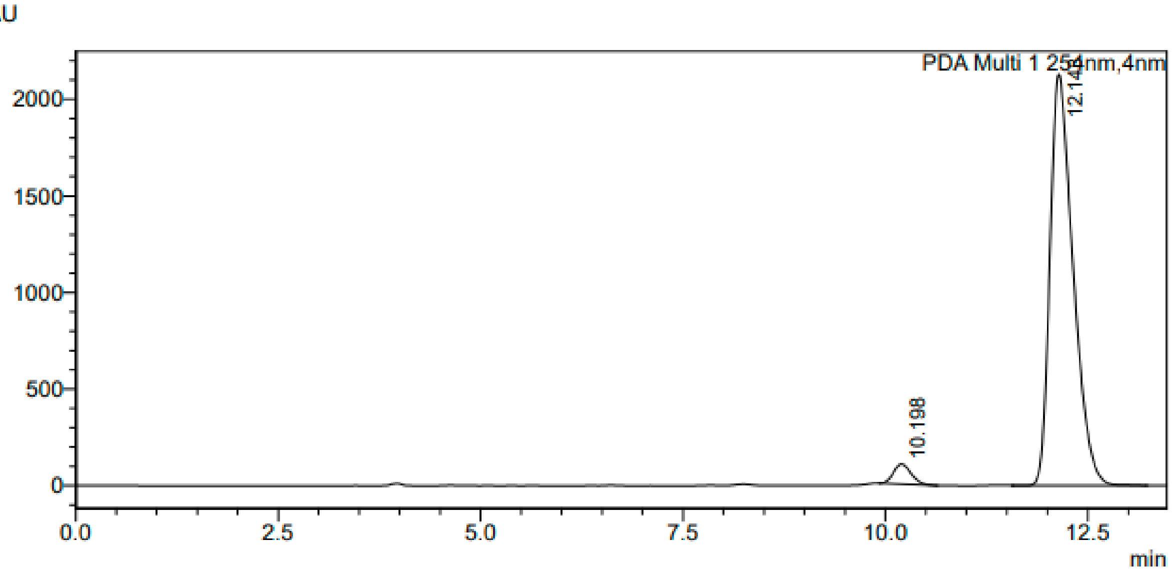

<Peak Table>

PDA Ch1 254nm

Peak#	Ret. Time	Area	Height	Conc.	Unit	Mark	Name
1	10.198	1577466	104258	0.000		M	
2	12.143	42402662	2128527	0.000		M	
Total		43980128	2232785				

Supplementary Figure 95. HPLC spectrum of (*S*)-**7ag**

(*Z*)-5-phenyl-5-((*S*)-4-((*E*)-styryl)-1-tosylpyrrolidin-3-ylidene)pentanenitrile (7ah)

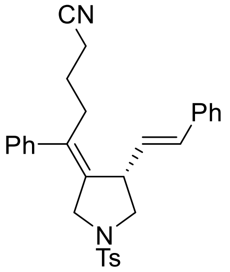

Chemical Formula: $C_{30}H_{30}N_2O_2S$
Exact Mass: 482.2028

7ah was prepared according to general procedure **2.2** using (*R*,*E*)-**4a** (0.1 mmol, 47.4 mg) and methyl 4-bromobutanenitrile **5h** (0.2 mmol, 29.6 mg). Purification by silica gel column chromatography (PE/EA = 5/1) gave **7ah** as a colorless oil (34.3 mg, 71% yield).

^{1}H NMR (600 MHz, $CDCl_3$): δ 7.66–7.61 (m, 2H), 7.35 (dd, *J* = 8.1, 6.7 Hz, 2H), 7.34–7.28 (m, 7H), 7.26–7.23 (m, 1H), 7.06–7.02 (m, 2H), 6.48 (dd, *J* = 15.8, 0.9 Hz, 1H), 6.04 (dd, *J* = 15.8, 8.6 Hz, 1H), 3.86 (dd, *J* = 14.5, 1.5 Hz, 1H), 3.73–3.67 (m, 1H), 3.57 (d, *J* = 14.5 Hz, 1H), 3.43 (dd, *J* = 10.1, 7.0 Hz, 1H), 3.34 (dd, *J* = 10.1, 3.1 Hz, 1H), 2.56–2.46 (m, 2H), 2.42 (s, 3H), 2.12 (t, *J* = 7.0 Hz, 2H), 1.53–1.45 (m, 2H);

^{13}C NMR (151 MHz, $CDCl_3$): δ 143.7, 140.2, 136.4, 135.4, 135.1, 132.5, 130.8, 129.7, 129.1, 128.8, 128.6, 127.8, 127.7, 127.6, 127.4, 126.2, 54.0, 51.0, 44.8, 33.0, 23.4, 21.5, 16.8;

HRMS: (ESI) calcd for $C_{30}H_{31}N_2O_2S^+$ ($[M+H]^+$): 483.2101; found: 483.2100;

HPLC conditions: AD-H column (20% ^{i}PrOH in hexane, 1.0 mL/min, λ = 254 nm, 30 °C), t_R (minor) = 14.3 min, t_R (major) = 21.5 min;

Optical Rotation: $[\alpha]^{25}_D$ = 1.6 (*c* = 0.5, $CHCl_3$) for 88% ee;

Absolute stereochemistry was determined through analogy with **7aa**.

<Chromatogram>

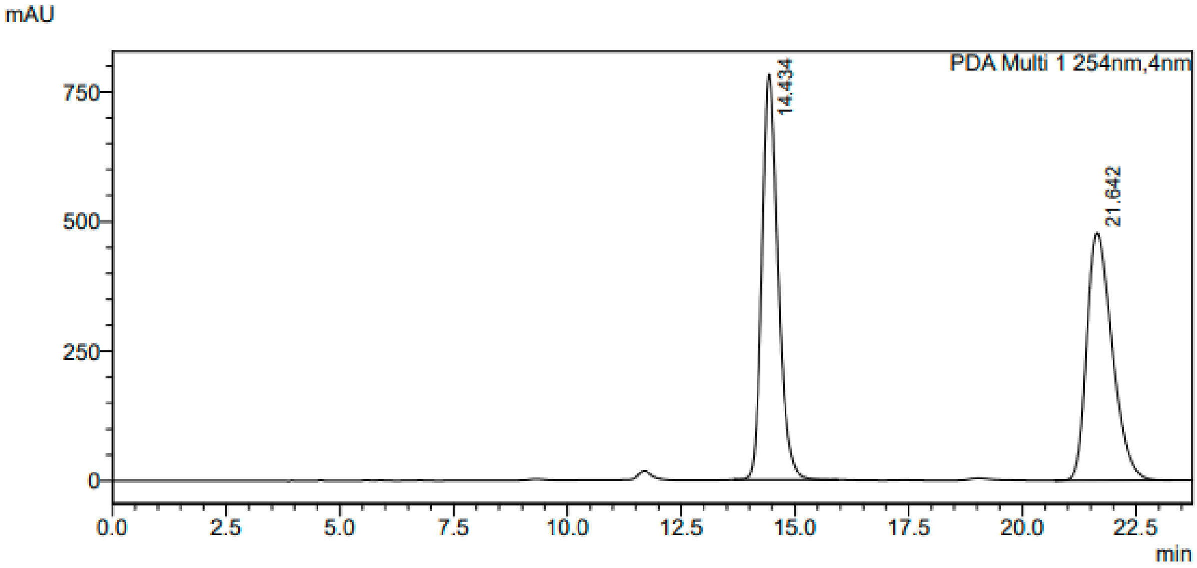

<Peak Table>

PDA Ch1 254nm

Peak#	Ret. Time	Area	Height	Conc.	Unit	Mark	Name
1	14.434	19270785	781713	0.000		M	
2	21.642	18618096	477098	0.000		M	
Total		37888881	1258811				

Supplementary Figure 96. HPLC spectrum of racemic-**7ah**

<Chromatogram>

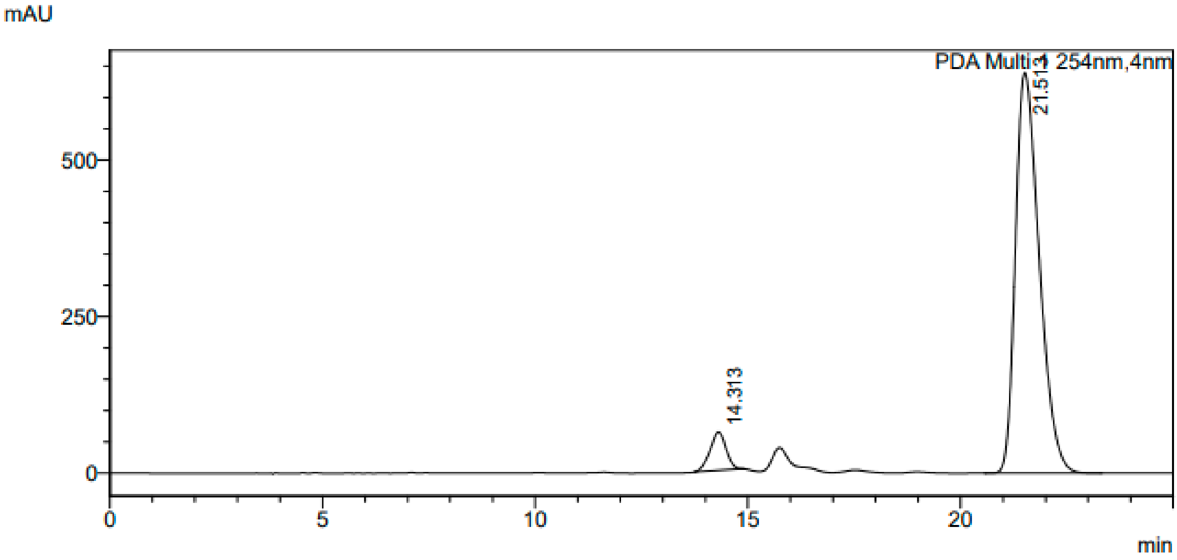

<Peak Table>

PDA Ch1 254nm

Peak#	Ret. Time	Area	Height	Conc.	Unit	Mark	Name
1	14.313	1609555	61169	0.000		M	
2	21.513	24974307	639452	0.000		M	
Total		26583862	700622				

Supplementary Figure 97. HPLC spectrum of (*S*)-**7ah**

(*S*,*Z*)-3-(1-phenyl-4-(4,4,5,5-tetramethyl-1,3,2-dioxaborolan-2-yl)butylidene)-4-((*E*)-styryl)-1-tosylpyrrolidine (7ai)

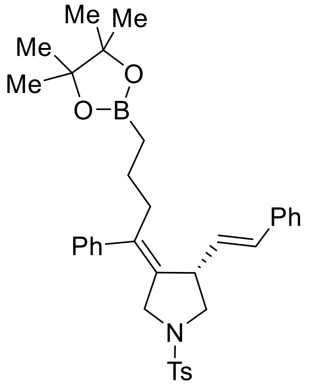

Chemical Formula: $C_{35}H_{42}BNO_4S$
Exact Mass: 583.2928

7ai was prepared according to general procedure **2.2** using (*R*,*E*)-**4a** (0.1 mmol, 47.4 mg) and methyl 2-(3-bromopropyl)-4,4,5,5-tetramethyl-1,3,2-dioxaborolane **5i** (0.2 mmol, 49.8 mg). Purification by silica gel column chromatography (PE/EA = 10/1) gave **7ai** as a colorless oil (29.8 mg, 51% yield).

^{1}H NMR (600 MHz, $CDCl_3$): δ 7.67–7.61 (m, 2H), 7.34–7.25 (m, 9H), 7.25–7.21 (m, 1H), 7.08–7.02 (m, 2H), 6.43 (dd, *J* = 15.8, 1.0 Hz, 1H), 6.03 (dd, *J* = 15.8, 7.9 Hz, 1H), 3.84 (d, *J* = 14.2 Hz, 1H), 3.72–3.66 (m, 1H), 3.57 (d, *J* = 14.3 Hz, 1H), 3.40 (d, *J* = 4.7 Hz, 2H), 2.40 (s, 3H), 2.37–2.30 (m, 2H), 1.38–1.27 (m, 2H), 1.15 (d, *J* = 2.4 Hz, 12H), 0.71–0.62 (m, 2H);

^{13}C NMR (151 MHz, $CDCl_3$) δ 143.5, 141.4, 137.7, 136.8, 132.8, 132.6, 130.1, 129.6, 129.5, 128.4, 128.3, 127.7, 127.5, 127.3, 126.9, 126.2, 82.8, 53.8, 50.7, 44.3, 37.0, 24.7, 24.6, 22.3, 21.4;

HRMS: (ESI) calcd for $C_{35}H_{43}BNO_4S^+$ ($[M+H]^+$): 584.3000; found: 584.3001;

HPLC conditions: AD-H column (15% ^{i}PrOH in hexane, 1.0 mL/min, λ = 254 nm, 30 °C), t_R (minor) = 5.9 min, t_R (major) = 7.4 min;

Optical Rotation: $[\alpha]^{25}_{D}$ = 0.1 (*c* = 0.67, $CHCl_3$) for 90% ee;

Absolute stereochemistry was determined through analogy with **7aa**.

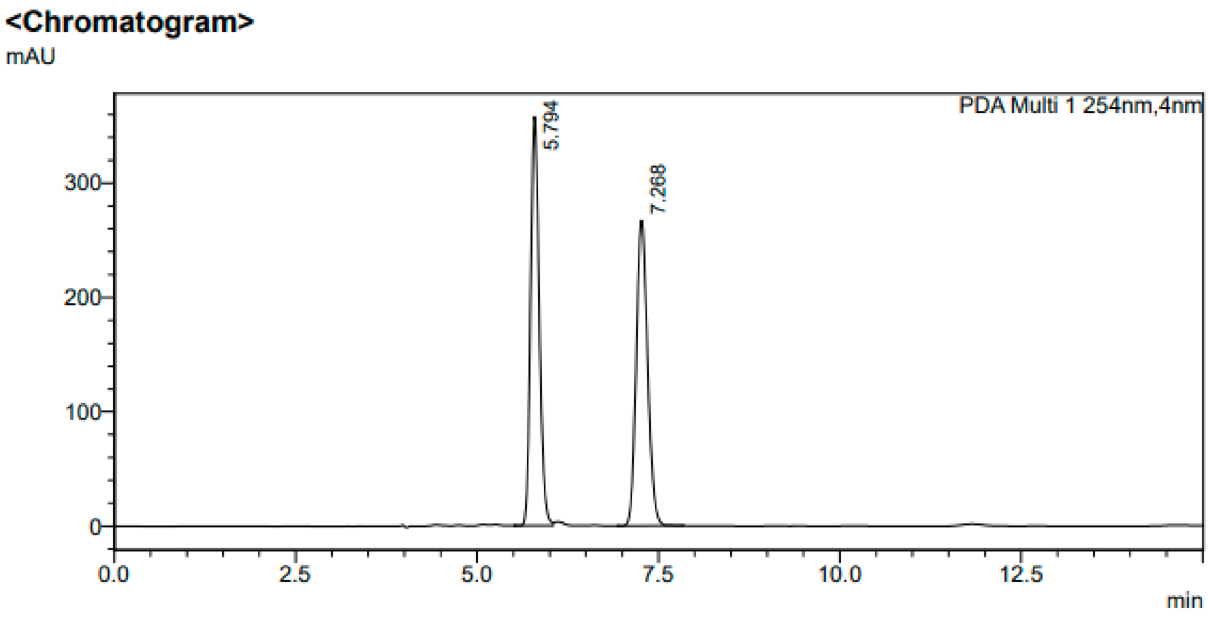

<Peak Table>

PDA Ch1 254nm

Peak#	Ret. Time	Area	Height	Conc.	Unit	Mark	Name
1	5.794	2941194	357607	0.000		M	
2	7.268	2929591	266973	0.000		M	
Total		5870784	624580				

Supplementary Figure 98. HPLC spectrum of racemic-**7ai**

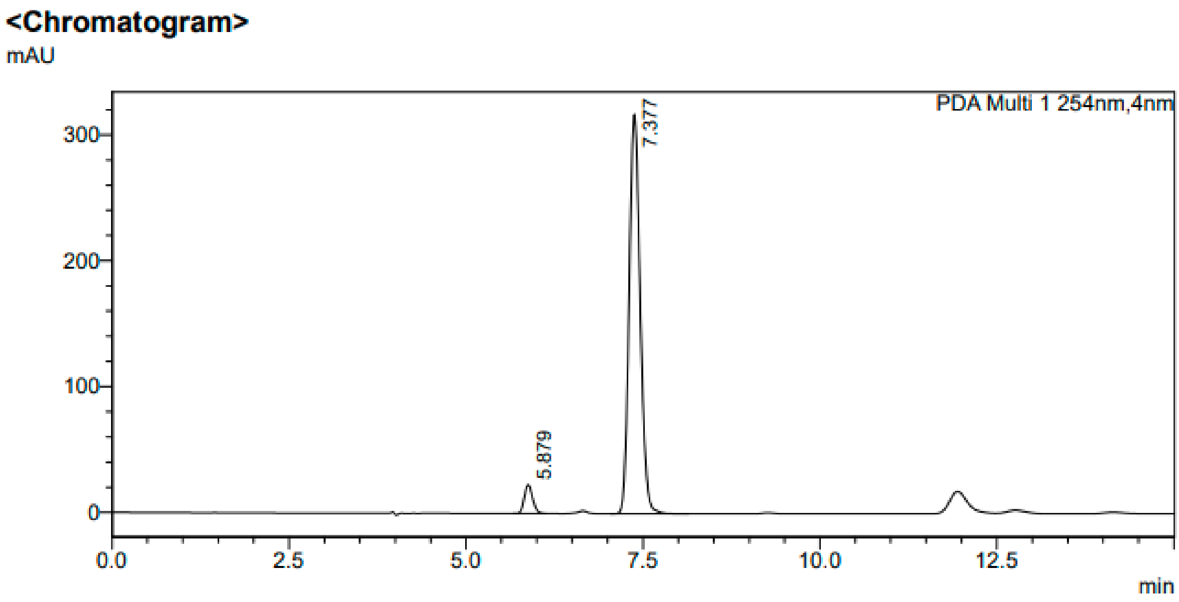

<Peak Table>

PDA Ch1 254nm

Peak#	Ret. Time	Area	Height	Conc.	Unit	Mark	Name
1	5.879	186316	22888	0.000		M	
2	7.377	3433409	317378	0.000		M	
Total		3619725	340266				

Supplementary Figure 99. HPLC spectrum of (*S*)-**7ai**

(*S*,*Z*)-3-(2-(1,3-dioxolan-2-yl)-1-phenylethylidene)-4-((*E*)-styryl)-1-tosylpyrrolidine (7aj)

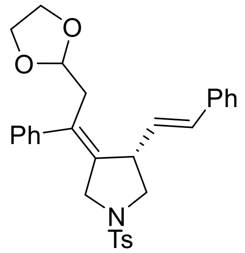

Chemical Formula: $C_{30}H_{31}NO_4S$
Exact Mass: 501.1974

7aj was prepared according to general procedure **2.2** using (*R*,*E*)-**4a** (0.1 mmol, 47.4 mg) and 2-(bromomethyl)-1,3-dioxolane **5j** (0.2 mmol, 33.4 mg). Purification by silica gel column chromatography (PE/EA = 5/1) gave **7aj** as a colorless oil (33.1 mg, 66% yield).

^{1}H NMR (600 MHz, $CDCl_3$): δ 7.63 (d, *J* = 8.2 Hz, 2H), 7.37–7.32 (m, 2H), 7.32–7.26 (m, 7H), 7.25–7.20 (m, 1H), 7.12–7.07 (m, 2H), 6.44 (d, *J* = 15.8 Hz, 1H), 6.09 (dd, *J* = 15.8, 7.9 Hz, 1H), 4.66 (t, *J* = 5.2 Hz, 1H), 3.84–3.76 (m, 2H), 3.76–3.71 (m, 2H), 3.69–3.62 (m, 2H), 3.62–3.57 (m, 1H), 3.42 (dd, *J* = 9.9, 6.8 Hz, 1H), 3.37 (dd, *J* = 9.9, 3.1 Hz, 1H), 2.75 (dd, *J* = 14.2, 5.3 Hz, 1H), 2.67 (dd, *J* = 14.2, 5.0 Hz, 1H), 2.41 (s, 3H); ^{13}C NMR (151 MHz, $CDCl_3$): δ 143.7, 140.8, 136.9, 136.2, 132.5, 132.2, 130.7, 129.6, 129.3, 128.6, 128.5, 127.9, 127.6, 127.4, 127.3, 126.3, 102.7, 64.8, 64.7, 54.0, 51.1, 44.5, 39.0, 21.5;

HRMS: (ESI) calcd for $C_{30}H_{32}N_1O_4S^+$ ($[M+H]^+$): 502.2047; found: 502.2049;

HPLC conditions: OD-H column (20% ^{i}PrOH in hexane, 1.0 mL/min, λ = 254 nm, 30 °C), t_R (minor) = 14.1 min, t_R (major) = 16.1 min;

Optical Rotation: $[\alpha]^{25}_D$ = 22.7 (*c* = 1.00, $CHCl_3$) for 94% ee;

Absolute stereochemistry was determined through analogy with **7aa**.

<Chromatogram>

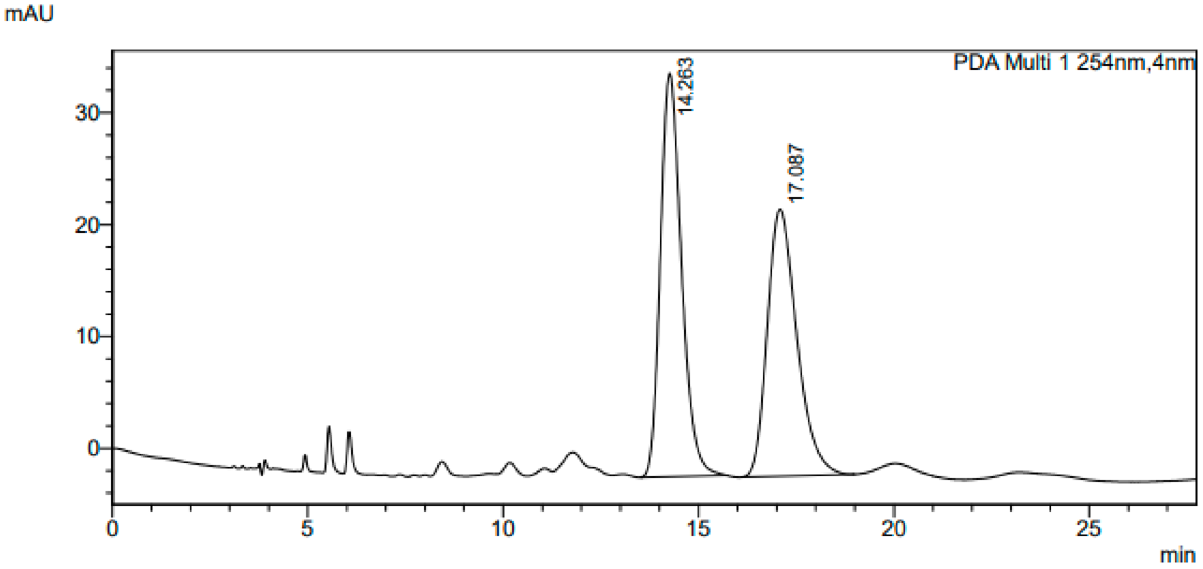

<Peak Table>

PDA Ch1 254nm

Peak#	Ret. Time	Area	Height	Conc.	Unit	Mark	Name
1	14.263	1354533	36041	0.000		M	
2	17.087	1255655	23863	0.000		M	
Total		2610188	59904				

Supplementary Figure 100. HPLC spectrum of racemic-**7aj**

<Chromatogram>

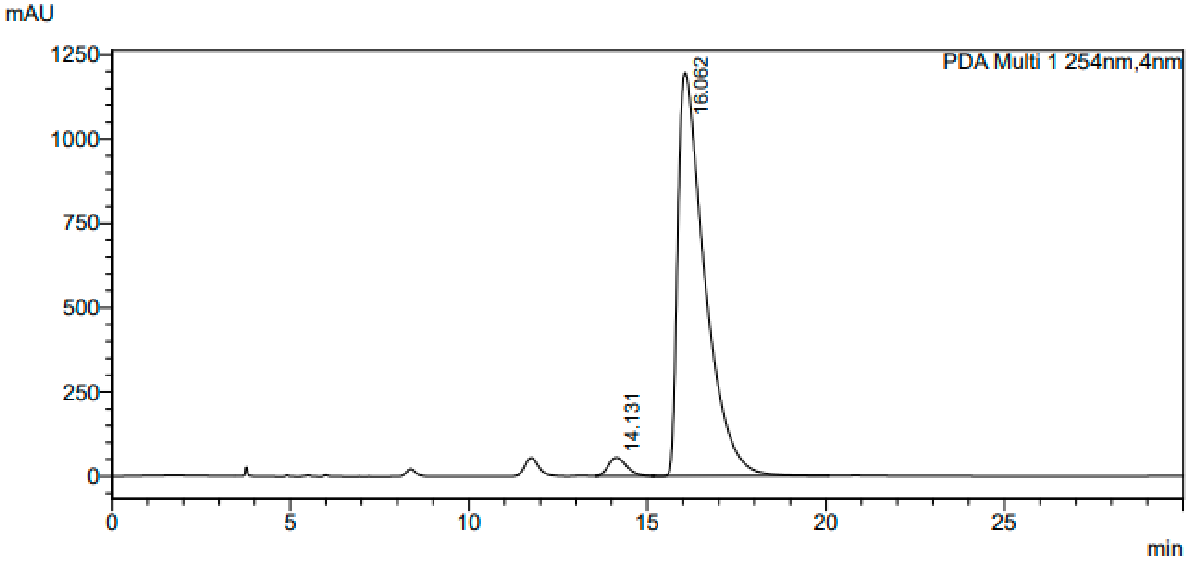

<Peak Table>

PDA Ch1 254nm

Peak#	Ret. Time	Area	Height	Conc.	Unit	Mark	Name
1	14.131	1951909	54110	0.000		M	
2	16.062	62611349	1195132	0.000		M	
Total		64563258	1249243				

Supplementary Figure 101. HPLC spectrum of (*S*)-**7aj**

2-((*Z*)-3-phenyl-3-((*S*)-4-((*E*)-styryl)-1-tosylpyrrolidin-3-ylidene)propyl)isoindoline-1,3-dione (7ak)

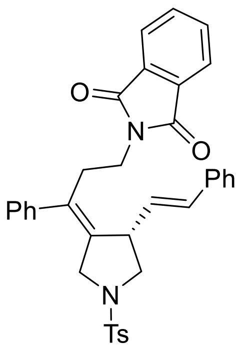

Chemical Formula: $C_{36}H_{32}N_2O_4S$
Exact Mass: 588.2083

7ak was prepared according to general procedure **2.2** using (*R*,*E*)-**4a** (0.1 mmol, 47.4 mg) and 2-(2-bromoethyl)isoindoline-1,3-dione **5k** (0.2 mmol, 50.8 mg). Purification by silica gel column chromatography (PE/EA = 3/1) gave **7ak** as a colorless oil (42.4 mg, 72% yield).

^{1}H NMR (600 MHz, $CDCl_3$): δ 7.66–7.59 (m, 4H), 7.59–7.55 (m, 2H), 7.29–7.25 (m, 6H), 7.23–7.18 (m, 3H), 7.13–7.06 (m, 3H), 6.50 (d, *J* = 15.8 Hz, 1H), 6.03 (dd, *J* = 15.8, 8.0 Hz, 1H), 3.90 (dd, *J* = 14.5, 1.5 Hz, 1H), 3.76–3.67 (m, 1H), 3.66–3.53 (m, 3H), 3.40 (dd, *J* = 10.1, 6.9 Hz, 1H), 3.33 (dd, *J* = 10.1, 2.9 Hz, 1H), 2.89–2.81 (m, 1H), 2.76–2.69 (m, 1H), 2.39 (s, 3H);

^{13}C NMR (151 MHz, $CDCl_3$): δ 168.1, 143.7, 140.2, 136.7, 136.0, 133.9, 133.7, 132.8, 131.8, 130.9, 129.7, 129.1, 128.5, 128.5, 127.8, 127.6, 127.5, 127.3, 126.4, 122.9, 53.9, 51.0, 44.8, 36.7, 32.6, 21.6;

HRMS: (ESI) calcd for $C_{36}H_{33}N_2O_4S^+$ ($[M+H]^+$): 589.2156; found: 589.2155;

HPLC conditions: AD-H column (40% ^{i}PrOH in hexane, 1.0 mL/min, λ = 254 nm, 30 °C), t_R (major) = 13.0 min, t_R (minor) = 23.8 min;

Optical Rotation: $[\alpha]^{25}_D$ = 15.7 (*c* = 1.33, $CHCl_3$) for 94% ee;

Absolute stereochemistry was determined through analogy with **7aa**.

<Chromatogram>

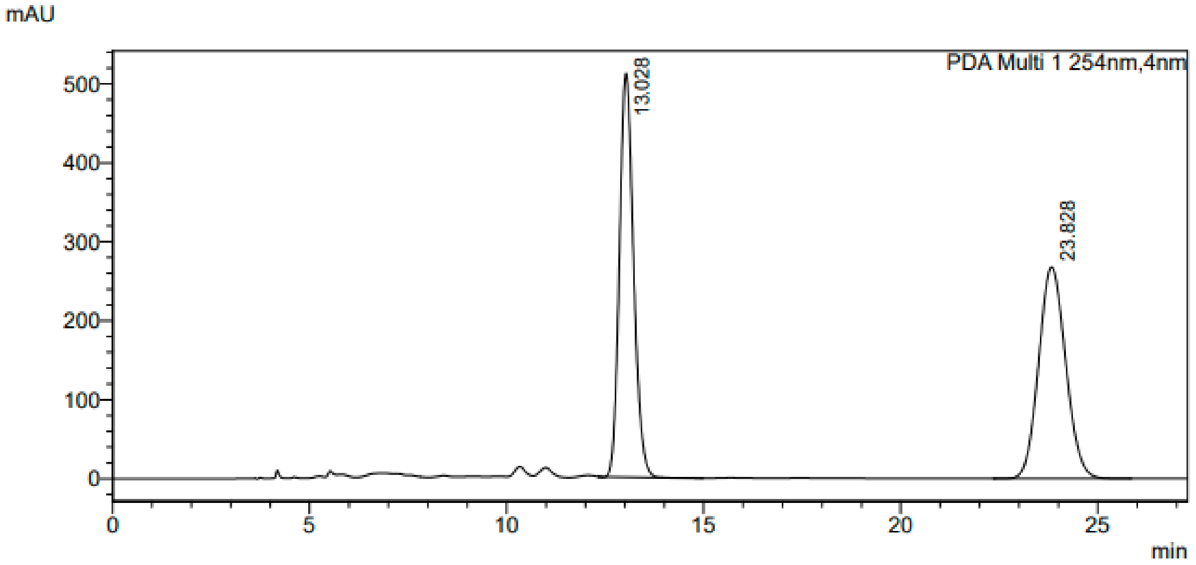

<Peak Table>

PDA Ch1 254nm

Peak#	Ret. Time	Area	Height	Conc.	Unit	Mark	Name
1	13.028	12723963	510845	0.000		M	
2	23.828	12773004	267649	0.000		M	
Total		25496967	778493				

Supplementary Figure 102. HPLC spectrum of racemic-**7ak**

<Chromatogram>

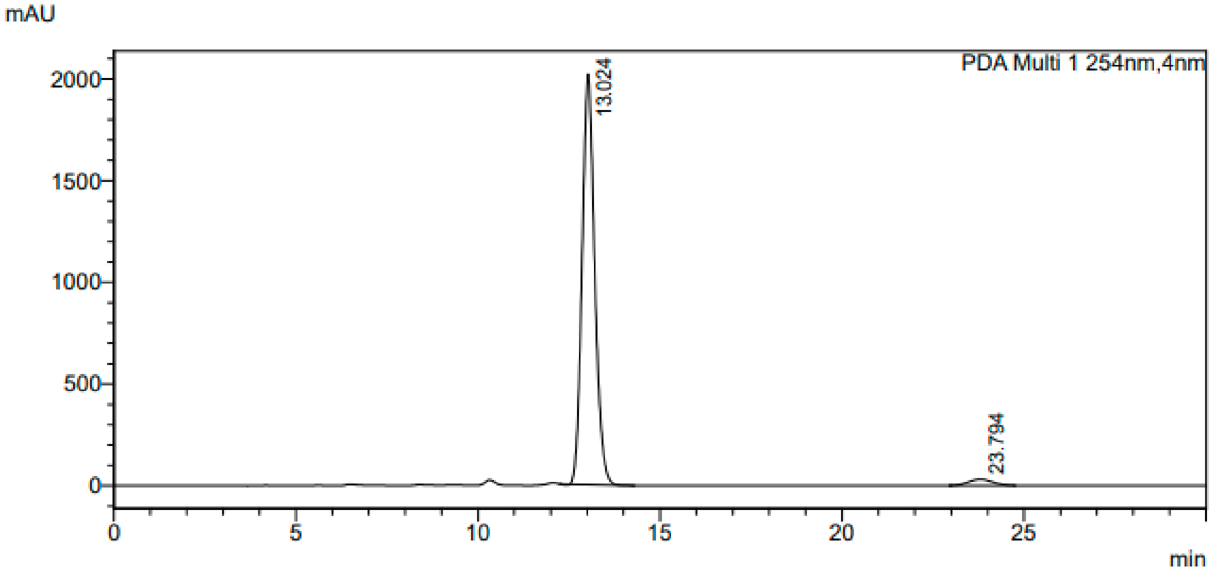

<Peak Table>

PDA Ch1 254nm

Peak#	Ret. Time	Area	Height	Conc.	Unit	Mark	Name
1	13.024	48633193	2018170	0.000		M	
2	23.794	1426458	31284	0.000		M	
Total		50059652	2049454				

Supplementary Figure 103. HPLC spectrum of (*S*)-**7ak**

***tert*-butyl((*Z*)-4-phenyl-4-((*S*)-4-((*E*)-styryl)-1-tosylpyrrolidin-3-ylidene)butyl) carbamate (7al)**

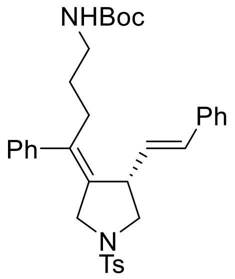

Chemical Formula: $C_{34}H_{40}N_2O_4S$
Exact Mass: 572.2709

7al was prepared according to general procedure **2.2** using (*R*,*E*)-**4a** (0.1 mmol, 47.4 mg) and *tert*-butyl (3-bromopropyl)carbamate **5l** (0.2 mmol, 50.8 mg). Purification by silica gel column chromatography (PE/EA = 3/1) gave **7al** as a colorless oil (38.6 mg, 67% yield).

^{1}H NMR (600 MHz, $CDCl_3$): δ 7.63 (d, *J* = 8.0 Hz, 2H), 7.35–7.26 (m, 9H), 7.25–7.22 (m, 1H), 7.06–7.01 (m, 2H), 6.42 (d, *J* = 15.8 Hz, 1H), 6.03 (dd, *J* = 15.8, 8.3 Hz, 1H), 4.24 (t, *J* = 6.1 Hz, 1H), 3.83 (d, *J* = 14.3 Hz, 1H), 3.68–3.61 (m, 1H), 3.53 (d, *J* = 14.3 Hz, 1H), 3.40 (dd, *J* = 10.0, 6.9 Hz, 1H), 3.34 (dd, *J* = 10.0, 3.0 Hz, 1H), 3.00–2.88 (m, 2H), 2.41 (s, 3H), 2.39–2.29 (m, 2H), 1.39 (s, 8H), 1.36–1.25 (m, 3H);

^{13}C NMR (151 MHz, $CDCl_3$): δ 155.8, 143.7, 141.0, 137.0, 136.6, 133.5, 132.5, 130.4, 129.6, 129.5, 128.6, 128.6, 127.8, 127.6, 127.5, 127.2, 126.2, 79.1, 54.0, 51.0, 44.7, 40.2, 31.6, 28.4, 28.3, 21.5;

HRMS: (ESI) calcd for $C_{34}H_{41}N_2O_4S^+$ ($[M+H]^+$): 573.2782; found: 573.2779;

HPLC conditions: IA-H column (30% ^{i}PrOH in hexane, 1.0 mL/min, λ = 254 nm, 30 °C), t_R (minor) = 5.8 min, t_R (major) = 6.3 min;

Optical Rotation: $[\alpha]^{25}_D$ = -8.5 (*c* = 1.00, $CHCl_3$) for 93% ee;

Absolute stereochemistry was determined through analogy with **7aa**.

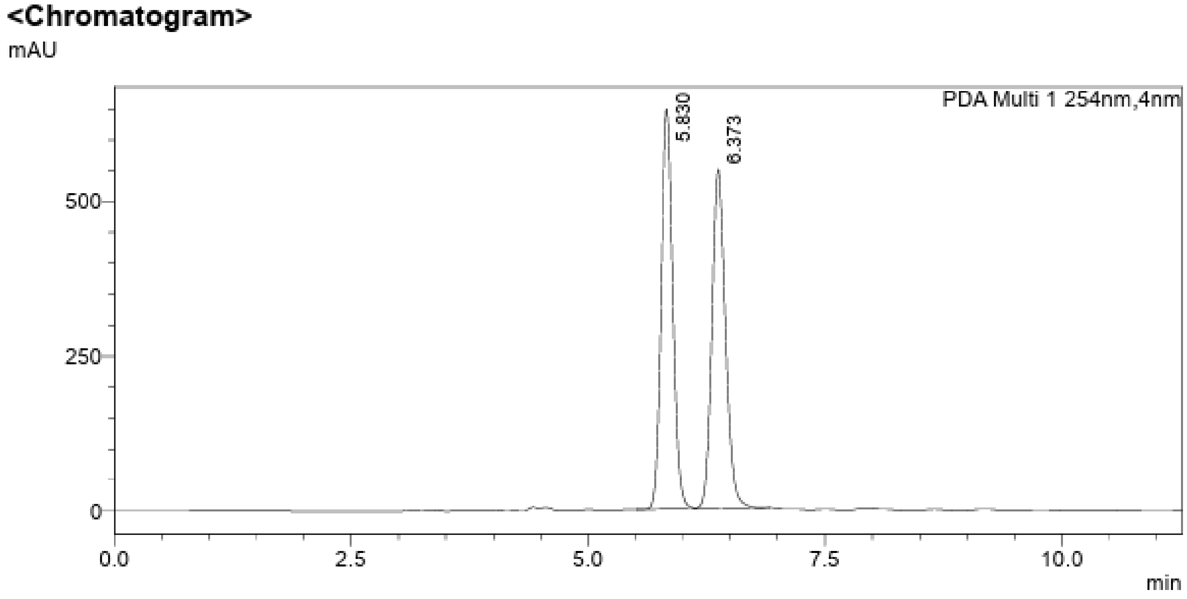

<Peak Table>

PDA Ch1 254nm

Peak#	Ret. Time	Area	Height	Conc.	Unit	Mark	Name
1	5.830	5560222	646808	0.000		M	
2	6.373	5533843	548359	0.000		V M	
Total		11094065	1195167				

Supplementary Figure 104. HPLC spectrum of racemic-**7al**

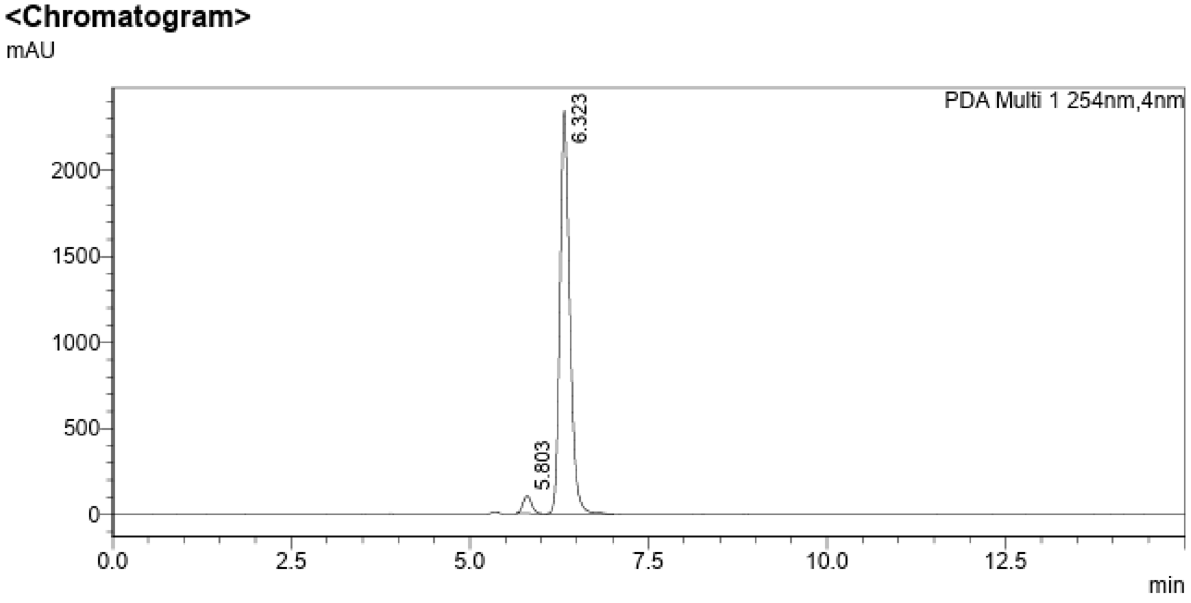

<Peak Table>

PDA Ch1 254nm

Peak#	Ret. Time	Area	Height	Conc.	Unit	Mark	Name
1	5.803	854545	102494	0.000		M	
2	6.323	22334284	2343625	0.000		M	
Total		23188829	2446119				

Supplementary Figure 105. HPLC spectrum of (*S*)-**7al**

benzyl((*Z*)-4-phenyl-4-((*S*)-4-((*E*)-styryl)-1-tosylpyrrolidin-3-ylidene)butyl) carbamate (7am)

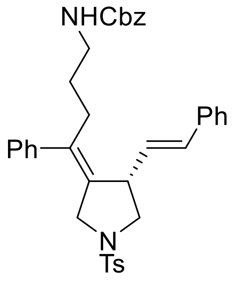

7am was prepared according to general procedure **2.2** using (*R*,*E*)-**4a** (0.1 mmol, 47.4 mg) and benzyl (3-bromopropyl)carbamate **5m** (0.2 mmol, 54.4 mg). Purification by silica gel column chromatography (PE/EA = 3/1) gave **7am** as a colorless oil (44.3 mg, 73% yield).

^{1}H NMR (600 MHz, $CDCl_3$): δ 7.63 (d, *J* = 8.2 Hz, 2H), 7.36–7.26 (m, 14H), 7.24–7.21 (m, 1H), 7.05–7.01 (m, 2H), 6.38 (d, *J* = 15.8 Hz, 1H), 6.01 (dd, *J* = 15.8, 8.4 Hz, 1H), 5.07–4.95 (m, 2H), 4.45 (t, *J* = 5.8 Hz, 1H), 3.82 (dd, *J* = 14.4, 1.7 Hz, 1H), 3.61 (d, *J* = 8.4 Hz, 1H), 3.53 (d, *J* = 14.3 Hz, 1H), 3.40 (dd, *J* = 10.0, 7.0 Hz, 1H), 3.31 (dd, *J* = 10.1, 3.1 Hz, 1H), 3.05–2.97 (m, 2H), 2.41 (s, 3H), 2.39–2.28 (m, 2H), 1.40–1.27 (m, 2H);

^{13}C NMR (151 MHz, $CDCl_3$): δ 156.2, 143.7, 140.9, 136.9, 136.6, 136.6, 133.7, 132.6, 130.6, 129.7, 129.4, 128.7, 128.5, 128.2, 127.9, 127.7, 127.5, 127.3, 126.3, 66.6, 54.0, 51.1, 44.7, 40.7, 31.5, 28.3, 21.6;

HRMS: (ESI) calcd for $C_{37}H_{39}N_2O_4S^+$ ($[M+H]^+$): 607.2625; found: 607.2624;

HPLC conditions: OD-H column (30% ^{i}PrOH in hexane, 1.0 mL/min, λ = 254 nm, 30 °C), t_R (major) = 18.0 min, t_R (minor) = 27.0 min;

Optical Rotation: $[\alpha]^{25}_{D}$ = -8.3 (*c* = 1.63, $CHCl_3$) for 94% ee;

Absolute stereochemistry was determined through analogy with **7aa**.

<Chromatogram>

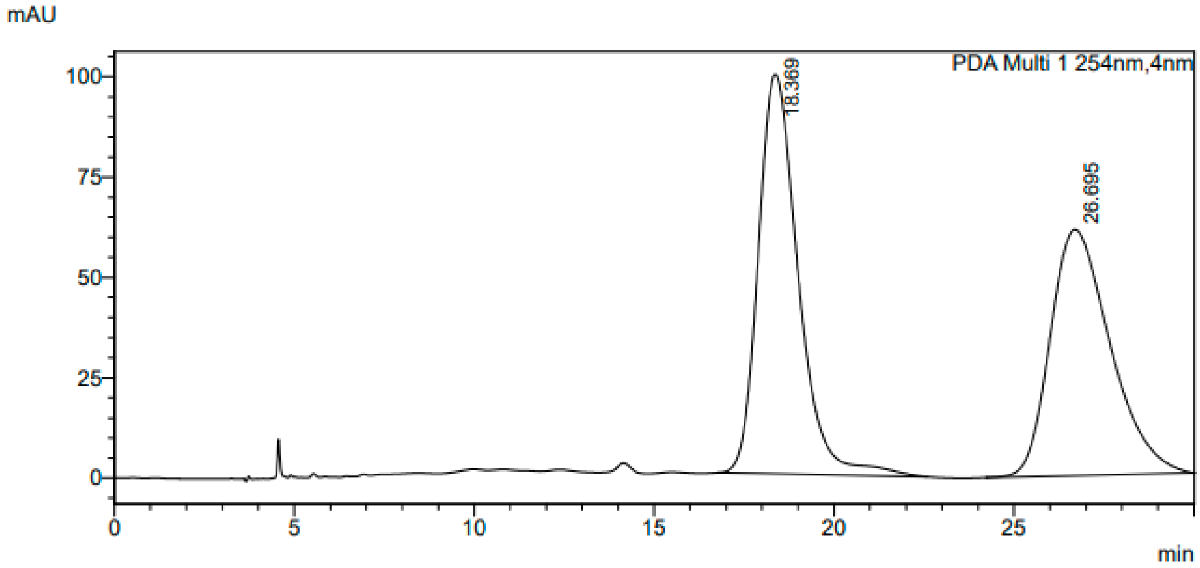

<Peak Table>

PDA Ch1 254nm

Peak#	Ret. Time	Area	Height	Conc.	Unit	Mark	Name
1	18.369	7681888	99545	0.000		M	
2	26.695	7106211	61258	0.000		M	
Total		14788099	160803				

Supplementary Figure 106. HPLC spectrum of racemic-**7am**

<Chromatogram>

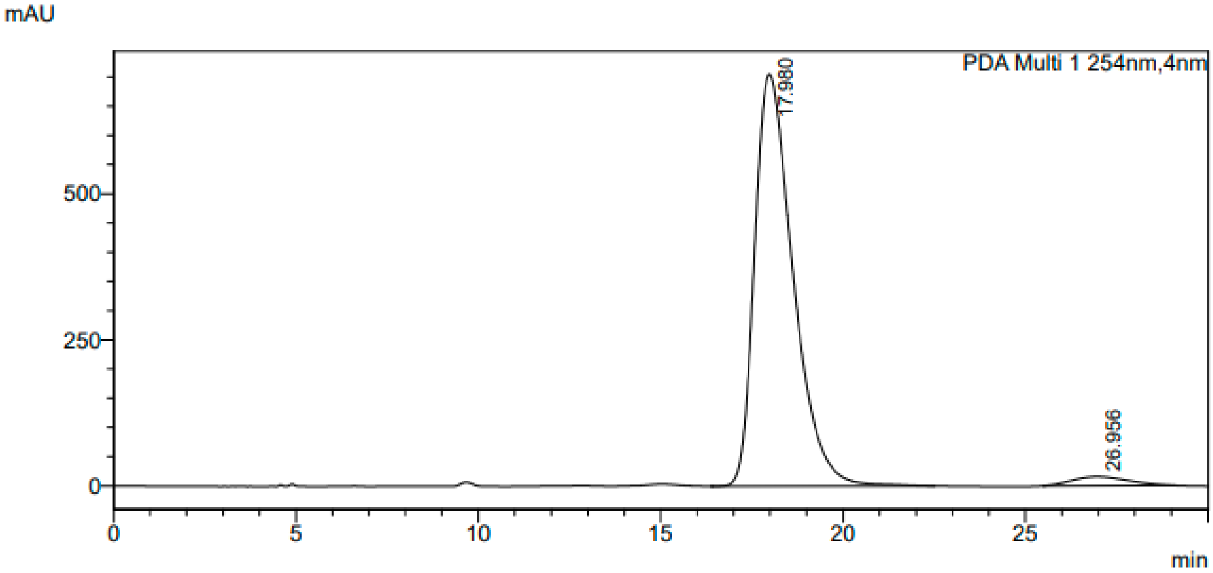

<Peak Table>

PDA Ch1 254nm

Peak#	Ret. Time	Area	Height	Conc.	Unit	Mark	Name
1	17.980	51644609	705071	0.000		M	
2	26.956	1622567	15378	0.000		M	
Total		53267176	720449				

Supplementary Figure 107. HPLC spectrum of (*S*)-**7am**

(*S*,*Z*)-3-(5-methyl-1-phenylhex-4-en-1-ylidene)-4-((*E*)-styryl)-1-tosylpyrrolidine (7an)

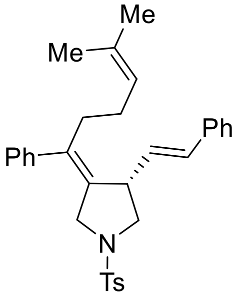

Chemical Formula: $C_{32}H_{35}NO_2S$
Exact Mass: 497.2389

7an was prepared according to general procedure **2.2** using (*R*,*E*)-**4a** (0.1 mmol, 47.4 mg) and 5-bromo-2-methylpent-2-ene **5n** (0.2 mmol, 32.6 mg). Purification by silica gel column chromatography (PE/EA = 10/1) gave **7an** as a colorless oil (30.4 mg, 61% yield).

^{1}H NMR (600 MHz, $CDCl_3$): δ 7.67–7.60 (m, 2H), 7.36–7.27 (m, 9H), 7.25–7.21 (m, 1H), 7.07–7.00 (m, 2H), 6.41 (d, *J* = 15.8 Hz, 1H), 6.03 (dd, *J* = 15.8, 8.0 Hz, 1H), 4.92 (t, *J* = 7.1 Hz, 2H), 3.85 (dd, *J* = 14.3, 1.6 Hz, 1H), 3.72–3.65 (m, 1H), 3.57 (d, *J* = 14.3 Hz, 1H), 3.41 (dd, *J* = 10.0, 6.9 Hz, 1H), 3.36 (dd, *J* = 10.0, 3.0 Hz, 1H), 2.40 (s, 3H), 2.39–2.28 (m, 2H), 1.89-1.77 (m, 2H);

^{13}C NMR (151 MHz, $CDCl_3$): δ 143.6, 141.2, 137.5, 136.8, 133.0, 132.8, 131.9, 130.4, 129.6, 129.4, 128.5, 128.4, 127.8, 127.6, 127.4, 127.1, 126.2, 123.4, 53.9, 50.9, 44.5, 34.3, 26.3, 25.5, 21.5, 17.5;

HRMS: (ESI) calcd for $C_{32}H_{36}NO_2S^+$ ($[M+H]^+$): 498.2461; found: 498.2469;

HPLC conditions: AD-H column (15% ^{i}PrOH in hexane, 1.0 mL/min, λ = 254 nm, 30 °C), t_R (minor) = 6.4 min, t_R (major) = 7.1 min;

Optical Rotation: $[\alpha]^{25}_D$ = 2.7 (*c* = 1.00, $CHCl_3$) for 95% ee;

Absolute stereochemistry was determined through analogy with **7aa**.

<Chromatogram>

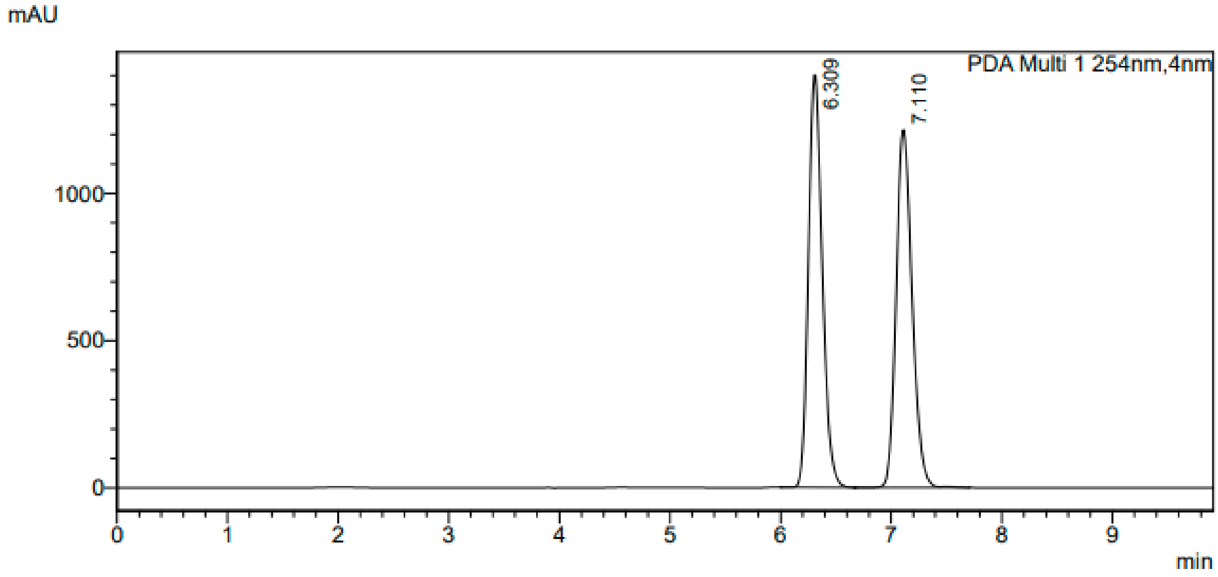

<Peak Table>

PDA Ch1 254nm

Peak#	Ret. Time	Area	Height	Conc.	Unit	Mark	Name
1	6.309	12217438	1399594	0.000		M	
2	7.110	12213639	1212884	0.000		M	
Total		24431077	2612478				

Supplementary Figure 108. HPLC spectrum of racemic-**7an**

<Chromatogram>

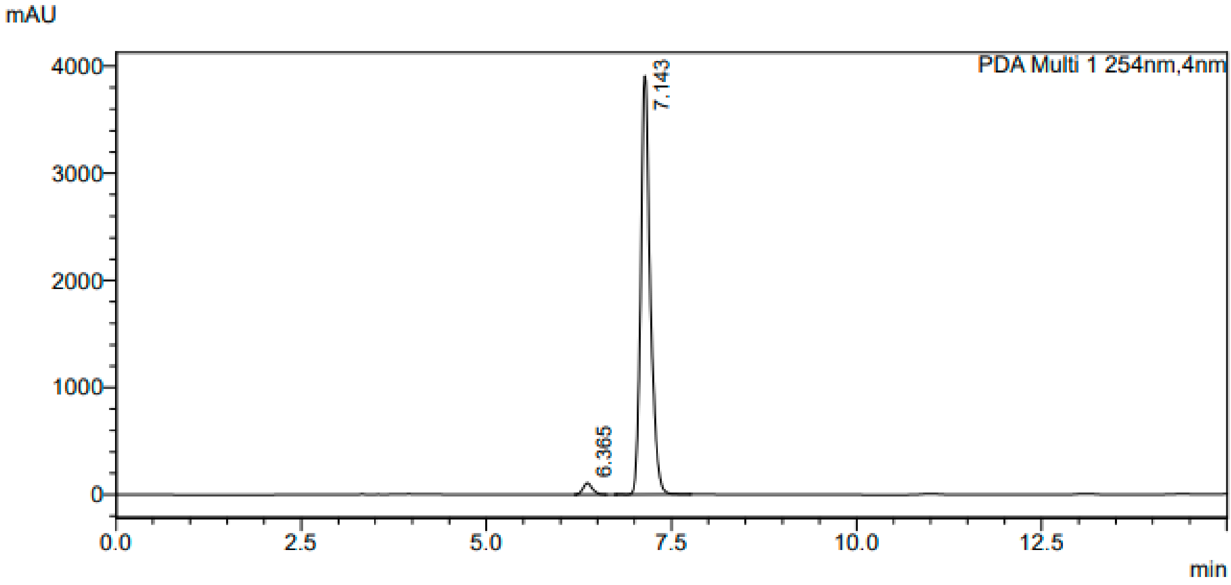

<Peak Table>

PDA Ch1 254nm

Peak#	Ret. Time	Area	Height	Conc.	Unit	Mark	Name
1	6.365	938654	107761	0.000		M	
2	7.143	35291374	3905959	0.000		M	
Total		36230028	4013719				

Supplementary Figure 109. HPLC spectrum of (*S*)-**7an**

(*S*,*Z*)-3-(1-phenyl-6-(triisopropylsilyl)hex-5-yn-1-ylidene)-4-((*E*)-styryl)-1-tosylpyrrolidine (7ao)

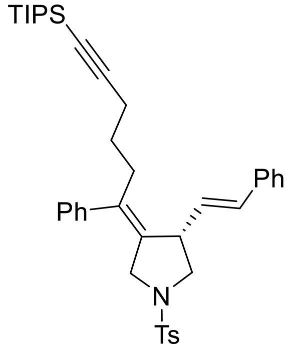

Chemical Formula: $C_{40}H_{51}NO_2SSi$
Exact Mass: 637.3410

7ao was prepared according to general procedure **2.2** using (*R*,*E*)-**4a** (0.1 mmol, 47.4 mg) and (5-bromopent-1-yn-1-yl)triisopropylsilane **5o** (0.2 mmol, 60.7 mg). Purification by silica gel column chromatography (PE/EA = 15/1) gave **7ao** as a colorless oil (42.1 mg, 66% yield).

^{1}H NMR (600 MHz, $CDCl_3$): δ 7.63 (d, *J* = 8.1 Hz, 2H), 7.35–7.30 (m, 3H), 7.30–7.26 (m, 5H), 7.25–7.21 (m, 1H), 7.07–7.00 (m, 2H), 6.41 (d, *J* = 15.8 Hz, 1H), 6.02 (dd, *J* = 15.9, 7.6 Hz, 1H), 3.83 (dd, *J* = 14.5, 1.6 Hz, 1H), 3.75-3.70 (m, 1H), 3.58 (d, *J* = 14.3 Hz, 1H), 3.45-3.35 (m, 2H), 2.57-2.41 (m, 2H), 2.39 (s, 3H), 2.15-2.05 (m, 2H), 1.45-1.31 (m, 2H), 1.11-0.87 (m, 21H);

^{13}C NMR (151 MHz, $CDCl_3$): δ 143.6, 141.0, 137.0, 136.7, 133.6, 132.8, 130.3, 129.6, 129.5, 128.5, 127.8, 127.5, 127.5, 127.2, 126.2, 108.3, 80.6, 53.8, 50.8, 44.3, 33.5, 26.8, 21.5, 19.6, 18.6, 11.2;

HRMS: (ESI) calcd for $C_{40}H_{52}NO_2SSi^+$ ($[M+H]^+$): 638.3483; found: 638.3482;

HPLC conditions: AD-H column (5% ^{i}PrOH in hexane, 1.0 mL/min, λ = 254 nm, 30 °C), t_R (minor) = 5.5 min, t_R (major) = 5.9 min;

Optical Rotation: $[\alpha]^{25}_D$ = 0.4 (*c* = 1.25, $CHCl_3$) for 91% ee;

Absolute stereochemistry was determined through analogy with **7aa**.

<Chromatogram>

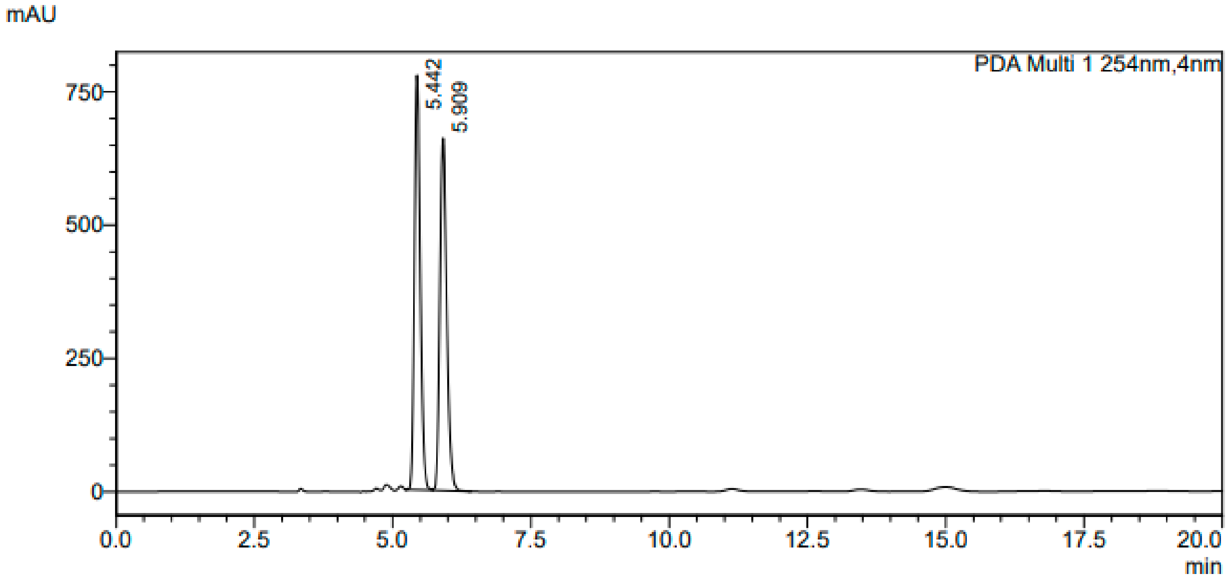

<Peak Table>

PDA Ch1 254nm

Peak#	Ret. Time	Area	Height	Conc.	Unit	Mark	Name
1	5.442	5692715	777082	0.000		M	
2	5.909	5706842	660402	0.000		V M	
Total		11399558	1437484				

Supplementary Figure 110. HPLC spectrum of racemic-**7ao**

<Chromatogram>

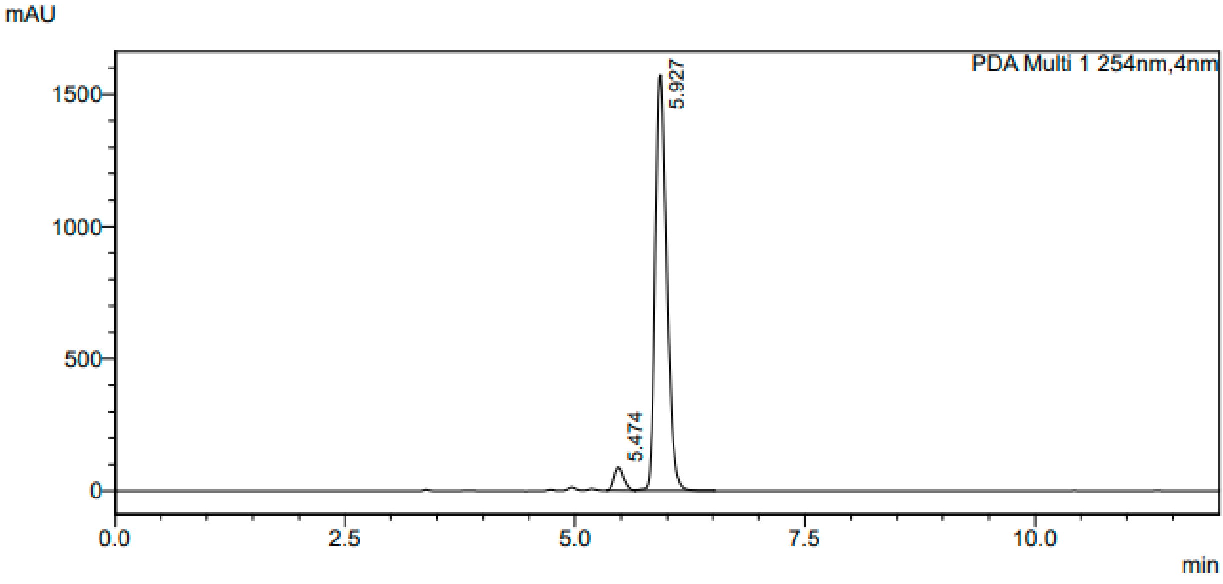

<Peak Table>

PDA Ch1 254nm

Peak#	Ret. Time	Area	Height	Conc.	Unit	Mark	Name
1	5.474	630115	87836	0.000		M	
2	5.927	13419394	1571727	0.000		V M	
Total		14049508	1659563				

Supplementary Figure 111. HPLC spectrum of (*S*)-**7ao**

methyl 4-((*Z*)-3-phenyl-1-((*S*)-4-((*E*)-styryl)-1-tosylpyrrolidin-3-ylidene)propyl) benzoate (7ba)

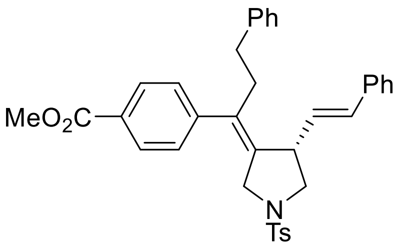

Chemical Formula: $C_{36}H_{35}NO_4S$
Exact Mass: 577.2287

7ba was prepared according to general procedure **2.2** using (*R*,*E*)-**4b** (0.1 mmol, 53.2 mg) and (2-bromoethyl)benzene **5a** (0.2 mmol, 37.0 mg). Purification by silica gel column chromatography (PE/EA = 5/1) gave **7ba** as a colorless oil (45.1 mg, 78% yield).

^{1}H NMR (600 MHz, $CDCl_3$): δ 8.05 (d, *J* = 8.2 Hz, 2H), 7.62 (d, *J* = 8.3 Hz, 2H), 7.33–7.28 (m, 6H), 7.26–7.24 (m, 1H), 7.19–7.17 (m, 2H), 7.13–7.08 (m, 3H), 6.91–6.87 (m, 2H), 6.42 (d, *J* = 15.8 Hz, 1H), 6.02 (dd, *J* = 15.8, 8.3 Hz, 1H), 3.96 (s, 3H), 3.83–3.79 (m, 1H), 3.58–3.54 (m, 1H), 3.52 (d, *J* = 14.3 Hz, 1H), 3.36 (dd, *J* = 10.0, 7.0 Hz, 1H), 3.30 (dd, *J* = 10.0, 3.4 Hz, 1H), 2.74–2.68 (m, 1H), 2.63–2.57 (m, 1H), 2.44–2.37 (m, 5H); ^{13}C NMR (151 MHz, $CDCl_3$): δ 166.8, 143.7, 136.5, 136.1, 134.8, 132.4, 130.9, 130.0, 129.7, 129.1, 128.9, 128.6, 128.3, 128.2, 127.9, 127.8, 127.7, 126.3, 126.0, 53.9, 52.2, 50.9, 44.9, 36.3, 34.1, 21.6;

HRMS: (ESI) calcd for $C_{36}H_{36}NO_4S^+$ ($[M+H]^+$): 578.2360; found: 578.2355;

HPLC conditions: AD-H column (20% ^{i}PrOH in hexane, 1.0 mL/min, λ = 254 nm, 30 °C), t_R (minor) = 18.7 min, t_R (major) = 21.2 min;

Optical Rotation: $[\alpha]^{25}_D$ = 17.7 (*c* = 1.57, $CHCl_3$) for 93% ee;

Absolute stereochemistry was determined through analogy with **7aa**.

<Chromatogram>

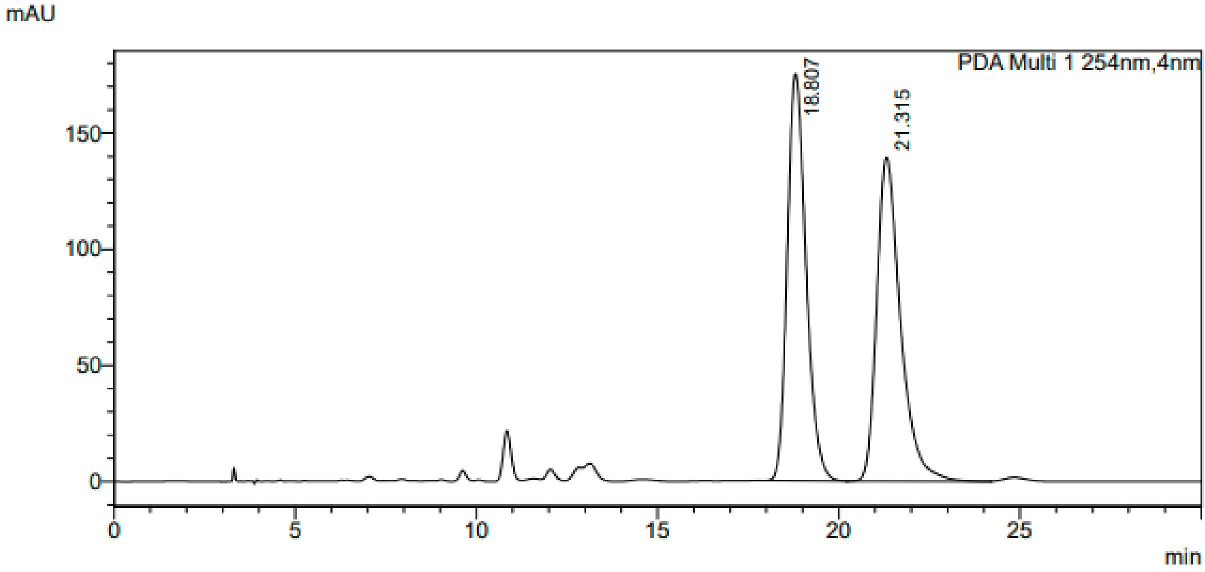

<Peak Table>

PDA Ch1 254nm

Peak#	Ret. Time	Area	Height	Conc.	Unit	Mark	Name
1	18.807	6289284	175147	0.000		M	
2	21.315	6361338	139489	0.000		M	
Total		12650622	314636				

Supplementary Figure 112. HPLC spectrum of racemic-**7ba**

<Chromatogram>

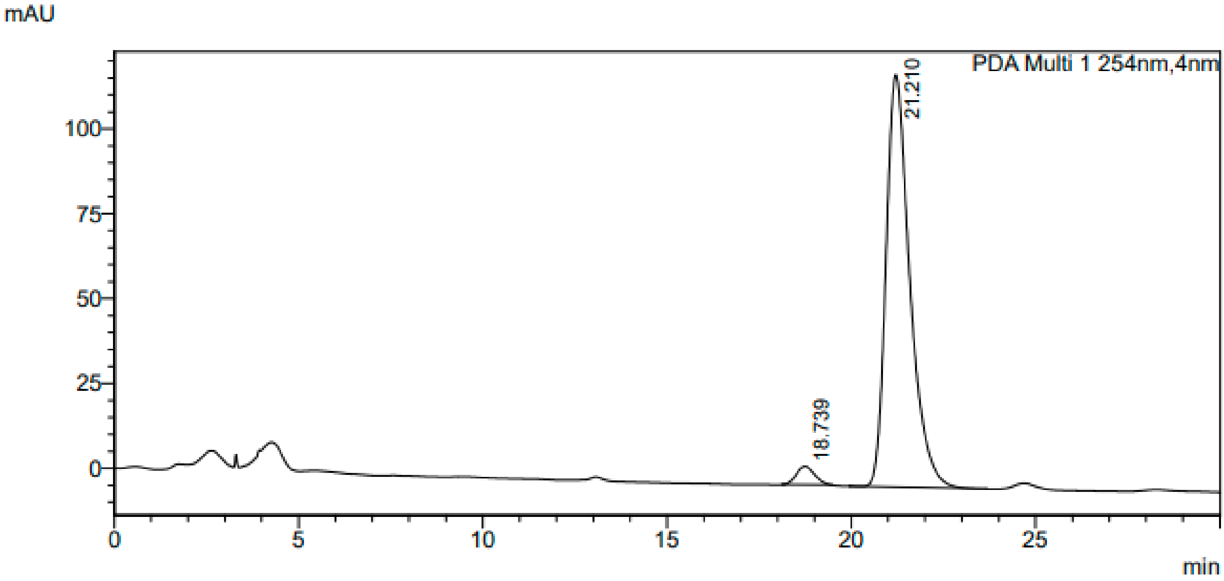

<Peak Table>

PDA Ch1 254nm

Peak#	Ret. Time	Area	Height	Conc.	Unit	Mark	Name
1	18.739	184380	5414	0.000		M	
2	21.210	5453698	121394	0.000		M	
Total		5638078	126808				

Supplementary Figure 113. HPLC spectrum of (*S*)-**7ba**

(*Z*)-4-(4-methoxyphenyl)-4-((*S*)-4-((*E*)-styryl)-1-tosylpyrrolidin-3-ylidene)butyl acetate (7cf)

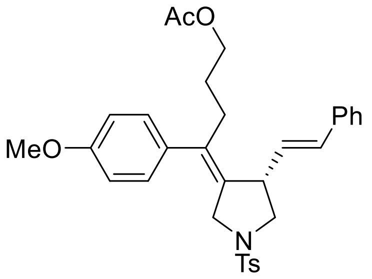

Chemical Formula: $C_{32}H_{35}NO_5S$
Exact Mass: 545.2236

7cf was prepared according to general procedure **2.2** using (*R*,*E*)-**4c** (0.1 mmol, 50.4 mg) and 3-bromopropyl acetate **5f** (0.2 mmol, 36.2 mg). Purification by silica gel column chromatography (PE/EA = 3/1) gave **7cf** as a colorless oil (39.3 mg, 72% yield).

^{1}H NMR (600 MHz, $CDCl_3$): δ 7.66–7.61 (m, 2H), 7.33–7.26 (m, 6H), 7.25–7.22 (m, 1H), 6.97 (d, *J* = 8.7 Hz, 2H), 6.87 (d, *J* = 8.7 Hz, 2H), 6.40 (d, *J* = 15.8 Hz, 1H), 6.02 (dd, *J* = 15.8, 8.2 Hz, 1H), 3.90–3.80 (m, 6H), 3.66–3.61 (m, 1H), 3.57 (d, *J* = 14.3 Hz, 1H), 3.41 (dd, *J* = 10.0, 7.0 Hz, 1H), 3.33 (dd, *J* = 10.0, 3.2 Hz, 1H), 2.44–2.32 (m, 5H), 1.88 (s, 3H), 1.51–1.45 (m, 2H);

^{13}C NMR (151 MHz, $CDCl_3$): δ 171.0, 158.7, 143.7, 136.6, 136.1, 133.6, 133.0, 132.6, 130.5, 129.6, 129.4, 128.7, 128.6, 127.8, 127.6, 126.2, 114.0, 64.0, 55.3, 54.0, 51.1, 44.6, 30.7, 26.9, 21.5, 20.8;

HRMS: (ESI) calcd for $C_{32}H_{36}NO_5S^+$ ($[M+H]^+$): 546.2309; found: 546.2304;

HPLC conditions: AD-H column (30% ^{i}PrOH in hexane, 1.0 mL/min, λ = 254 nm, 30 °C), t_R (minor) = 8.0 min, t_R (major) = 8.6 min;

Optical Rotation: $[\alpha]^{25}_D$ = 3.3 (*c* = 1.40, $CHCl_3$) for 90% ee;

Absolute stereochemistry was determined through analogy with **7aa**.

<Chromatogram>

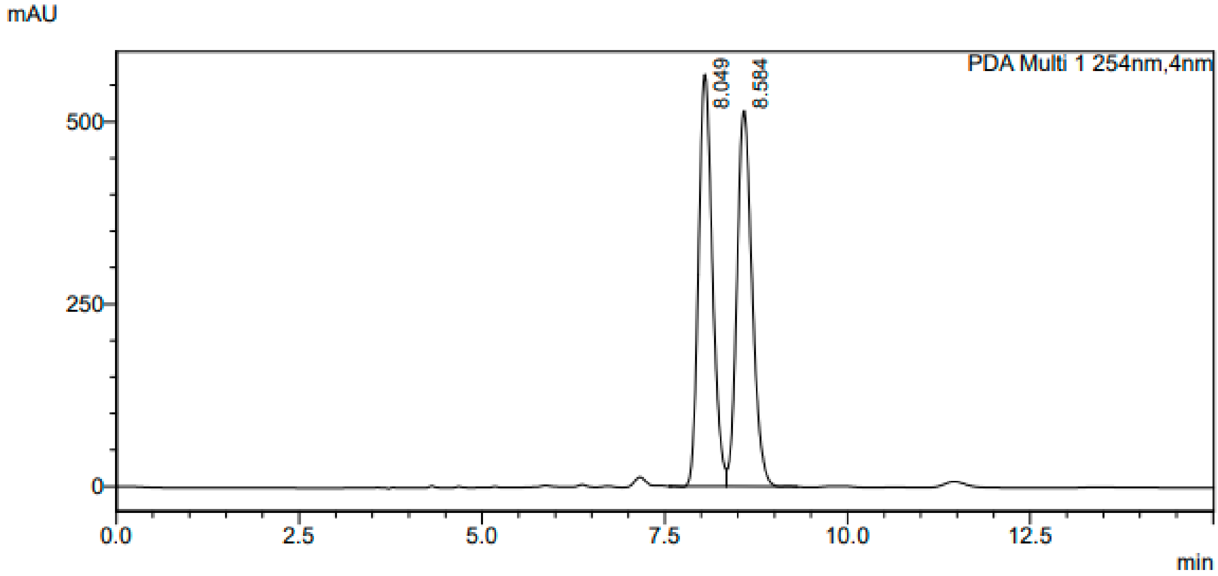

<Peak Table>

PDA Ch1 254nm

Peak#	Ret. Time	Area	Height	Conc.	Unit	Mark	Name
1	8.049	7449958	564386	0.000		M	
2	8.584	7428103	515039	0.000		V M	
Total		14878061	1079424				

Supplementary Figure 114. HPLC spectrum of racemic-**7cf**

<Chromatogram>

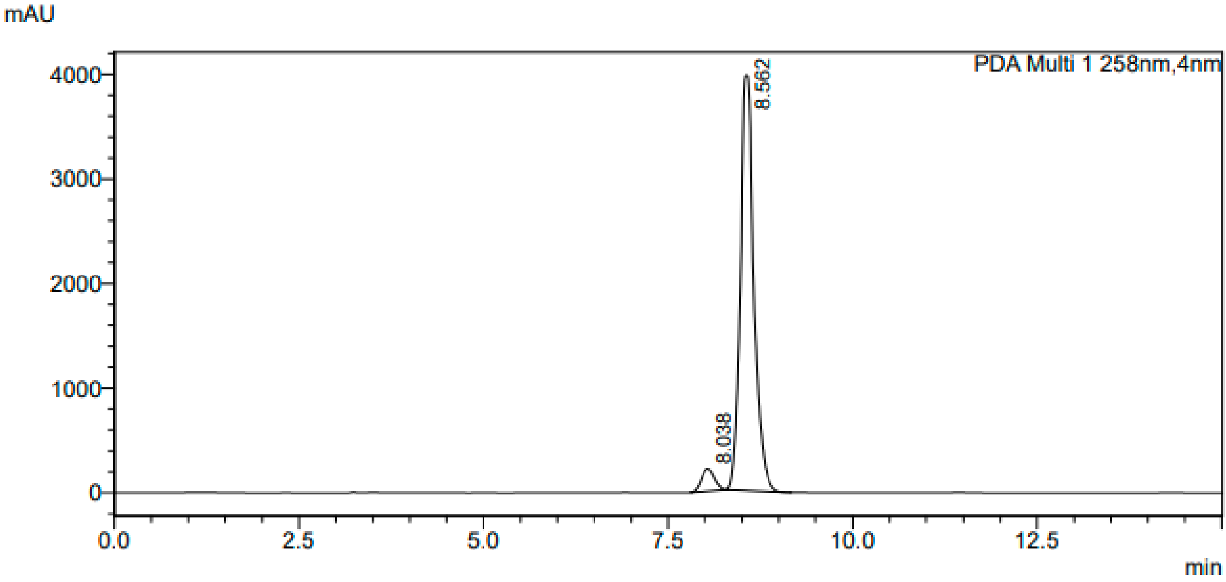

<Peak Table>

PDA Ch1 258nm

Peak#	Ret. Time	Area	Height	Conc.	Unit	Mark	Name
1	8.038	2570673	210641	0.000		M	
2	8.562	51370983	3964163	0.000		M	
Total		53941656	4174804				

Supplementary Figure 115. HPLC spectrum of (*S*)-**7cf**

(*S*,*Z*)-3-(1-(4-chlorophenyl)-3-phenylpropylidene)-4-((*E*)-styryl)-1-tosylpyrrolidine (7da)

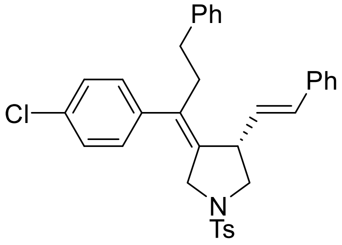

Chemical Formula: $C_{34}H_{32}ClNO_2S$
Exact Mass: 553.1842

7da was prepared according to general procedure **2.2** using (*R*,*E*)-**4d** (0.1 mmol, 50.8 mg) and (2-bromoethyl)benzene **5a** (0.2 mmol, 37.0 mg). Purification by silica gel column chromatography (PE/EA = 10/1) gave **7da** as a colorless oil (45.4 mg, 82% yield).

^{1}H NMR (600 MHz, $CDCl_3$): δ 7.67–7.60 (m, 2H), 7.37–7.34 (m, 2H), 7.33 (d, J = 7.6 Hz, 1H), 7.31 (d, J = 2.4 Hz, 2H), 7.29 (d, J = 1.8 Hz, 2H), 7.28 (d, J = 1.4 Hz, 1H), 7.25 (d, J = 1.7 Hz, 1H), 7.15–7.09 (m, 3H), 7.07–7.02 (m, 2H), 6.93–6.87 (m, 2H), 6.40 (d, J = 15.8 Hz, 1H), 6.01 (dd, J = 15.8, 8.4 Hz, 1H), 3.81 (dd, J = 14.5, 1.7 Hz, 1H), 3.58–3.50 (m, 2H), 3.36 (dd, J = 10.0, 7.0 Hz, 1H), 3.29 (dd, J = 10.0, 3.3 Hz, 1H), 2.72–2.61 (m, 1H), 2.60–2.51 (m, 1H), 2.45–2.37 (m, 5H);

^{13}C NMR (151 MHz, $CDCl_3$): δ 143.7, 141.2, 139.4, 135.8, 134.5, 133.1, 132.5, 130.8, 129.7, 129.1, 129.0, 128.9, 128.6, 128.3, 128.2, 127.8, 127.7, 126.3, 125.9, 53.9, 50.9, 44.8, 36.5, 34.0, 21.5;

HRMS: (ESI) calcd for $C_{34}H_{33}ClNO_2S^+$ ($[M+H]^+$): 554.1915; found: 554.1917;

HPLC conditions: AD-H column (20% ^{i}PrOH in hexane, 1.0 mL/min, λ = 254 nm, 30 °C), t_R (minor) = 8.4 min, t_R (major) = 9.6 min;

Optical Rotation: $[\alpha]^{25}_{D}$ = -8.7 (c = 1.20, $CHCl_3$) for 94% ee;

Absolute stereochemistry was determined through analogy with **7aa**.

<Chromatogram>

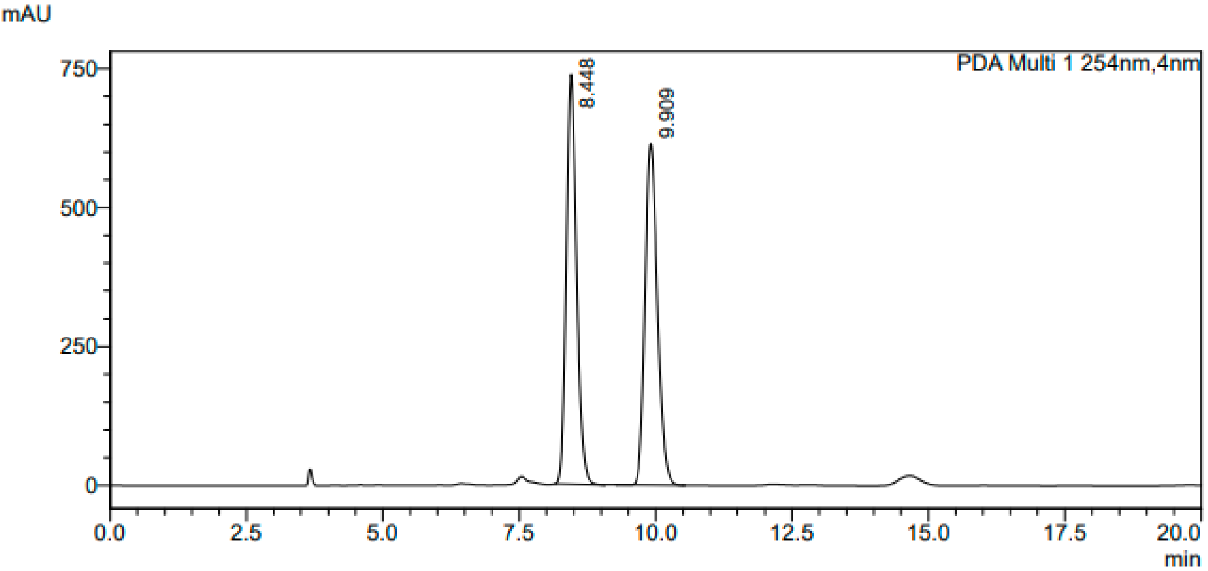

<Peak Table>

PDA Ch1 254nm

Peak#	Ret. Time	Area	Height	Conc.	Unit	Mark	Name
1	8.448	9736983	737348	0.000		M	
2	9.909	9757304	614349	0.000		M	
Total		19494287	1351697				

Supplementary Figure 116. HPLC spectrum of racemic-**7da**

<Chromatogram>

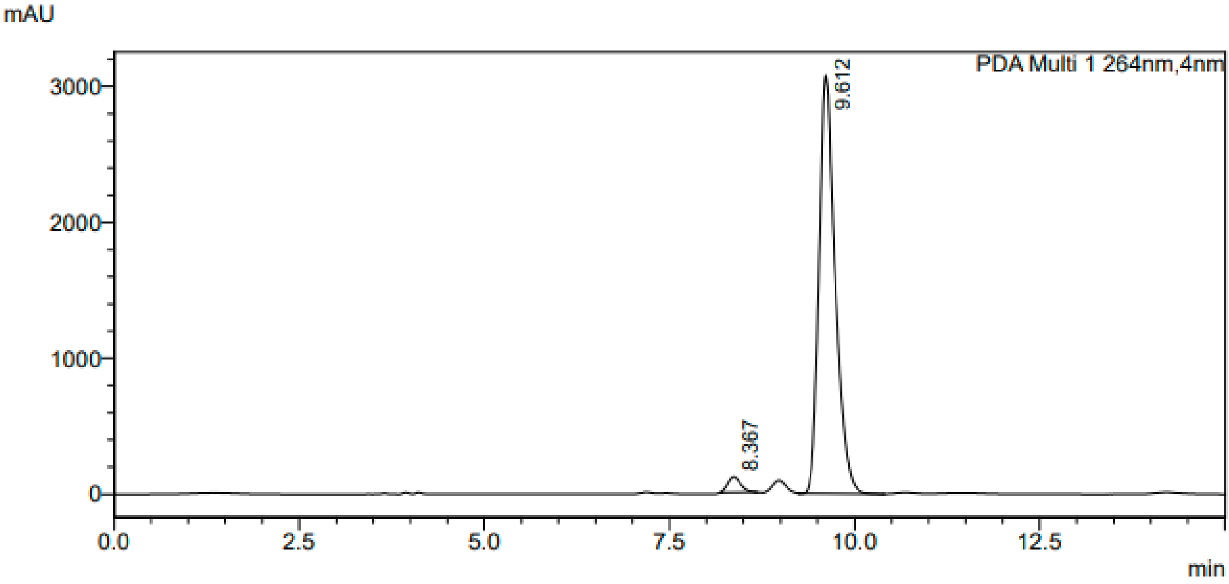

<Peak Table>

PDA Ch1 264nm

Peak#	Ret. Time	Area	Height	Conc.	Unit	Mark	Name
1	8.367	1371560	112793	0.000		M	
2	9.612	46536959	3077080	0.000		M	
Total		47908519	3189872				

Supplementary Figure 117. HPLC spectrum of (*S*)-**7da**

***tert*-butyl ((*E*)-6-phenyl-4-((*S*)-4-((*E*)-styryl)-1-tosylpyrrolidin-3-ylidene)hexyl) carbamate (7el)**

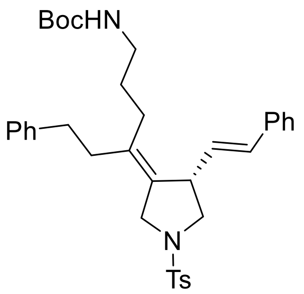

Chemical Formula: $C_{36}H_{44}N_2O_4S$
Exact Mass: 600.3022

7el was prepared according to general procedure **2.2** using (*R*,*E*)-**4e** (0.1 mmol, 50.2 mg) and *tert*-butyl (3-bromopropyl)carbamate **5l** (0.2 mmol, 50.8 mg). Purification by silica gel column chromatography (PE/EA = 3/1) gave **7el** as a colorless oil (40.3 mg, 67% yield).

^{1}H NMR (600 MHz, $CDCl_3$): δ 7.69–7.63 (m, 2H), 7.34–7.28 (m, 4H), 7.27–7.21 (m, 5H), 7.19–7.15 (m, 1H), 7.14–7.09 (m, 2H), 6.27 (d, *J* = 15.8 Hz, 1H), 5.88 (dd, *J* = 15.8, 8.3 Hz, 1H), 4.40 (s, 1H), 3.67 (d, *J* = 13.7 Hz, 1H), 3.58 (d, *J* = 13.8 Hz, 1H), 3.45 (t, *J* = 7.6 Hz, 1H), 3.31 (dd, *J* = 9.8, 2.3 Hz, 1H), 3.23 (dd, *J* = 9.8, 6.7 Hz, 1H), 3.01 (q, *J* = 6.8 Hz, 2H), 2.69–2.55 (m, 2H), 2.41 (s, 3H), 2.21 (t, *J* = 7.8 Hz, 2H), 2.14–2.04 (m, 1H), 2.02–1.94 (m, 1H), 1.55–1.45 (m, 2H), 1.42 (s, 9H);

^{13}C NMR (151 MHz, $CDCl_3$): δ 155.8, 143.6, 141.2, 136.7, 133.9, 132.5, 132.3, 129.8, 129.7, 129.6, 128.5, 128.4, 128.3, 127.8, 127.4, 126.1, 126.1, 79.2, 53.9, 49.8, 44.4, 40.5, 34.6, 33.8, 28.8, 28.4, 28.3, 21.5;

HRMS: (ESI) calcd for $C_{36}H_{45}N_2O_4S^+$ ($[M+H]^+$): 601.3095; found: 601.3099;

HPLC conditions: AD-H column (15% ^{i}PrOH in hexane, 1.0 mL/min, λ = 254 nm, 30 °C), t_R (minor) = 14.4 min, t_R (major) = 15.4 min;

Optical Rotation: $[\alpha]^{25}_D$ = -4.9 (*c* = 1.20, $CHCl_3$) for 88% ee;

Absolute stereochemistry was determined through analogy with **7aa**.

<Chromatogram>

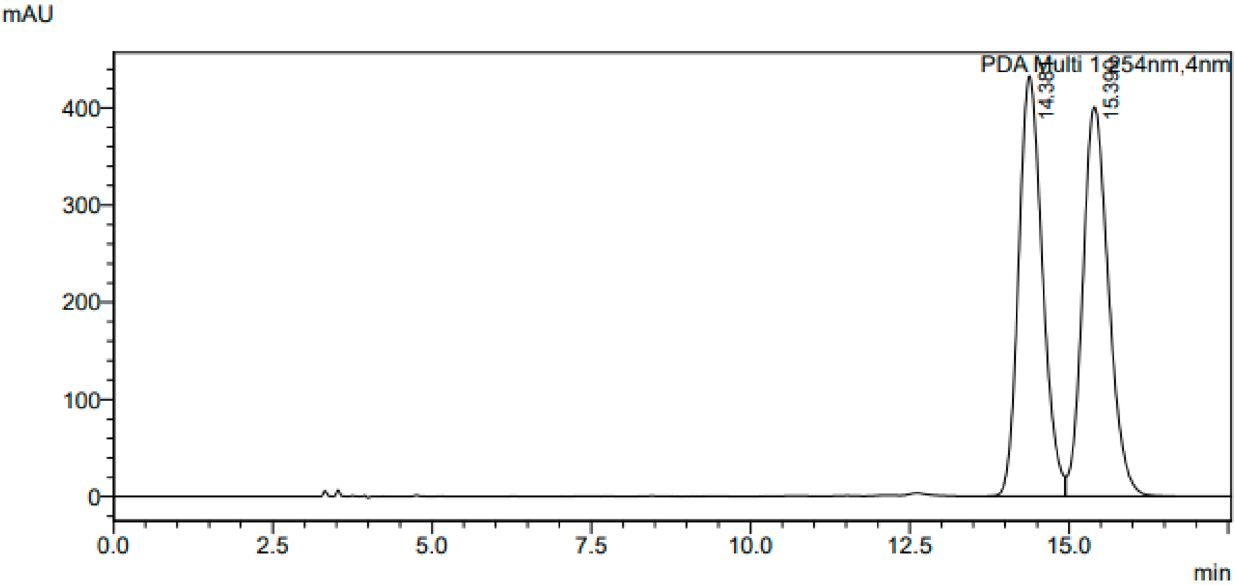

<Peak Table>

PDA Ch1 254nm

Peak#	Ret. Time	Area	Height	Conc.	Unit	Mark	Name
1	14.381	11242464	432279	0.000			
2	15.399	11315616	400501	0.000		V	
Total		22558079	832781				

Supplementary Figure 118. HPLC spectrum of racemic-**7el**

<Chromatogram>

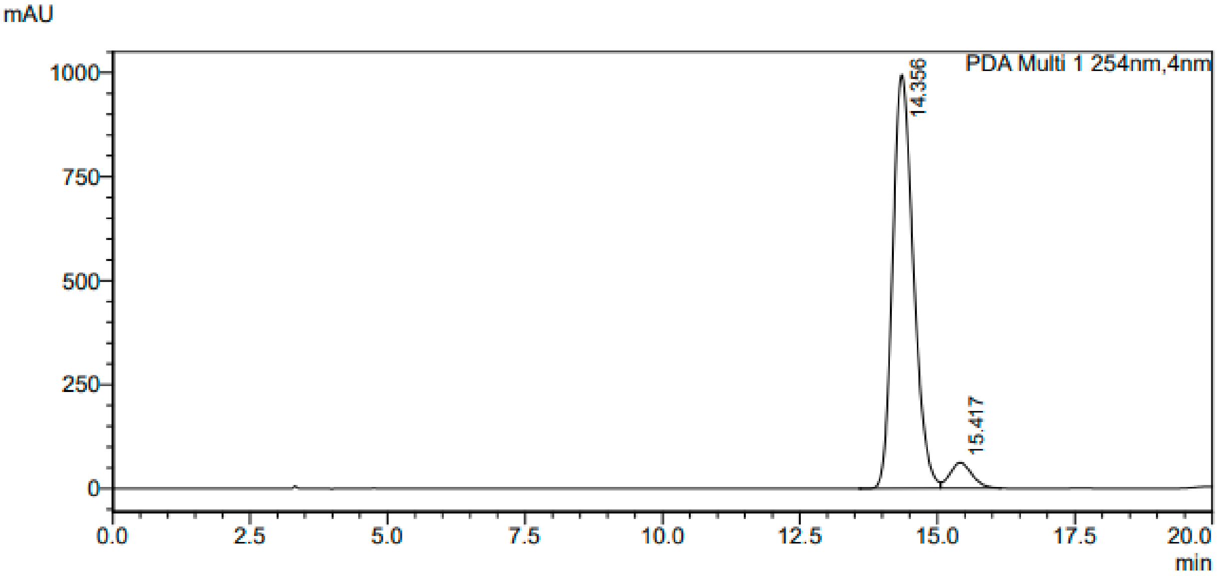

<Peak Table>

PDA Ch1 254nm

Peak#	Ret. Time	Area	Height	Conc.	Unit	Mark	Name
1	14.356	26120979	994500	0.000		M	
2	15.417	1719222	61135	0.000		V M	
Total		27840201	1055634				

Supplementary Figure 119. HPLC spectrum of (*S*)-**7el**

(*S*,*E*)-3-(4-phenylbutan-2-ylidene)-4-((*E*)-styryl)-1-tosylpyrrolidine (7fa)

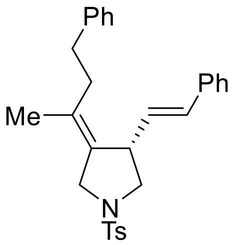

Chemical Formula: $C_{29}H_{31}NO_2S$
Exact Mass: 457.2075

7fa was prepared according to general procedure **2.2** using (*R*,*E*)-**4f** (0.1 mmol, 41.2 mg) and (2-bromoethyl)benzene **5a** (0.2 mmol, 37.0 mg). Purification by silica gel column chromatography (PE/EA = 10/1) gave **7fa** as a colorless oil (29.7 mg, 65% yield).

^{1}H NMR (600 MHz, $CDCl_3$): δ 7.74–7.69 (m, 2H), 7.33 (d, *J* = 8.0 Hz, 2H), 7.31–7.27 (m, 2H), 7.26 (d, *J* = 1.6 Hz, 1H), 7.25–7.23 (m, 1H), 7.23–7.19 (m, 1H), 7.17–7.09 (m, 3H), 7.02–6.97 (m, 2H), 6.27 (d, *J* = 15.8 Hz, 1H), 5.96 (dd, *J* = 15.8, 8.2 Hz, 1H), 3.97–3.91 (m, 1H), 3.73–3.65 (m, 1H), 3.30 (dd, *J* = 9.5, 2.4 Hz, 1H), 3.26 (t, *J* = 7.7 Hz, 1H), 3.09 (dd, *J* = 9.5, 6.6 Hz, 1H), 2.62–2.53 (m, 2H), 2.42 (s, 3H), 2.32–2.22 (m, 2H), 1.68–1.64 (m, 3H);

^{13}C NMR (151 MHz, $CDCl_3$): δ 143.6, 141.7, 136.8, 132.5, 131.2, 130.1, 129.9, 129.8, 129.6, 128.5, 128.3, 128.2, 127.9, 127.4, 126.2, 125.8, 54.2, 50.4, 44.4, 36.8, 33.9, 21.5, 18.9;

HRMS: (ESI) calcd for $C_{29}H_{32}NO_2S^+$ ($[M+H]^+$): 458.2148; found: 458.2142;

HPLC conditions: AD-H column (20% ^{i}PrOH in hexane, 1.0 mL/min, λ = 254 nm, 30 °C), t_R (major) = 8.4 min, t_R (minor) = 9.0 min;

Optical Rotation: $[\alpha]^{25}_D$ = -108.9 (*c* = 1.00, $CHCl_3$) for 93% ee;

Absolute stereochemistry was determined through analogy with **7aa**.

<Chromatogram>

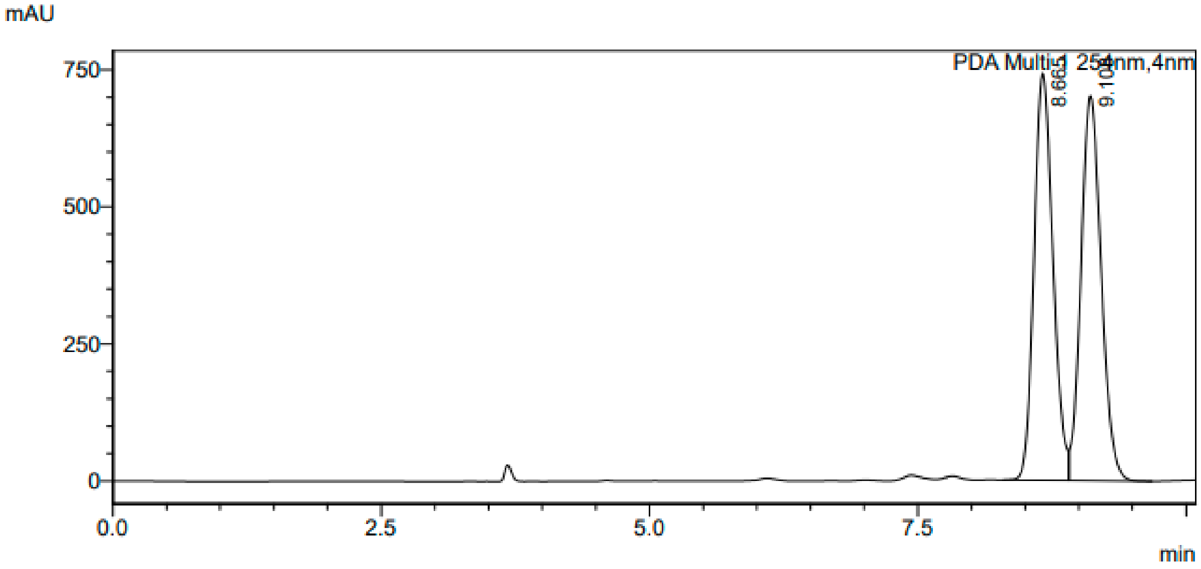

<Peak Table>

PDA Ch1 254nm

Peak#	Ret. Time	Area	Height	Conc.	Unit	Mark	Name
1	8.665	9099669	741983	0.000		M	
2	9.108	9180972	701696	0.000		V M	
Total		18280640	1443678				

Supplementary Figure 120. HPLC spectrum of racemic-**7fa**

<Chromatogram>

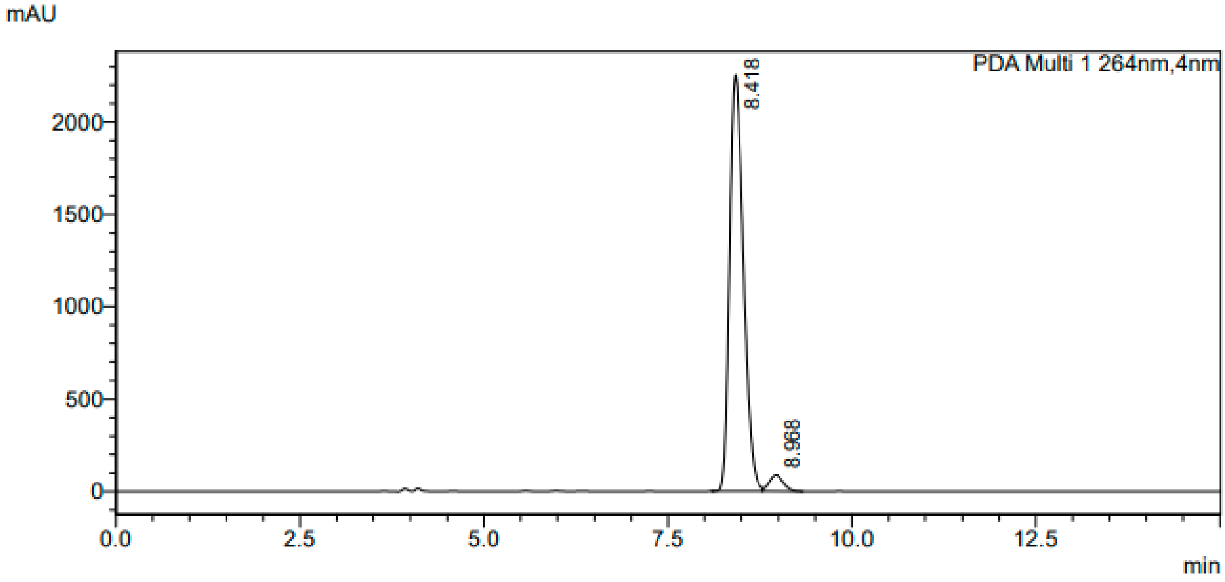

<Peak Table>

PDA Ch1 264nm

Peak#	Ret. Time	Area	Height	Conc.	Unit	Mark	Name
1	8.418	29690960	2252281	0.000		M	
2	8.968	1152960	87553	0.000		V M	
Total		30843921	2339834				

Supplementary Figure 121. HPLC spectrum of (*S*)-**7fa**

(*Z*)-5-(benzyloxy)-4-((*S*)-4-((*E*)-styryl)-1-tosylpyrrolidin-3-ylidene)pentyl acetate (7gf)

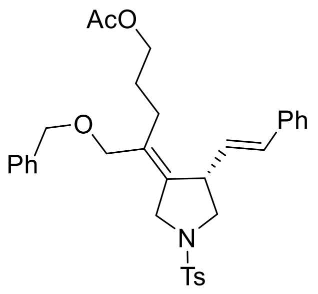

Chemical Formula: $C_{33}H_{37}NO_5S$
Exact Mass: 559.2392

7gf was prepared according to general procedure **2.2** using (*R*,*E*)-**4g** (0.1 mmol, 51.8 mg) and 3-bromopropyl acetate **5f** (0.2 mmol, 36.2 mg). Purification by silica gel column chromatography (PE/EA = 10/1) gave **7gf** as a colorless oil (31.1 mg, 55% yield).

^{1}H NMR (600 MHz, $CDCl_3$): δ 7.72–7.65 (m, 2H), 7.40–7.35 (m, 2H), 7.34–7.27 (m, 7H), 7.26–7.20 (m, 3H), 6.35 (d, *J* = 15.8 Hz, 1H), 5.98 (dd, *J* = 15.8, 8.4 Hz, 1H), 4.45 (s, 2H), 4.03–3.84 (m, 5H), 3.76 (d, *J* = 14.2 Hz, 1H), 3.54 (t, *J* = 7.0 Hz, 1H), 3.34 (dd, *J* = 9.7, 2.7 Hz, 1H), 3.26 (dd, *J* = 9.7, 6.7 Hz, 1H), 2.40 (s, 3H), 2.24–2.17 (m, 1H), 2.17–2.10 (m, 1H), 1.93 (s, 3H), 1.68–1.62 (m, 2H);

^{13}C NMR (151 MHz, $CDCl_3$): δ 171.0, 143.8, 137.8, 136.5, 135.8, 132.1, 131.3, 130.6, 129.7, 129.0, 128.5, 128.4, 127.9, 127.90, 127.84, 127.5, 126.2, 72.6, 69.4, 64.2, 53.8, 50.0, 44.7, 27.4, 26.8, 21.5, 20.8;

HRMS: (ESI) calcd for $C_{33}H_{38}NO_5S^+$ ($[M+H]^+$): 560.2465; found: 560.2461;

HPLC conditions: AD-H column (30% ^{i}PrOH in hexane, 1.0 mL/min, λ = 254 nm, 30 °C), t_R (major) = 8.7 min, t_R (minor) = 9.6 min;

Optical Rotation: $[\alpha]^{25}_D$ = -53.3 (*c* = 1.00, $CHCl_3$) for 96% ee;

Absolute stereochemistry was determined through analogy with **7aa**.

<Chromatogram>

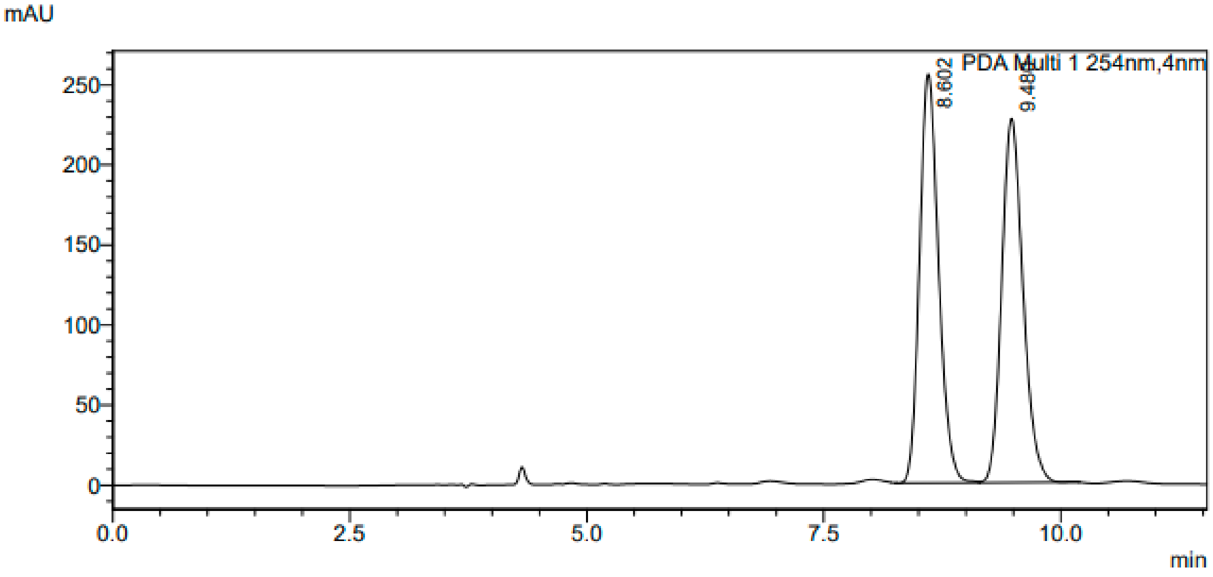

<Peak Table>

PDA Ch1 254nm

Peak#	Ret. Time	Area	Height	Conc.	Unit	Mark	Name
1	8.602	3573846	255231	0.000		M	
2	9.480	3553063	227368	0.000		V M	
Total		7126909	482599				

Supplementary Figure 122. HPLC spectrum of racemic-**7gf**

<Chromatogram>

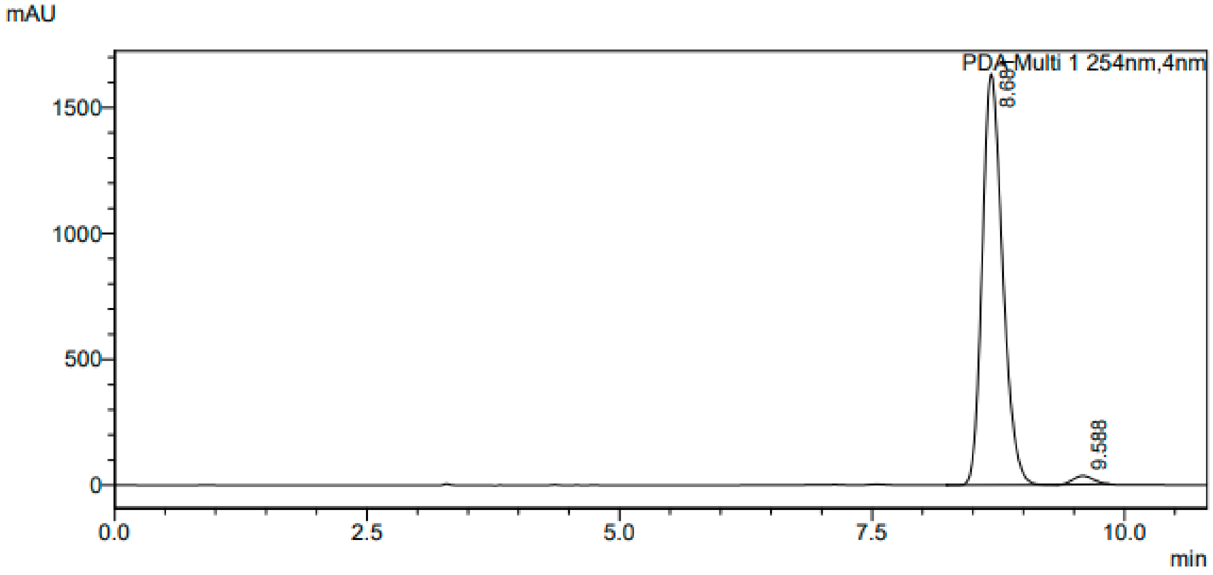

<Peak Table>

PDA Ch1 254nm

Peak#	Ret. Time	Area	Height	Conc.	Unit	Mark	Name
1	8.681	22971635	1633013	0.000		M	
2	9.588	486240	34639	0.000		M	
Total		23457875	1667652				

Supplementary Figure 123. HPLC spectrum of (*S*)-**7gf**

(*S,E*)-3-(3-phenylpropylidene)-4-((*E*)-styryl)-1-tosylpyrrolidine (7ha)

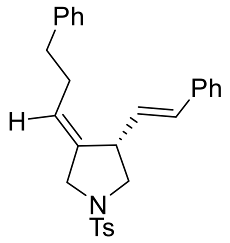

Chemical Formula: $C_{28}H_{29}NO_2S$
Exact Mass: 443.1919

7ha was prepared according to general procedure 2.2 using **(*R,E*)-4h** (0.1 mmol, 39.7 mg) and (2-bromoethyl)benzene **5a** (0.2 mmol, 37.0 mg). Purification by silica gel column chromatography (PE/EA = 10/1) gave **7ha** as a colorless oil (22.6 mg, 51% yield); ^{1}H NMR (600 MHz, $CDCl_3$) δ 7.73-7.67 (m, 2H), 7.33-7.27 (m, 4H), 7.24-7.21 (m, 3H), 7.21-7.17 (m, 2H), 7.16-7.12 (m, 1H), 7.07-7.00 (m, 2H), 6.28 (d, *J* = 15.8 Hz, 1H), 5.87 (dd, *J* = 15.8, 7.9 Hz, 1H), 5.43 (ddd, *J* = 9.3, 6.4, 1.9 Hz, 1H), 3.93-3.88 (m, 1H), 3.73 (dd, *J* = 13.4, 1.7 Hz, 1H), 3.38 (d, *J* = 8.6 Hz, 1H), 3.33-3.28 (m, 1H), 3.26 (dd, *J* = 9.9, 3.3 Hz, 1H), 2.63-2.52 (m, 2H), 2.40 (s, 3H), 2.37-2.25 (m, 2H);

^{13}C NMR (151 MHz, $CDCl_3$) δ 143.7, 141.4, 136.9, 136.7, 132.6, 130.5, 129.6, 128.8, 128.5, 128.4, 128.3, 127.9, 127.5, 126.2, 125.9, 124.2, 54.2, 52.1, 43.6, 35.4, 30.6, 21.5.

HRMS: (ESI) calcd for $C_{28}H_{30}NO_2S^+$ ($[M+H]^+$): 444.1992; found: 444.1990;

HPLC conditions: OD-H column (15% ^{i}PrOH in hexane, 1.0 mL/min, λ = 254 nm, 30 °C), t_R (major) = 13.8 min, t_R (minor) = 16.1 min;

Optical Rotation: $[\alpha]^{25}_D$ = -42.6 (*c* = 0.33, $CHCl_3$) for 89% ee;

Absolute stereochemistry was determined through analogy with **7aa**.

<Chromatogram>

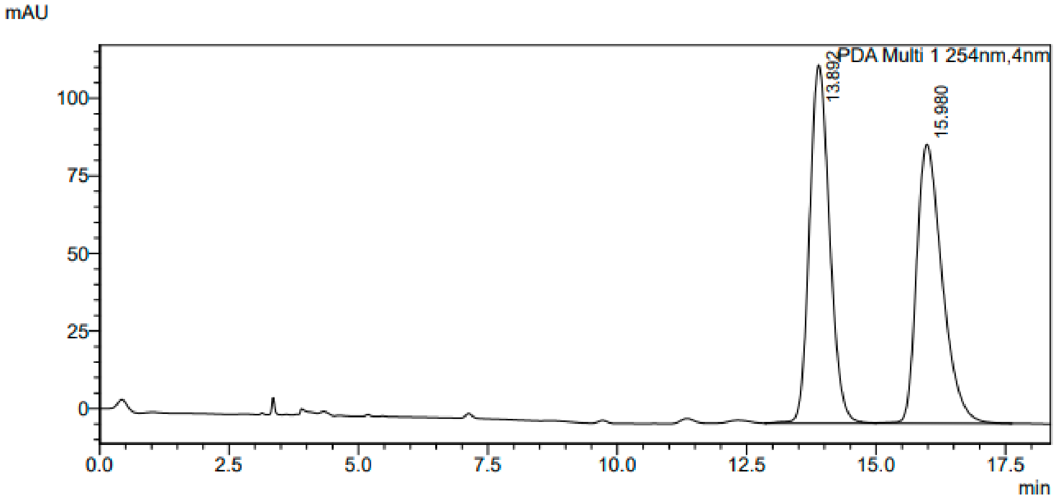

<Peak Table>

PDA Ch1 254nm

Peak#	Ret. Time	Area	Height	Conc.	Unit	Mark	Name
1	13.892	3121829	115392	0.000			
2	15.980	3094333	89885	0.000		V	
Total		6216162	205277				

Supplementary Figure 124. HPLC spectrum of racemic-**7ha**

<Chromatogram>

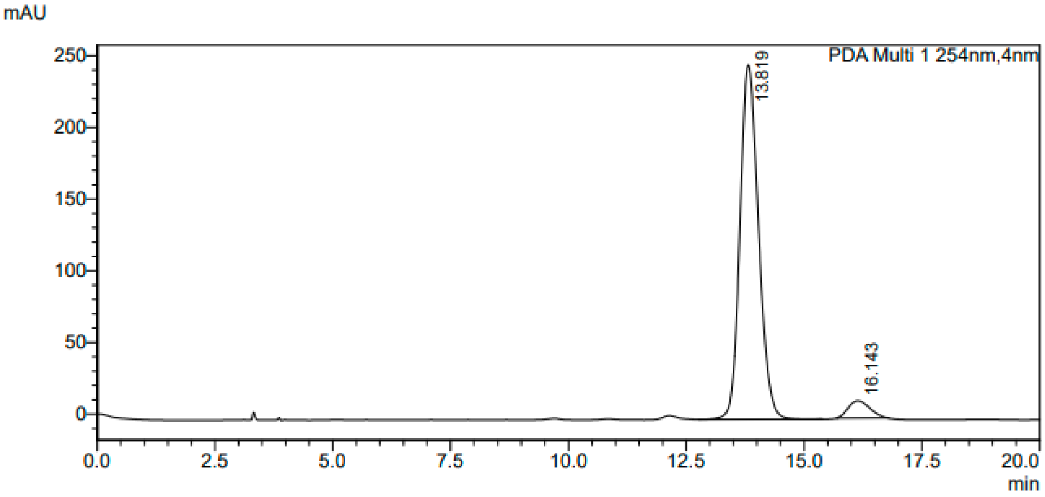

<Peak Table>

PDA Ch1 254nm

Peak#	Ret. Time	Area	Height	Conc.	Unit	Mark	Name
1	13.819	6783080	247157	0.000		M	
2	16.143	387009	12153	0.000		M	
Total		7170088	259310				

Supplementary Figure 125. HPLC spectrum of (*S*)-**7ha**

(*S*,*Z*)-3-(1,3-diphenylpropylidene)-2,2-dimethyl-4-((*E*)-styryl)-1-tosylpyrrolidine (7ia)

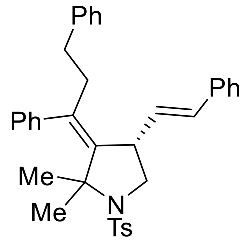

Chemical Formula: $C_{36}H_{37}NO_2S$
Exact Mass: 547.2545

7ia was prepared according to general procedure **2.2** using (*R*,*E*)-**4i** (0.1 mmol, 50.2mg) and (2-bromoethyl)benzene **5a** (0.2 mmol, 37.0 mg). Purification by silica gel column chromatography (PE/EA = 10/1) gave **7ia** as a colorless oil (37.8 mg, 69% yield).

^{1}H NMR (600 MHz, $CDCl_3$): δ 7.72–7.68 (m, 2H), 7.37–7.28 (m, 5H), 7.27–7.25 (m, 1H), 7.24–7.12 (m, 9H), 7.04–7.01 (m, 2H), 6.38 (d, J = 15.8 Hz, 1H), 6.10–6.04 (m, 1H), 3.48–3.43 (m, 2H), 3.43–3.39 (m, 1H), 2.60–2.47 (m, 4H), 2.37 (s, 3H), 1.53 (s, 3H), 1.32 (s, 3H);

^{13}C NMR (151 MHz, $CDCl_3$): δ 143.8, 142.7, 141.6, 140.3, 138.6, 136.8, 136.1, 130.2, 130.2, 129.7, 129.3, 128.5, 128.3, 128.3, 127.8, 127.4, 127.2, 126.9, 126.2, 125.9, 68.3, 51.6, 44.2, 40.2, 33.5, 28.3, 27.2, 21.4;

HRMS: (ESI) calcd for $C_{36}H_{38}NO_2S^+$ ($[M+H]^+$): 548.2618; found: 548.2617;

HPLC conditions: AD-H column (15% ^{i}PrOH in hexane, 1.0 mL/min, λ = 254 nm, 30 °C), t_R (major) = 8.3 min, t_R (minor) = 9.1 min;

Optical Rotation: $[\alpha]^{25}_D$ = -12.2 (c = 1.20, $CHCl_3$) for 88% ee;

Absolute stereochemistry was determined through analogy with **7aa**.

<Chromatogram>

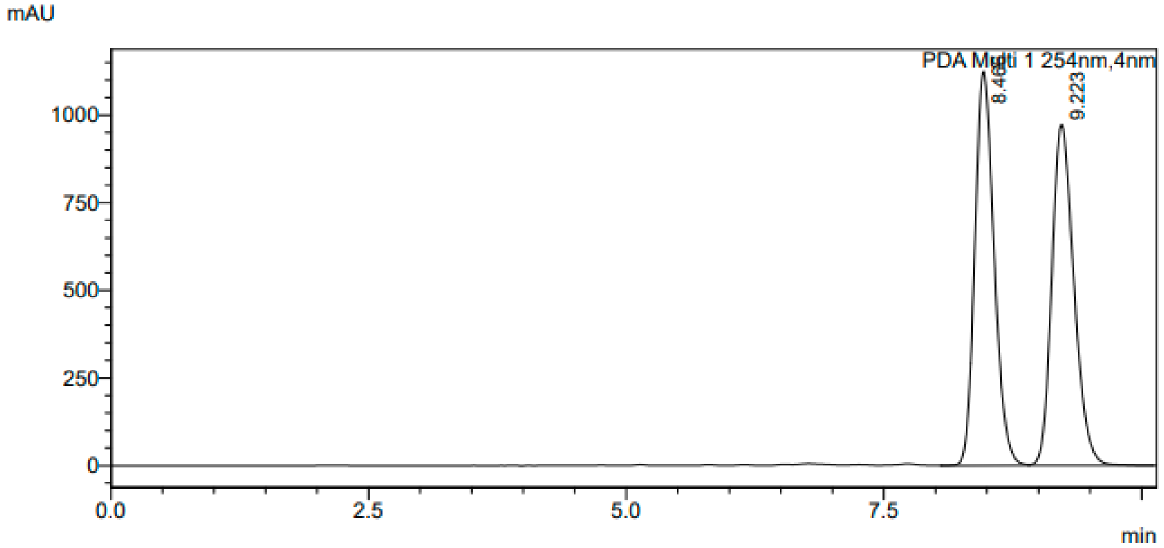

<Peak Table>

PDA Ch1 254nm

Peak#	Ret. Time	Area	Height	Conc.	Unit	Mark	Name
1	8.466	14658682	1124960	0.000			
2	9.223	14545287	972108	0.000		V	
Total		29203970	2097068				

Supplementary Figure 126. HPLC spectrum of racemic-**7ia**

<Chromatogram>

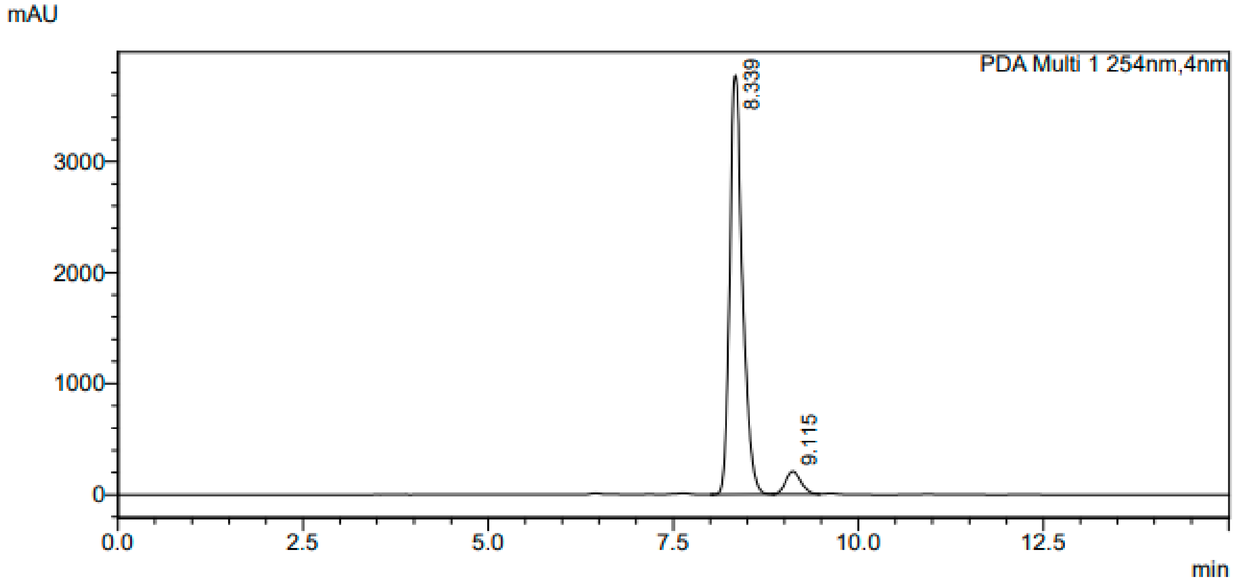

<Peak Table>

PDA Ch1 254nm

Peak#	Ret. Time	Area	Height	Conc.	Unit	Mark	Name
1	8.339	45496280	3772407	0.000		M	
2	9.115	2932027	204669	0.000		M	
Total		48428307	3977076				

Supplementary Figure 127. HPLC spectrum of (*S*)-**7ia**

(*S*,*E*)-4-(1,3-diphenylpropylidene)-3-((*E*)-styryl)-1-tosylpiperidine (7ja)

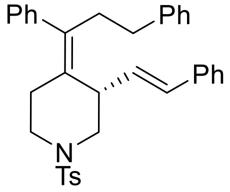

Chemical Formula: $C_{35}H_{35}NO_2S$
Exact Mass: 533.2389

7ja was prepared according to general procedure **2.2** using (*R*,*E*)-**4j** (0.1 mmol, 48.8 mg) and (2-bromoethyl)benzene **5a** (0.2 mmol, 37.0 mg). Purification by silica gel column chromatography (PE/EA = 10/1) gave **7ja** as a colorless oil (26.2 mg, 49% yield).

^{1}H NMR (600 MHz, $CDCl_3$): δ 7.62-7.59 (m, 2H), 7.39–7.36 (m, 2H), 7.36–7.30 (m, 6H), 7.29–7.26 (m, 1H), 7.24–7.21 (m, 1H), 7.15–7.12 (m, 2H), 7.10–7.06 (m, 5H), 6.49 (dd, *J* = 16.1, 1.6 Hz, 1H), 6.33 (dd, *J* = 16.1, 5.8 Hz, 1H), 3.77 (dt, *J* = 11.4, 2.3 Hz, 1H), 3.69 (ddt, *J* = 10.0, 4.7, 2.4 Hz, 1H), 3.43 (dq, *J* = 5.8, 2.2 Hz, 1H), 2.81–2.74 (m, 1H), 2.68–2.60 (m, 2H), 2.47 (s, 3H), 2.44–2.39 (m, 1H), 2.29–2.22 (m, 1H), 2.20–2.15 (m, 1H), 2.10–2.03 (m, 1H), 1.97 (dd, *J* = 11.5, 3.5 Hz, 1H);

^{13}C NMR (151 MHz, $CDCl_3$): δ 143.4, 141.9, 141.5, 137.2, 136.2, 133.2, 131.9, 131.0, 129.9, 129.6, 128.9, 128.6, 128.5, 128.3, 128.2, 127.7, 127.3, 126.7, 126.3, 125.9, 51.0, 47.7, 40.2, 35.7, 34.1, 27.1, 21.6;

HRMS: (ESI) calcd for $C_{35}H_{36}NO_2S^+$ ($[M+H]^+$): 534.2461; found: 534.2463;

HPLC conditions: AD-H column (15% ^{i}PrOH in hexane, 1.0 mL/min, λ = 254 nm, 30 °C), t_R (major) = 13.3 min, t_R (minor) = 18.0 min;

Optical Rotation: $[\alpha]^{25}_D$ = -23.7 (*c* = 0.67, $CHCl_3$) for 44% ee;

Absolute stereochemistry was determined through analogy with **7aa.**

<Chromatogram>

mAU

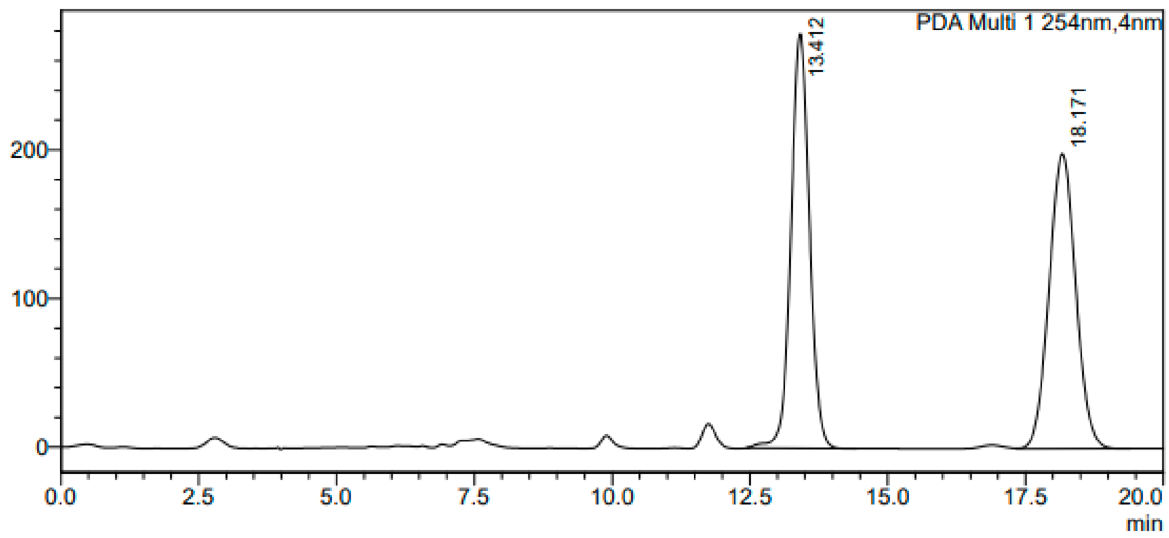

<Peak Table>

PDA Ch1 254nm

Peak#	Ret. Time	Area	Height	Conc.	Unit	Mark	Name
1	13.412	6542507	278803	0.000		M	
2	18.171	6683827	198333	0.000			
Total		13226334	477136				

Supplementary Figure 128. HPLC spectrum of racemic-**7ja**

<Chromatogram>

mAU

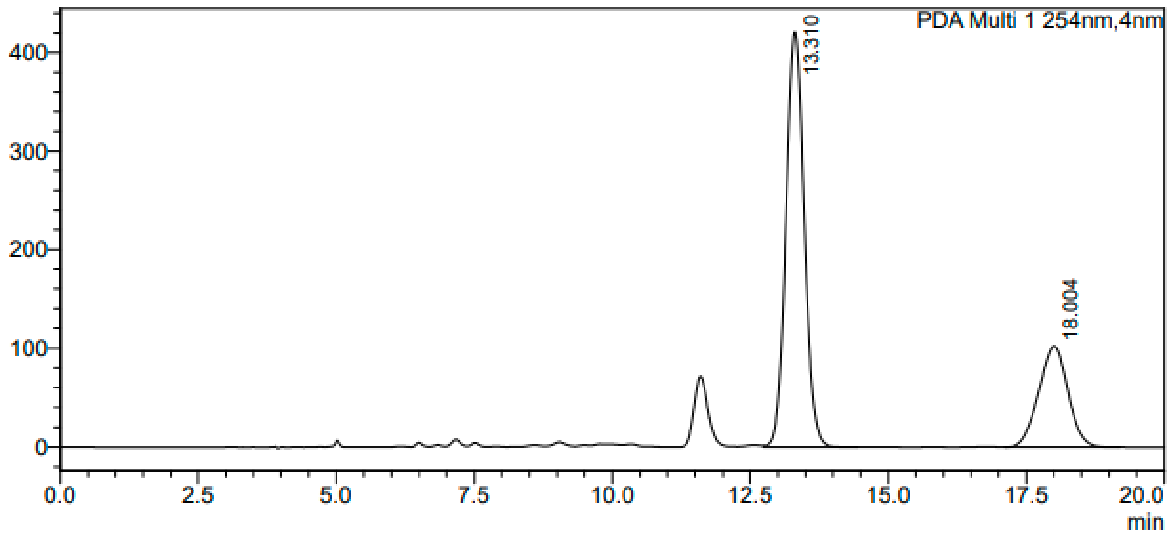

<Peak Table>

PDA Ch1 254nm

Peak#	Ret. Time	Area	Height	Conc.	Unit	Mark	Name
1	13.310	9654685	421091	0.000			
2	18.004	3764244	102415	0.000			
Total		13418929	523506				

Supplementary Figure 129. HPLC spectrum of (*S*)-**7ja**

(*R*,*Z*)-3-(1,3-diphenylpropylidene)-4-((*E*)-prop-1-en-1-yl)-1-tosylpyrrolidine (7ka)

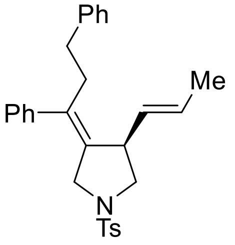

Chemical Formula: $C_{29}H_{31}NO_2S$
Exact Mass: 457.2075

7ka was prepared according to general procedure **2.2** using (*R*,*E*)-**4k** (0.1 mmol, 41.2 mg) and (2-bromoethyl)benzene **5a** (0.2 mmol, 37.0 mg). Purification by silica gel column chromatography (PE/EA = 10/1) gave **7ka** as a colorless oil (32.0 mg, 70% yield).

^{1}H NMR (600 MHz, $CDCl_3$): δ 7.66–7.56 (m, 2H), 7.39–7.33 (m, 2H), 7.30 (dd, J = 7.7, 2.3 Hz, 3H), 7.24–7.18 (m, 2H), 7.17–7.12 (m, 1H), 7.11–7.05 (m, 2H), 7.05–6.97 (m, 2H), 5.53–5.44 (m, 1H), 5.34–5.24 (m, 1H), 3.81–3.72 (m, 1H), 3.50 (d, J = 14.2 Hz, 1H), 3.36–3.29 (m, 1H), 3.29–3.13 (m, 2H), 2.67–2.57 (m, 1H), 2.56–2.50 (m, 1H), 2.49–2.38 (m, 5H), 1.64 (dd, J = 6.6, 1.6 Hz, 3H);

^{13}C NMR (151 MHz, $CDCl_3$): δ 141.8, 141.2, 136.1, 134.1, 132.7, 130.8, 129.5, 128.5, 128.2, 128.2, 127.8, 127.7, 127.1, 126.0, 125.8, 54.1, 50.9, 44.2, 36.4, 34.1, 21.5, 17.8;

HRMS: (ESI) calcd for $C_{29}H_{32}NO_2S^+$ ($[M+H]^+$): 458.2148; found: 458.2146;

HPLC conditions: AD-H column (10% ^{i}PrOH in hexane, 1.0 mL/min, λ = 254 nm, 30 °C), t_R (minor) = 17.6 min, t_R (major) = 18.4 min;

Optical Rotation: $[\alpha]^{25}_D$ = -7.3 (*c* = 1.00, $CHCl_3$) for 83% ee;

Absolute stereochemistry was determined through analogy with **7aa**.

<Chromatogram>

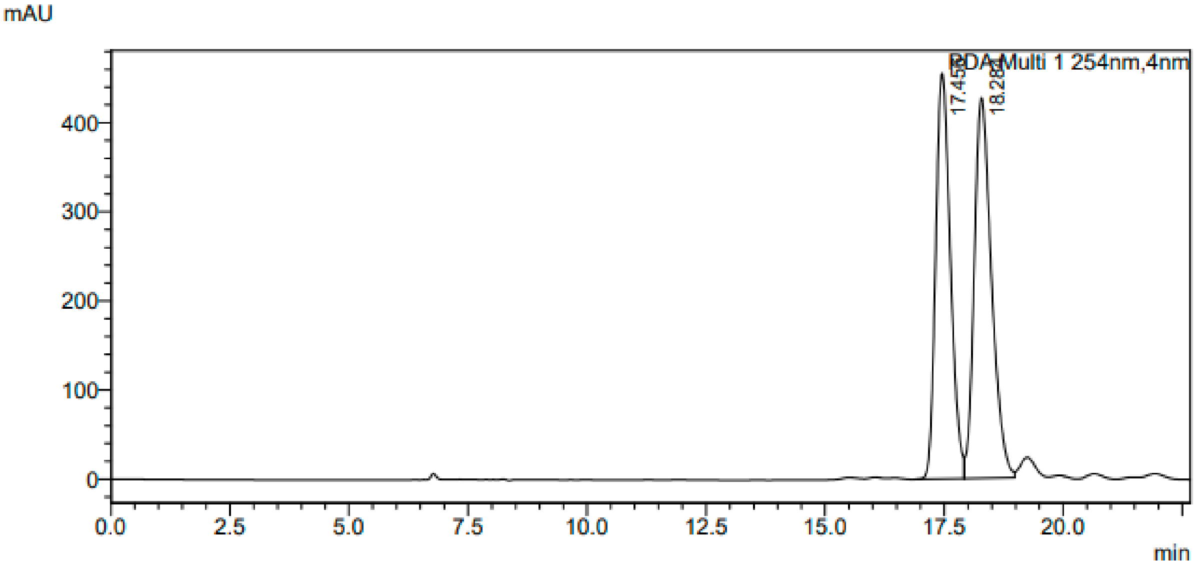

<Peak Table>

PDA Ch1 254nm

Peak#	Ret. Time	Area	Height	Conc.	Unit	Mark	Name
1	17.456	9768118	455034	0.000		M	
2	18.284	10758801	427063	0.000		V M	
Total		20526919	882097				

Supplementary Figure 130. HPLC spectrum of racemic-**7ka**

<Chromatogram>

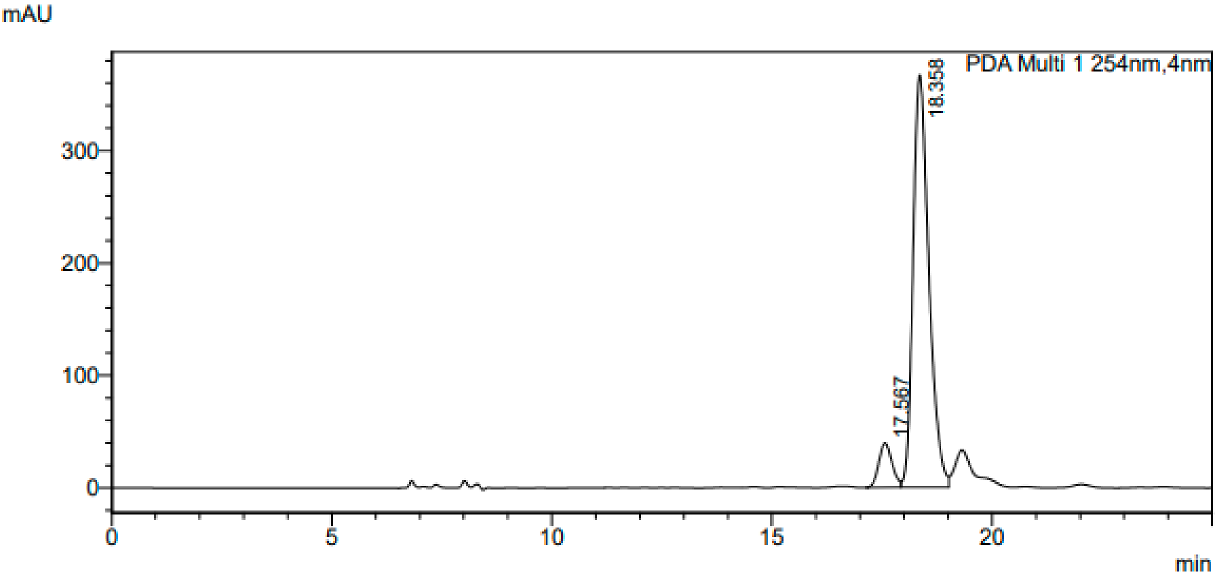

<Peak Table>

PDA Ch1 254nm

Peak#	Ret. Time	Area	Height	Conc.	Unit	Mark	Name
1	17.567	832772	39337	0.000		M	
2	18.358	9159681	366740	0.000		V M	
Total		9992452	406076				

Supplementary Figure 131. HPLC spectrum of (*R*)-**7ka**

(8*R*,9*S*,13*S*,14*S*)-13-methyl-17-oxo-7,8,9,11,12,13,14,15,16,17-decahydro-6*H*-cyclopenta[a]phenanthren-3-yl (*Z*)-5-phenyl-5-((*S*)-4-((*E*)-styryl)-1-tosylpyrrolidin-3-ylidene)pentanoate (7ap)

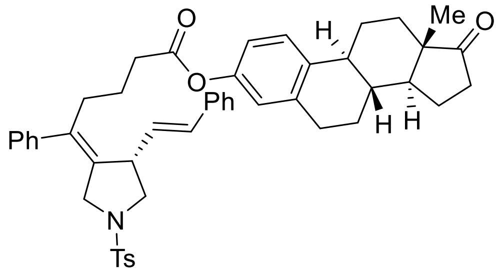

Chemical Formula: $C_{48}H_{51}NO_5S$
Exact Mass: 753.3488

7ap was prepared according to general procedure **2.2** using (*R*,*E*)-**4a** (0.1 mmol, 47.4 mg) and (8*R*,9*S*,13*S*,14*S*)-13-methyl-17-oxo-7,8,9,11,12,13,14,15,16,17-decahydro-6*H*-cyclopenta[*a*]phenanthren-3-yl 4-bromobutanoate **5p** (0.2 mmol, 83.8 mg). Purification by silica gel column chromatography (PE/EA = 4/1) gave **7ap** as a white solid (51.3 mg, 68% yield, > 20/1 d.r.).

^{1}H NMR (600 MHz, $CDCl_3$): δ 7.67–7.60 (m, 2H), 7.38–7.32 (m, 2H), 7.32–7.26 (m, 7H), 7.25–7.20 (m, 2H), 7.10–7.05 (m, 2H), 6.69 (dd, *J* = 8.5, 2.5 Hz, 1H), 6.65 (d, *J* = 2.5 Hz, 1H), 6.44 (d, *J* = 15.8 Hz, 1H), 6.04 (dd, *J* = 15.8, 8.2 Hz, 1H), 3.84 (dd, *J* = 14.3, 1.6 Hz, 1H), 3.74–3.67 (m, 1H), 3.59 (d, *J* = 14.4 Hz, 1H), 3.43 (dd, *J* = 10.0, 6.9 Hz, 1H), 3.36 (dd, *J* = 10.0, 3.0 Hz, 1H), 2.93–2.81 (m, 2H), 2.54–2.43 (m, 3H), 2.40 (s, 3H), 2.39–2.36 (m, 2H), 2.31–2.23 (m, 1H), 2.20–2.10 (m, 1H), 2.08–1.94 (m, 3H), 1.66–1.56 (m, 5H), 1.55–1.39 (m, 4H), 0.90 (s, 3H);

^{13}C NMR (151 MHz, $CDCl_3$): δ 220.8, 172.0, 148.4, 143.7, 140.9, 138.0, 137.3, 136.8, 136.7, 134.1, 132.7, 130.6, 129.7, 129.5, 128.7, 128.6, 127.9, 127.6, 127.4, 126.4, 126.3, 121.5, 118.7, 54.0, 51.0, 50.4, 48.0, 44.6, 44.1, 38.0, 35.9, 33.8, 33.6, 31.6, 29.4, 26.3, 25.8, 23.1, 21.6, 21.6, 13.8;

HRMS: (ESI) calcd for $C_{48}H_{52}N_1O_5S^+$[M+H]$^+$ 754.3561; found 754.2567.

(3a*R*,5*R*,6*S*,6a*R*)-5-((*R*)-2,2-dimethyl-1,3-dioxolan-4-yl)-2,2-dimethyltetrahydrofuro[2,3-*d*][1,3]dioxol-6-yl (*Z*)-5-phenyl-5-((*S*)-4-((*E*)-styryl)-1-tosylpyrrolidin-3-ylidene)pentanoate (7aq)

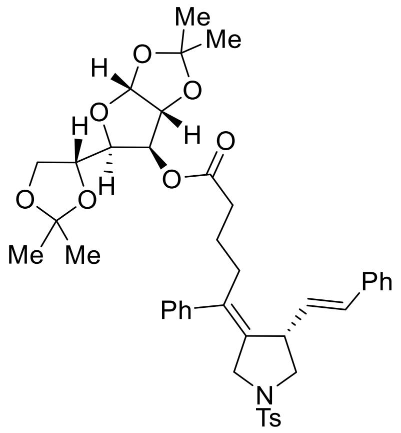

Chemical Formula: $C_{42}H_{49}NO_9S$
Exact Mass: 743.3128

7aq was prepared according to general procedure **2.2** using (*R*,*E*)-**4a** (0.1 mmol, 47.4 mg) and (3a*R*,5*R*,6*S*,6a*R*)-5-((*R*)-2,2-dimethyl-1,3-dioxolan-4-yl)-2,2-dimethyltetrahydrofuro[2,3-*d*][1,3]dioxol-6-yl 4-bromobutanoate **5q** (0.2 mmol, 81.8 mg). Purification by silica gel column chromatography (PE/EA = 4/1) gave **7ap** as a white solid (46.1 mg, 62% yield, > 20/1 d.r.).

^{1}H NMR (600 MHz, $CDCl_3$): δ 7.68–7.59 (m, 2H), 7.37–7.26 (m, 9H), 7.25–7.21 (m, 1H), 7.08–7.00 (m, 2H), 6.43 (d, *J* = 15.7 Hz, 1H), 6.02 (dd, *J* = 15.8, 8.2 Hz, 1H), 5.70 (d, *J* = 3.6 Hz, 1H), 5.13 (d, *J* = 3.0 Hz, 1H), 4.25 (d, *J* = 3.6 Hz, 1H), 4.12 (dd, *J* = 7.7, 3.1 Hz, 1H), 4.03–3.90 (m, 3H), 3.82 (dd, *J* = 14.3, 1.6 Hz, 1H), 3.70–3.64 (m, 1H), 3.56 (d, *J* = 14.3 Hz, 1H), 3.43 (dd, *J* = 10.0, 7.0 Hz, 1H), 3.33 (dd, *J* = 10.0, 3.2 Hz, 1H), 2.46–2.32 (m, 5H), 2.14 (t, *J* = 7.6 Hz, 2H), 1.55–1.44 (m, 5H), 1.34 (s, 3H), 1.27 (s, 3H), 1.21 (s, 3H);

^{13}C NMR (151 MHz, $CDCl_3$): δ 171.6, 143.7, 140.8, 136.7, 136.6, 134.0, 132.5, 130.6, 129.6, 129.4, 128.6, 128.6, 127.8, 127.6, 127.5, 127.3, 126.2, 112.2, 109.2, 104.9, 83.2, 79.7, 75.8, 72.3, 67.1, 53.9, 51.0, 44.6, 33.6, 33.4, 26.7, 26.7, 26.2, 25.2, 23.0, 21.5;

HRMS: (ESI) calcd for $C_{42}H_{50}NO_9S^+$ ([M+H]$^+$): 744.3201; found: 744.3202.

Absolute stereochemistry was determined through analogy with **7aa**.

(*S*,*Z*)-3-(1-phenyl-2-((3a*R*,5*R*,5a*S*,8a*S*,8b*R*)-2,2,7,7-tetramethyltetrahydro-5*H*-bis([1,3]dioxolo)[4,5-*b*:4',5'-*d*]pyran-5-yl)ethylidene)-4-((*E*)-styryl)-1-tosylpyrrolidine (7ar)

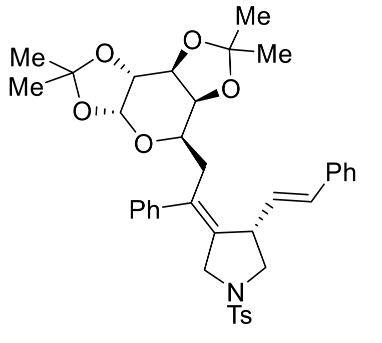

Chemical Formula: $C_{38}H_{43}NO_7S$
Exact Mass: 657.2760

7ar was prepared according to general procedure **2.2** using (*R*,*E*)-**4a** (0.1 mmol, 47.4 mg) and (3a*R*,5*S*,5a*R*,8a*S*,8b*R*)-5-(bromomethyl)-2,2,7,7-tetramethyltetrahydro-5*H*-bis([1,3] dioxolo)[4,5-*b*:4',5'-*d*]pyran **5r** (0.2 mmol, 64.6 mg). Purification by silica gel column chromatography (PE/EA = 4/1) to **7ar** as a colorless oil (38.8 mg, 59% yield, > 20/1 d.r.).
^{1}H NMR (600 MHz, $CDCl_3$): δ 7.67–7.60 (m, 2H), 7.33–7.29 (m, 2H), 7.29–7.26 (m, 3H), 7.26–7.21 (m, 4H), 7.21–7.16 (m, 1H), 7.12–7.06 (m, 2H), 6.41 (dd, *J* = 16.0, 1.2 Hz, 1H), 6.06 (dd, *J* = 15.9, 7.3 Hz, 1H), 5.33 (d, *J* = 5.1 Hz, 1H), 4.46 (dd, *J* = 7.9, 2.4 Hz, 1H), 4.14 (dd, *J* = 5.1, 2.4 Hz, 1H), 3.97 (dd, *J* = 7.9, 1.9 Hz, 1H), 3.86–3.81 (m, 1H), 3.78 (d, *J* = 14.3 Hz, 1H), 3.66 (d, *J* = 14.2 Hz, 1H), 3.51–3.43 (m, 2H), 3.32 (dd, *J* = 10.1, 3.4 Hz, 1H), 2.79 (dd, *J* = 14.2, 8.2 Hz, 1H), 2.63 (dd, *J* = 14.4, 5.5 Hz, 1H), 2.39 (s, 3H), 1.27 (s, 3H), 1.25 (s, 3H), 1.17 (s, 3H), 0.85 (s, 3H);
^{13}C NMR (151 MHz, $CDCl_3$): δ 143.6, 140.5, 137.1, 135.8, 133.0, 132.8, 130.3, 129.6, 129.4, 128.6, 128.3, 127.8, 127.8, 127.3, 127.1, 126.2, 109.0, 108.2, 96.4, 72.5, 70.9, 70.1, 65.1, 53.7, 51.0, 43.8, 34.8, 25.8, 25.2, 24.8, 24.5, 21.5;
HRMS: (ESI) calcd for $C_{39}H_{46}NO_7S^+$ ($[M+H]^+$): 658.2833; found: 658.2840.
Absolute stereochemistry was determined through analogy with **7aa**.

(*S*,*Z*)-3-((*R*)-3-(6-methoxynaphthalen-2-yl)-1-phenylbutylidene)-4-((*E*)-styryl)-1-tosylpyrrolidine (7as)

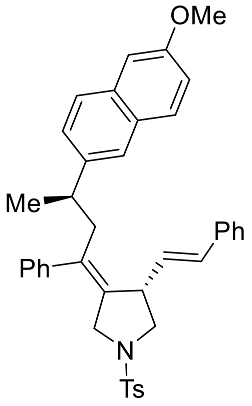

Chemical Formula: $C_{40}H_{39}NO_3S$
Exact Mass: 613.2651

7as was prepared according to general procedure **2.2** using (*R*,*E*)-**4a** (0.1 mmol, 47.4 mg) and (S)-2-(1-bromopropan-2-yl)-6-methoxynaphthalene **5s** (0.2 mmol, 55.8 mg). Purification by silica gel column chromatography (PE/EA = 5/1) gave **7as** as a colorless oil (35.0 mg, 57% yield, 15/1 d.r.).

^{1}H NMR (600 MHz, $CDCl_3$): δ 7.60–7.54 (m, 3H), 7.53–7.49 (m, 1H), 7.39–7.31 (m, 6H), 7.31–7.28 (m, 2H), 7.28–7.22 (m, 4H), 7.14–7.05 (m, 5H), 6.42 (d, *J* = 15.8 Hz, 1H), 6.03 (dd, *J* = 15.8, 8.3 Hz, 1H), 3.90 (s, 3H), 3.82 (d, *J* = 14.3 Hz, 1H), 3.55 (d, *J* = 14.3 Hz, 1H), 3.41–3.35 (m, 1H), 3.22 (dd, *J* = 10.0, 3.2 Hz, 1H), 3.15 (dd, *J* = 10.0, 7.0 Hz, 1H), 2.74–2.61 (m, 3H), 2.37 (s, 3H), 1.09 (d, *J* = 6.6 Hz, 3H);

^{13}C NMR (151 MHz, $CDCl_3$): δ 157.2, 143.5, 141.9, 140.8, 136.7, 136.2, 134.4, 133.1, 132.7, 130.8, 129.63, 129.56, 128.85, 128.82, 128.6, 128.5, 127.73, 127.66, 127.6, 127.2, 126.6, 126.2, 126.1, 124.8, 118.6, 105.5, 55.2, 53.8, 51.0, 44.7, 42.5, 37.5, 21.5, 20.9;

HRMS: (ESI) calcd for $C_{40}H_{40}NO_3S^+$ ($[M+H]^+$): 614.2723; found: 614.2722.

Absolute stereochemistry was determined through analogy with **7aa**.

9. Synthetic Transformations and Applications

7.1 Synthesis of (*R*)-8-phenyl-2-tosyl-1,2,3,3a,6,7-hexahydrocyclohepta[*c*]pyrrole (8)

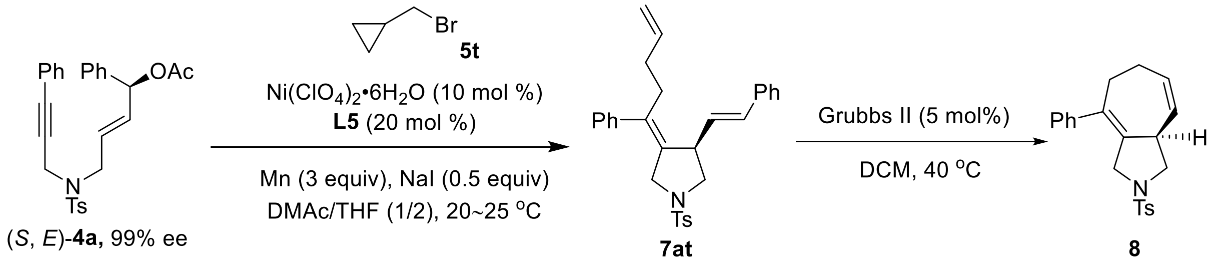

7at was prepared according to general procedure **2.2** from (*S*,*E*)-**4a** (0.1 mmol, 47.4 mg) and (bromomethyl)cyclopropane **5t** (0.2 mmol, 27.0 mg).

To a solution of the crude product **7at** in dry DCM (1 mL) was added Grubbs II catalyst (0.005 mmol, 4.2 mg). The resulting mixture was stirred under N_2 for 8 hours at 40 °C. Then the reaction mixture was concentrated under vacuum. The residue was purified by chromatography on silica gel, eluting with PE/EA = 20/1 to afford **8** as a colorless oil (16.1 mg, 44% yield in two steps).

^{1}H NMR (600 MHz, $CDCl_3$): δ 7.66–7.63 (m, 2H), 7.34–7.31 (m, 4H), 7.26–7.22 (m, 1H), 7.08 (dd, *J* = 8.1, 1.3 Hz, 2H), 5.68–5.63 (m, 1H), 5.35 (dd, *J* = 11.2, 1.9 Hz, 1H), 3.93–3.88 (m, 1H), 3.84 (d, *J* = 14.0 Hz, 1H), 3.74 (t, *J* = 9.0 Hz, 1H), 3.63–3.58 (m, 1H), 2.90–2.84 (m, 1H), 2.79 (t, *J* = 9.2 Hz, 1H), 2.45 (s, 3H), 2.34–2.28 (m, 2H), 2.24–2.17 (m, 1H);

^{13}C NMR (151 MHz, $CDCl_3$): δ 143.7, 142.5, 136.7, 135.4, 131.9, 130.9, 129.6, 128.5, 128.1, 128.1, 127.0, 126.9, 54.8, 52.2, 40.5, 32.5, 26.5, 21.6;

HRMS: (ESI) calcd for $C_{22}H_{24}NO_2S^+$ ($[M+H]^+$): 366.1522; found: 366.1527;

HPLC conditions: AD-H column (15% ^{i}PrOH in hexane, 1.0 mL/min, λ = 254 nm, 30 °C), t_R (major) = 9.3 min, t_R (minor) = 11.7 min;

Optical Rotation: $[\alpha]^{25}_D$ = -78.5 (*c* = 0.10, $CHCl_3$) for 94% ee;

Absolute stereochemistry was determined through analogy with **7aa**.

<Chromatogram>

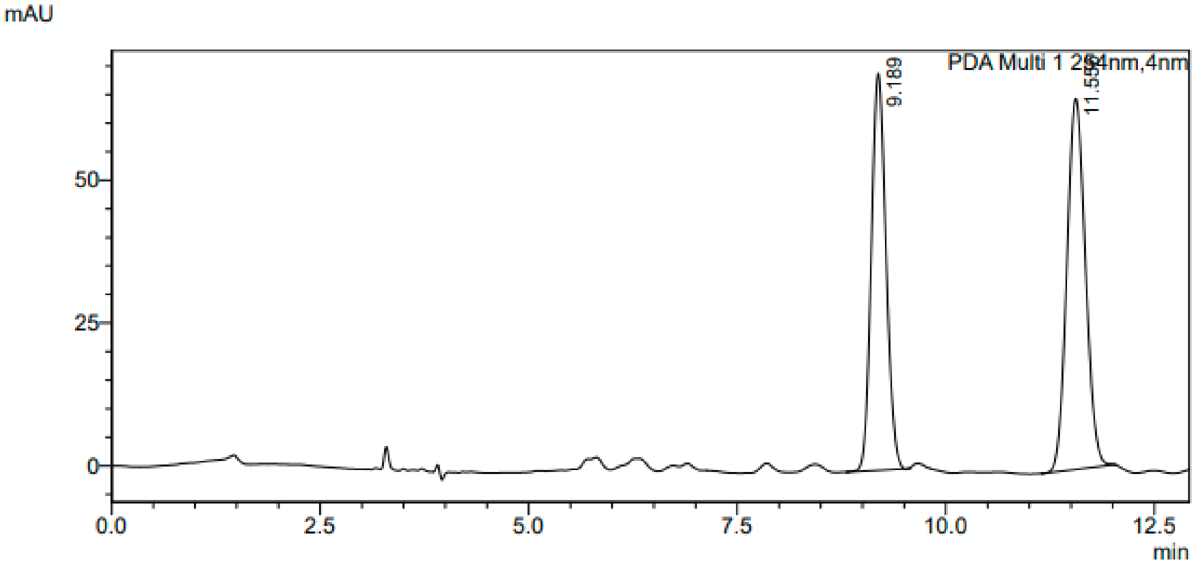

<Peak Table>

PDA Ch1 254nm

Peak#	Ret. Time	Area	Height	Conc.	Unit	Mark	Name
1	9.189	846452	69451	0.000		M	
2	11.556	1001561	64938	0.000		M	
Total		1848013	134389				

Supplementary Figure 132. HPLC spectrum of racemic-**8**

<Chromatogram>

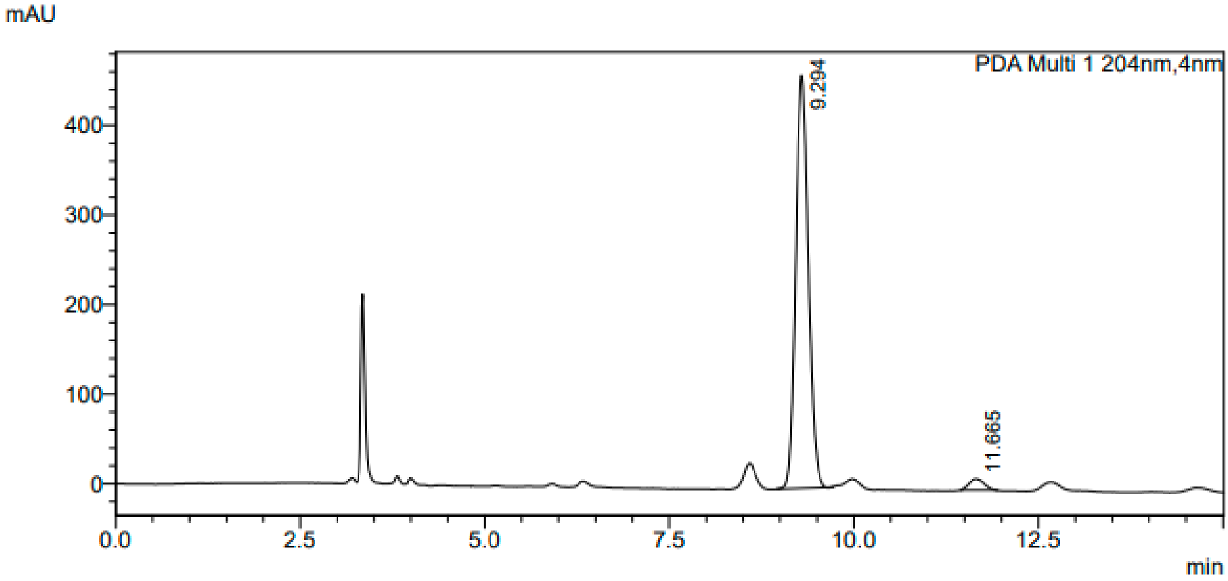

<Peak Table>

PDA Ch1 204nm

Peak#	Ret. Time	Area	Height	Conc.	Unit	Mark	Name
1	9.294	5550512	460843	0.000		M	
2	11.665	172562	12283	0.000		M	
Total		5723074	473126				

Supplementary Figure 133. HPLC spectrum of (*R*)-**8**

7.2 Synthesis of (*R*,*Z*)-3-(1,3-diphenylpropylidene)-4-phenethyl-1-tosylpyrrolidine (9aa)

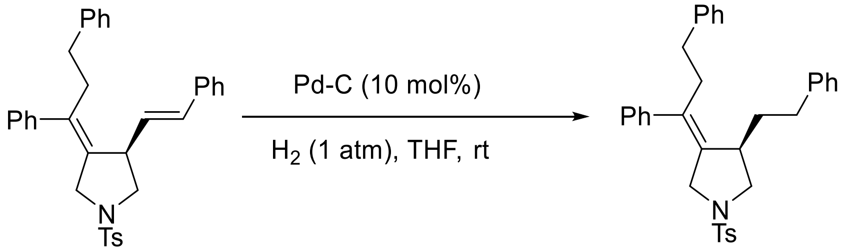

(***R***)-**7aa**, 95% ee **9aa**

To an oven-dried Schlenk tube was added (*R*)-**7aa** (0.05 mmol, 25.8 mg) and Pd/C (10% w, 0.005 mmol, 0.5 mg). The tube was evacuated and refilled with H_2 three times. Under a H_2 airbag, THF (1 mL) was added and the reaction mixture was stirred at room temperature for 1 hour. The reaction mixture was filtered through a celite pad, and the filtrate was concentrated and purified by chromatography on silica gel, eluting with PE/EA = 10/1 to afford **9aa** (23.7 mg, 91% yield).

^{1}H NMR (600 MHz, $CDCl_3$): δ 7.66–7.61 (m, 2H), 7.38–7.33 (m, 2H), 7.32–7.26 (m, 5H), 7.23–7.17 (m, 3H), 7.16–7.09 (m, 3H), 7.07–7.03 (m, 2H), 6.96–6.92 (m, 2H), 3.83 (dd, *J* = 14.3, 1.4 Hz, 1H), 3.47–3.39 (m, 2H), 3.03 (dd, *J* = 9.7, 6.3 Hz, 1H), 2.73–2.65 (m, 2H), 2.53–2.46 (m, 3H), 2.43 (d, *J* = 4.8 Hz, 5H), 1.69–1.64 (m, 1H), 0.89–0.86 (m, 1H);

^{13}C NMR (151 MHz, $CDCl_3$): δ 143.4, 141.4, 141.1, 141.0, 136.0, 134.6, 132.9, 129.6, 128.5, 128.4, 128.4, 128.2, 127.8, 127.7, 127.1, 126.0, 125.9, 51.8, 50.3, 40.2, 36.5, 35.1, 34.3, 33.6, 21.5;

HRMS: (ESI) calcd for $C_{34}H_{36}NO_2S^+$ ($[M+H]^+$): 522.2461; found: 522.2469.

7.3 Synthesis of (3*S*,4*R*)-3-((*S*)-1,3-diphenylpropyl)-4-phenethyl-1-tosylpyrrolidine (10aa)

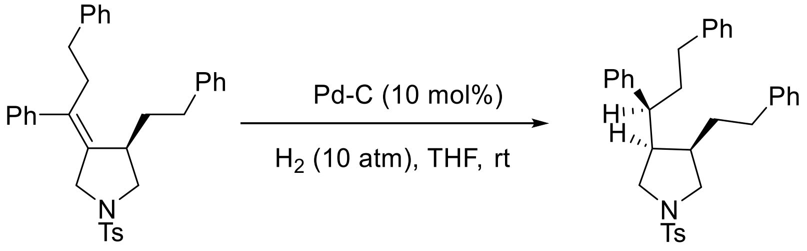

9aa **10aa**

To an oven-dried Schlenk tube was added **9aa** (0.04 mmol, 20.9 mg), Pd/C (10% w, 0.004 mmol, 0.4 mg) and THF (1 mL). The hydrogenation was performed at room temperature at a

hydrogen pressure of 10 atm for 8 hours. After carefully releasing the hydrogen gas. The reaction mixture was filtered through a celite pad, and the filtrate was concentrated and purified by chromatography on silica gel, eluting with PE/EA = 10/1 to afford the **10aa** (19.5 mg, 93% yield, > 20/1 d.r.).

^{1}H NMR (600 MHz, $CDCl_3$): δ 7.73–7.66 (m, 2H), 7.34–7.28 (m, 4H), 7.28–7.23 (m, 3H), 7.21–7.12 (m, 4H), 7.06–6.99 (m, 4H), 6.86–6.78 (m, 2H), 3.45 (dd, *J* = 10.0, 7.6 Hz, 1H), 3.33 (dd, *J* = 9.9, 7.5 Hz, 1H), 3.04 (dd, *J* = 10.0, 6.8 Hz, 1H), 2.87 (dd, *J* = 9.9, 6.4 Hz, 1H), 2.44 (s, 3H), 2.37–2.28 (m, 2H), 2.26–2.13 (m, 3H), 1.99–1.91 (m, 1H), 1.89–1.80 (m, 2H), 1.69–1.62 (m, 1H), 1.12–1.01 (m, 2H);

^{13}C NMR (151 MHz, $CDCl_3$): δ 143.5, 142.6, 141.9, 141.2, 133.1, 129.7, 128.7, 128.4, 128.3, 128.3, 128.2, 128.2, 127.7, 126.8, 125.9, 125.8, 52.8, 51.8, 49.9, 48.6, 41.7, 35.4, 35.0, 33.8, 33.5, 21.6;

HRMS: (ESI) calcd for $C_{34}H_{36}NO_2S^+[M+H]^+$ 524.2618; found 524.2617.

7.4 Enantioselective synthesis of the antiepileptic drug Brivaracetam

(*R*,*Z*)-3-(1,3-diphenylpropylidene)-1-(4-methoxybenzyl)-4-((*E*)-prop-1-en-1-yl)pyrrolidin-2-one (7la)

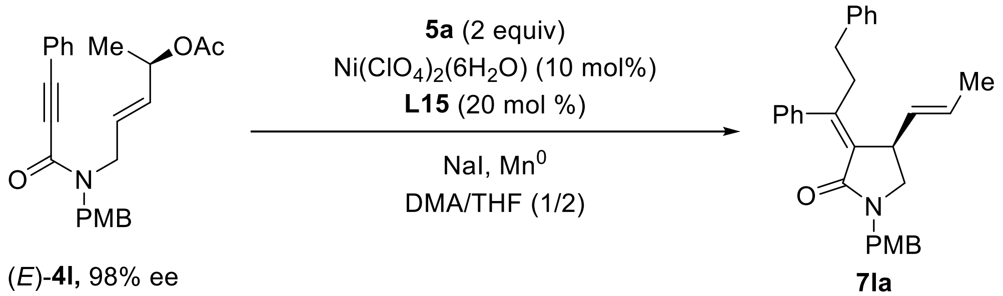

An oven-dried sealed tube equipped with a PTFE-coated stir bar was charged with $Ni(ClO)_2{\bullet}6H_2O$ (0.02 mmol, 7.4 mg), **L15** (0.04 mmol, 8.0 mg), NaI (0.05 mmol, 15.0 mg) and anhydrous DMA (1340 μL). This reaction mixture was stirred at room temperature for 1 hour in an argon-filled glovebox. (*E*)-**4l** (0.2 mmol, 78.2 mg), (2-bromoethyl)benzene **5a** (0.4 mmol, 74.0 mg), Mn powder (0.3 mmol, 33.0 mg) and anhydrous THF (2660 μL) were then added. The sealed tube was sealed and removed from the glovebox. The resulting reaction mixture was allowed to stir at 20 °C for 24 hours. The reaction was quenched by the addition

of a saturated aqueous of NH_4Cl (10 mL) and EA (20 mL). The organic layer was separated and the aqueous layer was extracted with EA (20 mL × 3). The combined organic layers were washed with brine, dried over Na_2SO_4, filtered and concentrated. Purification by chromatography on silica gel, eluting with PE/EA = 5/1 afforded the desired product **7la** as a colorless oil (56.9 mg, 65% yield).

^{1}H NMR (600 MHz, $CDCl_3$): δ 7.42–7.37 (m, 2H), 7.35–7.30 (m, 1H), 7.26–7.22 (m, 4H), 7.18–7.14 (m, 1H), 7.12–7.05 (m, 4H), 6.82 (dd, *J* = 8.3, 1.2 Hz, 2H), 5.57–5.50 (m, 1H), 5.42–5.36 (m, 1H), 4.46 (d, *J* = 14.8 Hz, 1H), 4.22 (d, *J* = 14.6 Hz, 1H), 3.78 (s, 3H), 3.37 (t, *J* = 7.7 Hz, 1H), 3.31 (dd, *J* = 9.7, 7.7 Hz, 1H), 2.84 (dd, *J* = 9.7, 1.8 Hz, 1H), 2.75–2.67 (m, 2H), 2.64–2.55 (m, 1H), 2.55–2.46 (m, 1H), 1.69–1.65 (m, 3H);

^{13}C NMR (151 MHz, $CDCl_3$): δ 166.6, 158.9, 148.2, 141.6, 140.0, 131.7, 129.6, 129.5, 128.6, 128.3, 128.3, 128.1, 127.6, 127.1, 125.9, 125.8, 113.8, 55.2, 49.7, 46.0, 38.9, 38.6, 33.3, 17.8;

HRMS: (ESI) calcd for $C_{30}H_{32}NO_2^+[M+H]^+$ 438.2427; found 438.2427;

HPLC conditions: IA-H column (15% ^{i}PrOH in hexane, 1.0 mL/min, λ = 190 nm, 30 °C), t_R (major) = 6.6 min, t_R (minor) = 9.0 min;

Optical Rotation: $[\alpha]^{25}_D$ = -12.3 (*c* = 0.50, $CHCl_3$) for 82% ee;

Absolute stereochemistry was determined through analogy with **7aa**.

<Chromatogram>

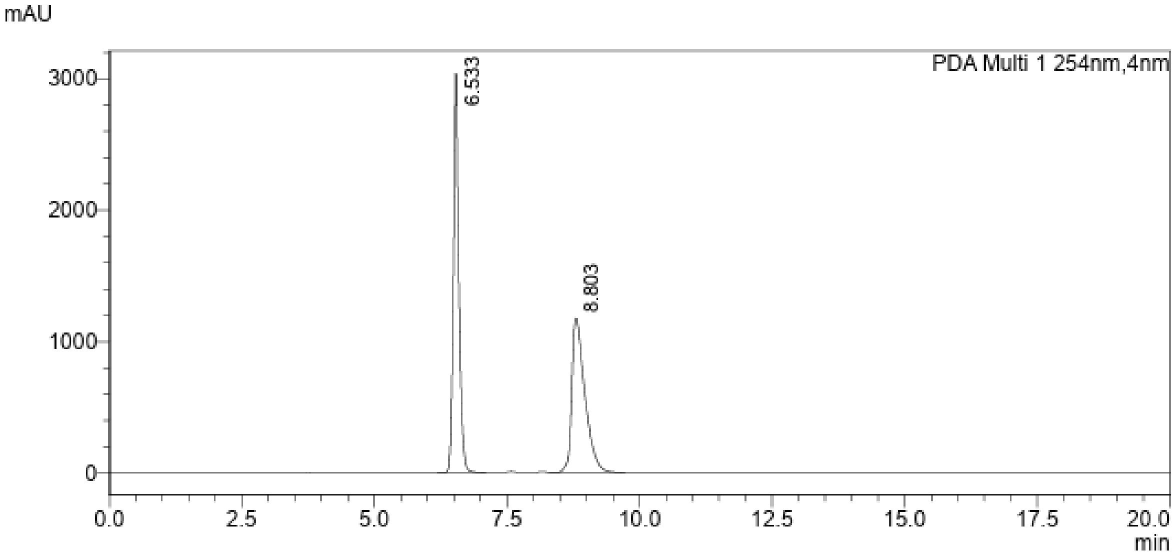

<Peak Table>

PDA Ch1 254nm

Peak#	Ret. Time	Area	Height	Conc.	Unit	Mark	Name
1	6.533	21547792	3040361	0.000		M	
2	8.803	20251491	1175446	0.000		M	
Total		41799283	4215806				

Supplementary Figure 134. HPLC spectrum of racemic-**7la**

<Chromatogram>

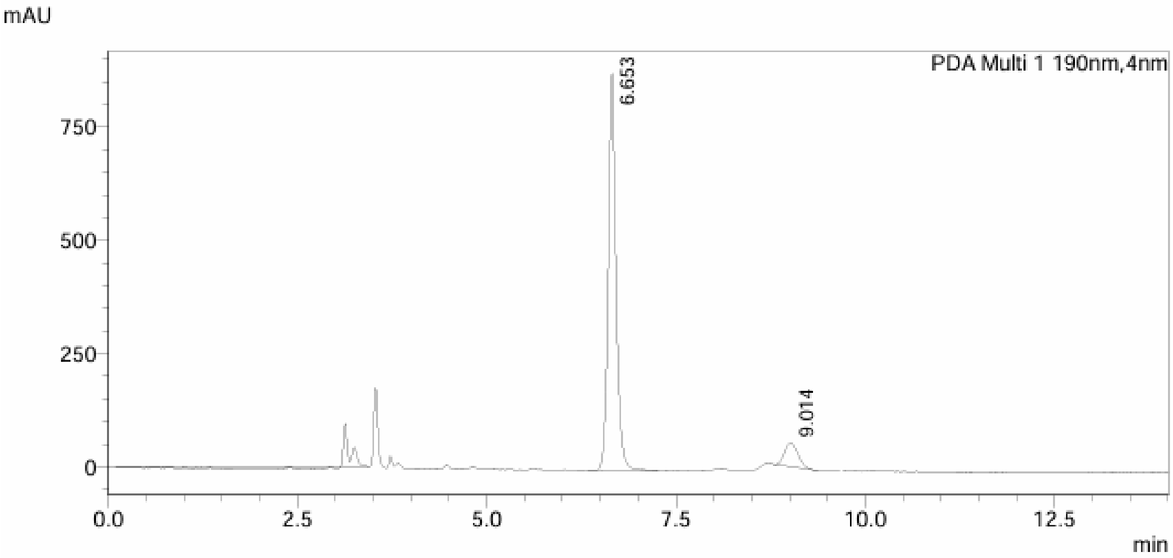

<Peak Table>

PDA Ch1 190nm

Peak#	Ret. Time	Area	Height	Conc.	Unit	Mark	Name
1	6.653	6286215	876890	0.000		M	
2	9.014	640455	52529	0.000		M	
Total		6926670	929419				

Supplementary Figure 135. HPLC spectrum of (*R*)-**7la**

(*R*,*Z*)-3-(1,3-diphenylpropylidene)-1-(4-methoxybenzyl)-4-propylpyrrolidin-2-one (9la)

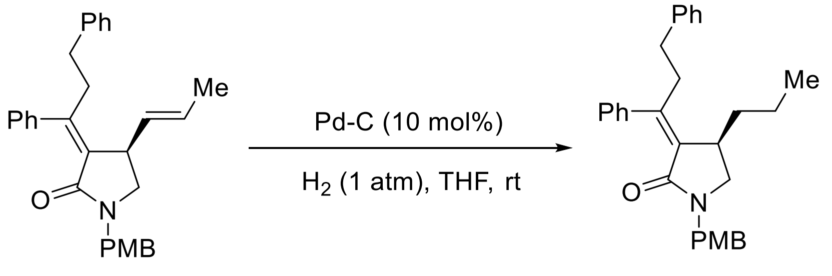

To an oven-dried Schlenk tube was added **7la** (0.13 mmol, 56.9 mg) and Pd/C (10% w, 0.013 mmol, 1.3 mg). The tube was evacuated and refilled with H_2 three times. Under a H_2 airbag, THF (3 mL) was added and the reaction mixture was stirred at room temperature for 1 hour. The reaction mixture was filtered through a celite pad, and the filtrate was concentrated to give the **9la** (56.6 mg, 99% yield).

^{1}H NMR (600 MHz, $CDCl_3$): δ 7.42–7.38 (m, 2H), 7.35–7.31 (m, 1H), 7.28–7.23 (m, 4H), 7.21–7.15 (m, 1H), 7.15–7.09 (m, 4H), 6.87–6.81 (m, 2H), 4.51 (d, *J* = 14.5 Hz, 1H), 4.18 (d, *J* = 14.5 Hz, 1H), 3.79 (s, 3H), 3.18 (dd, *J* = 9.7, 7.0 Hz, 1H), 2.81 (dd, *J* = 9.8, 1.2 Hz, 1H), 2.79–2.68 (m, 3H), 2.66–2.50 (m, 2H), 1.45–1.37 (m, 2H), 1.35–1.28 (m, 1H), 1.24–1.16 (m, 1H), 0.87 (t, *J* = 7.3 Hz, 3H);

^{13}C NMR (151 MHz, $CDCl_3$): δ 166.8, 158.9, 145.9, 141.5, 139.8, 132.2, 129.6, 128.7, 128.4, 128.3, 128.2, 127.6, 127.0, 126.0, 113.8, 55.2, 48.3, 46.1, 38.7, 37.6, 35.5, 33.7, 19.7, 14.0;

HRMS: (ESI) calcd for $C_{30}H_{34}NO_2^+$ ($[M+H]^+$): 440.2584; found: 440.2587.

(4*S*)-3-hydroxy-1-(4-methoxybenzyl)-4-propylpyrrolidin-2-one (11)

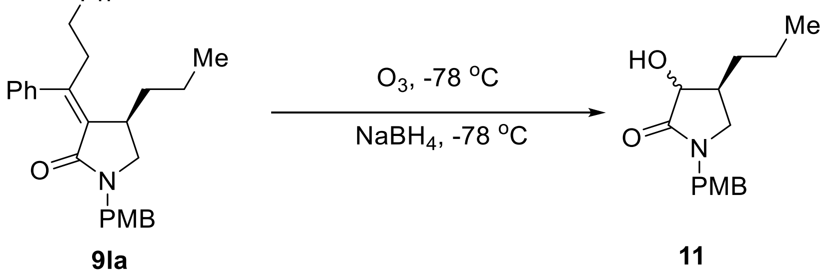

In an oven-dried Schlenk tube, a solution of **9la** (0.13 mmol, 56.6 mg) in DCM (2 mL) was cooled down to -78°C and ozone was bubbled into the reaction mixture until a persistent blue colour appeared. Oxygen was then bubbled in to remove excess ozone and $NaBH_4$ (0.65 mmol,

24.1 mg) was added in one portion. The mixture stirred at -78°C overnight. H_2O was then was added at room temperature. The aqueous layer was extracted with EA (3×5 mL) and the combined organic layers was concentrated under vacuum. The residue was purified by chromatography on silica gel, eluting with DCM/MeOH = 40/1 to give the desired product **11** (24.6 mg, 72% yield, 2/1 d.r.).

^{1}H NMR (600 MHz, $CDCl_3$): δ 7.15 (d, J = 8.4 Hz, 2H), 6.86 (dd, J = 8.7, 2.2 Hz, 2H), 4.43–4.35 (m, 2H), 4.33 (d, J = 7.2 Hz, 0.3H), 4.00 (d, J = 9.1 Hz, 0.7H), 3.80 (d, J = 1.6 Hz, 3H), 3.65 (s, 0.7H), 3.57 (s, 0.3H), 3.26 (dd, J = 9.8, 8.2 Hz, 0.7H), 3.22 (dd, J = 9.9, 6.7 Hz, 0.3H), 2.97 (dd, J = 9.9, 4.4 Hz, 0.3H), 2.78 (t, J = 9.4 Hz, 0.7H), 2.38–2.29 (m, 0.3H), 2.21–2.14 (m, 0.7H), 1.72–1.62 (m, 1H), 1.43–1.31 (m, 2.7H), 1.20–1.12 (m, 0.3H), 0.92–0.85 (m, 3H);

^{13}C NMR (151 MHz, $CDCl_3$): δ 174.6, 174.5, 159.19, 159.16, 129.50, 129.48, 127.9, 127.8, 114.12, 114.07, 75.5, 71.8, 55.3, 48.9, 48.5, 46.3, 46.2, 41.6, 37.3, 34.4, 28.4, 20.5, 20.4, 14.14, 14.10;

HRMS: (ESI) calcd for $C_{15}H_{22}NO_3^+$ ($[M+H]^+$): 264.1594; found: 264.1590.

(*R*)-1-(4-methoxybenzyl)-4-propylpyrrolidin-2-one (12)

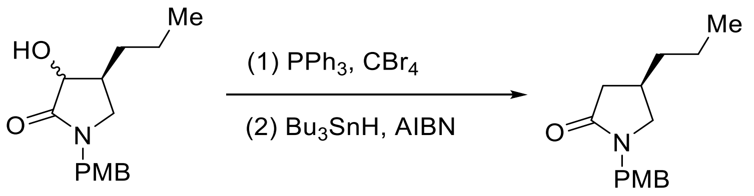

11 **12**

Following the reported literature procedure from Baudoin and co-workers.[8]

To a solution of **11** (0.09 mmol, 24.6 mg) in dry DCM (2 mL) was added CBr_4 (0.46 mmol, 155.2 mg) and PPh_3 (0.46 mmol, 120.5 mg) under an argon atmosphere. The reaction mixture was stirred at room temperature for 3 hours. The reaction mixture was evaporated under reduced pressure and the residue was purified by flash chromatography on silica (PE/EA = 2/1) to afford 24.9 mg of the crude bromide product.

To a solution of the bromide (0.08 mmol, 24.9 mg) in toluene (2 mL) was added Bu_3SnH (0.24 mmol, 69.9 mg) and AIBN (0.008 mmol, 1.3 mg). The reaction mixture was heated at reflux under an argon atmosphere for 2 hours. The reaction mixture was poured into a saturated

aqueous of sodium hydrogen carbonate and extracted three times with ethyl acetate. The combined organic extracts were dried over Na_2SO_4, filtered and concentrated under reduced pressure. The residue was purification by chromatography on silica gel, eluting with PE/EA = 2/1 to give the desired product **12** (15.1 mg, 68% yield, 81% ee).

^{1}H NMR (600 MHz, $CDCl_3$): δ 7.20–7.13 (m, 2H), 6.89–6.83 (m, 2H), 4.40 (d, J = 14.5 Hz, 1H), 4.34 (d, J = 14.5 Hz, 1H), 3.80 (s, 3H), 3.32 (dd, J = 9.7, 8.0 Hz, 1H), 2.85 (dd, J = 9.7, 6.8 Hz, 1H), 2.56 (dd, J = 16.7, 8.7 Hz, 1H), 2.35–2.26 (m, 1H), 2.10 (dd, J = 16.7, 7.9 Hz, 1H), 1.39–1.33 (m, 2H), 1.32–1.27 (m, 2H), 0.88 (t, J = 7.2 Hz, 3H);

^{13}C NMR (151 MHz, $CDCl_3$): δ 174.4, 159.0, 129.4, 128.6, 114.0, 55.3, 52.3, 45.9, 37.8, 36.9, 31.4, 20.5, 14.0;

HRMS: (ESI) calcd for $C_{32}H_{34}NO_2S^+$ ($[M+H]^+$): 248.1645; found: 248.1638;

HPLC conditions: AD-H column (10% ^{i}PrOH in hexane, 1.0 mL/min, λ = 254 nm, 30 °C), t_R (minor) = 11.4 min, t_R (major) = 10.2 min;

Optical Rotation: $[\alpha]^{25}_D$ = -32.6 (c = 0.09, $CHCl_3$) for 81% ee.

<Chromatogram>

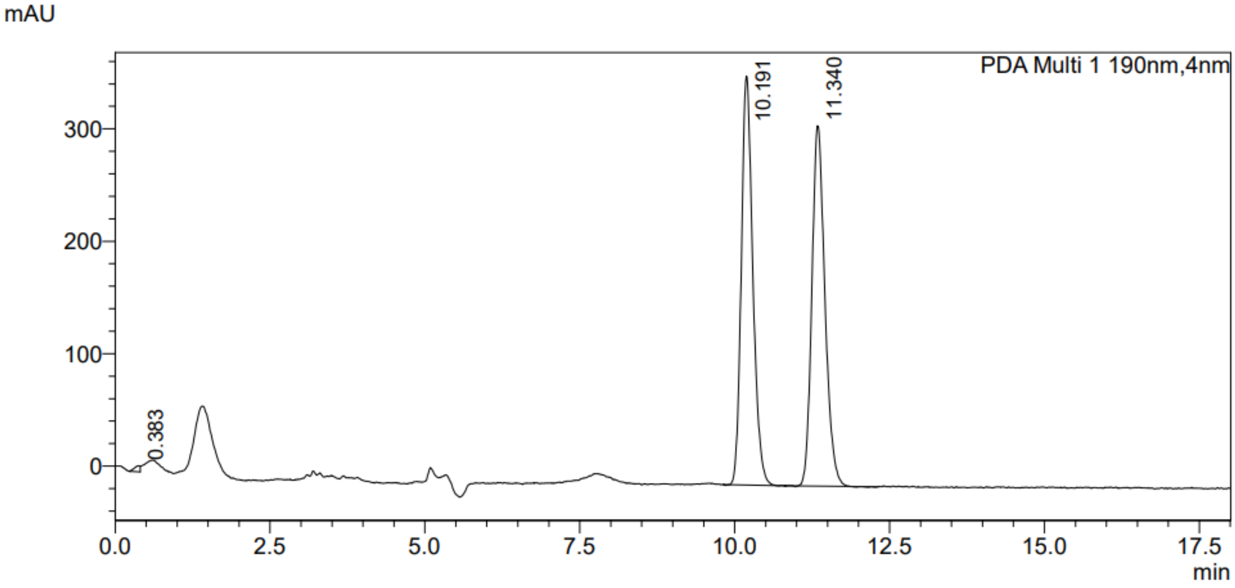

<Peak Table>

PDA Ch1 190nm

Peak#	Ret. Time	Area	Height	Conc.	Unit	Mark	Name
1	0.383	34625	5412	0.000			
2	10.191	4703486	363785	0.000		M	
3	11.340	4702352	320560	0.000		M	
Total		9440462	689757				

Supplementary Figure 136. HPLC spectrum of racemic-**12**

<Chromatogram>

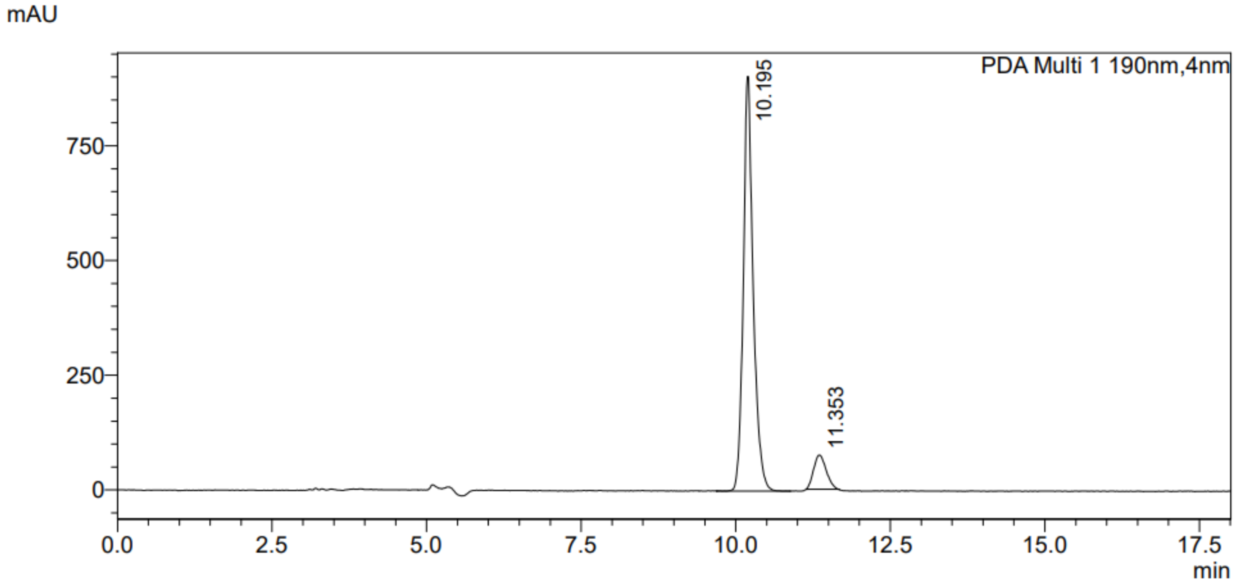

<Peak Table>

PDA Ch1 190nm

Peak#	Ret. Time	Area	Height	Conc.	Unit	Mark	Name
1	10.195	10050381	903579	0.000		M	
2	11.353	1054709	74433	0.000		M	
Total		11105090	978011				

Supplementary Figure 137. HPLC spectrum of (*R*)-**12**

10. Mechanistic Studies

10.1 Alkylative and arylative cyclization reaction using 1,7-enyne

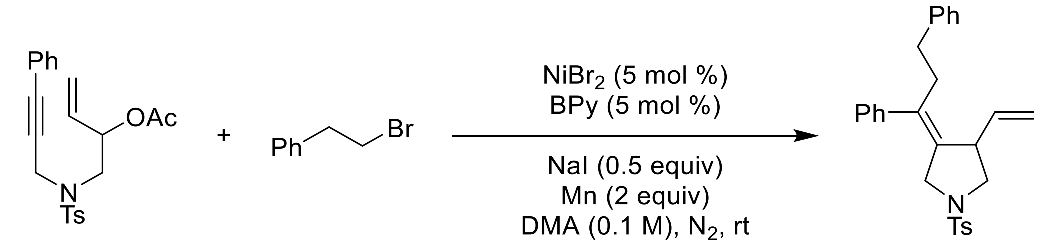

1o **2a** **6aa**

6aa was prepared according to general procedure **2.2** in 0.5 mmol scale using **1o** (0.5 mmol, 198.7 mg) as substrate. Purification by silica gel column chromatography (PE/EA = 10/1) give **6aa** as a white solid (154.7 mg, 70% yield). This result indicates that the alkylative cyclization reaction involves a π-allylic nickel intermediate.

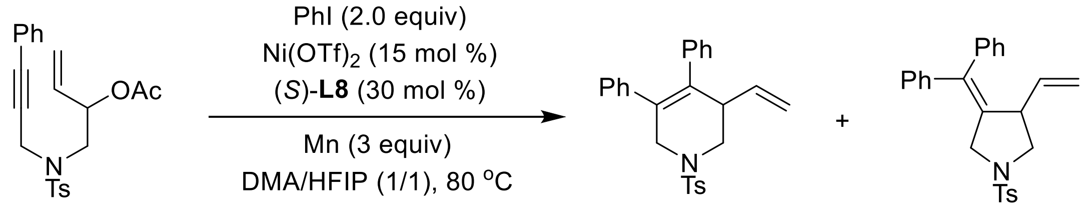

1o **3aa**, 0% **3aa'**, 0%

The reaction was performed according to general procedure **2.1** using 1,7-enyne **1o** as substrate, neither product **3aa** nor product **3aa’** was detected. This result indicates that the arylative cyclization reaction may not involve an π-allylic nickel intermediate.

10.2 Reaction with alkenyl iodine

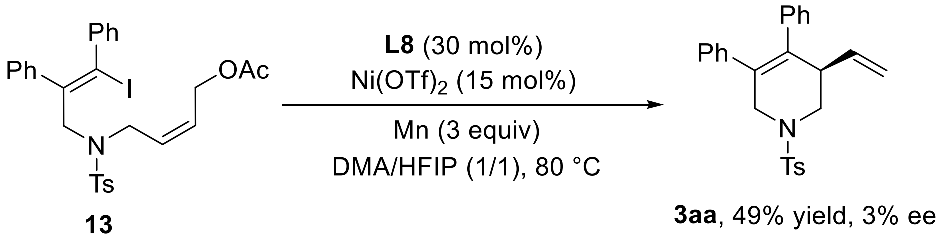

An oven-dried sealed tube equipped with a PTFE-coated stir bar was charged with $Ni(OTf)_2$ (0.015 mmol, 5.3 mg), (*S*)-**L8** (0.03 mmol, 10.1 mg) and anhydrous DMA/HFIP (1:1, 1 mL). This reaction mixture was stirred at room temperature for 1 hour in an argon-filled glovebox. Mn (16.5 mg, 0.3 mmol), alkenyl iodine **13** (0.1 mmol, 60.0 mg), and DMA/HFIP (1:1, 1 mL) were then added. The sealed tube was sealed and removed from the glovebox and the resulting reaction mixture was stirred at 80 °C for 12 hours. Then the reaction was quenched by the addition of H_2O (10 mL) and EtOAc (20 mL). The organic layer was separated and the aqueous layer was extracted with EtOAc (20 mL × 3). The combined organic layers were washed with brine, dried over Na_2SO_4, filtered and concentrated. Purification by chromatography on silica gel, eluting with PE/EtOAc (20/1) to give the desired product **3aa** (19.5 mg, 49% yield, 3% ee).

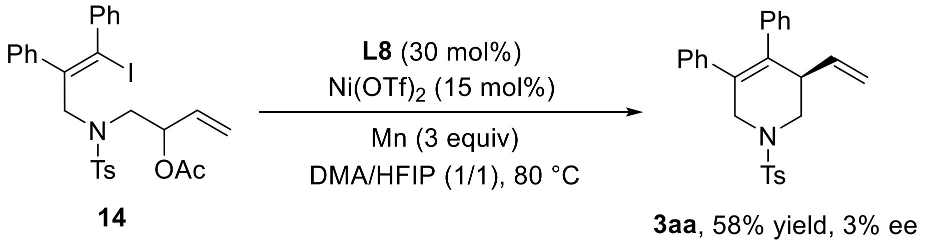

An oven-dried sealed tube equipped with a PTFE-coated stir bar was charged with $Ni(OTf)_2$ (0.015 mmol, 5.3 mg), (*S*)-**L8** (0.03 mmol, 10.1 mg) and anhydrous DMA/HFIP (1:1, 1 mL). This reaction mixture was stirred at room temperature for 1 hour in an argon-filled glovebox. Mn (16.5 mg, 0.3 mmol), alkenyl iodine **14** (0.1 mmol, 60.0 mg), and DMA/HFIP (1:1, 1 mL) were then added. The sealed tube was sealed and removed from the glovebox and the resulting reaction mixture was stirred at 80 °C for 12 hours. Then the reaction was quenched by the addition of H_2O (10 mL) and EtOAc (20 mL). The organic layer was separated and the aqueous layer was extracted with EtOAc (20 mL × 3). The combined organic layers were washed with

brine, dried over Na_2SO_4, filtered and concentrated. Purification by chromatography on silica gel, eluting with PE/EtOAc (20/1) to give the desired product **3aa** (24.0 mg, 58% yield, 3% ee).

10.3 Crossover experiment

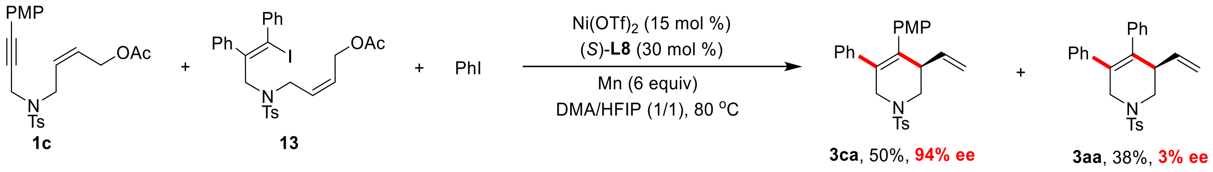

An oven-dried sealed tube equipped with a PTFE-coated stir bar was charged with $Ni(OTf)_2$ (0.015 mmol, 5.3 mg), (*S*)-**L8** (0.03 mmol, 10.1 mg) and anhydrous DMA/HFIP (1:1, 1 mL). This reaction mixture was stirred at room temperature for 1 hour in an argon-filled glovebox. Mn (16.5 mg, 0.3 mmol), enyne **1c** (0.1 mmol, 42.7 mmg), alkenyl iodine **13** (0.1 mmol, 60.0 mg), and DMA/HFIP (1:1, 1 mL) were then added. The sealed tube was sealed and removed from the glovebox and the resulting reaction mixture was stirred at 80 ºC for 12 hours. Then the reaction was quenched by the addition of H_2O (10 mL) and EtOAc (20 mL). The organic layer was separated and the aqueous layer was extracted with EtOAc (20 mL × 3). The combined organic layers were washed with brine, dried over Na_2SO_4, filtered and concentrated. Purification by chromatography on silica gel, eluting with PE/EtOAc (20/1) to give the product **3ca** (22.3 mg, 50% yield, 94% ee) and **3aa** (15.8 mg, 38% yield, 3% ee).

10.4 Reaction with Ar-Ni complex

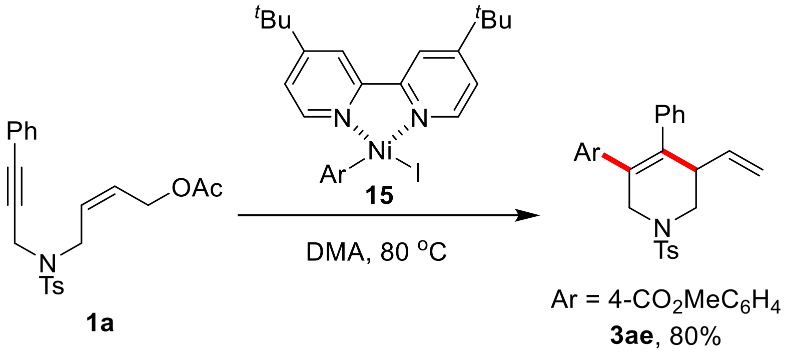

To a Schlenk tube containing a magnetic stir bar was added **1a** (79.9 mg, 0.2 mmol), Ni-complex **15** (163.4 mg, 0.2 mmol)[9] and anhydrous DMA (2 mL). The tube was degassed and refilled with N_2 for three times. The reaction mixture was allowed to stir at 80 °C under N_2 for 24 hours. Then the reaction was quenched by the addition of H_2O (5 mL) and EtOAc (10 mL). The organic layer was separated and the aqueous layer was extracted with EtOAc (10 mL × 3). The combined organic layers were washed with brine, dried over Na_2SO_4, filtered and concentrated. Purification by column chromatography (10% EtOAc in petroleum ether) gave product **3ae** as a white solid (76.0 mg, 80% yield).

10.5 quenching alkenyl nickel with water

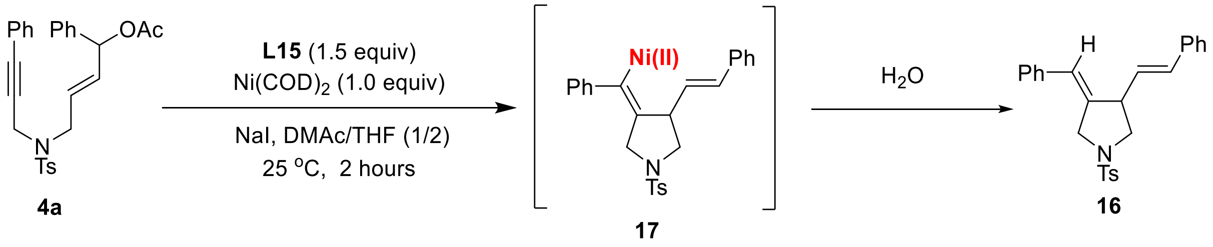

An oven-dried sealed tube equipped with a PTFE-coated stir bar was charged with $Ni(COD)_2$ (0.1 mmol, 27.6 mg), **L15** (0.15 mmol, 30.3 mg), NaI (0.05 mmol, 7.5 mg) and anhydrous DMA (1 mL). This reaction mixture was stirred at room temperature for 1 hour in an argon-filled glovebox. **4a** (0.1 mmol), Mn powder (0.3 mmol, 16.5 mg) and anhydrous THF (2 mL) were then added. The sealed tube was sealed and removed from the glovebox. The reaction mixture was stirred at 25 °C for 2 hours. Then the reaction was quenched by the addition of H_2O (5 mL) and stirred for another 2 hours. The organic layer was separated and the aqueous layer was extracted with EtOAc (20 mL × 3). The combined organic layers were washed with

brine, dried over Na_2SO_4, filtered and concentrated. Purification by chromatography on silica gel, eluting with PE/EtOAc (20/1~5/1) gave the product **16** as a white solid (34.5 mg, 77% yield).

^{1}H NMR (600 MHz, $CDCl_3$): δ 7.83–7.70 (m, 2H), 7.40–7.30 (m, 8H), 7.25 (m, J = 7.2 Hz, 2H), 7.17–7.13 (m, 2H), 6.52 (d, J = 15.7 Hz, 1H), 6.25 (d, J = 2.5 Hz, 1H), 5.94 (dd, J = 15.7, 8.5 Hz, 1H), 4.38 (d, J = 15.0 Hz, 1H), 4.06 (d, J = 15.0 Hz, 1H), 3.77–3.68 (m, 1H), 3.63 (t, J = 8.4 Hz, 1H), 2.95 (t, J = 9.0 Hz, 1H), 2.43 (s, 3H);

^{13}C NMR (151 MHz, $CDCl_3$): δ 143.8, 139.3, 136.5, 136.3, 133.5, 132.7, 129.8, 128.6, 128.6, 128.1, 127.8, 127.8, 127.3, 127.2, 126.3, 124.5, 52.3, 50.8, 48.7, 21.5;

HRMS: (ESI) calcd for $C_{26}H_{26}NO_2S^+$ ($[M+H]^+$): 416.1679; found: 416.1677.

10.6 radical trapping experiments

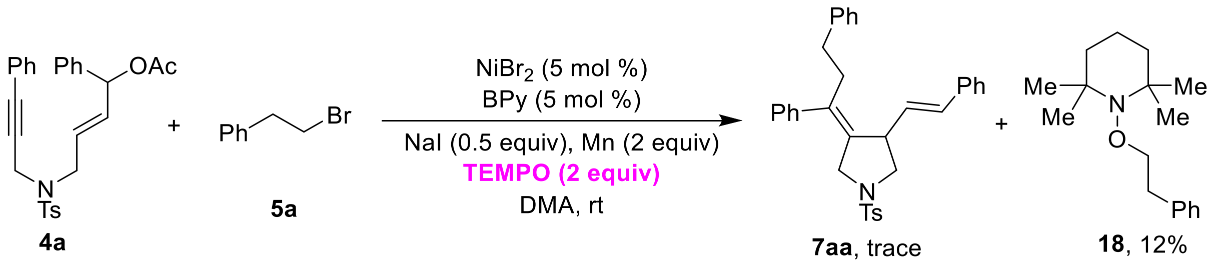

An oven-dried sealed tube equipped with a PTFE-coated stir bar was charged with $NiBr_2$ (0.05 mmol, 1.1 mg), BPy (0.05 mmol, 0.05 mg), NaI (0.05 mmol, 7.5 mg), Mn (0.2 mmol, 11.0 mg), enyne **4a** (0.1 mmol, 47.4 mg), **5a** (0.2 mmol, 37.0 mg), TEMPO (0.2 mmol, 31.2 mg) and anhydrous DMA (1 mL). The sealed tube was sealed and removed from the glovebox. The reaction mixture was stirred at 20 °C for 12 hours. The reaction was almost completely suppressed and the TEMPO-trapped phenylethane product **18** was isolated in 12% yield.

^{1}H NMR (600 MHz, $CDCl_3$) δ 7.29-7.26 (m, 2H), 7.25-7.21 (m, 2H), 7.22-7.17 (m, 1H), 3.94 (t, J = 7.0 Hz, 2H), 2.82 (t, J = 7.0 Hz, 2H), 1.55-1.49 (m, 1H), 1.44-1.39 (m, 4H), 1.34-1.26 (m, 2H), 1.07 (s, 12H);

^{13}C NMR (151 MHz, $CDCl_3$) δ 139.6, 129.1, 128.1, 125.9, 77.5, 77.2, 59.7, 39.6, 35.4, 32.9, 20.1, 17.1.

10.7 Stoichiometric experiment

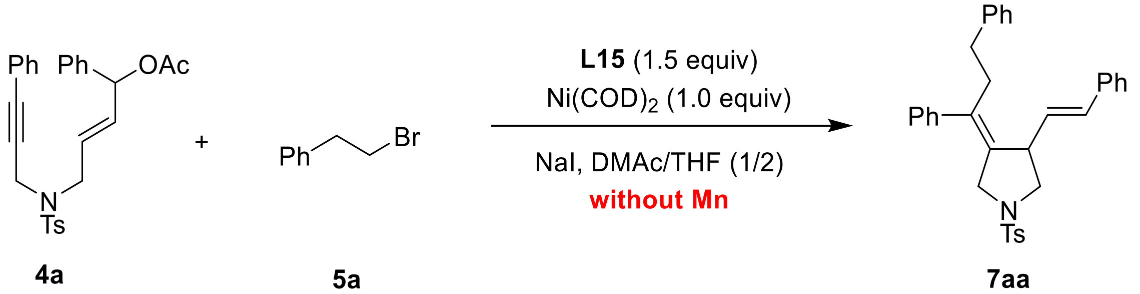

An oven-dried sealed tube equipped with a PTFE-coated stir bar was charged with Ni(COD)$_2$ (0.1 mmol, 27.5 mg), **L15** (0.15 mmol, 30.3 mg), NaI (0.05mmol, 7.5 mg) and anhydrous DMA (1 mL). This reaction mixture was stirred at room temperature for 1 hour in an argon-filled glovebox. Racemic enyne **4a** (0.1 mmol, 47.4 mg), **5a** (0.2 mmol, 37.0 mg) and anhydrous THF (2 mL) were then added. The sealed tube was sealed and removed from the glovebox. The reaction mixture was stirred at 20 °C for 12 hours. Then the reaction was quenched by the addition of H_2O (10 mL). The organic layer was separated and the aqueous layer was extracted with EtOAc (20 mL × 3). The combined organic layers were washed with brine, dried over Na_2SO_4, filtered and concentrated. Purification by chromatography on silica gel, eluting with PE/EtOAc (20/1) to give the racemic product **7aa** (34.3 mg, 66% yield).

10.8 Radical ring-opening reaction

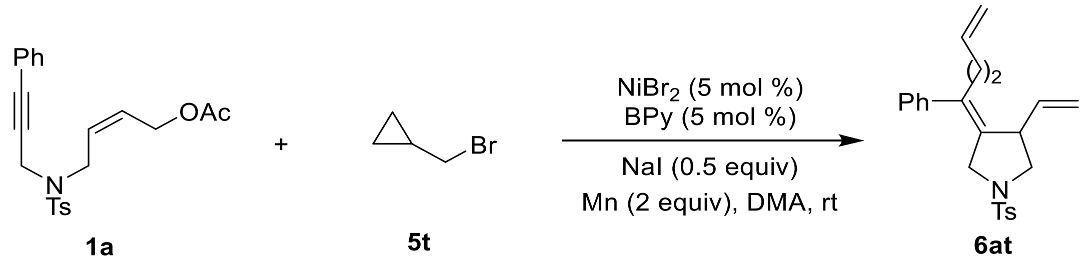

6at was prepared according to general procedure **2.2** on 0.5 mmol scale, using **1a** (198.7 mg, 0.5 mmol) and (bromomethyl)cyclopropane **5t** (81.0 mg, 0.6 mmol) as substrates. Purification by silica gel column chromatography (PE/EA = 10/1) gave **6at** as a yellow oil (102.3 mg, 52% yield).

^{1}H NMR (400 MHz, $CDCl_3$): δ 7.61 (d, *J* = 8.4 Hz, 2H), 7.36–7.27 (m, 5H), 7.07–6.95 (m, 2H), 5.83–5.57 (m, 2H), 5.11 (d, *J* = 16.8 Hz, 1H), 5.05 (d, *J* = 10.4 Hz, 1H), 4.95–4.75 (m, 2H), 3.76 (dd, *J* = 14.0, 1.2 Hz, 1H), 3.56–3.40 (m, 2H), 3.35–3.22 (m, 2H), 2.43 (s, 3H), 2.40–2.28 (m, 2H), 1.96–1.81 (m, 2H);

^{13}C NMR (100 MHz, $CDCl_3$): δ 143.5, 141.1, 137.9, 137.8, 136.9, 133.1, 132.7, 129.6, 128.5, 127.8, 127.6, 127.1, 115.3, 114.7, 53.6, 50.9, 45.1, 33.6, 31.8, 29.3, 21.5;

IR (neat): 3360, 2920, 1636, 1347, 1161, 1093, 914, 813, 705, 662, 548 cm^{-1};

HRMS-ESI (m/z) Calcd for ($C_{24}H_{27}NO_2SNa$) ([M+Na]$^+$): 416.1655; found: 416.1652.

10.9 Relationship of radical clock cyclization on the concentration of Ni-catalyst.

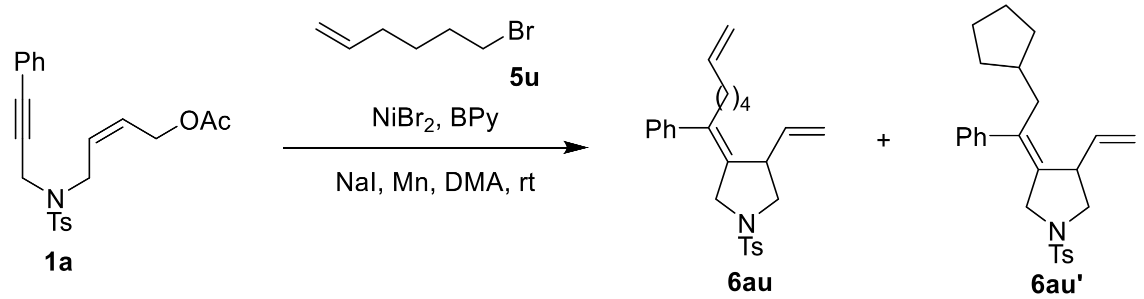

An oven-dried sealed tube equipped with a PTFE-coated stir bar was charged with $NiBr_2$ (5 mol%, 10 mol%, 15 mol%, 20 mol% or 25 mol%), BPy (5 mol%, 10 mol%, 15 mol%, 20 mol% or 25 mol%), NaI (0.05mmol, 7.5 mg), enyne **1a** (0.1 mmol, 39.7 mg), alkyl bromide **5u** (0.12 mmol, 19.6 mg), Mn powder (0.2 mmol, 11.0 mg) and anhydrous DMA (2 mL). The sealed tube was sealed and removed from the glovebox. The reaction mixture was stirred at room temperature until the reaction was completed (monitored by TLC). Then the reaction was quenched by the addition of H_2O (10 mL). The organic layer was separated and the aqueous layer was extracted with EtOAc (20 mL × 3). The combined organic layers were washed with brine, dried over Na_2SO_4, filtered and concentrated. The residue was used for ^1H NMR analysis to determine the yields of products **6au** and **6au'**

(*Z*)-3-(1-phenylhept-6-en-1-ylidene)-1-tosyl-4-vinylpyrrolidine (6au)

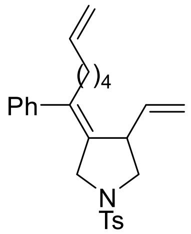

Chemical Formula: $C_{26}H_{31}NO_2S$
Exact Mass: 421.2075

^1H NMR (600 MHz, $CDCl_3$): δ 7.63–7.58 (m, 2H), 7.33–7.28 (m, 4H), 7.26–7.23 (m, 1H), 7.03–6.97 (m, 2H), 5.77–5.65 (m, 2H), 5.11 (dt, *J* = 17.1, 1.3 Hz, 1H), 5.04 (dt, *J* = 10.0, 1.2 Hz, 1H), 4.93–4.83 (m, 2H), 3.78–3.72 (m, 1H), 3.50–3.44 (m, 2H), 3.32–3.26 (m, 2H), 2.43 (s, 3H), 2.30–2.24 (m, 2H), 1.95–1.87 (m, 2H), 1.25–1.22 (m, 2H), 1.20–1.13 (m, 2H);

^{13}C NMR (151 MHz, $CDCl_3$): δ 143.5, 141.4, 138.7, 137.9, 137.6, 132.7, 132.5, 129.6, 128.4, 127.9, 127.5, 127.0, 115.2, 114.3, 53.7, 50.9, 45.0, 34.1, 33.4, 28.8, 27.2, 21.5;

HRMS: (ESI) calcd for $C_{26}H_{32}NO_2S$ ($[M+H]^+$): 422.2148; found: 422.2145.

(*Z*)-3-(2-cyclopentyl-1-phenylethylidene)-1-tosyl-4-vinylpyrrolidine (6au')

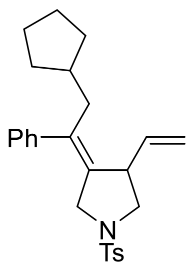

Chemical Formula: $C_{26}H_{31}NO_2S$
Exact Mass: 421.2075

^{1}H NMR (400 MHz, $CDCl_3$): δ 7.60 (d, *J* = 8.4 Hz, 2H), 7.35–7.27 (m, 5H), 7.01 (dt, *J* = 6.8, 1.6 Hz, 2H), 5.85–5.65 (m, 1H), 5.09 (dt, *J* = 16.8, 1.2 Hz, 1H), 5.04 (d, *J* = 10.4 Hz, 1H), 3.74 (dd, *J* = 14.4, 1.2 Hz, 1H), 3.46 (dd, *J* = 14.0, 8.8 Hz, 2H), 3.38–3.22 (m, 2H), 2.43 (s, 3H), 2.30 (d, *J* = 7.6 Hz, 2H), 1.54–1.44 (m, 4H), 1.41–1.29 (m, 3H), 1.09–0.90 (m, 2H);

^{13}C NMR (100 MHz, $CDCl_3$): δ 143.5, 141.6, 138.0, 137.5, 132.7, 130.0, 128.3, 127.8, 127.5, 126.9, 115.3, 53.7, 50.8, 45.0, 39.9, 38.0, 32.3, 32.1, 25.0, 24.9, 21.5;

IR (neat): 3362, 2947, 1738, 1598, 1348, 1161, 1094, 916, 815, 663, 550 cm^{-1};

HRMS-ESI (m/z) Calcd for ($C_{26}H_{31}NO_2SNa$) ($[M+Na]^+$): 444.1968; found: 444.1963.

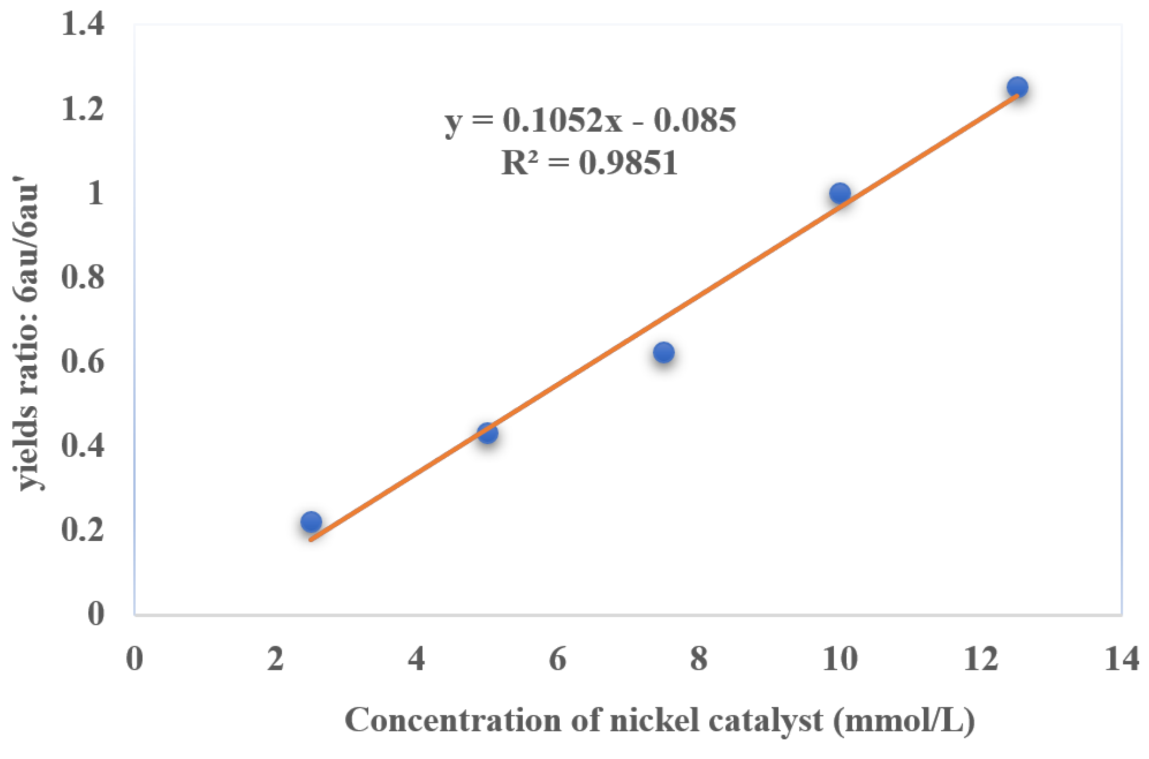

Supplementary Figure 138. The liner relationship between 6au/6au' and Ni-catalyst concentration

11. X-Ray Crystallographic Data

11.1 X-Ray Crystallographic Analysis of 3aj

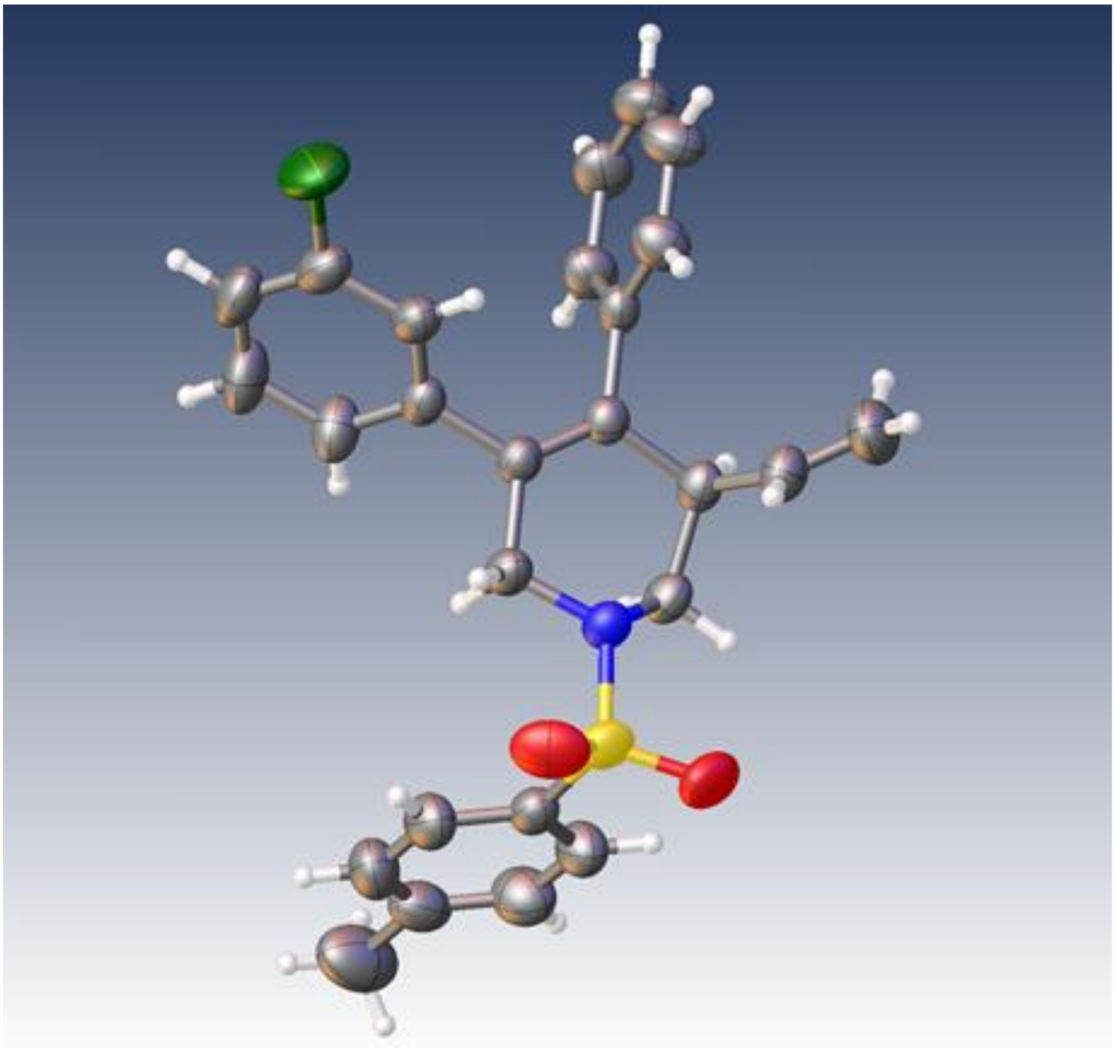

Supplementary Figure 139. ORTEP diagram of compound 3aj with 50% of thermal ellipsoid probability

CCDC 22837839 contains the supplementary crystallographic data for this paper. These data can be obtained free of charge from The Cambridge Crystallographic Data Centre via https://www.ccdc.cam.ac.uk/structures/.

Crystal structure determination of **3aj**

Crystal Data for $C_{26}H_{24}ClNO_2S$, M_r = 449.97, orthorhombic, $P2_12_12_1$ (No. 19), a = 8.0994(2) Å, b = 13.7028(3) Å, c = 21.5431(4) Å, $a = b = g = 90^{\circ}$, V = 2390.95(9) Å^3, T = 299.33(10) K, Z = 4, Z' = 1, m(Cu K_a) = 2.400, 18517 reflections measured, 4706 unique (R_{int} = 0.0300) which were used in all calculations. The final wR_2 was 0.1264 (all data) and R_1 was 0.0416 (I > 2(I)).

Supplementary Table 4: Crystal data and structure refinement for 3aj.

Empirical formula	$C_{26}H_{24}ClNO_2S$
Formula weight	449.97
Temperature/K	299.33(10)
Crystal system	orthorhombic
Space group	$P2_12_12_1$
a/Å	8.0994(2)
b/Å	13.7028(3)
c/Å	21.5431(4)
α/°	90
β/°	90
γ/°	90
Volume/Å^3	2390.95(9)
Z	4
ρ_{calc}g/cm^3	1.250
μ/mm^{-1}	2.400
F(000)	944.0
Radiation	Cu Kα (λ = 1.54184)
2Θ range for data collection/°	7.646 to 152.568
Index ranges	$-9 \le h \le 7$, $-15 \le k \le 16$, $-26 \le l \le 26$
Reflections collected	18517
Independent reflections	4706 [R_{int} = 0.0300, R_{sigma} = 0.0219]
Data/restraints/parameters	4706/0/290
Goodness-of-fit on F^2	1.090
Final R indexes [I>=2σ (I)]	R_1 = 0.0416, wR_2 = 0.1246
Final R indexes [all data]	R_1 = 0.0433, wR_2 = 0.1264
Largest diff. peak/hole / e Å^{-3}	0.32/-0.40
Flack parameter	-0.002(9)

Supplementary Table 5: **Fractional Atomic Coordinates (×10^4) and Equivalent Isotropic Displacement Parameters (Å^2×10^3) for 3aj.** ***U_{eq}*** **is defined as 1/3 of the trace of the orthogonalised** ***U_{ij}*****.**

Atom	x	y	z	U_{eq}
S	2041.5(9)	5399.9(6)	5139.3(3)	57.4(2)
Cl13	3399.1(18)	1091.1(9)	7989.0(6)	102.6(4)
O22	2581(4)	5515(2)	4506.8(10)	79.9(8)
O21	516(3)	4925(2)	5276.0(15)	81.2(8)
N6	3487(3)	4785.3(18)	5495.2(10)	47.4(5)
C2	4506(3)	3914(2)	6418.3(12)	42.6(5)
C3	5901(3)	3715.6(19)	6114.2(11)	42.0(5)
C4	6305(3)	4181(2)	5488.0(12)	46.4(6)
C14	7222(3)	3076(2)	6376.8(12)	44.5(5)
C7	4131(3)	3569(2)	7060.6(12)	46.8(6)
C5	5215(3)	5073(2)	5382.5(14)	49.9(6)
C12	4016(3)	2587(2)	7207.7(13)	54.6(7)
C1	3165(3)	4551(2)	6147.3(13)	50.9(6)
C25	1990(4)	6568(2)	5479.3(13)	52.5(6)
C30	3001(4)	7306(3)	5260.2(16)	64.9(8)
C15	7320(4)	2098(2)	6223.2(16)	60.6(7)
C19	8416(3)	3467(2)	6766.5(15)	57.1(7)
C11	3632(4)	2319(3)	7813.7(15)	63.7(8)
C23	6186(4)	3472(3)	4963.2(13)	59.0(7)
C26	980(4)	6735(3)	5984.7(17)	68.2(9)
C18	9664(4)	2892(3)	7003.0(16)	67.3(9)
C8	3839(4)	4247(3)	7528.1(15)	66.0(8)
C17	9721(4)	1902(3)	6855.8(19)	70.8(9)
C10	3394(4)	2996(3)	8275.4(15)	72.9(10)
C29	2992(5)	8198(3)	5554.5(19)	76.2(10)
C9	3490(5)	3958(4)	8126.3(16)	80.2(11)
C28	2022(5)	8378(3)	6063.4(18)	71.9(9)
C16	8569(5)	1523(3)	6463(2)	72.3(9)
C27	991(5)	7629(3)	6267.2(18)	78.5(11)
C24	7460(7)	3173(4)	4634(2)	90.6(14)
C6	2042(8)	9369(4)	6381(3)	113.3(19)

Supplementary Table 6: **Anisotropic Displacement Parameters (×10⁴) 3aj. The anisotropic displacement factor exponent takes the form:** ***$-2p^2[h^2a^{*2} \times U_{11} + ... + 2hka^* \times b^* \times U_{12}]$***

Atom	U_{11}	U_{22}	U_{33}	U_{23}	U_{13}	U_{12}
S	58.4(4)	65.7(4)	48.0(4)	4.2(3)	-15.9(3)	6.8(3)
Cl13	129.9(9)	85.0(7)	93.0(7)	43.3(6)	18.3(7)	25.3(7)
O22	111(2)	90.3(18)	38.8(10)	-0.2(10)	-18.3(12)	23.4(16)
O21	53.6(12)	84.0(17)	106(2)	5.8(16)	-29.2(13)	-1.5(11)
N6	44.0(11)	57.4(13)	40.9(11)	4.4(9)	-4.1(9)	3.4(9)
C2	40.7(11)	51.6(14)	35.5(12)	0.4(10)	-1.0(10)	0.2(10)
C3	40.3(11)	48.8(13)	36.9(12)	-2.4(10)	-1.3(9)	-0.8(10)
C4	41.4(12)	59.1(15)	38.8(12)	2.1(11)	1.5(10)	-3.0(11)
C14	37.5(11)	61.2(15)	34.9(11)	-1.2(10)	2.4(9)	3.8(10)
C7	38.8(11)	64.6(16)	37.1(12)	3.4(11)	0.7(10)	3.8(11)
C5	52.3(14)	55.2(15)	42.2(13)	7.7(12)	2.6(11)	-3.3(11)
C12	52.2(14)	65.9(17)	45.8(15)	9.7(12)	1.9(11)	13.5(13)
C1	43.6(12)	66.0(16)	43.0(13)	8.9(12)	1.8(10)	9.8(12)
C25	48.0(13)	65.2(16)	44.2(13)	8.9(12)	0.0(12)	9.2(12)
C30	63.7(17)	65.8(19)	65.0(19)	9.7(15)	16.6(16)	8.4(14)
C15	53.3(15)	62.0(17)	66.5(18)	-8.6(15)	-6.1(14)	4.6(13)
C19	50.2(14)	68.3(18)	52.9(15)	-7.4(13)	-9.2(12)	6.8(13)
C11	52.7(14)	83(2)	55.5(17)	22.3(15)	1.2(13)	11.3(15)
C23	67.5(17)	68.5(18)	40.8(14)	-0.5(12)	4.5(13)	4.3(14)
C26	61.1(17)	80(2)	63(2)	11.3(17)	15.7(15)	12.3(16)
C18	50.6(15)	93(2)	58.5(18)	-3.8(18)	-14.0(14)	9.5(15)
C8	78(2)	74(2)	46.3(16)	-6.9(15)	11.8(15)	-8.4(16)
C17	57.0(16)	86(2)	69(2)	13.1(18)	-2.8(16)	20.8(16)
C10	67.5(19)	111(3)	40.6(15)	14.0(17)	6.3(14)	8.2(19)
C29	74(2)	68(2)	87(3)	9.9(18)	7(2)	3.7(17)
C9	93(3)	106(3)	41.4(16)	-11.5(17)	13.8(17)	-4(2)
C28	80(2)	66(2)	69(2)	-3.6(16)	-11.7(18)	22.5(18)
C16	72.2(19)	60.7(19)	84(2)	2.2(17)	-2.3(18)	16.6(16)
C27	81(2)	95(3)	59(2)	-2.0(19)	13.4(17)	25(2)
C24	107(3)	105(3)	60(2)	-10(2)	13(2)	37(3)
C6	146(4)	90(3)	104(4)	-32(3)	-31(4)	37(3)

Supplementary Table 7: Bond Lengths in Å for 3aj.

Atom	Atom	Length/Å
S	O22	1.439(3)
S	O21	1.427(3)
S	N6	1.633(2)
S	C25	1.760(3)
Cl13	C11	1.735(4)
N6	C5	1.475(3)
N6	C1	1.464(3)
C2	C3	1.334(4)
C2	C7	1.494(4)
C2	C1	1.510(4)
C3	C4	1.528(3)
C3	C14	1.495(3)
C4	C5	1.525(4)
C4	C23	1.494(4)
C14	C15	1.382(4)
C14	C19	1.388(4)
C7	C12	1.386(5)
C7	C8	1.390(4)
C12	C11	1.391(4)
C25	C30	1.385(5)
C25	C26	1.381(4)
C30	C29	1.377(5)
C15	C16	1.383(5)
C19	C18	1.380(4)
C11	C10	1.373(5)
C23	C24	1.317(5)
C26	C27	1.368(6)
C18	C17	1.393(6)
C8	C9	1.378(5)
C17	C16	1.363(6)
C10	C9	1.360(6)
C29	C28	1.371(6)
C28	C27	1.395(6)
C28	C6	1.520(6)

Supplementary Table 8: Bond Angles in Å for 3aj.

Atom	Atom	Atom	Angle/°
O22	S	N6	106.45(14)
O22	S	C25	107.56(16)
O21	S	O22	120.52(19)
O21	S	N6	106.78(14)
O21	S	C25	107.93(17)
N6	S	C25	106.87(13)
C5	N6	S	117.71(19)
C1	N6	S	115.86(18)
C1	N6	C5	112.7(2)
C3	C2	C7	124.2(2)
C3	C2	C1	122.5(2)
C7	C2	C1	113.3(2)
C2	C3	C4	122.0(2)
C2	C3	C14	122.7(2)
C14	C3	C4	115.2(2)
C5	C4	C3	110.0(2)
C23	C4	C3	112.5(2)
C23	C4	C5	111.8(2)
C15	C14	C3	121.3(2)
C15	C14	C19	118.6(3)
C19	C14	C3	120.1(3)
C12	C7	C2	122.2(3)
C12	C7	C8	118.2(3)
C8	C7	C2	119.6(3)
N6	C5	C4	108.1(2)
C7	C12	C11	119.0(3)
N6	C1	C2	111.7(2)
C30	C25	S	120.6(2)
C26	C25	S	119.5(3)
C26	C25	C30	119.8(3)
C29	C30	C25	119.3(3)
C14	C15	C16	120.3(3)
C18	C19	C14	120.9(3)
C12	C11	Cl13	118.9(3)
C10	C11	Cl13	118.8(3)
C10	C11	C12	122.2(3)
C24	C23	C4	124.0(4)
C27	C26	C25	119.7(4)
C19	C18	C17	119.8(3)
C9	C8	C7	121.4(4)
C16	C17	C18	119.3(3)
C9	C10	C11	118.4(3)
C28	C29	C30	122.1(4)
C10	C9	C8	120.8(4)
C29	C28	C27	117.5(4)
C29	C28	C6	120.9(4)
C27	C28	C6	121.5(4)
C17	C16	C15	121.1(3)
C26	C27	C28	121.5(3)

Supplementary Table 9: Hydrogen Fractional Atomic Coordinates (×10^4) and Equivalent Isotropic Displacement Parameters (Å^2×10^3) for 3aj. U_{eq} is defined as 1/3 of the trace of the orthogonalised U_{ij}.

Atom	x	y	z	U_{eq}
H4	7451.15	4408.93	5506.84	56
H5A	5530.31	5593.48	5664.11	60
H5B	5342.1	5307.4	4960.22	60
H12	4194.38	2113.36	6905.72	66
H1A	2113.2	4216.13	6180.29	61
H1B	3091.25	5150.83	6384.51	61
H30	3677.95	7201.17	4918.05	78
H15	6541.42	1826.1	5957.17	73
H19	8373.72	4125.65	6869.39	69
H23	5149.45	3227.49	4862.64	71
H26	296.13	6241.67	6132.38	82
H18	10464.48	3163.34	7259.79	81
H8	3880.96	4909.28	7434.97	79
H17	10537.23	1504.62	7023.87	85
H10	3172.12	2800.03	8680.26	87
H29	3665.39	8694.01	5403.4	91
H9	3317.09	4426.71	8431.68	96
H16	8623.95	867.02	6354.43	87
H27	294.26	7739.08	6603.04	94
H6A	3112.73	9482.55	6557.83	170
H6B	1223.62	9381.44	6702.62	170
H6C	1803.69	9868.78	6081.42	170
H24A	7020(90)	2600(50)	4360(30)	150(20)
H24B	8850(80)	3280(50)	4830(30)	130(20)

10.2 X-Ray Crystallographic Analysis of 7aa.

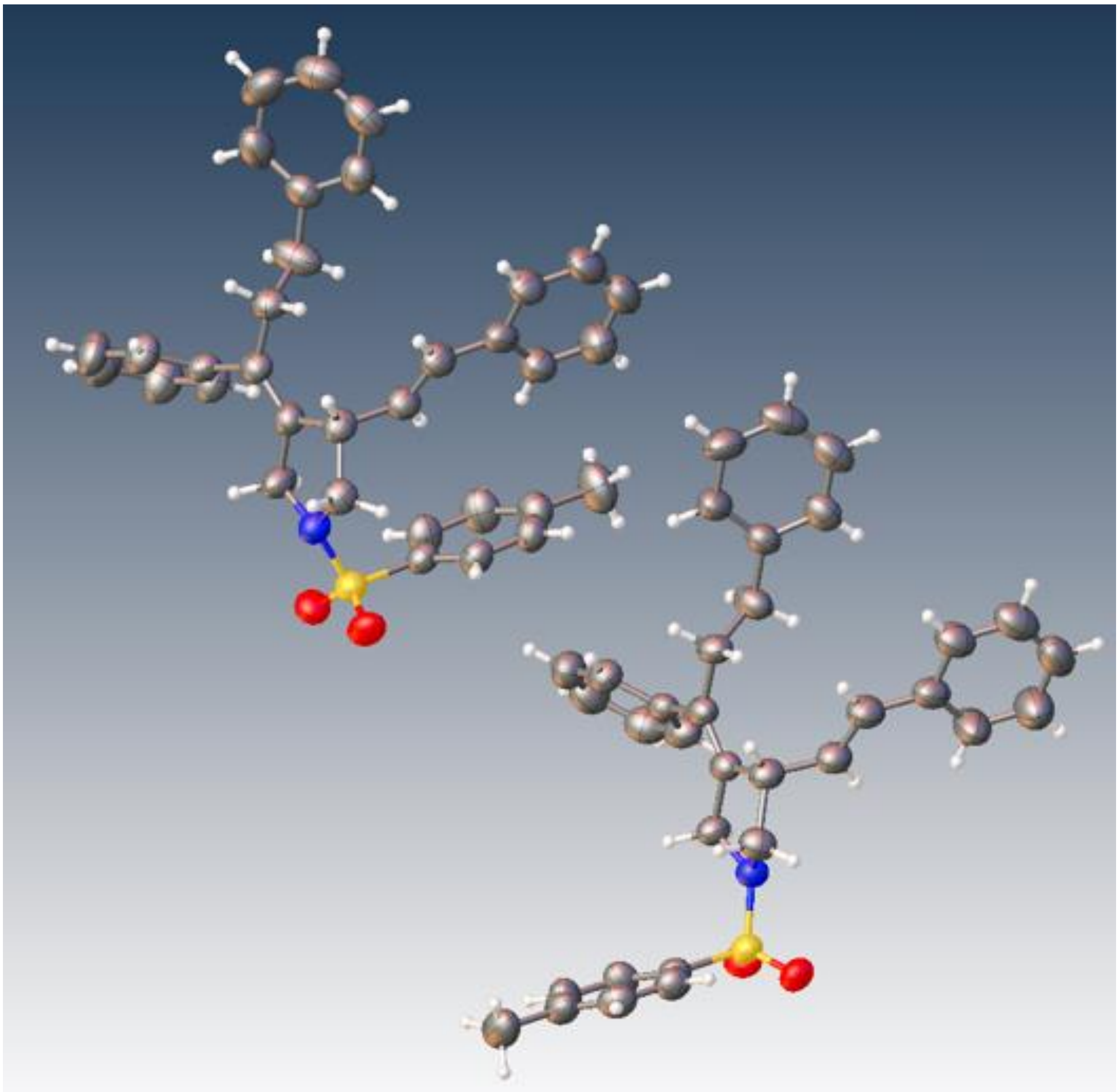

Supplementary Figure 140. ORTEP diagram of compound 7aa with 50% of thermal ellipsoid probability

CCDC 2271665 contains the supplementary crystallographic data for this paper. These data can be obtained free of charge from The Cambridge Crystallographic Data Centre via https://www.ccdc.cam.ac.uk/structures/.

Crystal structure determination of **7aa**

Crystal Data for $C_{68}H_{66}N_2O_4S_2$, M_r = 1039.34, monoclinic, $P2_1$ (No. 4), a = 11.68544(11) Å, b = 10.33108(11) Å, c = 24.4395(2) Å, b = 101.9866(9)°, a = g = 90°, V = 2886.09(5) Å^3, T = 297.63(10) K, Z = 2, Z' = 1, m(Cu K$_a$) = 1.224, 55452 reflections measured, 10768 unique (R_{int} = 0.0264) which were used in all calculations. The final wR_2 was 0.0927 (all data) and R_1 was 0.0348 (I > 2(I)).

Supplementary Table 10: Crystal data and structure refinement for 7aa.

Formula	$C_{68}H_{66}N_2O_4S_2$
$D_{calc.}$/ g cm^{-3}	1.196
m/mm^{-1}	1.224
Formula Weight	1039.34
Colour	clear light colourless
Shape	block
Size/mm^3	0.32×0.22×0.04
T/K	297.63(10)
Crystal System	monoclinic
Flack Parameter	-0.014(5)
Hooft Parameter	-0.022(3)
Space Group	$P2_1$
a/Å	11.68544(11)
b/Å	10.33108(11)
c/Å	24.4395(2)
a/°	90
b/°	101.9866(9)
g/°	90
V/Å^3	2886.09(5)
Z	2
Z'	1
Wavelength/Å	1.54184
Radiation type	Cu K_a
Q_{min}/°	1.848
Q_{max}/°	75.788
Measured Refl.	55452
Independent Refl.	10768
Reflections with I > 2(I)	9521

R_{int}	0.0264
Parameters	688
Restraints	1
Largest Peak	0.165
Deepest Hole	-0.164
GooF	1.034
wR_2 (all data)	0.0927
wR_2	0.0895
R_1 (all data)	0.0402
R_1	0.0348

Supplementary Table 11: Fractional Atomic Coordinates (×10^4) and Equivalent Isotropic Displacement Parameters (Å^2×10^3) for 7aa. U_{eq} is defined as 1/3 of the trace of the orthogonalised U_{ij}.

Atom	x	y	z	U_{eq}
S7	4446.4(5)	5935.0(6)	9756.6(2)	51.70(15)
S45	5341.3(5)	4027.0(7)	5280.0(3)	53.39(16)
O9	3801.3(15)	4766.6(19)	9767.7(8)	61.9(5)
O8	4822.5(17)	6669(2)	10258.0(7)	67.4(5)
O47	5218.7(18)	5221(2)	5555.7(8)	70.5(5)
O46	4327.1(15)	3288(2)	5035.3(8)	67.6(5)
N24	5624.7(16)	5547(2)	9542.1(8)	50.5(5)
N68	6005.5(18)	4344(2)	4780.0(8)	53.8(5)
C39	6261(2)	3036(3)	5767.2(10)	51.0(6)
C1	3615(2)	6979(3)	9256.3(10)	50.5(6)
C44	7062(2)	3591(3)	6207.2(11)	56.9(6)
C58	6706(2)	2305(3)	3319.6(10)	51.2(6)
C20	5563(2)	4609(3)	9079.1(10)	52.9(6)
C19	7178(2)	3961(3)	8578.6(10)	51.2(5)
C43	7798(2)	2799(3)	6571.3(11)	63.0(7)
C21	6778(2)	4681(3)	8955.9(10)	50.7(6)
C57	7547(2)	3040(3)	3751.6(10)	52.4(6)
C33	6463(2)	2939(3)	8243.5(10)	51.1(6)
C66	7979(2)	4401(3)	4639.8(11)	55.4(6)
C17	9174(2)	2951(3)	8616.8(14)	73.4(8)
C6	2738(2)	6485(3)	8838.6(12)	60.4(7)
C65	7230(2)	3549(3)	4199.0(10)	51.7(6)
C4	2338(3)	8645(3)	8461.8(12)	61.8(7)
C56	8770(2)	3147(3)	3643.2(12)	58.9(6)
C11	10374(2)	3069(3)	8482.7(12)	59.5(7)

Atom	x	y	z	U_{eq}
C71	10807(2)	3193(3)	5648.4(12)	62.9(7)
C40	6223(3)	1710(3)	5703.0(12)	65.5(7)
C22	7463(2)	5736(3)	9321.0(11)	57.9(6)
C2	3851(2)	8293(3)	9277.4(12)	58.6(7)
C25	8286(2)	5175(3)	9815.5(11)	60.9(7)
C70	9950(2)	3840(3)	5209.5(12)	64.6(7)
C67	7064(2)	5168(3)	4883.6(12)	59.2(6)
C18	8399(2)	4142(3)	8466.1(11)	59.0(6)
C64	6036(2)	3396(3)	4334.1(11)	62.4(7)
C23	6488(2)	6546(3)	9489.4(12)	61.0(7)
C34	5945(2)	1945(3)	8488.4(12)	63.4(7)
C27	10290(2)	4782(3)	10386.9(13)	69.7(8)
C38	6320(2)	2921(3)	7662.0(11)	62.6(7)
C69	8801(2)	3655(3)	5080.2(11)	57.9(6)
C5	2116(3)	7326(3)	8444.7(13)	67.6(8)
C37	5692(3)	1964(4)	7343.5(13)	75.2(8)
C14	12598(3)	3176(4)	8239.6(18)	82.4(10)
C49	10560(2)	1889(3)	3536.8(13)	63.1(7)
C3	3223(2)	9109(3)	8885.5(12)	63.4(7)
C35	5327(3)	982(3)	8162.9(15)	76.5(8)
C15	12464(3)	2807(3)	8751.8(18)	80.6(10)
C59	6404(3)	2754(3)	2778.0(12)	69.5(8)
C36	5204(3)	995(4)	7594.6(15)	80.1(9)
C32	11478(2)	4938(3)	10396.9(15)	76.6(9)
C10	1639(3)	9556(4)	8035.6(14)	85.5(10)
C63	6199(3)	1169(3)	3444.0(12)	70.1(8)
C16	11364(3)	2757(3)	8876.1(14)	69.9(8)
C26	9434(2)	5301(3)	9915.5(13)	70.1(8)

Atom	x	y	z	U_{eq}
C12	10533(3)	3465(4)	7966.0(13)	75.8(9)
C72	11996(2)	3420(4)	5686.7(14)	77.0(9)
C54	11555(3)	2226(3)	3918.5(13)	68.1(8)
C76	10491(3)	2378(4)	6041.7(14)	78.4(9)
C41	6977(3)	942(3)	6076.8(14)	75.6(8)
C62	5434(3)	495(4)	3033.2(14)	83.3(10)
C42	7769(3)	1469(3)	6521.1(13)	66.7(8)
C60	5639(3)	2098(4)	2371.0(13)	83.0(10)
C75	11316(3)	1820(4)	6459.5(15)	89.7(10)
C31	12309(3)	4488(4)	10835.8(18)	89.3(11)
C13	11643(3)	3532(4)	7841.6(16)	86.4(10)
C61	5153(3)	958(4)	2498.4(12)	78.6(9)
C53	12618(3)	2312(3)	3778.2(18)	80.3(10)
C55	9389(3)	1841(3)	3704.9(18)	87.9(11)
C52	12722(3)	2086(4)	3254(2)	91.3(11)
C50	10679(3)	1645(4)	3000.0(15)	87.9(10)
C73	12818(3)	2858(5)	6100.5(16)	90.7(11)
C51	11774(4)	1744(4)	2855.6(17)	99.3(13)
C30	11988(3)	3909(4)	11273.6(16)	93.3(11)
C28	9987(3)	4160(5)	10830.2(15)	101.9(13)
C74	12484(3)	2081(4)	6489.9(16)	89.4(11)
C48	8541(3)	615(4)	6948.4(17)	100.5(13)
C29	10823(3)	3723(6)	11273.2(17)	113.9(15)

Supplementary Table 12: Anisotropic Displacement Parameters (×10⁴) 7aa. The anisotropic displacement factor exponent takes the form: $-2p^2[h^2a^{*2} \times U_{11} + ... +2hka^* \times b^* \times U_{12}]$.

Atom	U_{11}	U_{22}	U_{33}	U_{23}	U_{13}	U_{12}
S7	52.0(3)	60.6(4)	45.2(3)	-0.9(3)	16.4(2)	6.4(3)
S45	51.6(3)	61.3(4)	48.8(3)	-0.5(3)	13.9(2)	5.9(3)
O9	58.5(10)	63.7(12)	69.2(11)	10.3(9)	26.2(9)	2.9(9)
O8	70.9(12)	86.5(14)	46.1(9)	-9.8(9)	14.9(8)	10.7(10)
O47	85.4(13)	66.4(13)	63.2(11)	-3.9(10)	23.4(10)	24.2(10)
O46	48.4(10)	90.8(15)	63.9(11)	0.6(10)	12.6(8)	-4.0(9)
N24	45.9(11)	55.5(13)	51.5(11)	-3.9(9)	13.5(9)	3.1(8)
N68	54.3(12)	59.4(14)	48.6(11)	-1.6(9)	12.7(9)	-2.4(9)
C39	54.7(14)	54.6(16)	45.9(13)	-1.7(11)	15.4(11)	-3.9(11)
C1	50.0(13)	53.0(16)	50.0(13)	-6.2(11)	14.1(11)	5.2(10)
C44	70.1(16)	52.5(16)	48.9(14)	-4.4(11)	14.2(12)	-3.6(12)
C58	52.7(13)	55.1(15)	49.7(13)	-1.7(11)	19.7(11)	1.0(11)
C20	50.9(13)	60.5(16)	50.8(14)	-6.3(11)	18.6(11)	-2.4(11)
C19	49.4(12)	57.6(15)	48.6(13)	8.2(12)	15.2(10)	7.5(11)
C43	63.5(16)	70(2)	52.9(15)	-1.6(14)	6.4(12)	-4.2(14)
C21	48.3(13)	56.8(15)	49.2(13)	7.2(11)	15.4(10)	4.1(11)
C57	50.7(13)	55.2(16)	53.0(13)	6.3(11)	14.5(11)	-0.8(11)
C33	45.9(12)	57.4(16)	52.6(13)	5.0(12)	16.3(10)	11.2(11)
C66	54.0(14)	59.8(17)	53.5(14)	-1.0(11)	13.4(11)	-13.2(11)
C17	54.0(16)	83(2)	86(2)	17.8(18)	21.3(14)	15.0(14)
C6	60.8(16)	52.0(16)	65.2(16)	-7.7(13)	5.9(12)	3.0(12)
C65	49.5(13)	57.4(15)	48.2(13)	2.2(11)	10.3(10)	-3.3(10)
C4	71.0(17)	61(2)	56.4(16)	0.6(13)	20.6(13)	14.8(13)
C56	54.1(14)	62.4(17)	64.0(15)	1.9(13)	21.1(12)	-2.9(11)
C11	46.1(13)	64.7(18)	67.1(16)	-6.4(14)	10.2(12)	6.1(11)

Atom	U_{11}	U_{22}	U_{33}	U_{23}	U_{13}	U_{12}
C71	50.5(14)	78(2)	60.0(15)	-15.8(14)	11.0(12)	-0.6(13)
C40	71.0(18)	54.5(18)	66.5(17)	-5.6(14)	4.1(14)	-6.9(13)
C22	51.9(13)	65.6(18)	59.8(15)	-1.6(13)	20.4(11)	-4.0(12)
C2	60.9(15)	55.9(18)	60.2(15)	-9.6(13)	15.4(12)	0.0(12)
C25	51.3(14)	72.8(18)	60.7(15)	-6.7(14)	16.5(12)	-4.9(12)
C70	54.9(14)	75(2)	64.1(16)	-7.4(14)	11.9(12)	-8.9(13)
C67	64.5(16)	52.6(16)	61.3(15)	-3.0(13)	14.6(12)	-6.6(12)
C18	52.8(14)	68.0(17)	61.0(15)	2.4(13)	23.4(11)	4.7(12)
C64	54.9(14)	81(2)	54.1(14)	-14.1(14)	18.6(12)	-11.9(13)
C23	53.6(15)	59.5(17)	71.6(17)	-4.9(14)	16.9(12)	-1.7(12)
C34	66.4(16)	64.1(18)	65.3(16)	8.7(14)	26.6(13)	5.8(13)
C27	50.4(15)	88(2)	71.6(18)	-15.2(16)	15.9(13)	1.4(14)
C38	62.3(16)	73.0(19)	55.0(15)	4.8(14)	18.3(12)	2.8(13)
C69	51.7(14)	65.2(17)	57.2(14)	-4.1(12)	12.6(11)	-6.3(11)
C5	65.3(17)	71(2)	60.4(17)	-10.2(14)	-1.6(13)	10.6(14)
C37	68.6(18)	97(3)	58.4(16)	-7.3(17)	8.2(14)	1.3(16)
C14	46.1(16)	82(2)	120(3)	-29(2)	19.3(18)	-1.2(14)
C49	53.8(15)	56.3(17)	78.6(19)	0.1(15)	12.6(13)	8.5(12)
C3	72.3(17)	51.2(16)	70.7(17)	-3.6(14)	23.6(14)	4.7(13)
C35	68.8(17)	68(2)	97(2)	5.0(18)	26.7(16)	-4.0(15)
C15	50.4(16)	74(2)	110(3)	-13(2)	-2.5(17)	5.9(14)
C59	79.6(19)	71(2)	58.5(16)	6.7(15)	15.3(14)	-12.1(15)
C36	63.3(17)	85(2)	89(2)	-13(2)	7.3(16)	-3.1(17)
C32	53.4(16)	88(2)	88(2)	-10.0(18)	14.1(15)	-2.2(15)
C10	103(2)	85(2)	69(2)	10.2(17)	19.1(18)	32(2)
C63	83.7(19)	68(2)	55.9(16)	2.8(14)	9.4(14)	-13.0(15)
C16	62.4(17)	62.9(19)	81(2)	-1.8(16)	6.5(14)	9.9(13)
C26	52.5(15)	89(2)	71.3(18)	-6.5(16)	18.4(13)	-4.5(14)

Atom	U_{11}	U_{22}	U_{33}	U_{23}	U_{13}	U_{12}
C12	50.0(15)	113(3)	64.3(18)	-6.6(17)	10.9(13)	6.6(15)
C72	51.6(16)	99(2)	79(2)	-12.1(18)	10.7(14)	-10.9(15)
C54	73.2(18)	59.4(18)	67.3(17)	1.7(14)	4.6(14)	7.7(14)
C76	55.4(16)	103(3)	76(2)	-0.7(18)	11.6(15)	5.2(16)
C41	86(2)	49.5(17)	86(2)	3.1(16)	6.4(16)	-2.2(16)
C62	98(2)	78(2)	73(2)	-7.4(17)	13.9(17)	-27.2(18)
C42	65.9(18)	66(2)	68.2(18)	9.6(15)	12.6(14)	2.5(13)
C60	92(2)	105(3)	50.0(16)	5.9(17)	8.8(15)	-11(2)
C75	82(2)	107(3)	78(2)	8(2)	10.4(17)	13(2)
C31	57.1(18)	94(3)	113(3)	-22(2)	8.4(19)	1.4(16)
C13	67.4(19)	116(3)	83(2)	-20(2)	33.0(17)	-2.7(18)
C61	82(2)	95(2)	58.9(17)	-19.2(18)	15.2(15)	-17.8(19)
C53	58.0(18)	64(2)	112(3)	4.7(19)	0.3(18)	4.1(14)
C55	62.4(18)	70(2)	138(3)	19(2)	36.2(19)	9.5(15)
C52	75(2)	81(3)	125(3)	10(2)	37(2)	16.2(18)
C50	78(2)	92(3)	83(2)	-15(2)	-8.4(18)	13.7(18)
C73	51.8(17)	124(3)	90(2)	-23(2)	1.4(17)	0.5(18)
C51	134(4)	92(3)	82(2)	-5(2)	45(2)	29(3)
C30	73(2)	113(3)	84(2)	-12(2)	-5.4(18)	24(2)
C28	55.5(17)	171(4)	81(2)	24(3)	18.0(16)	14(2)
C74	67(2)	111(3)	81(2)	-13(2)	-4.5(17)	17.9(19)
C48	98(3)	87(3)	106(3)	25(2)	-6(2)	11(2)
C29	84(3)	172(5)	87(3)	28(3)	19(2)	20(3)

Supplementary Table 13: Bond Lengths in Å for **7aa**.

Atom	Atom	Length/Å	Atom	Atom	Length/Å
S7	O9	1.426(2)	C57	C56	1.511(3)
S7	O8	1.4304(19)	C33	C34	1.389(4)
S7	N24	1.622(2)	C33	C38	1.396(3)
S7	C1	1.763(3)	C66	C65	1.520(3)
S45	O47	1.427(2)	C66	C67	1.546(4)
S45	O46	1.432(2)	C66	C69	1.498(4)
S45	N68	1.612(2)	C17	C11	1.510(4)
S45	C39	1.758(3)	C17	C18	1.527(4)
N24	C20	1.480(3)	C6	C5	1.386(4)
N24	C23	1.467(3)	C65	C64	1.507(3)
N68	C67	1.479(3)	C4	C5	1.385(5)
N68	C64	1.471(3)	C4	C3	1.389(4)
C39	C44	1.393(4)	C4	C10	1.511(4)
C39	C40	1.379(4)	C56	C55	1.522(4)
C1	C6	1.385(4)	C11	C16	1.380(4)
C1	C2	1.383(4)	C11	C12	1.376(4)
C44	C43	1.372(4)	C71	C70	1.467(4)
C58	C57	1.492(4)	C71	C72	1.393(4)
C58	C59	1.378(4)	C71	C76	1.384(4)
C58	C63	1.376(4)	C40	C41	1.380(4)
C20	C21	1.513(3)	C22	C25	1.496(4)
C19	C21	1.342(4)	C22	C23	1.537(4)
C19	C33	1.483(4)	C2	C3	1.369(4)
C19	C18	1.519(3)	C25	C26	1.319(4)
C43	C42	1.379(5)	C70	C69	1.328(4)
C21	C22	1.525(4)	C34	C35	1.380(4)
C57	C65	1.333(4)	C27	C32	1.393(4)

Atom	Atom	Length/Å
C27	C26	1.462(4)
C27	C28	1.368(5)
C38	C37	1.372(4)
C37	C36	1.360(5)
C14	C15	1.349(5)
C14	C13	1.370(5)
C49	C54	1.375(4)
C49	C55	1.510(4)
C49	C50	1.371(5)
C35	C36	1.366(4)
C15	C16	1.382(5)
C59	C60	1.371(4)
C32	C31	1.370(5)
C63	C62	1.386(4)
C12	C13	1.393(4)

Atom	Atom	Length/Å
C72	C73	1.371(5)
C54	C53	1.359(5)
C76	C75	1.377(5)
C41	C42	1.384(4)
C62	C61	1.366(4)
C42	C48	1.514(4)
C60	C61	1.371(5)
C75	C74	1.378(5)
C31	C30	1.346(6)
C53	C52	1.332(5)
C52	C51	1.361(6)
C50	C51	1.400(6)
C73	C74	1.363(6)
C30	C29	1.375(5)
C28	C29	1.375(5)

Supplementary Table 14: Bond Angles for 7aa.

Atom	Atom	Atom	Angle/°	Atom	Atom	Atom	Angle/°
O9	S7	O8	120.13(12)	C63	C58	C59	117.6(3)
O9	S7	N24	106.54(11)	N24	C20	C21	102.77(19)
O9	S7	C1	108.30(12)	C21	C19	C33	122.5(2)
O8	S7	N24	106.10(11)	C21	C19	C18	122.0(2)
O8	S7	C1	107.25(12)	C33	C19	C18	115.5(2)
N24	S7	C1	108.02(11)	C44	C43	C42	122.4(3)
O47	S45	O46	120.16(13)	C20	C21	C22	108.6(2)
O47	S45	N68	107.09(12)	C19	C21	C20	125.8(2)
O47	S45	C39	106.90(12)	C19	C21	C22	125.6(2)
O46	S45	N68	106.19(11)	C58	C57	C56	114.9(2)
O46	S45	C39	108.03(12)	C65	C57	C58	121.7(2)
N68	S45	C39	107.99(11)	C65	C57	C56	123.3(2)
C20	N24	S7	119.92(16)	C34	C33	C19	122.3(2)
C23	N24	S7	120.01(17)	C34	C33	C38	117.2(3)
C23	N24	C20	108.57(19)	C38	C33	C19	120.4(2)
C67	N68	S45	120.30(17)	C65	C66	C67	103.1(2)
C64	N68	S45	121.08(18)	C69	C66	C65	113.6(2)
C64	N68	C67	111.2(2)	C69	C66	C67	112.2(2)
C44	C39	S45	120.1(2)	C11	C17	C18	114.5(3)
C40	C39	S45	120.1(2)	C1	C6	C5	118.8(3)
C40	C39	C44	119.8(3)	C57	C65	C66	126.3(2)
C6	C1	S7	120.1(2)	C57	C65	C64	124.5(2)
C2	C1	S7	119.8(2)	C64	C65	C66	109.2(2)
C2	C1	C6	120.0(3)	C5	C4	C3	117.9(3)
C43	C44	C39	119.0(3)	C5	C4	C10	121.4(3)
C59	C58	C57	120.5(2)	C3	C4	C10	120.7(3)
C63	C58	C57	121.9(2)	C57	C56	C55	111.4(2)

Atom	Atom	Atom	Angle/°	Atom	Atom	Atom	Angle/°
C16	C11	C17	120.8(3)	C50	C49	C55	122.1(3)
C12	C11	C17	122.1(2)	C2	C3	C4	121.2(3)
C12	C11	C16	117.1(3)	C36	C35	C34	120.7(3)
C72	C71	C70	119.5(3)	C14	C15	C16	120.5(3)
C76	C71	C70	123.0(3)	C60	C59	C58	121.9(3)
C76	C71	C72	117.5(3)	C37	C36	C35	120.0(3)
C39	C40	C41	119.6(3)	C31	C32	C27	121.1(3)
C21	C22	C23	102.57(19)	C58	C63	C62	120.7(3)
C25	C22	C21	111.5(2)	C11	C16	C15	121.4(3)
C25	C22	C23	112.5(2)	C25	C26	C27	127.8(3)
C3	C2	C1	120.3(3)	C11	C12	C13	121.6(3)
C26	C25	C22	124.7(3)	C73	C72	C71	121.0(3)
C69	C70	C71	127.7(3)	C53	C54	C49	122.2(3)
N68	C67	C66	105.1(2)	C75	C76	C71	121.5(3)
C19	C18	C17	112.9(2)	C40	C41	C42	121.6(3)
N68	C64	C65	105.0(2)	C61	C62	C63	120.5(3)
N24	C23	C22	101.6(2)	C43	C42	C41	117.5(3)
C35	C34	C33	120.5(3)	C43	C42	C48	121.3(3)
C32	C27	C26	119.2(3)	C41	C42	C48	121.1(3)
C28	C27	C32	117.5(3)	C59	C60	C61	119.9(3)
C28	C27	C26	123.3(3)	C76	C75	C74	119.4(4)
C37	C38	C33	121.6(3)	C30	C31	C32	120.3(3)
C70	C69	C66	124.2(3)	C14	C13	C12	119.3(3)
C4	C5	C6	121.9(3)	C62	C61	C60	119.3(3)
C36	C37	C38	119.9(3)	C52	C53	C54	120.2(3)
C15	C14	C13	120.0(3)	C49	C55	C56	112.5(3)
C54	C49	C55	120.6(3)	C53	C52	C51	120.7(3)
C50	C49	C54	117.2(3)	C49	C50	C51	120.4(3)

**Atom**	**Atom**	**Atom**	**Angle/°**	**Atom**	**Atom**	**Atom**	**Angle/°**
C74	C73	C72	120.4(3)	C27	C28	C29	121.3(3)
C52	C51	C50	119.3(3)	C73	C74	C75	120.1(3)
C31	C30	C29	120.1(3)	C30	C29	C28	119.7(4)

Supplementary Table 15: Hydrogen Fractional Atomic Coordinates (×10^4) and Equivalent Isotropic Displacement Parameters (Å^2×10^3) for 7aa. U_{eq} is defined as 1/3 of the trace of the orthogonalised U_{ij}.

Atom	x	y	z	U_{eq}
H44	7096.74	4484.27	6253.4	68
H20A	4973.08	4860.07	8755.63	64
H20B	5391.43	3744.4	9194.18	64
H43	8335.37	3172.56	6862.55	76
H66	8429.05	5003.8	4457.25	67
H17A	9263.74	2784.34	9014.01	88
H17B	8781.13	2209.72	8418.08	88
H6	2569.32	5604.21	8822.9	72
H56A	9217.32	3757.24	3904.82	71
H56B	8731.75	3474.77	3267.98	71
H40	5692.46	1334.31	5409.42	79
H22	7896.22	6262.78	9099.41	69
H2	4439.26	8622.46	9558.89	70
H25	7965.12	4702.55	10070.8	73
H70	10247.22	4451.07	4996.67	77
H67A	7339.86	5315.44	5281.07	71
H67B	6903.04	5997	4697.37	71
H18A	8766.38	4876.07	8680.47	71
H18B	8336.91	4337.95	8072.86	71
H64A	5427.5	3576.74	4007.89	75
H64B	5930.25	2524.49	4462.29	75
H23A	6765.76	6991.58	9841.22	73
H23B	6169.17	7173.47	9202.88	73
H34	6015.42	1927.98	8874.44	76
H38	6658.04	3572.26	7486.21	75

Atom	x	y	z	U_{eq}
H69	8485.45	3022.09	5275.7	69
H5	1533.19	6996.82	8160.77	81
H37	5600.25	1978.46	6956.37	90
H14	13339.37	3190.03	8156.66	99
H3	3392.68	9989.13	8903.44	76
H35	4990.99	317.09	8332.25	92
H15	13116.4	2583.39	9023.34	97
H59	6730.09	3524.52	2685.7	83
H36	4785.79	341.02	7379.43	96
H32	11710.83	5354.81	10100.75	92
H10A	1196.81	9064.64	7730.07	128
H10B	2161.79	10129.18	7898.36	128
H10C	1114.87	10052.09	8207.48	128
H63	6373	850.94	3807.82	84
H16	11289.01	2507.48	9232.57	84
H26	9734.42	5778.67	9654.99	84
H12	9884.66	3693.81	7693.29	91
H72	12235.6	3960.52	5427.7	92
H54	11497.15	2401.4	4285.14	82
H76	9702.93	2204.05	6023.46	94
H41	6952.27	48.34	6028.87	91
H62	5108.41	-279.01	3122.14	100
H60	5449.45	2426.2	2009.07	100
H75	11086.93	1271.43	6718.9	108
H31	13098.54	4583.58	10831.07	107
H13	11733.61	3815.61	7491.98	104
H61	4638.01	504.97	2223.57	94
H53	13274.09	2529.07	4048.65	96

Atom	x	y	z	U_{eq}
H55A	9501.65	1560.39	4091.14	106
H55B	8896.17	1208.38	3474.92	106
H52	13450.26	2162.39	3160.04	110
H50	10027.16	1411.79	2729.88	105
H73	13608.9	3008.73	6115.7	109
H51	11853.18	1578.89	2491.24	119
H30	12555.04	3634.21	11576.85	112
H28	9199.75	4030.79	10831.99	122
H74	13045.56	1725.3	6776.54	107
H48A	9243.86	1073.93	7110.92	151
H48B	8737.8	-155.89	6768.82	151
H48C	8130.93	384.99	7236.4	151
H29	10600.8	3303.34	11571.44	137

12. NMR Spectra

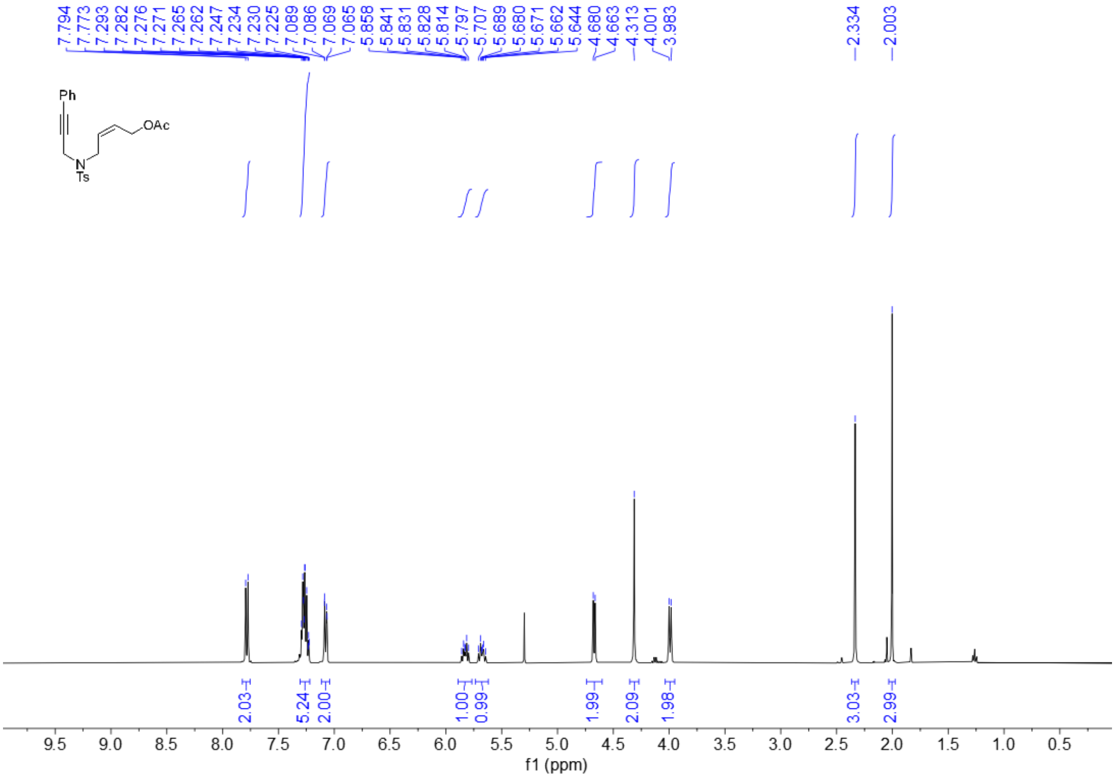

Supplementary Figure 141. ^{1}H NMR spectrum (400 MHz, $CDCl_3$) of **1a**

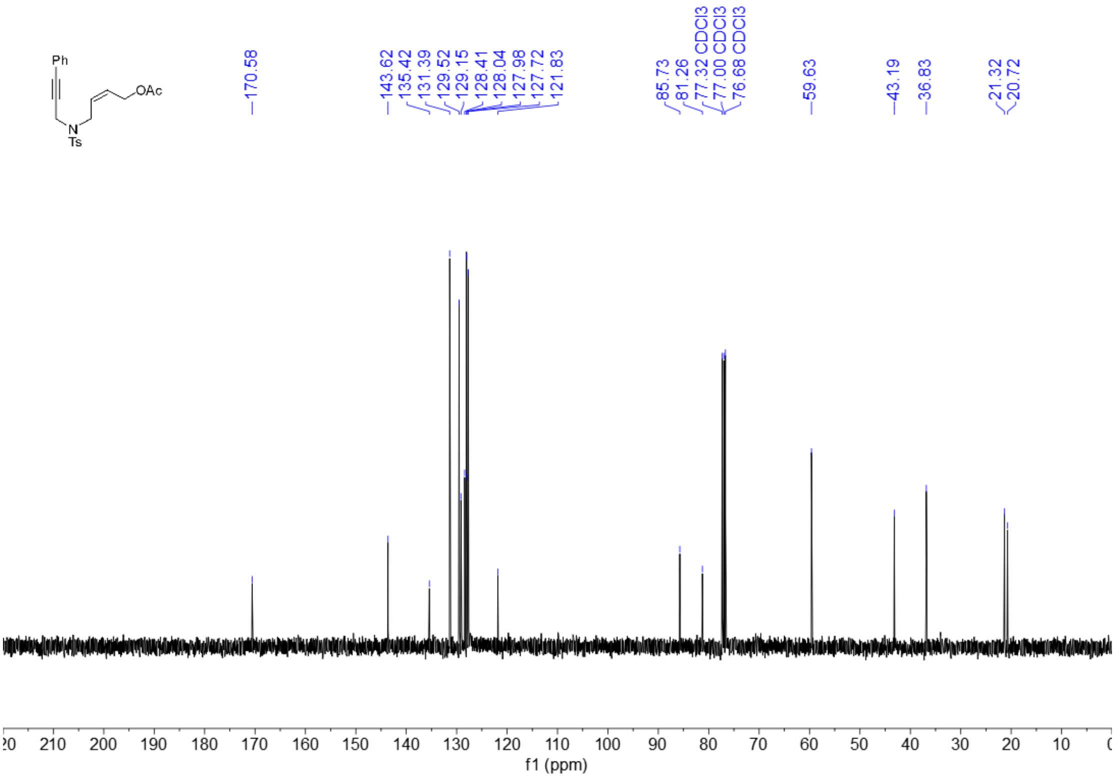

Supplementary Figure 142. ^{13}C NMR spectrum (100 MHz, $CDCl_3$) of **1a**

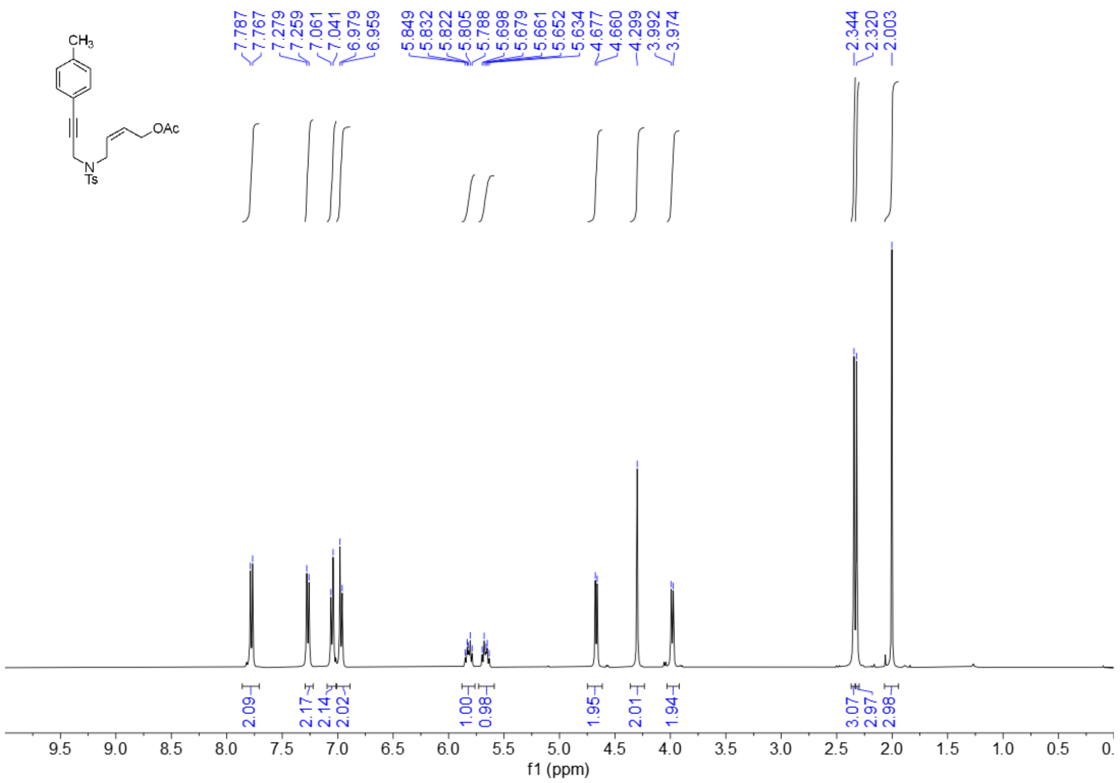

Supplementary Figure 143. ^{1}H NMR spectrum (400 MHz, $CDCl_3$) of **1b**

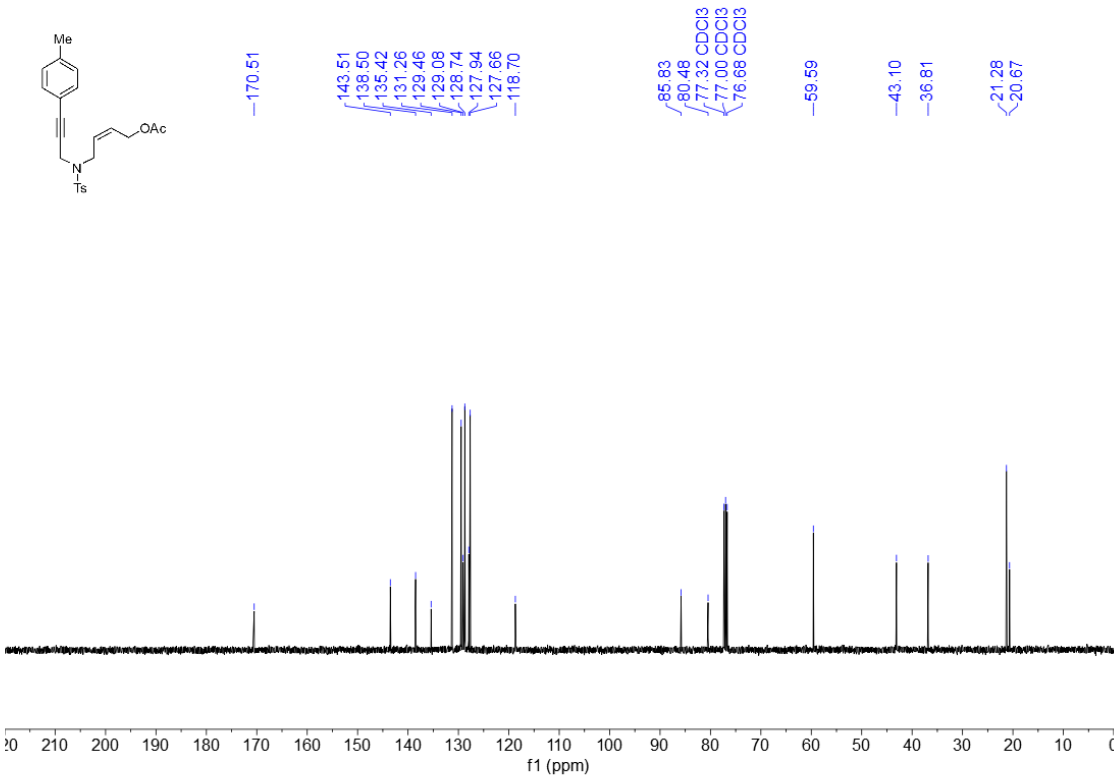

Supplementary Figure 144. ^{13}C NMR spectrum (100 MHz, $CDCl_3$) of **1b**

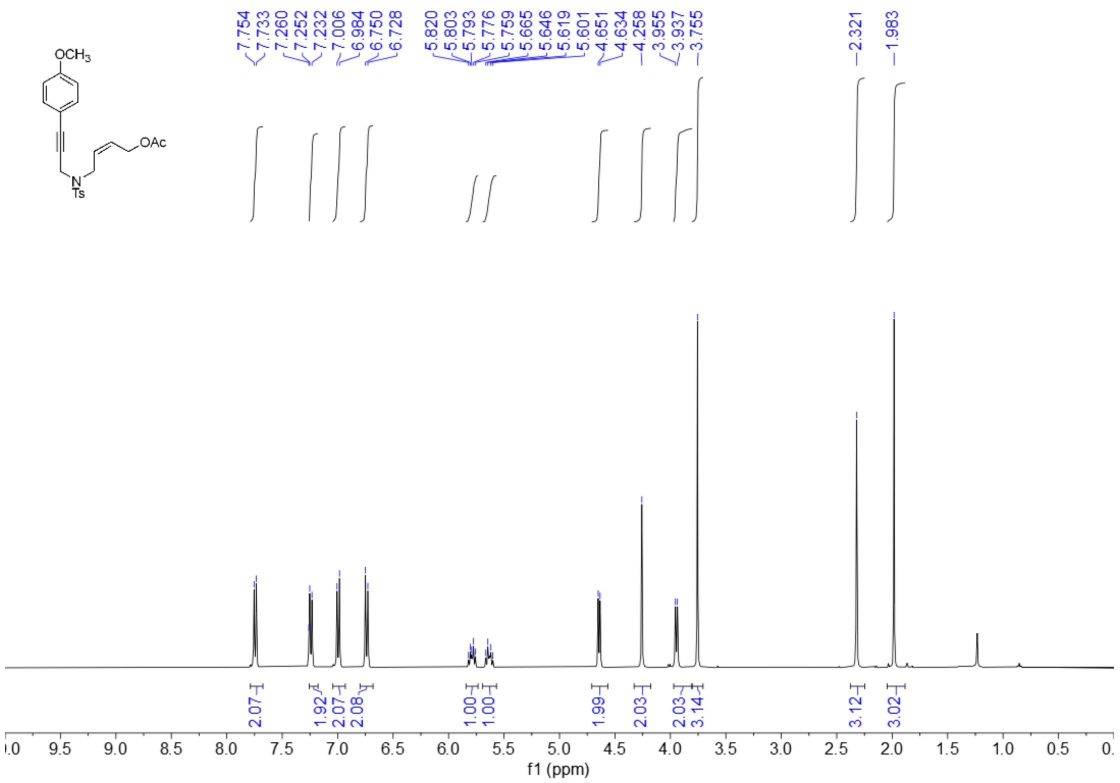

Supplementary Figure 145. ^{1}H NMR spectrum (400 MHz, $CDCl_3$) of **1c**

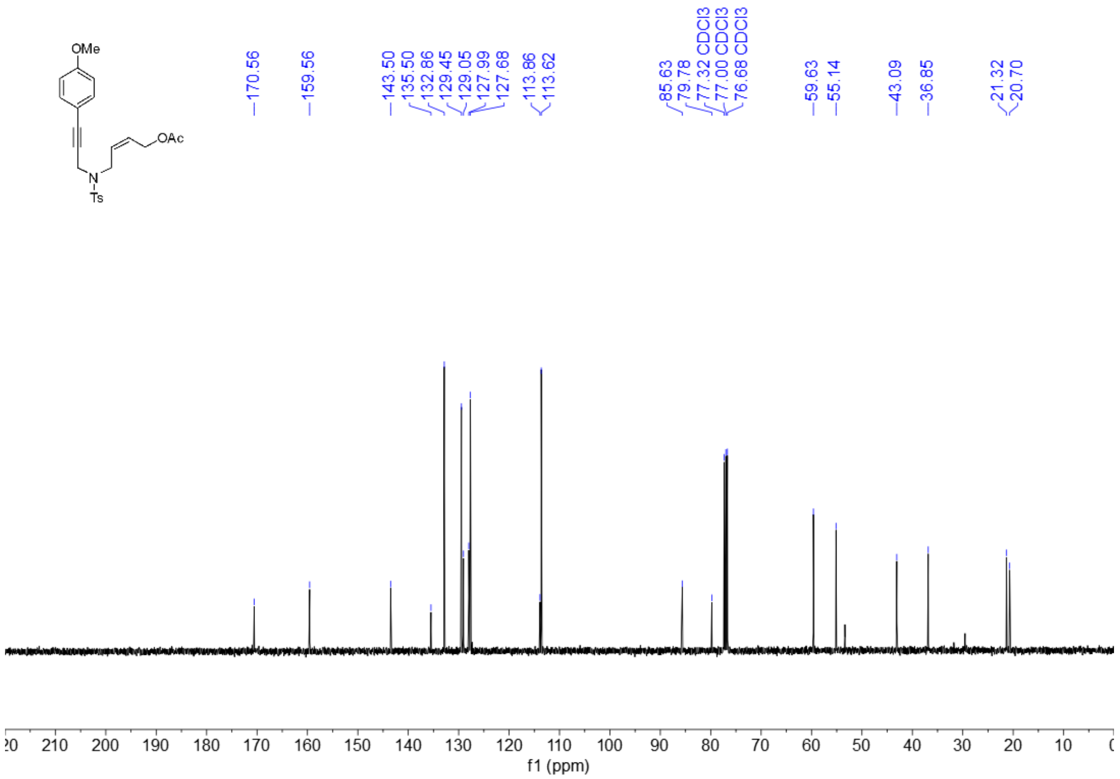

Supplementary Figure 146. ^{13}C NMR spectrum (100 MHz, $CDCl_3$) of **1c**

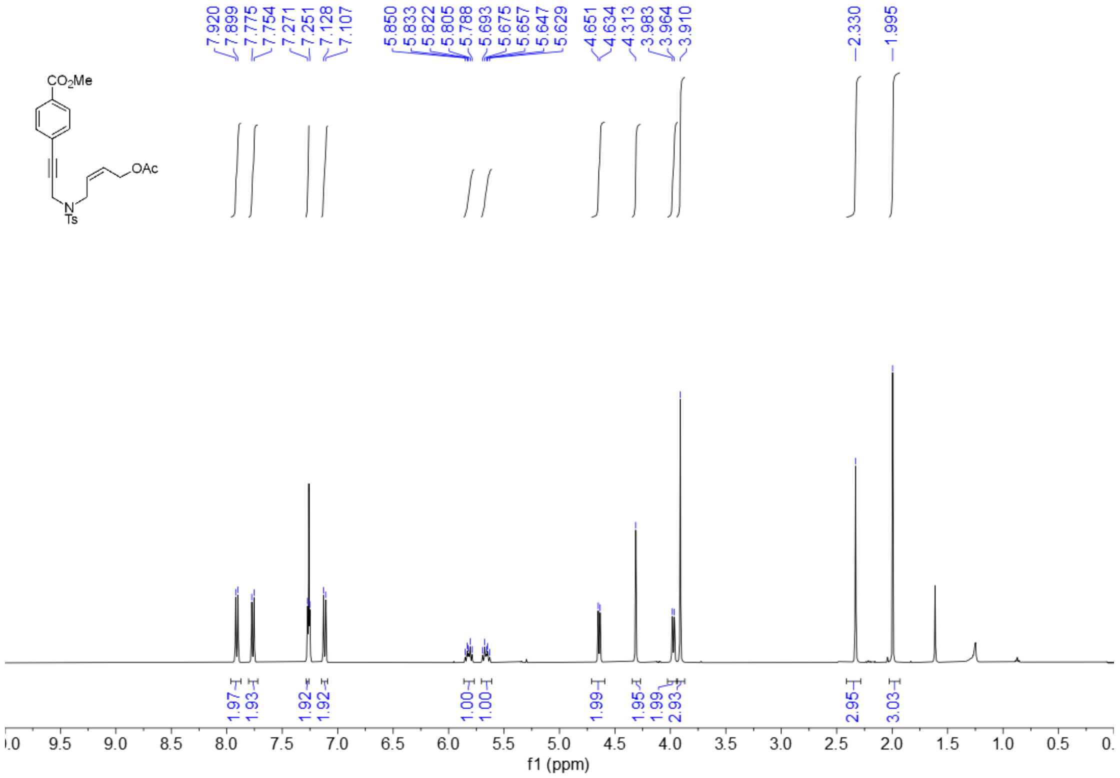

Supplementary Figure 147. ^{1}H NMR spectrum (400 MHz, $CDCl_3$) of **1d**

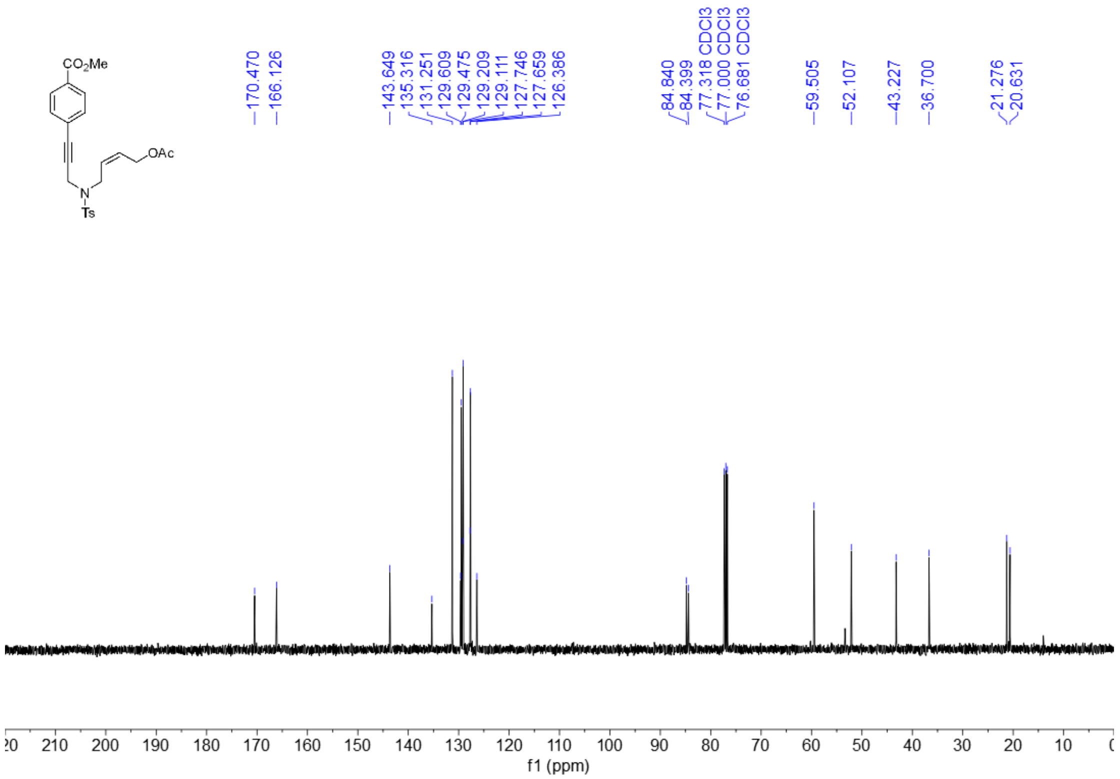

Supplementary Figure 148. ^{13}C NMR spectrum (100 MHz, $CDCl_3$) of **1d**

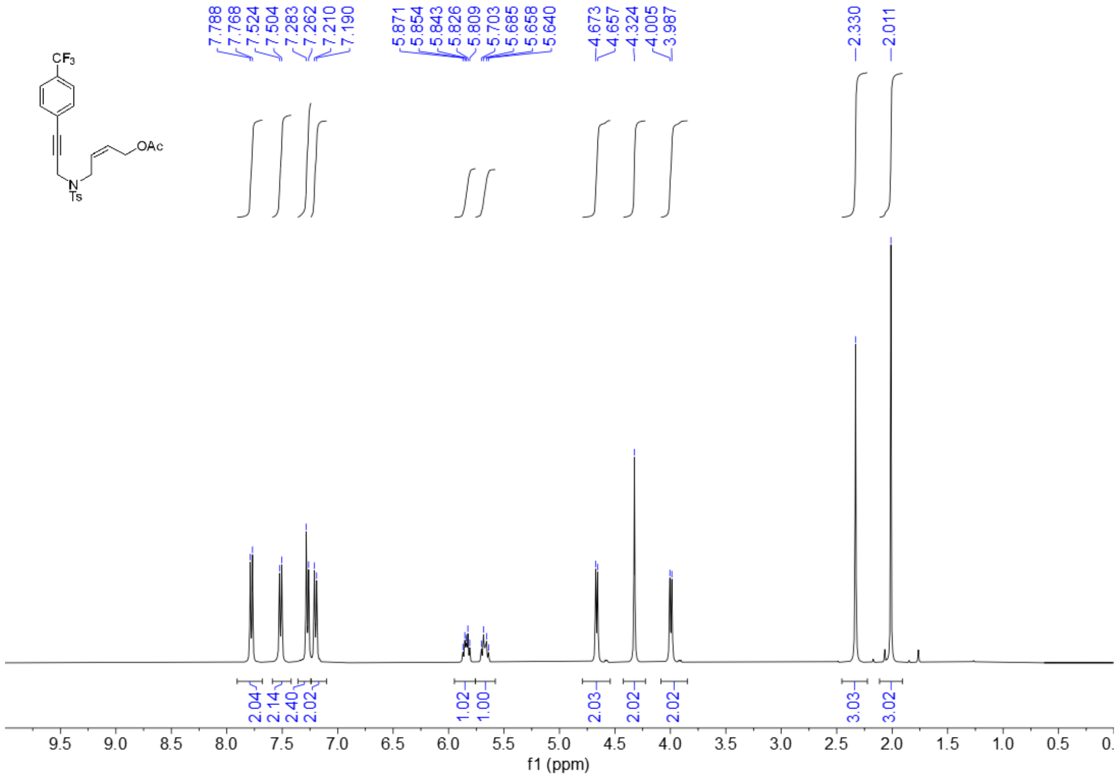

Supplementary Figure 149. ^{1}H NMR spectrum (400 MHz, $CDCl_3$) of **1e**

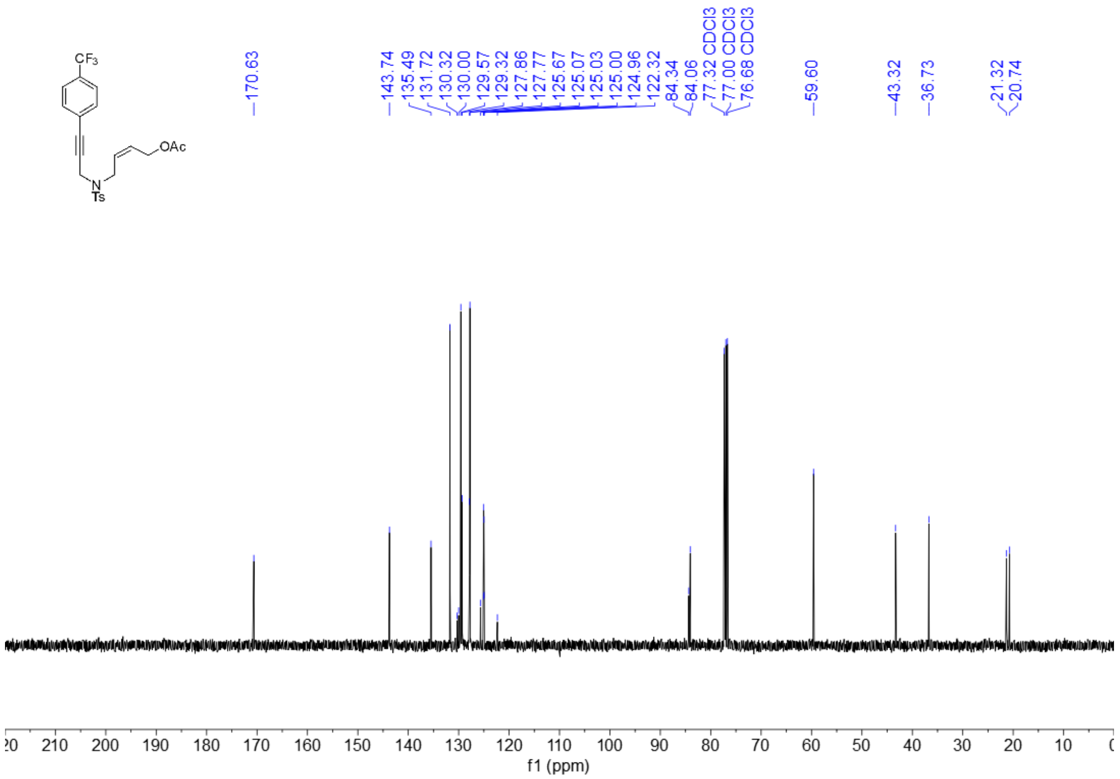

Supplementary Figure 150. ^{13}C NMR spectrum (100 MHz, $CDCl_3$) of **1e**

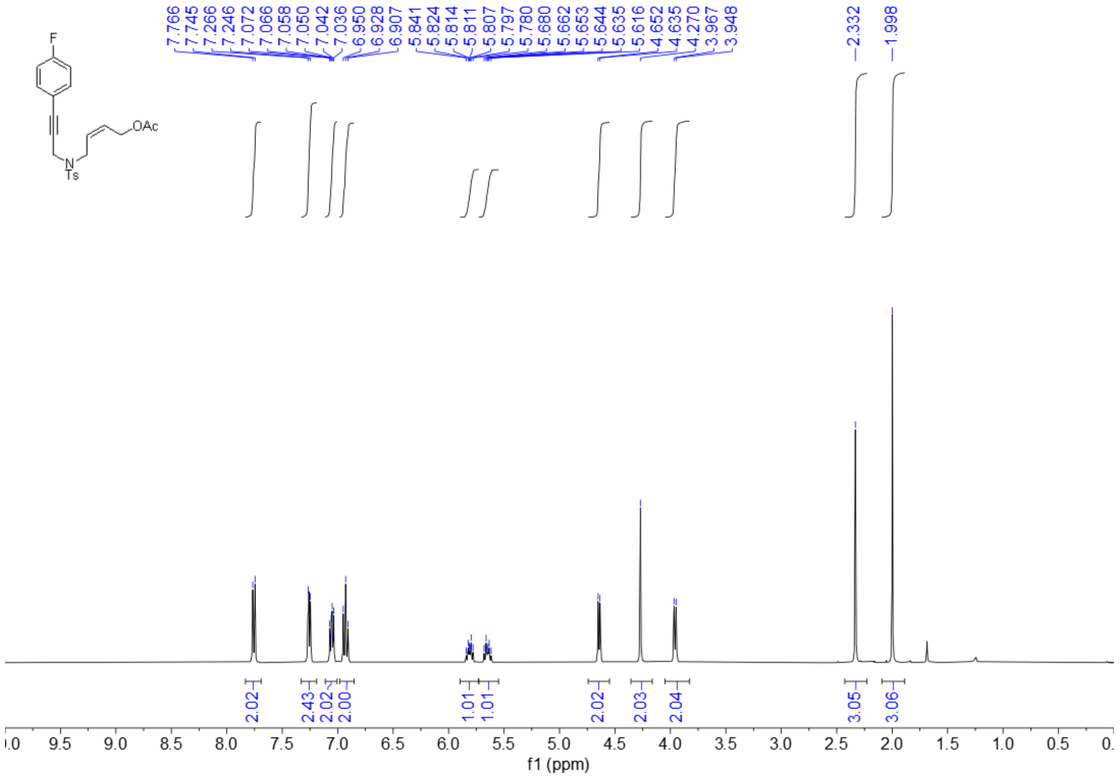

Supplementary Figure 151. ^{1}H NMR spectrum (400 MHz, $CDCl_3$) of **1f**

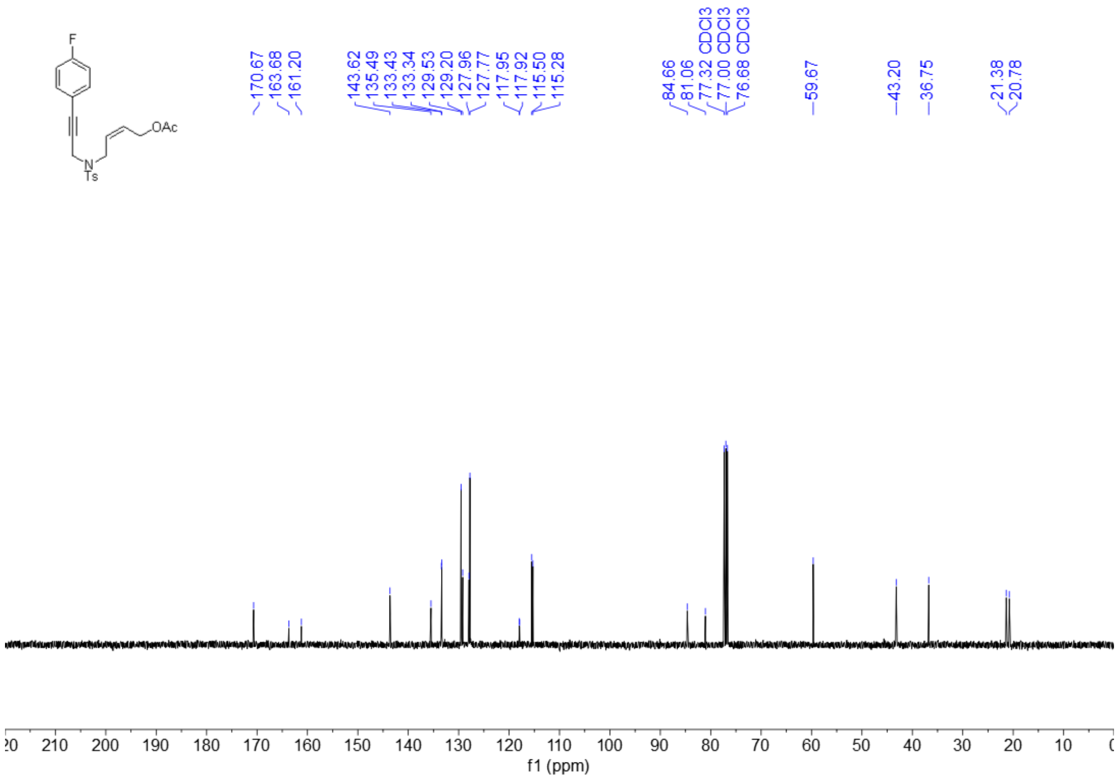

Supplementary Figure 152. ^{13}C NMR spectrum (100 MHz, $CDCl_3$) of **1f**

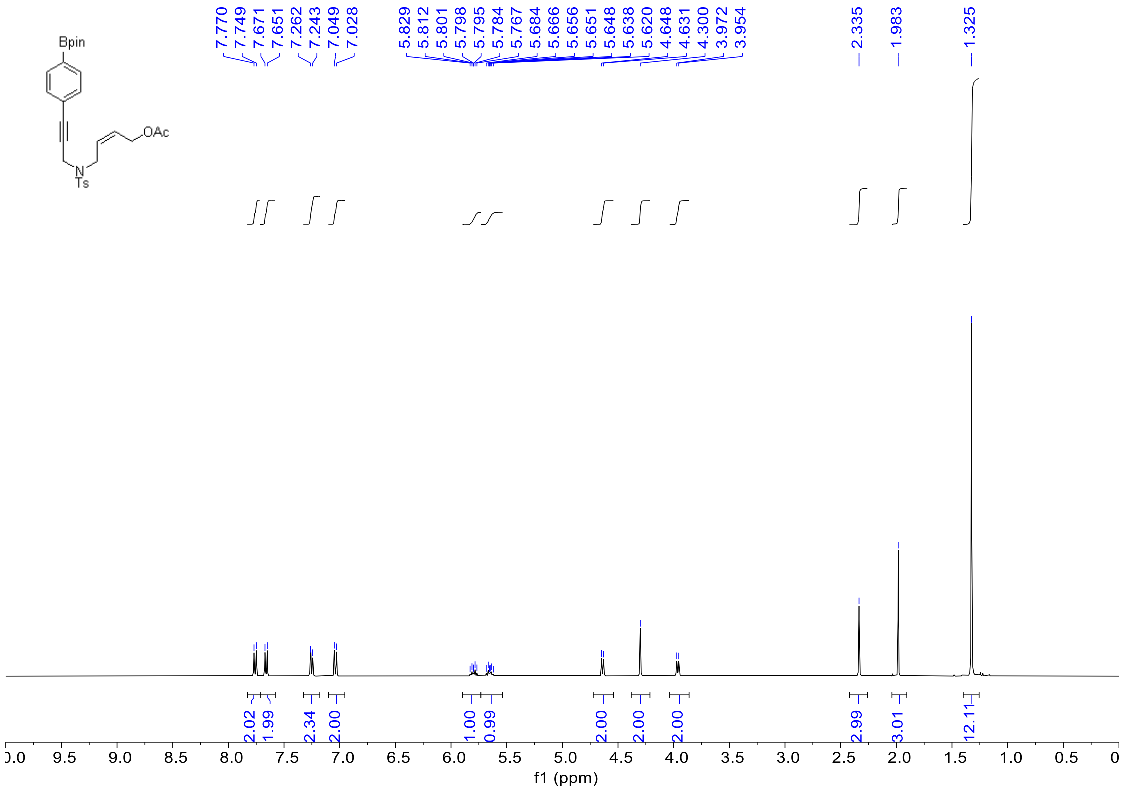

Supplementary Figure 153. ^{1}H NMR spectrum (400 MHz, $CDCl_3$) of **1g**

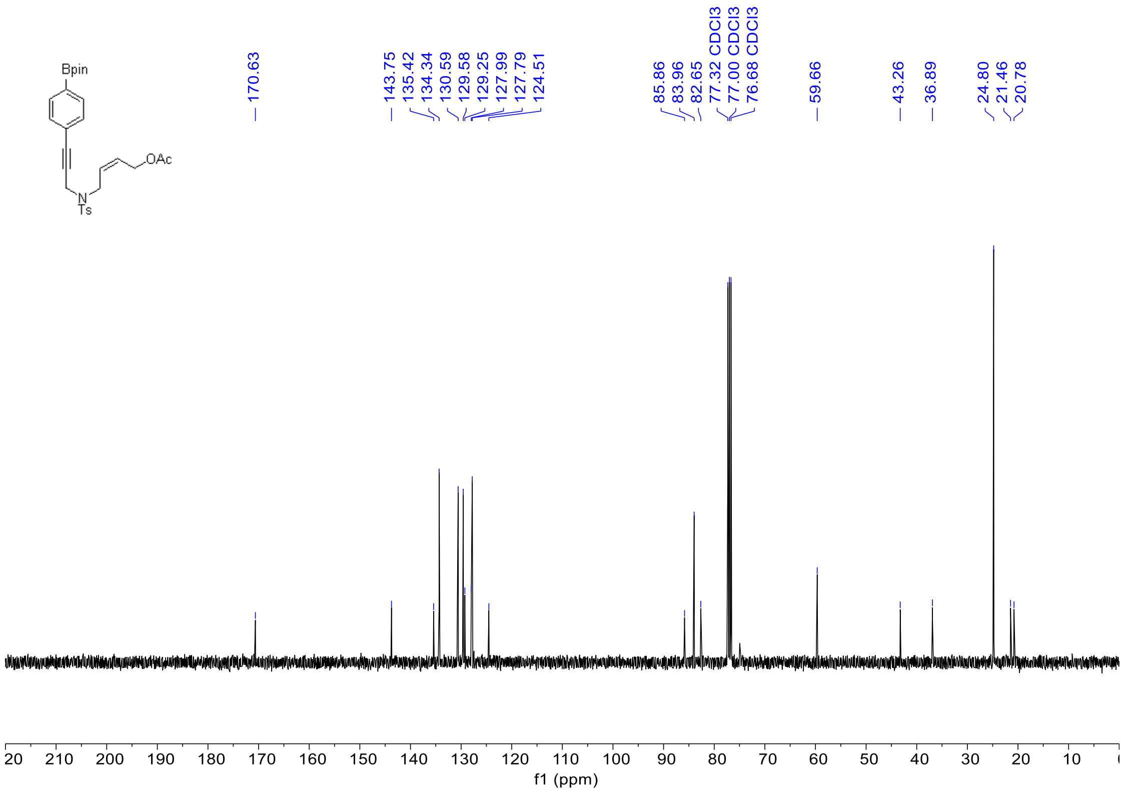

Supplementary Figure 154. ^{13}C NMR spectrum (100 MHz, $CDCl_3$) of **1g**

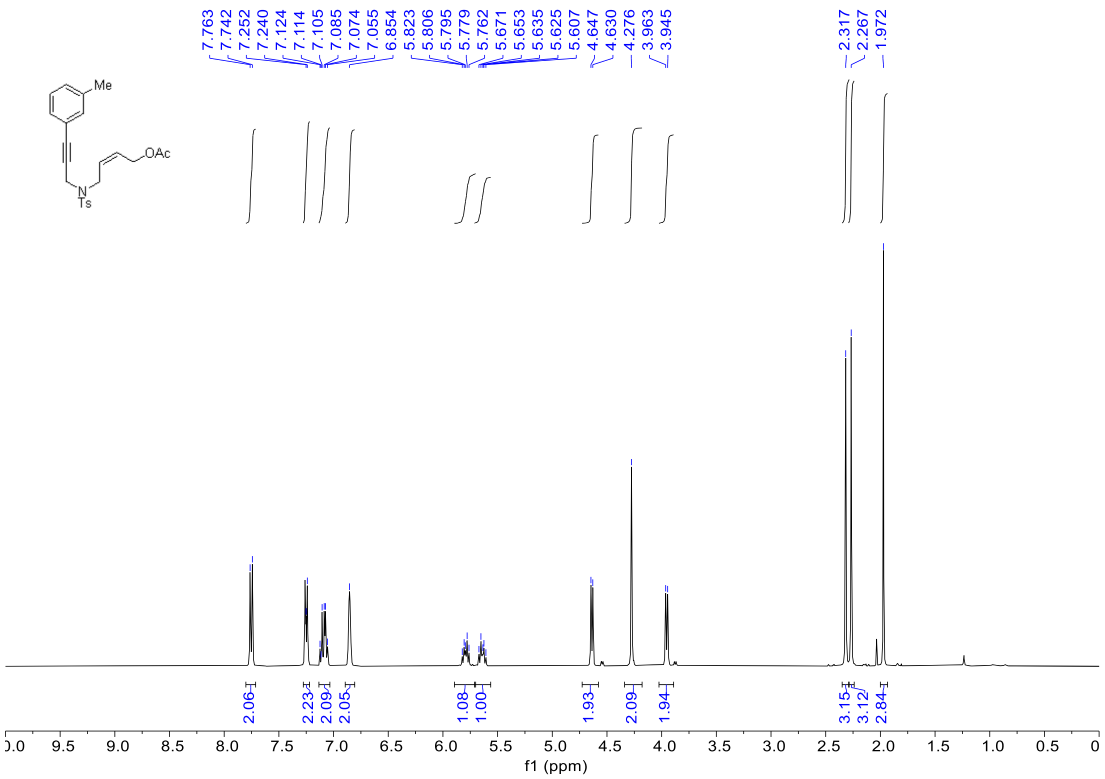

Supplementary Figure 155. ^{1}H NMR spectrum (400 MHz, $CDCl_3$) of **1h**

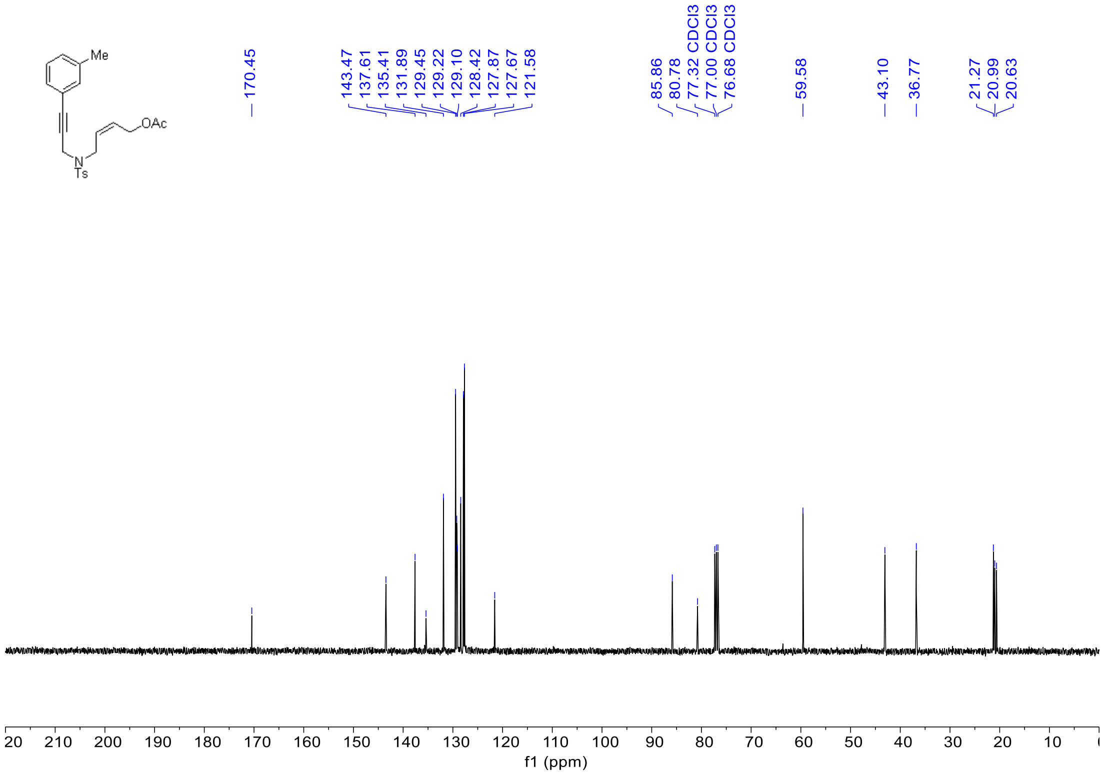

Supplementary Figure 156. ^{13}C NMR spectrum (100 MHz, $CDCl_3$) of **1h**

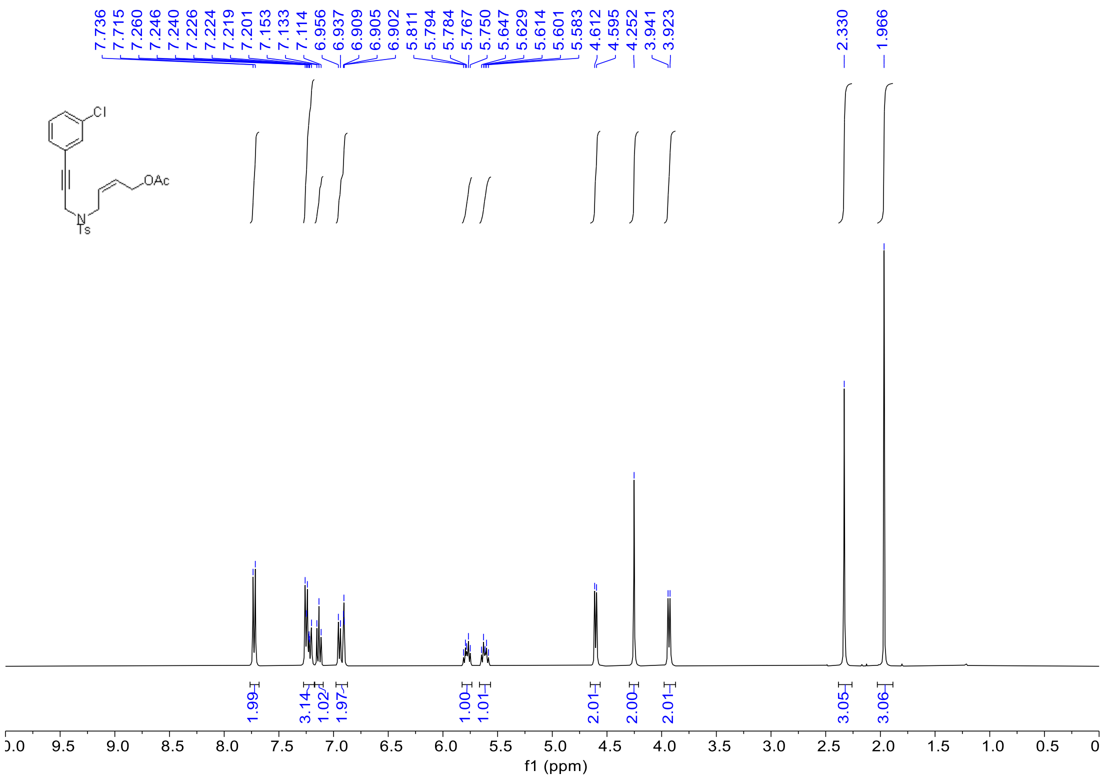

Supplementary Figure 157. ^{1}H NMR spectrum (400 MHz, $CDCl_3$) of **1i**

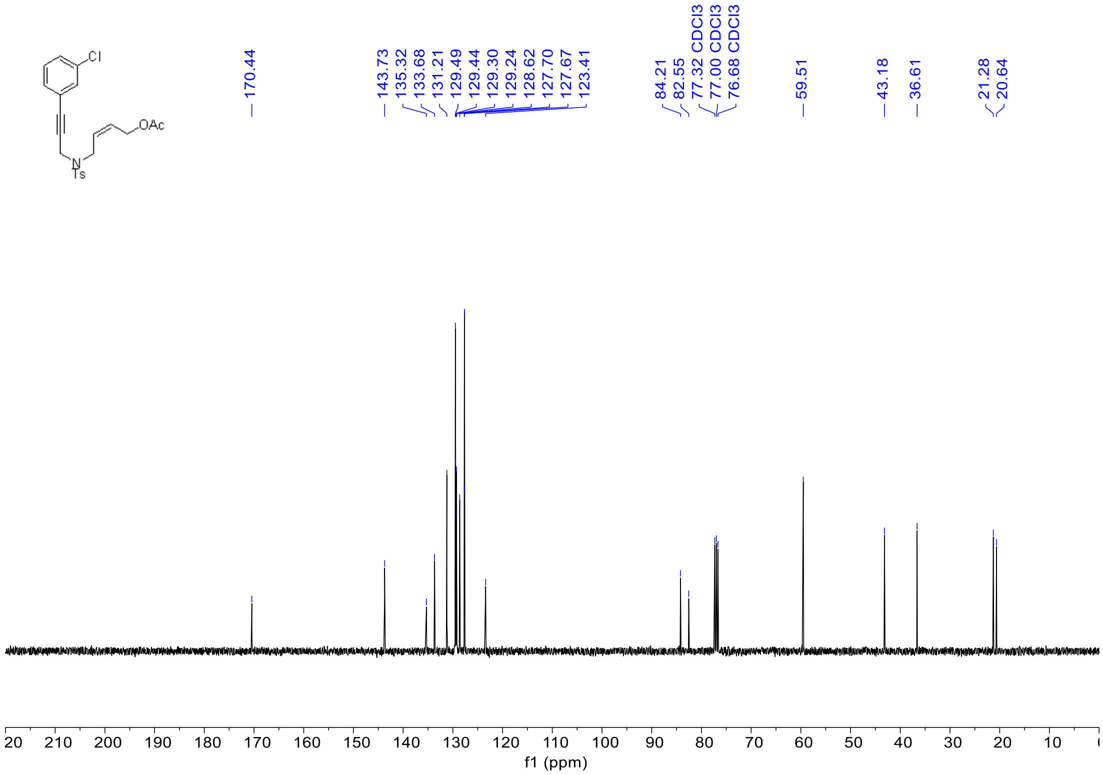

Supplementary Figure 158. ^{13}C NMR spectrum (100 MHz, $CDCl_3$) of **1i**

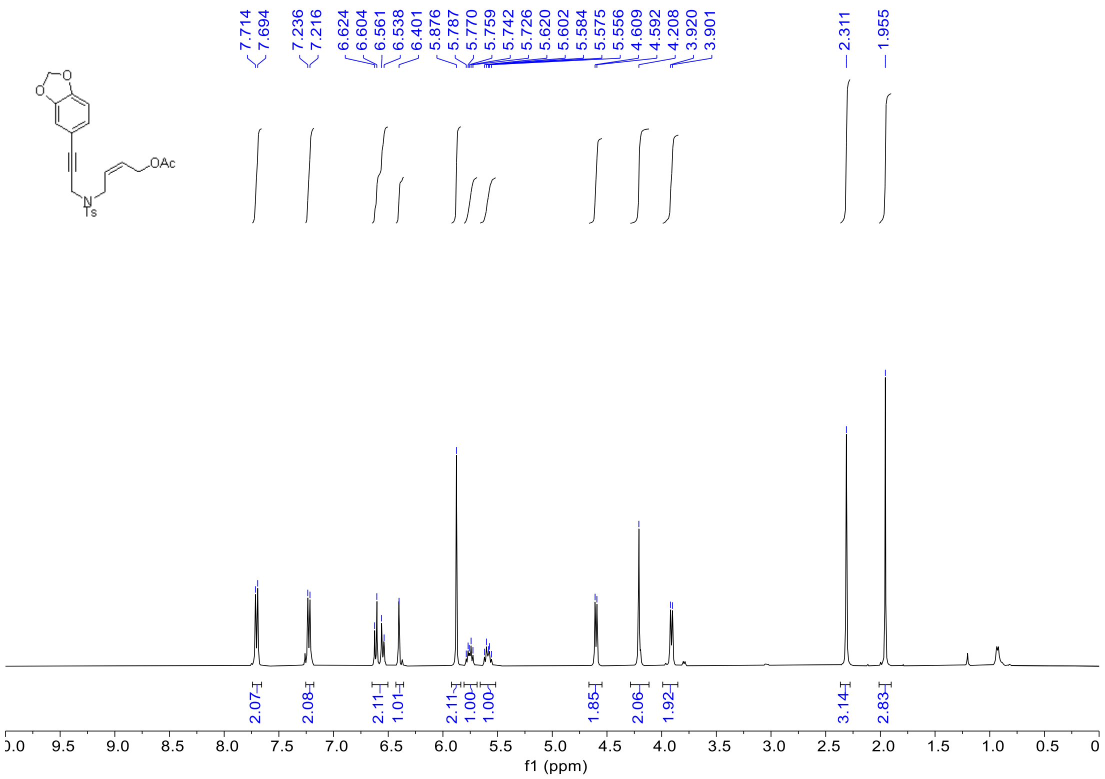

Supplementary Figure 159. ^{1}H NMR spectrum (400 MHz, $CDCl_3$) of **1j**

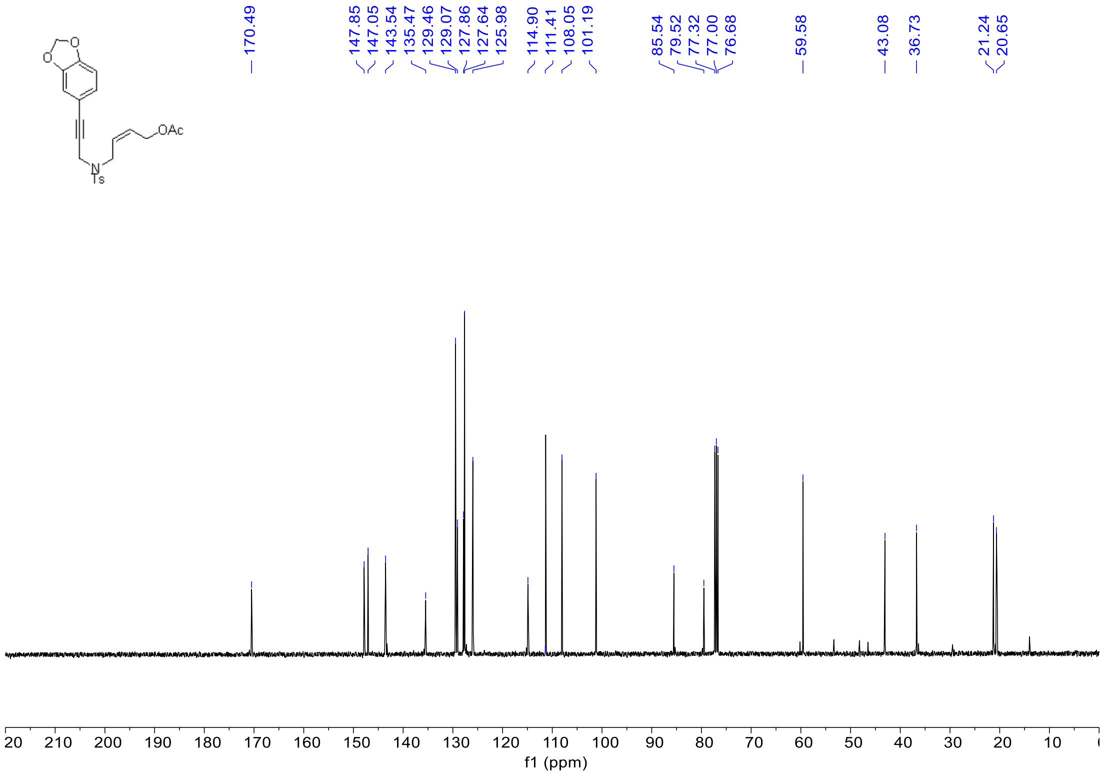

Supplementary Figure 160. ^{13}C NMR spectrum (100 MHz, $CDCl_3$) of **1j**

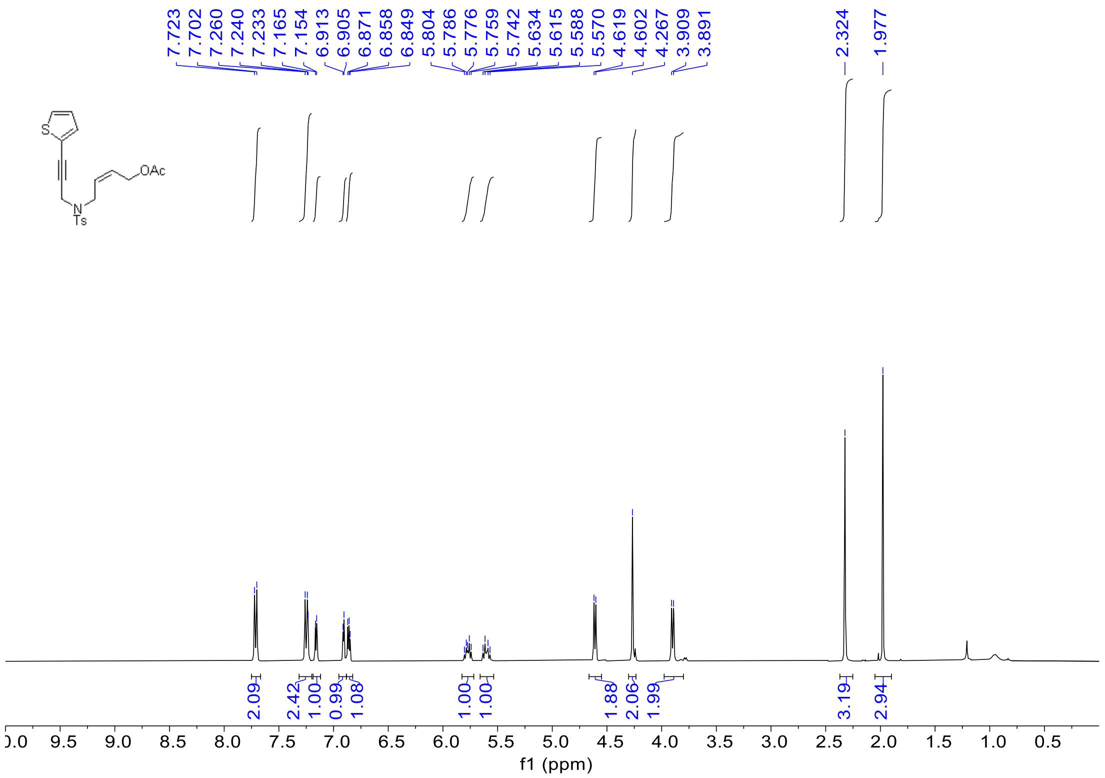

Supplementary Figure 161. ^{1}H NMR spectrum (400 MHz, $CDCl_3$) of **1k**

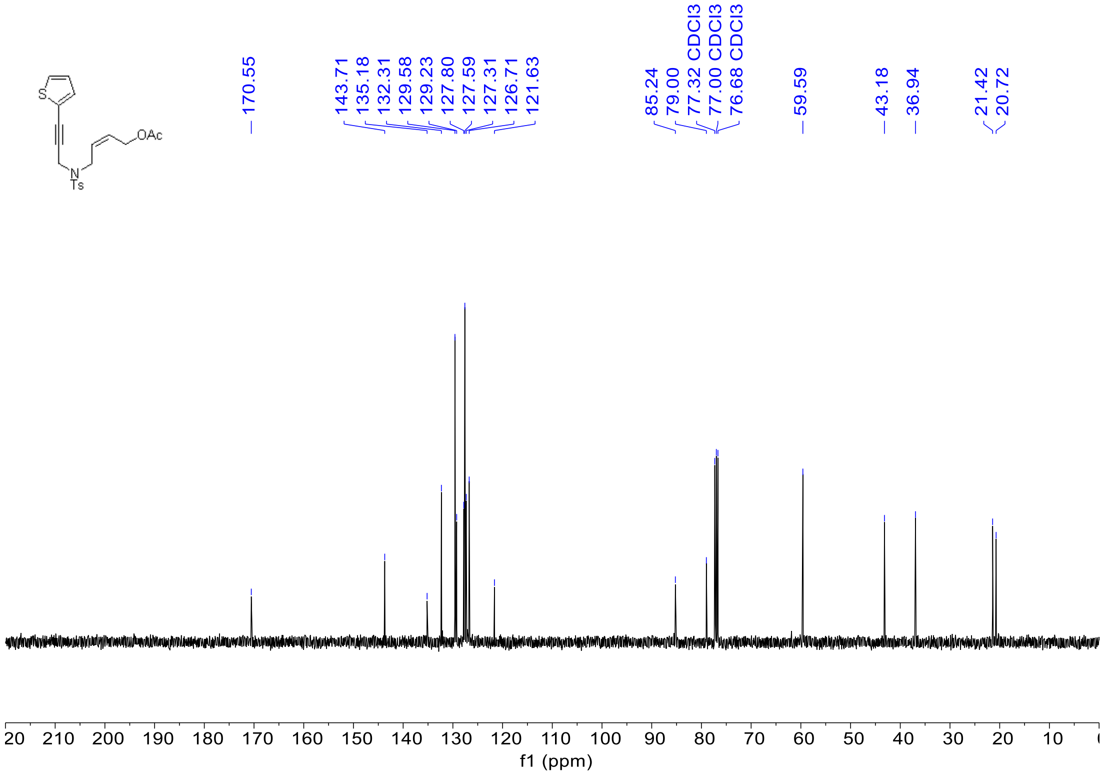

Supplementary Figure 162. ^{13}C NMR spectrum (100 MHz, $CDCl_3$) of **1k**

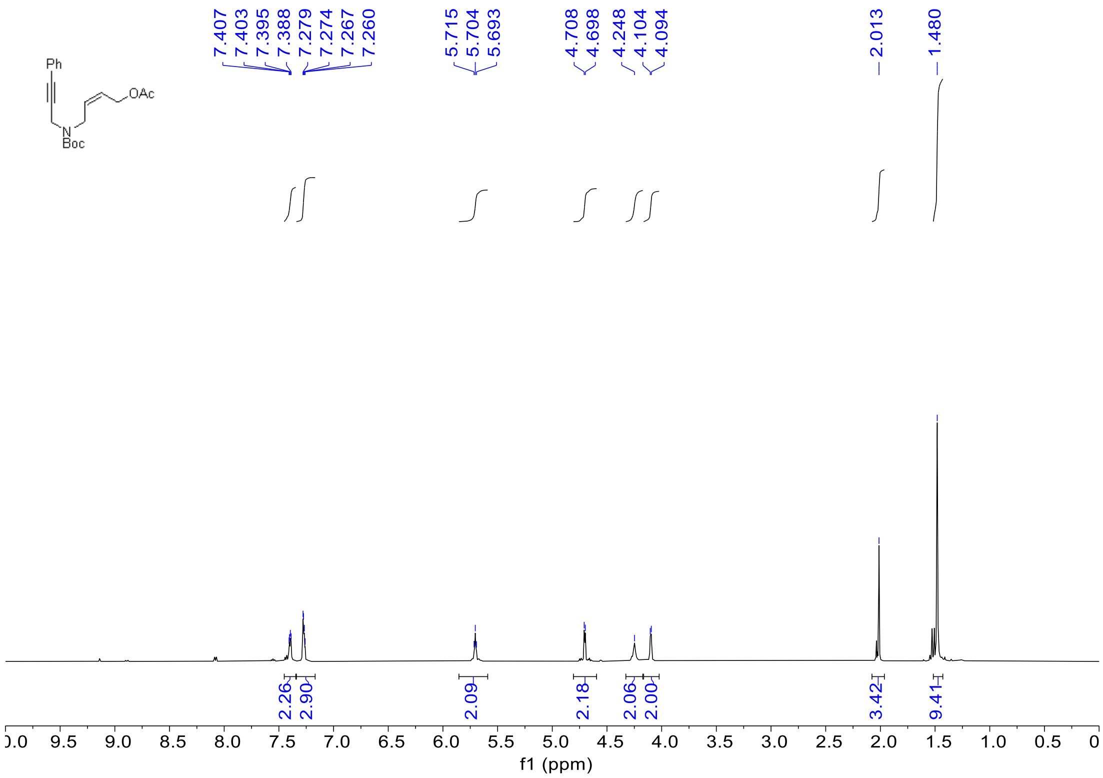

Supplementary Figure 163. ^{1}H NMR spectrum (500 MHz, $CDCl_3$, 50 °C) of **1l**

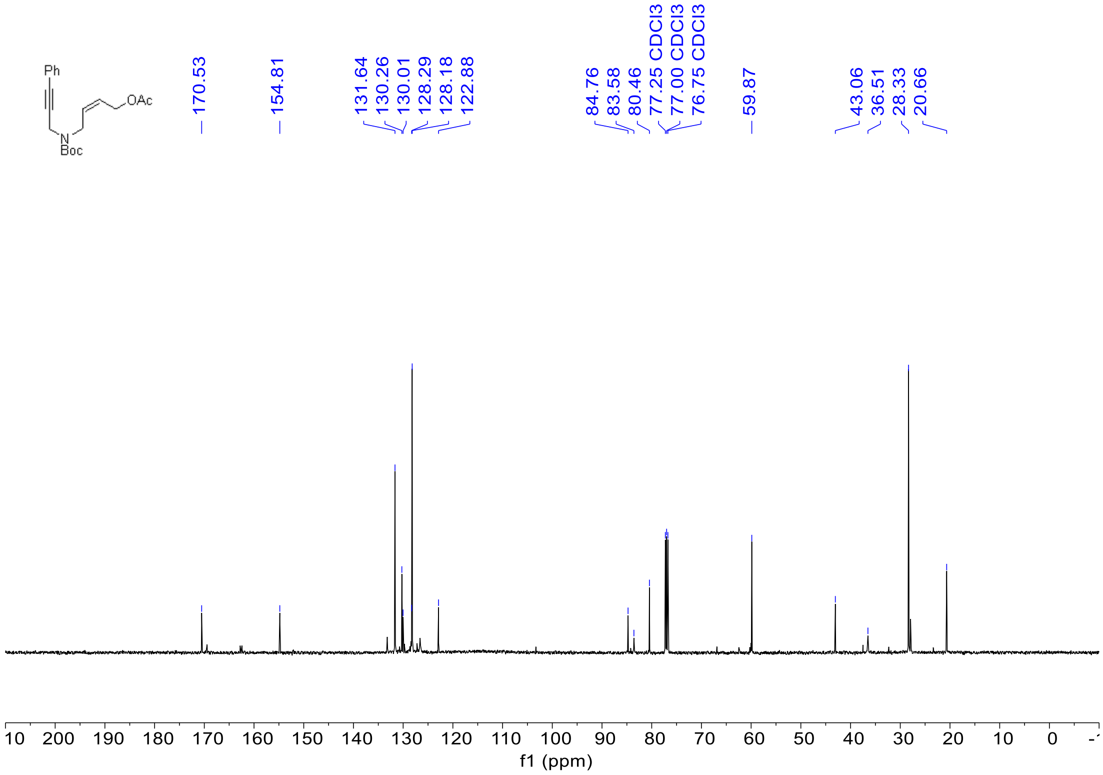

Supplementary Figure 164. ^{13}C NMR spectrum (125 MHz, $CDCl_3$, 50 °C) of **1l**

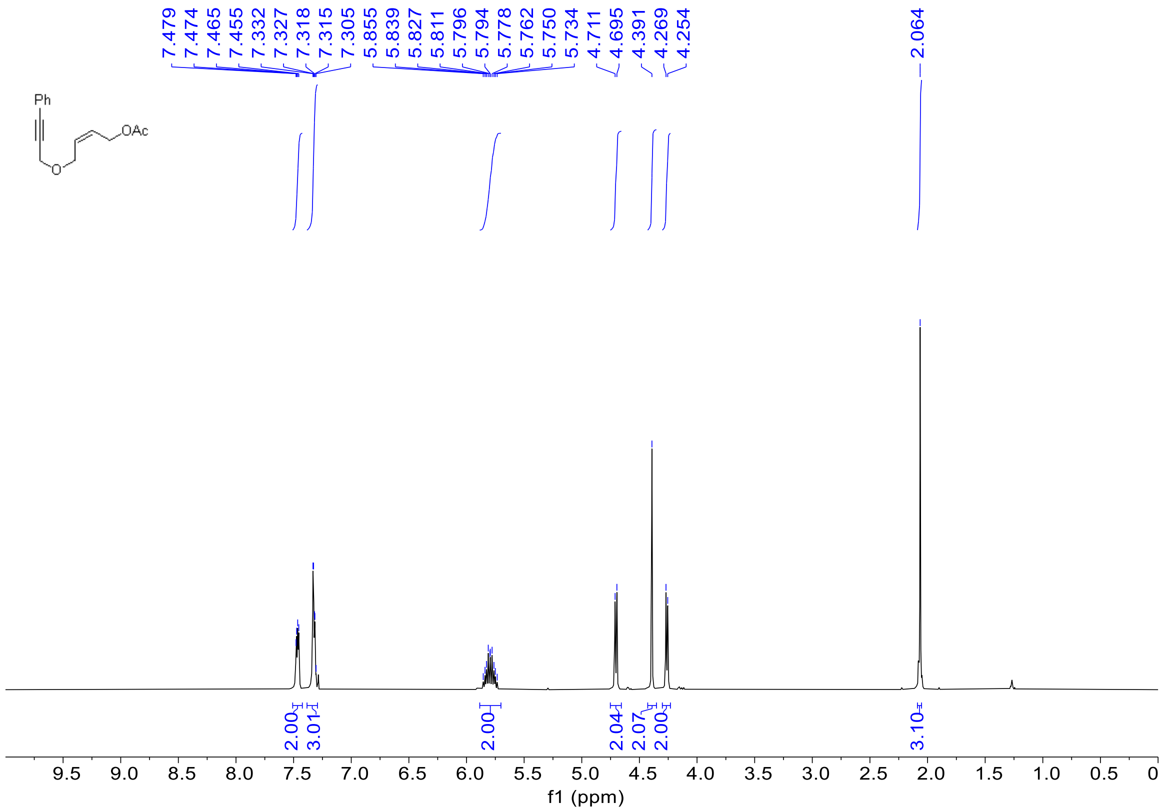

Supplementary Figure 165. ^{1}H NMR spectrum (400 MHz, $CDCl_3$) of **1m**

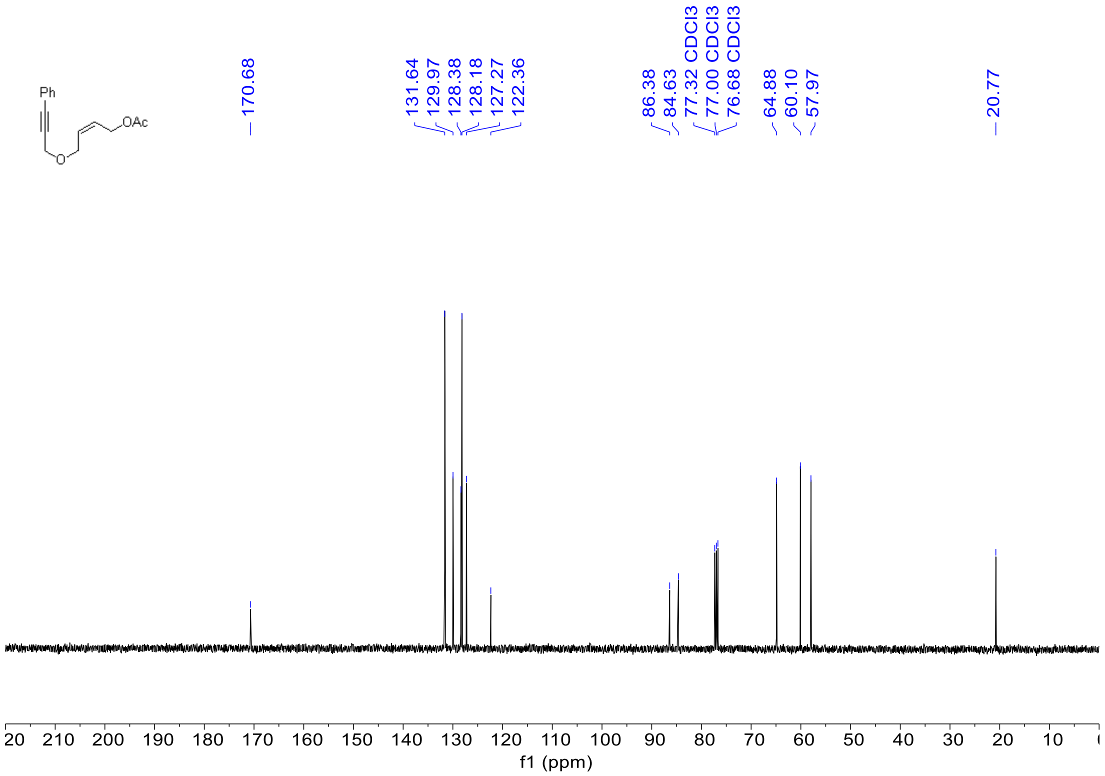

Supplementary Figure 166. ^{13}C NMR spectrum (100 MHz, $CDCl_3$) of **1m**

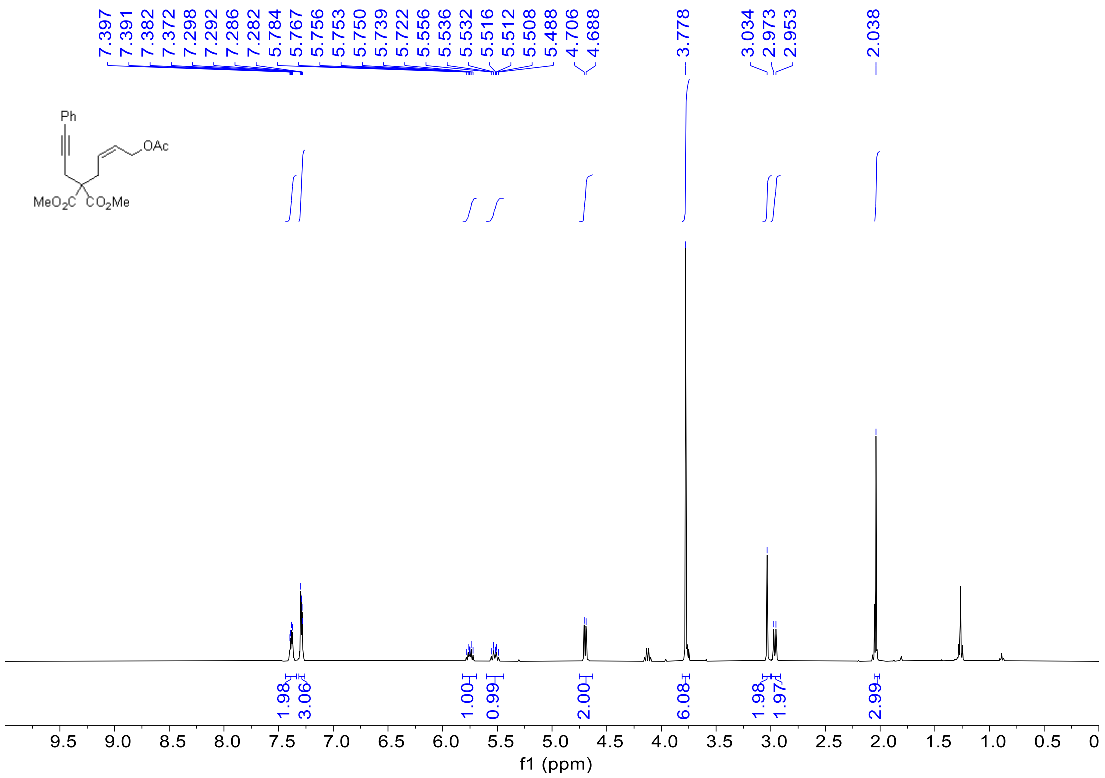

Supplementary Figure 167. ^{1}H NMR spectrum (400 MHz, $CDCl_3$) of **1n**

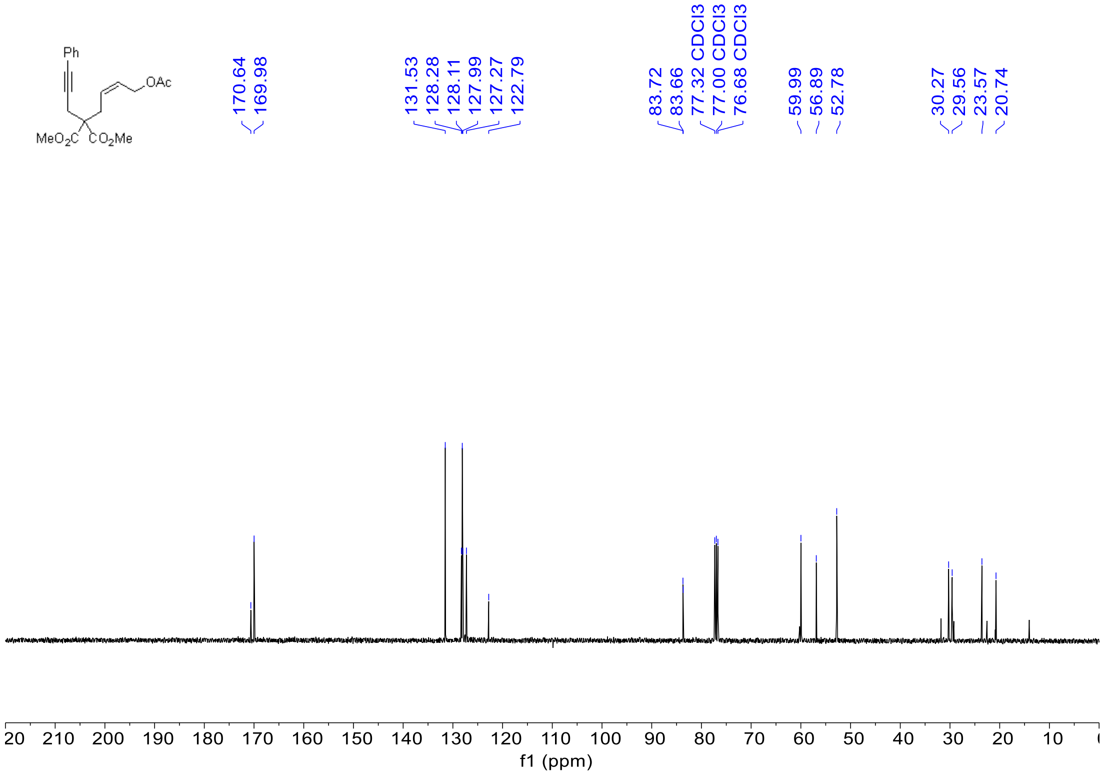

Supplementary Figure 168. ^{13}C NMR spectrum (100 MHz, $CDCl_3$) of **1n**

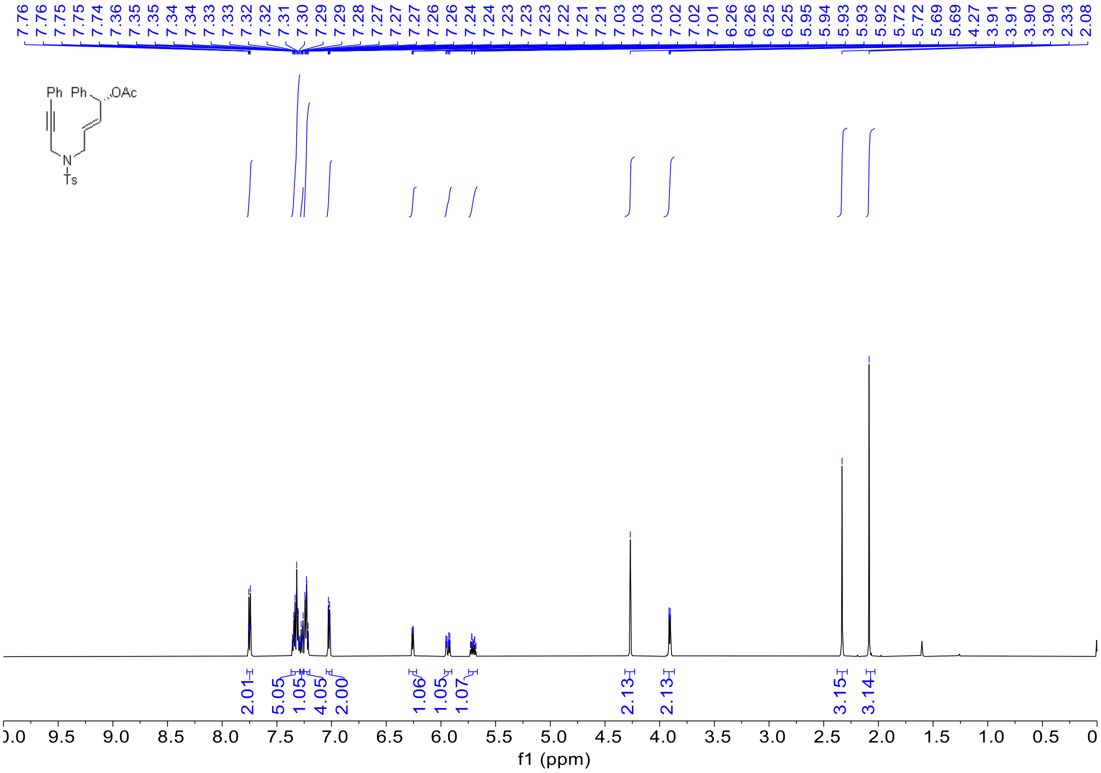

Supplementary Figure 169. ^{1}H NMR spectrum (600 MHz, $CDCl_3$) of ***(E)*-4a**

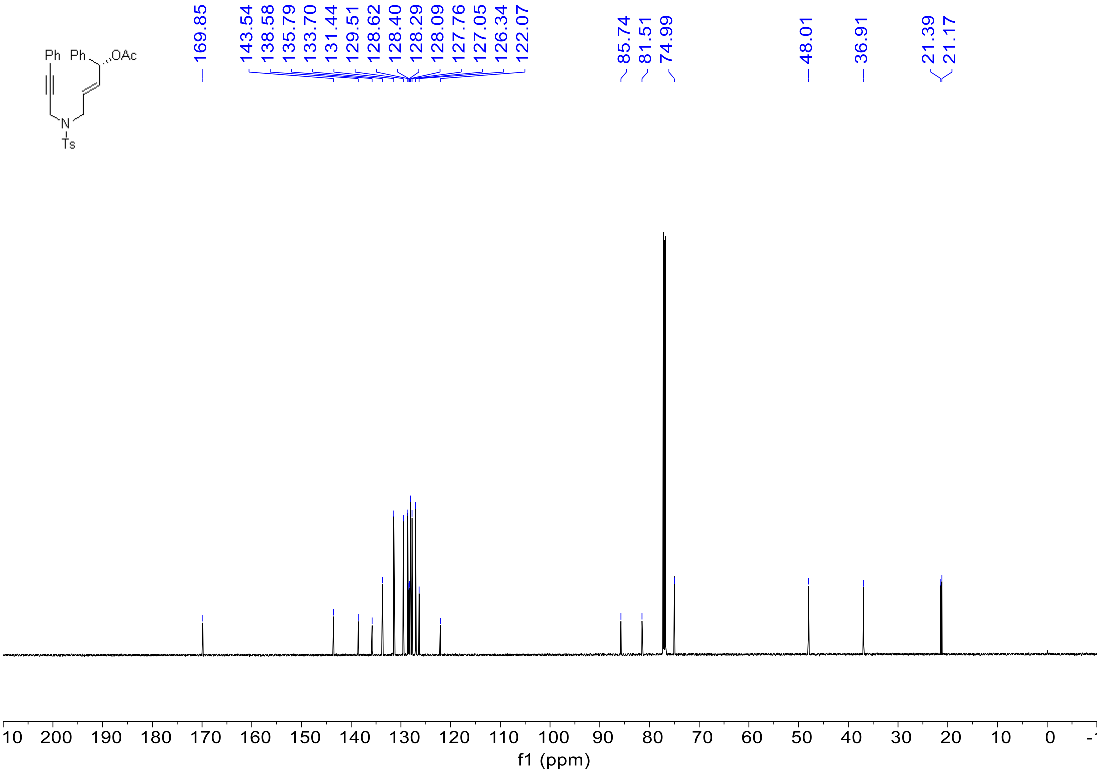

Supplementary Figure 170. ^{13}C NMR spectrum (151 MHz, $CDCl_3$) of ***(E)*-4a**

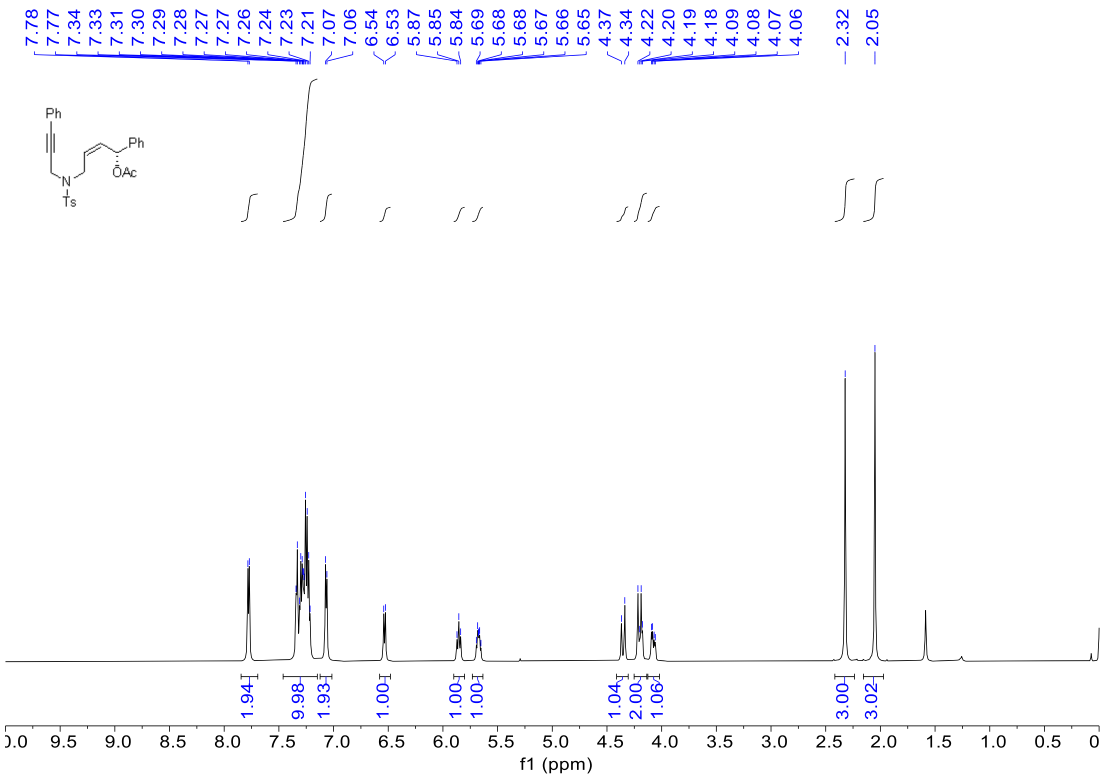

Supplementary Figure 171. ^{1}H NMR spectrum (600 MHz, $CDCl_3$) of ***(Z)*-4a**

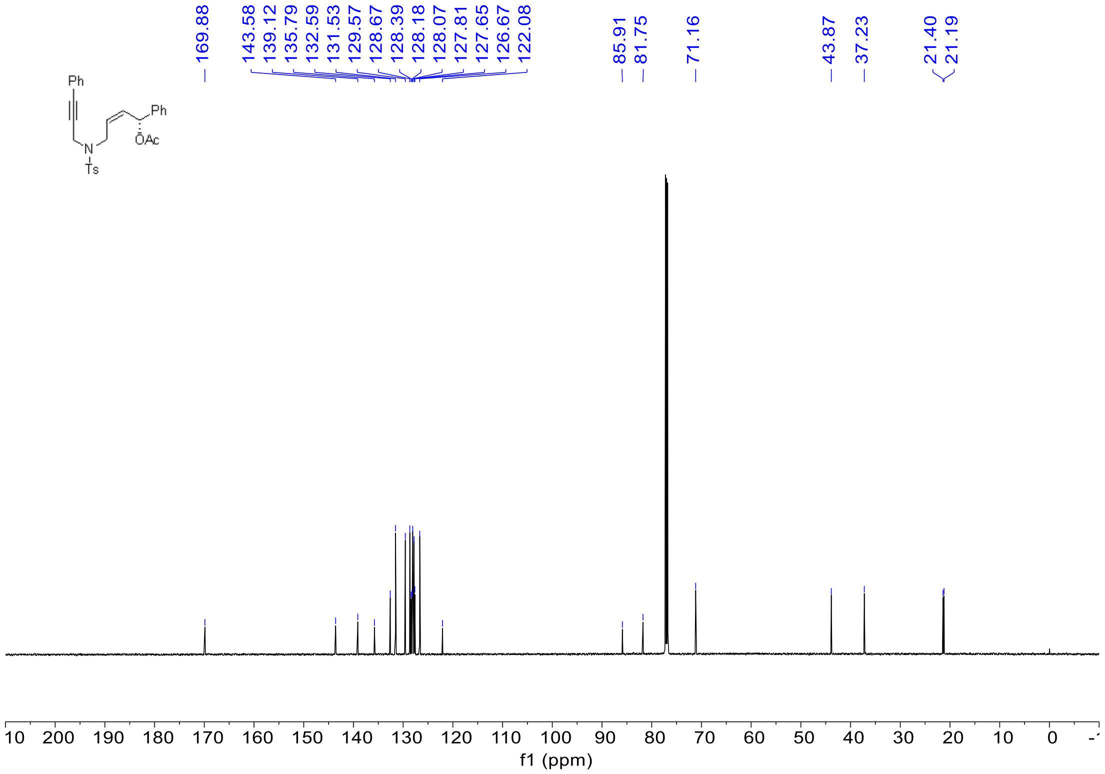

Supplementary Figure 172. ^{13}C NMR spectrum (151 MHz, $CDCl_3$) of ***(Z)*-4a**

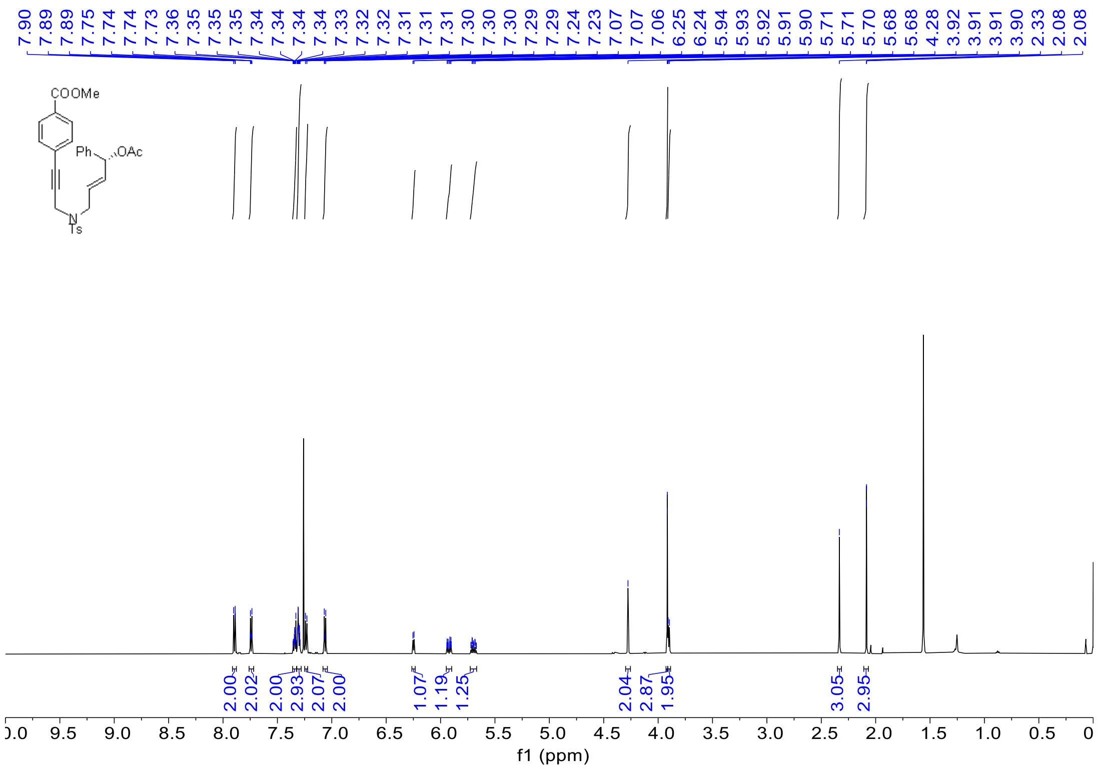

Supplementary Figure 173. ^{1}H NMR spectrum (600 MHz, $CDCl_3$) of **4b**

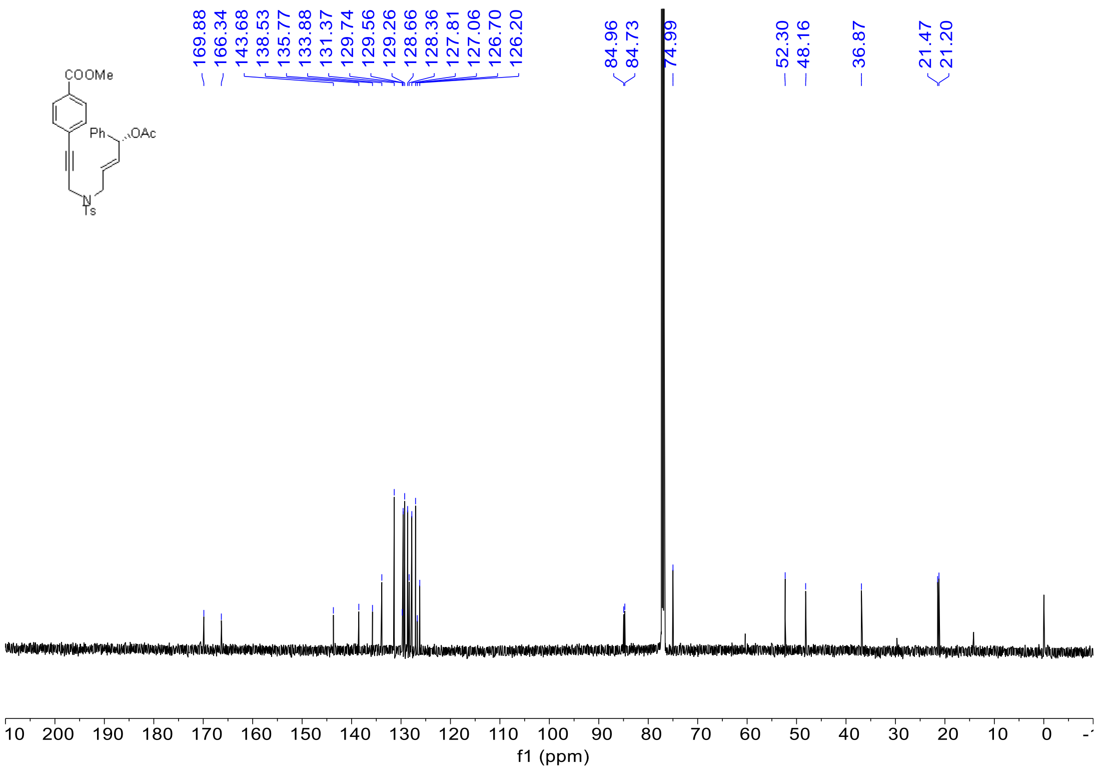

Supplementary Figure 174. ^{13}C NMR spectrum (151 MHz, $CDCl_3$) of **4b**

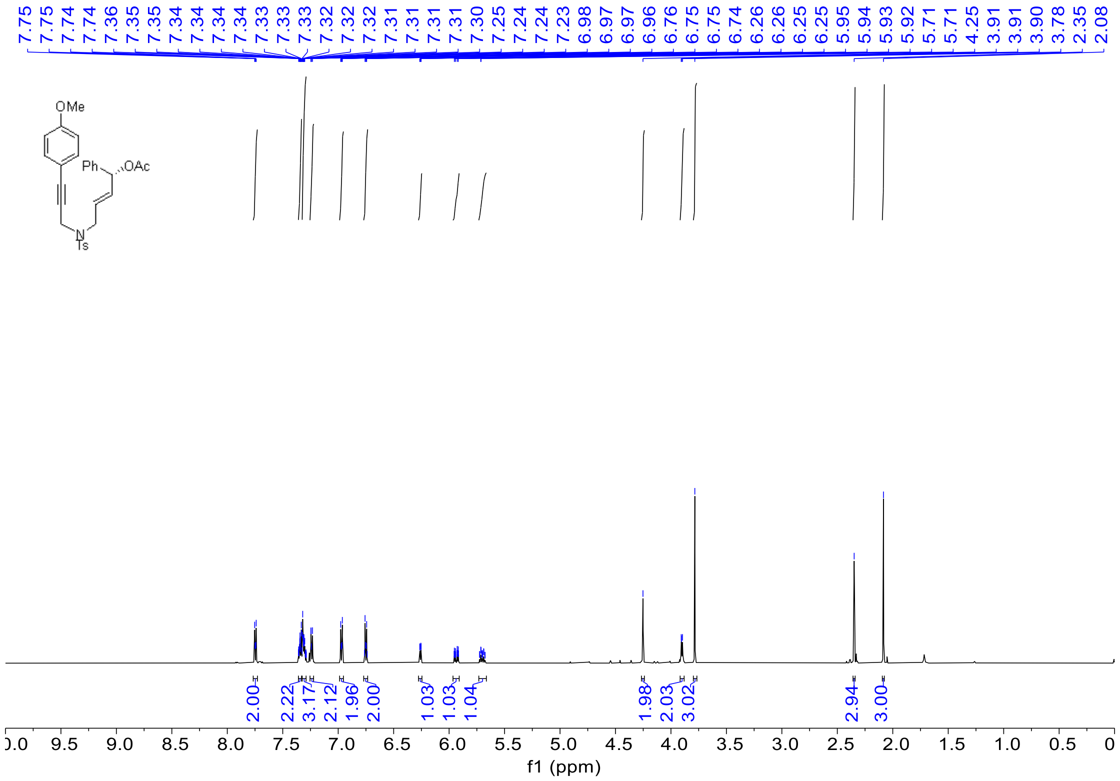

Supplementary Figure 173. ^{1}H NMR spectrum (600 MHz, $CDCl_3$) of **4c**

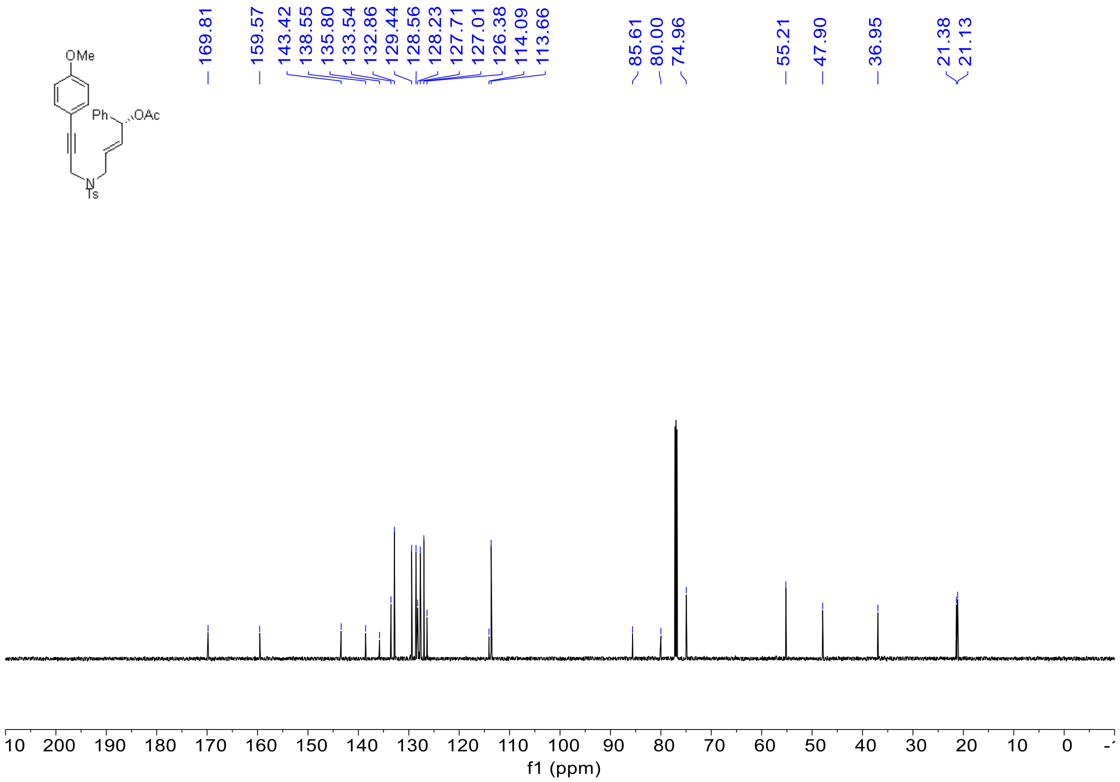

Supplementary Figure 176. ^{13}C NMR spectrum (151 MHz, $CDCl_3$) of **4c**

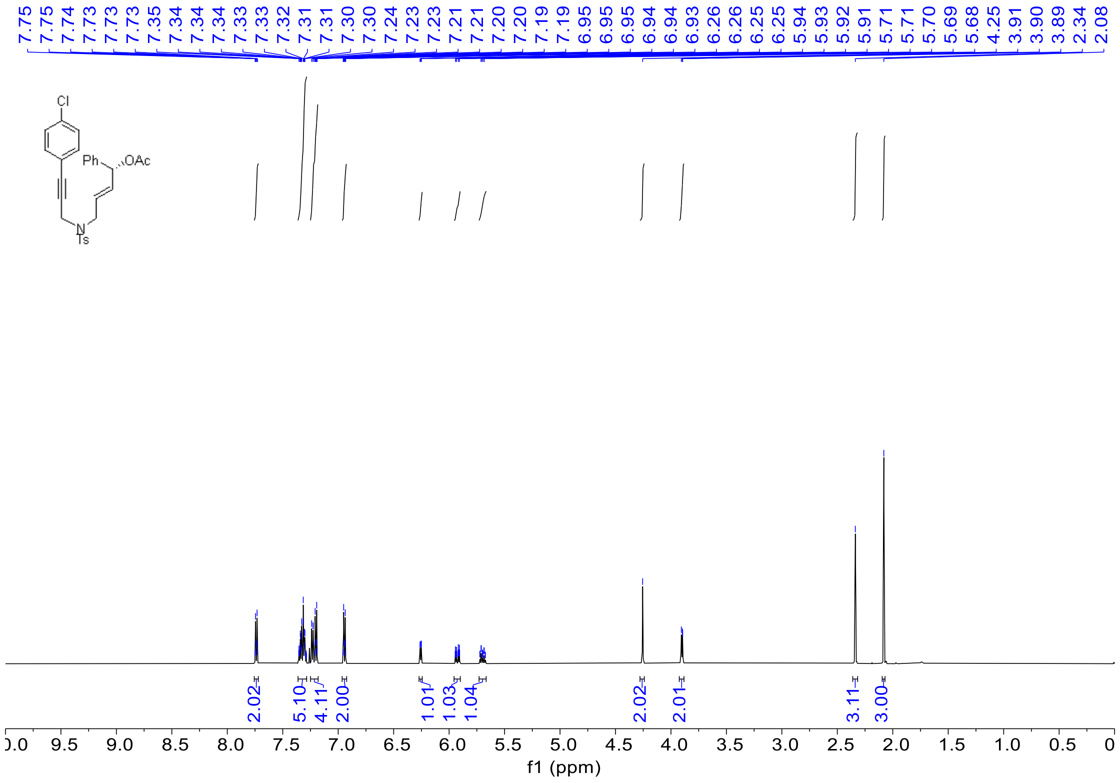

Supplementary Figure 177. ^{1}H NMR spectrum (600 MHz, $CDCl_3$) of **4d**

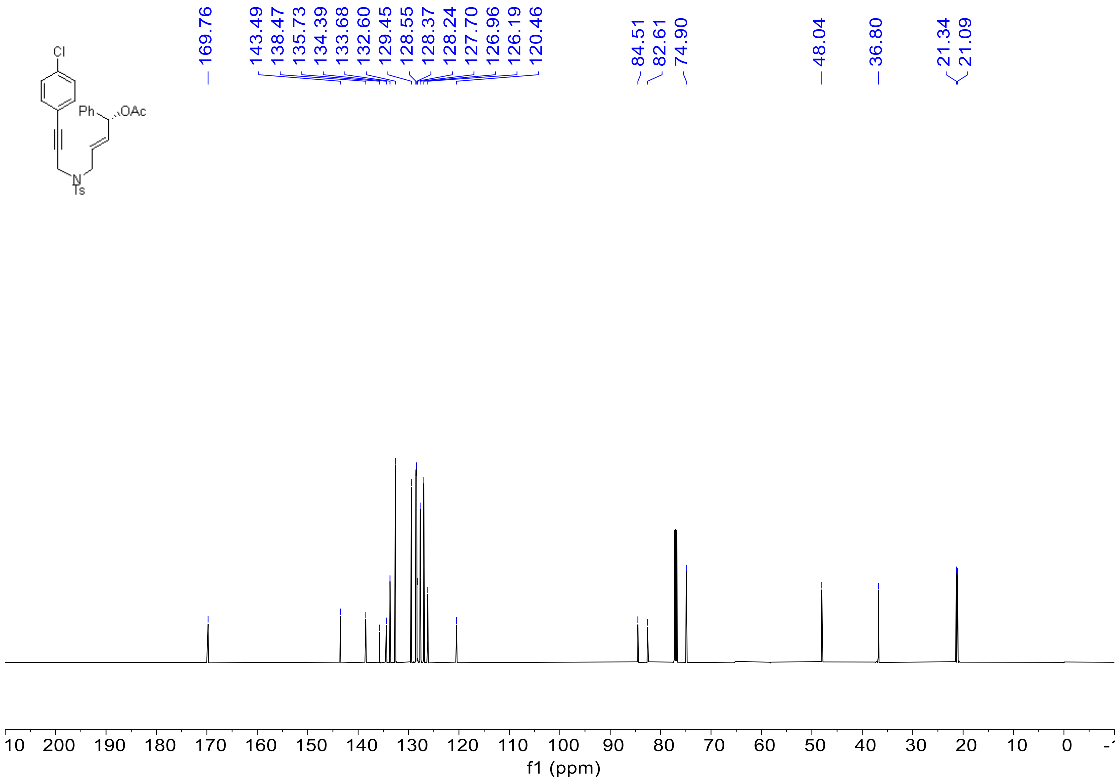

Supplementary Figure 178. ^{13}C NMR spectrum (151 MHz, $CDCl_3$) of **4d**

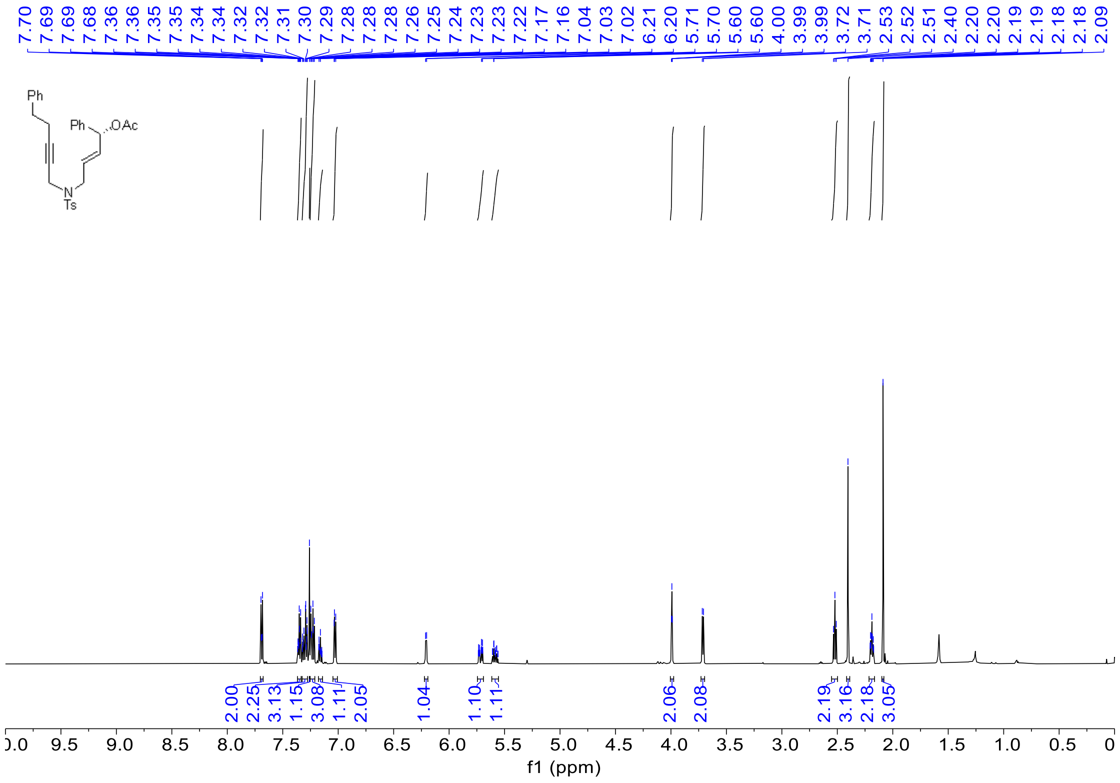

Supplementary Figure 179. ^{1}H NMR spectrum (600 MHz, $CDCl_3$) of **4e**

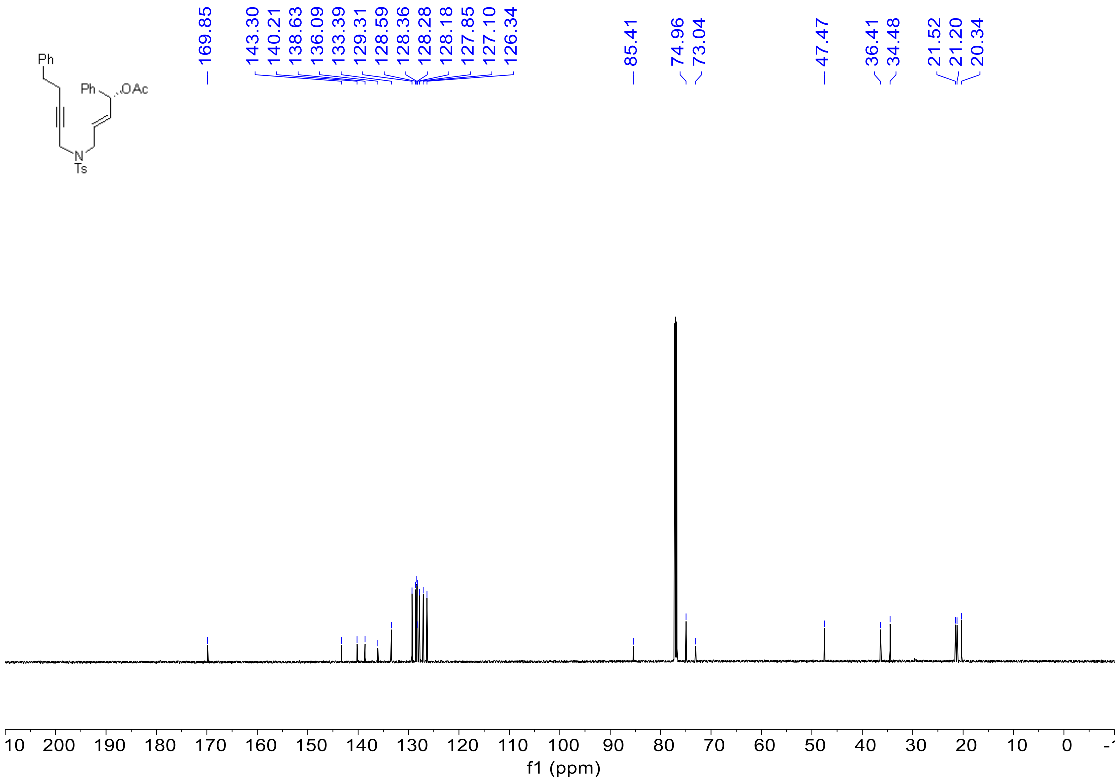

Supplementary Figure 180. ^{13}C NMR spectrum (151 MHz, $CDCl_3$) of **4e**

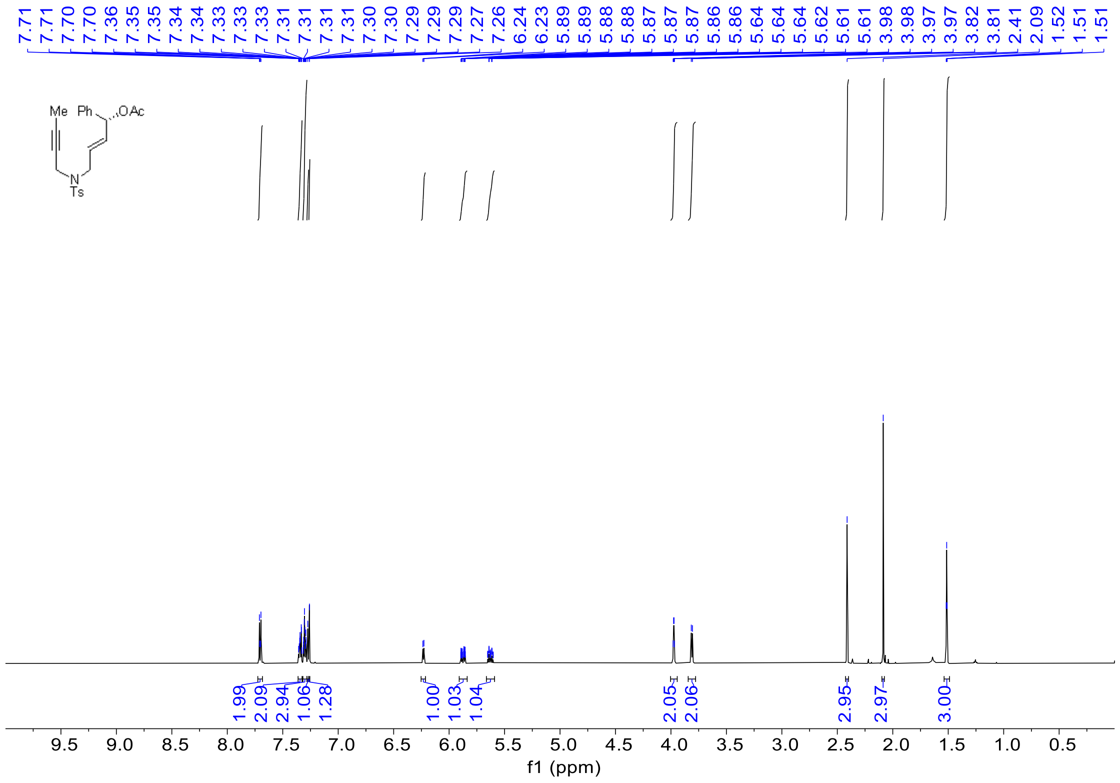

Supplementary Figure 181. ^{1}H NMR spectrum (600 MHz, $CDCl_3$) of **4f**

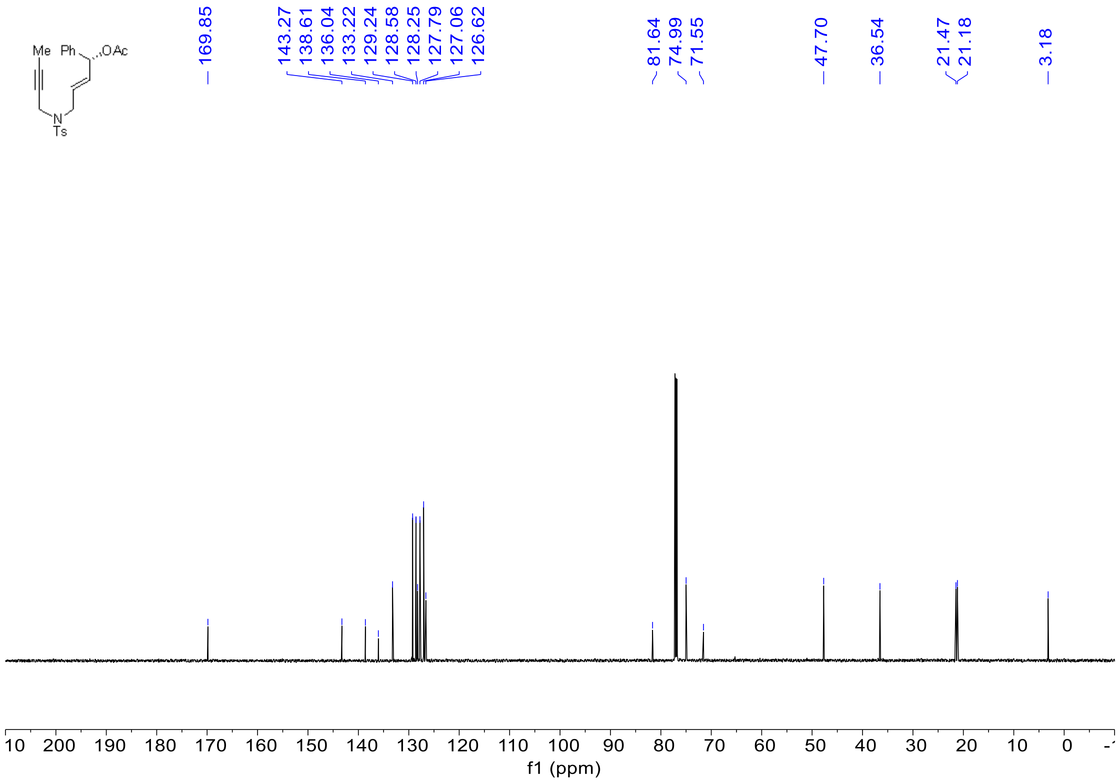

Supplementary Figure 182. ^{13}C NMR spectrum (151 MHz, $CDCl_3$) of **4f**

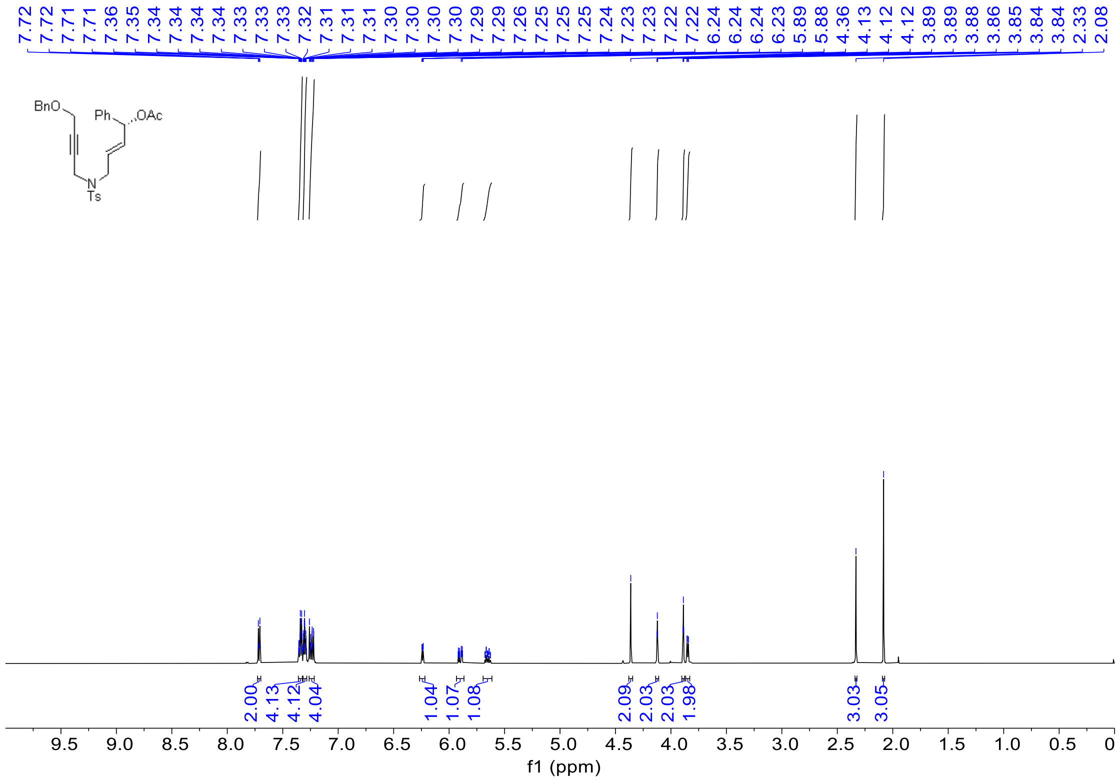

Supplementary Figure 183. ^{1}H NMR spectrum (600 MHz, $CDCl_3$) of **4g**

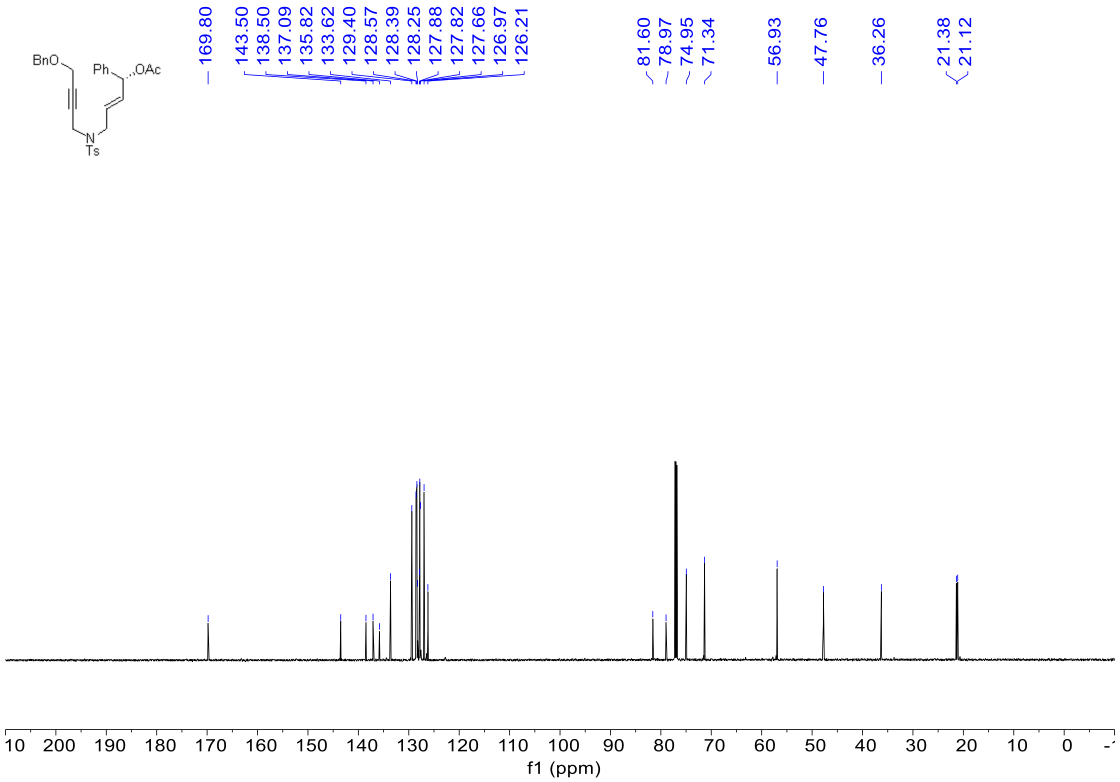

Supplementary Figure 184. ^{13}C NMR spectrum (151 MHz, $CDCl_3$) of **4g**

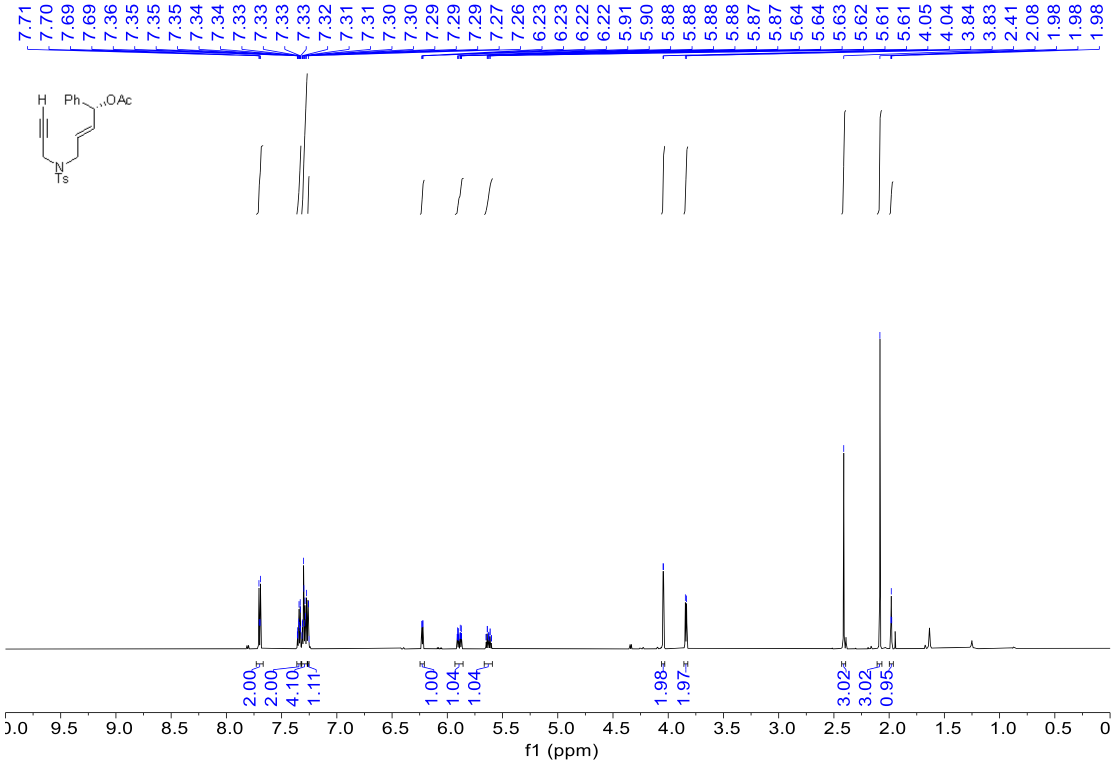

Supplementary Figure 185. ^{1}H NMR spectrum (600 MHz, $CDCl_3$) of **4h**

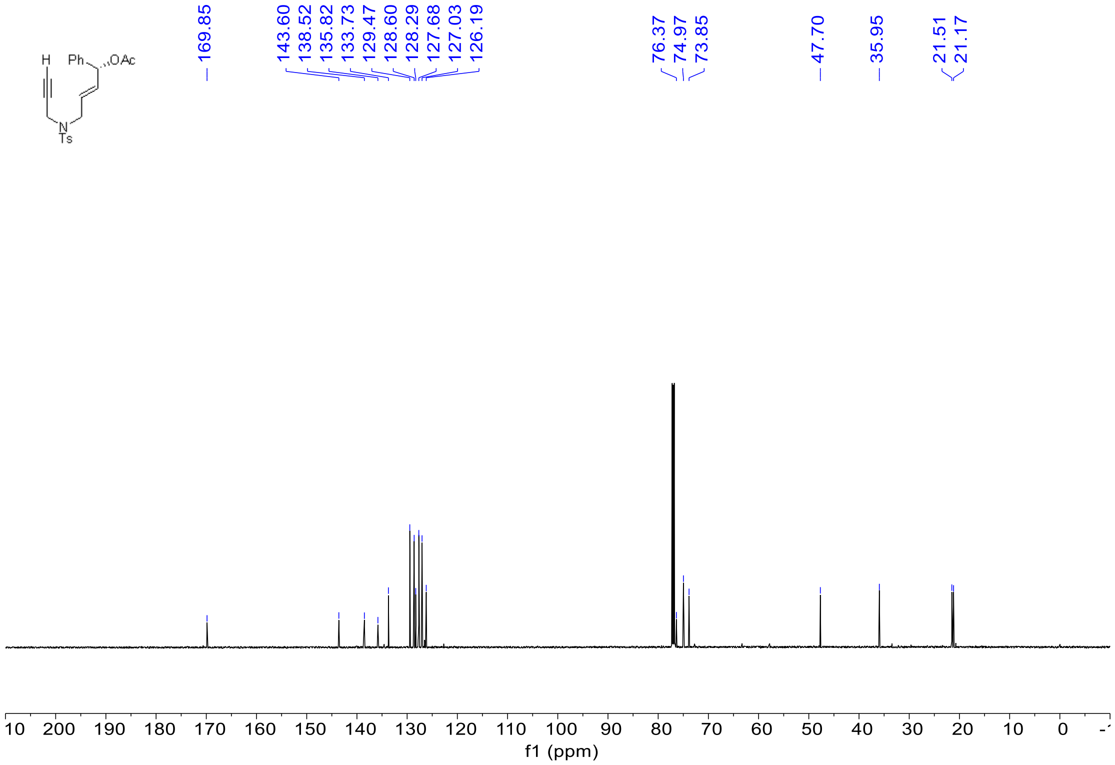

Supplementary Figure 186. ^{13}C NMR spectrum (151 MHz, $CDCl_3$) of **4h**

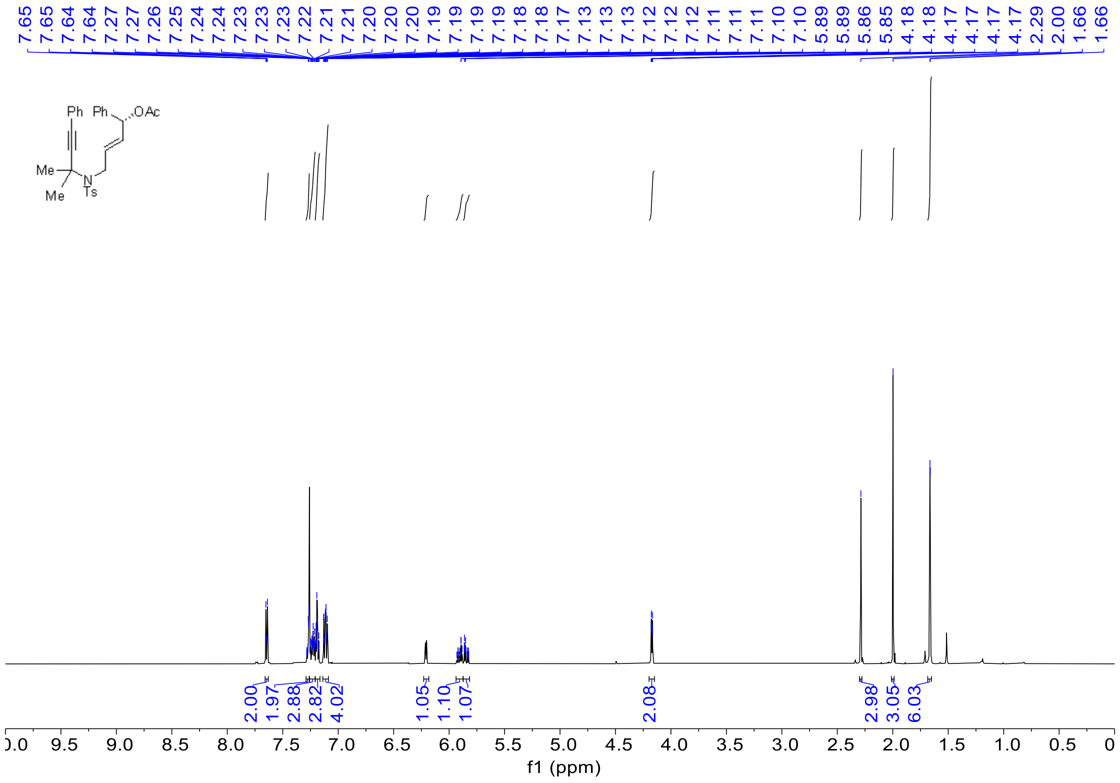

Supplementary Figure 187. ^{1}H NMR spectrum (600 MHz, $CDCl_3$) of **4i**

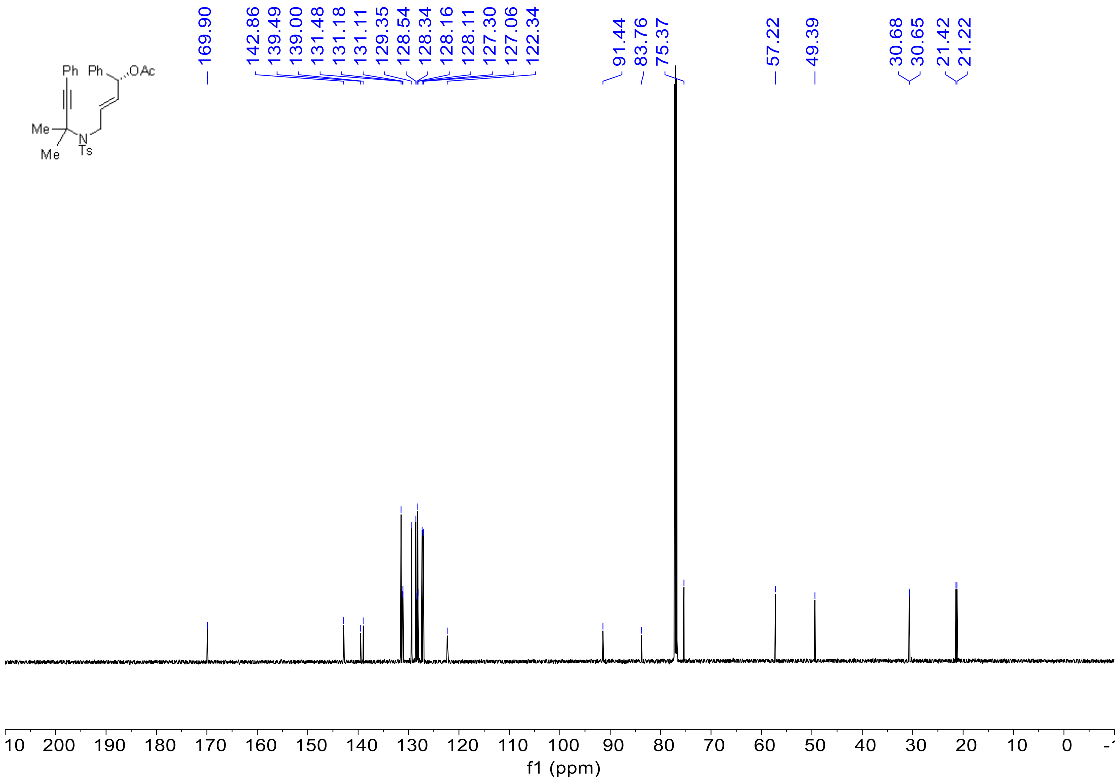

Supplementary Figure 188. ^{13}C NMR spectrum (151 MHz, $CDCl_3$) of **4i**

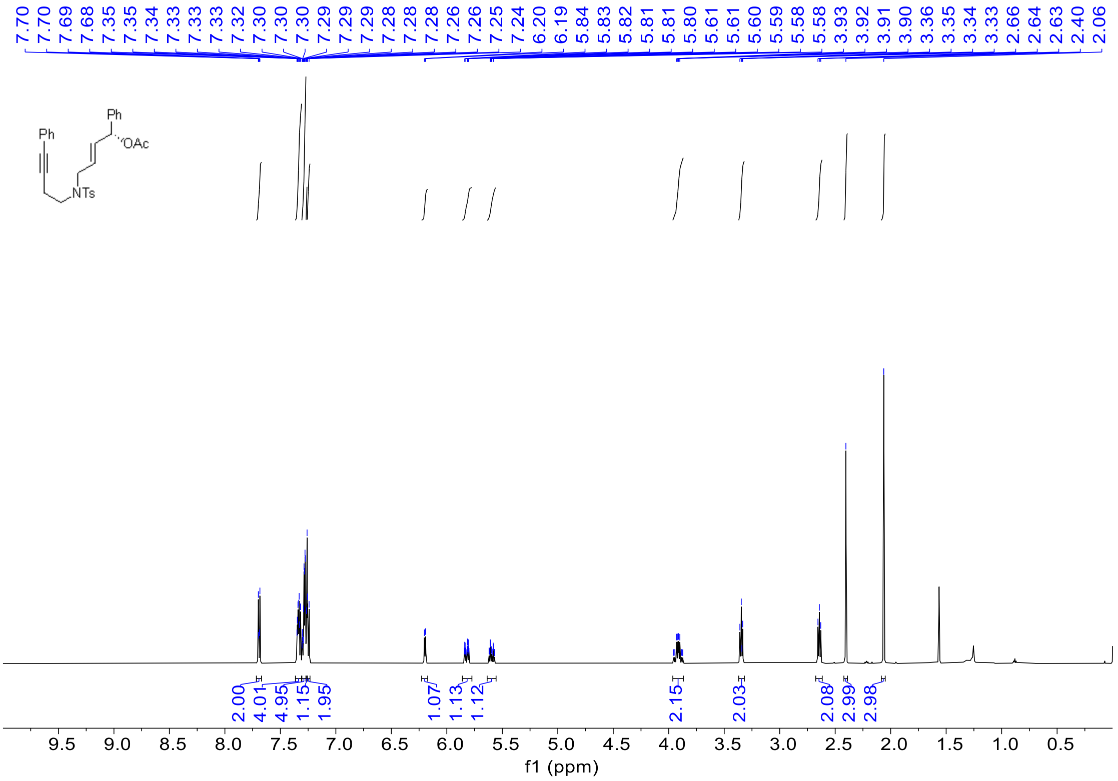

Supplementary Figure 189. ^{1}H NMR spectrum (600 MHz, $CDCl_3$) of **4j**

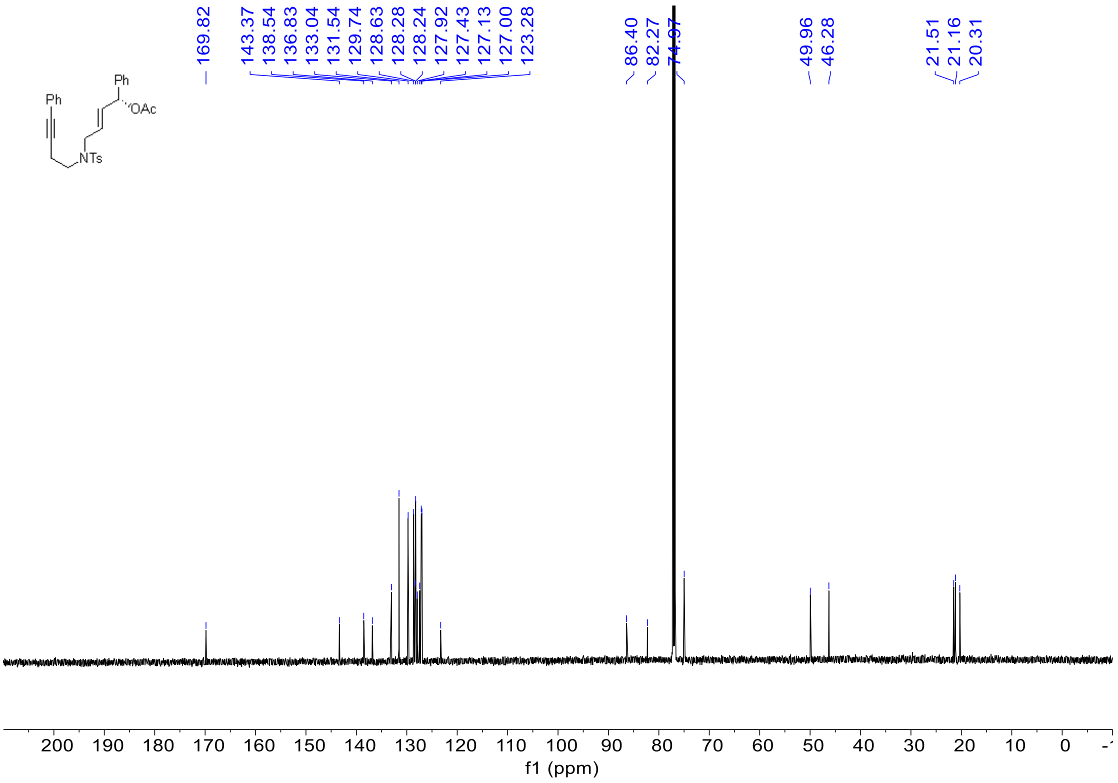

Supplementary Figure 190. ^{13}C NMR spectrum (151 MHz, $CDCl_3$) of **4j**

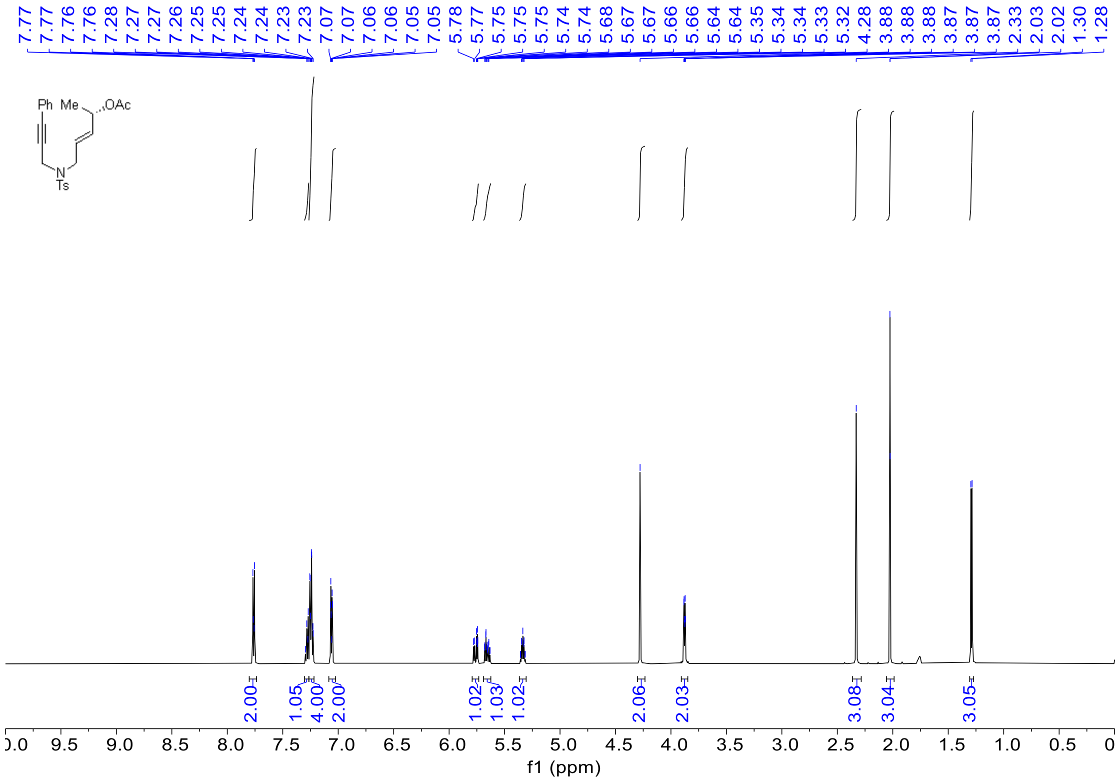

Supplementary Figure 191. ^{1}H NMR spectrum (600 MHz, $CDCl_3$) of **4k**

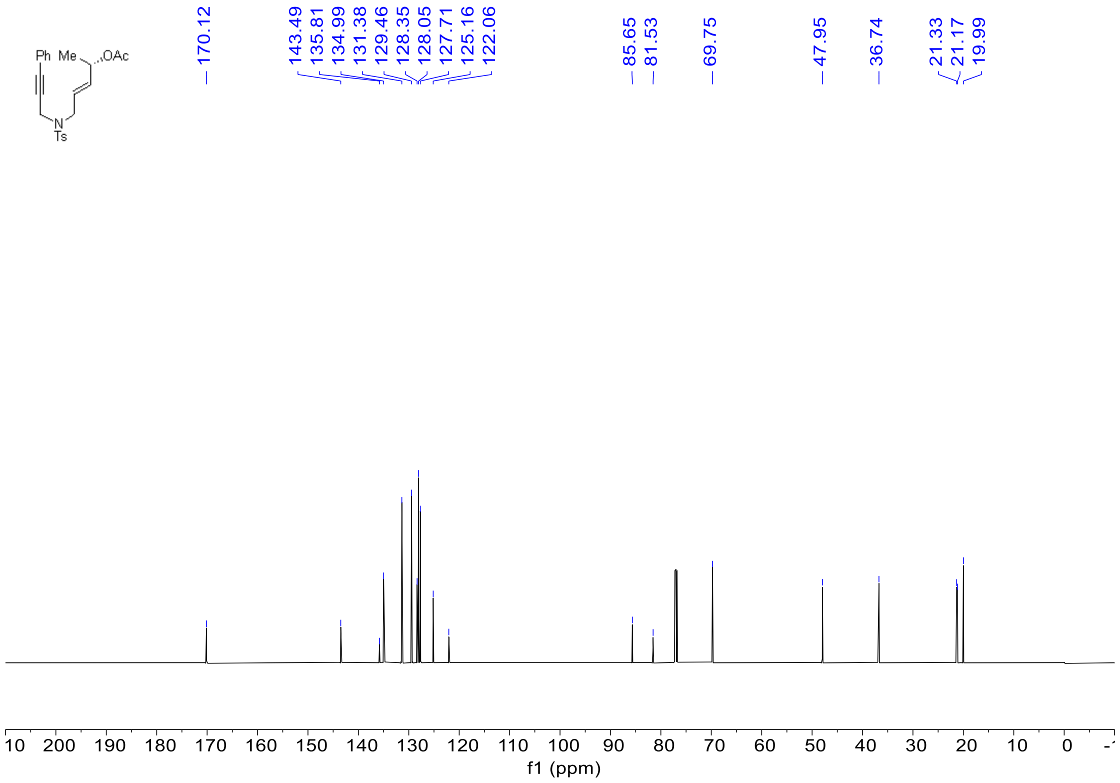

Supplementary Figure 192. ^{13}C NMR spectrum (151 MHz, $CDCl_3$) of **4k**

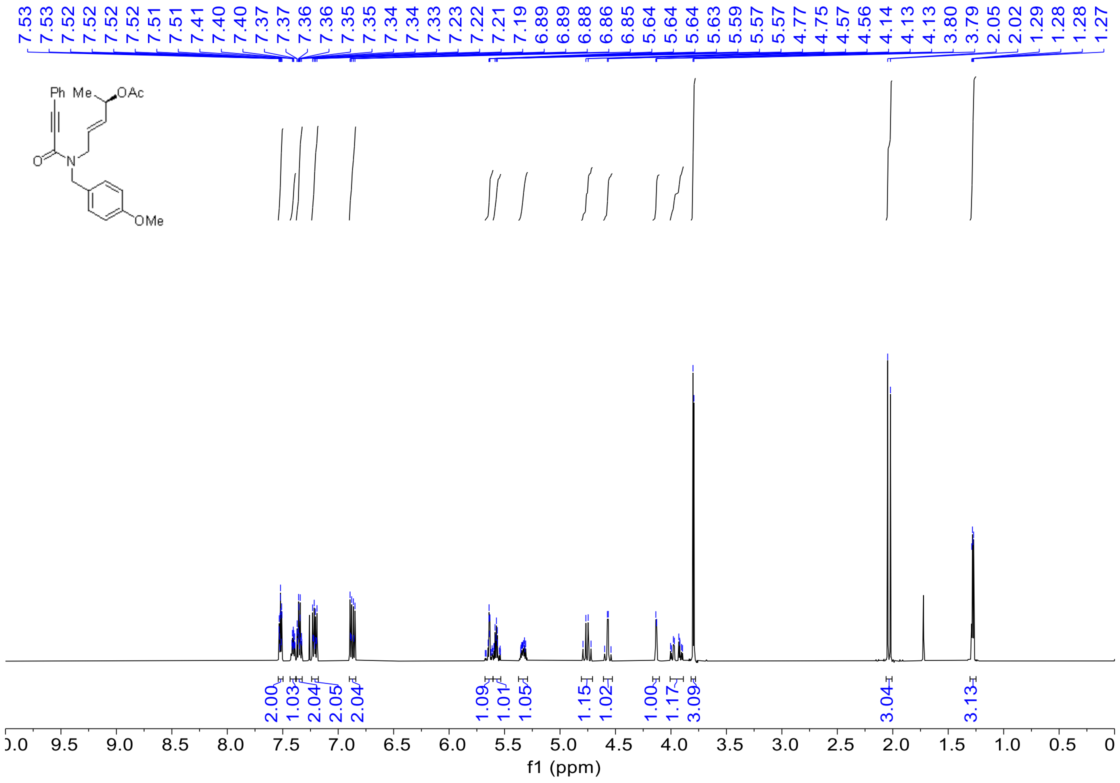

Supplementary Figure 193. ^{1}H NMR spectrum (600 MHz, $CDCl_3$) of **4l**

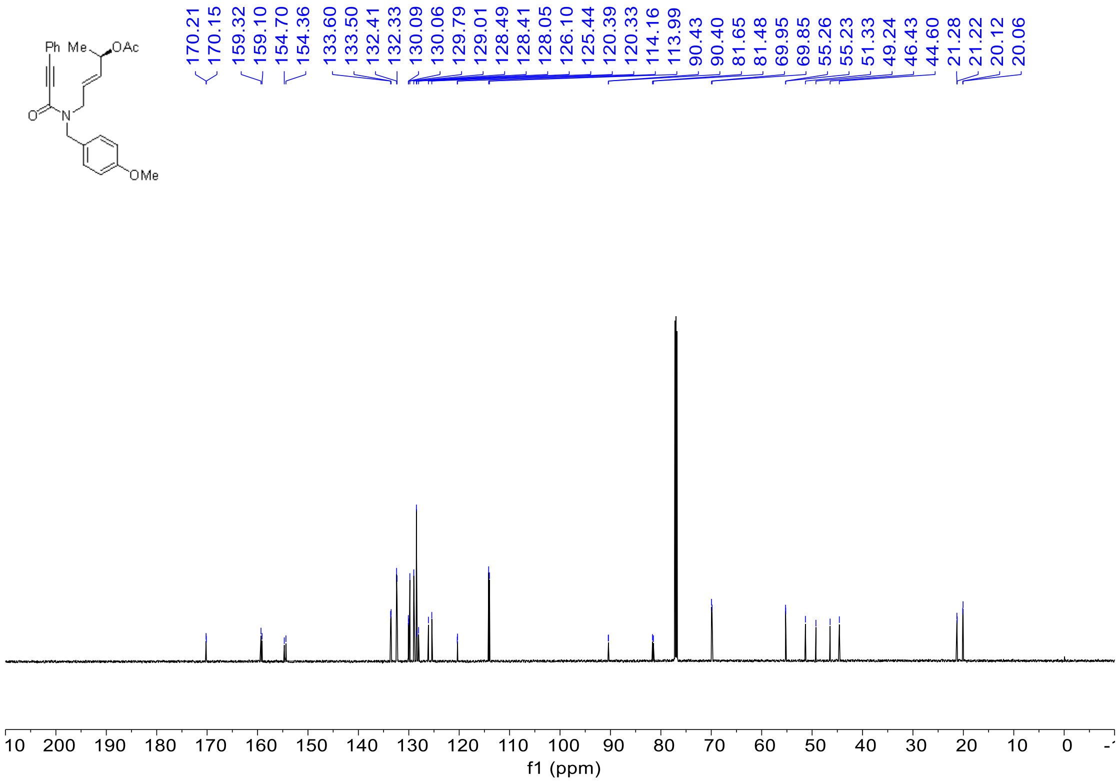

Supplementary Figure 194. ^{13}C NMR spectrum (151 MHz, $CDCl_3$) of **4l**

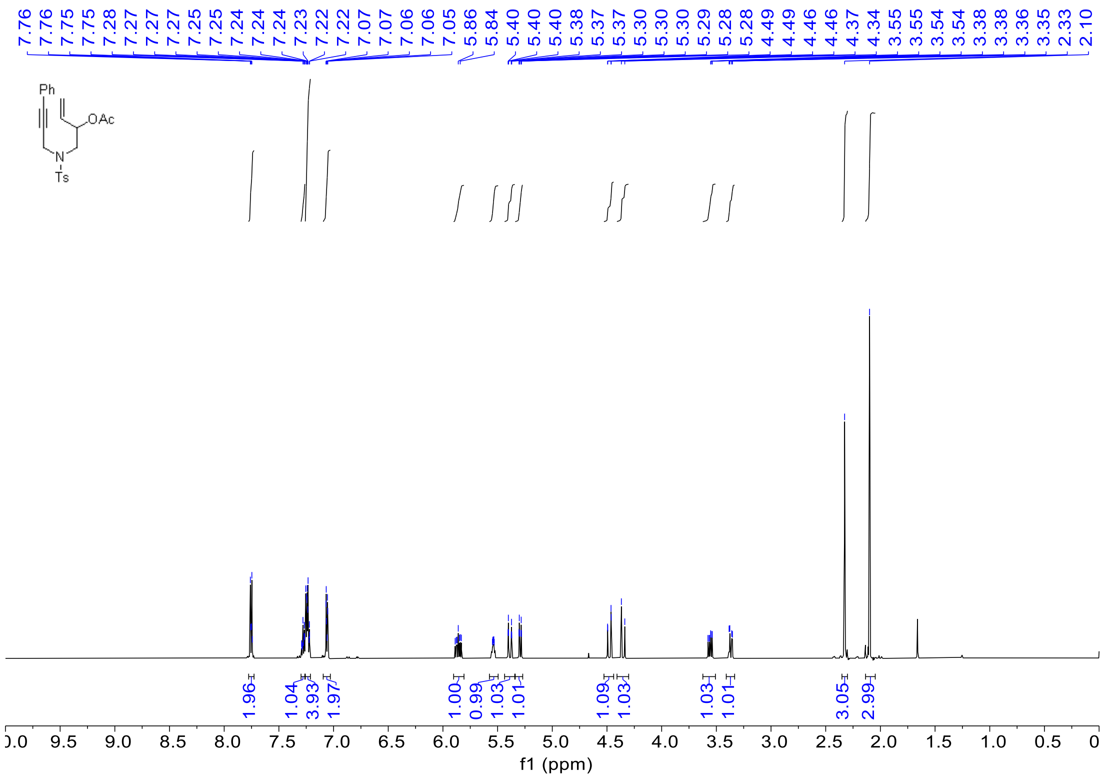

Supplementary Figure 195. ^{1}H NMR spectrum (400 MHz, $CDCl_3$) of **1o**

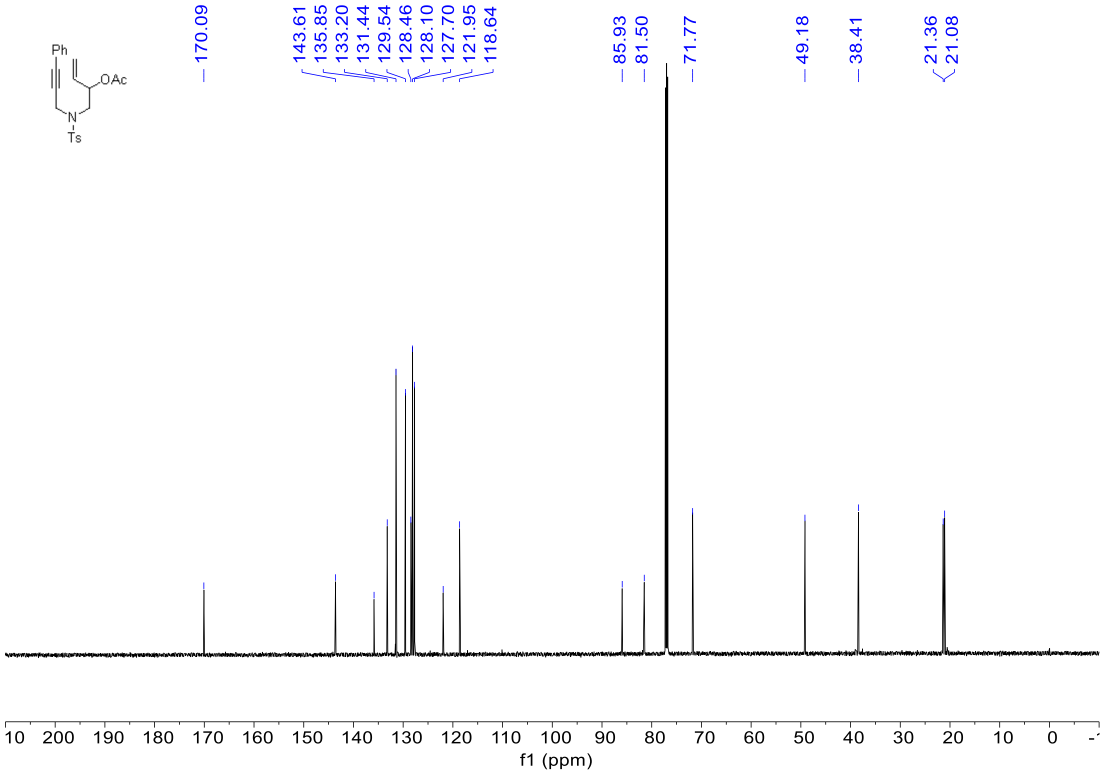

Supplementary Figure 196. ^{13}C NMR spectrum (100 MHz, $CDCl_3$) of **1o**

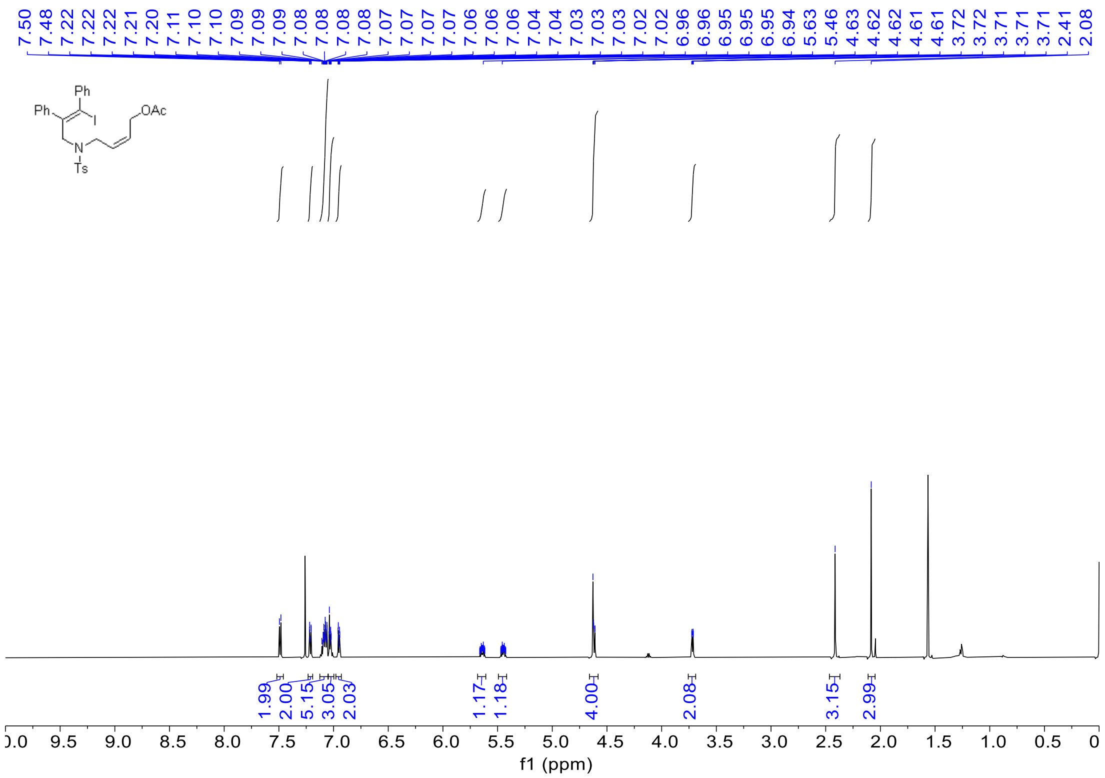

Supplementary Figure 197. ^{1}H NMR spectrum (600 MHz, $CDCl_3$) of **14**

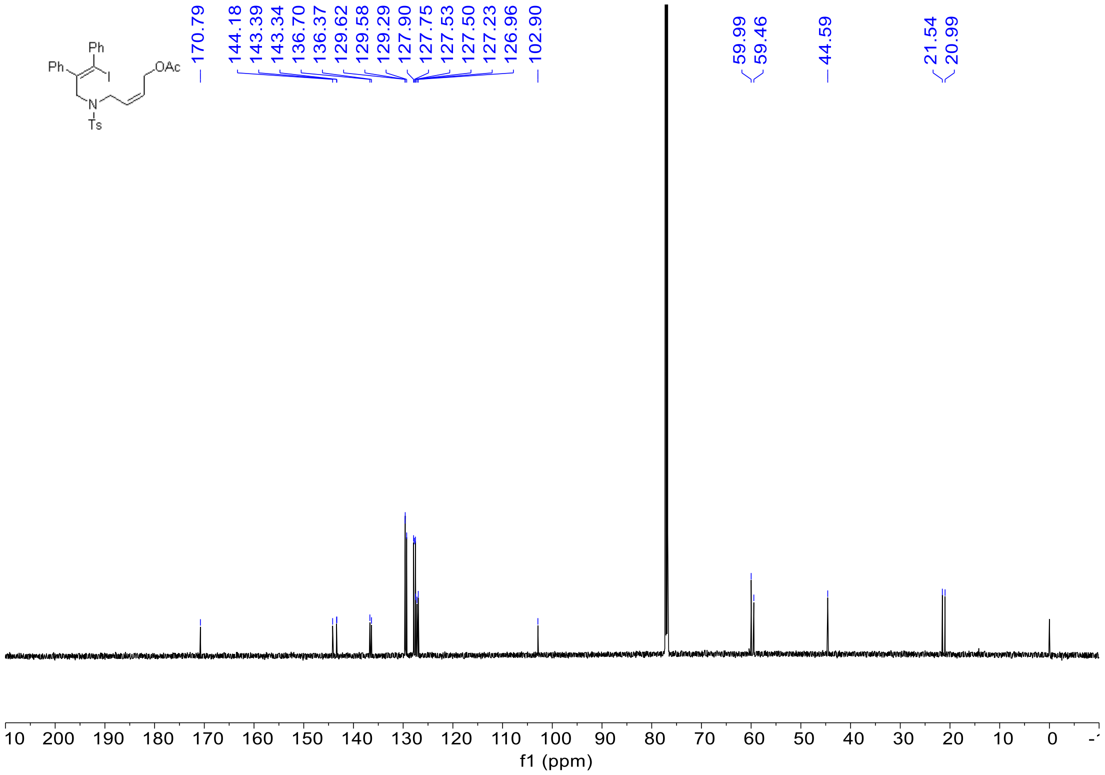

Supplementary Figure 198. ^{13}C NMR spectrum (151 MHz, $CDCl_3$) of **14**

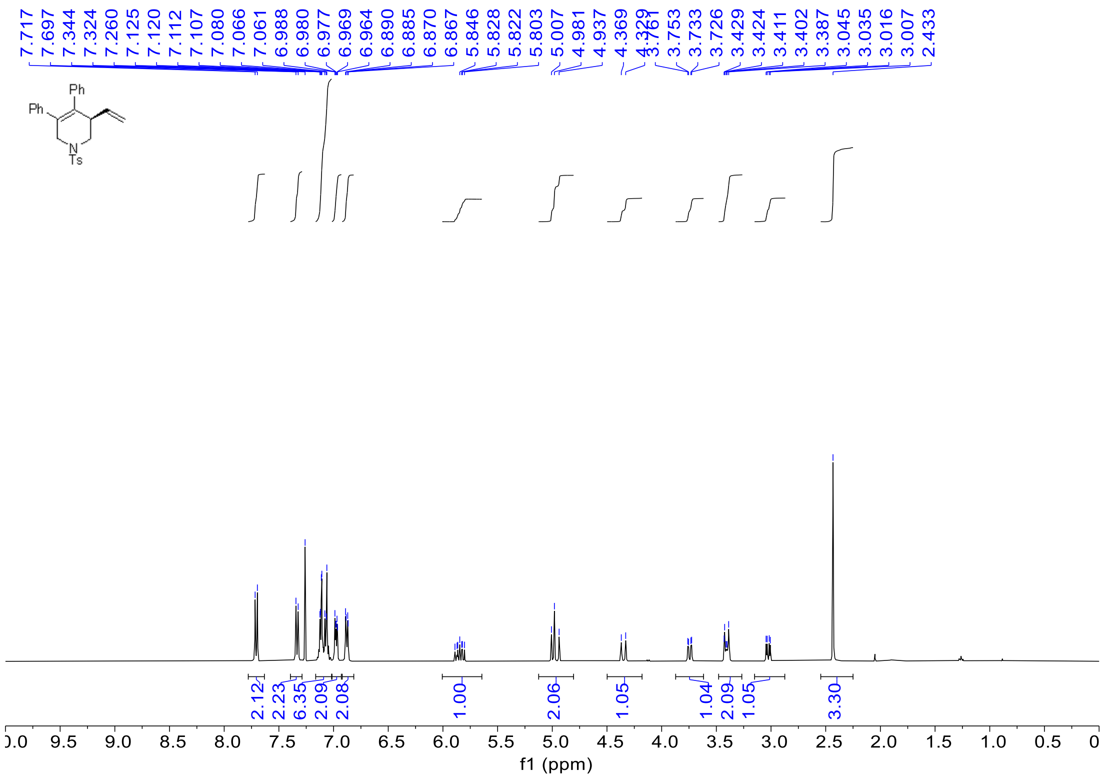

Supplementary Figure 199. ^{1}H NMR spectrum (400 MHz, $CDCl_3$) of **3aa**

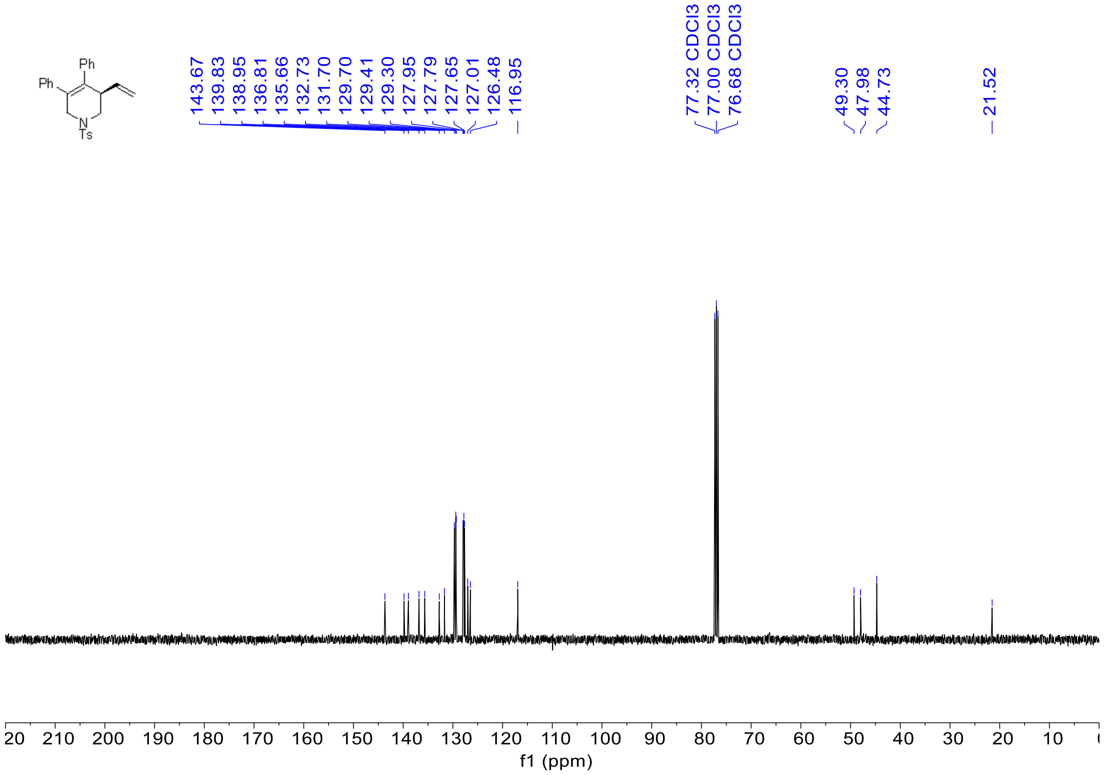

Supplementary Figure 200. ^{13}C NMR spectrum (100 MHz, $CDCl_3$) of **3aa**

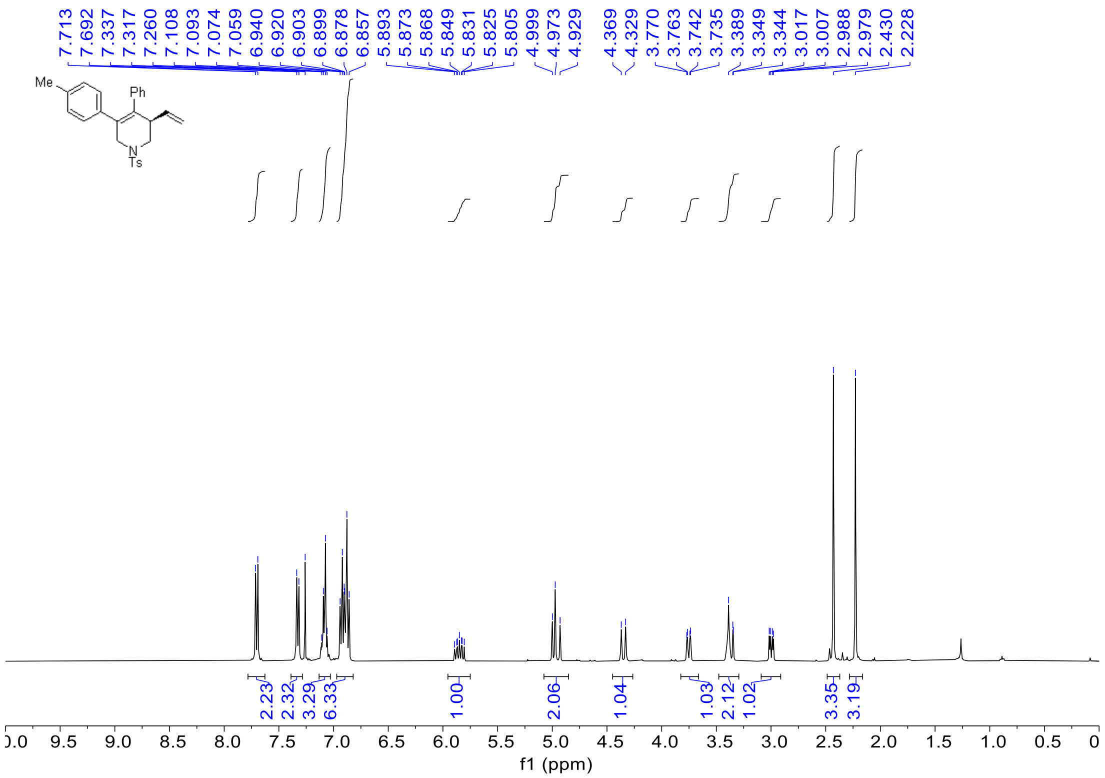

Supplementary Figure 201. ^{1}H NMR spectrum (400 MHz, $CDCl_3$) of **3ab**

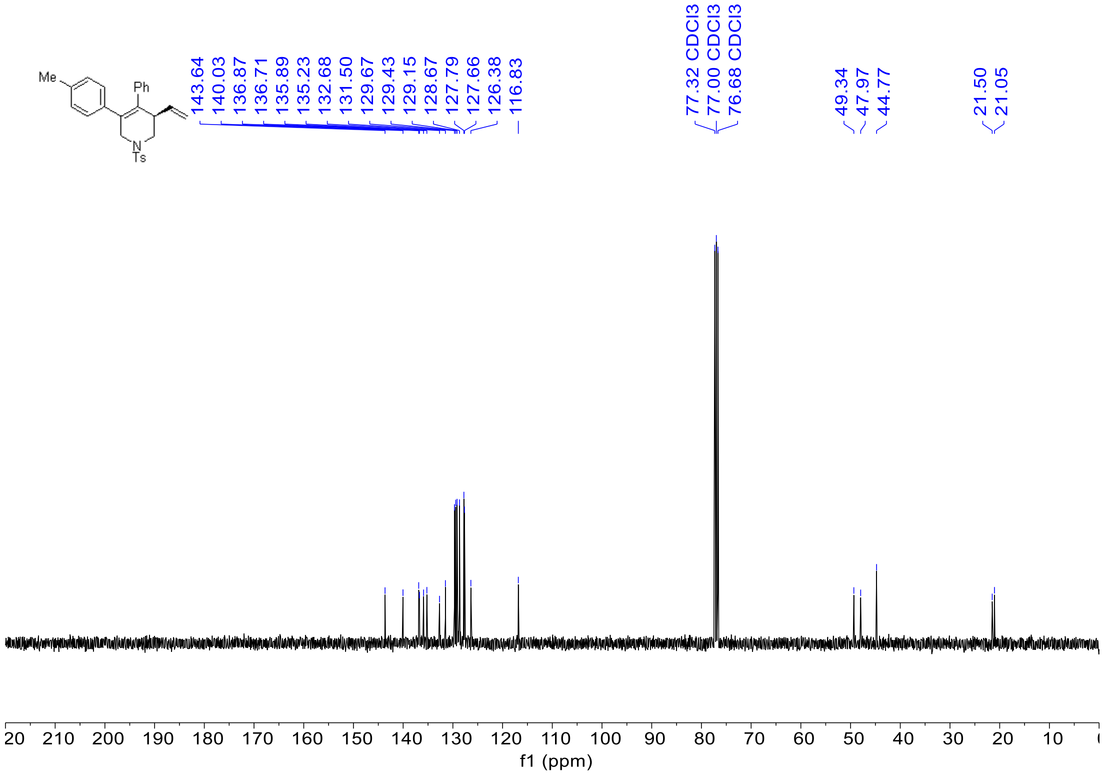

Supplementary Figure 202. ^{13}C NMR spectrum (100 MHz, $CDCl_3$) of **3ab**

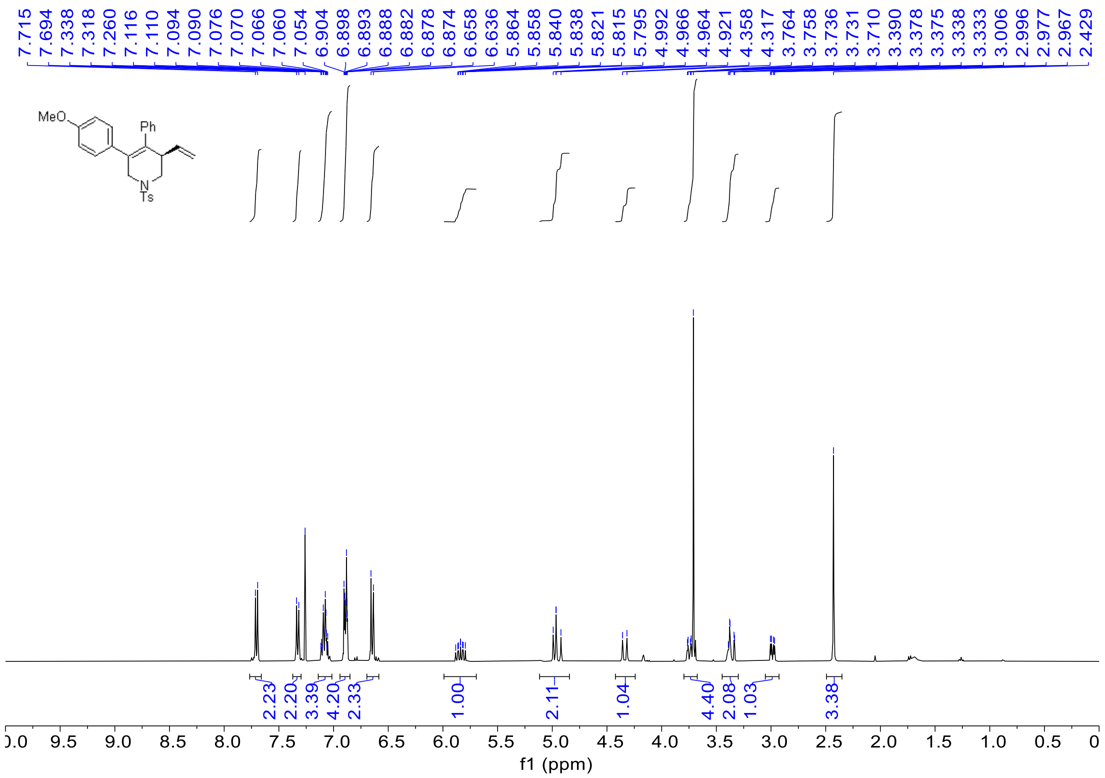

Supplementary Figure 203. ^{1}H NMR spectrum (400 MHz, $CDCl_3$) of **3ac**

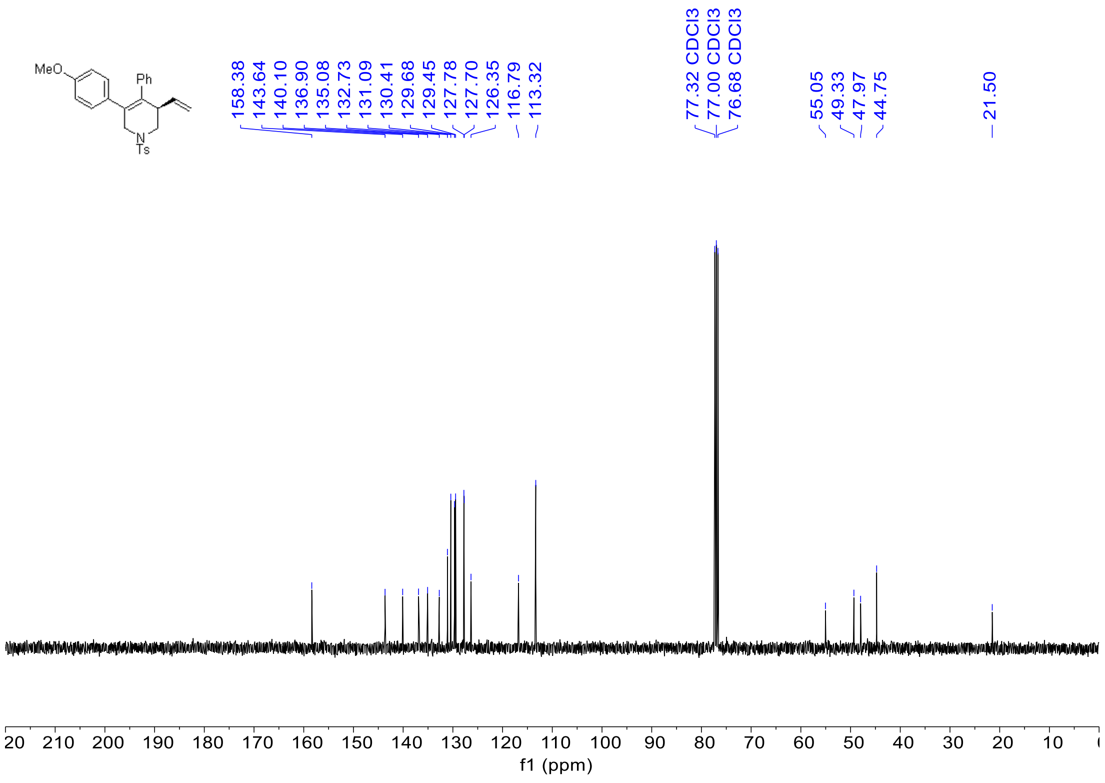

Supplementary Figure 204. ^{13}C NMR spectrum (100 MHz, $CDCl_3$) of **3ac**

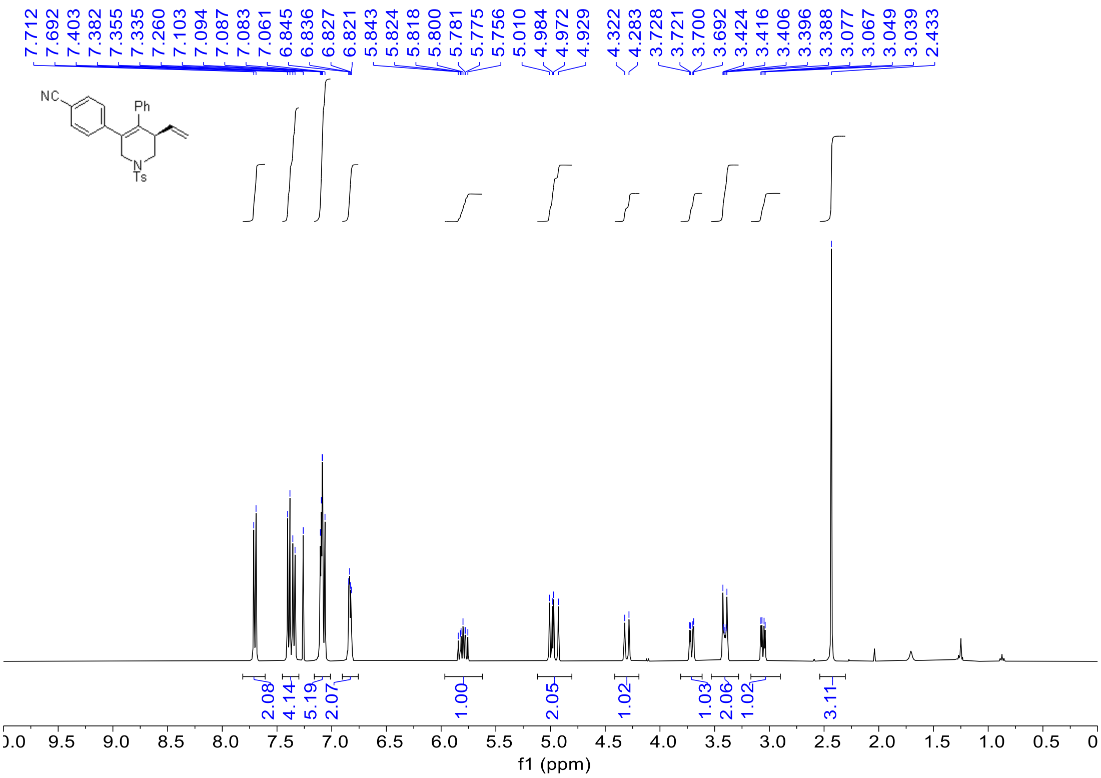

Supplementary Figure 205. ^{1}H NMR spectrum (400 MHz, $CDCl_3$) of **3ad**

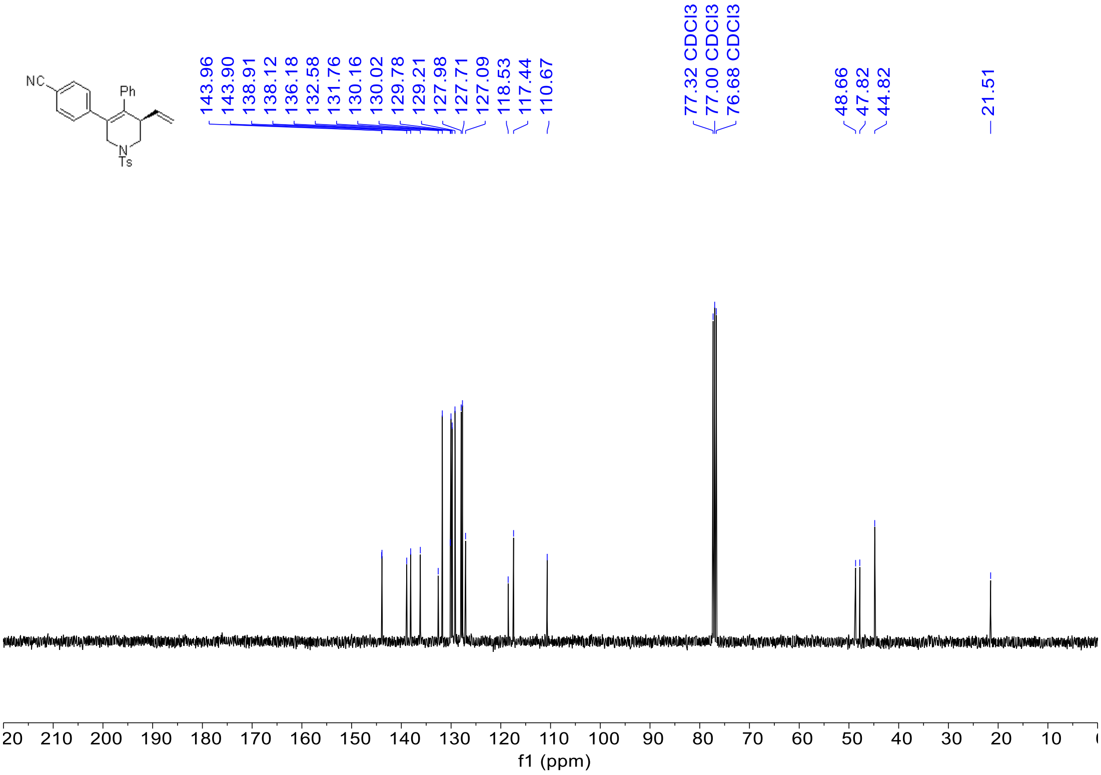

Supplementary Figure 206. ^{13}C NMR spectrum (100 MHz, $CDCl_3$) of **3ad**

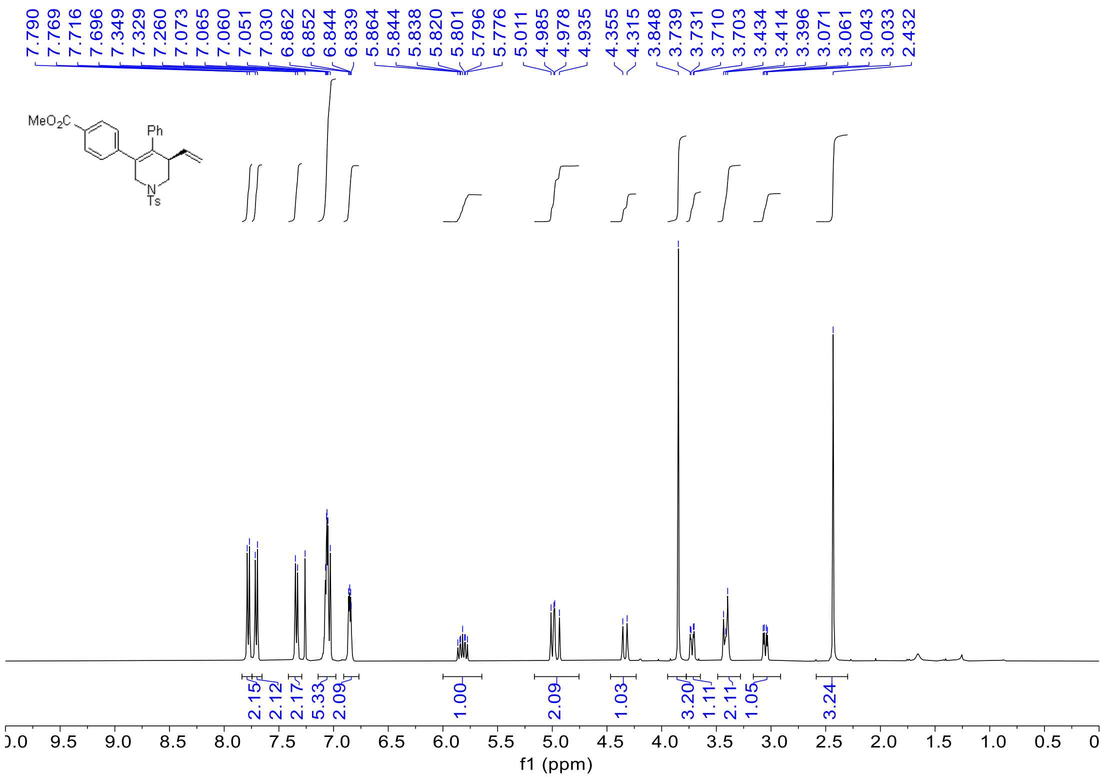

Supplementary Figure 207. ^{1}H NMR spectrum (400 MHz, $CDCl_3$) of **3ae**

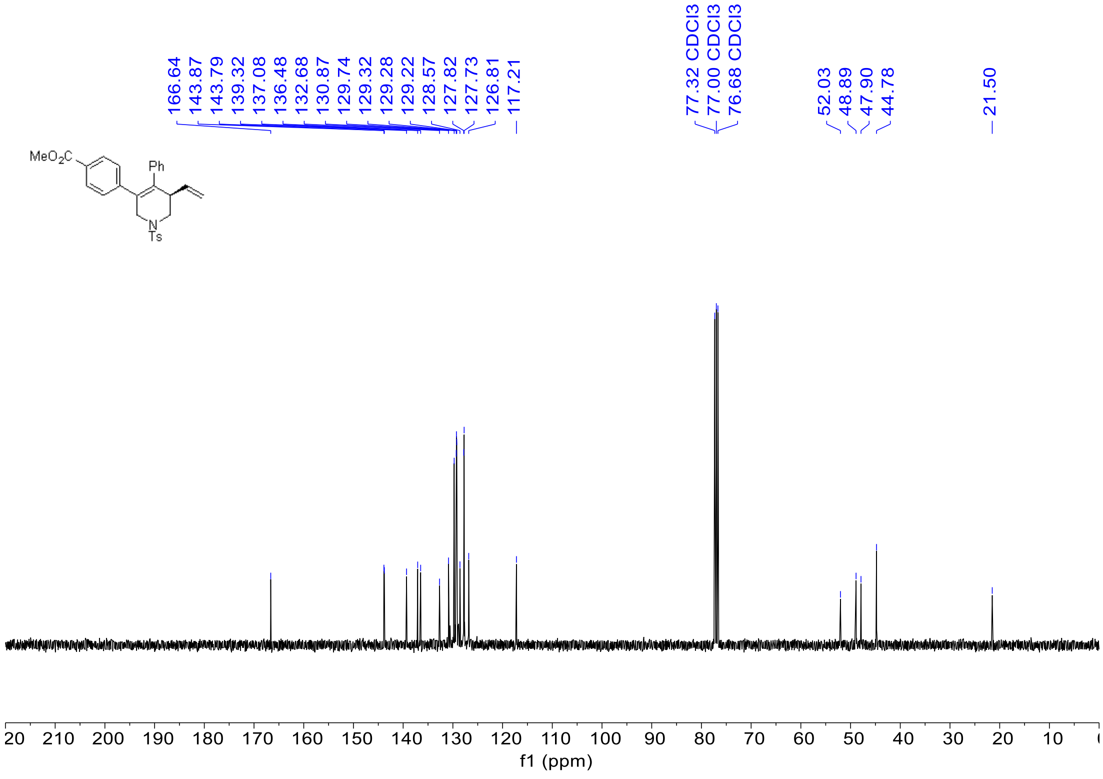

Supplementary Figure 208. ^{13}C NMR spectrum (100 MHz, $CDCl_3$) of **3ae**

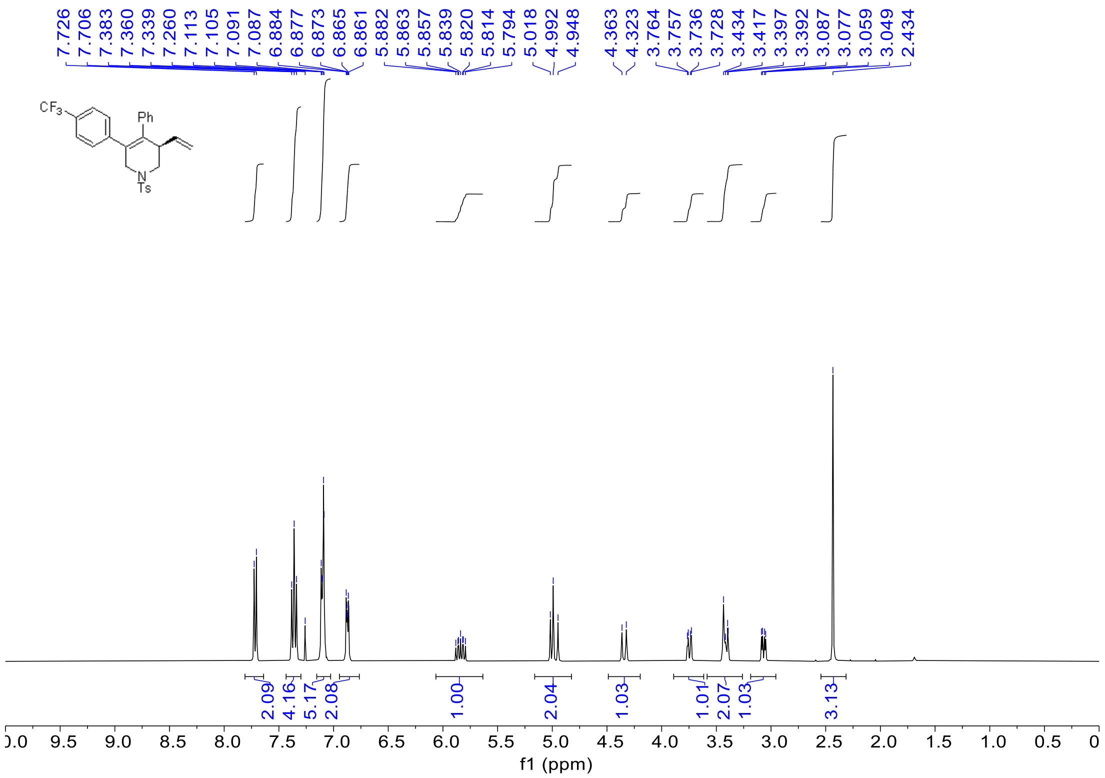

Supplementary Figure 209. ^{1}H NMR spectrum (400 MHz, $CDCl_3$) of **3af**

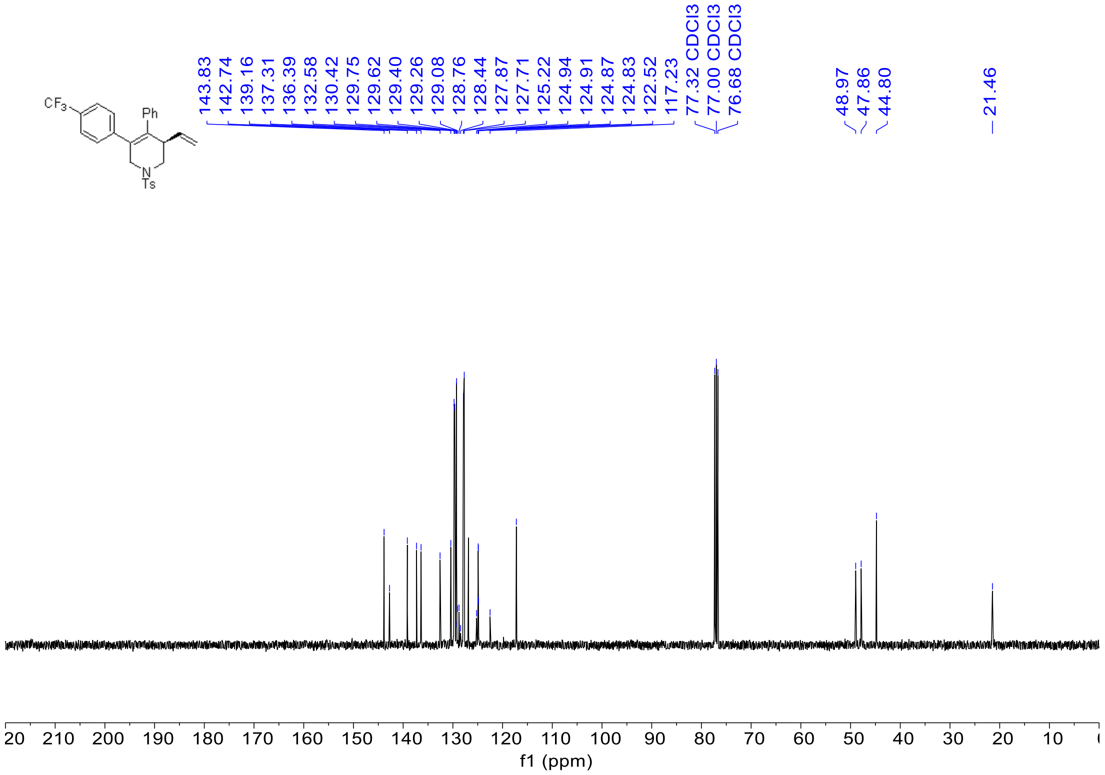

Supplementary Figure 210. ^{13}C NMR spectrum (100 MHz, $CDCl_3$) of **3af**

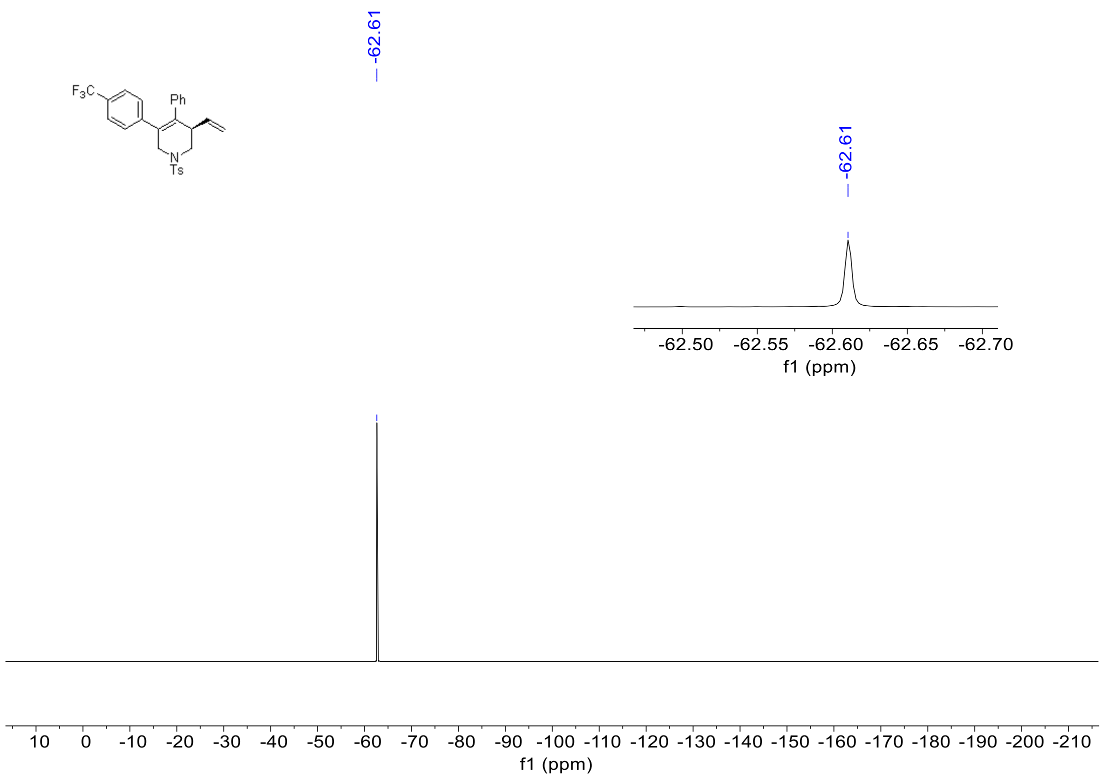

Supplementary Figure 211. ^{19}F NMR spectrum (565 MHz, $CDCl_3$) of **3af**

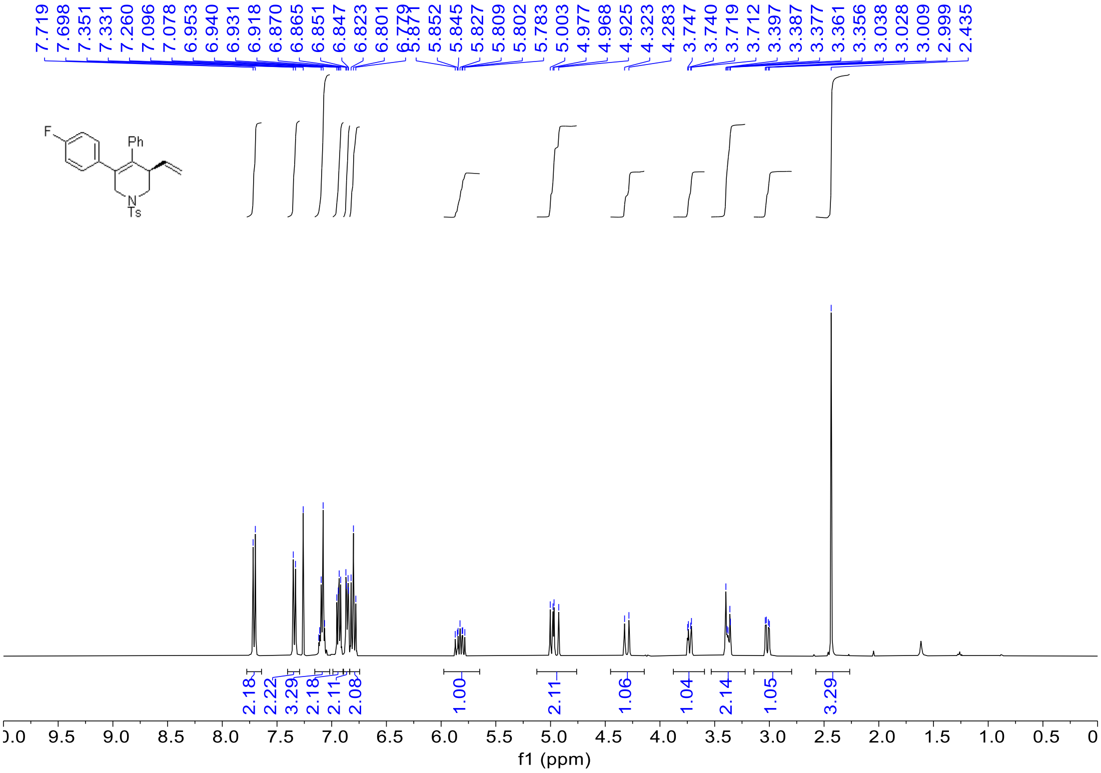

Supplementary Figure 212. ^{1}H NMR spectrum (400 MHz, $CDCl_3$) of **3ag**

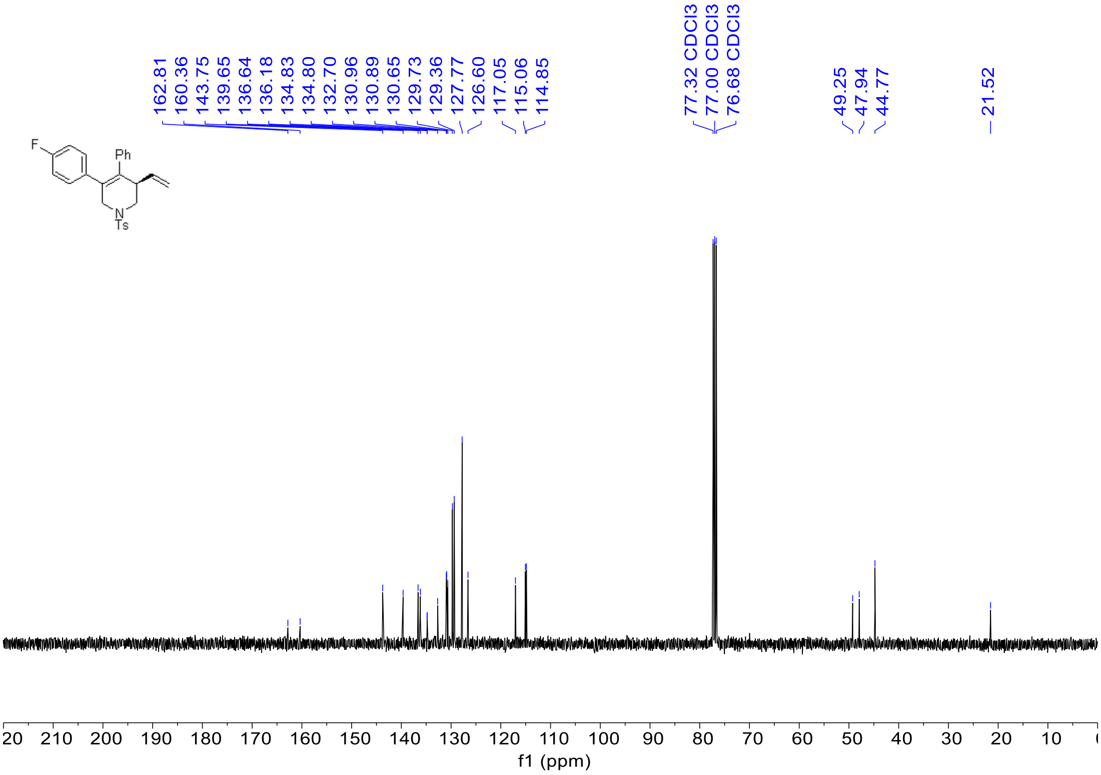

Supplementary Figure 213. ^{13}C NMR spectrum (100 MHz, $CDCl_3$) of **3ag**

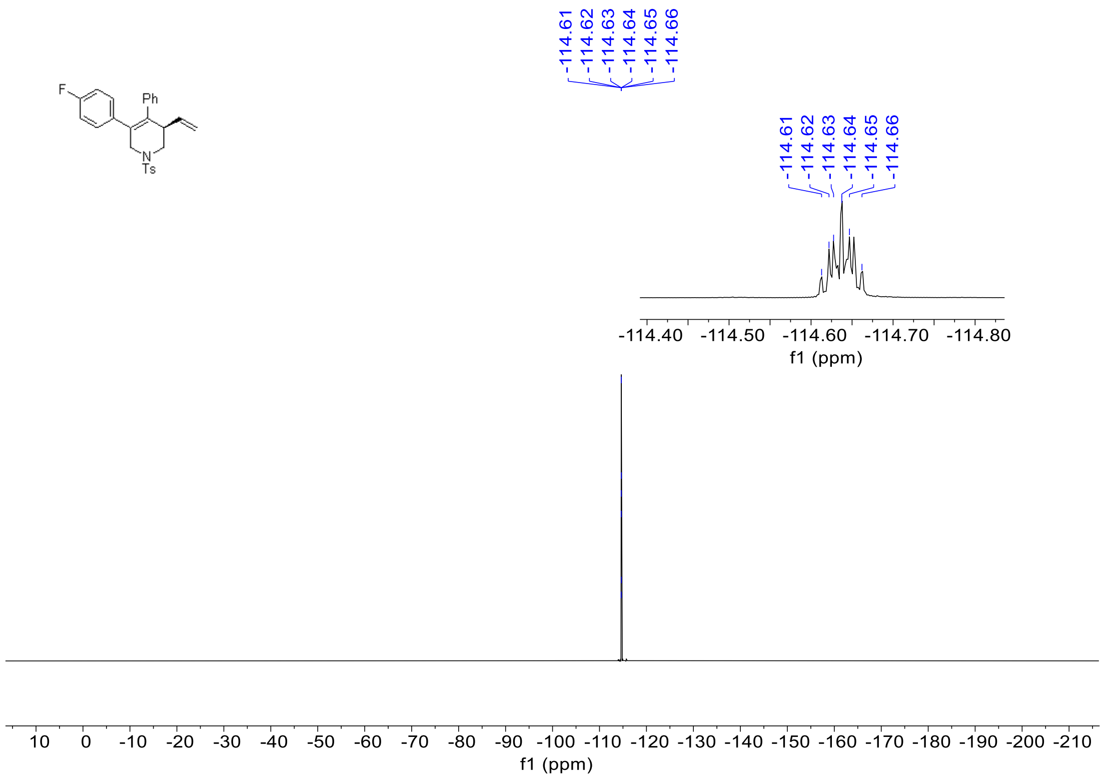

Supplementary Figure 214. ^{19}F NMR spectrum (565 MHz, $CDCl_3$) of **3ag**

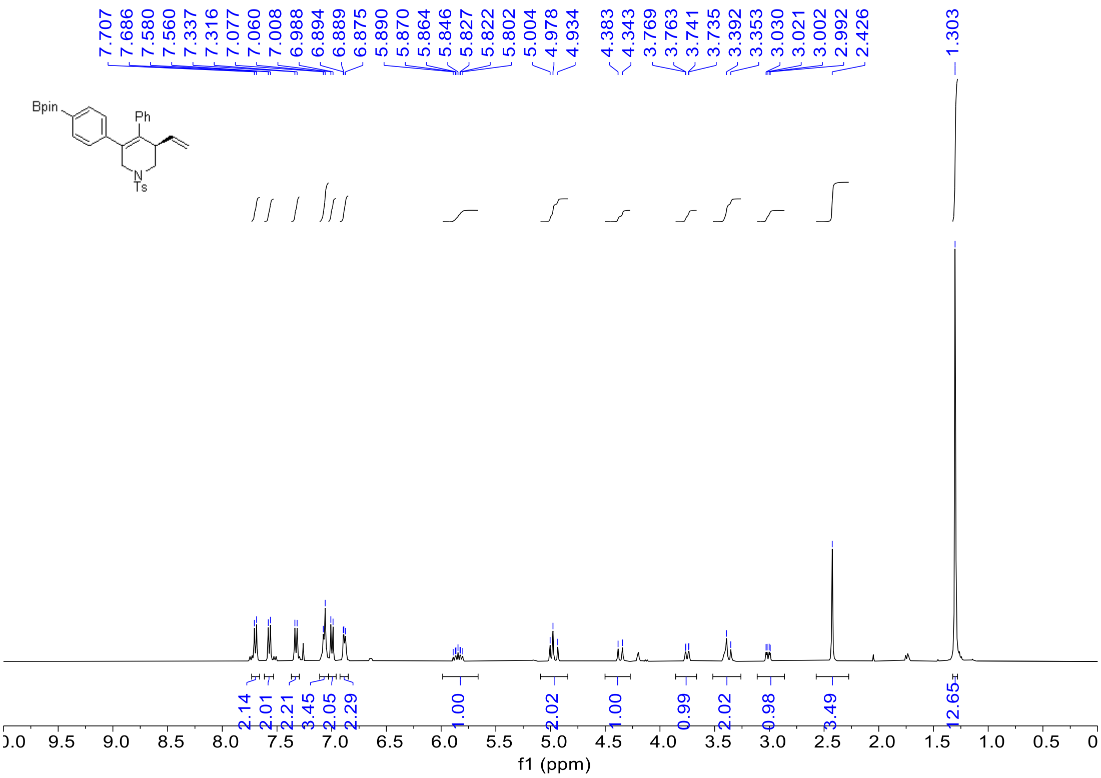

Supplementary Figure 215. ^{1}H NMR spectrum (400 MHz, $CDCl_3$) of **3ah**

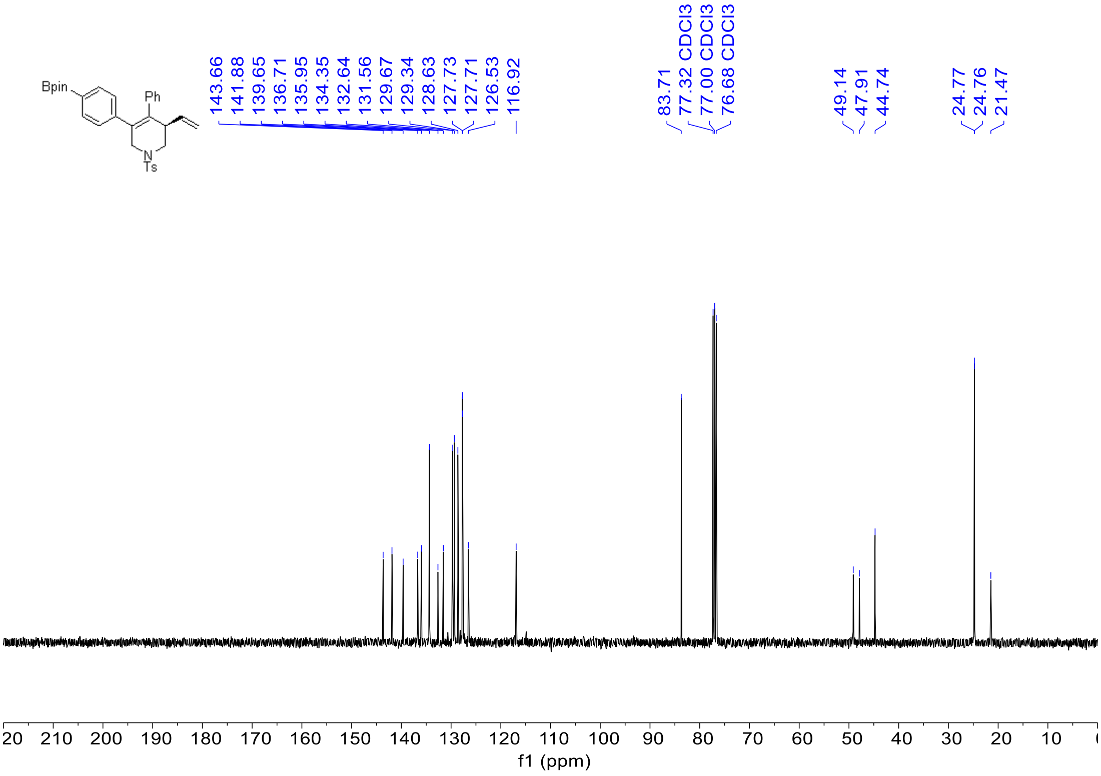

Supplementary Figure 216. ^{13}C NMR spectrum (100 MHz, $CDCl_3$) of **3ah**

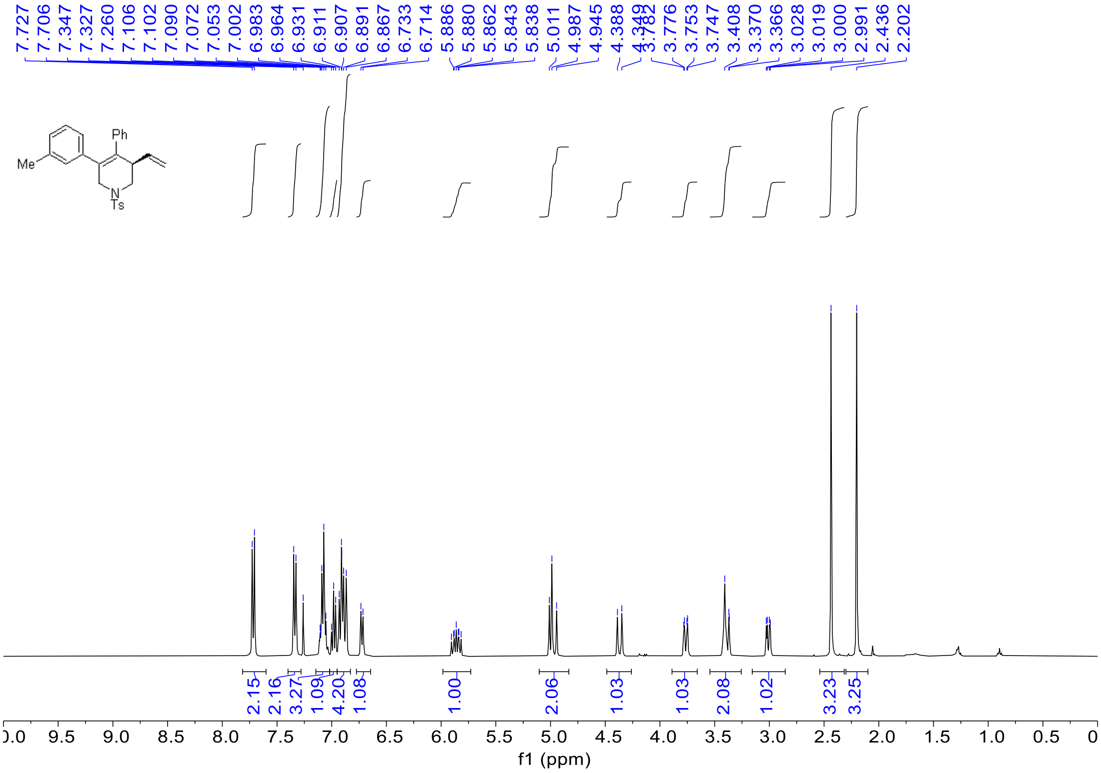

Supplementary Figure 217. ^{1}H NMR spectrum (400 MHz, $CDCl_3$) of **3ai**

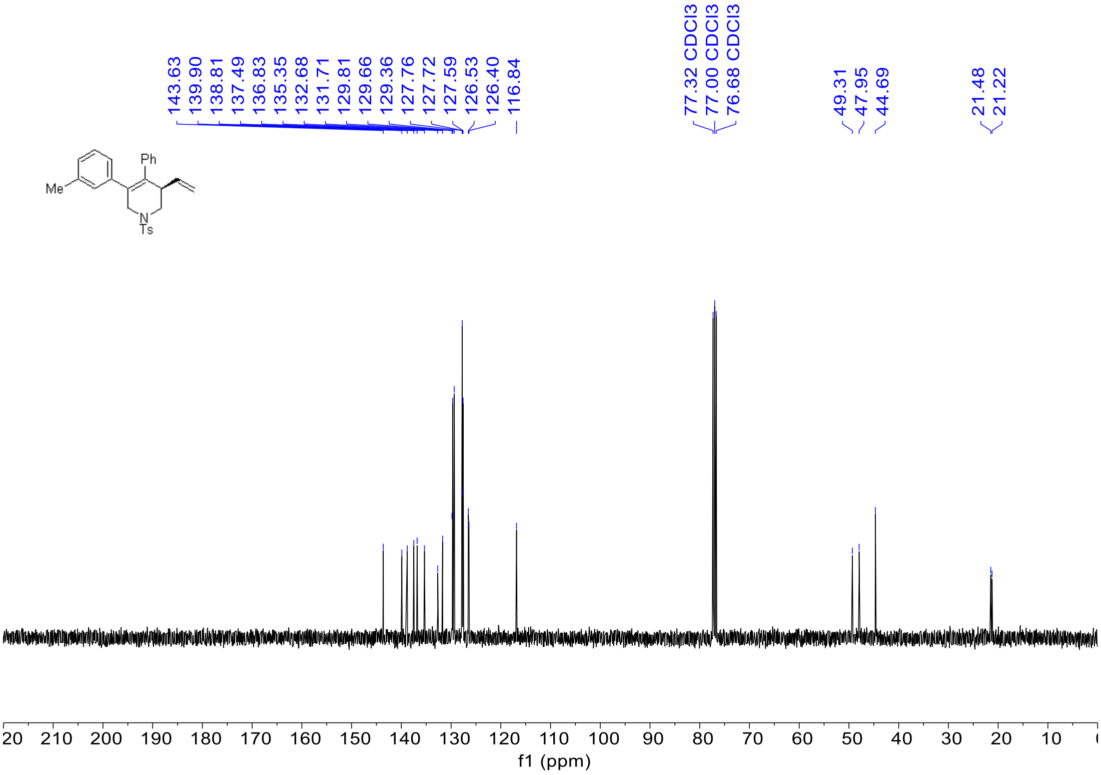

Supplementary Figure 218. ^{13}C NMR spectrum (100 MHz, $CDCl_3$) of **3ai**

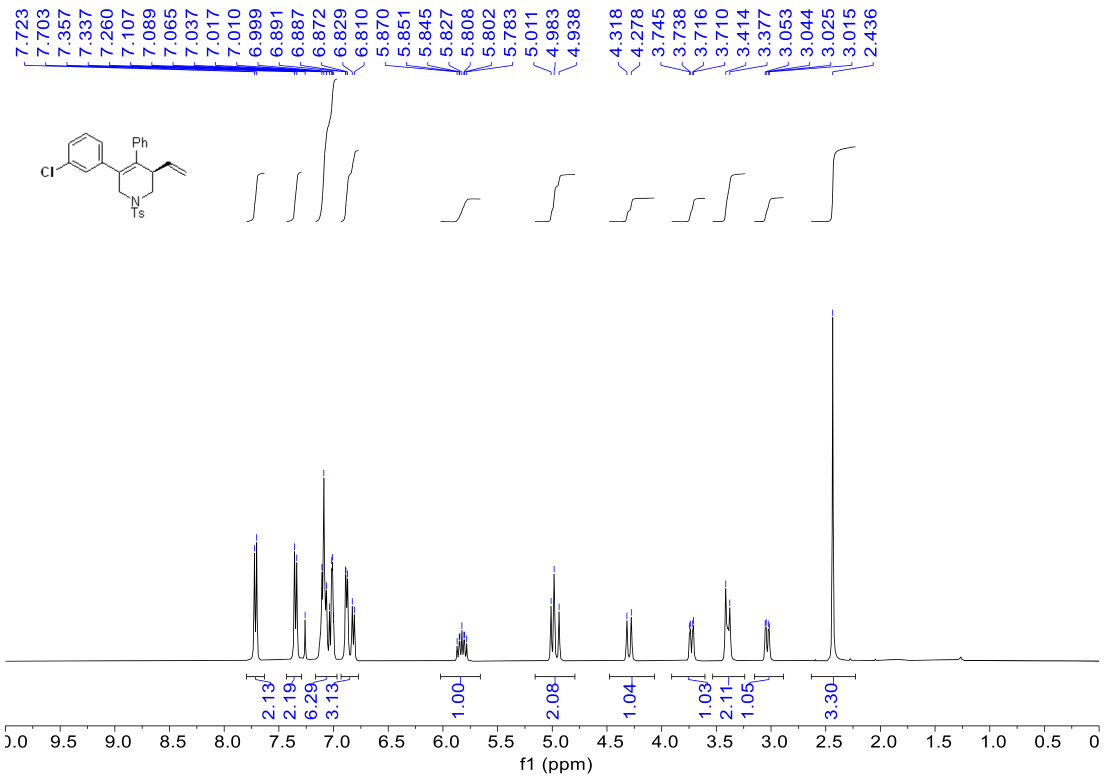

Supplementary Figure 219. ^{1}H NMR spectrum (400 MHz, $CDCl_3$) of **3aj**

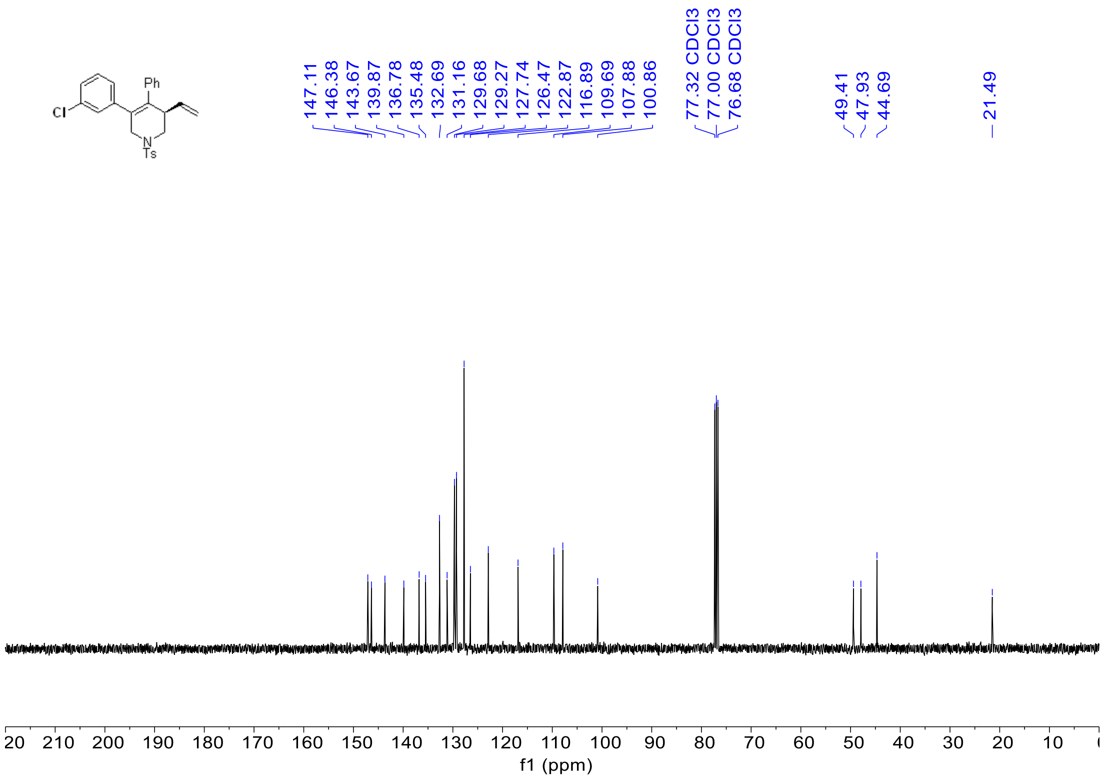

Supplementary Figure 220. ^{13}C NMR spectrum (100 MHz, $CDCl_3$) of **3aj**

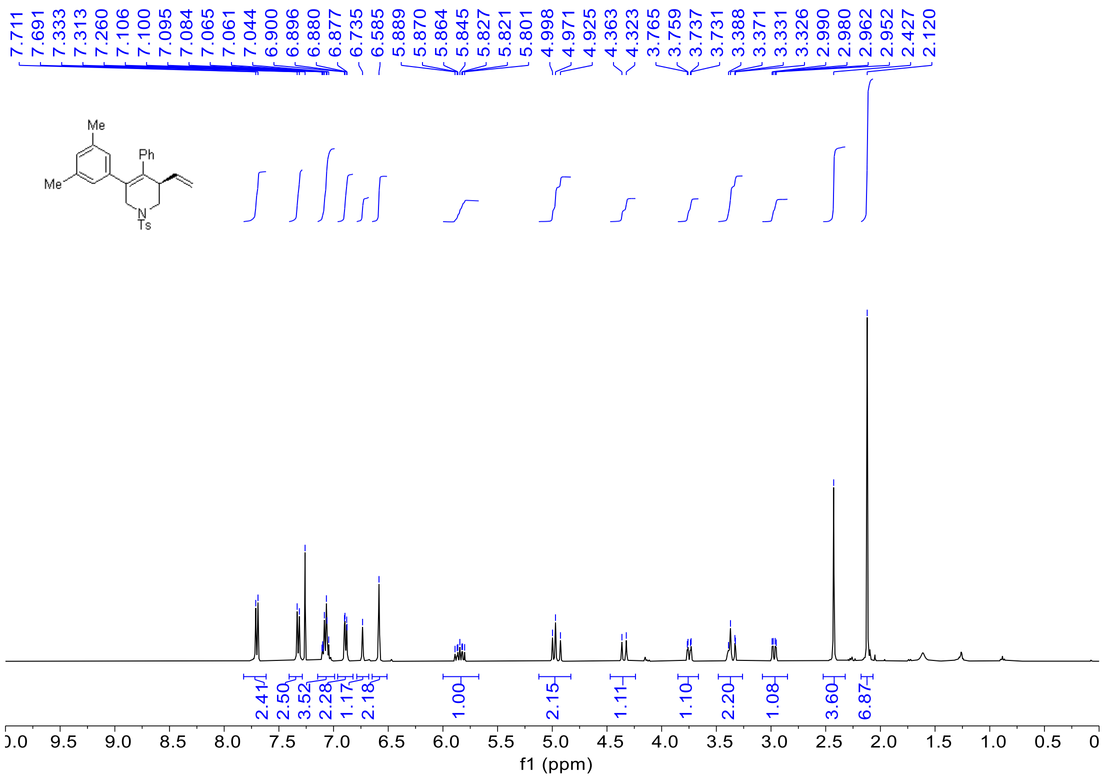

Supplementary Figure 221. ^{1}H NMR spectrum (400 MHz, $CDCl_3$) of **3ak**

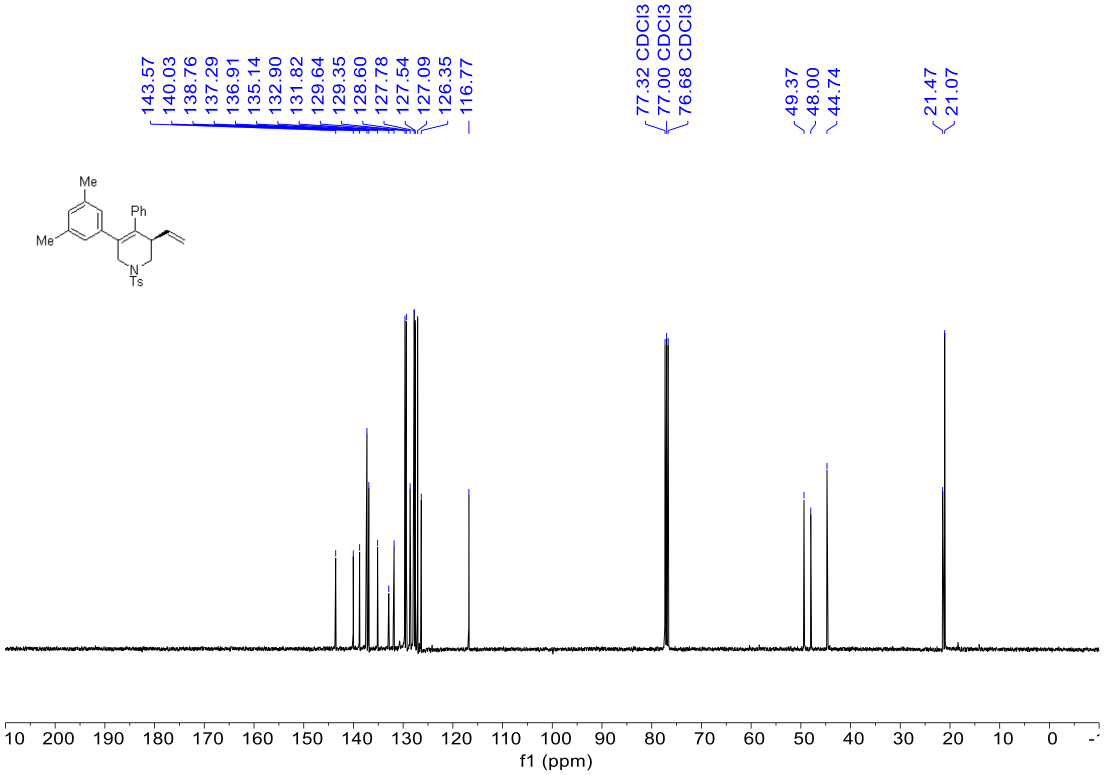

Supplementary Figure 222. ^{13}C NMR spectrum (100 MHz, $CDCl_3$) of **3ak**

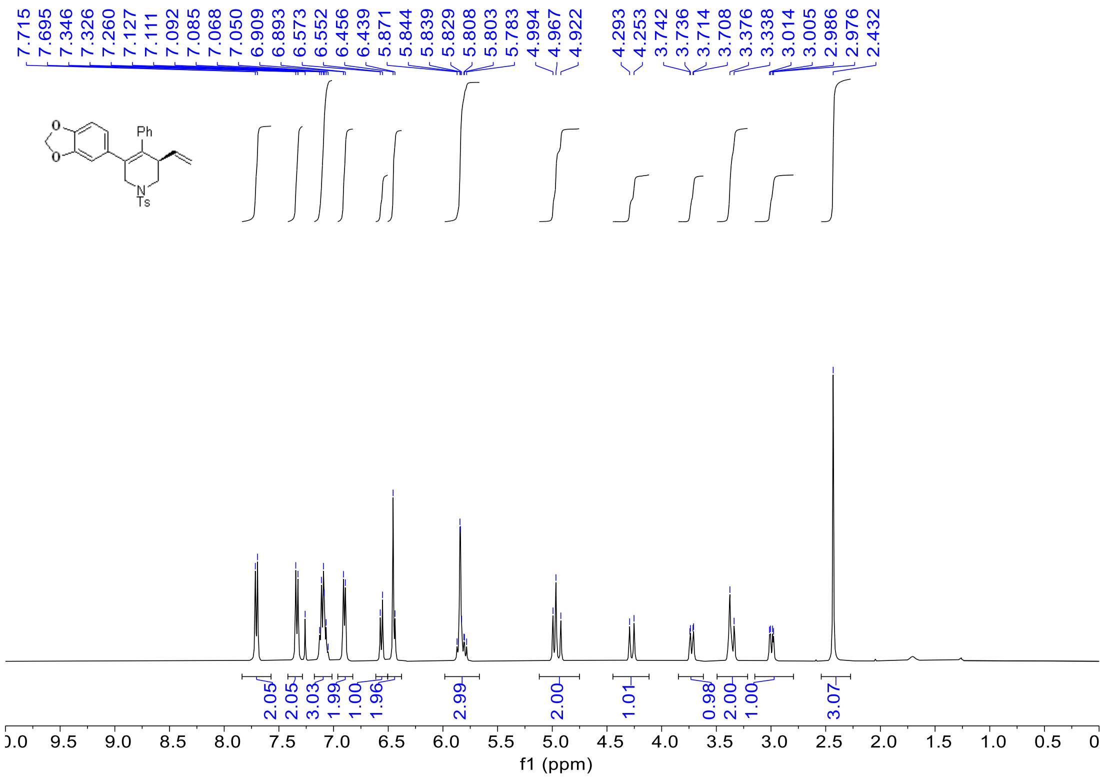

Supplementary Figure 223. ^{1}H NMR spectrum (400 MHz, $CDCl_3$) of **3al**

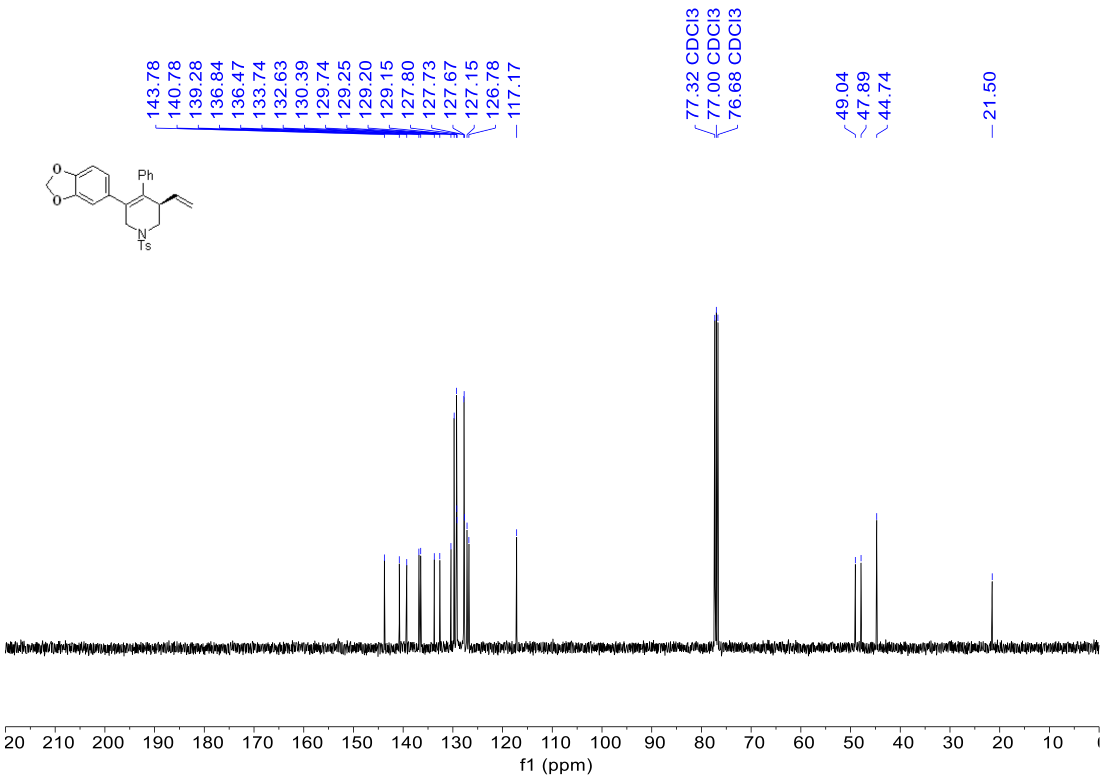

Supplementary Figure 224. ^{13}C NMR spectrum (100 MHz, $CDCl_3$) of **3al**

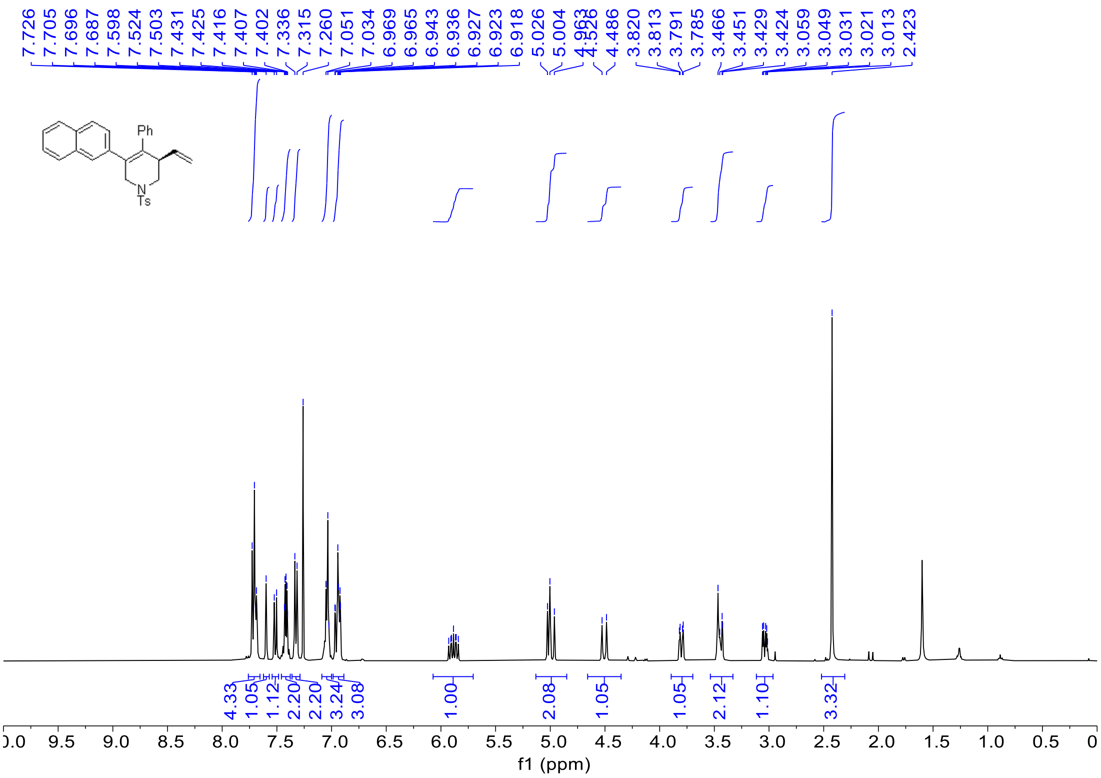

Supplementary Figure 225. ^{1}H NMR spectrum (400 MHz, $CDCl_3$) of **3am**

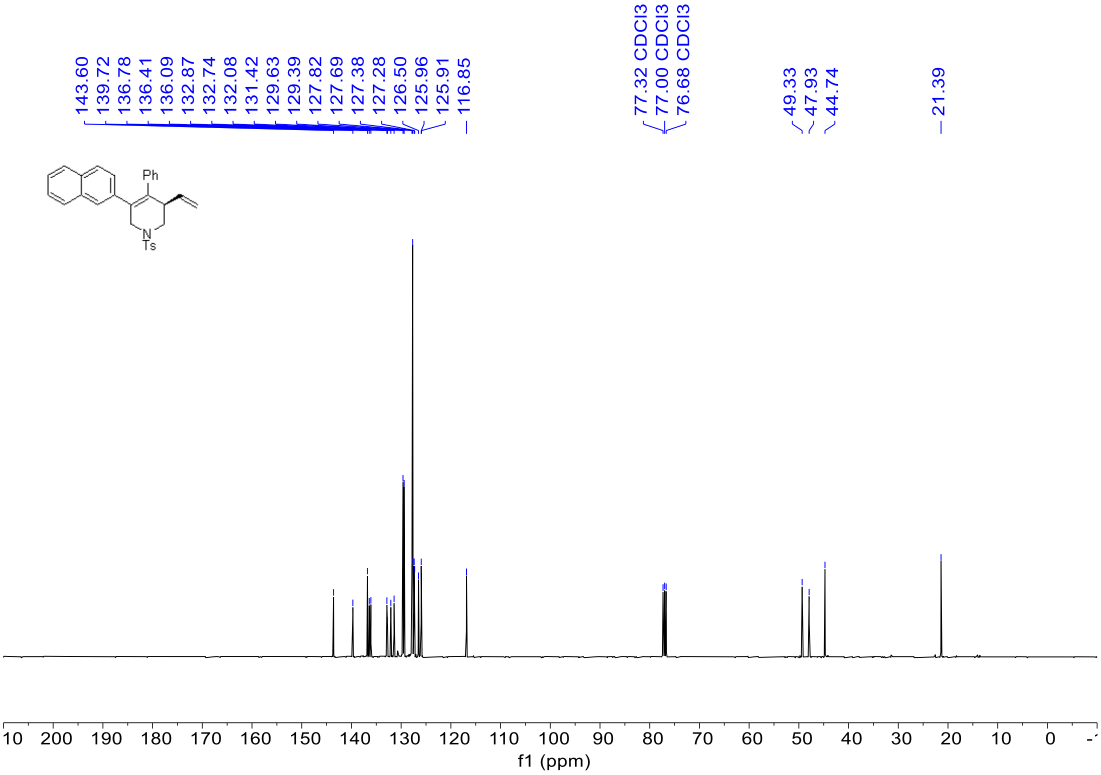

Supplementary Figure 226. ^{13}C NMR spectrum (100 MHz, $CDCl_3$) of **3am**

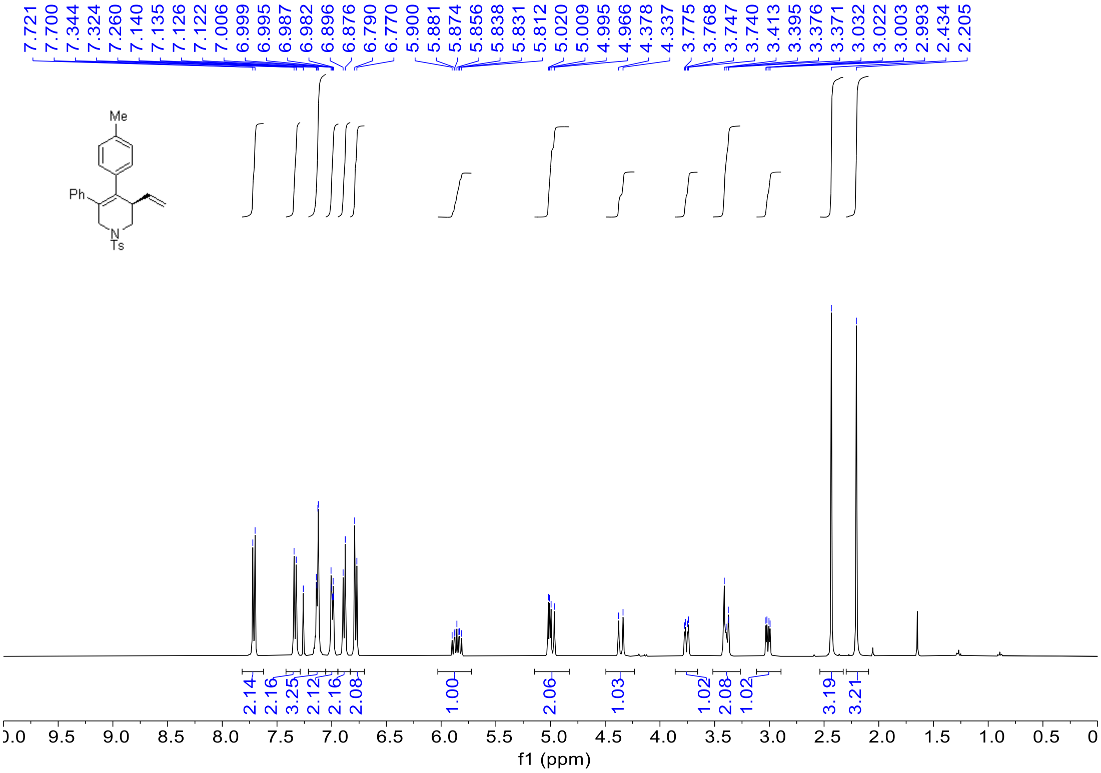

Supplementary Figure 227. ^{1}H NMR spectrum (400 MHz, $CDCl_3$) of **3ba**

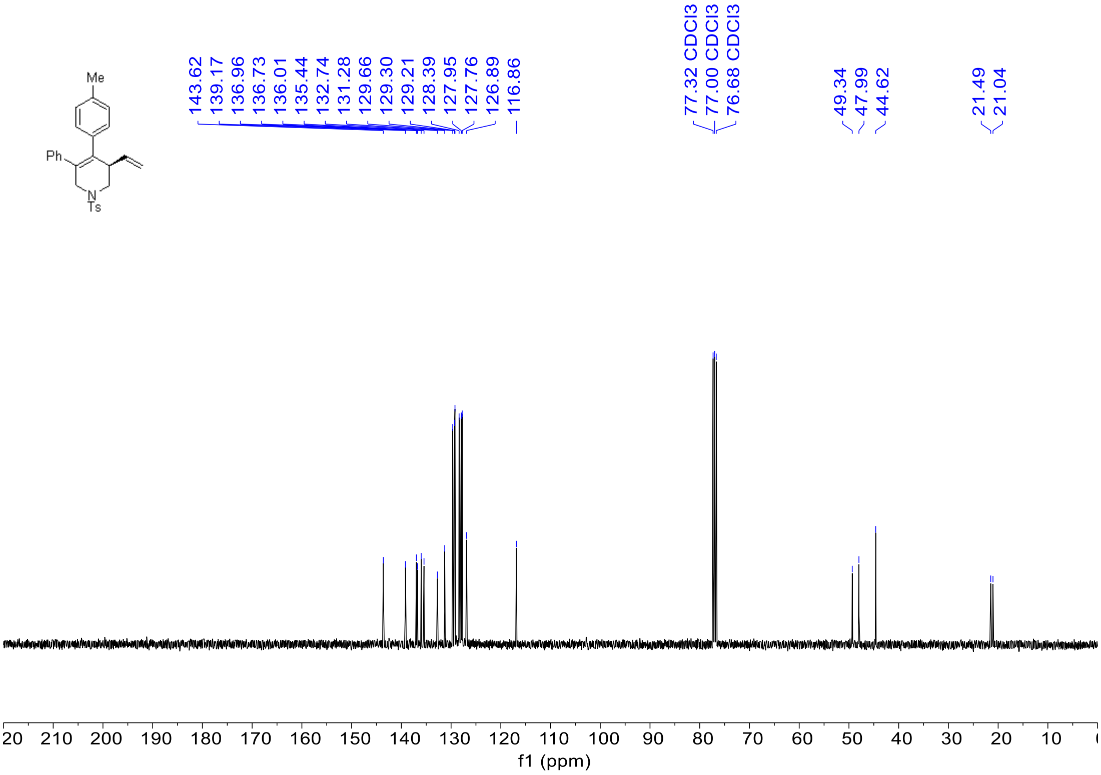

Supplementary Figure 228. ^{13}C NMR spectrum (100 MHz, $CDCl_3$) of **3ba**

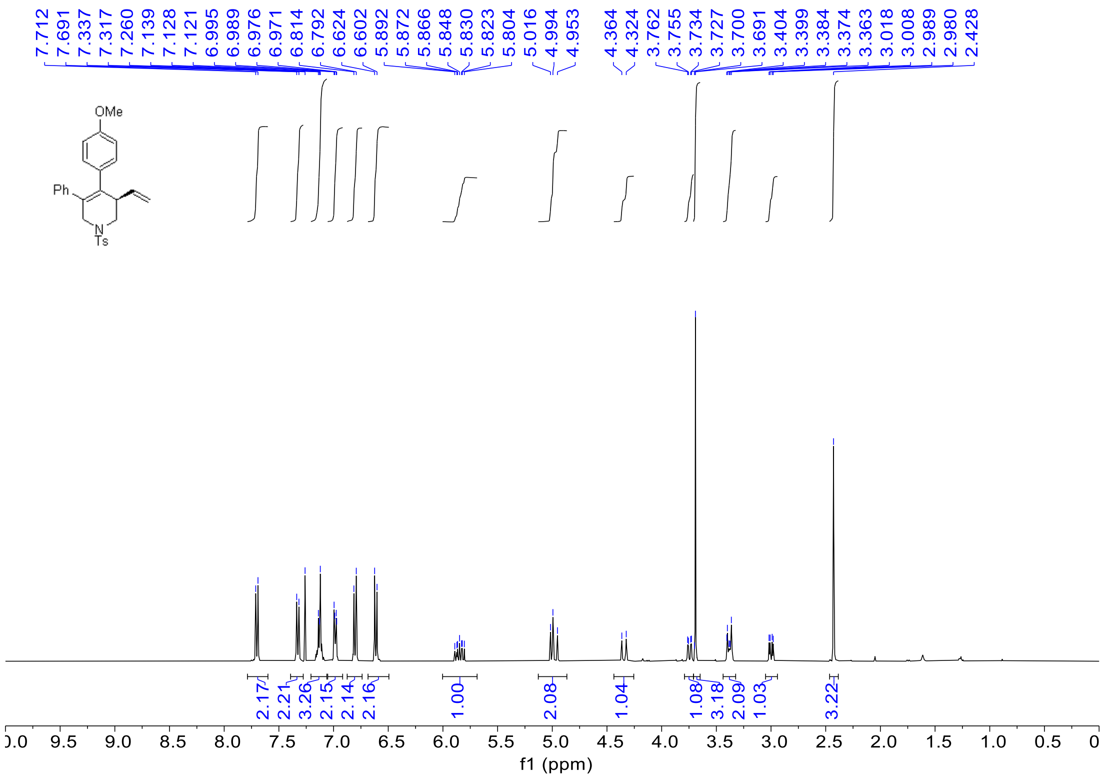

Supplementary Figure 229. ^{1}H NMR spectrum (400 MHz, $CDCl_3$) of **3ca**

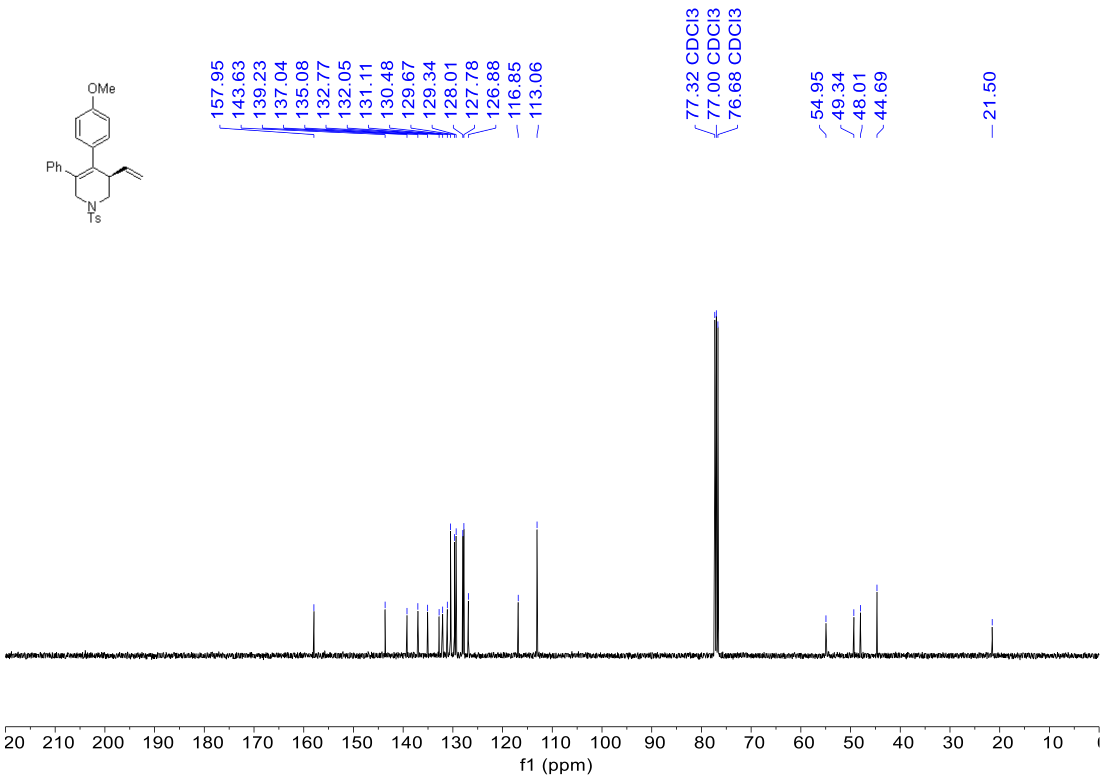

Supplementary Figure 230. ^{13}C NMR spectrum (100 MHz, $CDCl_3$) of **3ca**

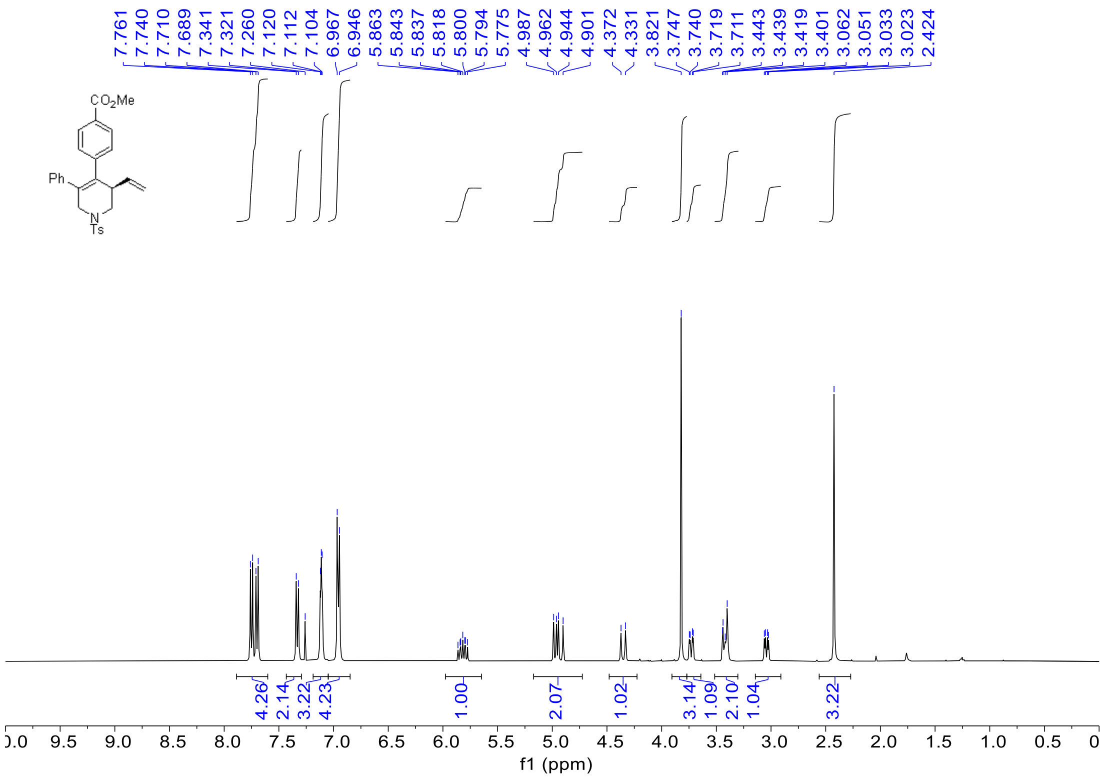

Supplementary Figure 231. ^{1}H NMR spectrum (400 MHz, $CDCl_3$) of **3da**

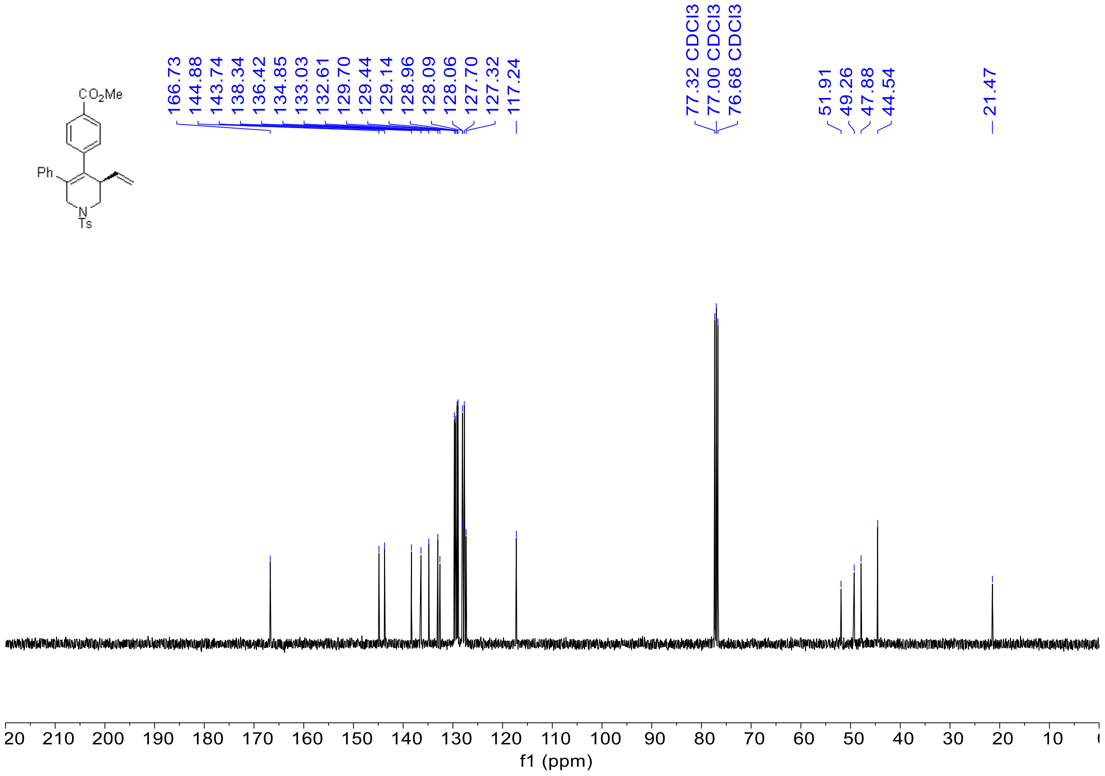

Supplementary Figure 232. ^{13}C NMR spectrum (100 MHz, $CDCl_3$) of **3da**

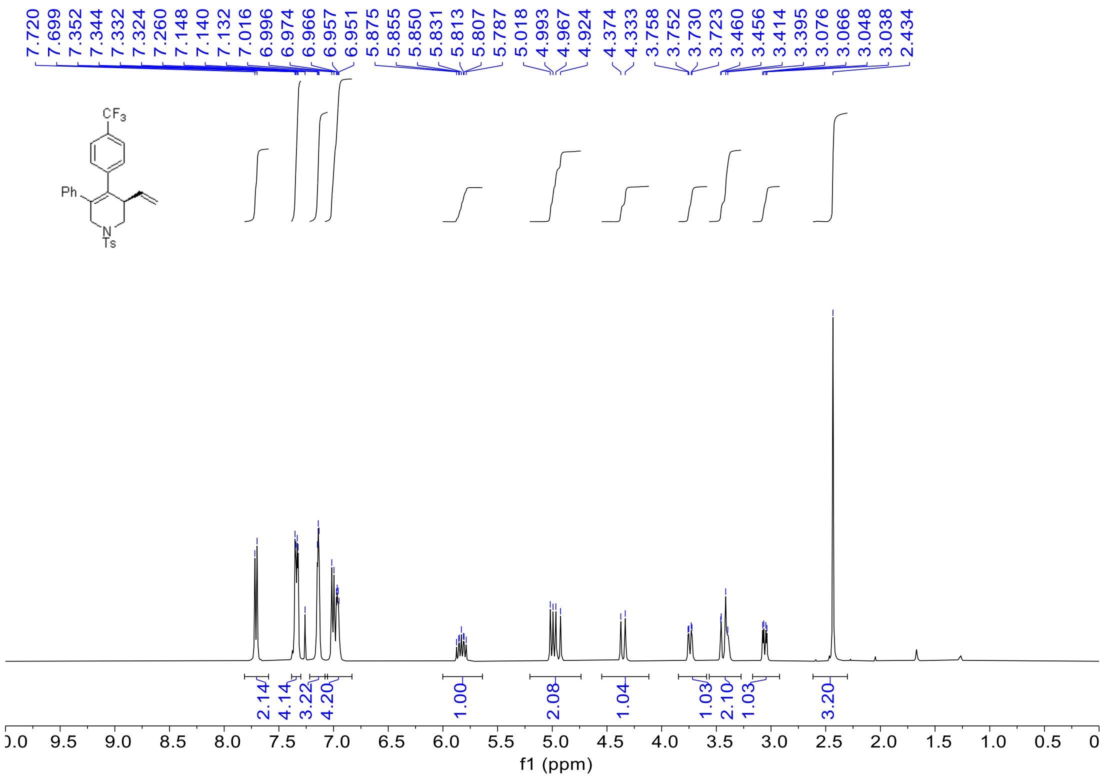

Supplementary Figure 233. ^{1}H NMR spectrum (400 MHz, $CDCl_3$) of **3ea**

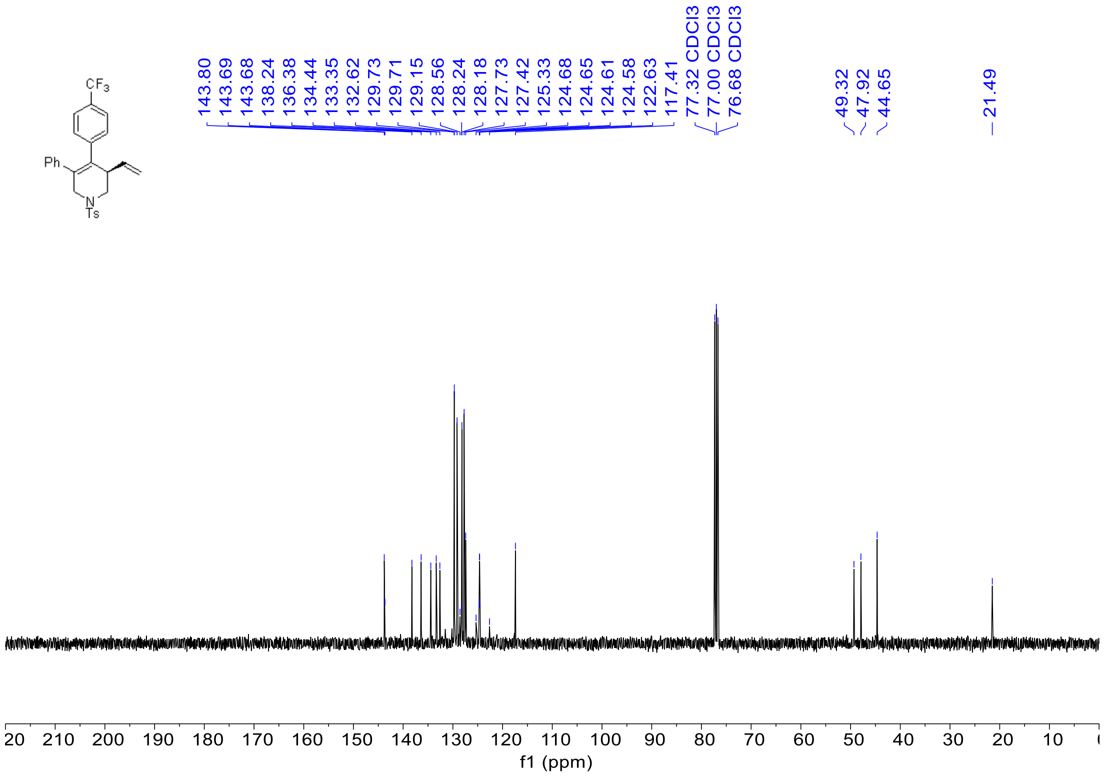

Supplementary Figure 234. ^{13}C NMR spectrum (100 MHz, $CDCl_3$) of **3ea**

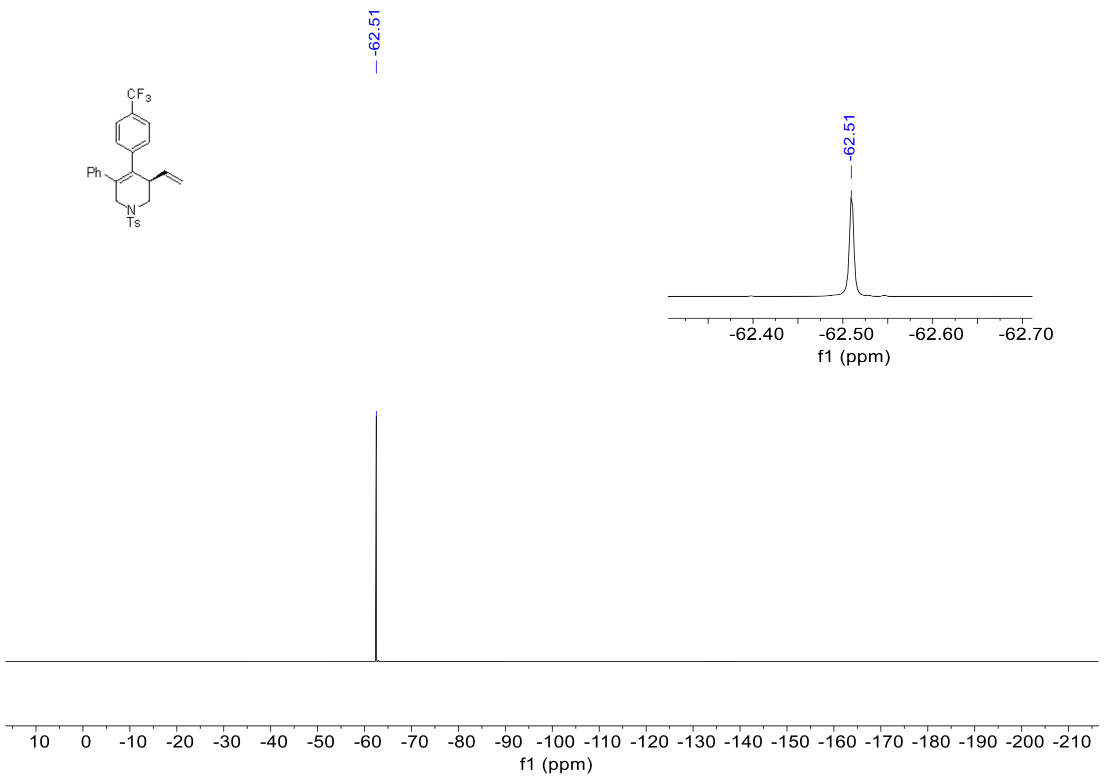

Supplementary Figure 235. ^{19}F NMR spectrum (565 MHz, $CDCl_3$) of **3ea**

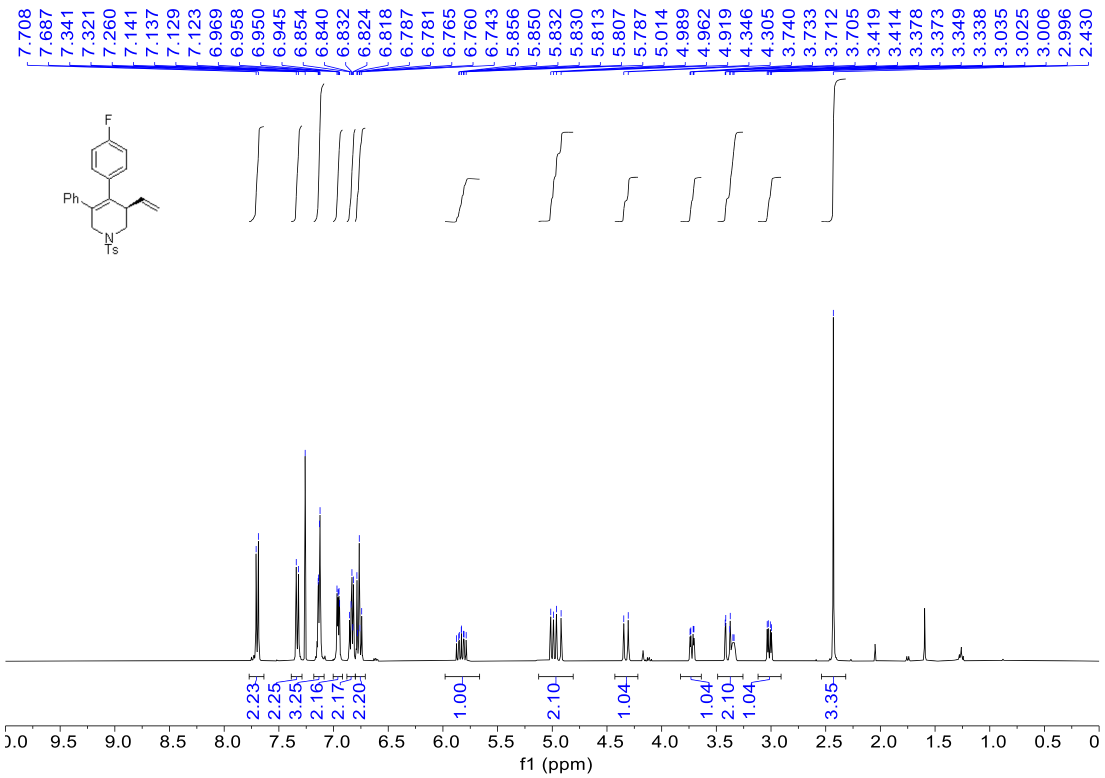

Supplementary Figure 236. ^{1}H NMR spectrum (400 MHz, $CDCl_3$) of **3fa**

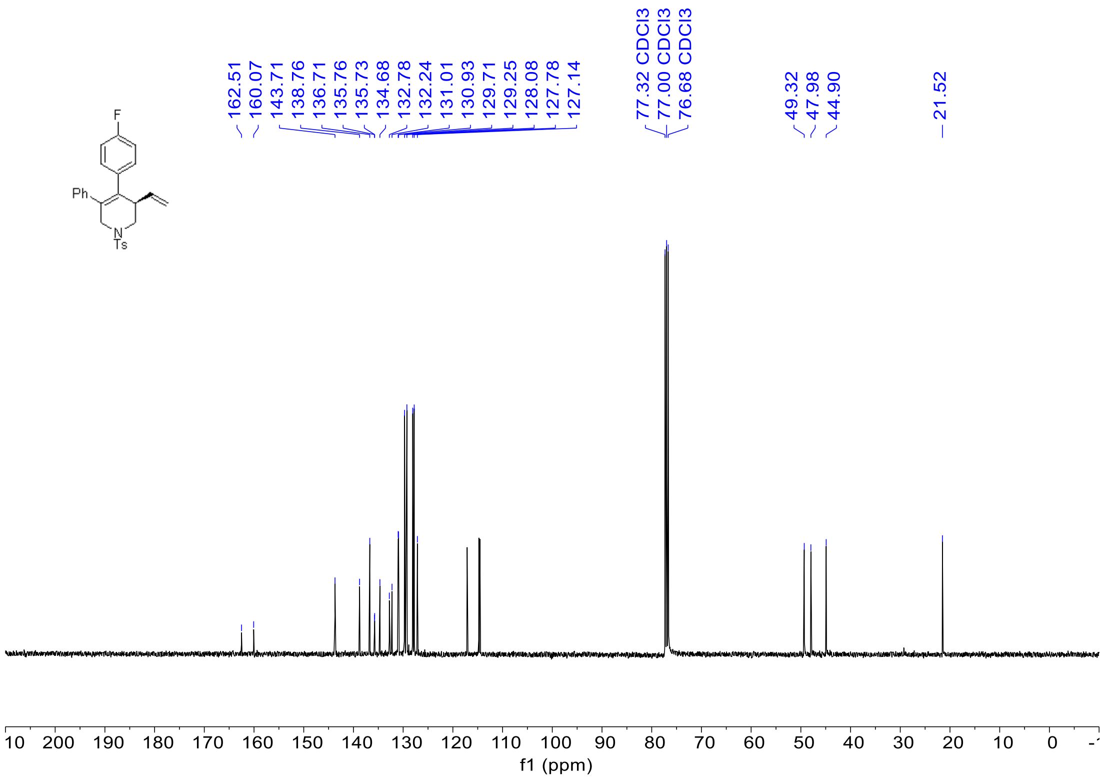

Supplementary Figure 237. ^{13}C NMR spectrum (100 MHz, $CDCl_3$) of **3fa**

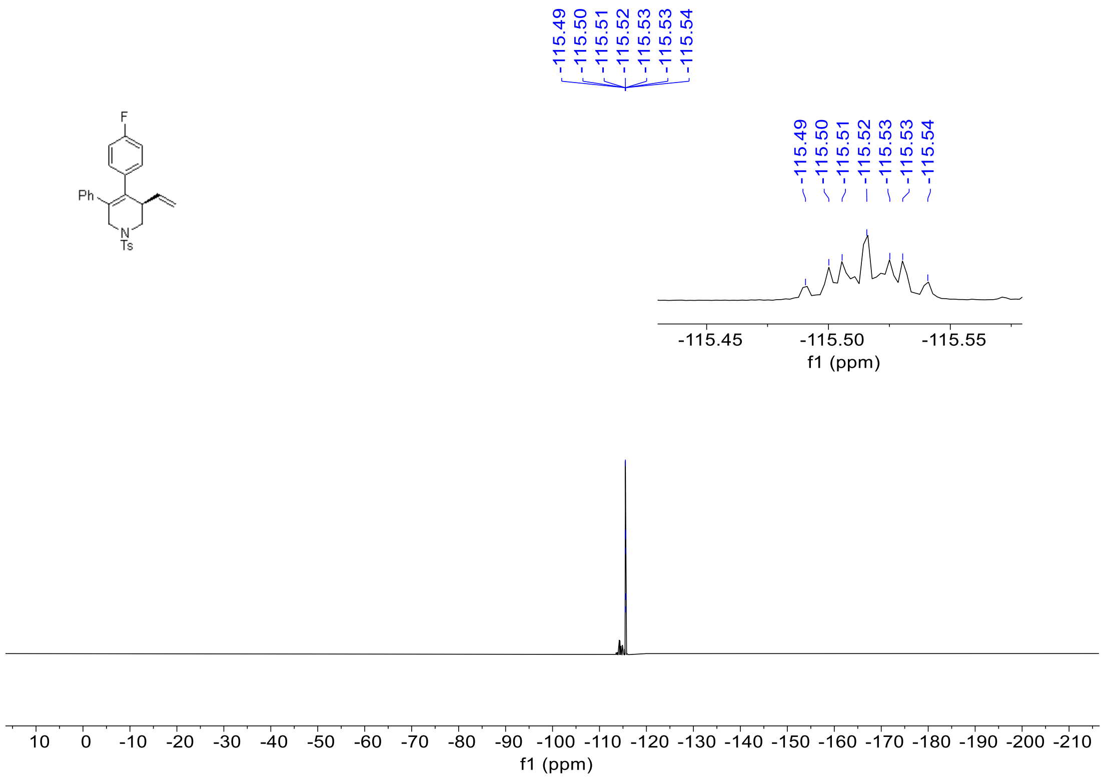

Supplementary Figure 238. ^{19}F NMR spectrum (565 MHz, $CDCl_3$) of **3fa**

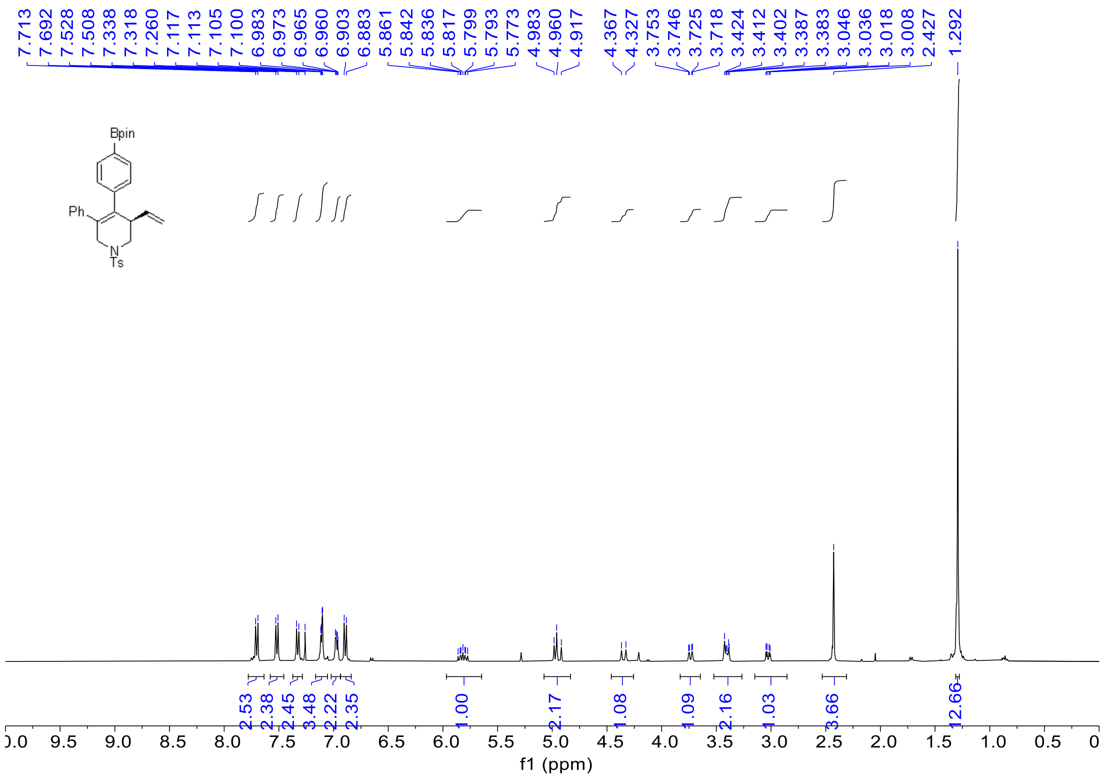

Supplementary Figure 239. ^{1}H NMR spectrum (400 MHz, $CDCl_3$) of **3ga**

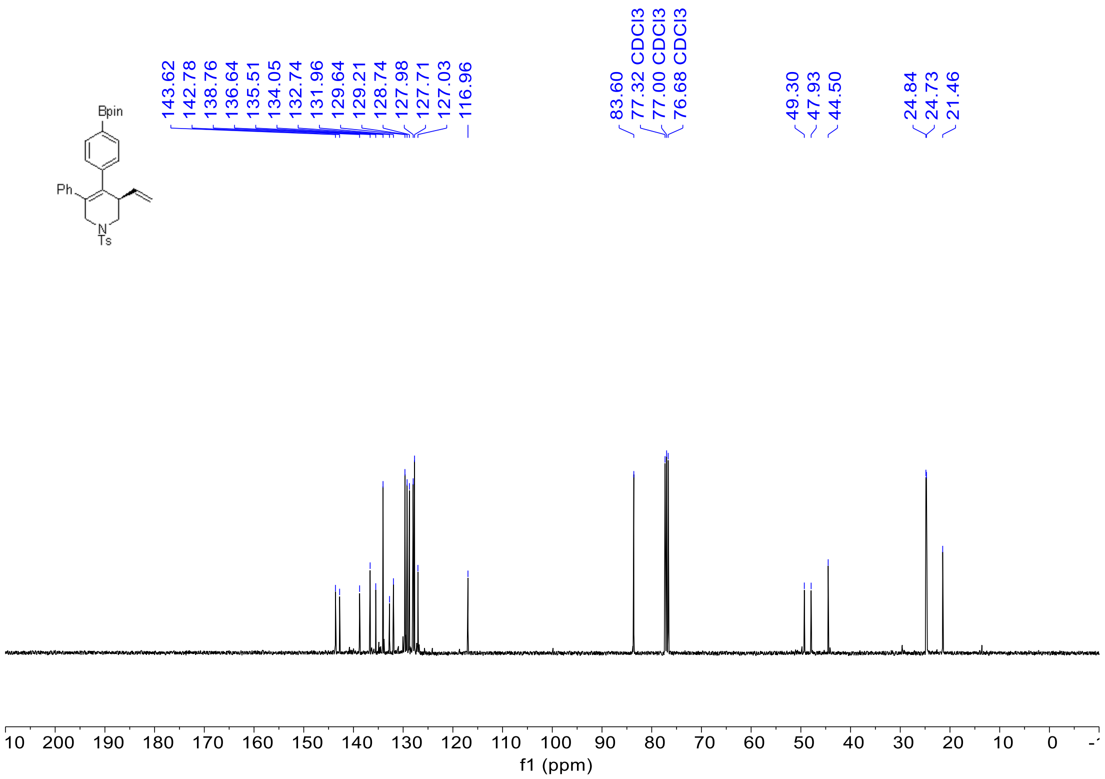

Supplementary Figure 240. ^{13}C NMR spectrum (100 MHz, $CDCl_3$) of **3ga**

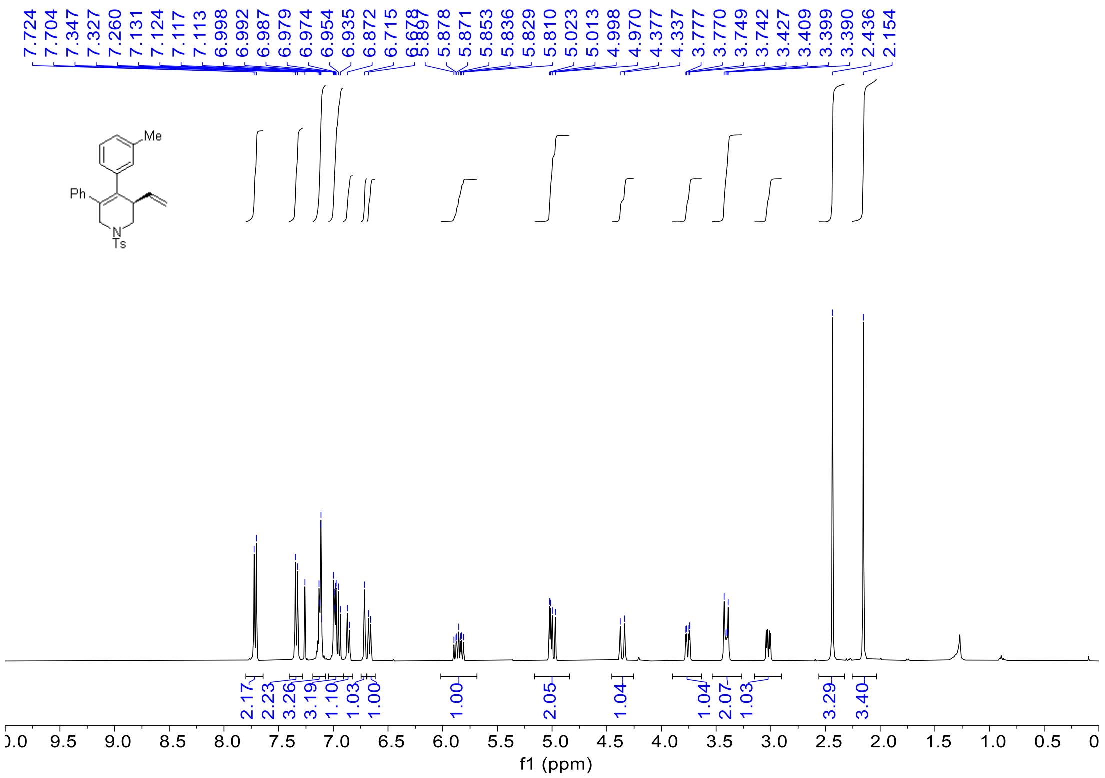

Supplementary Figure 241. ^{1}H NMR spectrum (400 MHz, $CDCl_3$) of **3ha**

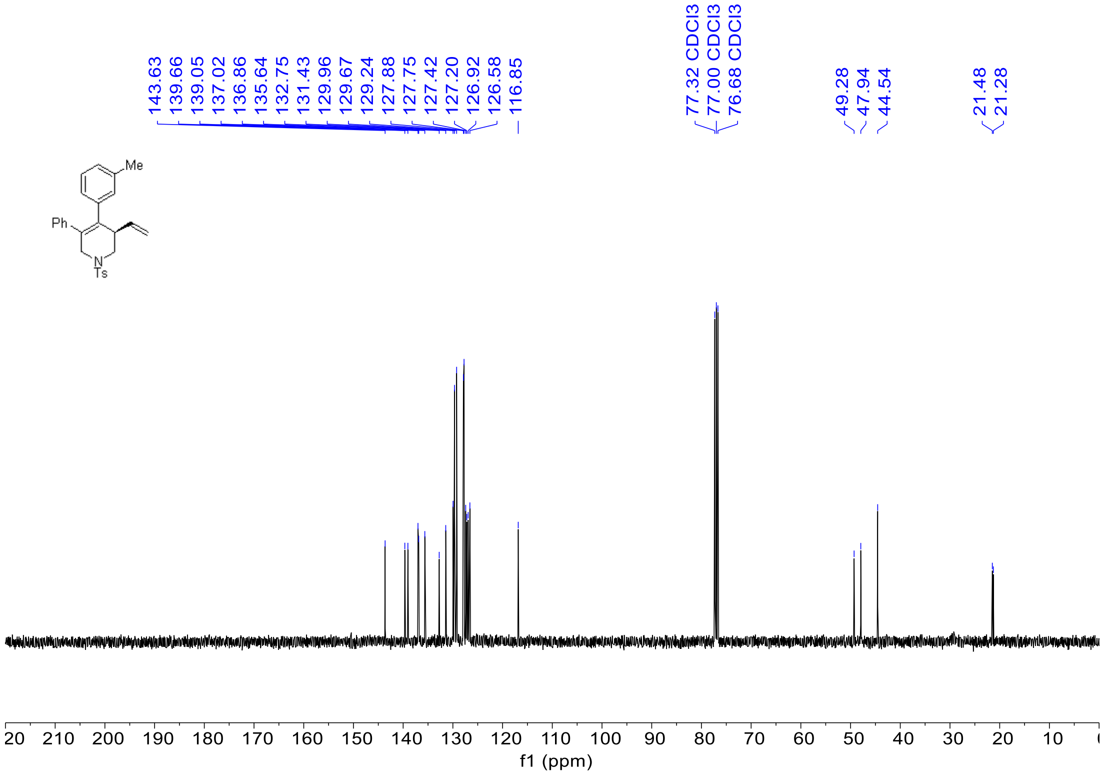

Supplementary Figure 242. ^{13}C NMR spectrum (100 MHz, $CDCl_3$) of **3ha**

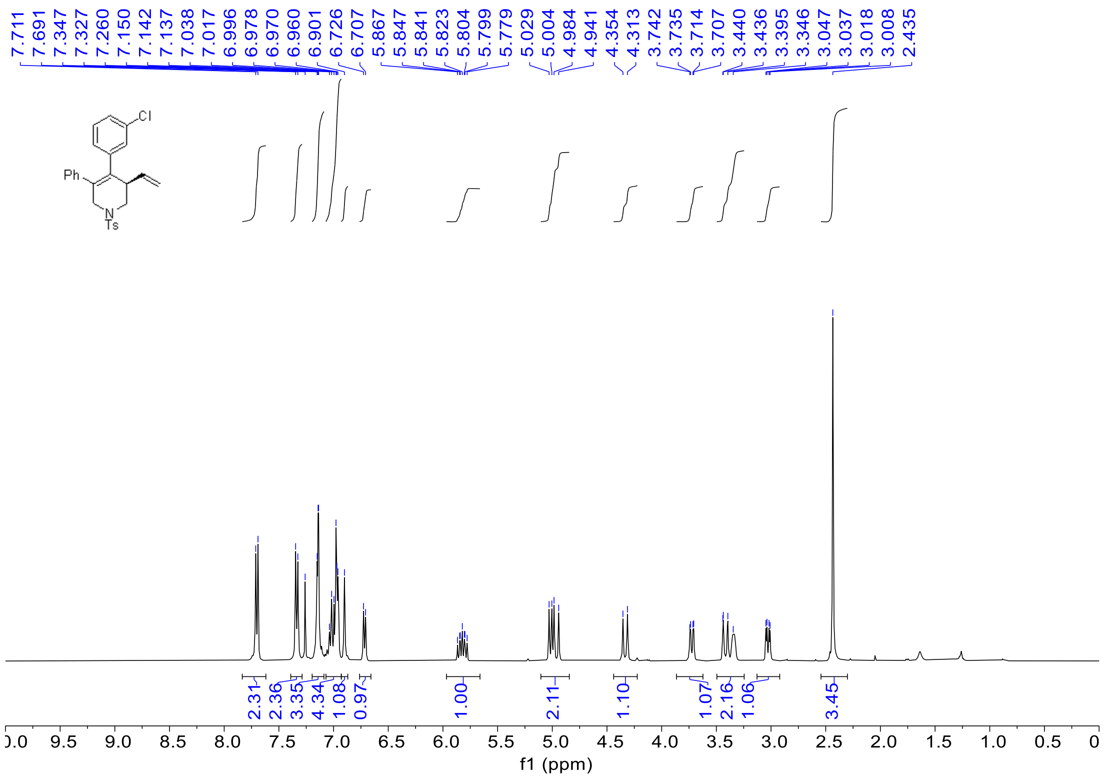

Supplementary Figure 243. ^{1}H NMR spectrum (400 MHz, $CDCl_3$) of **3ia**

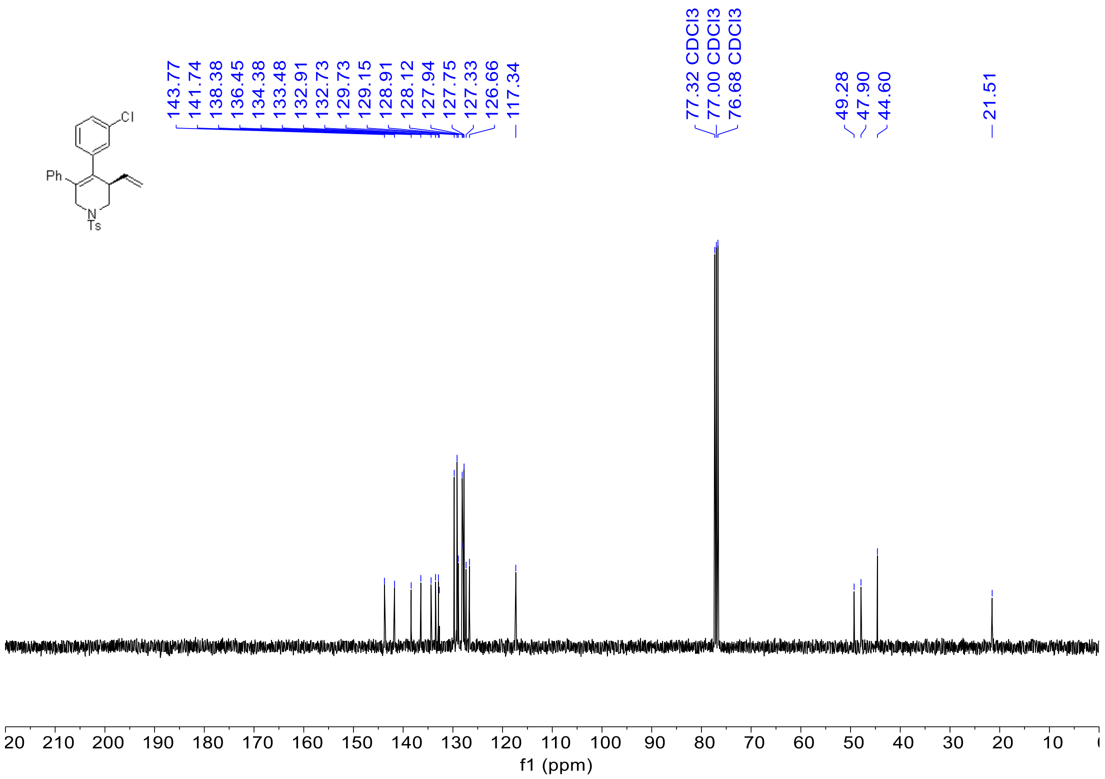

Supplementary Figure 244. ^{13}C NMR spectrum (100 MHz, $CDCl_3$) of **3ia**

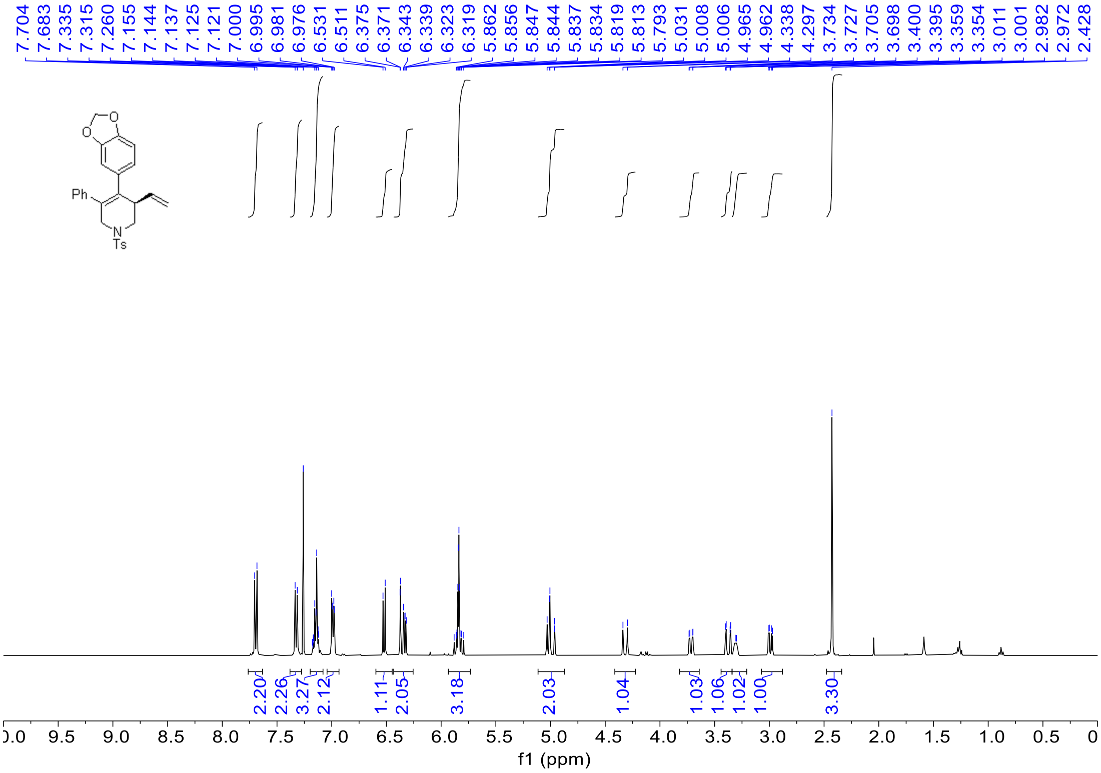

Supplementary Figure 245. ^{1}H NMR spectrum (400 MHz, $CDCl_3$) of **3ja**

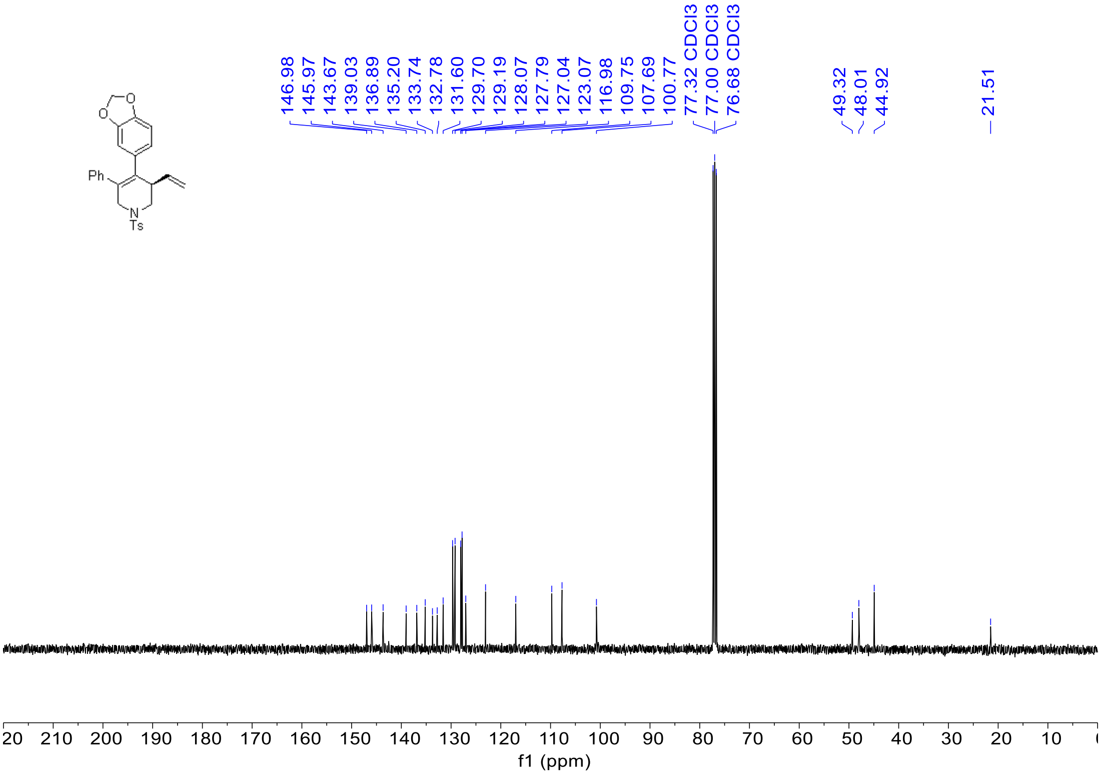

Supplementary Figure 246. ^{13}C NMR spectrum (100 MHz, $CDCl_3$) of **3ja**

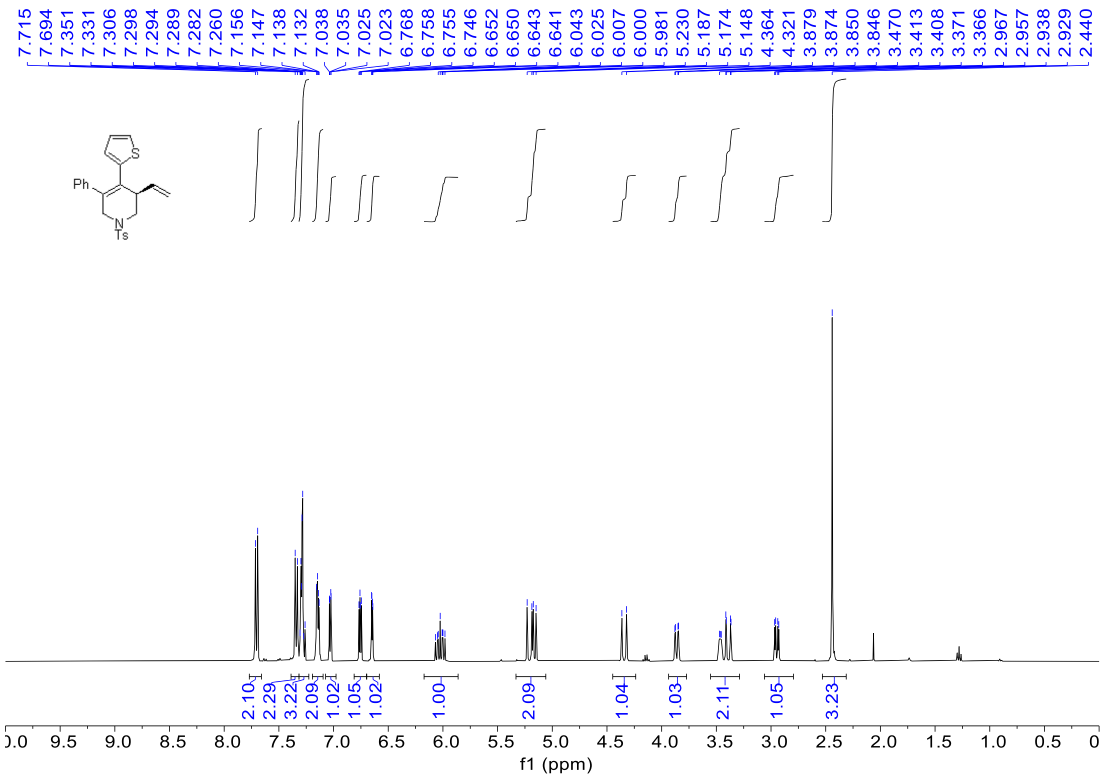

Supplementary Figure 247. ^{1}H NMR spectrum (400 MHz, $CDCl_3$) of **3ka**

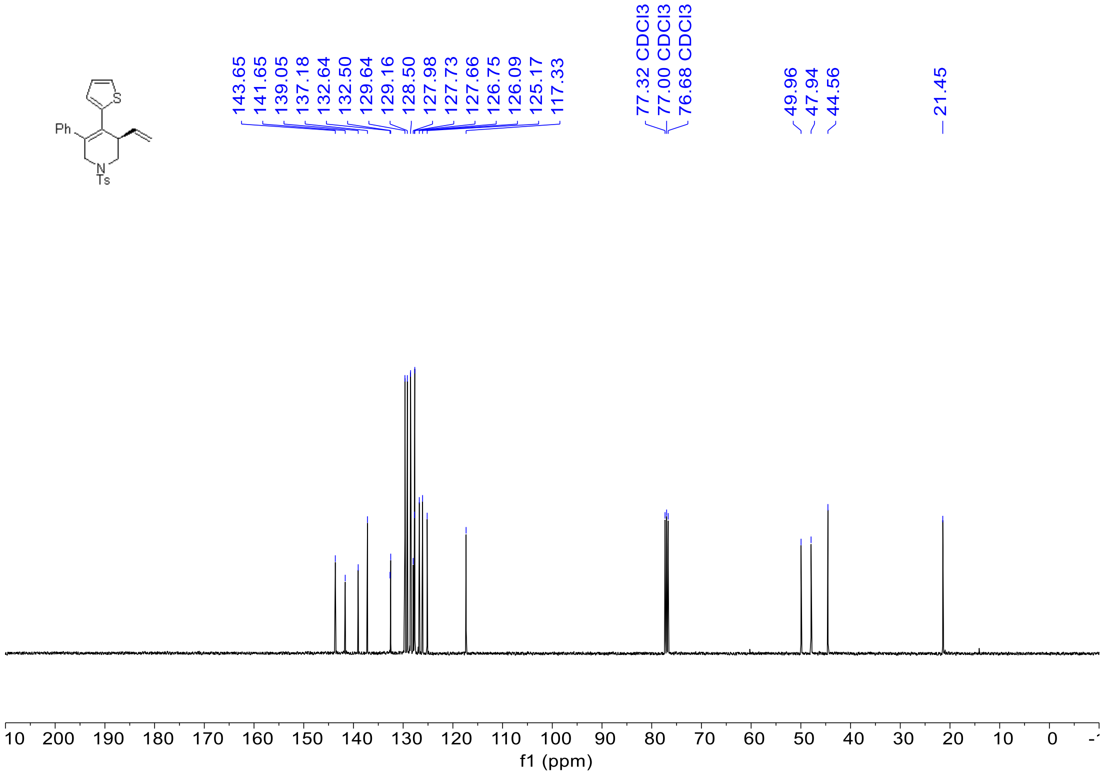

Supplementary Figure 248. ^{13}C NMR spectrum (100 MHz, $CDCl_3$) of **3ka**

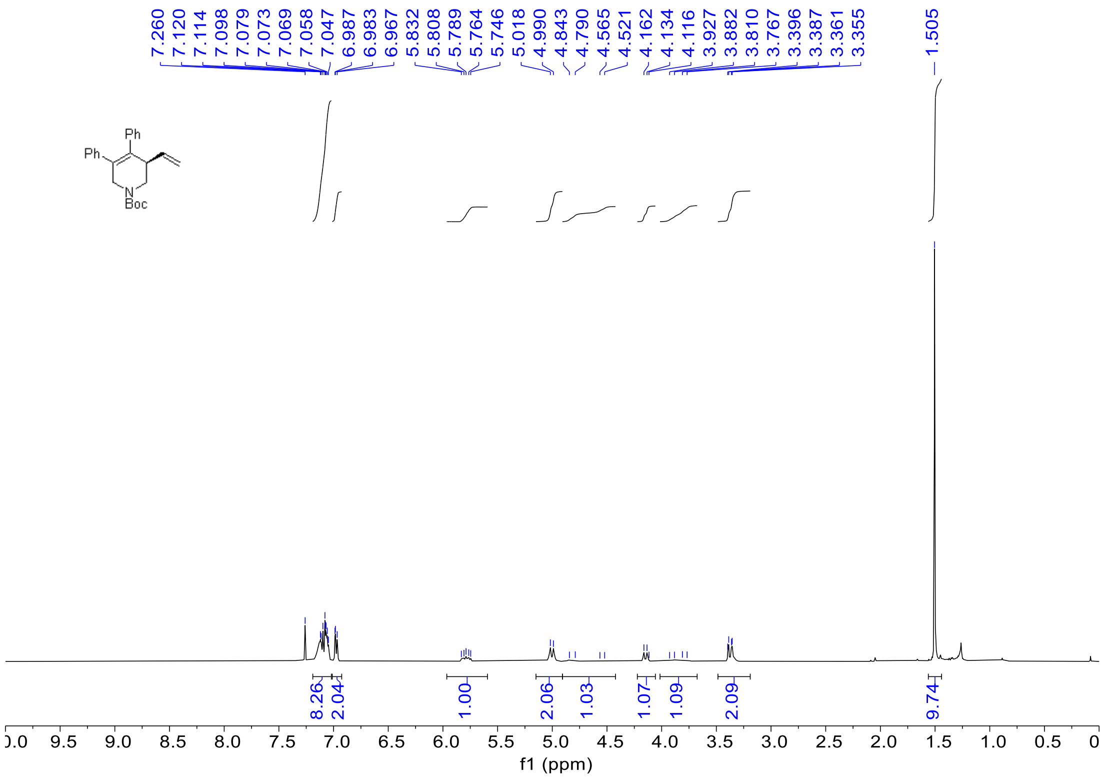

Supplementary Figure 249. ^{1}H NMR spectrum (400 MHz, $CDCl_3$) of **3la**

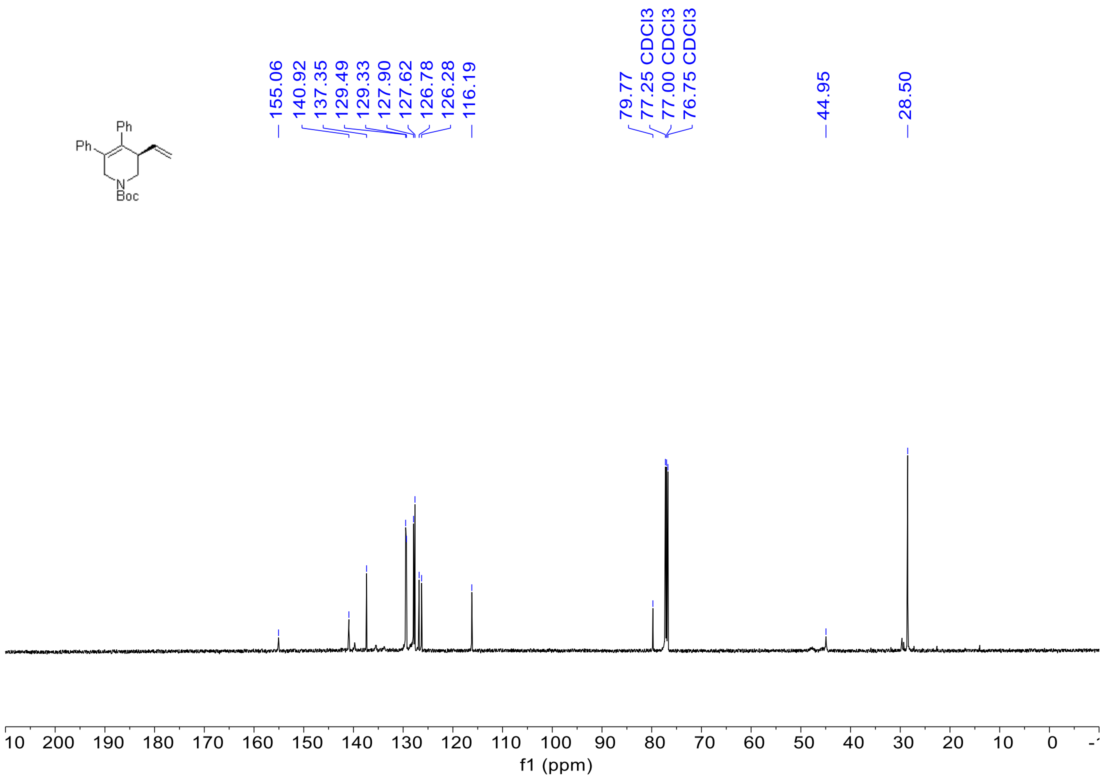

Supplementary Figure 250. ^{13}C NMR spectrum (125 MHz, $CDCl_3$, 50 ºC) of **3la**

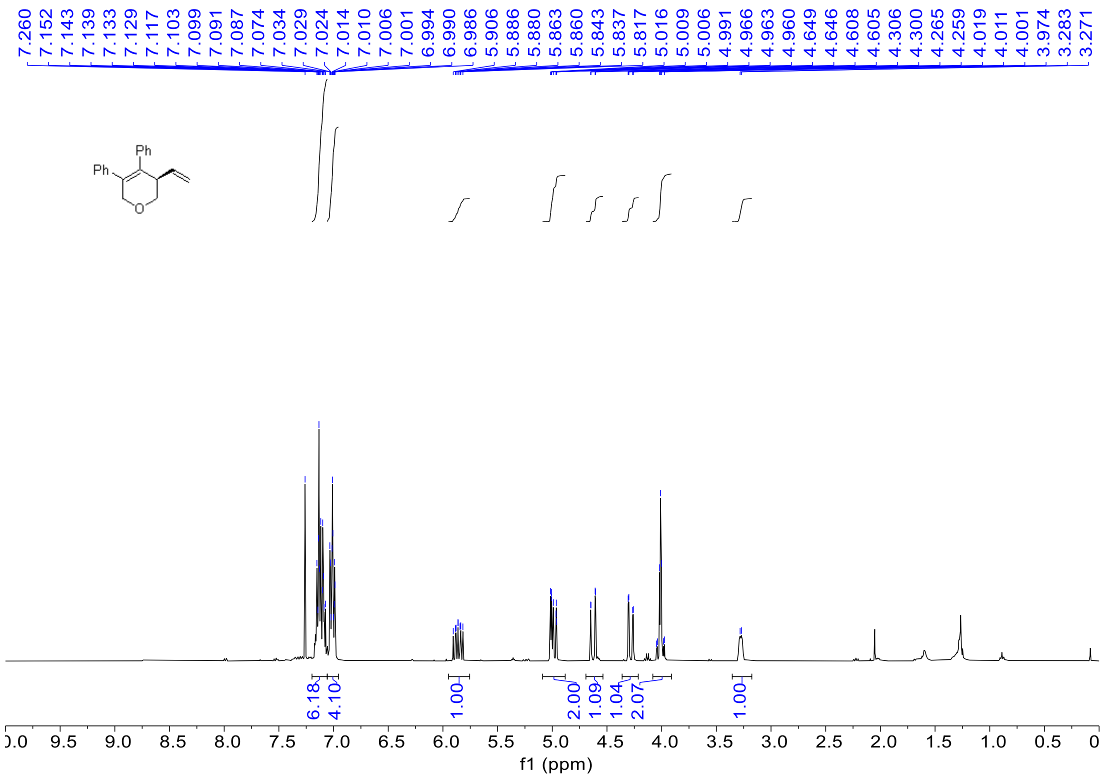

Supplementary Figure 251. ^{1}H NMR spectrum (400 MHz, $CDCl_3$) of **3ma**

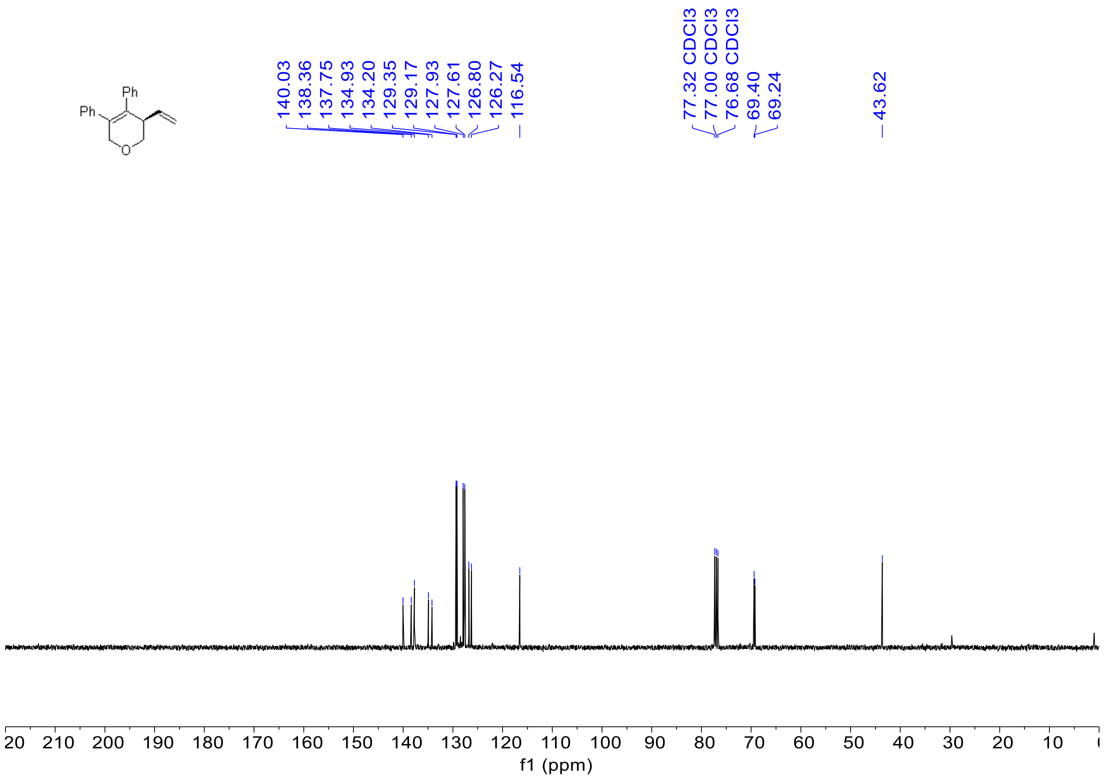

Supplementary Figure 252. ^{13}C NMR spectrum (100 MHz, $CDCl_3$) of **3ma**

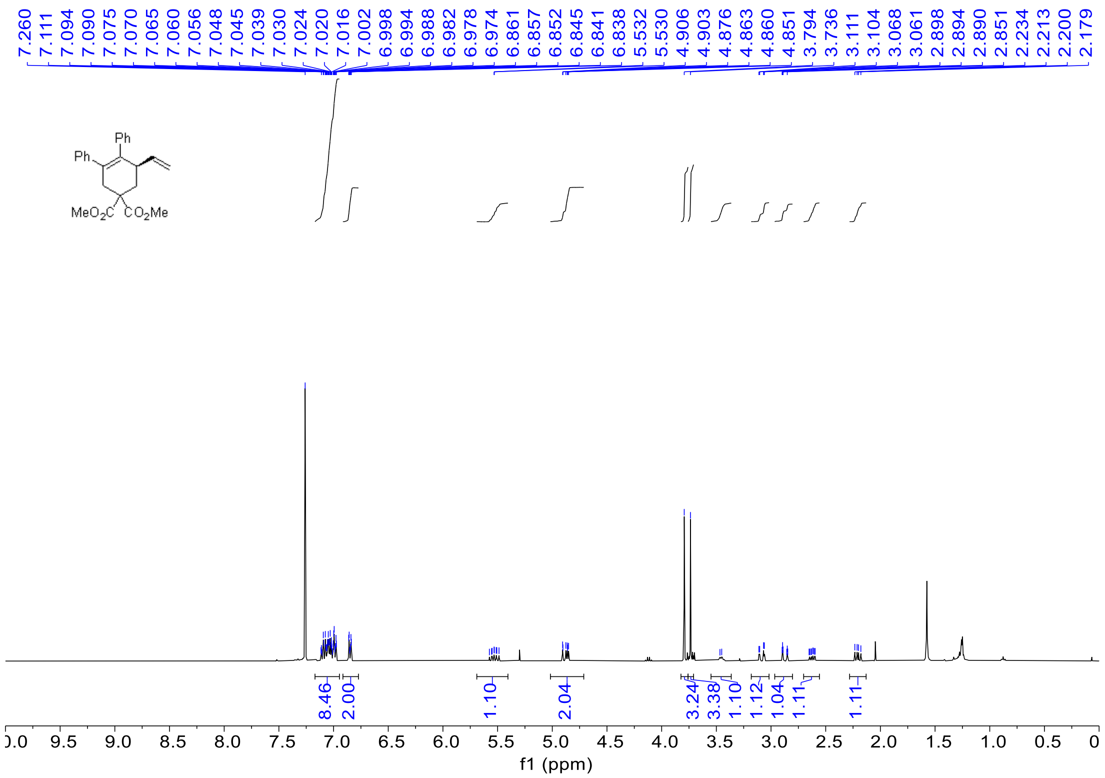

Supplementary Figure 253. ^{1}H NMR spectrum (400 MHz, $CDCl_3$) of **3na**

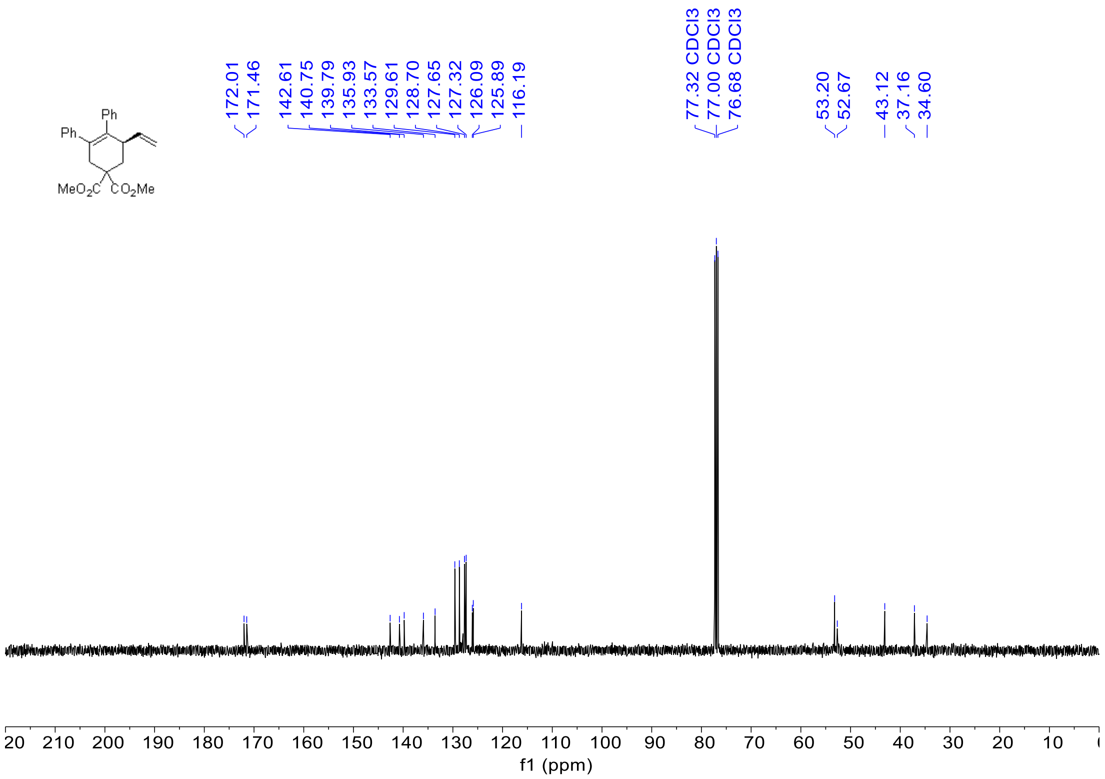

Supplementary Figure 254. ^{13}C NMR spectrum (100 MHz, $CDCl_3$) of **3na**

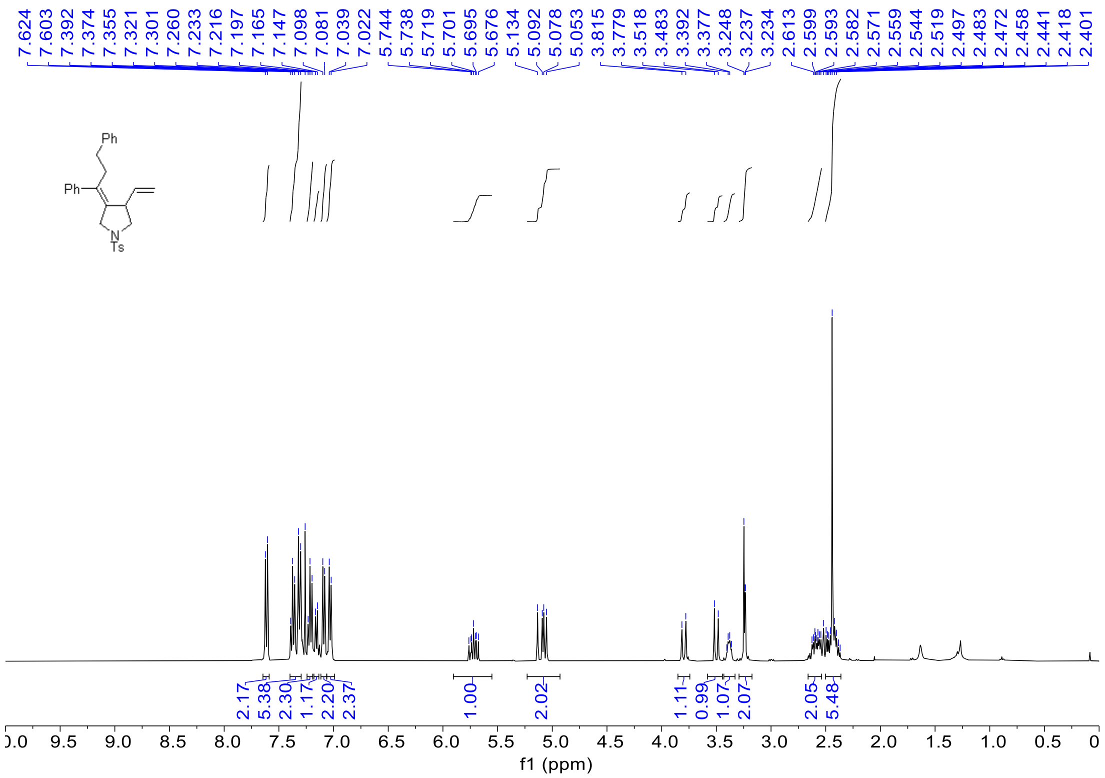

Supplementary Figure 255. ^{1}H NMR spectrum (400 MHz, $CDCl_3$) of **6aa**

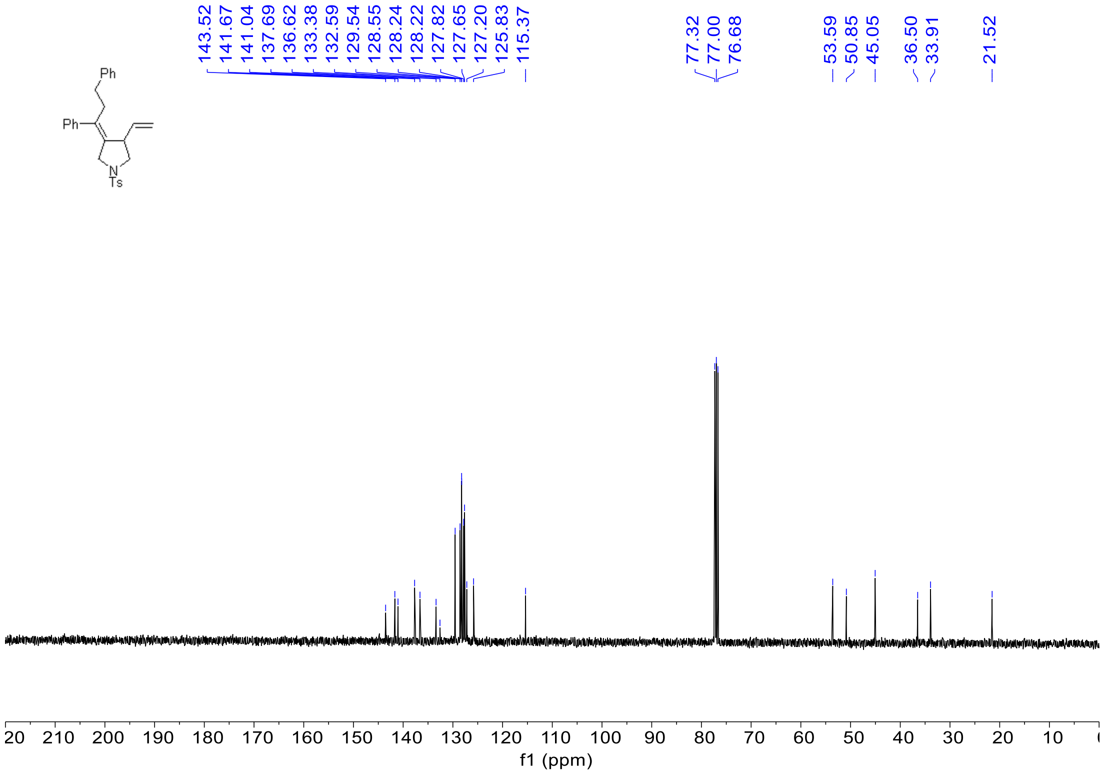

Supplementary Figure 256. ^{13}C NMR spectrum (100 MHz, $CDCl_3$) of **6aa**

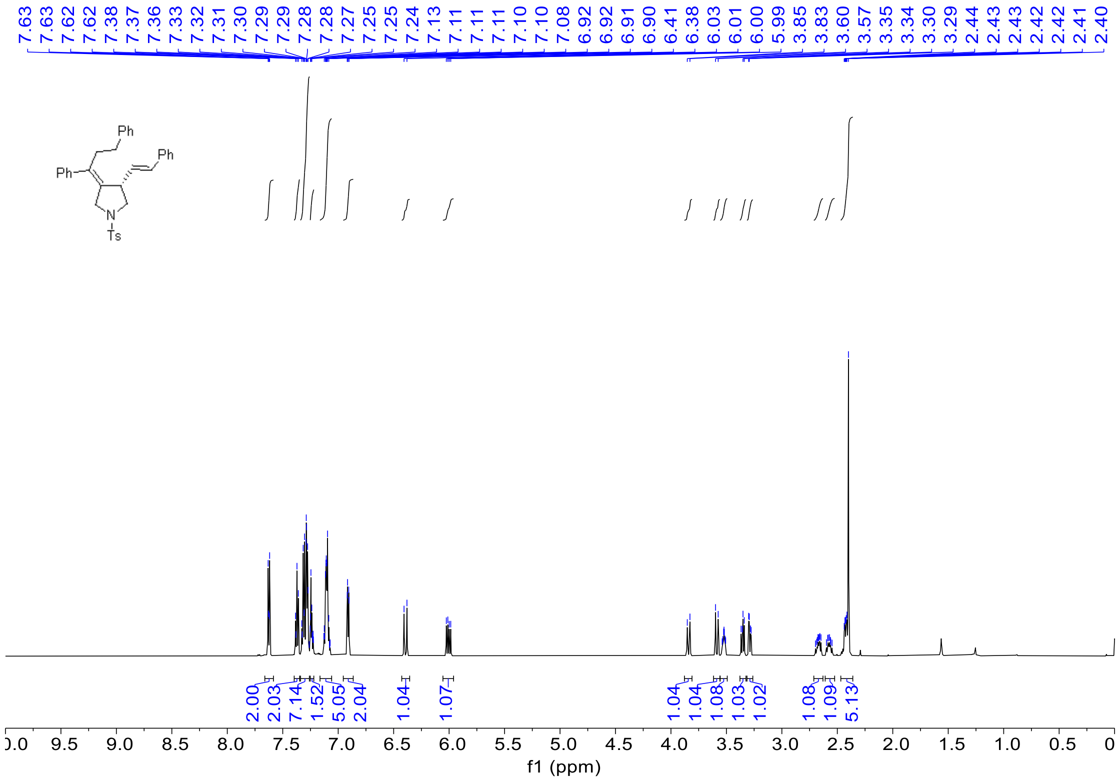

Supplementary Figure 257. ^{1}H NMR spectrum (600 MHz, $CDCl_3$) of **7aa**

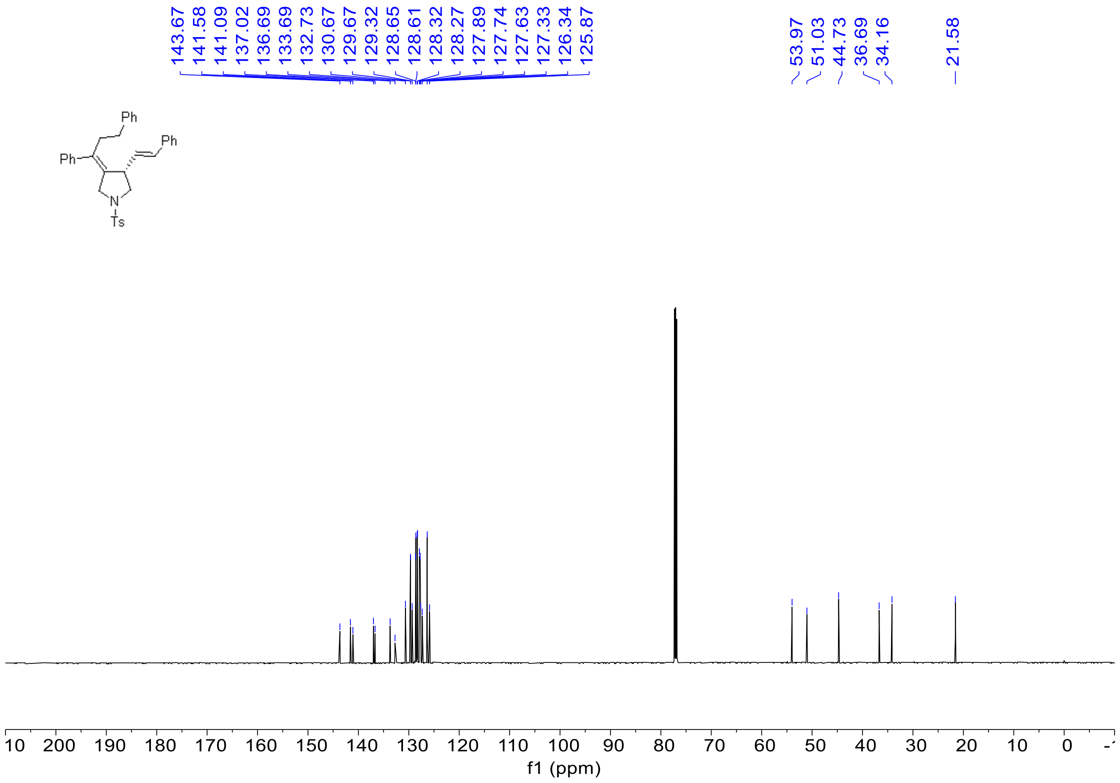

Supplementary Figure 258. ^{13}C NMR spectrum (151 MHz, $CDCl_3$) of **7aa**

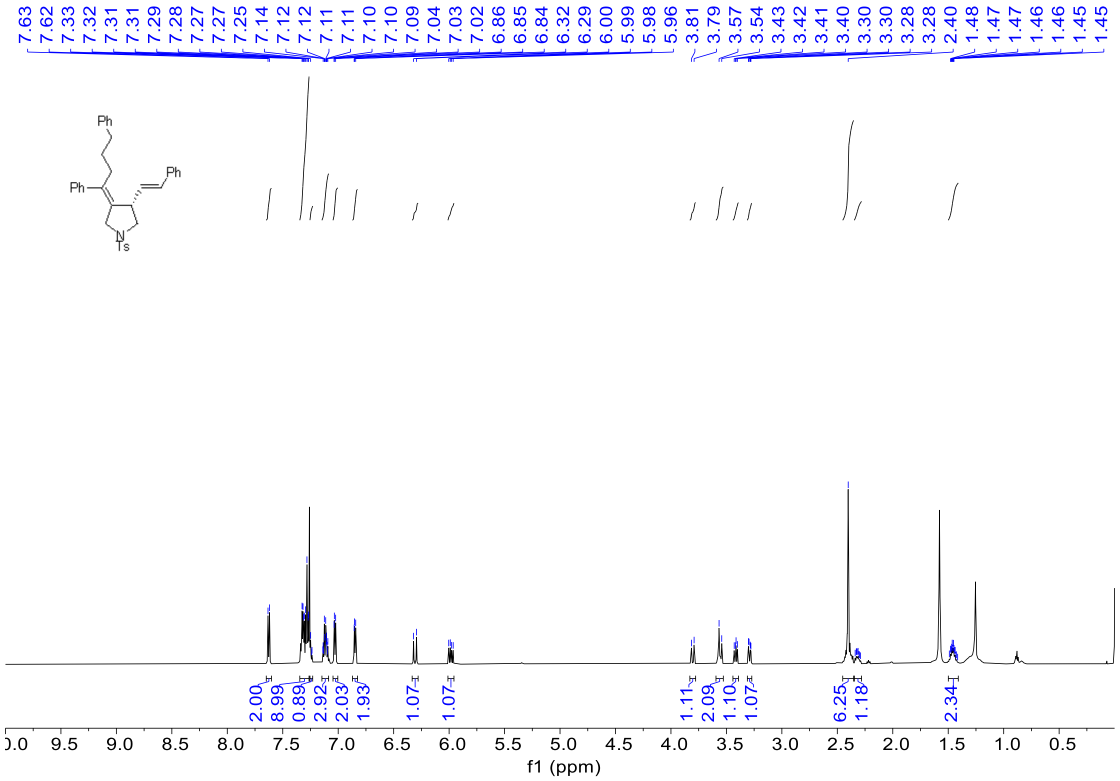

Supplementary Figure 259. ^{1}H NMR spectrum (600 MHz, $CDCl_3$) of **7ab**

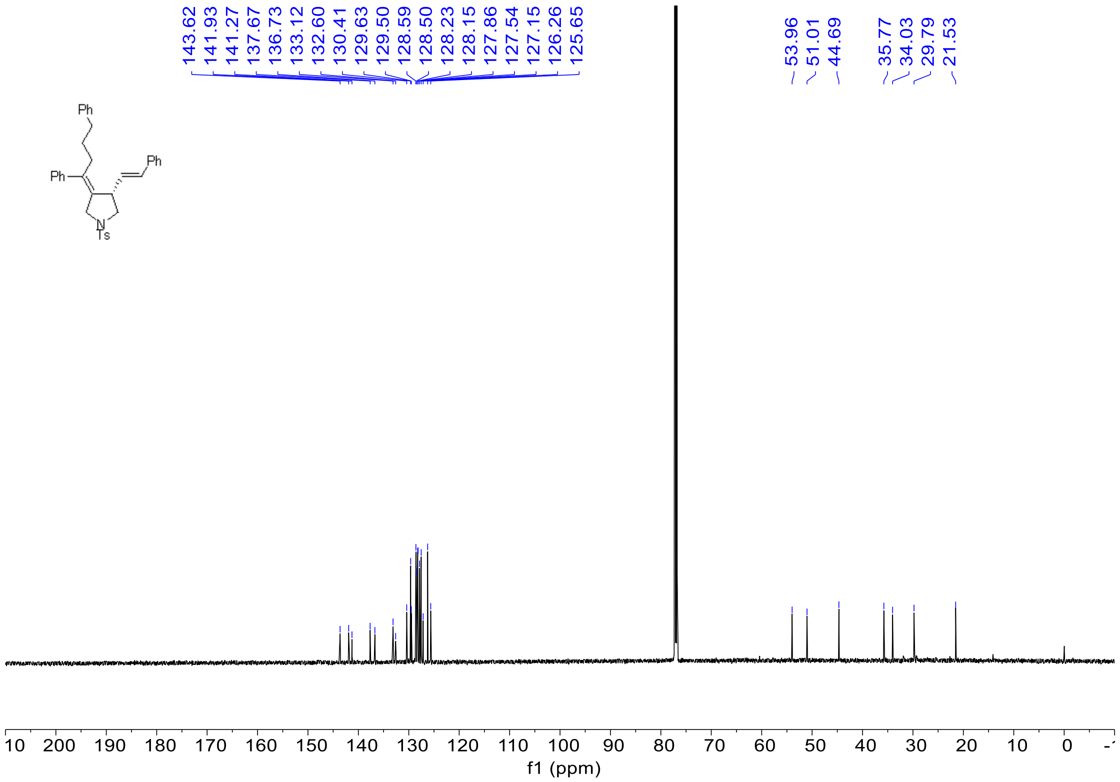

Supplementary Figure 260. ^{13}C NMR spectrum (151 MHz, $CDCl_3$) of **7ab**

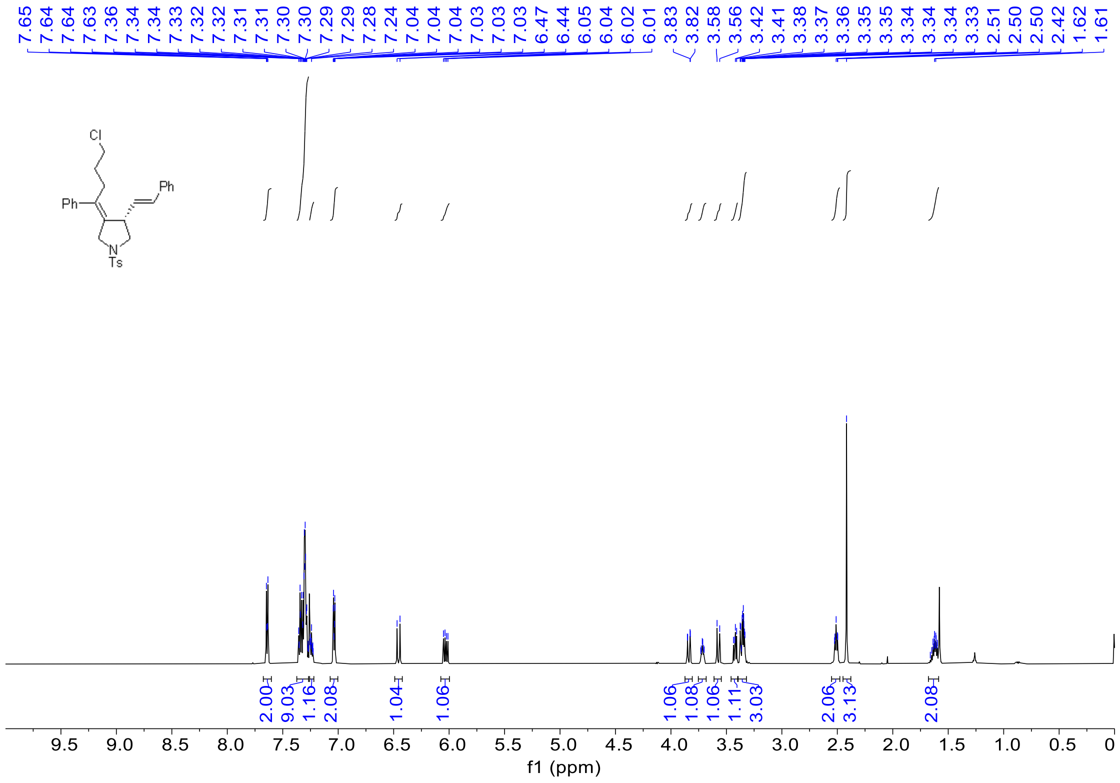

Supplementary Figure 261. ^{1}H NMR spectrum (600 MHz, $CDCl_3$) of **7ac**

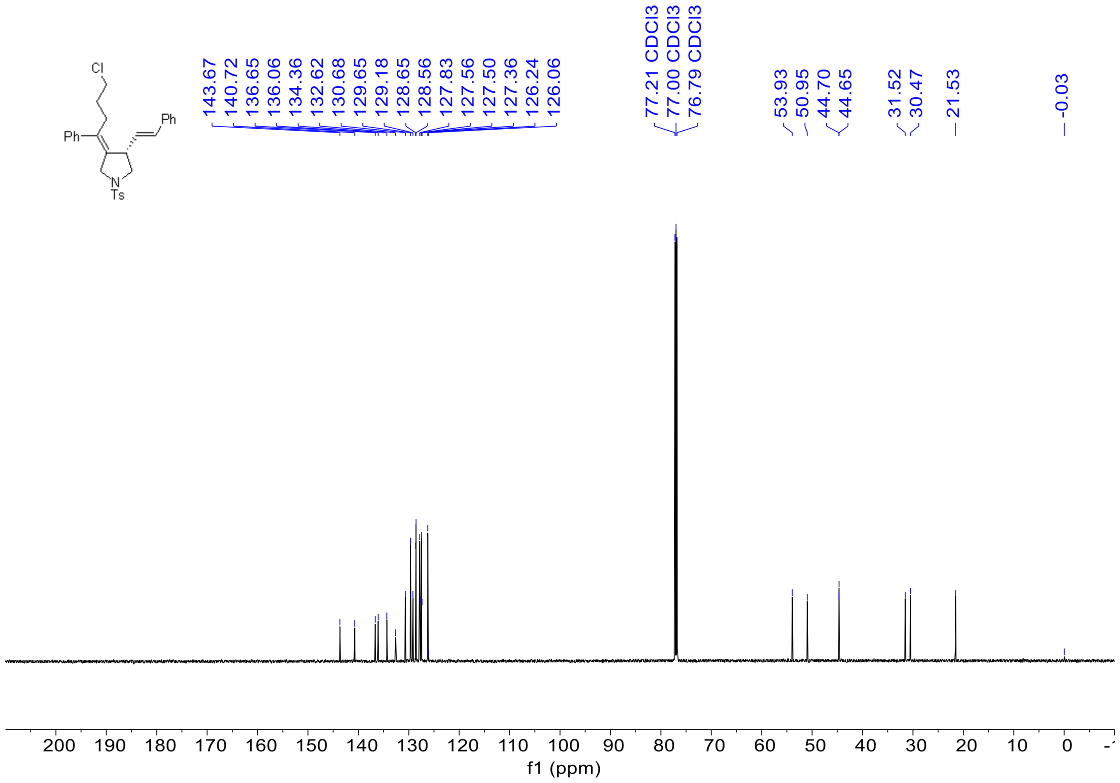

Supplementary Figure 262. ^{13}C NMR spectrum (151 MHz, $CDCl_3$) of **7ac**

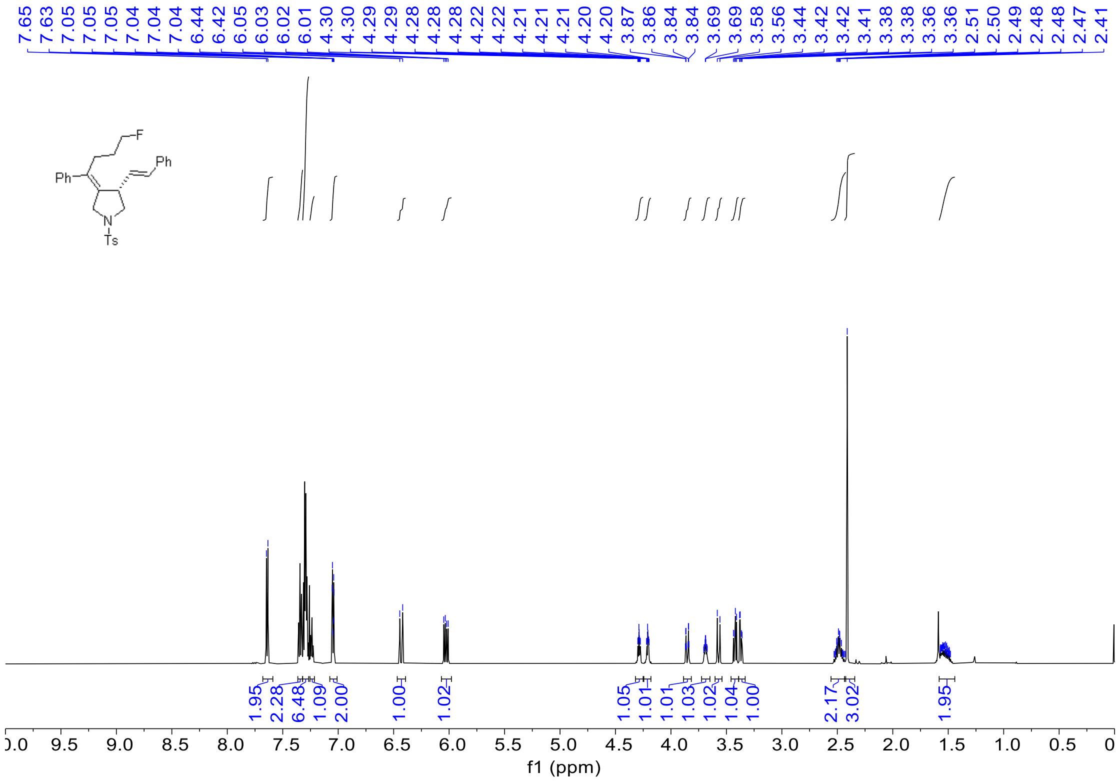

Supplementary Figure 263. ^{1}H NMR spectrum (600 MHz, $CDCl_3$) of **7ad**

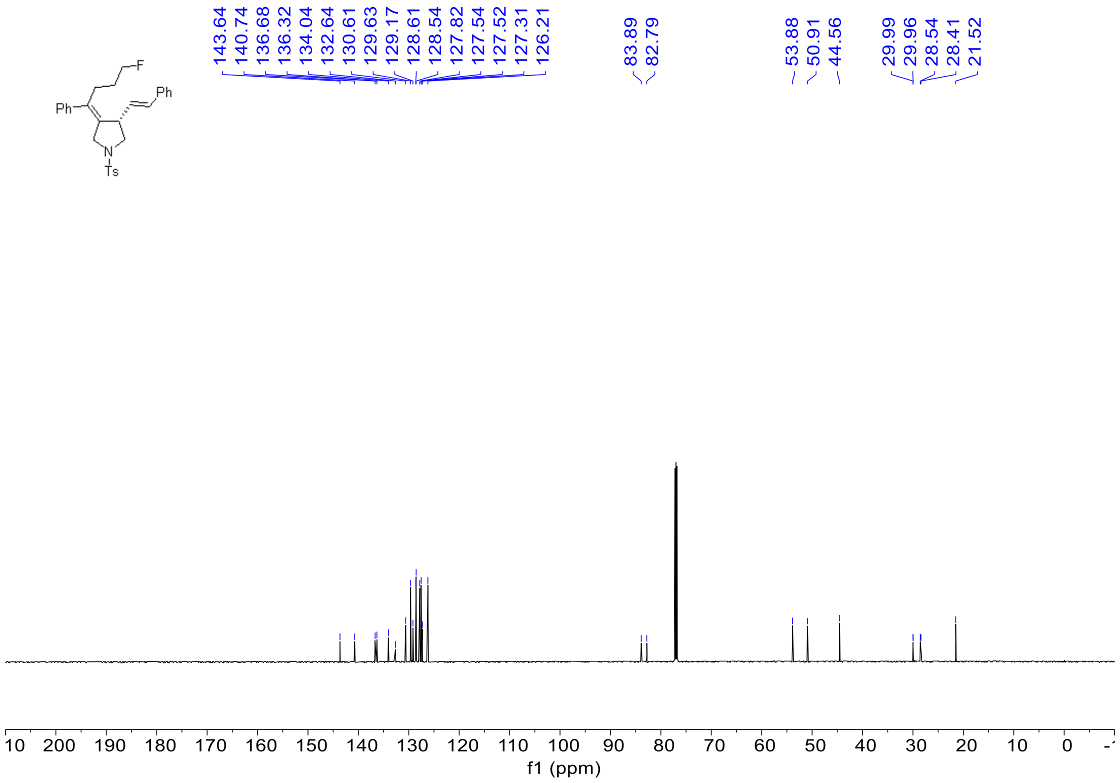

Supplementary Figure 264. ^{13}C NMR spectrum (151 MHz, $CDCl_3$) of **7ad**

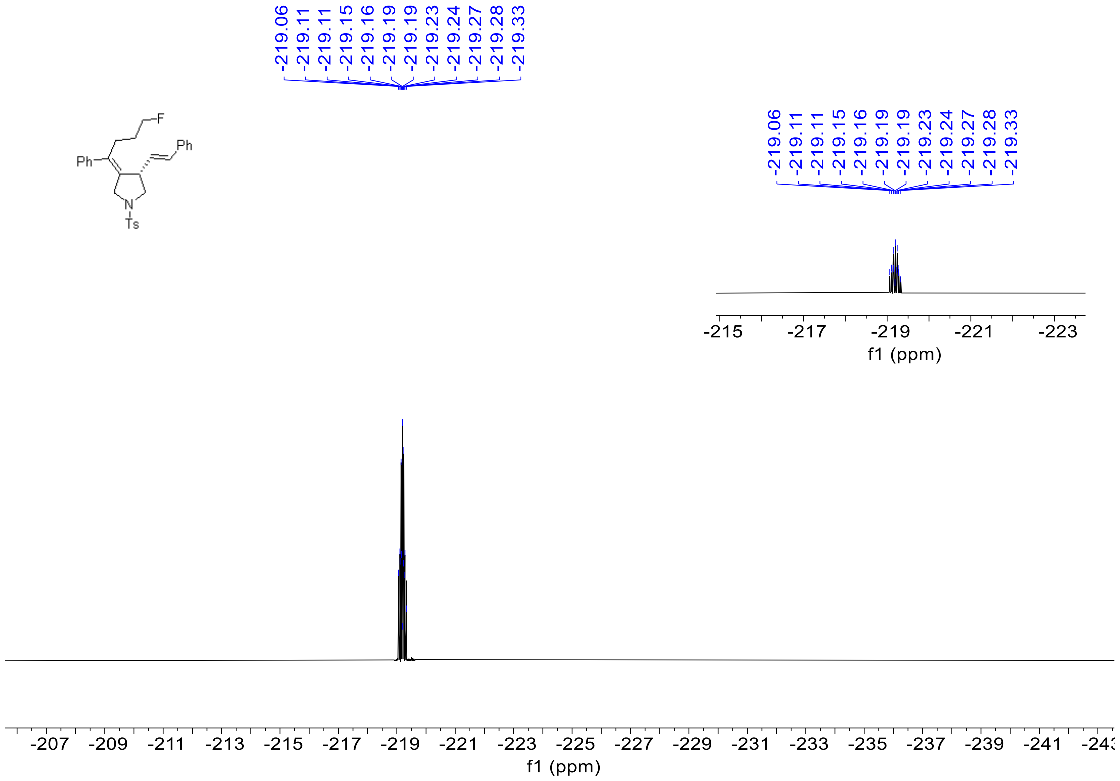

Supplementary Figure 265. ^{19}F NMR spectrum (565 MHz, $CDCl_3$) of **7ad**

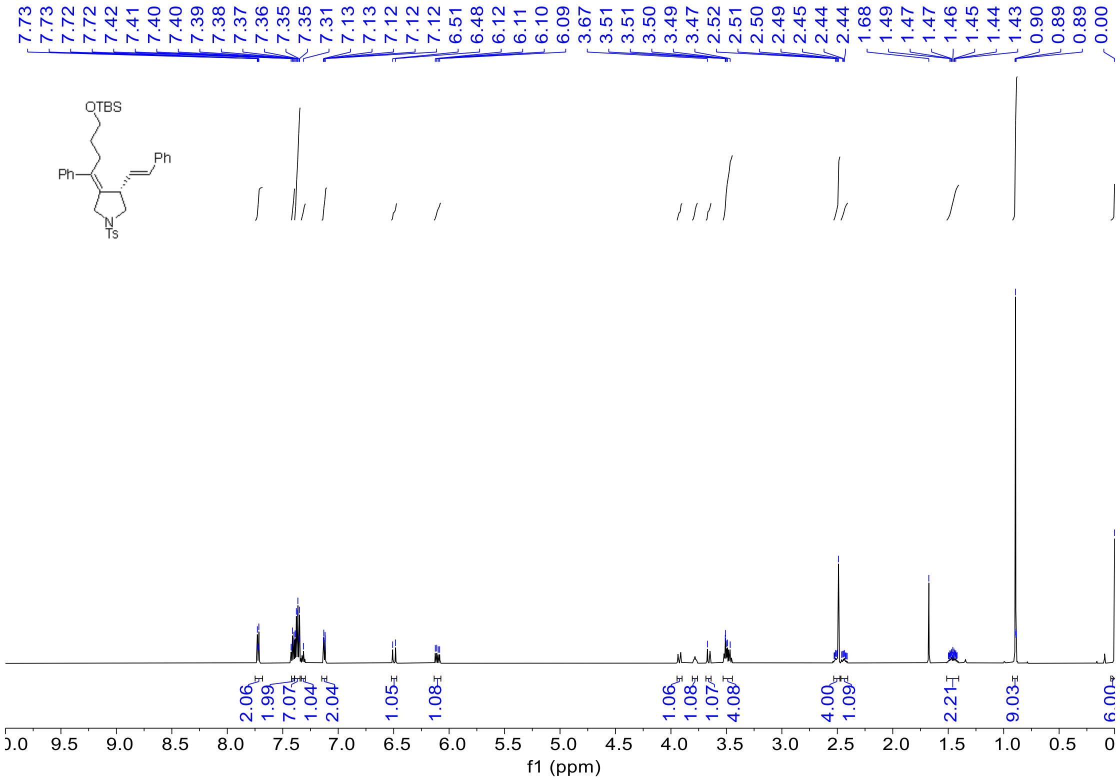

Supplementary Figure 266. ^{1}H NMR spectrum (600 MHz, $CDCl_3$) of **7ae**

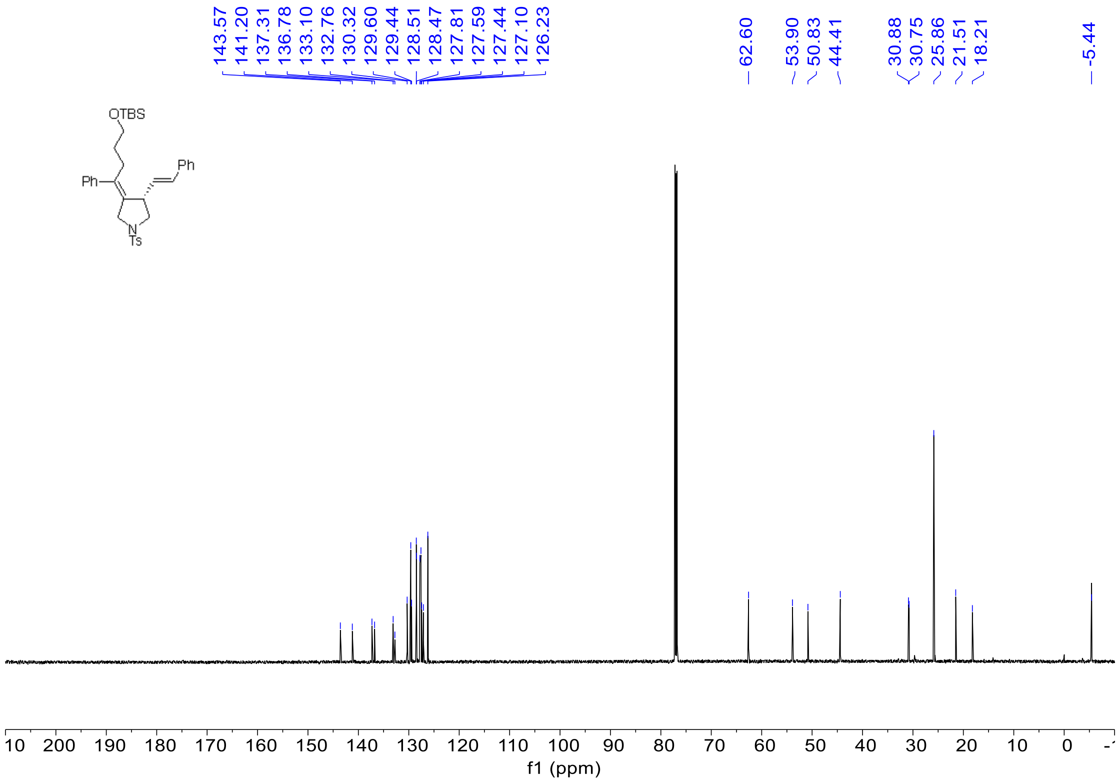

Supplementary Figure 267. ^{13}C NMR spectrum (151 MHz, $CDCl_3$) of **7ae**

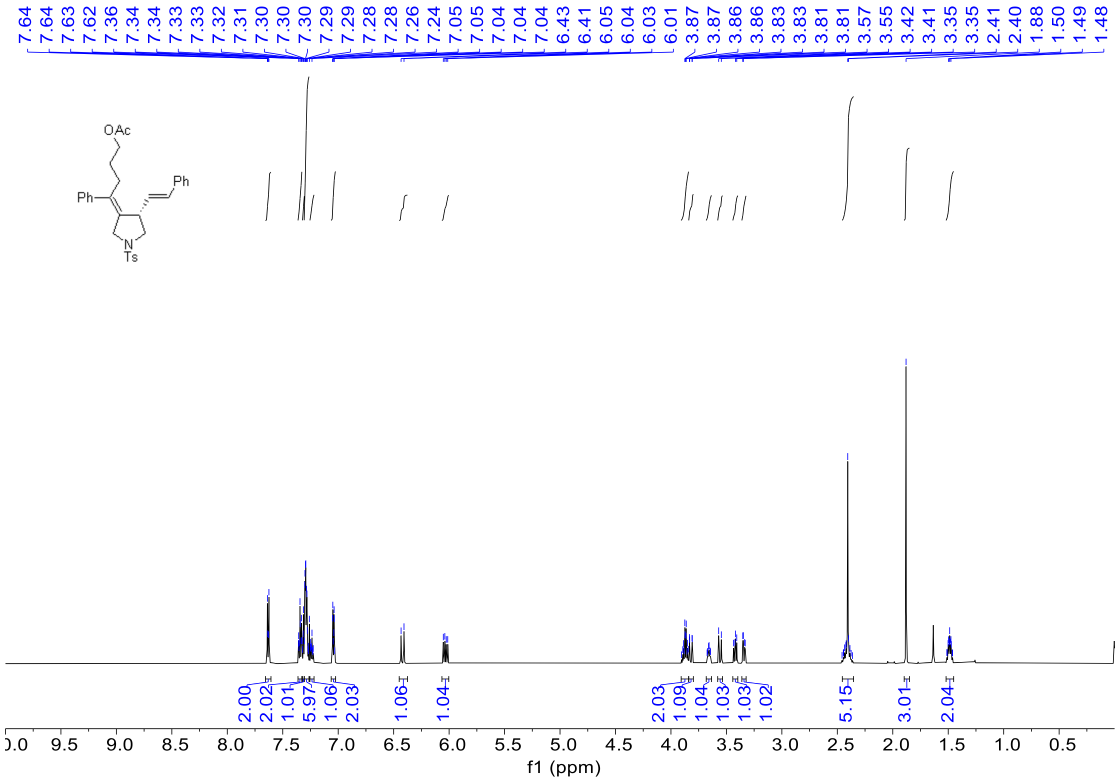

Supplementary Figure 268. ^{1}H NMR spectrum (600 MHz, $CDCl_3$) of **7af**

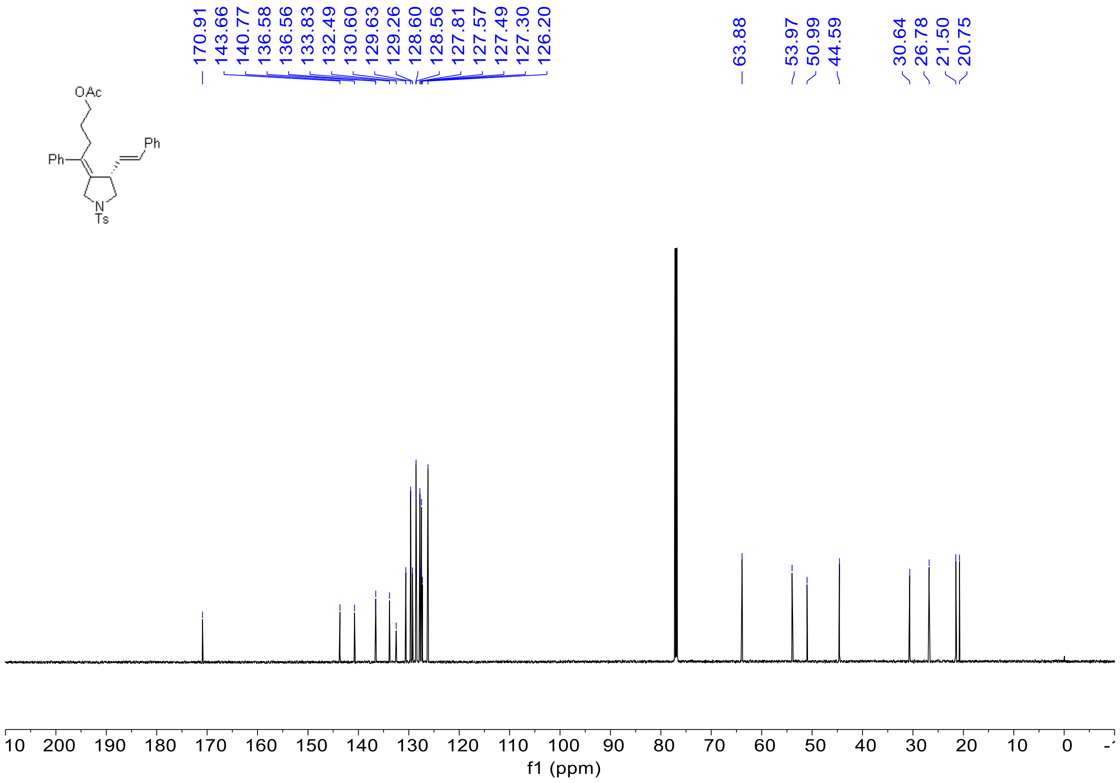

Supplementary Figure 269. ^{13}C NMR spectrum (151 MHz, $CDCl_3$) of **7af**

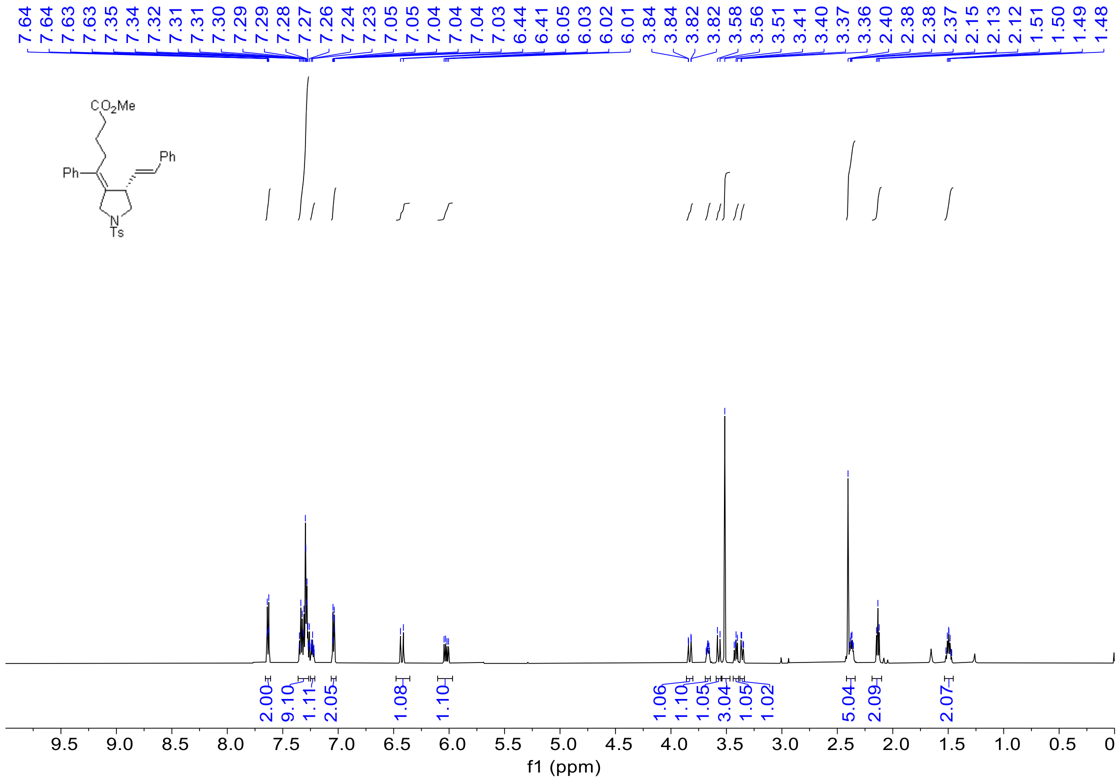

Supplementary Figure 270. ^{1}H NMR spectrum (600 MHz, $CDCl_3$) of **7ag**

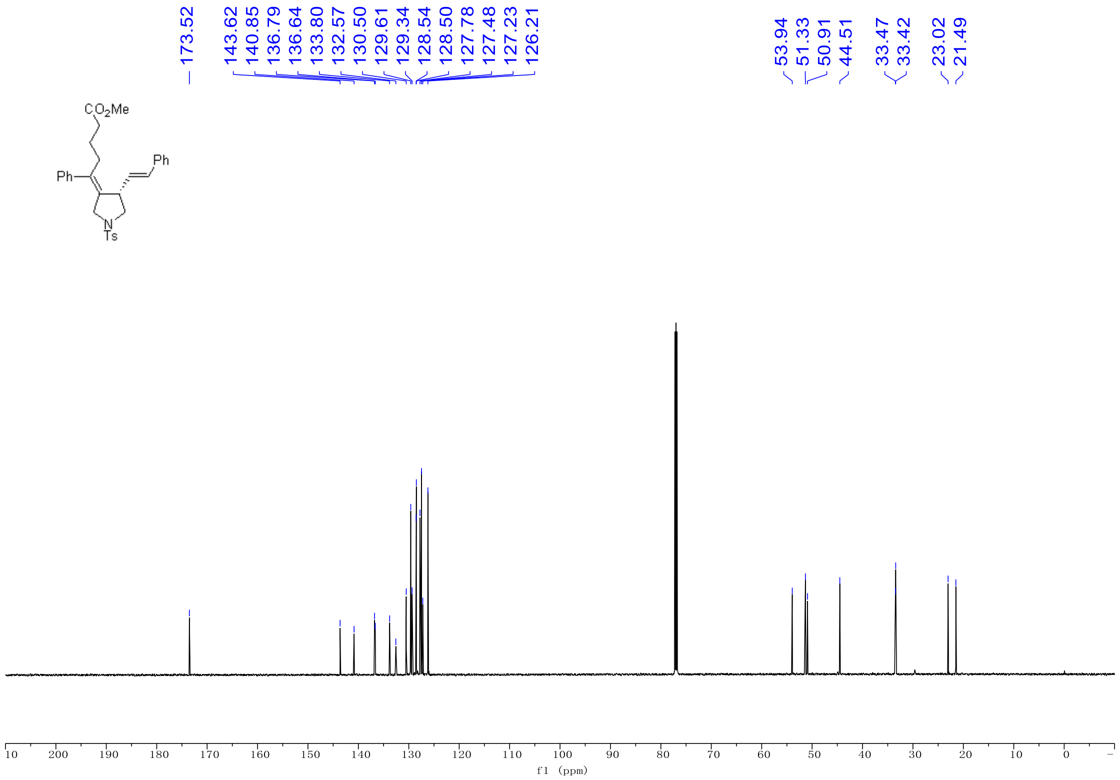

Supplementary Figure 271. ^{13}C NMR spectrum (151 MHz, $CDCl_3$) of **7ag**

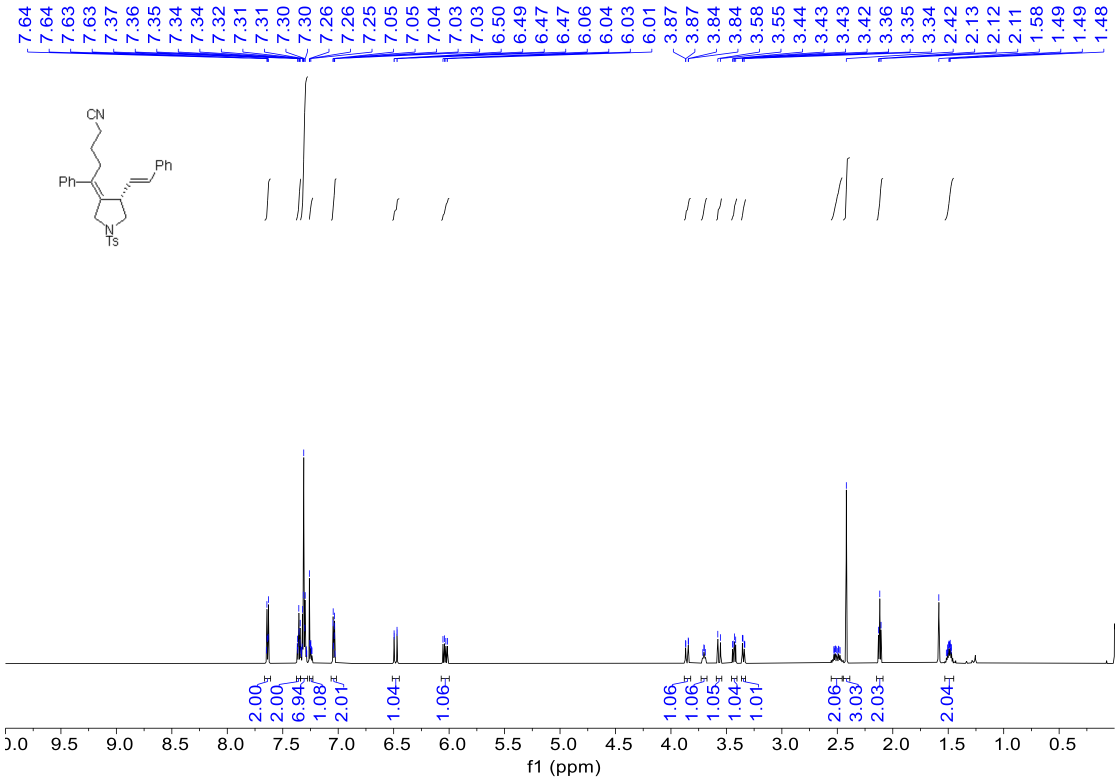

Supplementary Figure 272. ^{1}H NMR spectrum (600 MHz, $CDCl_3$) of **7ah**

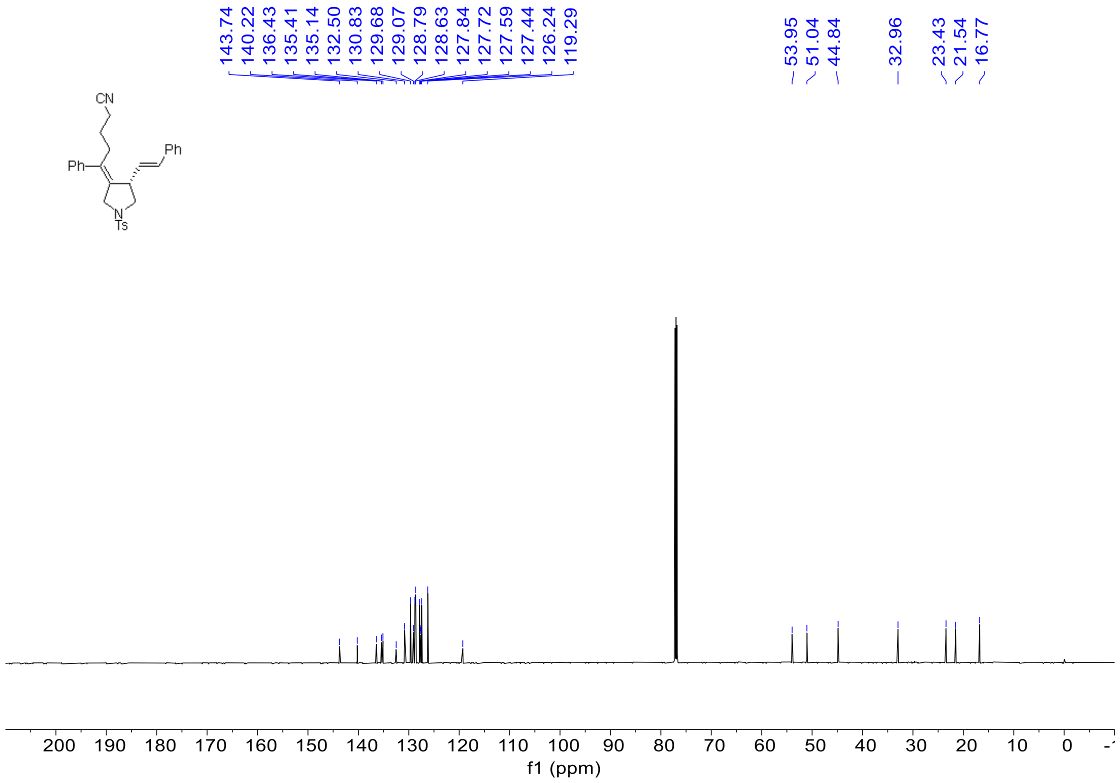

Supplementary Figure 273. ^{13}C NMR spectrum (151 MHz, $CDCl_3$) of **7ah**

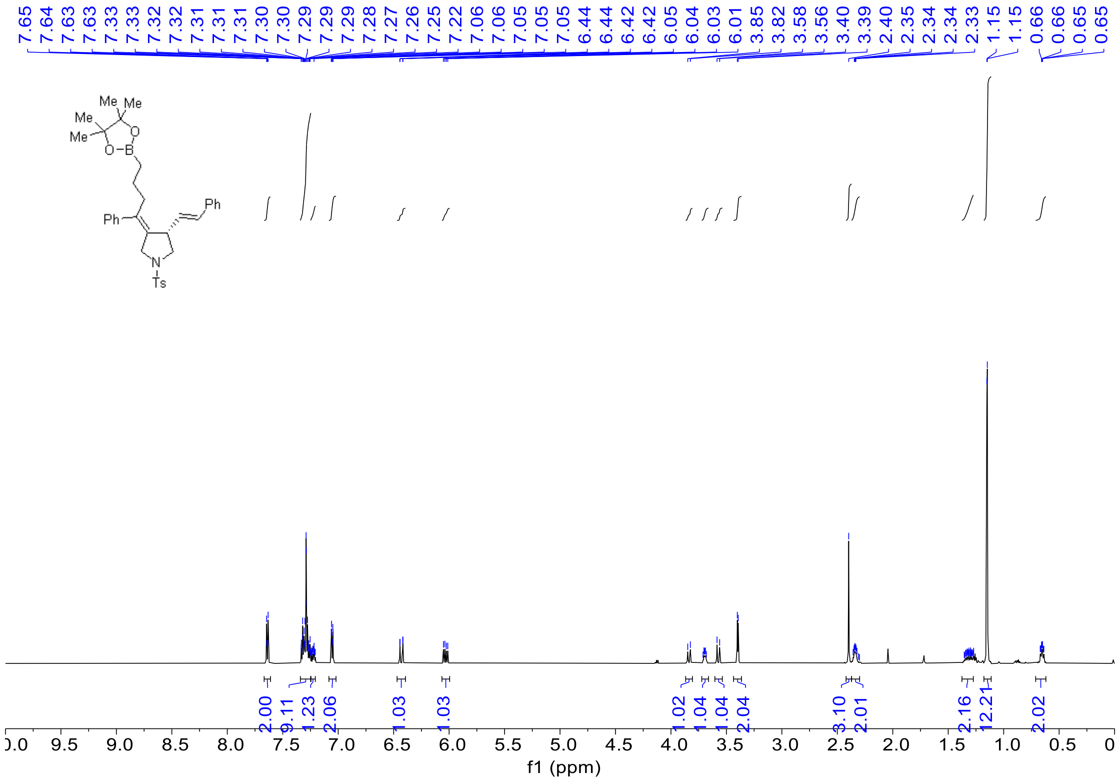

Supplementary Figure 274. ^{1}H NMR spectrum (600 MHz, $CDCl_3$) of **7ai**

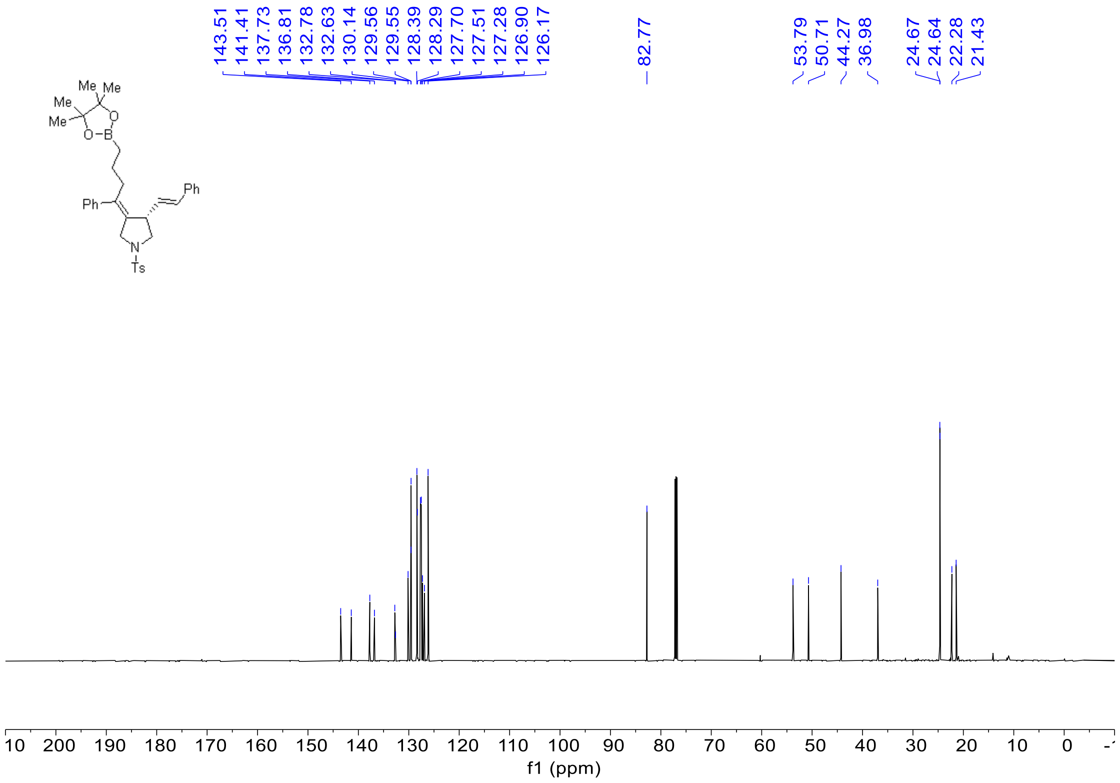

Supplementary Figure 275. ^{13}C NMR spectrum (151 MHz, $CDCl_3$) of **7ai**

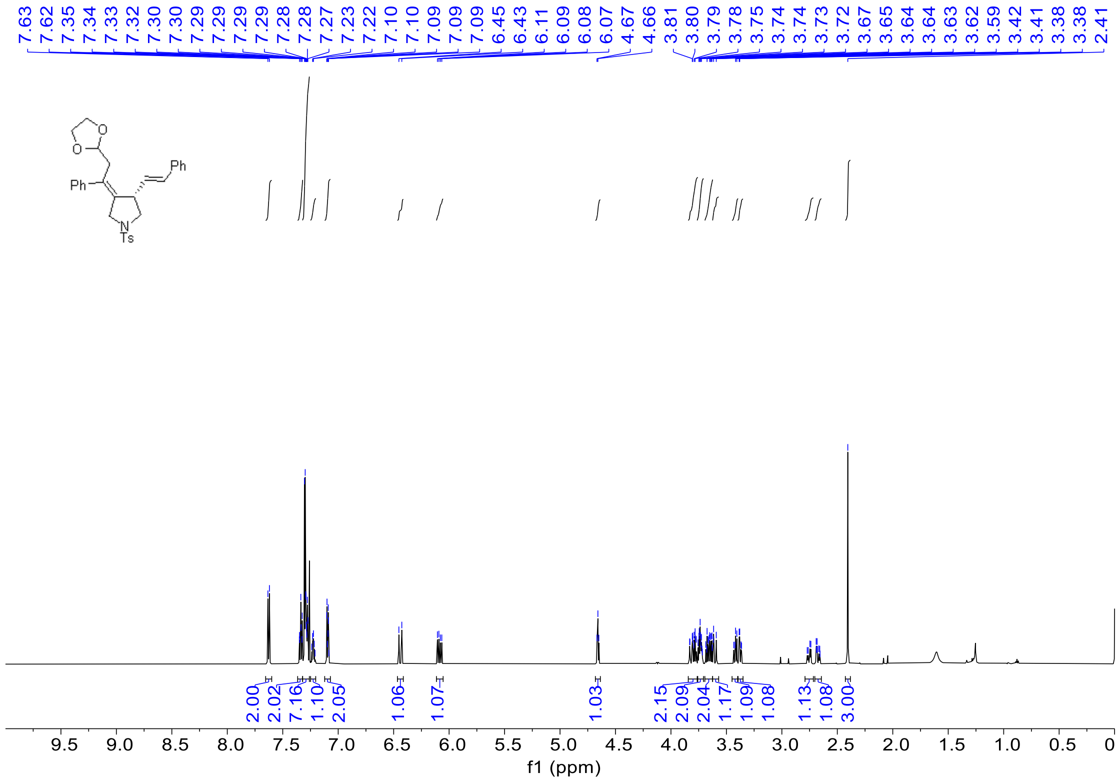

Supplementary Figure 276. ^{1}H NMR spectrum (600 MHz, $CDCl_3$) of **7aj**

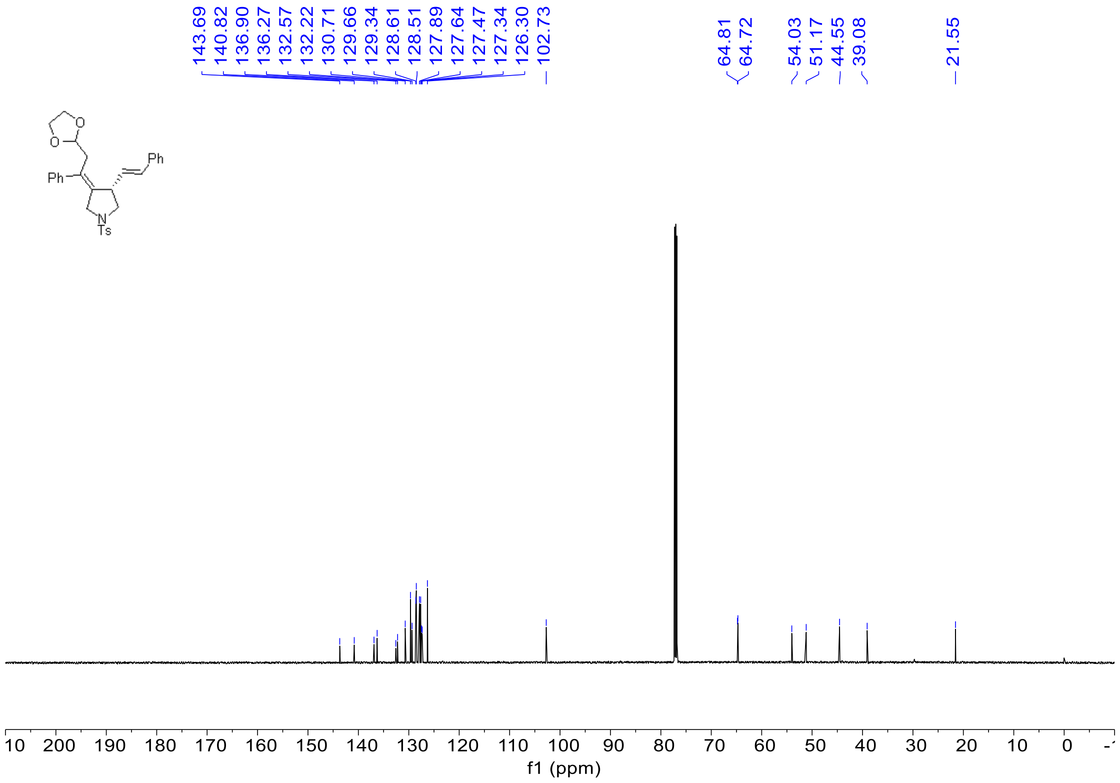

Supplementary Figure 277. ^{13}C NMR spectrum (151 MHz, $CDCl_3$) of **7aj**

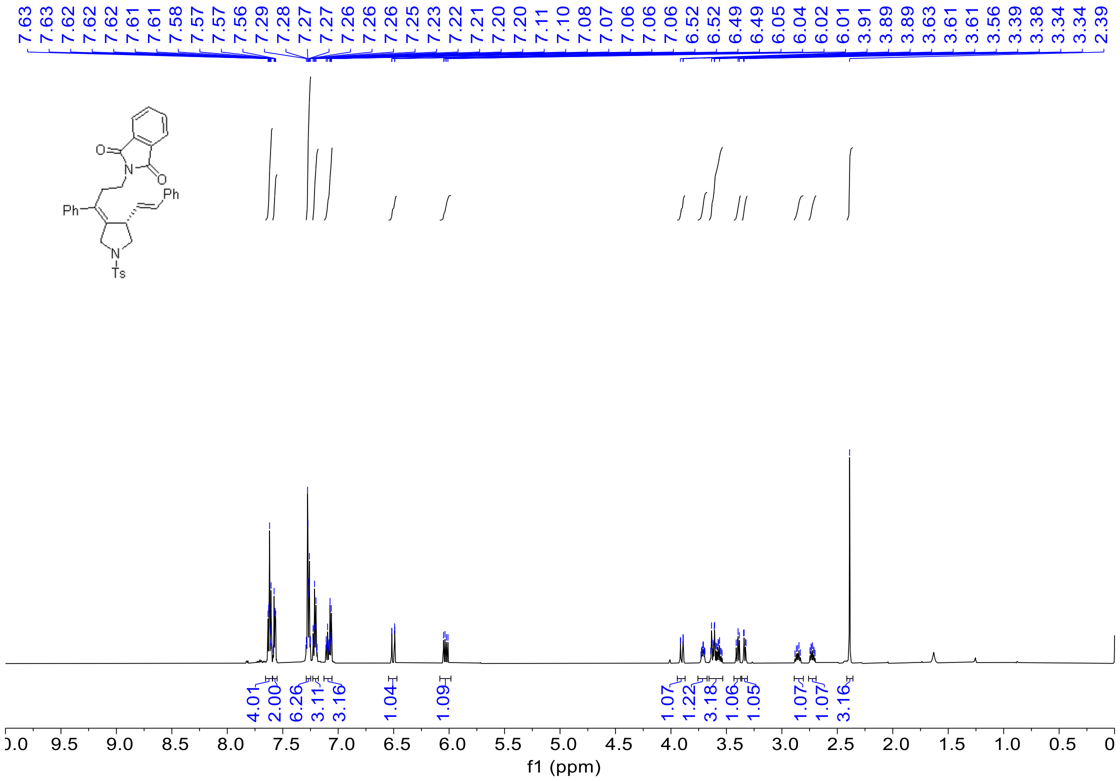

Supplementary Figure 278. ^{1}H NMR spectrum (600 MHz, $CDCl_3$) of **7ak**

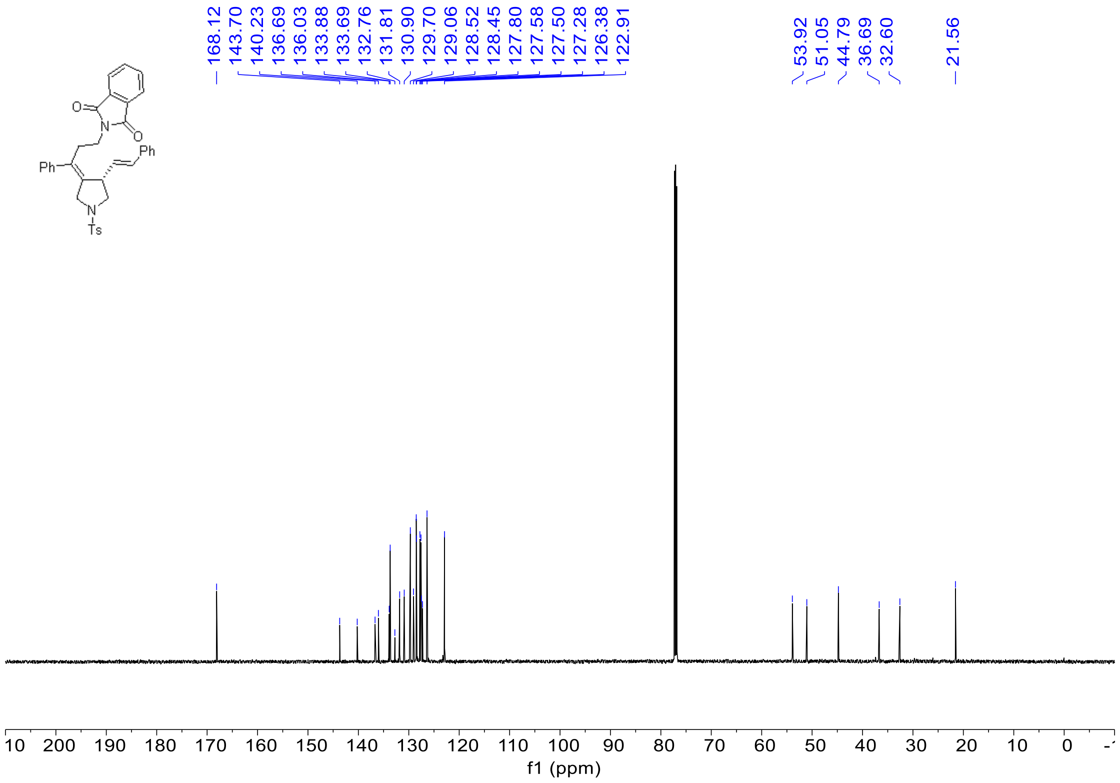

Supplementary Figure 279. ^{13}C NMR spectrum (151 MHz, $CDCl_3$) of **7ak**

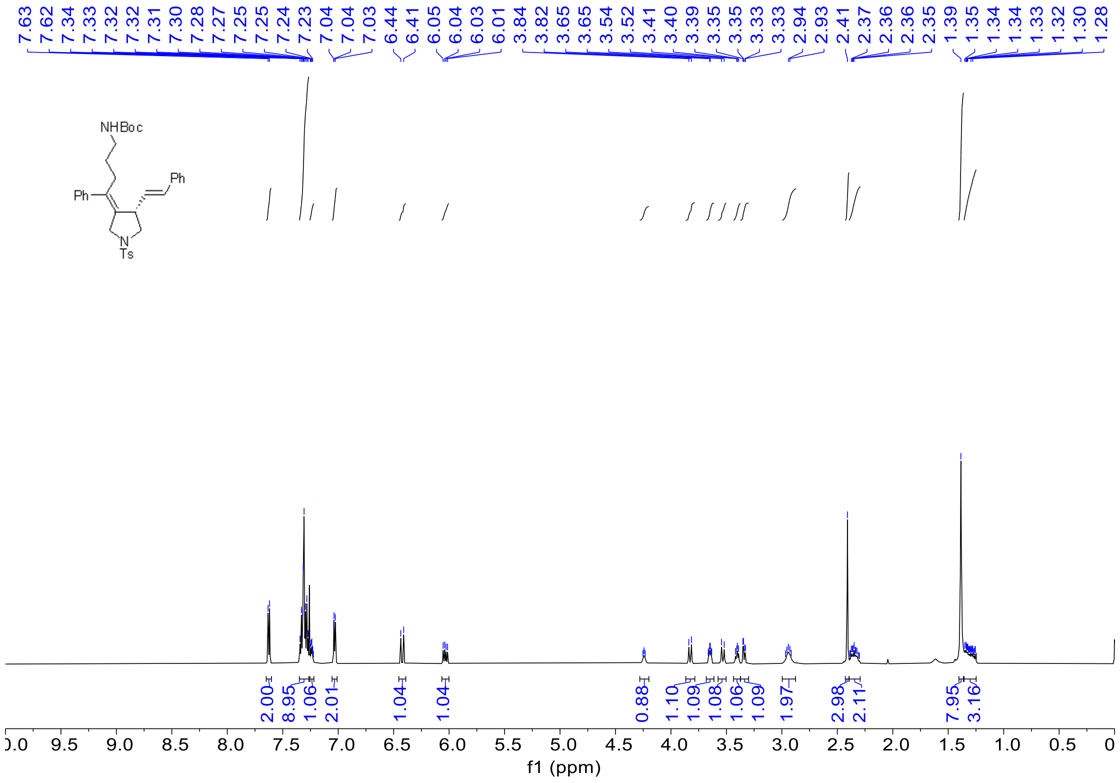

Supplementary Figure 280. ^{1}H NMR spectrum (600 MHz, $CDCl_3$) of **7al**

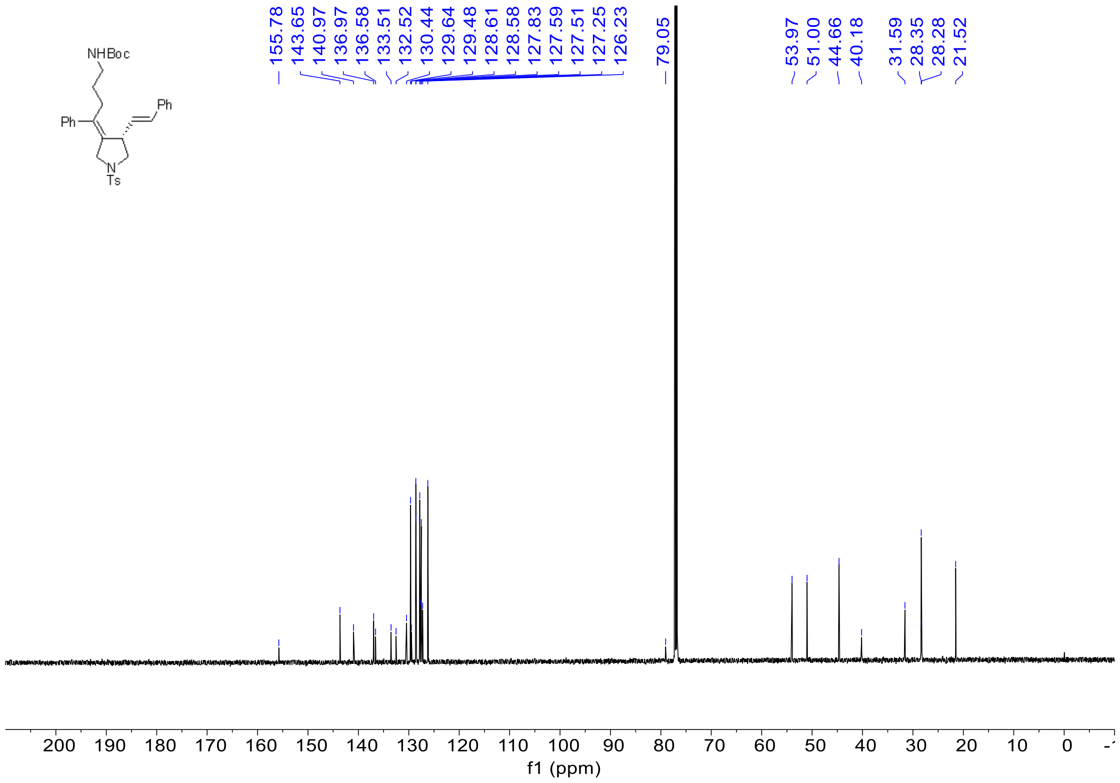

Supplementary Figure 281. ^{13}C NMR spectrum (151 MHz, $CDCl_3$) of **7al**

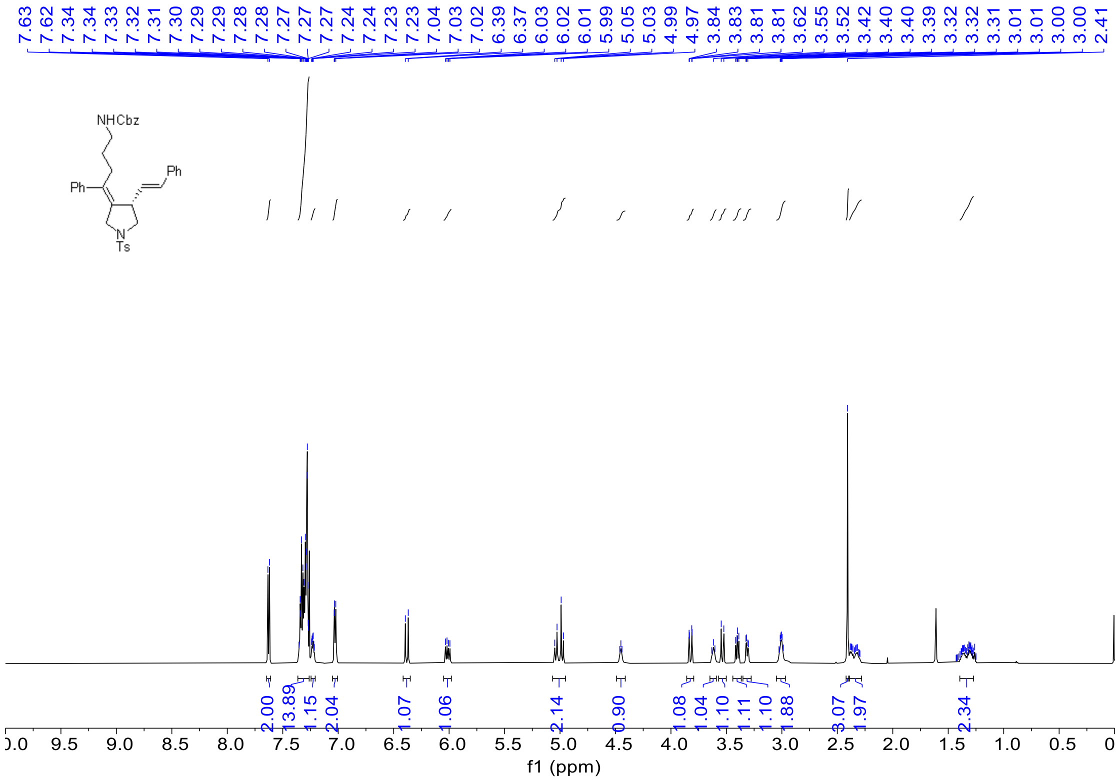

Supplementary Figure 282. ^{1}H NMR spectrum (600 MHz, $CDCl_3$) of **7am**

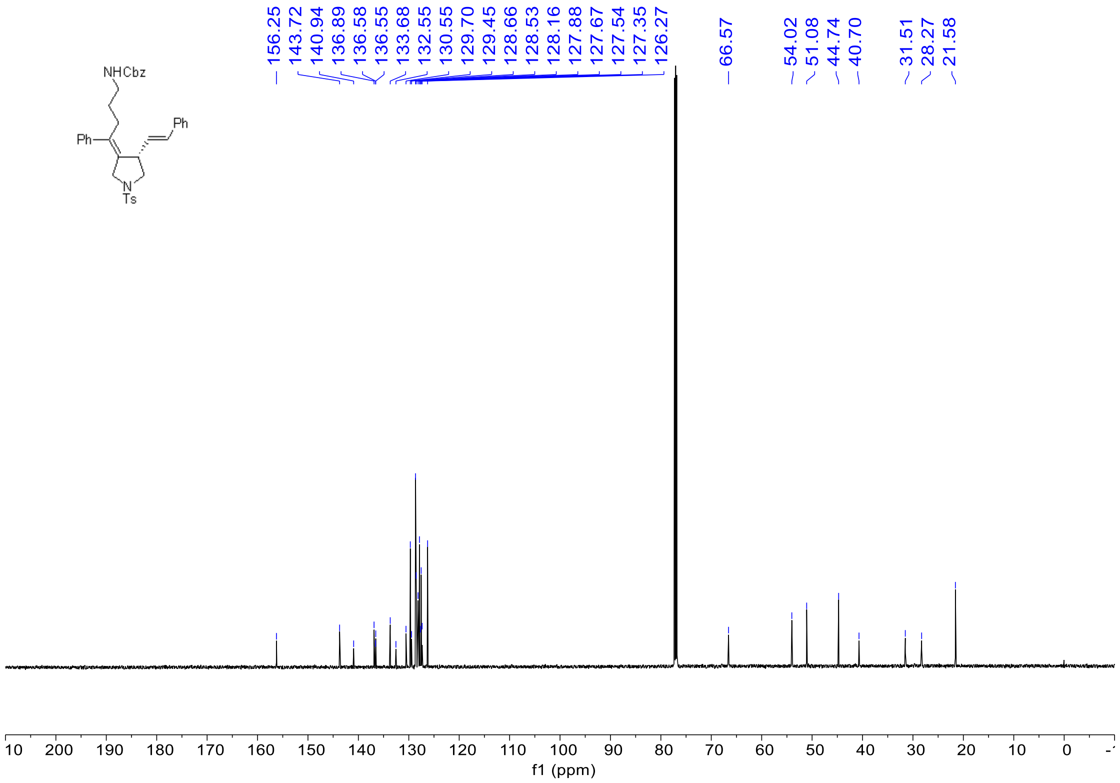

Supplementary Figure 283. ^{13}C NMR spectrum (151 MHz, $CDCl_3$) of **7am**

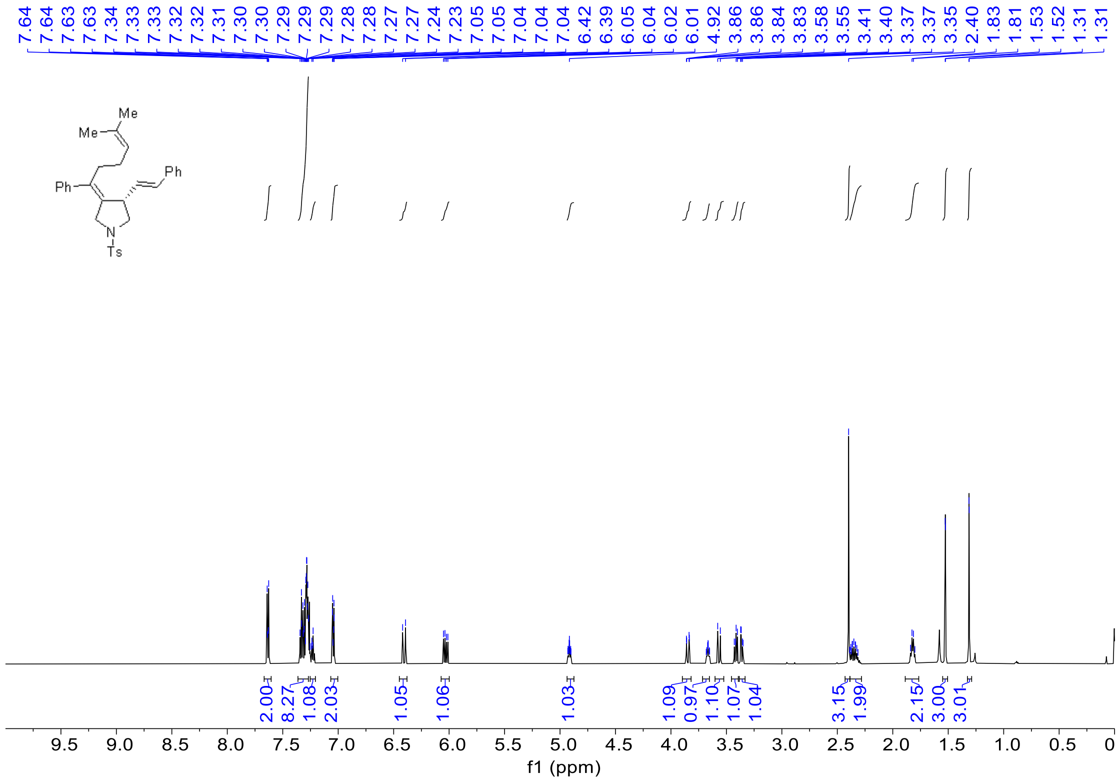

Supplementary Figure 284. ^{1}H NMR spectrum (600 MHz, $CDCl_3$) of **7an**

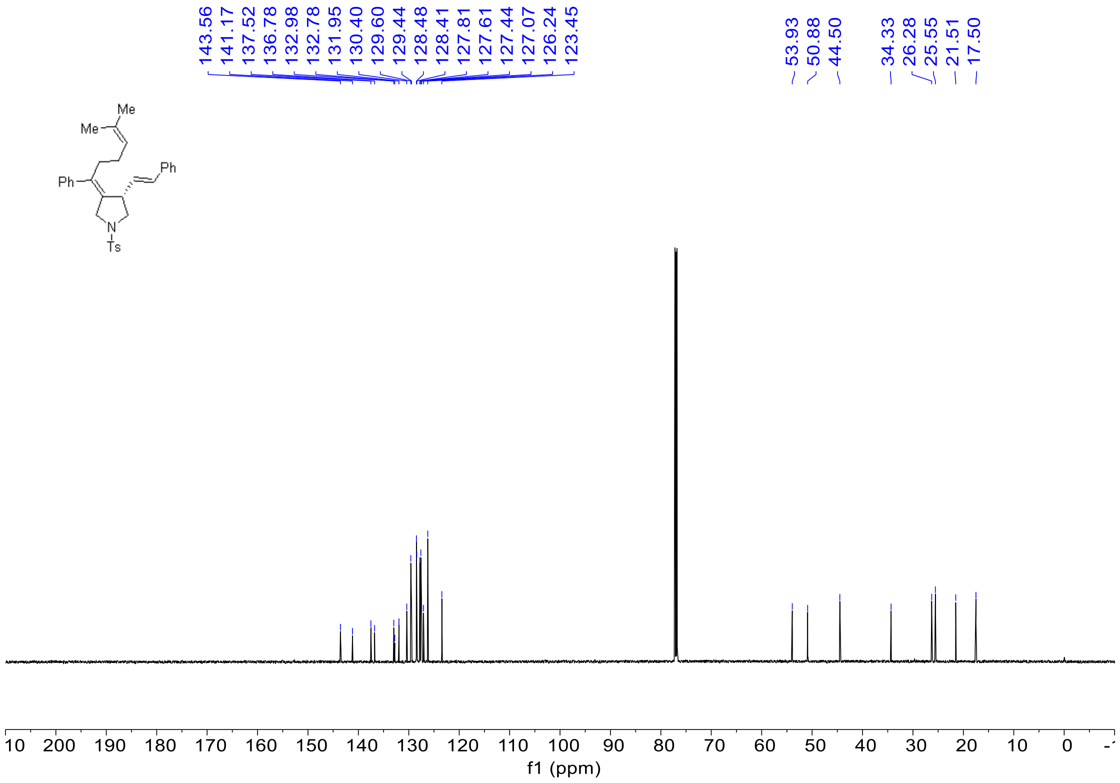

Supplementary Figure 285. ^{13}C NMR spectrum (151 MHz, $CDCl_3$) of **7an**

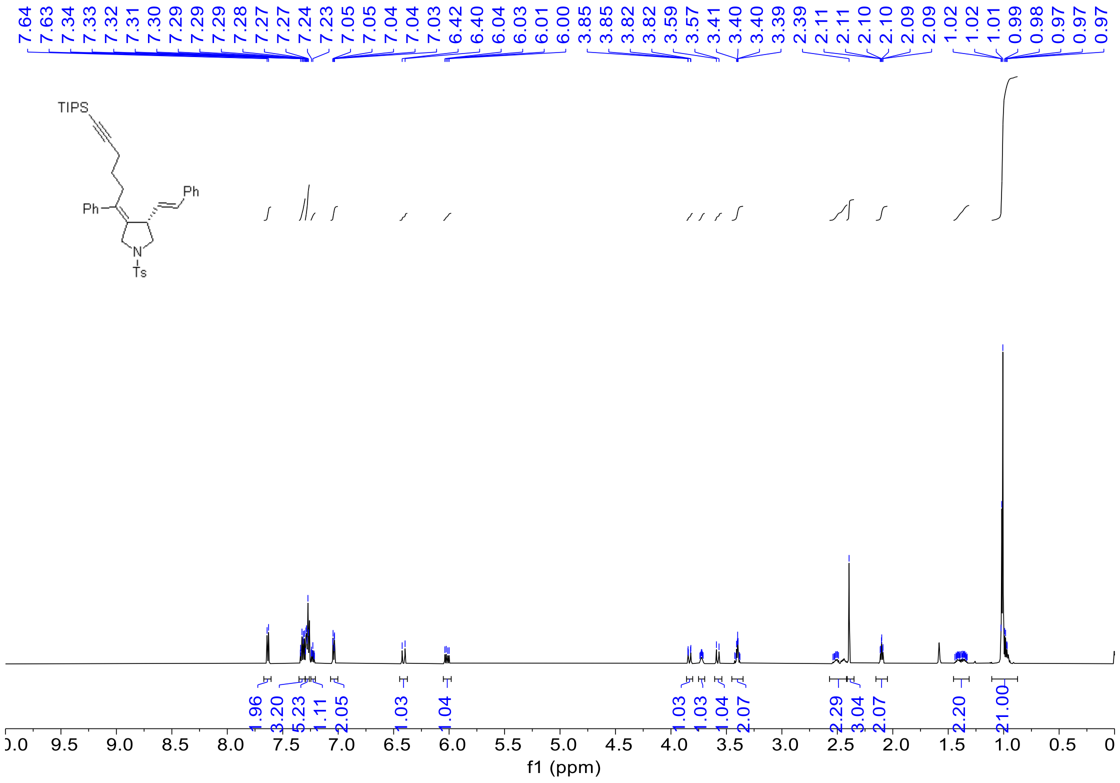

Supplementary Figure 286. ^{1}H NMR spectrum (600 MHz, $CDCl_3$) of **7ao**

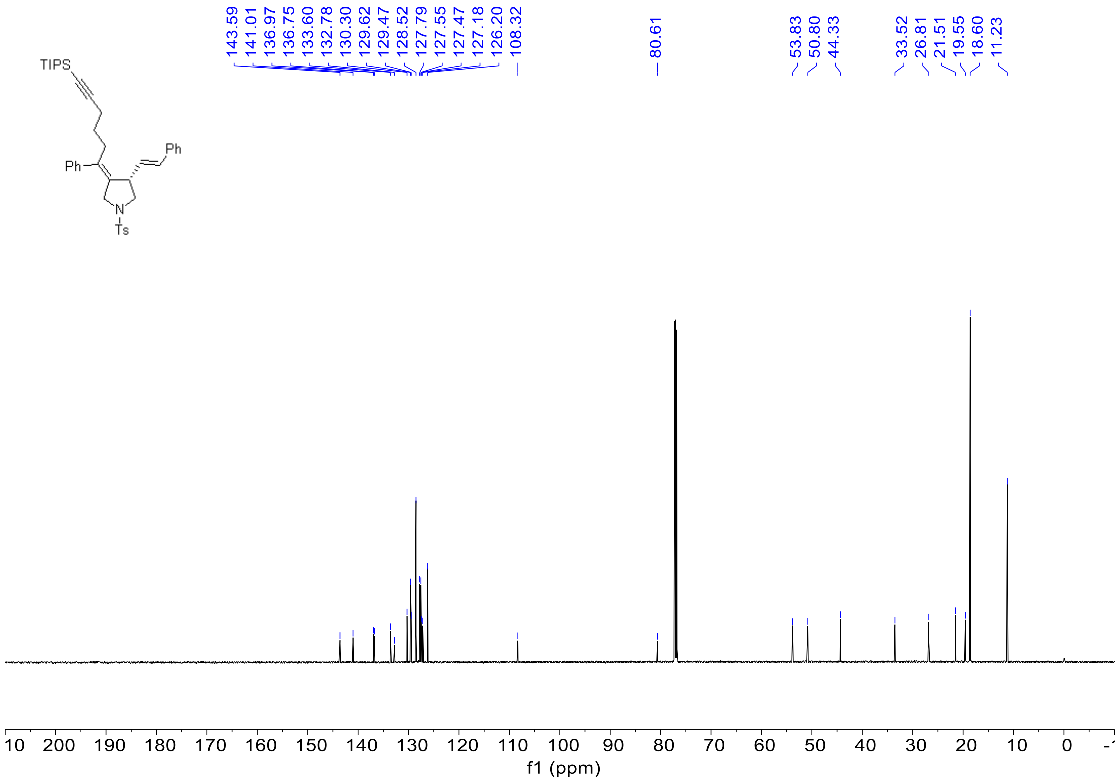

Supplementary Figure 287. ^{13}C NMR spectrum (151 MHz, $CDCl_3$) of **7ao**

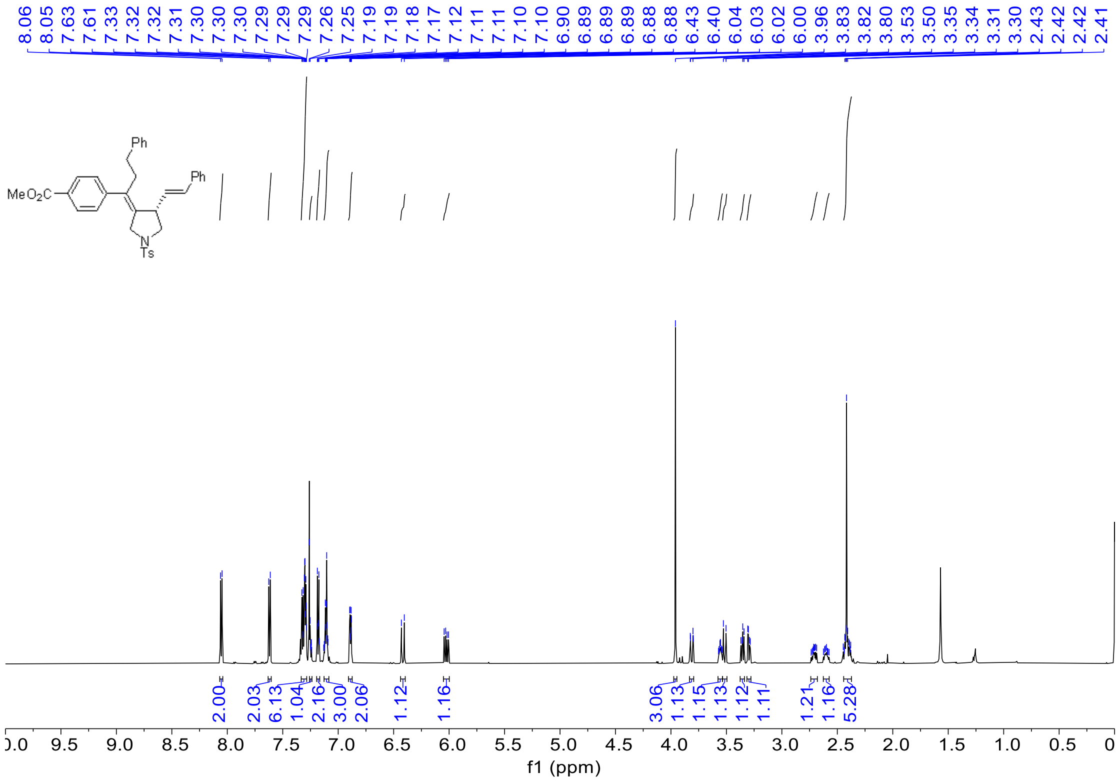

Supplementary Figure 288. ^{1}H NMR spectrum (600 MHz, $CDCl_3$) of **7ba**

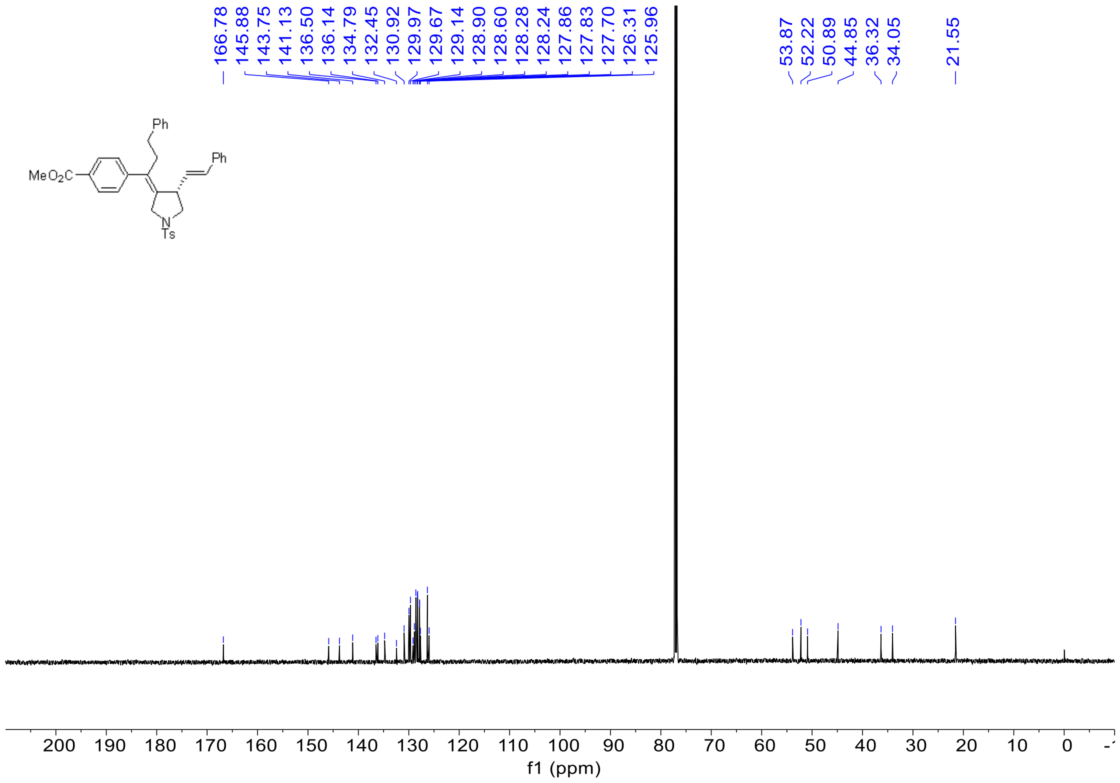

Supplementary Figure 289. ^{13}C NMR spectrum (151 MHz, $CDCl_3$) of **7ba**

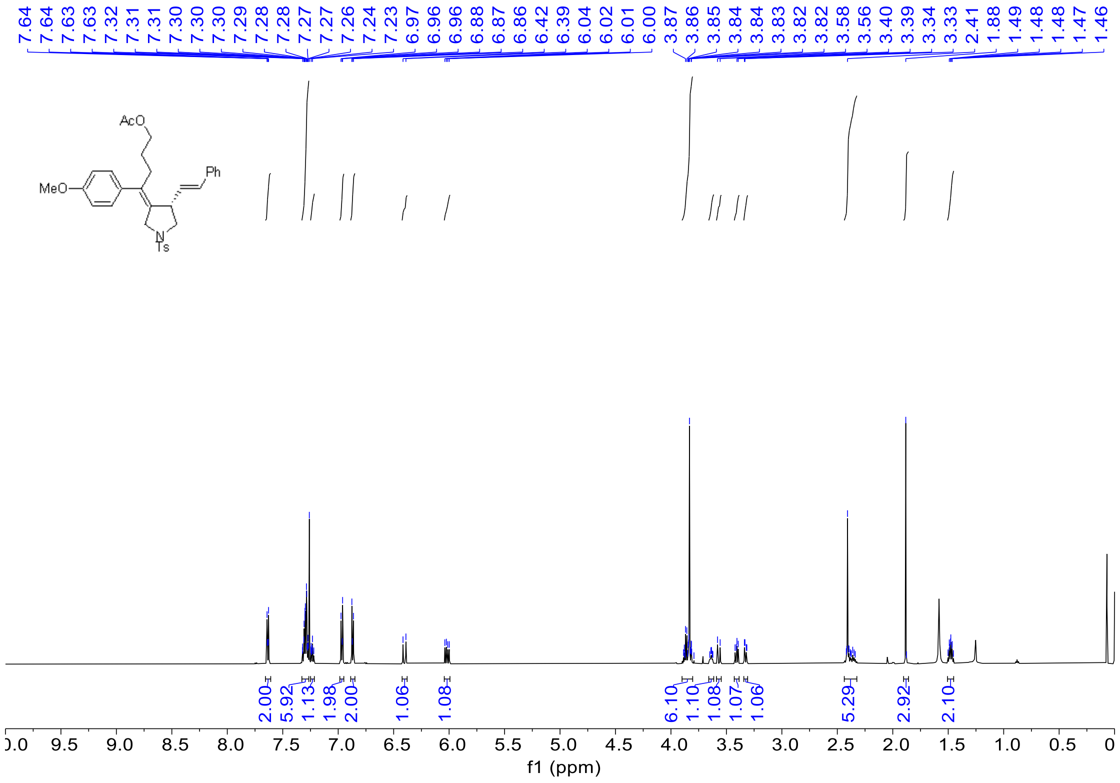

Supplementary Figure 290. ^{1}H NMR spectrum (600 MHz, $CDCl_3$) of **7cf**

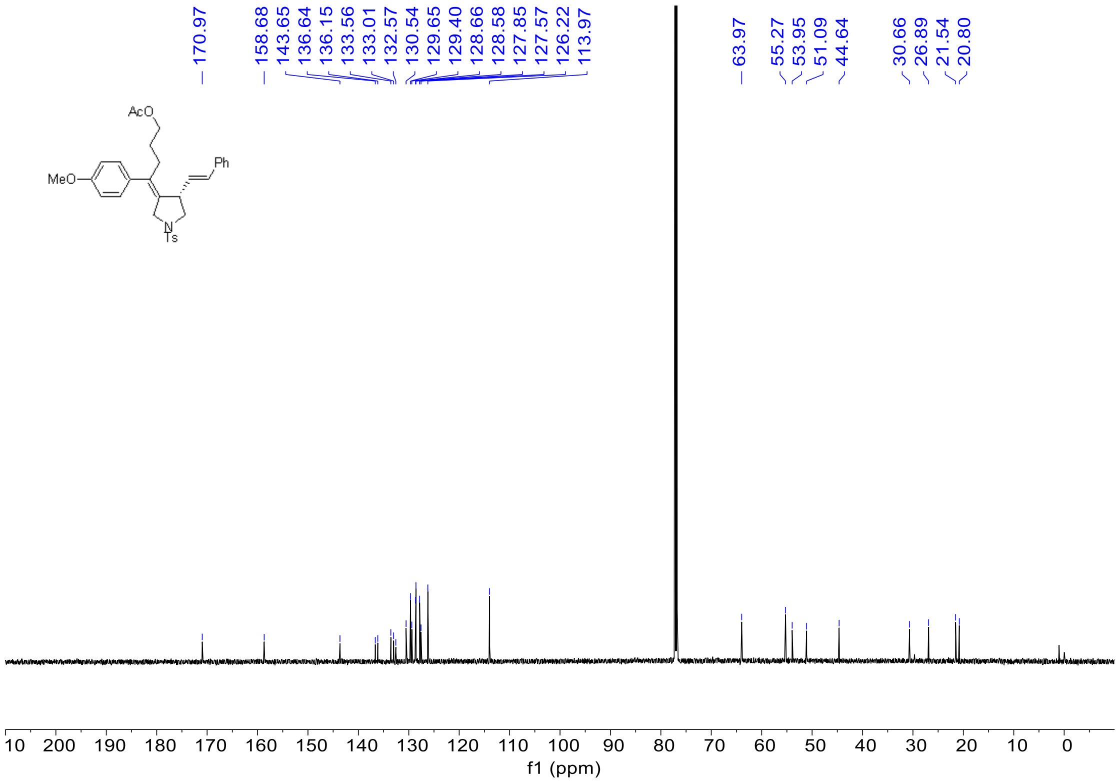

Supplementary Figure 291. ^{13}C NMR spectrum (151 MHz, $CDCl_3$) of **7cf**

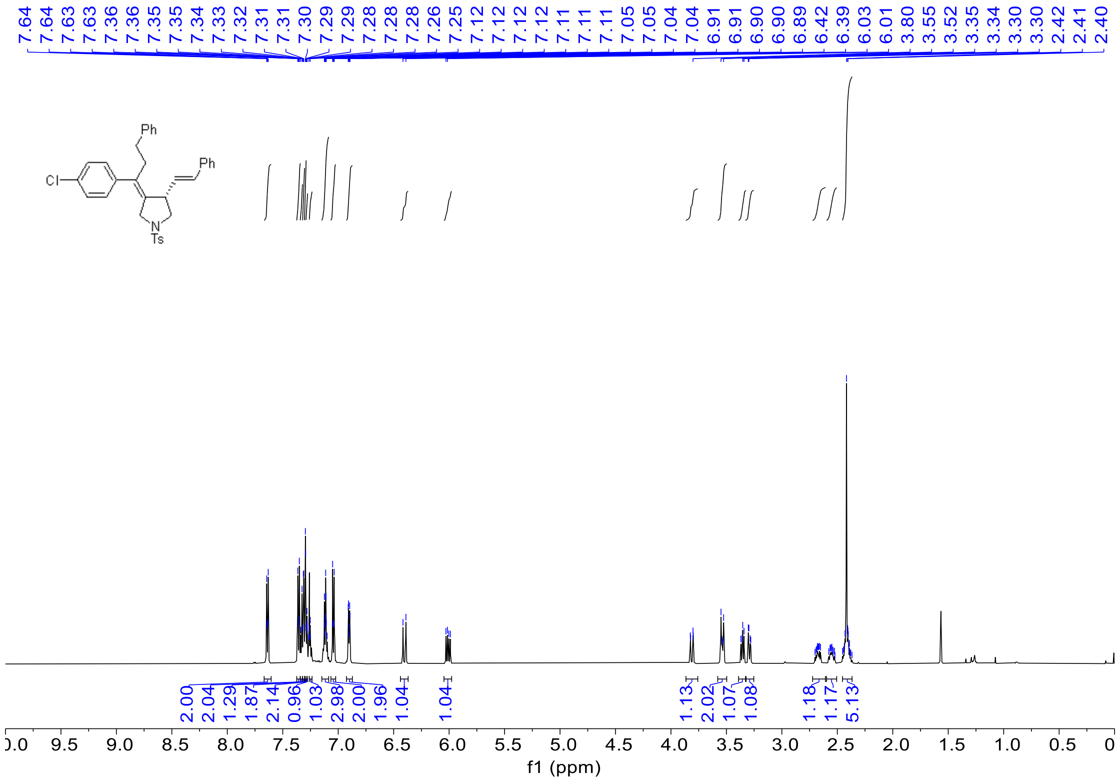

Supplementary Figure 292. ^{1}H NMR spectrum (600 MHz, $CDCl_3$) of **7da**

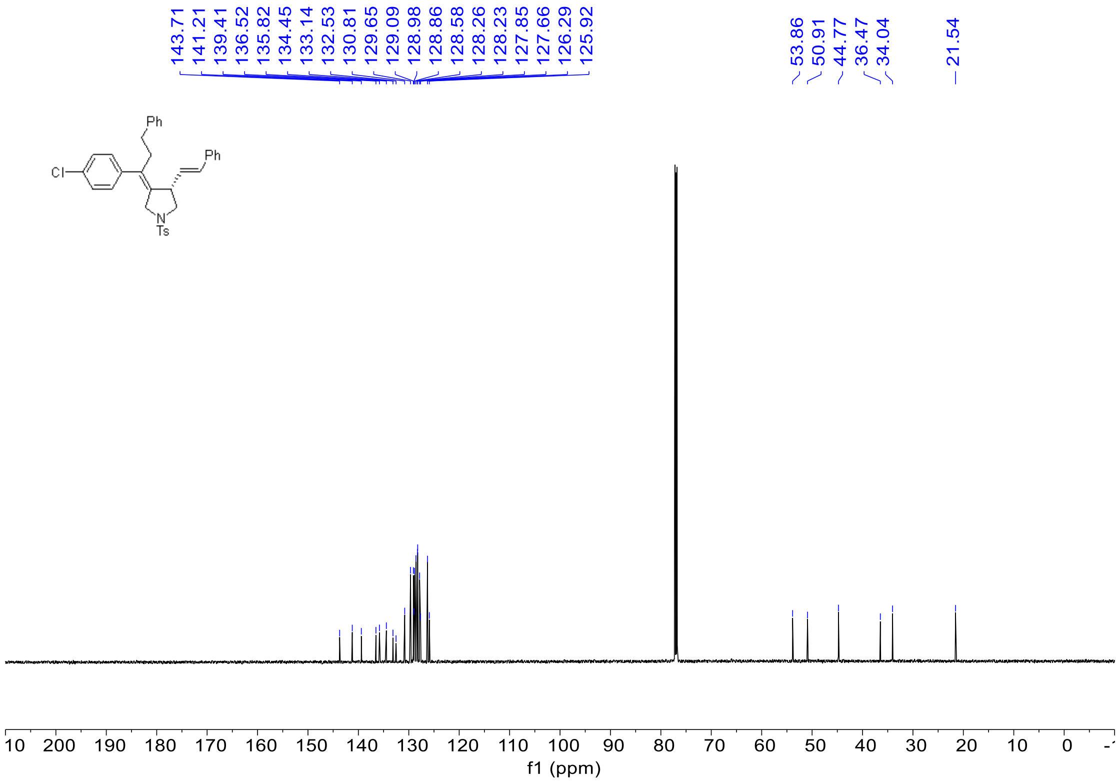

Supplementary Figure 293. ^{13}C NMR spectrum (151 MHz, $CDCl_3$) of **7da**

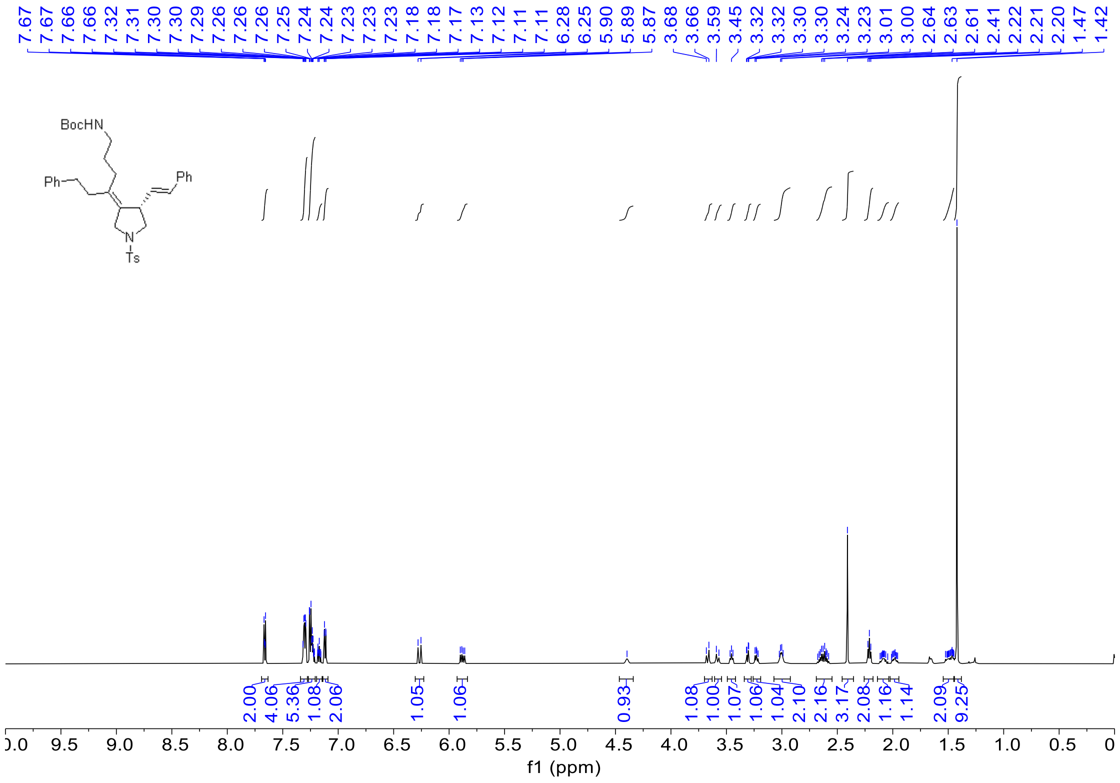

Supplementary Figure 294. ^{1}H NMR spectrum (600 MHz, $CDCl_3$) of **7el**

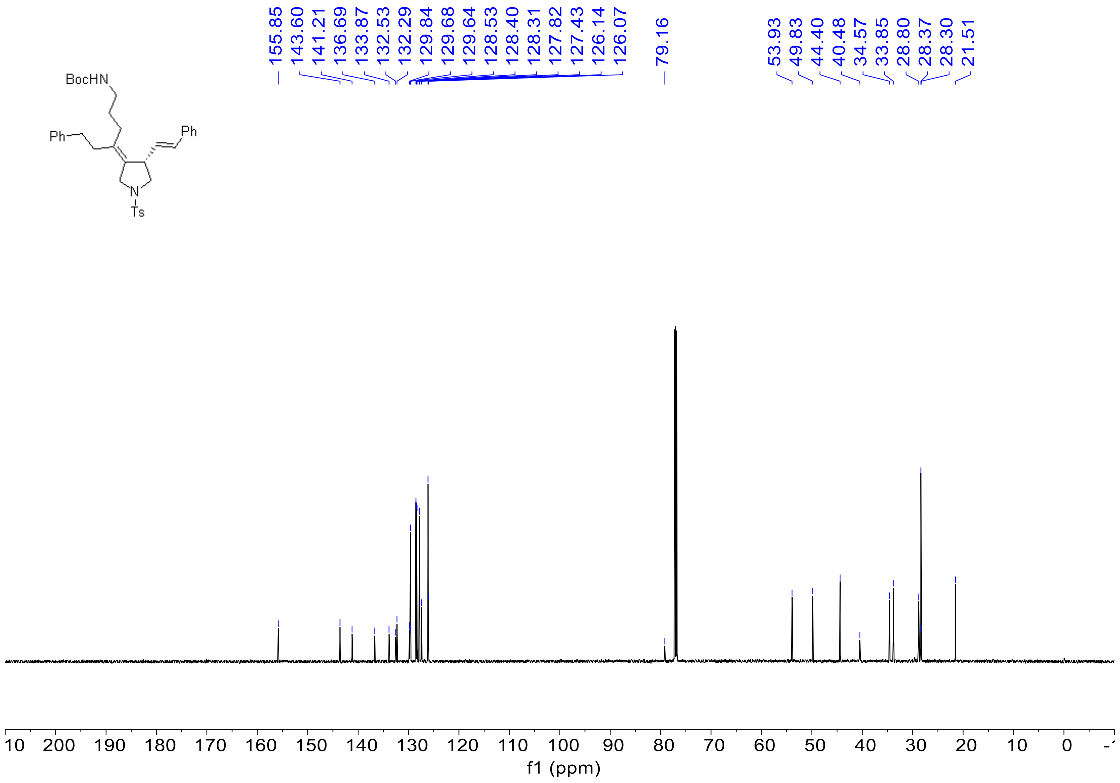

Supplementary Figure 295. ^{13}C NMR spectrum (151 MHz, $CDCl_3$) of **7el**

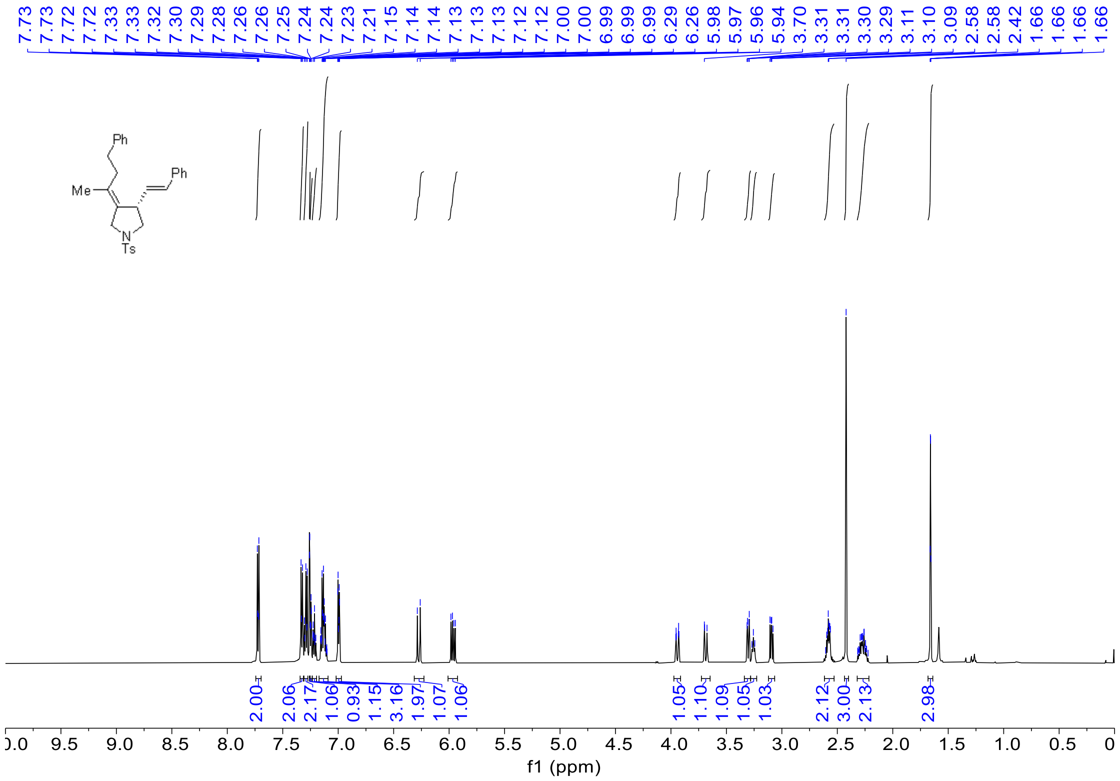

Supplementary Figure 296. ^{1}H NMR spectrum (600 MHz, $CDCl_3$) of **7fa**

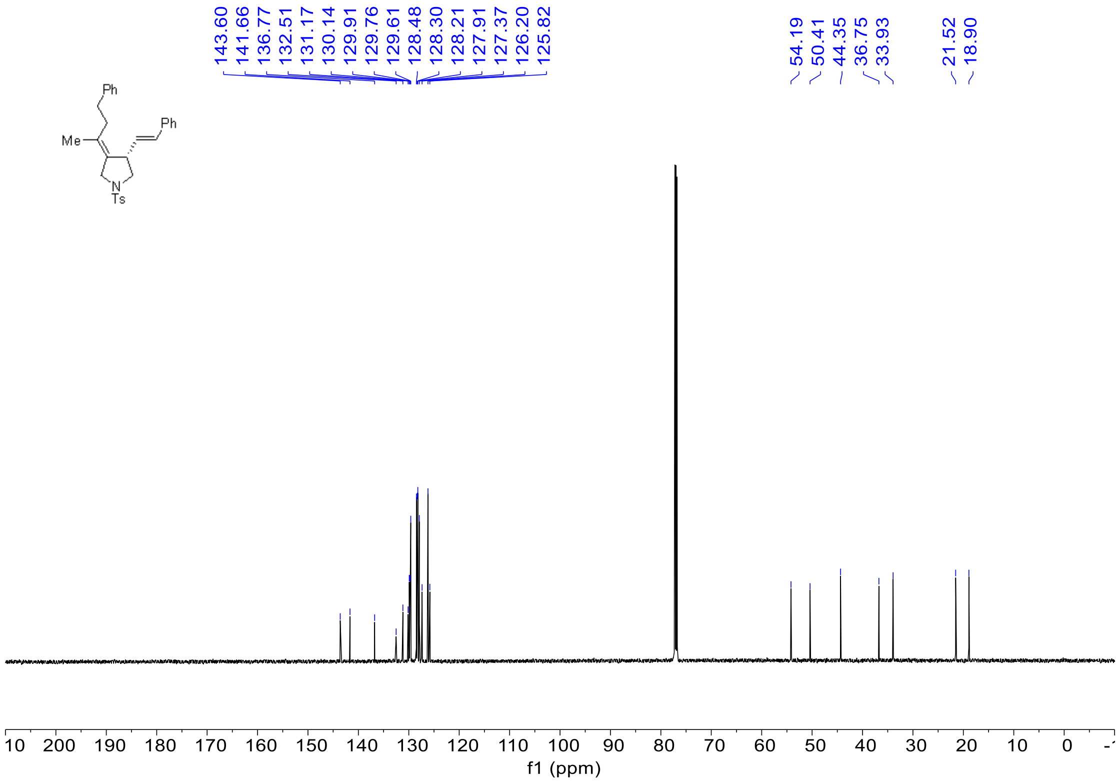

Supplementary Figure 297. ^{13}C NMR spectrum (151 MHz, $CDCl_3$) of **7fa**

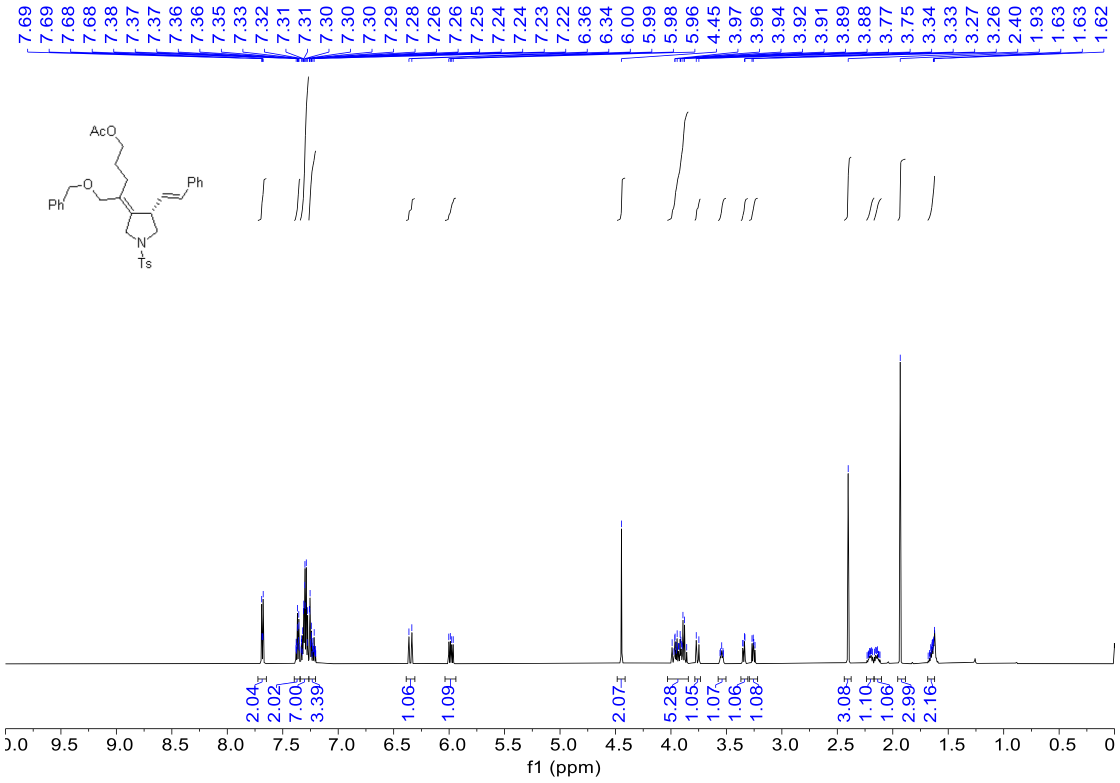

Supplementary Figure 298. ^{1}H NMR spectrum (600 MHz, $CDCl_3$) of **7gf**

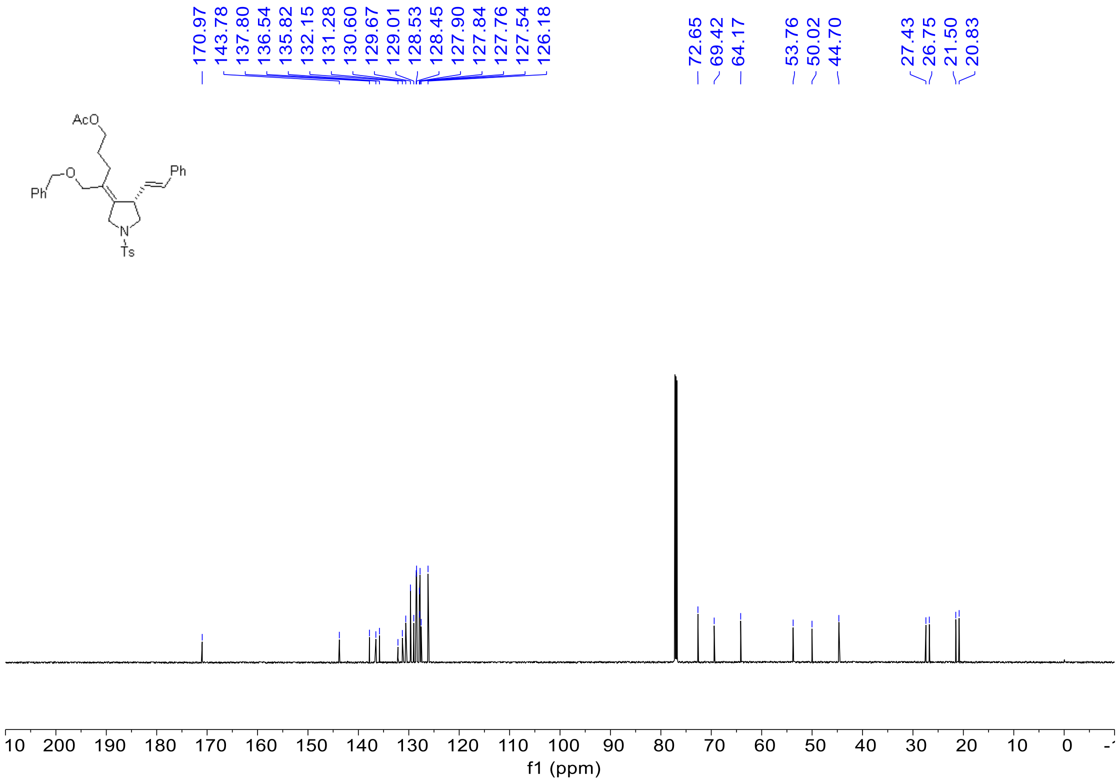

Supplementary Figure 299. ^{13}C NMR spectrum (151 MHz, $CDCl_3$) of **7gf**

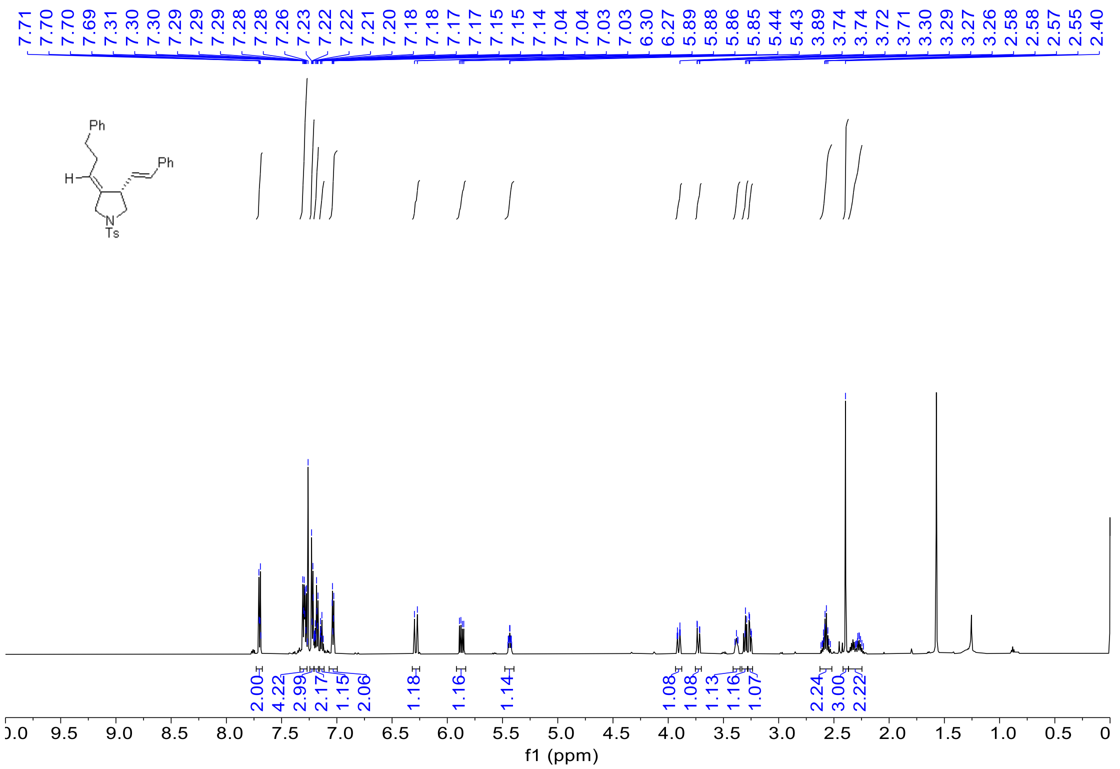

Supplementary Figure 300. ^{1}H NMR spectrum (600 MHz, $CDCl_3$) of **7ha**

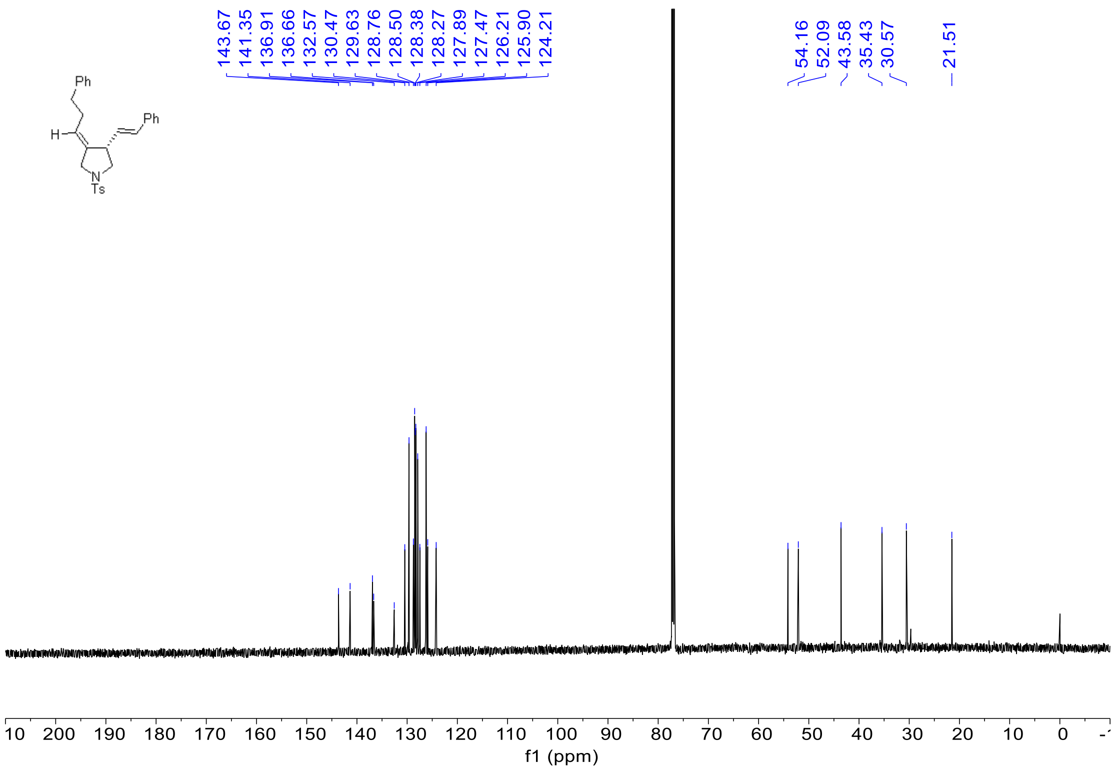

Supplementary Figure 301. ^{13}C NMR spectrum (151 MHz, $CDCl_3$) of **7ha**

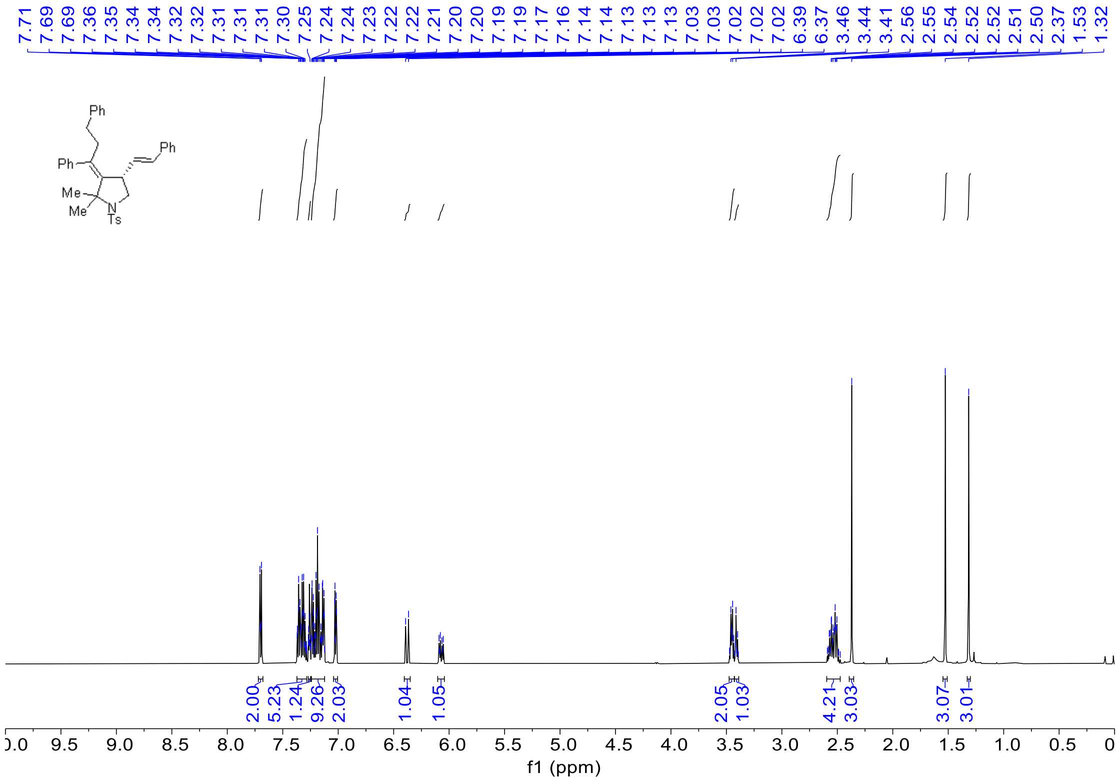

Supplementary Figure 302. ^{1}H NMR spectrum (600 MHz, $CDCl_3$) of **7ia**

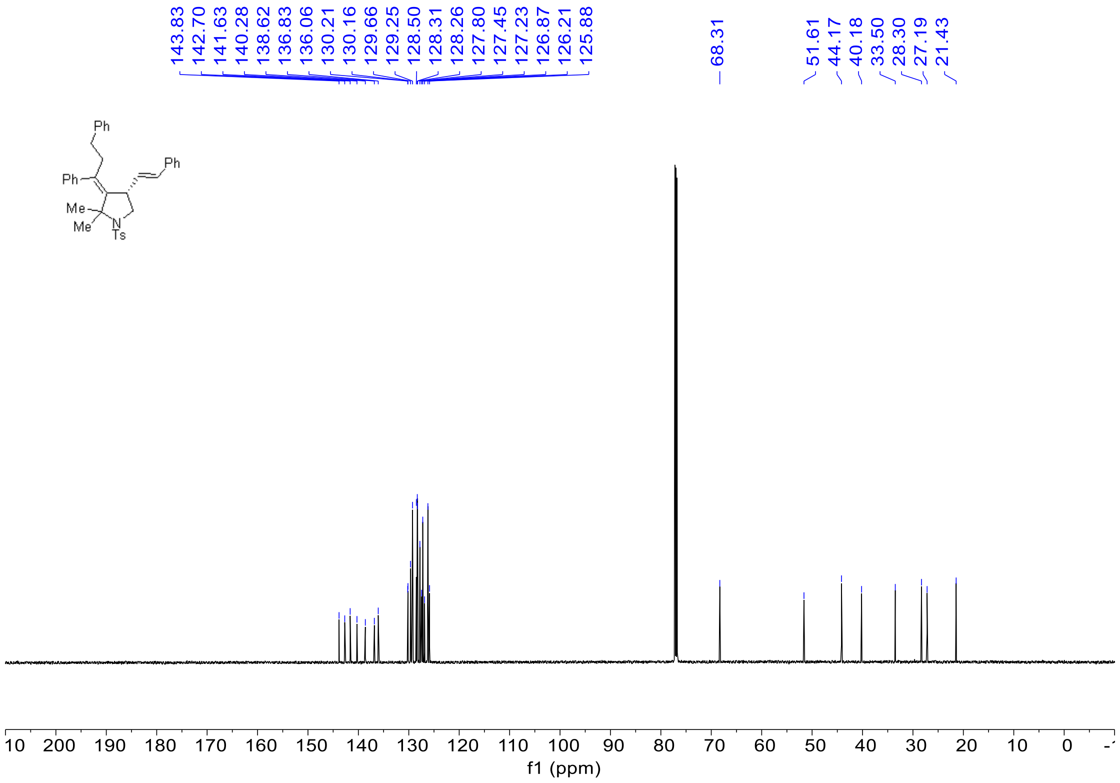

Supplementary Figure 303. ^{13}C NMR spectrum (151 MHz, $CDCl_3$) of **7ia**

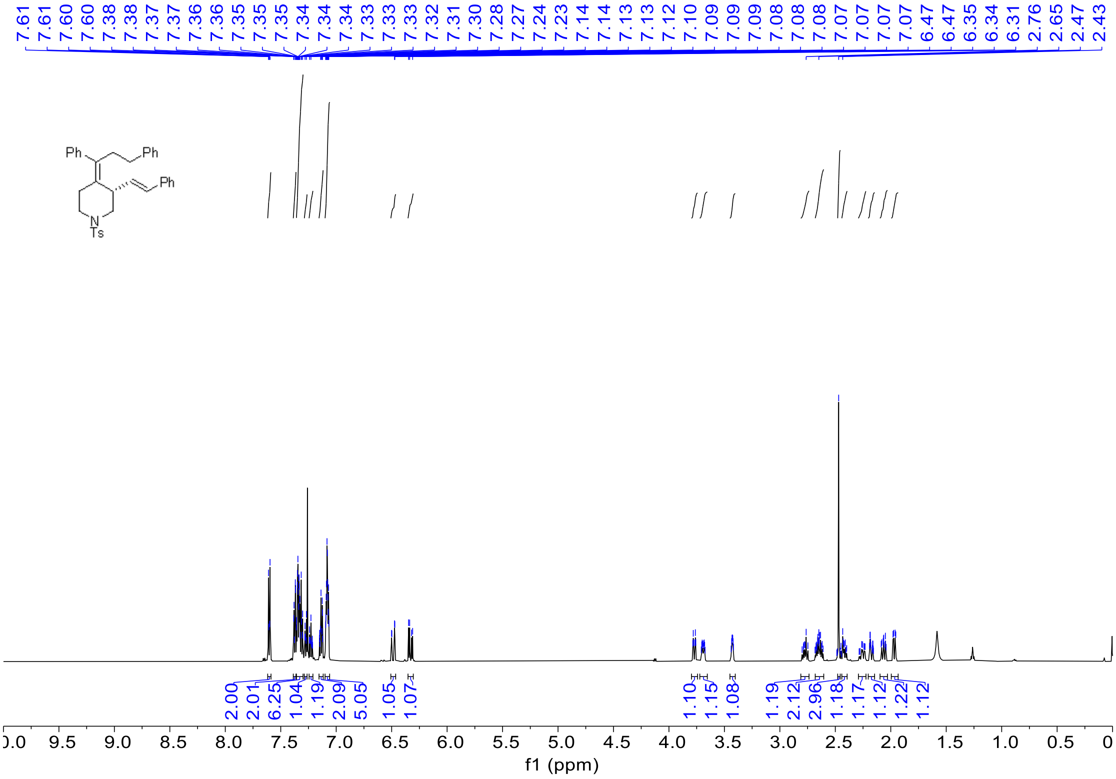

Supplementary Figure 304. ^{1}H NMR spectrum (600 MHz, $CDCl_3$) of **7ja**

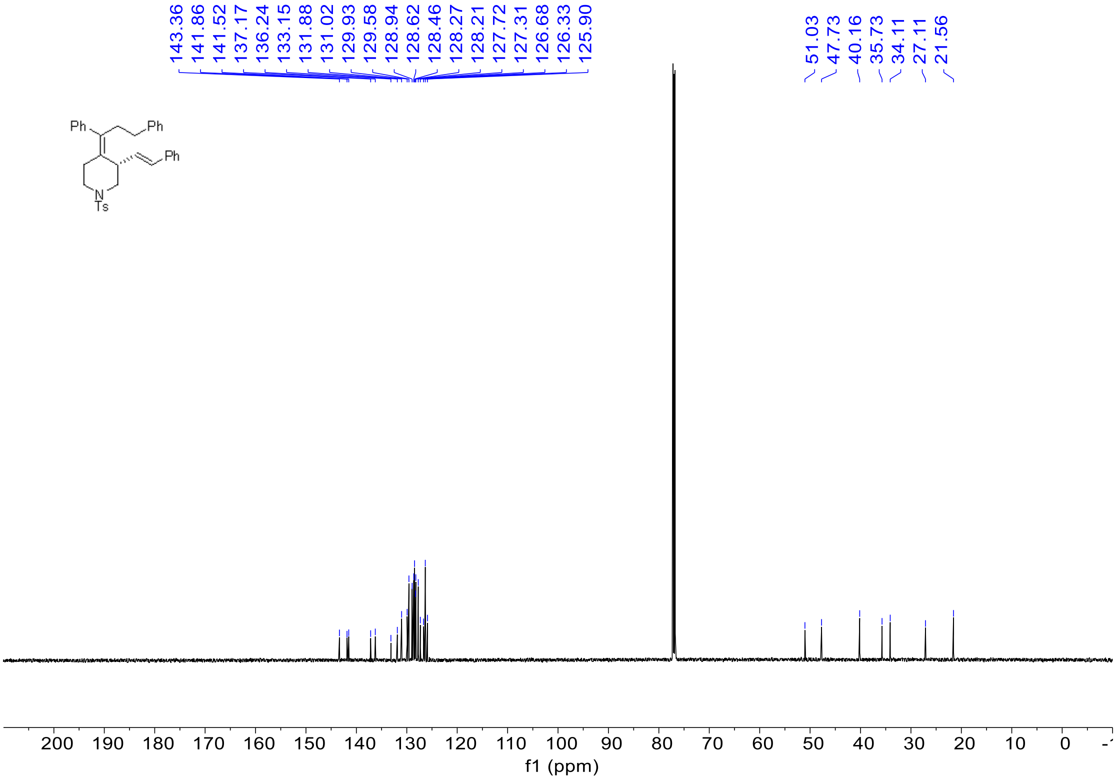

Supplementary Figure 305. ^{13}C NMR spectrum (151 MHz, $CDCl_3$) of **7ja**

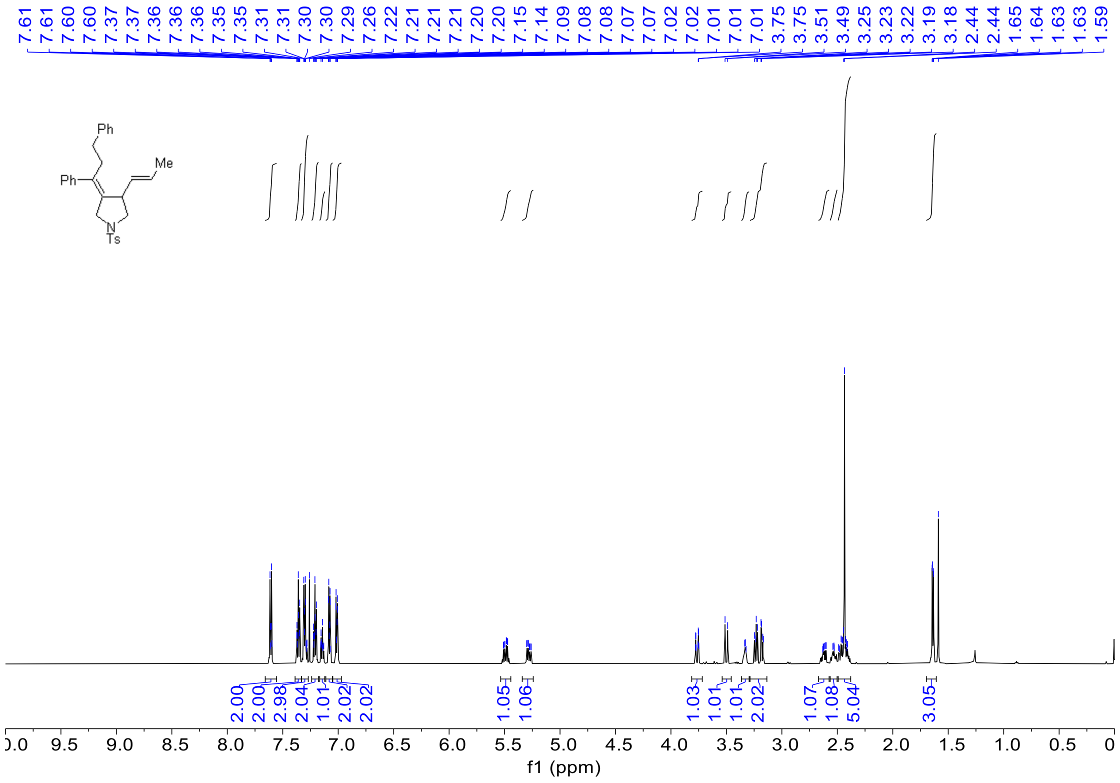

Supplementary Figure 306. ^{1}H NMR spectrum (600 MHz, $CDCl_3$) of **7ka**

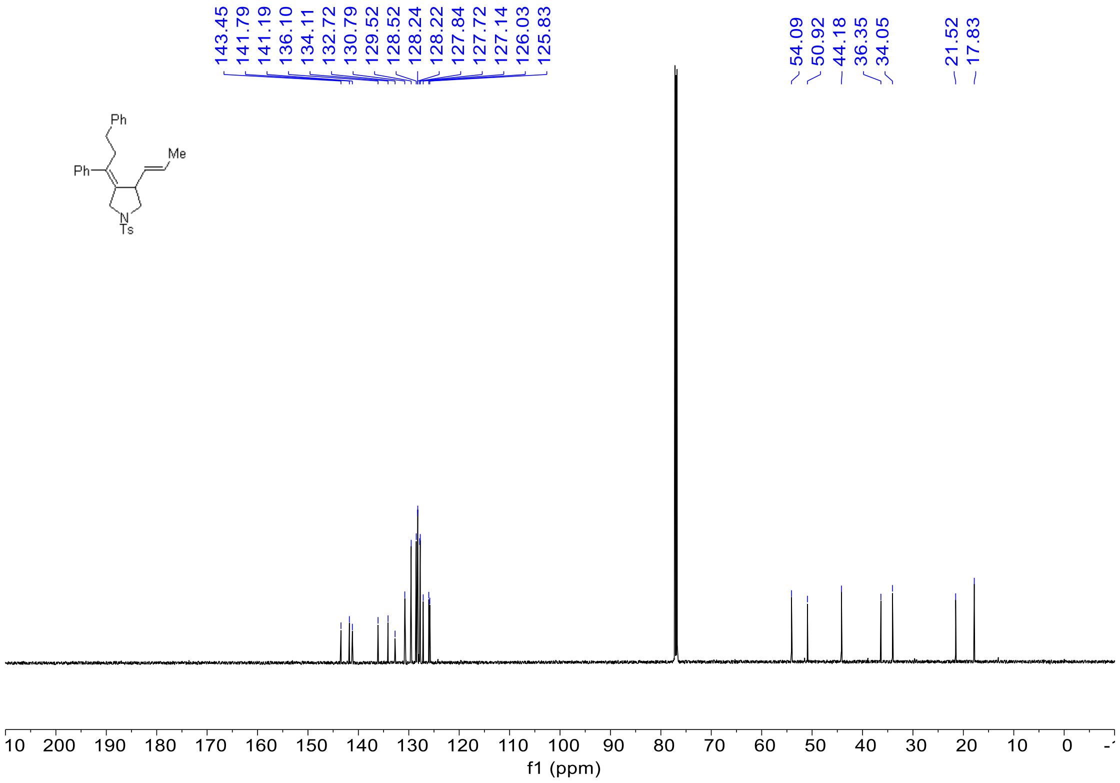

Supplementary Figure 307. ^{13}C NMR spectrum (151 MHz, $CDCl_3$) of **7ka**

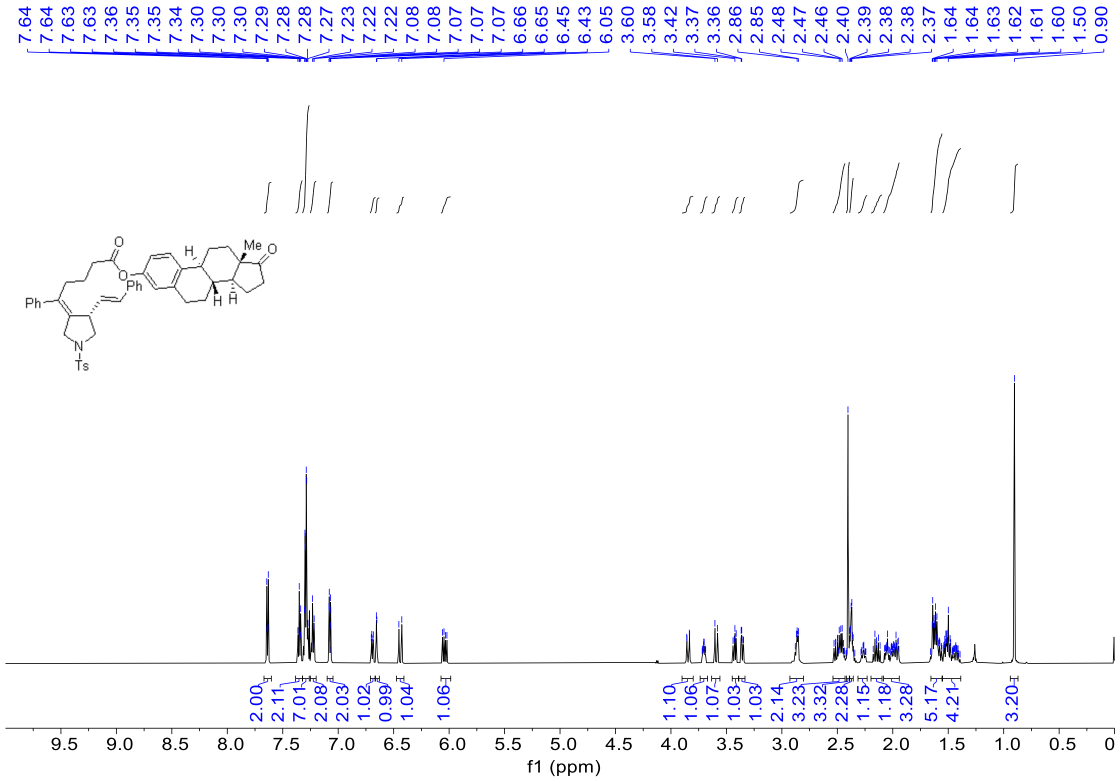

Supplementary Figure 308. ^{1}H NMR spectrum (600 MHz, $CDCl_3$) of **7ap**

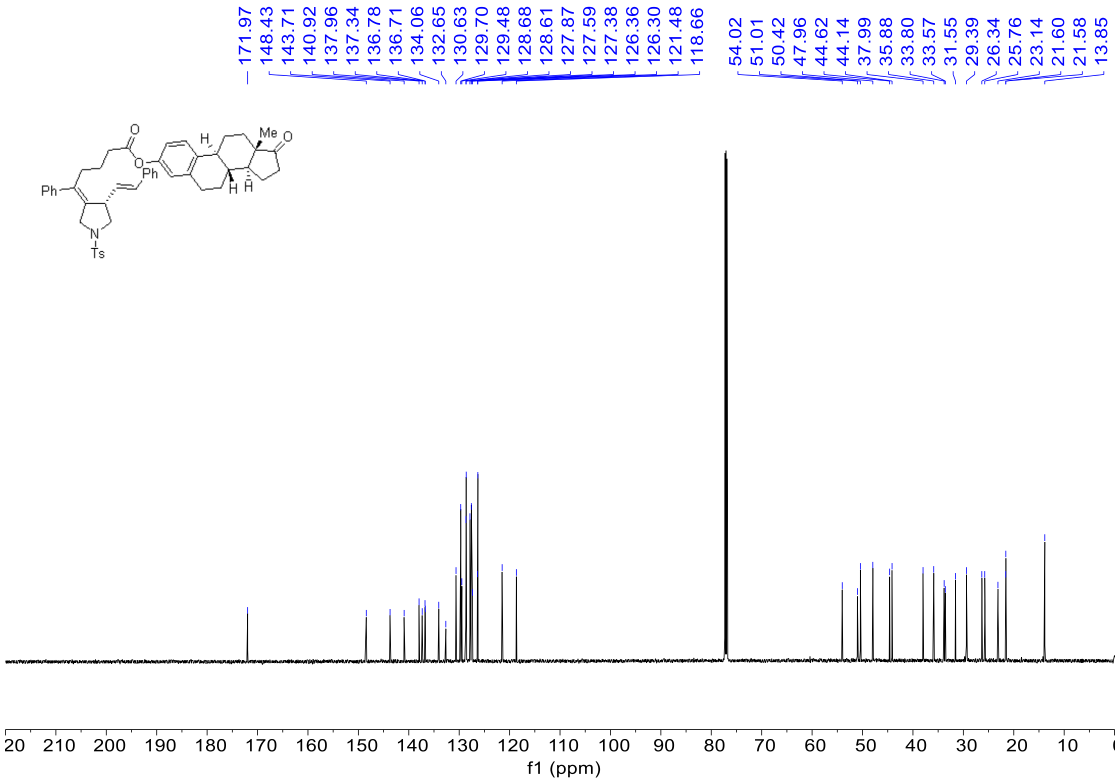

Supplementary Figure 309. ^{13}C NMR spectrum (151 MHz, $CDCl_3$) of **7ap**

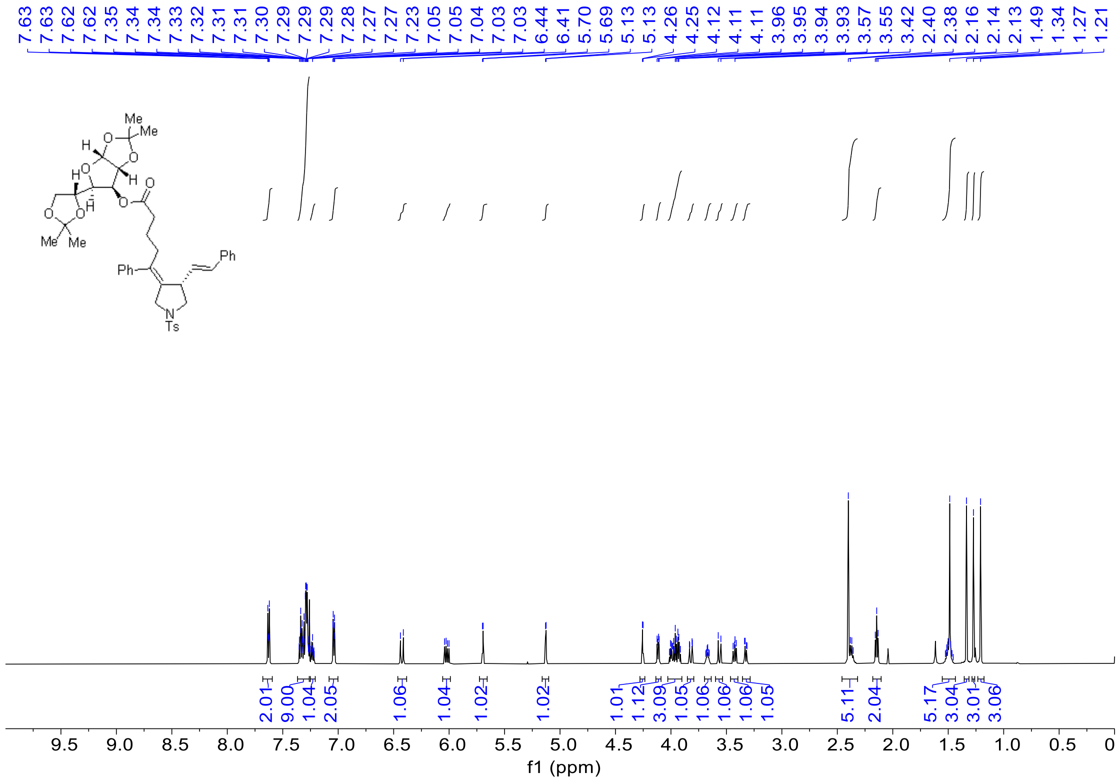

Supplementary Figure 310. ^{1}H NMR spectrum (600 MHz, $CDCl_3$) of **7aq**

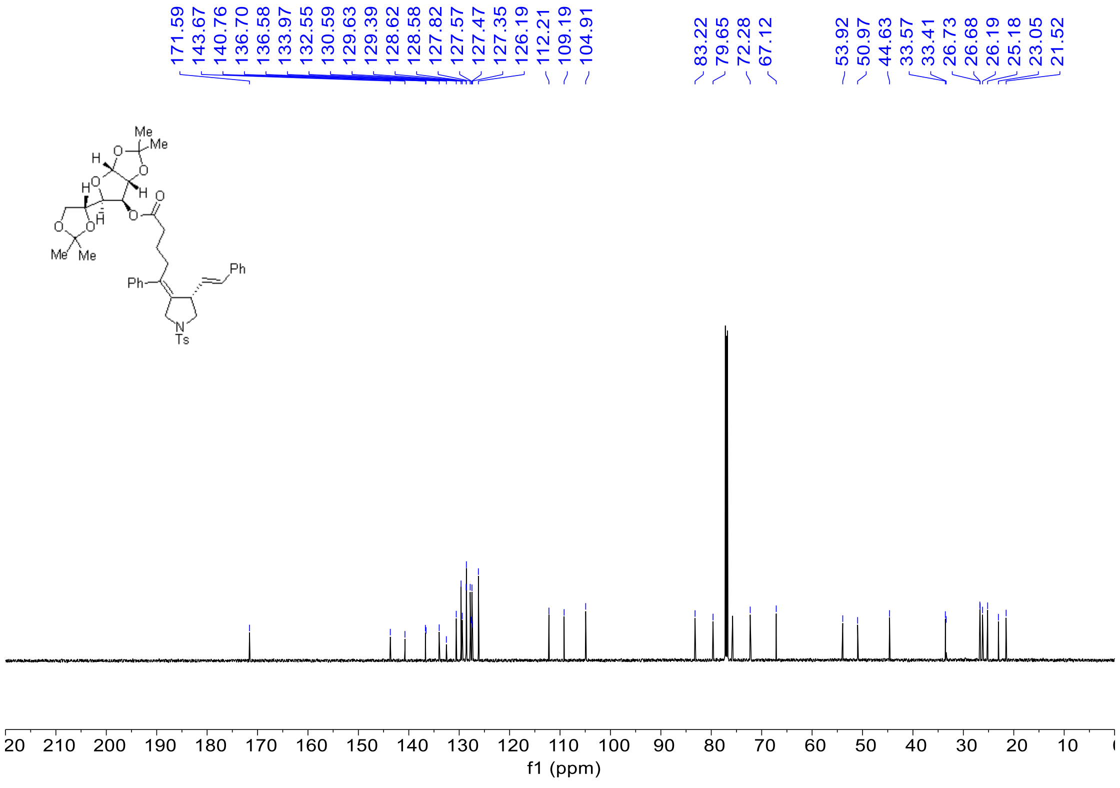

Supplementary Figure 311. ^{13}C NMR spectrum (151 MHz, $CDCl_3$) of **7aq**

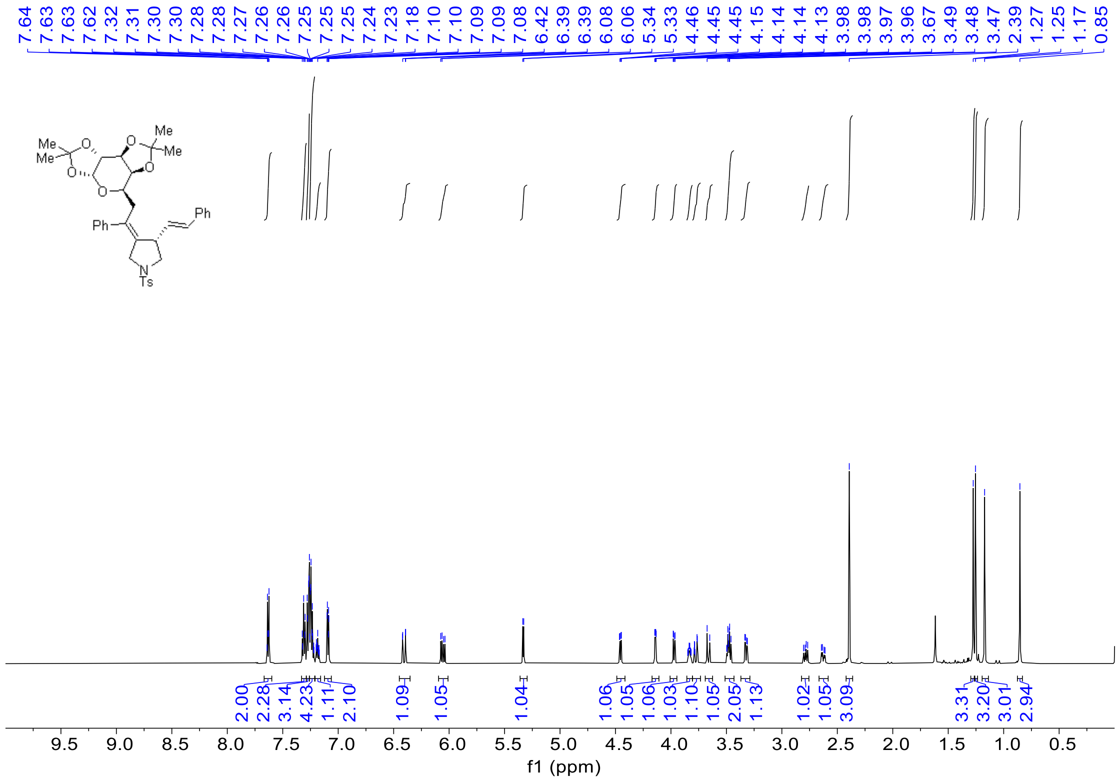

Supplementary Figure 312. ^{1}H NMR spectrum (600 MHz, $CDCl_3$) of **7ar**

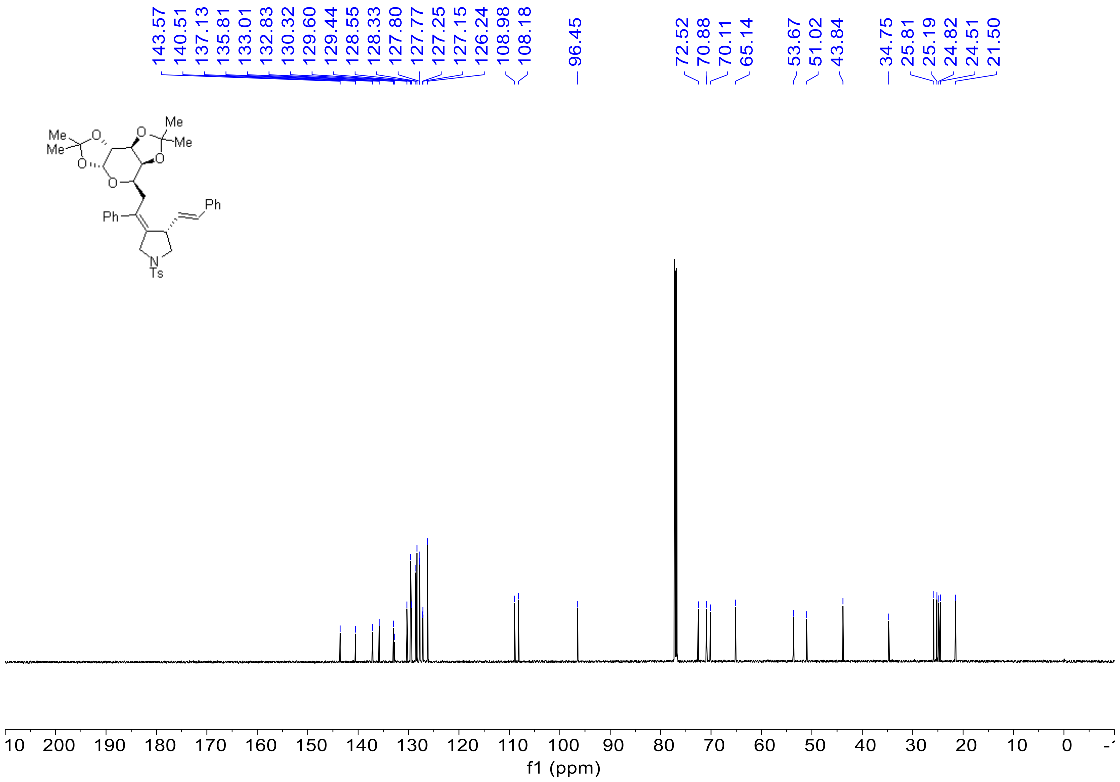

Supplementary Figure 313. ^{13}C NMR spectrum (151 MHz, $CDCl_3$) of **7ar**

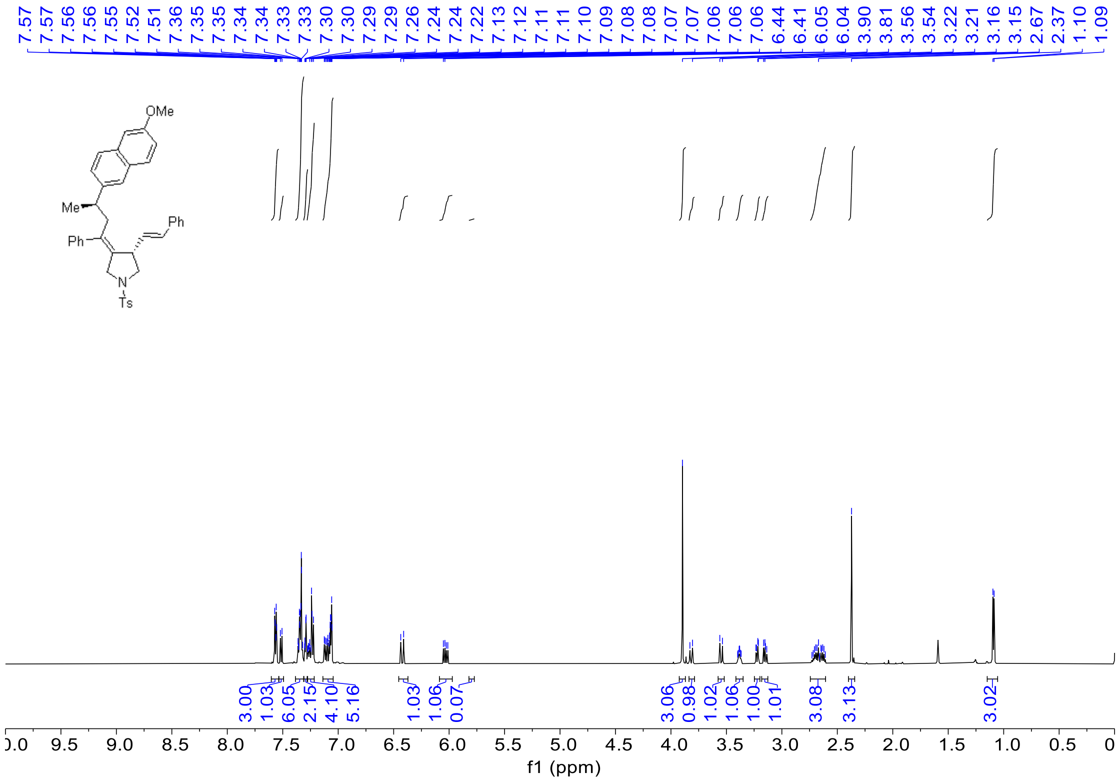

Supplementary Figure 314. ^{1}H NMR spectrum (600 MHz, $CDCl_3$) of **7as**

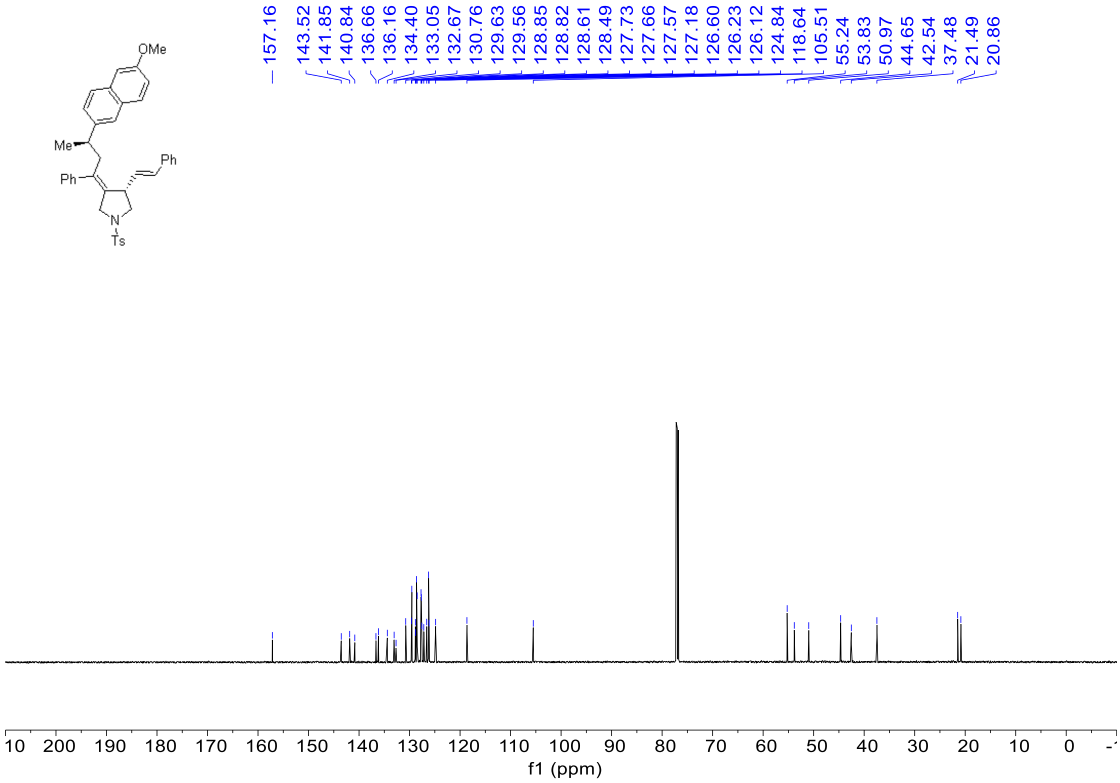

Supplementary Figure 315. ^{13}C NMR spectrum (151 MHz, $CDCl_3$) of **7as**

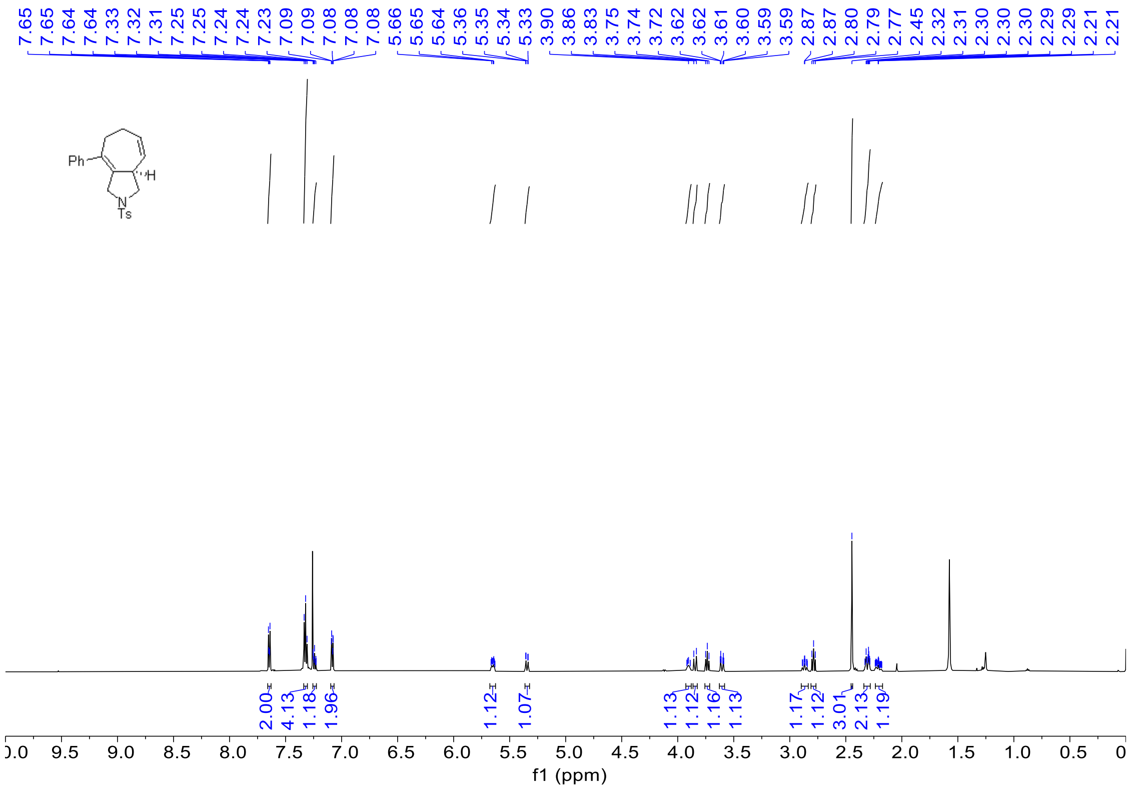

Supplementary Figure 316. ^{1}H NMR spectrum (600 MHz, $CDCl_3$) of **8**

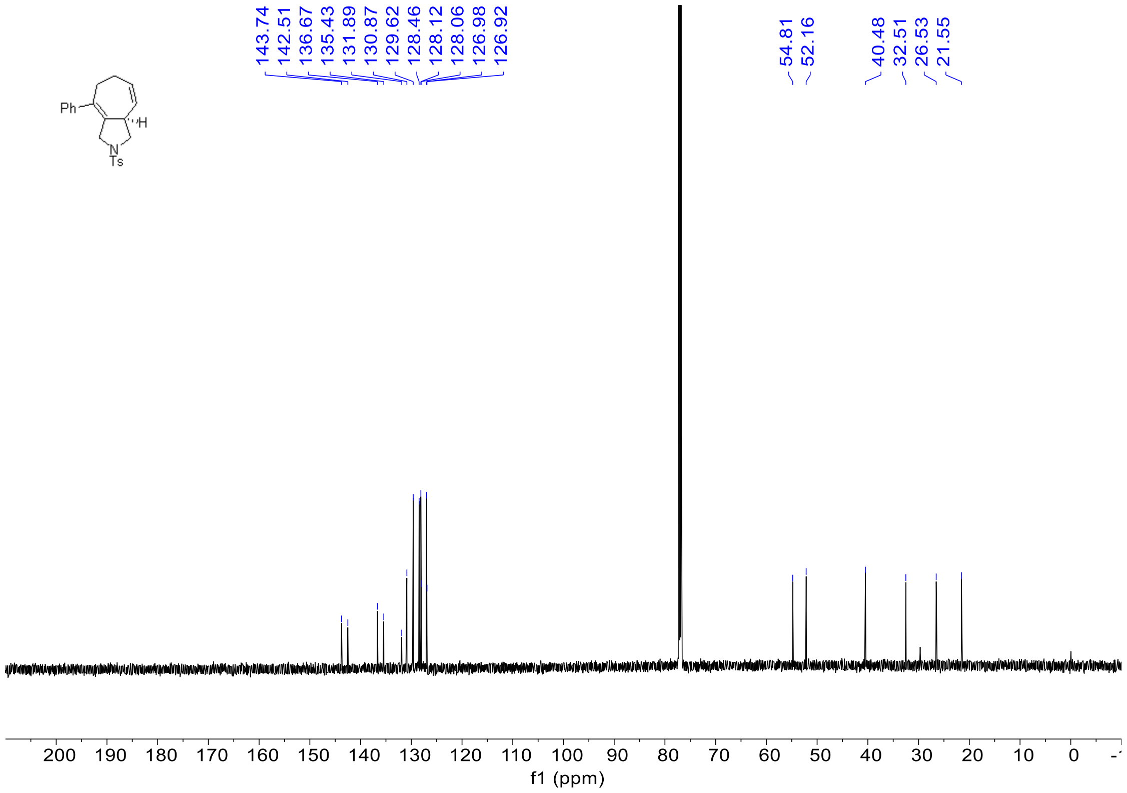

Supplementary Figure 317. ^{13}C NMR spectrum (151 MHz, $CDCl_3$) of **8**

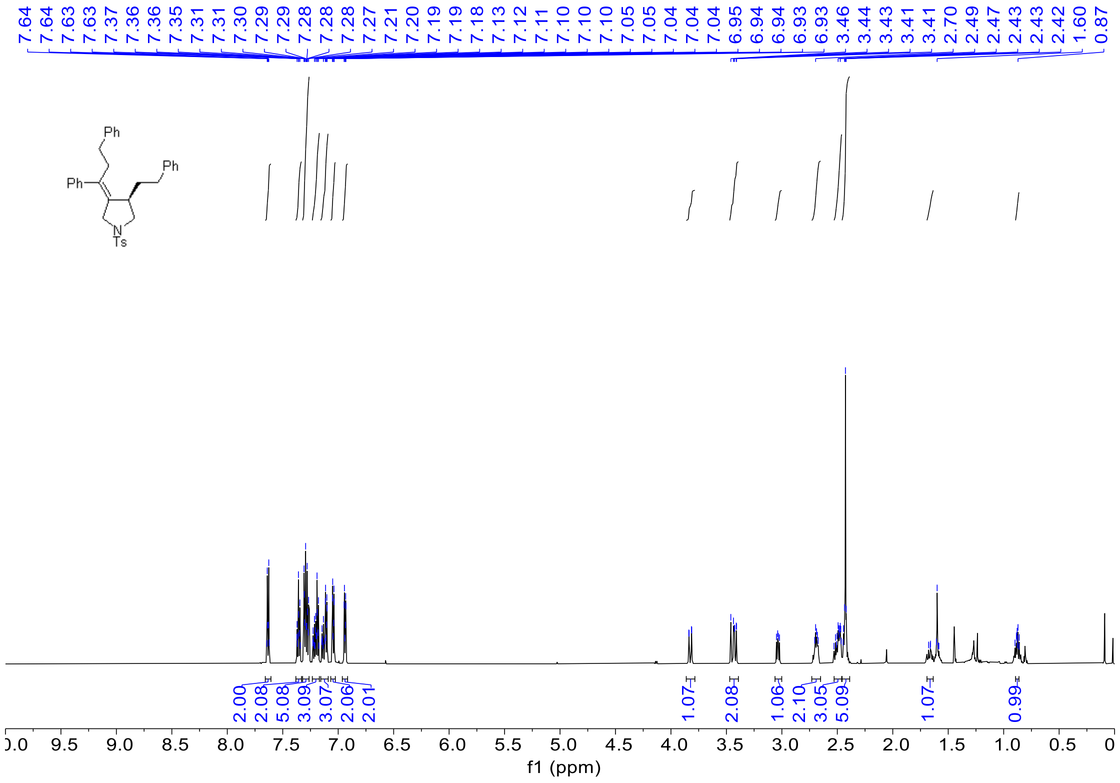

Supplementary Figure 318. ^{1}H NMR spectrum (600 MHz, $CDCl_3$) of **9aa**

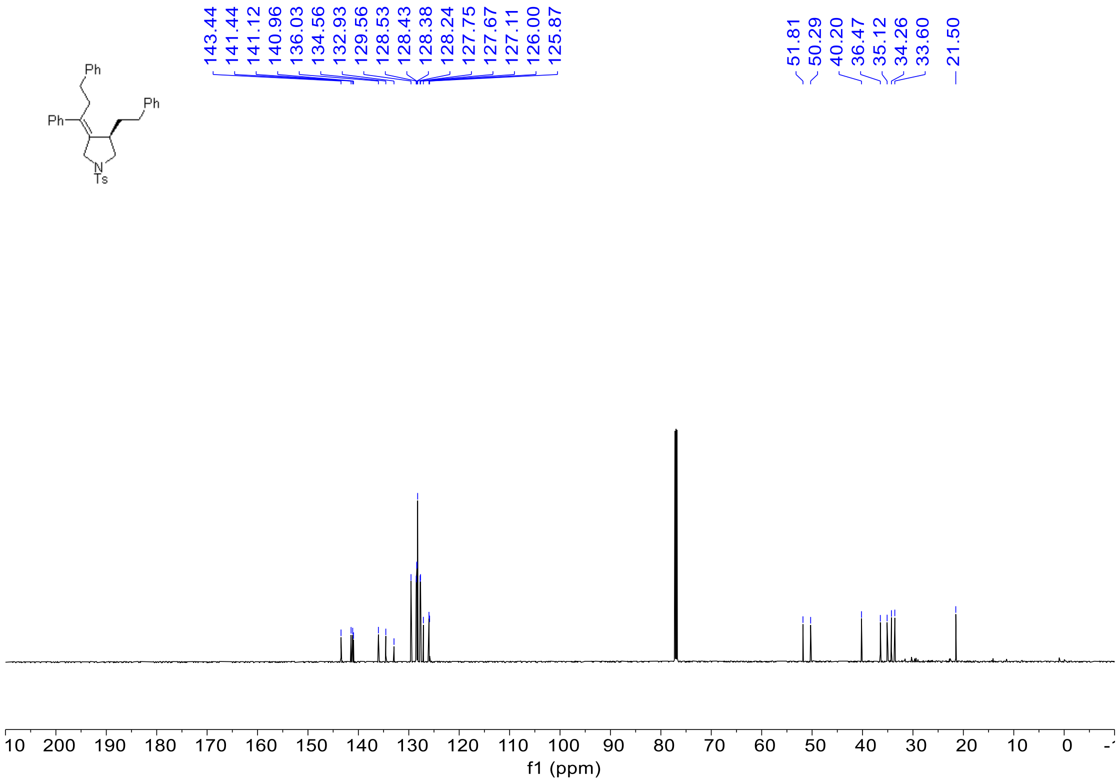

Supplementary Figure 319. ^{13}C NMR spectrum (151 MHz, $CDCl_3$) of **9aa**

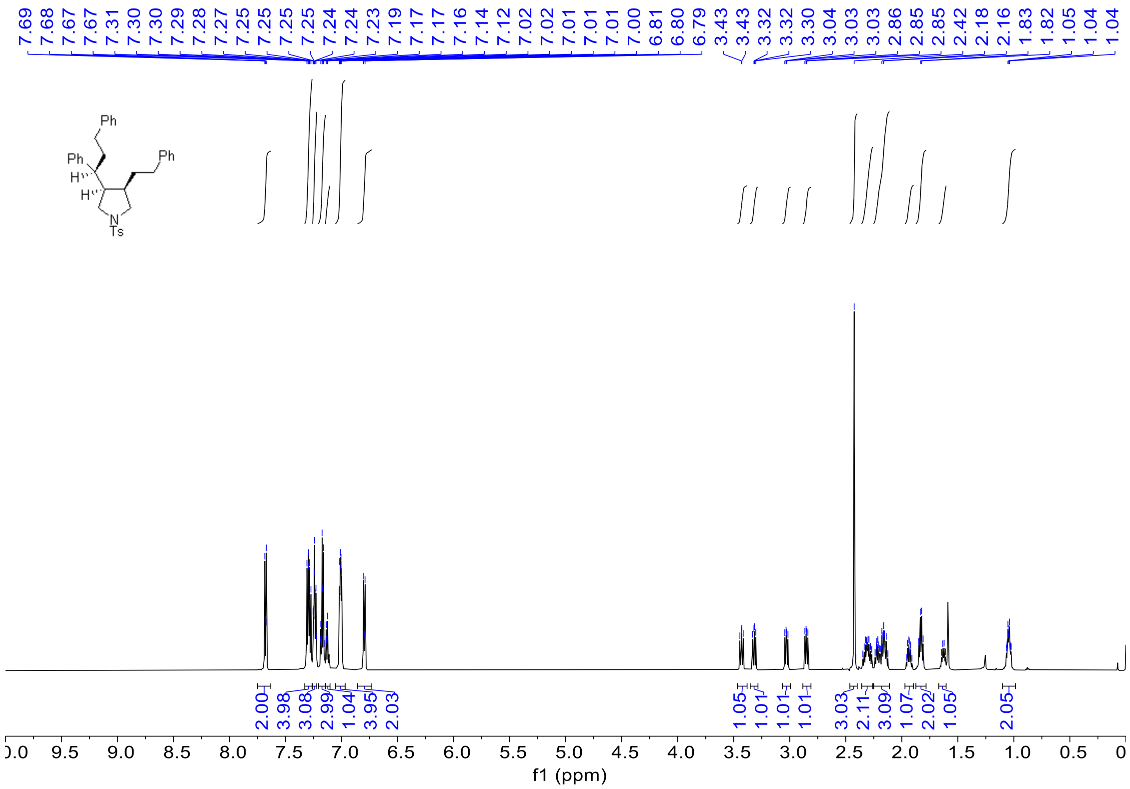

Supplementary Figure 320. ^{1}H NMR spectrum (600 MHz, $CDCl_3$) of **10aa**

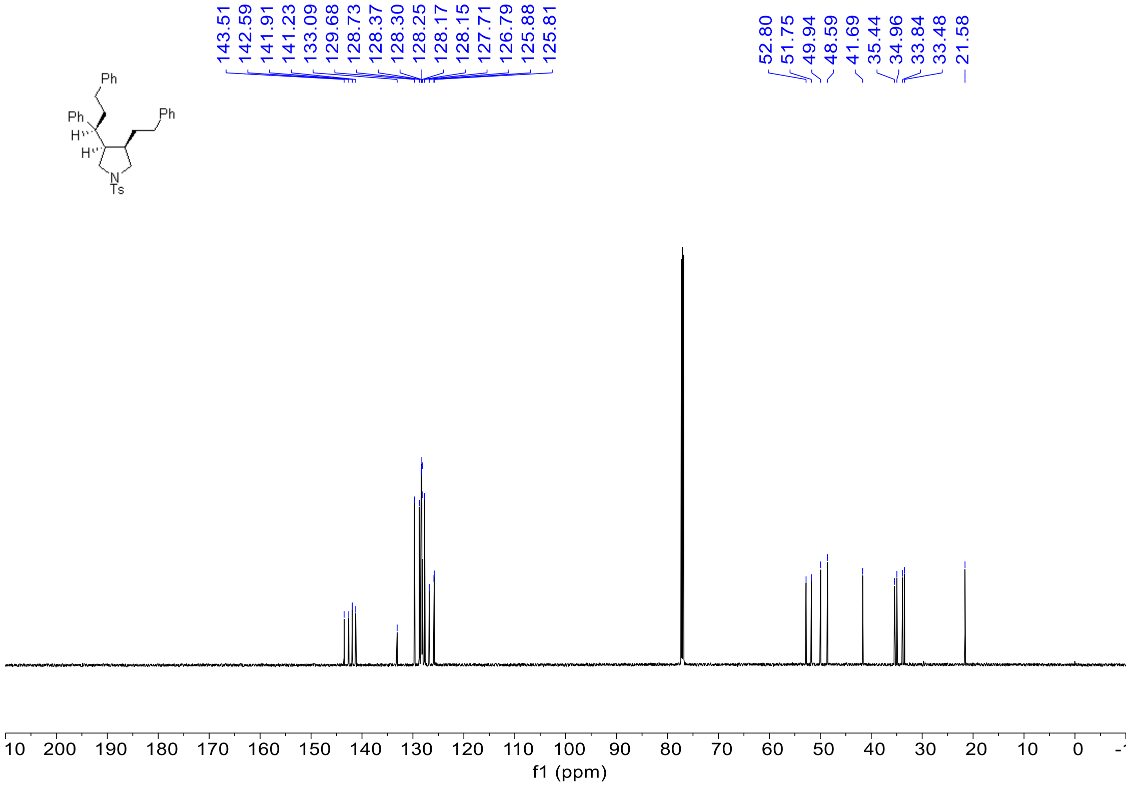

Supplementary Figure 321. ^{13}C NMR spectrum (151 MHz, $CDCl_3$) of **10aa**

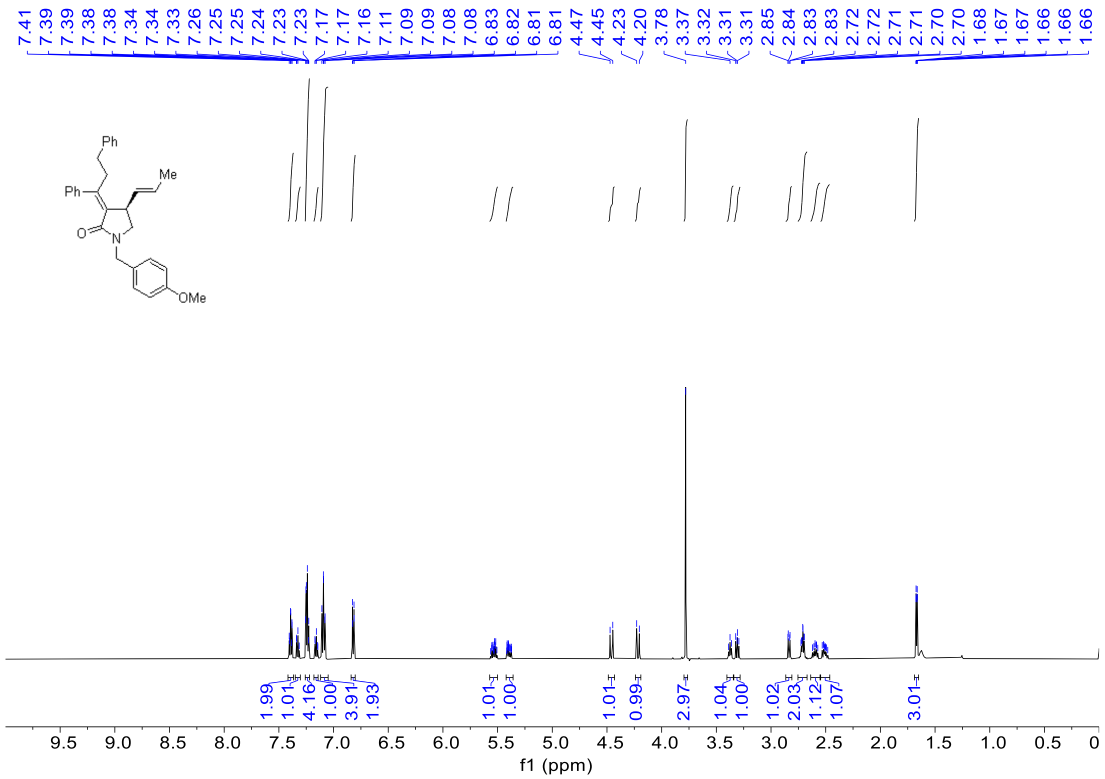

Supplementary Figure 322. ^{1}H NMR spectrum (600 MHz, $CDCl_3$) of **7la**

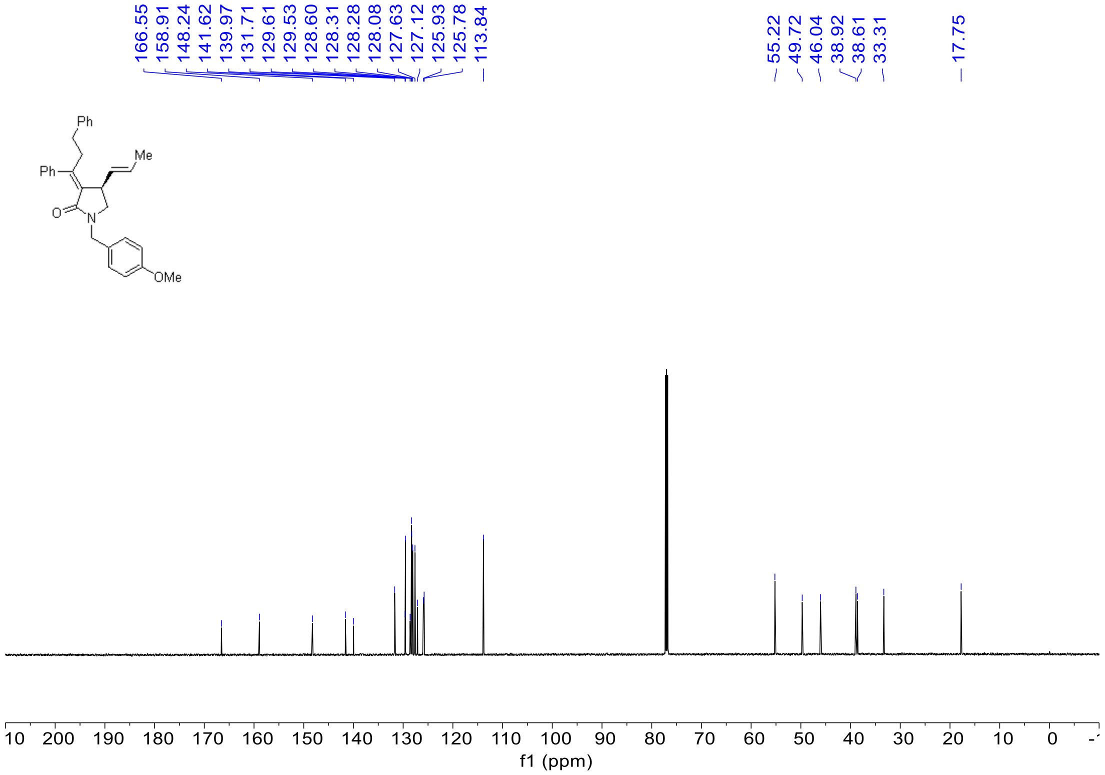

Supplementary Figure 323. ^{13}C NMR spectrum (151 MHz, $CDCl_3$) of **7la**

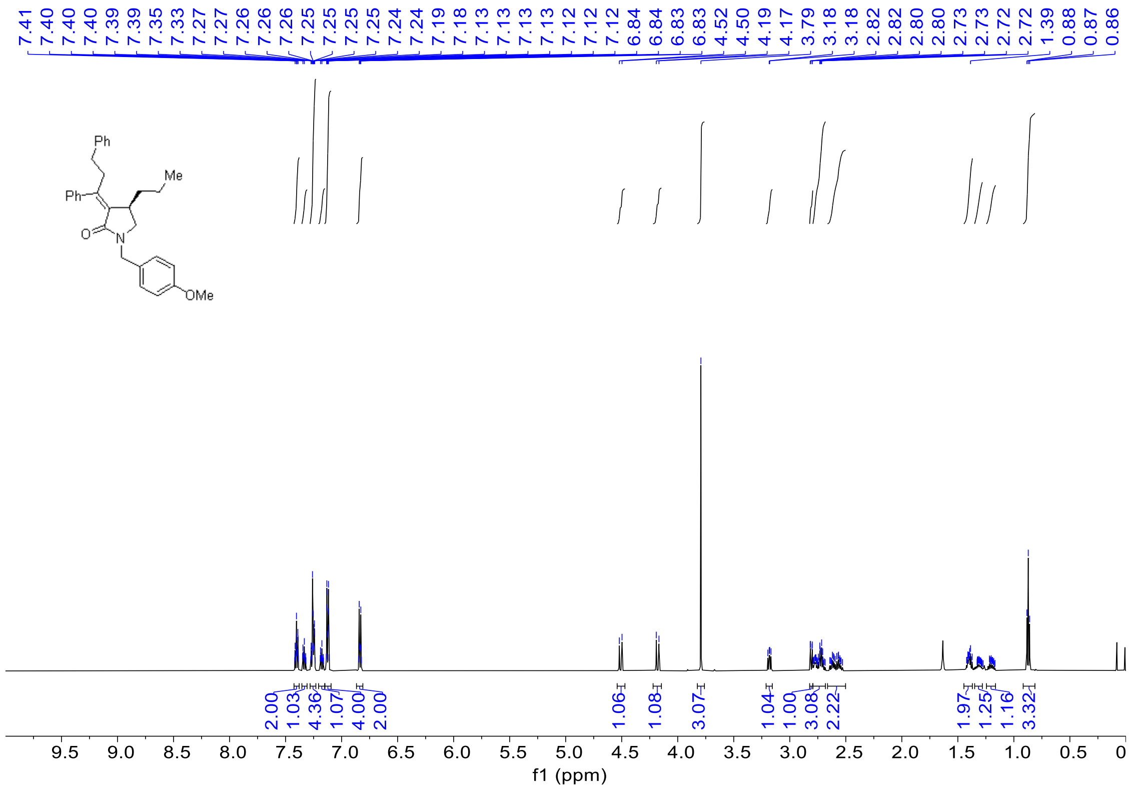

Supplementary Figure 324. ^{1}H NMR spectrum (600 MHz, $CDCl_3$) of **9la**

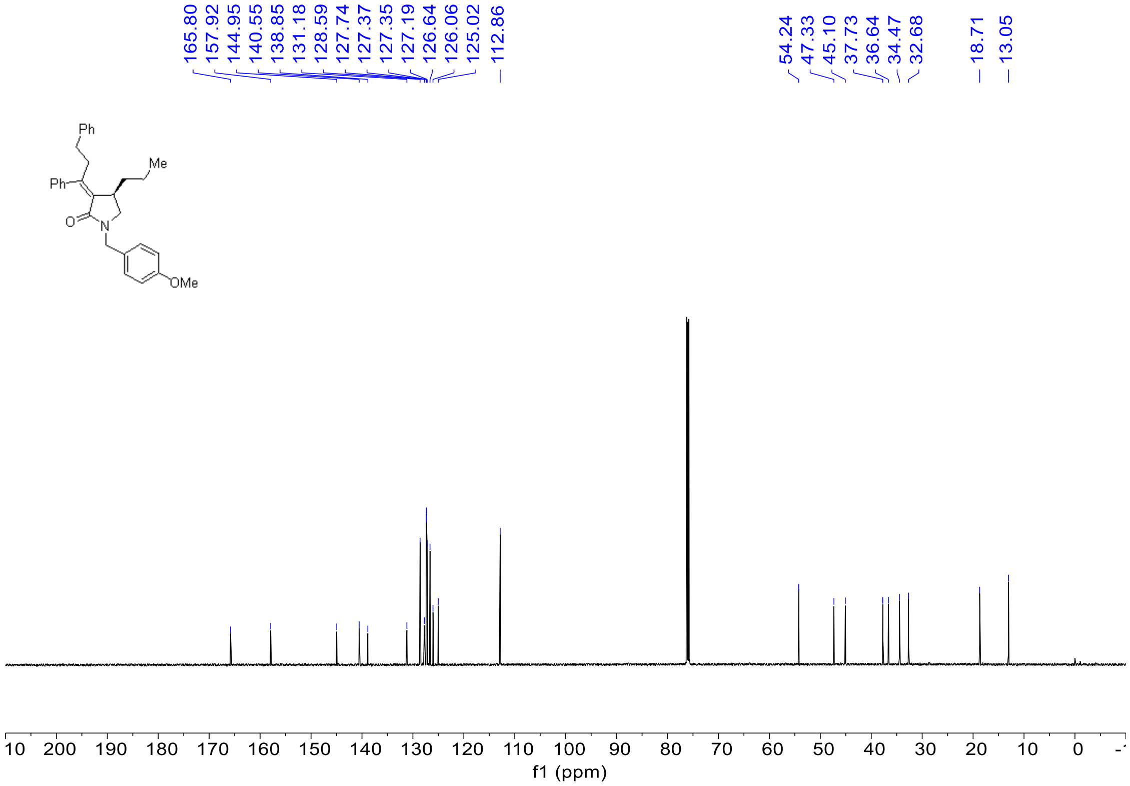

Supplementary Figure 325. ^{13}C NMR spectrum (151 MHz, $CDCl_3$) of **9la**

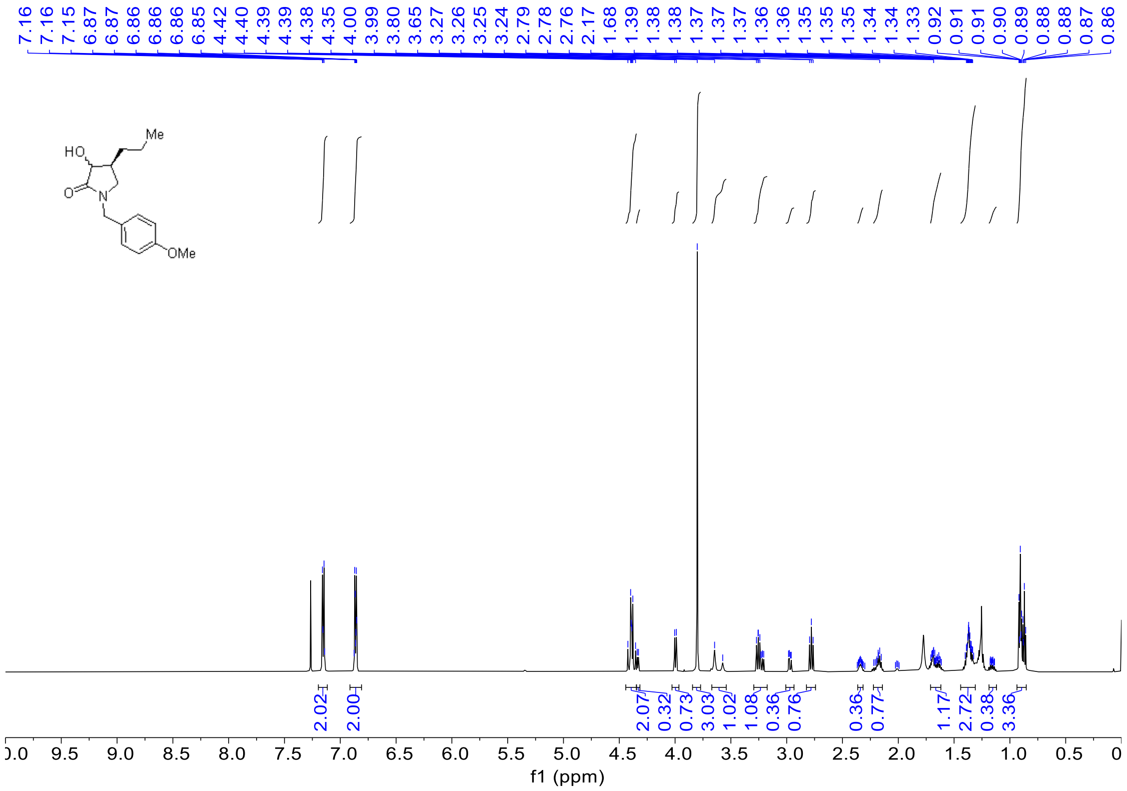

Supplementary Figure 326. ^{1}H NMR spectrum (600 MHz, $CDCl_3$) of **11**

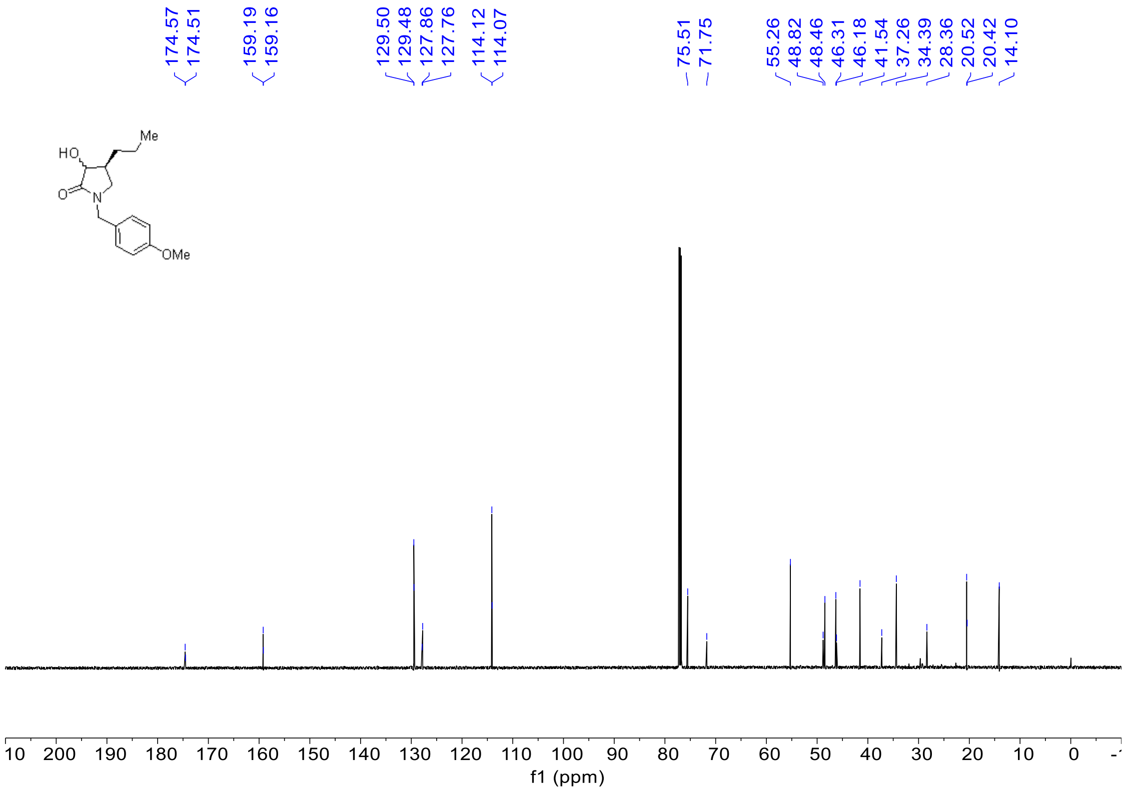

Supplementary Figure 327. ^{13}C NMR spectrum (151 MHz, $CDCl_3$) of **11**

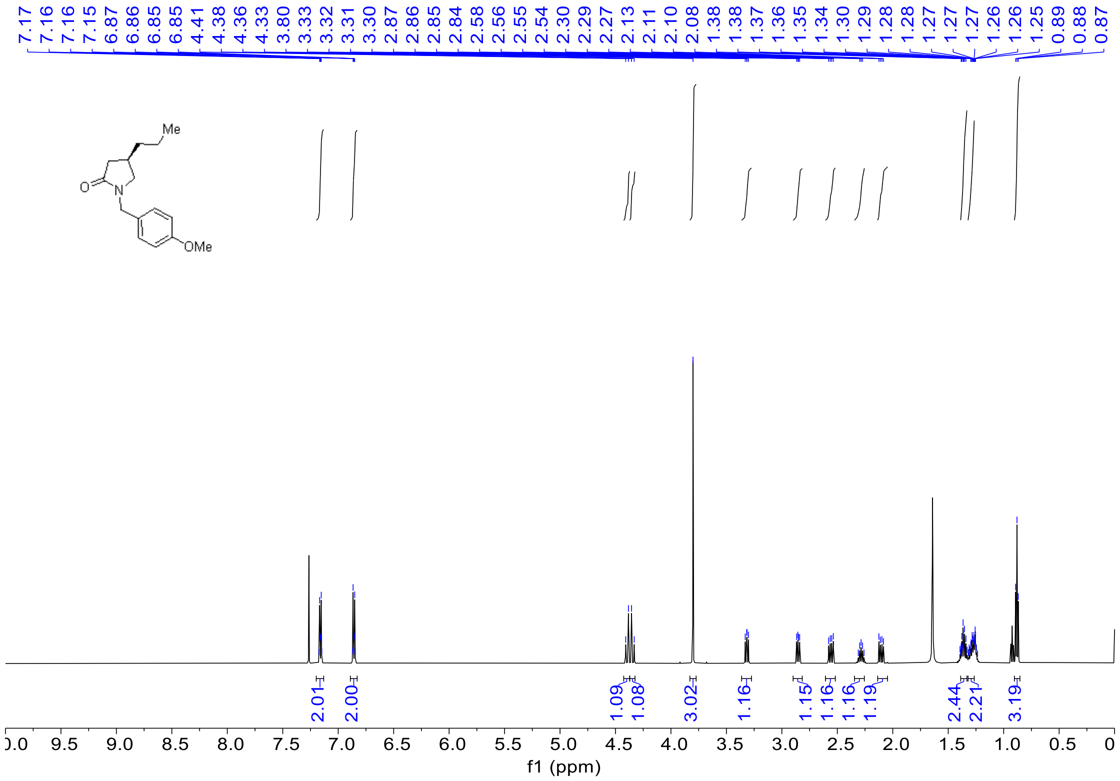

Supplementary Figure 328. ^{1}H NMR spectrum (600 MHz, $CDCl_3$) of **12**

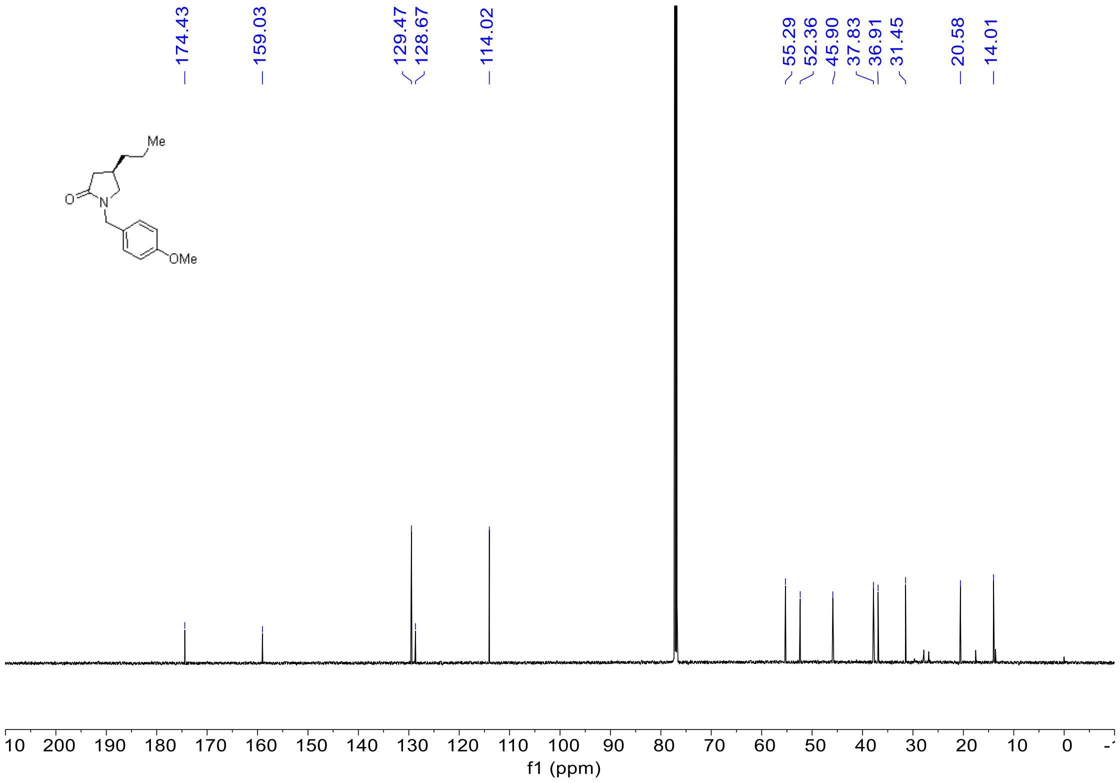

Supplementary Figure 329. ^{13}C NMR spectrum (151 MHz, $CDCl_3$) of **12**

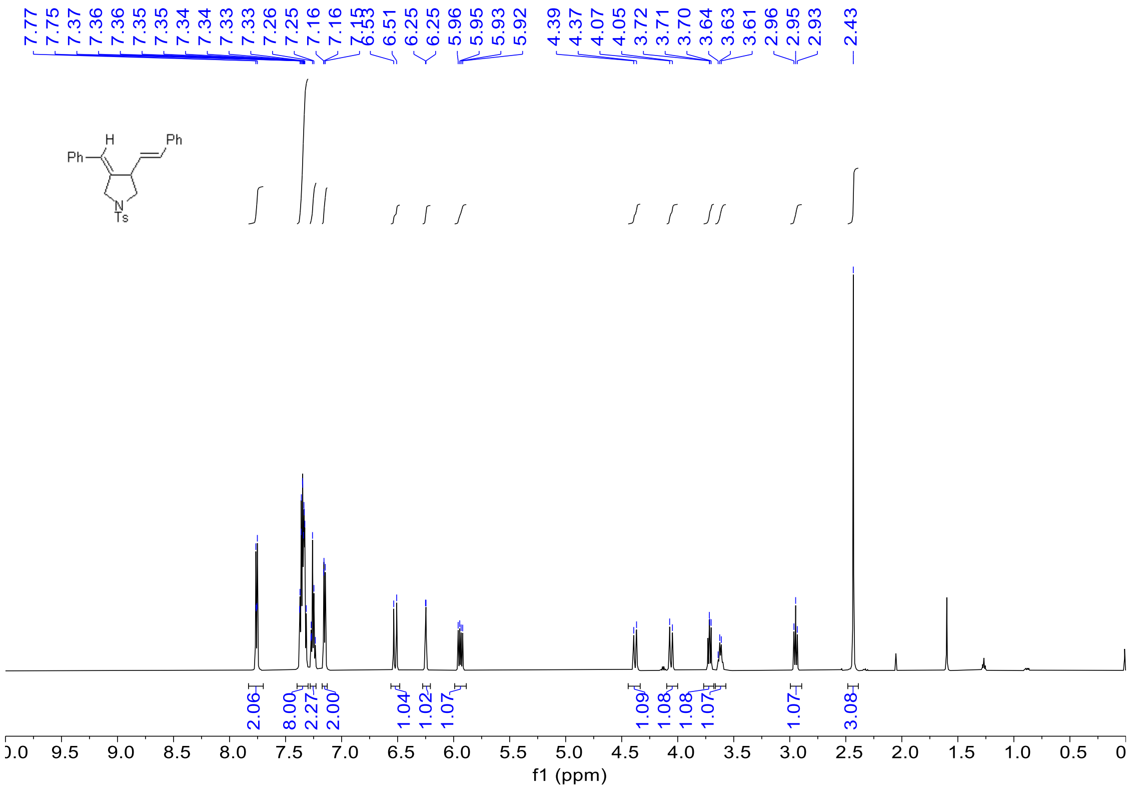

Supplementary Figure 330. ^{1}H NMR spectrum (600 MHz, $CDCl_3$) of **16**

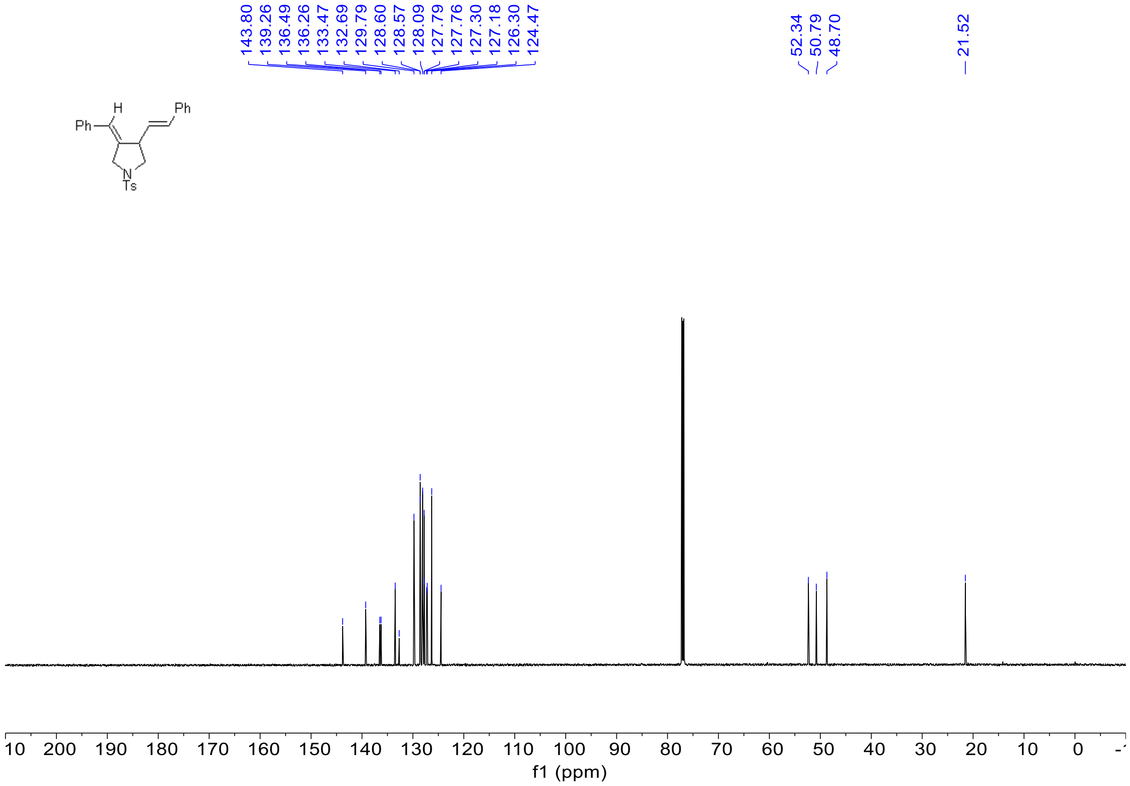

Supplementary Figure 331. ^{13}C NMR spectrum (151 MHz, $CDCl_3$) of **16**

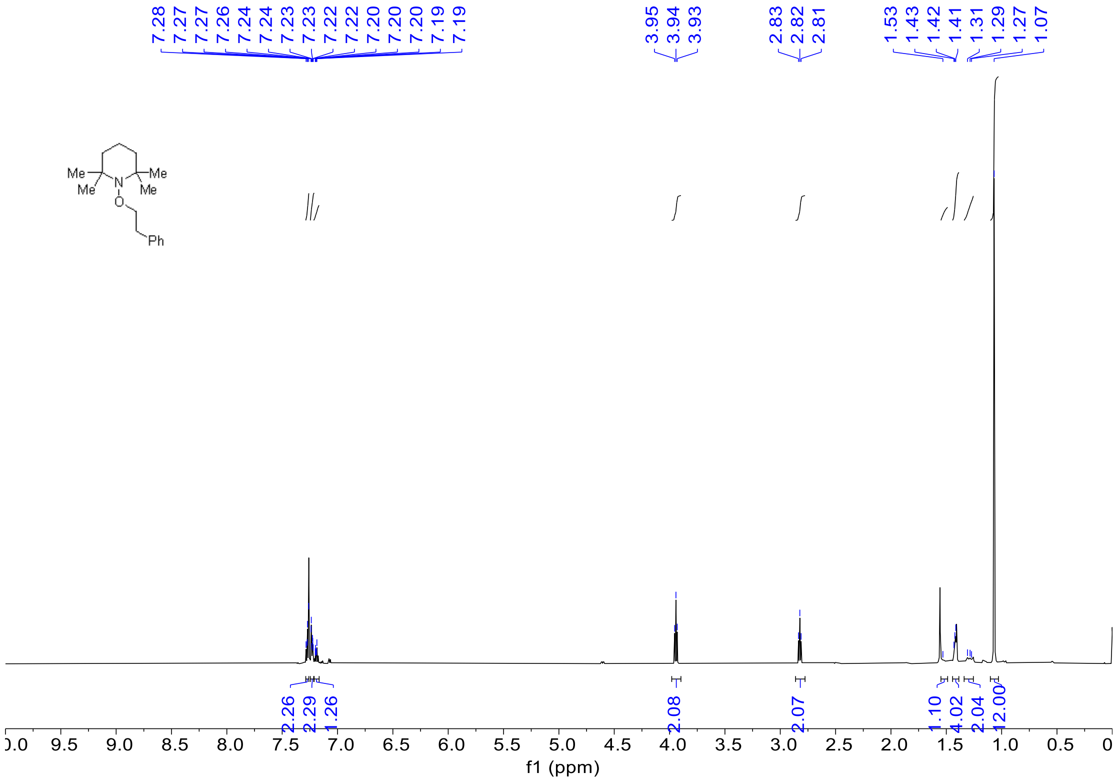

Supplementary Figure 332. ^{1}H NMR spectrum (600 MHz, $CDCl_3$) of **18**

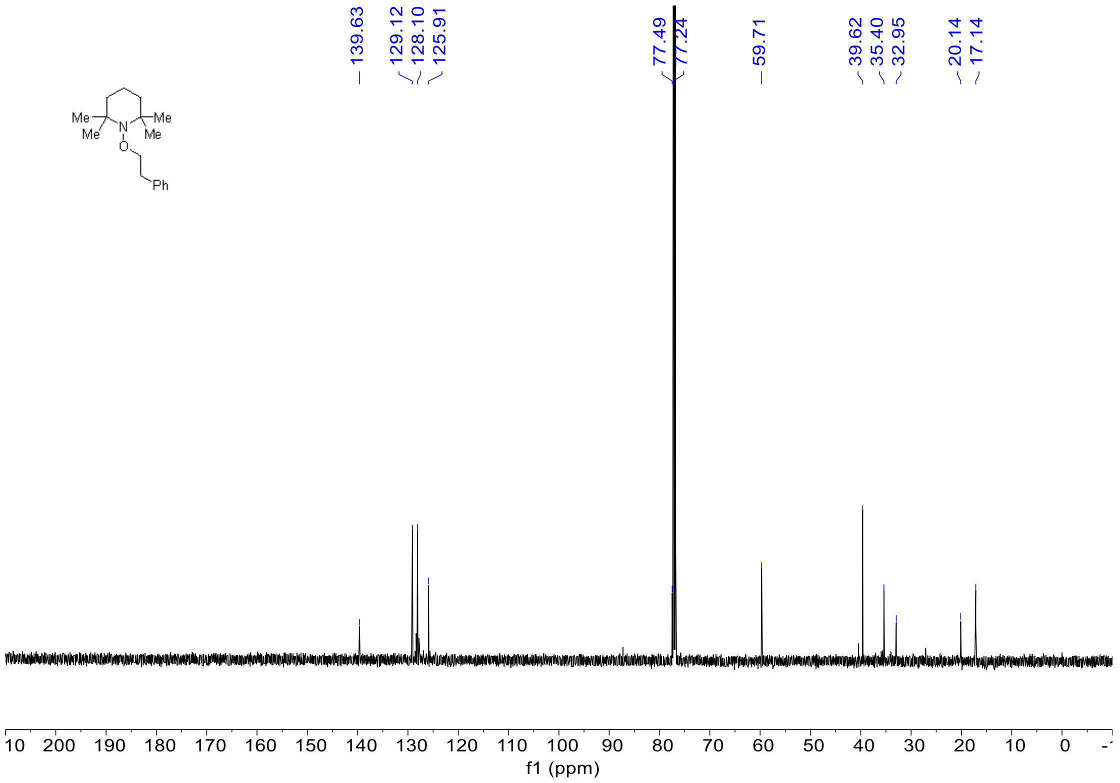

Supplementary Figure 333. ^{13}C NMR spectrum (151 MHz, $CDCl_3$) of **18**

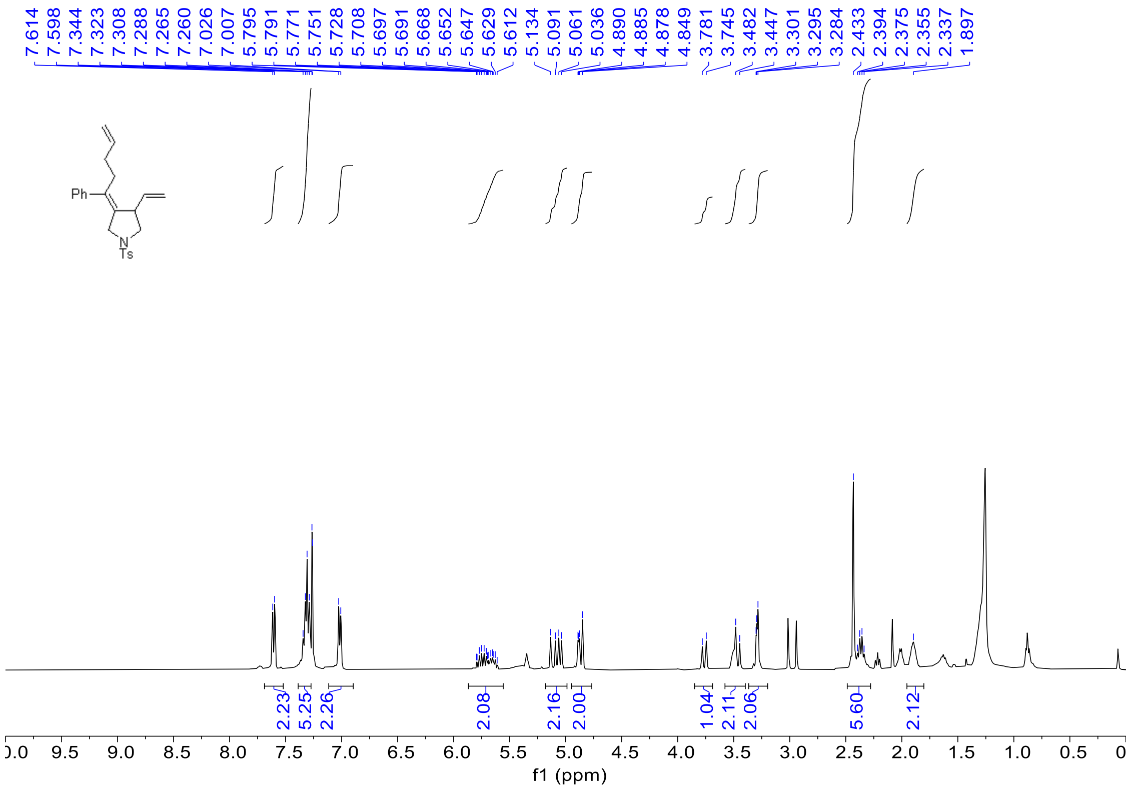

Supplementary Figure 334. ^{1}H NMR spectrum (400 MHz, $CDCl_3$) of **6at**

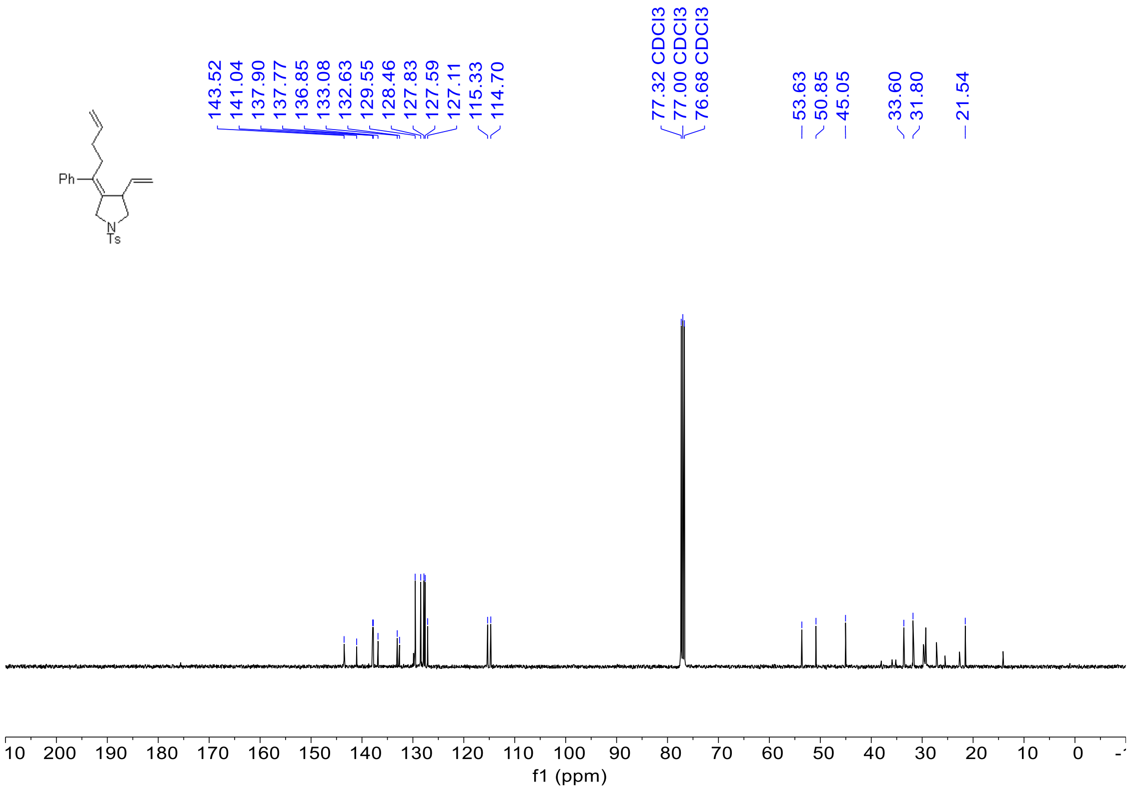

Supplementary Figure 335. ^{13}C NMR spectrum (100 MHz, $CDCl_3$) of **6at**

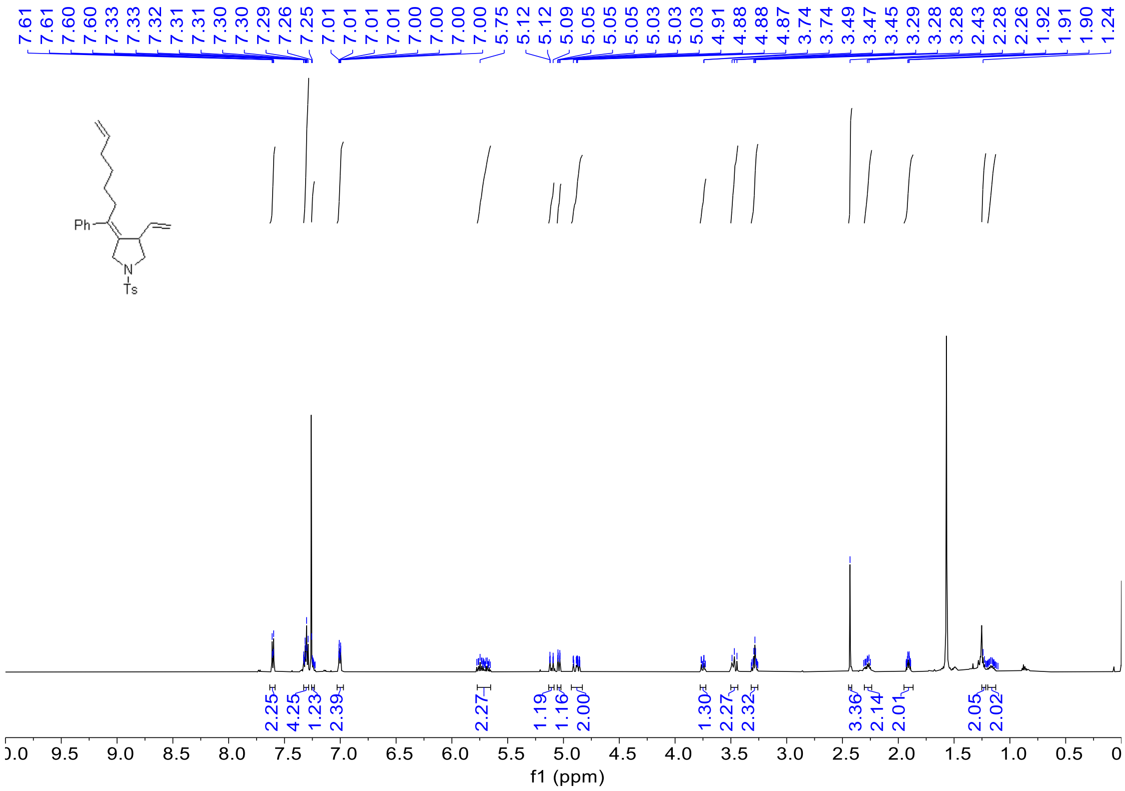

Supplementary Figure 336. ^{1}H NMR spectrum (600 MHz, $CDCl_3$) of **6au**

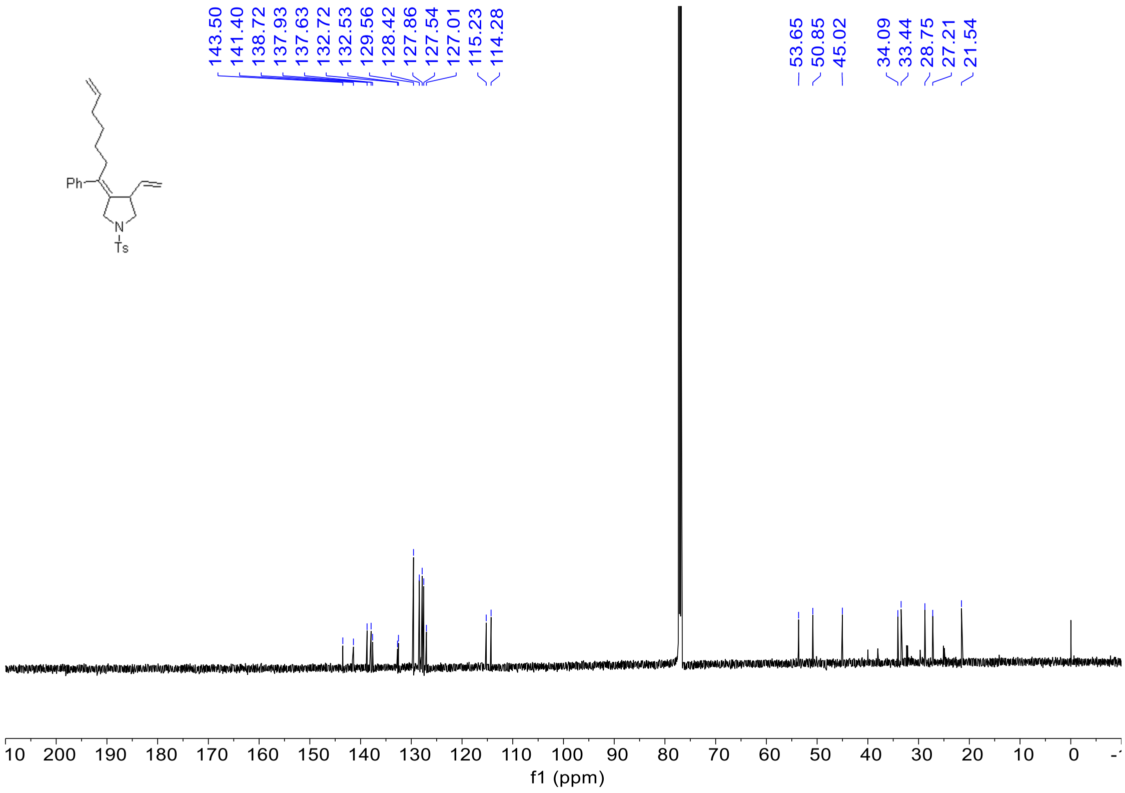

Supplementary Figure 337. ^{13}C NMR spectrum (151 MHz, $CDCl_3$) of **6au**

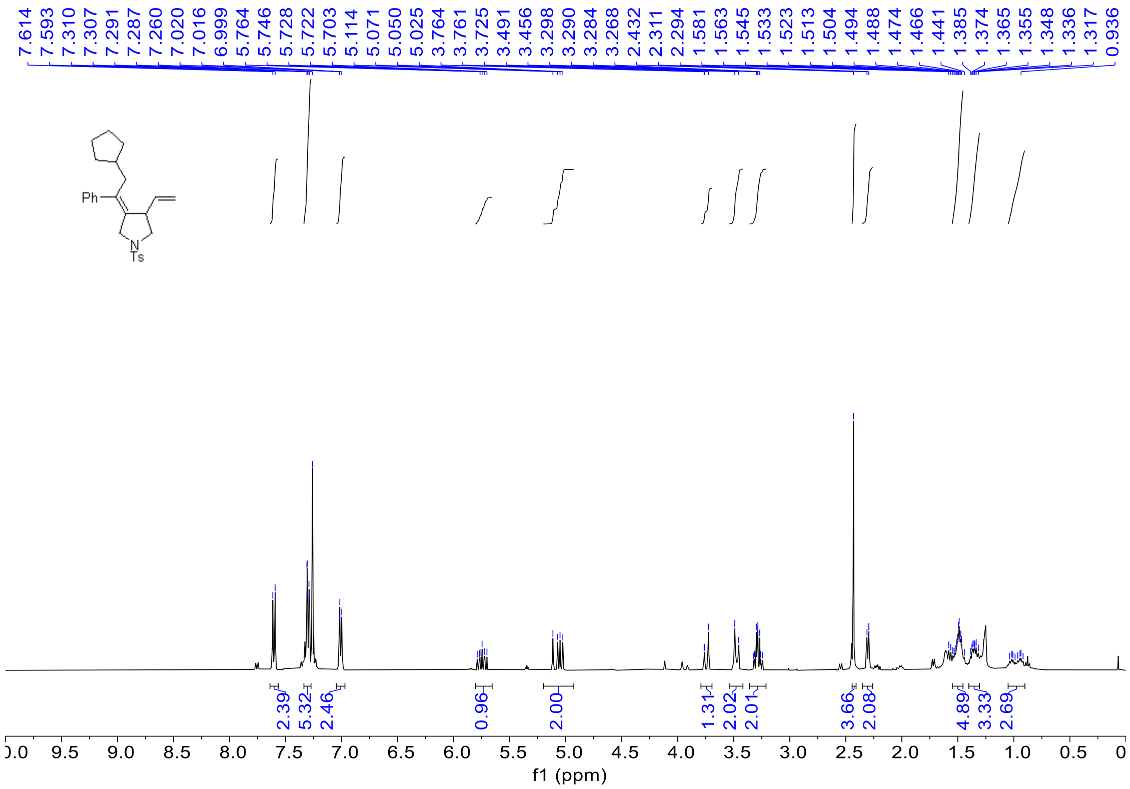

Supplementary Figure 338. ^{1}H NMR spectrum (400 MHz, $CDCl_3$) of **6au'**

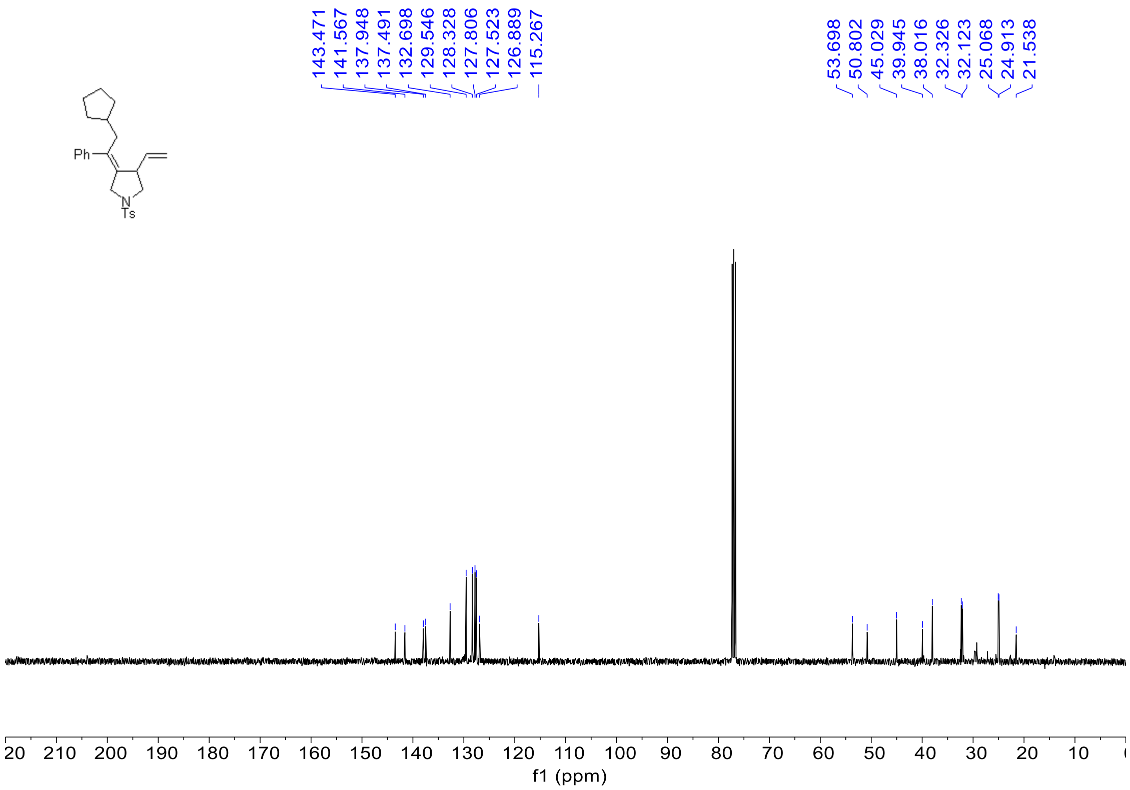

Supplementary Figure 339. ^{13}C NMR spectrum (100 MHz, $CDCl_3$) of **6au'**

13. Supplementary References

1. (a) Marquez, I. R.; Millan, A.; Campana, A. G.; Cuerva, J. M. Cp_2TiCl-catalyzed highly stereoselective intramolecular epoxide allylation using allyl carbonates. *Org. Chem. Front.* **2014**, *1*, 373-381. (b) Shu, X.; Schienebeck, C. M.; Li, X.; Zhou, X.; Song, W.; Chen, L.; Guzei, I. A.; Tang, W. Rhodium-Catalyzed Stereoselective Intramolecular [5+2] Cycloaddition of 3-Acyloxy 1,4-Enyne and Alkene. *Org. Lett.* **2015**, *17*, 5128-5131.

2. Zhao, L.; Lu, X.; Xu, W. Palladium(II)-Catalyzed Enyne Coupling Reaction Initiated by Acetoxypalladation of Alkynes and Quenched by Protonolysis of the Carbon-Palladium Bond. *J. Org. Chem.* **2005**, *70*, 4059-4063.

3. Zhou, Q.; Srinivas, H. D.; Zhang, S.; Watson, M. P. Accessing both retention and inversion pathways in stereospecific, nickel-catalyzed miyaura borylations of allylic pivalates. *J. Am. Chem. Soc.* **2016**, *138*, 11989-11995.

4. Muncipinto, G.; Kaya, T.; Wilson, J. A.; Kumagai, N.; Clemons, P. A.; Schreiber, S. L. Expanding Stereochemical and Skeletal Diversity Using Petasis Reactions and 1,3-Dipolar Cycloadditions. *Org. Lett.* **2010**, 12, 5230-5233.

5. Mohapatra, D. K.; Umamaheshwar, G.; Rao, R. N.; Rao, T. S.; R, S. K.; Yadav, J. S. Total Synthesis of Ivorenolide A Following Base Induced Elimination Protocol. *Org. Lett.* **2015**, *17*, 979-981.

6. (a) Tsukamoto, H.; Ueno, T.; Kondo, Y. Palladium(0)-Catalyzed Alkylative Cyclization of Alkynals and Alkynones: Remarkable trans-Addition of Organoboronic Reagents. *J. Am. Chem. Soc.* **2006**, *128*, 1406-1407. (b) Tanaka, R.; Noguchi, K.; Tanaka, K. Rhodium-catalyzed asymmetric reductive cyclization of heteroatom-linked 5-alkynals with heteroatom-substituted acetaldehydes. *J. Am. Chem. Soc.* **2010**, *132*, 1238-1239. (c) Huang, L.; Yang, H.; Zhang, D.; Zhang, Z.; Tang, X. Xu, Q.; Shi, M. Gold-Catalyzed Intramolecular Regio- and Enantioselective Cycloisomerization of 1,1-Bis(indolyl)-5-alkynes. *Angew. Chem. Int. Ed.* **2013**, *52*, 6767-6771.

7. Chi, X.; Meng, L.; Pang, Q.; Guo, L.; Liu, Q.; Zhao, P.; Zhang, D.; Sun, F.; Li, X.; Liu, H. Palladium-Catalyzed Domino Process to Construct 2,3,9,9a-Tetrahydro-1H-

Fluorene Derivatives: Transient σ-Alkyl Palladium(II) Complex Mediated C(sp^2)-H Bond Activation. *Asian J. Org. Chem*. **2019**, *8*, 2201-2204.

8. Holstein, P. M.; Dailler, D.; Vantourout, J.; Shaya, J.; Millet, A.; Baudoin, O. Synthesis of Strained β-Lactams by Palladium(0)-Catalyzed C(sp^3)-H Alkenylation and Application to Alkaloid Synthesis. *Angew. Chem. Int. Ed.* **2016**, *55*, 2805-2809.

9. Liu, J.; Ren, Q.; Zhang, X.; Gong, H. Preparation of Vinyl Arenes by Nickel-Catalyzed Reductive Coupling of Aryl Halides with Vinyl Bromides. *Angew. Chem. Int. Ed.* **2016**, *55*, 15544-15548.
